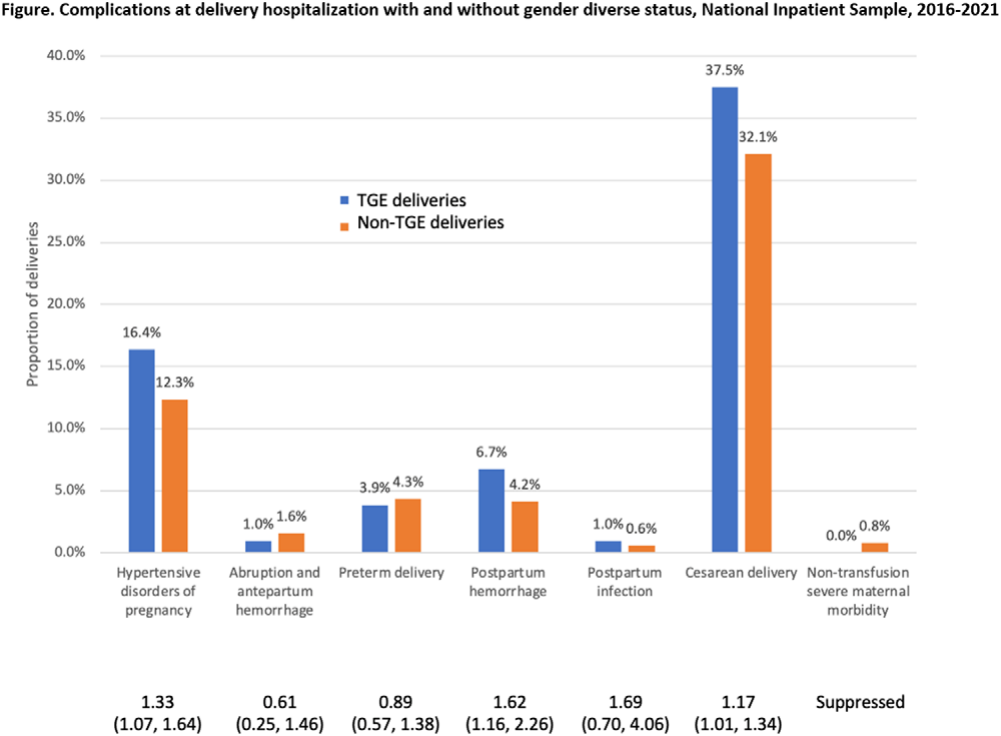# 2024 SMFM Global Congress Abstracts

**DOI:** 10.1002/pmf2.12045

**Published:** 2025-03-22

## ORAL CONCURRENT SESSION 1 ‐ FETAL, AMNIOTIC, AND PLACENTAL COMPLICATIONS

Thursday, 26 September 2024 14:45–17:15

## 1 | Prospective individual participant data meta‐analysis of pessary trials for preterm birth prevention (PROMPT) in twins

Christophe Vayssiere^1^

^1^Department of Obstetrics, Gynecology, Reproduction, Paule de Viguier Hospital, CHU Toulouse, France, Toulouse, Midi‐Pyrenees

16:00–16:15

**Objective**: To determine if cervical pessary use in the second trimester compared with no pessary in twin pregnancies with a short transvaginal ultrasound (TVU) cervical length (CL) reduces the risk of fetal death or preterm delivery using a prospectively planned Individual Participant Data Meta‐Analysis (IPDMA).

**Study Design**: We performed a prospectively planned Individual Participant Data Meta‐Analysis (IPD‐MA) (PROSPERO ID CRD42018067740) of four randomized trials. This analysis included enrolled individuals with a twin pregnancy between 16‐23 weeks gestation and TVU CL less than 30 mm who were randomized to pessary or no pessary. The primary outcome was a composite of fetal death or preterm delivery less than 32 weeks gestation (secondary neonatal and maternal outcomes listed in Table 1). Prespecified subgroup analyses were performed for type of pessary, CL, obstetrical history, gestational age (GA) at randomization, chorionicity, and randomization period. Log‐binomial or linear regression models were used to estimate relative risks or mean differences and their 95% confidence intervals (CI), as appropriate. A generalized estimating equations framework with exchangeable correlation structure was used to account for twins. Treatment by trial interactions were evaluated and random effects for trial were considered.

**Results**: 499 participants (998 neonates) were included from the four trials, with 257 (52%) in the pessary group and 242 (48%) in the no pessary group. There was no difference in the primary outcome of fetal death or preterm delivery before 32 weeks (aRR, 95% CI: 0.83, 0.63‐1.09) and no differences in maternal outcomes. Among those with monochorionic twins, there was a greater risk of genital or urinary tract infection in the pessary group compared with no pessary group (14/55, 25% vs. 2/56, 4%; aRR, 95% CI 7.92, 1.93–32.6). There were no differences in any other secondary outcomes (Table 1).

**Conclusion**: In a prospectively planned IPDMA, pessary use in individuals with twin pregnancy and short cervix did not have benefit and was associated with some harm.

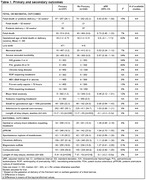

## 2 | Placenta in a dish

Lior Kashani Ligumsky^1^, Anhyo Jeong^2^, Haley Marks^3^, Guadalupe Martinez^1^, Davis Wachtell^3^, Angela Desmond^1^, Laurent Bentolila^3^, Deborah Krakow^1^, Yalda Afshar^1^

^1^University of California, Los Angeles, Los Angeles, California; ^2^Division of Maternal Fetal Medicine, Department of Obstetrics and Gynecology; David Geffen School of Medicine, University of California, Los Angeles, California; ^3^University of California, Los Angeles, University of California, Los Angeles, California

16:15–16:30

**Objective**: Placenta accreta spectrum (PAS) disorders are pregnancy complication associated with significant morbidity because of excessive adherence of placenta after birth. Despite robust clinical characterization, the pathophysiology is poorly understood. Our recent findings showed elevated levels of collagen in the decidua region at both the RNA and protein levels as well as disorganized in both surgical specimens and a PAS mouse model, have revealed collagen disorganization and alterations in microstructural alignment at the site of placental adherence. Building upon these observations, we hypothesize that the cesarean scar contributes to the formation of a disorganized fibrotic scaffold, accompanied by fundamental defects in the decidua. Our objective is to delineate changes in collagen architecture at the site of the scar using an in‐vitro PAS co‐culture model.

**Study Design**: Human uterine fibroblasts were decidualized (E2, MPA, cAMP) in‐vitro and underwent a wound (uterine scar). Trophoblasts were introduced and utilizing life cell imaging with co‐culture cell migration, invasion, and characteristics were assessed in the co‐culture system with and without collagen matrix.

**Results**: In our in‐vitro co‐culture model of PAS, a collagen matrix enhances the wound healing process and facilitates trophoblast distribution following a scar (wound) on decidualized fibroblasts. Additionally, we utilized fluorescence lifetime imaging microscopy (FLIM) to visualize collagen in the extracellular matrix produced by human fibroblast cells, rather than derived from trophoblast cells.

**Conclusion**: Disrupted collagen architecture at the uterine scar is a hallmark of PAS and site of trophoblast adherence. A paradigm shift in the biology of PAS highlights the scarred decidua creating a permissive environment for abnormal placentation. This in vitro model has the potential to identify therapeutic intervention and surgical strategies for the treatment and more so, prevention of PAS disorders.

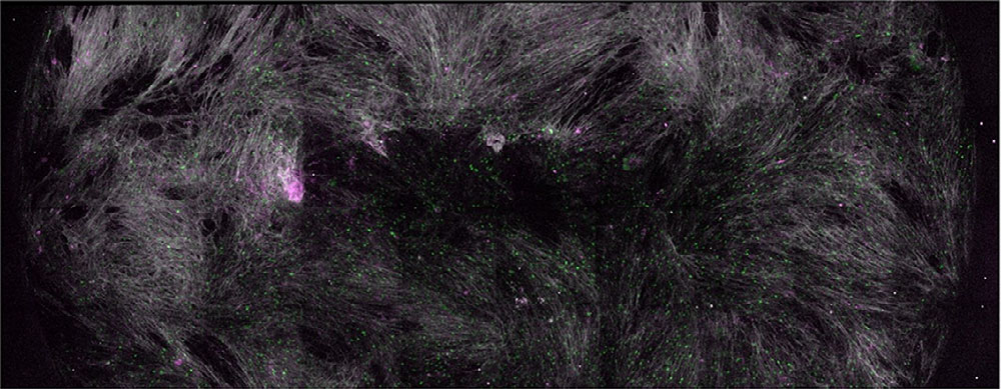

## 3 | Quantitative cervicovaginal fluid fetal fibronectin: Liquid biopsy for intra‐amniotic inflammation

Puntabut Warintaksa^1^, Roberto Romero^2^, Waranyu Lertrat^1^, Nutnaree Yuenyongdechawat^1^, Supakorn Chaiyakarn^1^, Pisut Pongchaikul^3^, Pornpun Vivithanaporn^3^, Piya Chaemsaithong^4^

^1^Faculty of Medicine Ramathibodi Hospital, Mahidol University, ratchathewi, Krung Thep; ^2^Pregnancy Research Branch, Division of Obstetrics and Maternal‐Fetal Medicine, Division of Intramural Research, Eunice Kennedy Shriver NICHD, NIH, DHHS, Bethesda, Maryland, USA, Bethesda, Maryland; ^3^Faculty of Medicine Ramathibodi Hospital, Mahidol University, Samut Prakan, Samut Prakan; ^4^Faculty of Medicine Ramathibodi Hospital, Mahidol University, Bangkok, Krung Thep

16:30–16:45

**Objective**: Intra‐amniotic inflammation is causally linked to spontaneous preterm labor. Cervicovaginal fluid fetal fibronectin is a widely‐used biomarker for spontaneous preterm labor. The aims of this study are to determine: 1)whether quantitative cervicovaginal fluid fetal fibronectin test can be used to identify the presence of intra‐amniotic inflammation in women presenting with spontaneous preterm labor and intact membranes; and 2)appropriate cut‐off value of cervicovaginal fluid fetal fibronectin concentration for the identification of intra‐amniotic inflammation.

**Study Design**: A prospective cohort study included 78 patients with preterm labor and intact membranes who had a sample collected for quantitative cervicovaginal fluid fetal fibronectin measurement and underwent transabdominal amniocentesis. Intra‐amniotic inflammation was defined as an amniotic fluid interleukin‐6 (IL‐6) concentration ≥2.6 ng/mL. The results of cervicovaginal fluid fetal fibronectin and amniotic fluid IL‐6 concentration were blinded to clinicians. The diagnostic indices of cervicovaginal fluid fetal fibronectin test for the identification of intra‐amniotic inflammation were calculated.

**Results**: 1)Frequency of intra‐amniotic inflammation was 26.9% (21/78); 2)the higher cervicovaginal fluid fetal fibronectin concentration, the greater the risk of intra‐amniotic inflammation (*p* < 0.001); 3)cervicovaginal fluid fetal fibronectin concentration ≥125 ng/mL had an area under the curve of 0.91 (95% CI: 0.83‐0.96) **(Figure 1)** for the identification of intra‐amniotic inflammation with 100% sensitivity, 82.46% specificity and a positive likelihood ratio of 5.7; 4)a cervicovaginal fluid fetal fibronectin cut‐off of 125 ng/mL had a significant higher predictive performance than the traditional cut‐off (50 ng/mL) for the identification of intra‐amniotic inflammation.

**Conclusion**: Quantitative cervicovaginal fluid fetal fibronectin with a cut‐off of 125 ng/mL had high sensitivity, specificity and positive likelihood ratio for the identification of intra‐amniotic inflammation in women presenting with spontaneous preterm labor and intact membranes. 

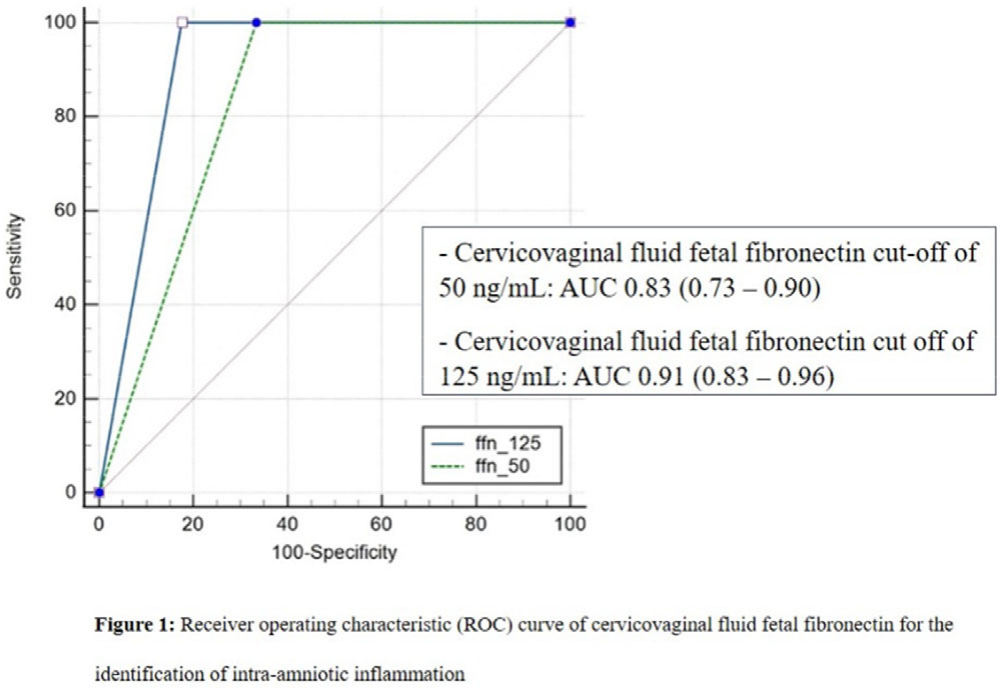

## 4 | Postpartum ultrasound evaluation of uterine caesarean scar

Maria Ivan^1^, Amrita Banerjee^1^, Charlotte Colley^1^, Davor Jurkovick^1^, Anna L. David^2^, Raffaele Napolitano^3^

^1^University College London Hospitals NHS Trust, London, England; ^2^Elizabeth Garrett Anderson Institute for Women's Health, University College London, London, England; ^3^University College London Hospital, University College London, England

16:45–17:00

**Objective**: To investigate the relationship between maternal factors, peripartum events and ultrasound features of uterine caesarean delivery scars (CDS).

**Study Design**: A prospective observational cohort study, ethically approved, was conducted at University College London Hospital, UK (2021‐2022) on women who underwent caesarean birth at 4‐10 cm cervical dilatation. Transvaginal ultrasound assessments were performed 4‐12 months postpartum to assess scar position and morphology (including niche dimensions and healing ratio, defined as residual/adjacent myometrial thickness). Multivariate binomial logistic and regression analysis were performed using IBM SPSS Statistics v29.

**Results**: 97% (90/93) participants had visualised CDS, with 38% (34/90) showing a niche of depth ≥2mm and 26.7% (24/90) a healing ratio ≤0.5. Each cm increase in cervical dilatation was associated with the scar positioned 0.88mm lower in the uterus/cervix (95% CI −1.14, −0.62; *p* < 0.001). Similarly, for every cm of descent of the fetal part in the maternal pelvis, the scar was positioned 1.5mm lower (95% CI −2.33, −0.71; *p* < 0.001). Women undergoing advanced labour CD (8‐10 cm) were nearly 8 times more likely to have a scar located at or below the internal os compared to those in early labour (4‐7 cm), RR 7.77 (95% CI 2.586‐23.39; *p* < 0.001). CDS located below the internal cervical os demonstrated significantly higher rates of niche formation and low healing ratios compared to those located above or at the internal cervical os (50% vs 29%, *p* = 0.04 and 42% vs 15%, *p* = 0.005, respectively). Increased uterine artery vascular resistance, fetal birthweight >4000g and cervical scar were significantly associated with an increased risk of niche development and reduced healing ratio (*p* < 0.05).

**Conclusion**: Maternal and peripartum factors significantly impact CDS healing and position, with scars located below the internal cervical os indicating inferior healing outcomes. These findings may explain the increased risk of spontaneous preterm birth in subsequent pregnancies, potentially attributed to the impact on cervical function.

## 5 | Ampicillin vs. Ampicillin and Gentamicin for term prolonged rupture of membranes: A randomized controlled trial

Raneen Abu Shqara^1^, Daniel Glikman^2^, Gabriela Goldinfeld^3^, Lior Lowenstein^1^, Olga Braude^4^, Silas Assy^4^, Dunia Hassan^4^, Inshirah Sgayer^1^, Nadir Ganem^4^, Marian matanis^5^, Enav Yefet^5^, Maya Frank Wolf^6^

^1^Galilee Medical Center, Nahariya, HaZafon; ^2^Azrieli Faculty of Medicine, Bar Ilan University, Safed, Israel., Safed, HaZafon; ^3^Bar‐Ilan university, Bar‐Ilan University, HaZafon; ^4^Raya Strauss Wing of Obstetrics and Gynecology Galilee Medical Center, Nahariya, HaZafon; ^5^Tzafon Medical Center, Poriya, HaZafon; ^6^Galilee Medical Center, Naharyia, HaZafon

17:00–17:15

**Objective**: Prolonged term rupture of membranes (ROM) is a risk factor for maternal and neonatal infectious morbidity. Ampicillin is indicated in women with unknown group B streptococcus (GBS) status and prolonged ROM (>18h). While GBS related peripartum infections have declined, ampicillin‐resistant Enterobacteriaceae infections have emerged, changing the epidemiology of perinatal infections. We compared two antibiotic regimens in women with term prolonged ROM.

**Study Design**: This randomized‐controlled trial was conducted between November, 2022 and March, 2024. A total of 204 women at term with unknown GBS status and ROM were randomized to two antibiotic prophylactic protocols: ampicillin alone (*n* = 102) vs. ampicillin and gentamicin (*n* = 102). Maternal outcomes were: intrapartum fever, clinical chorioamnionitis, and a composite postpartum outcome of fever, endometritis, wound infection, antibiotic treatment, and hospitalization >5 days. The neonatal outcomes were neonatal intensive unit admission, and a composite neonatal outcomes of sepsis work‐up, respiratory distress syndrome, need for respiratory support, and hospitalization >5 days. Intention to treat analysis was performed.

**Results**: Intrapartum fever and clinical chorioamnionitis were higher in the ampicillin group than in the ampicillin and gentamicin group: 18% vs 8%; *p* value = .036 and 8% vs 0%; *p* = .017, respectively. The pathogen distribution of chorioamniotic cultures differed between the groups (*p* = .001); Enterobacteriaceae spp. were identified in 51% of the ampicillin‐group and in 20% of the ampicillin and gentamicin group (*p* < .001). The composite postpartum maternal and neonatal outcome were higher in the ampicillin group than in the ampicillin and gentamicin group: 14% vs 5%; *p* = .030 and 22% vs. 11%, *p* = .036, respectively.

**Conclusion**: The use of prophylactic ampicillin alone vs. ampicillin and gentamicin, for term prolonged ROM, resulted in a higher proportion of intrapartum fever, clinical chorioamnionitis, postpartum maternal and neonatal adverse outcomes. It is time to reconsider prophylactic antibiotics treatment in prolonged ROM.

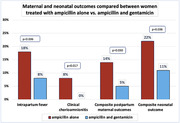

## ORAL CONCURRENT SESSION 2 ‐ HYPERTENSION

Thursday, 26 September 2024 14:45–17:15

## 6 | The role of CD20+ B cells in mediating hypertension during pregnancy

Nathan Campbell^1^, Miriam Hankins^2^, Owen T. Herrock^3^, Baoying Zheng^1^, Alexandra Demesa^1^, Ty Turner^1^, Evangeline Deer^1^, Lorena M. Amaral^1^, Rachael Morris^1^, Babbette LaMarca^1^

^1^University of Mississippi Medical Center, Jackson, Mississippi; ^2^University of Mississippi Medical Center, Jackson, MS; ^3^Department of Obstetrics and Gynecology, Institute of Clinical Sciences, Sahlgrenska Academy, University of Gothenburg, Gothenburg, Vastra Gotaland

16:00–16:15

**Objective**: Pre‐eclampsia (PE) is new‐onset hypertension after the 20^th^ week of pregnancy associated with organ dysfunction, endothelial dysfunction, and immune activation. We have previously shown that CD19+ B cell adoptive transfer causes a PE phenotype in athymic nude rats. CD19 is present on all B cells including plasma cells. CD20 is absent from plasma cells but is present on mature B2 and memory B cells. The objective of this study was to determine if CD20+ B cells, excluding plasma cells, cause maternal hypertension, fetal growth restriction, and endothelial dysfunction.

**Study Design**: Three hundred thousand placental CD20+ B Cells from normal pregnant (NP) or PE patients were isolated by magnetic separation and injected i.p. into nude athymic rats on gestational day (GD) 12. On GD18, carotid catheters were inserted. On GD19, mean arterial pressure (MAP) was measured and blood and tissues were collected. Preproendothelin (PPET) expression was quantified in kidney and placental tissues using RT‐PCR. A student's t‐test was used for statistical analysis.

**Results**: Participants whose placental B cells were used in this study had similar age, pre‐pregnancy BMI, and diastolic BP on admission. PE participants had elevated systolic BP at admission (135 ± 4 vs 120 ± 4 mmHg) and delivery (139 ± 6 vs 124 ± 3 mmHg) and diastolic BP at delivery (79 ± 4 vs 68 ± 2 mmHg). PE participants also had trending decreases in fetal weight (2621 ± 323 vs 3202 ± 102 g) and gestational age (36 ± 1 vs 39 ± 0 weeks) at delivery.

MAP increased from 107 ± 5 mmHg (*n* = 6) in the NP recipient rats to 123 ± 2 mmHg (*n* = 8, *p* < 0.01) in the PE recipient rats. Pup and placental weights were unchanged following adoptive transfer. Renal PPET expression increased by 2.78 ± 0.67 fold (ΔΔct) in PE recipient rats (*n* = 3, *p* < 0.05) compared to NP recipient rats (*n* = 4). Placental PPET expression was unchanged following adoptive transfer.

**Conclusion**: CD20+ B cells from PE women contribute to hypertension and endothelial dysfunction during pregnancy. This data supports the importance of B cells in the hypertension in PE.

## 7 | Evaluation of early risk assessment of developing preterm preeclampsia in a Swedish population – The EPES‐study

Núria Mans Gallart^1^, Sara Carlhäll^2^, Eric Hildebrand^3^, Daniel Axelsson^1^, Caroline Lilliecreutz^4^

^1^Department of Obstetrics and Gynecology, Ryhov County Hospital, Jönköping, Sweden and Department of Biomedical and Clinical Sciences, Linköping University, Linköping, Sweden, Jönköping, Jonkopings Lan; ^2^Dept of Obstetrics and Gynecology and Dept of Biomedical and Clinical Sciences, Linköping University, Linköping, Sweden, Linköping University, Linköping, Ostergotlands Lan; ^3^Department of Obstetrics and Gynaecology and Department of Biomedical and Clinical Sciences, Linköping University, Linköping, Sweden., Linköping, Ostergotlands Lan; ^4^Department of Obstetrics and Gynaecology and Department of Biomedical and Clinical Sciences, Linköping University, Linköping, Sweden, Linköping, Ostergotlands Lan

16:15–16:30

**Objective**: The Fetal Medicine Foundation (FMF) method combines anamnestic risk factors, biochemical and biophysical tests between gestational weeks (gw) 11+0‐13+6 to assess the risk of developing preterm preeclampsia (PE). It has been demonstrated that the use of the FMF method in combination with 150 mg acetylsalicylic acid treatment in high‐risk women has reduced the prevalence of preterm PE in a pre‐selected study‐population. To our knowledge, this method has not been assessed in an unselected pregnant population before. This study aimed to evaluate the FMF method by comparing the prevalence of preterm birth combined with PE before and after the FMF method was introduced in a population based antenatal care setting.

**Study Design**: This was a multicenter, retrospective cohort study conducted in five obstetric units in Sweden. The PreFMF‐cohort (*n* = 31063), including pregnant women at the participating study sites before the introduction of the FMF‐method (2012 to 2017‐2019) was compared with the FMF‐cohort (*n* = 30777), with the FMF method in use (2018‐2020 to 2022). All women in the FMF‐cohort were offered the risk assessment. The cut off for high‐risk was determined to ≥ 1/100. Multivariable logistic regression analyses were performed accounting for time and adjustments were made for confounders.

**Results**: The women in the FMF‐cohort were older, had higher BMI and more often type 1 or 2 Diabetes Mellitus (*p* < 0.05). No significant difference in prevalence of women with preterm birth and PE between the PreFMF‐cohort (0.48%) and the FMF‐cohort (0.52%) was found (aOR 0.83; 95% CI 0.6‐1.1).

**Conclusion**: No reduction in prevalence of preterm birth combined with PE was found after the introduction of the FMF‐method in this unselected population, when analyzing the results as intention‐to‐treat. There were fewer cases in the group delivered at gw < 28+0 but not significant due to the small number of individuals. The low prevalence of premature PE before the introduction of the FMF‐method may have contributed to the results. Further studies are planned comparing the effect of the FMF‐method in contemporary cohorts.

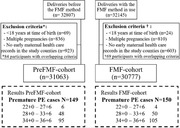

## 8 | Association between plasma HLA‐DR+ placental vesicles and preeclampsia: A pilot longitudinal cohort study

Marianna Onori^1^, Rita Franco^1^, Giuliana Beneduce^1^, Fabio Sannino^1^, Antonio Lanzone^2^, Giovanni Scambia^3^, Nicoletta Disimone^4^, Chiara Tersigni^5^

^1^Università Cattolica del Sacro Cuore, Università Cattolica del Sacro Cuore, Lazio; ^2^Fondazione Policlinico Agostino Gemelli, IRCSS, Rome Italy, Rome, Lazio; ^3^IRCCS Fondazione Policlinico Agostino Gemelli, Rome, Lazio; ^4^Humanitas University, Milan, Lombardia; ^5^IRCCS Fondazione Policlinico Agostino Gemelli, IRCCS Fondazione Policlinico Agostino Gemelli, Lazio

16:30–16:45

**Objective**: Reduced maternal‐fetal immunological tolerance is a possible trigger of poor placentation and pre‐eclampsia (PE). To allow fetal immunological escape from the semi‐allogenic mother, there is a complete suppression of Human Leukocyte Antigens (HLA) class II in the human placenta. Aberrant antigen expression HLA‐DR has been observed in the syncytiotrophoblast of PE patients. Aim of this study was to analyse plasma levels of HLA‐DR+ syncytiotrophoblast‐derived extracellular vesicles (STEVs) during the three trimesters of pregnancy in relation to the subsequent onset of PE.

**Study Design**: Pregnant women, recruited prospectively in a consecutive fashion during the first trimester screening, underwent venous blood sampling during the 3 trimesters of pregnancy. STEVs were collected from plasma via ultracentrifugation (120,000g) and characterized by Western blot, nanotracking analysis and flow cytometry for the expression of placental alkaline phosphatase (PLAP), a placental‐derived marker, and HLA‐DR. Clinical and laboratory data were analysed using Student's T test or Mann‐Whitney U test, according to type of variables.

**Results**: Out of 107 women recruited, 10 developed PE. A significant of circulating STEVs were detected in all three trimesters of pregnancy in maternal plasma (about 20% of all circulating vesicles) with a zenith in the second trimester. Significantly higher plasma levels of HLA‐DR+ STEVs were observed in women who developed PE, compared to the no‐PE women, in all the three trimesters of pregnancy. Women with circulating HLA‐DR+ STEVs during the first trimester show a six‐fold increased risk to develop PE during pregnancy compared to women with HLA‐DR‐ STEVs (RR 5.8; 95% CI 2.5‐11.9; *p* < 0.0001).

**Conclusion**: More research is needed to investigate HLA‐DR+STEVs as potential circulating early biomarkers of PE.

## 9 | Neuroinflammation and blood‐brain barrier injury in eclampsia

Valentina Bucher^1^, Owen T. Herrock^1^, Sonja Schell^2^, Jacqui Visser^2^, Henrik Imberg^3^, Henrik Zetterberg^4^, Kaj Blennow^5^, Sue Walker^6^, Stephen Tong^7^, Joakim Ek^8^, Catherine Anne. Cluver^9^, Lina Bergman^10^

^1^Department of Obstetrics and Gynecology, Institute of Clinical Sciences, Sahlgrenska Academy, University of Gothenburg, Gothenburg, Vastra Gotaland; ^2^Department of Obstetrics and Gynecology, Stellenbosch University, Cape Town, Western Cape; ^3^Statistiska Konsultgruppen, Gothenburg, Sweden; Department of Mathematical Sciences, Chalmers University of Technology and University of Gothenburg, Gothenburg, Vastra Gotaland; ^4^Institute of Neuroscience and Physiology, Department of Psychiatry and Neurochemistry, Sahlgrenska, Academy, University of Gothenburg, Mölndal, Vastra Gotaland; ^5^Institute of Neuroscience and Physiology, Department of Psychiatry and Neurochemistry, Sahlgrenska, Academy, University of Gothenburg, Möldnal, Vastra Gotaland; ^6^Department of Obstetrics, Gynaecology and Newborn Health, University of Melbourne, Melbourne, Victoria; ^7^Department of Obstetrics, Gynaecology and Newborn Health, University of Melbourne, Heidelberg, Victoria, Victoria; ^8^Institute of Neuroscience and Physiology, Sahlgrenska Academy, University of Gothenburg, Gothenburg, Vastra Gotaland; ^9^Stellenbosch University, Stellenbosch University, Western Cape; ^10^Department of Obstetrics and Gynecology, Sahlgrenska Academy, University of Gothenburg, Gothenburg, Vastra Gotaland

16:45–17:00

**Objective**: Cerebral complications secondary to preeclampsia like eclampsia are among the most common causes of direct maternal mortality. Proposed underlying mechanisms include involvement of the blood brain barrier (BBB) but evidence is mainly derived from animal models. Improved knowledge of the underlying pathophysiology could open for new treatment strategies to protect the maternal brain. Therefore, the aim of this study was to investigate presence of neuroinflammation and BBB disruption in women with eclampsia and preeclampsia compared to women with normotensive pregnancies.

**Study Design**: This study included women at admission to labor ward at Tygerberg University Hospital in Cape Town, South Africa. At time of spinal anesthesia for a caesarian section, the first portion of cerebrospinal fluid was collected, together with a paired plasma sample and frozen for later analyses. In total, 129 women were included. Of these, 11 women had eclampsia, 19 women had preeclampsia with severe end‐organ complications, 86 women had preeclampsia without end‐organ complications, and 13 women had a normotensive pregnancy. Markers of neuroinflammation were analyzed in cerebrospinal fluid (CSF) utilizing a multiplex screening assay. Injury of the BBB was assessed by the ratio of albumin concentrations in CSF and plasma and by levels of the BBB tight junction protein claudin‐5 in CSF.

**Results**: Women with eclampsia demonstrated a breached BBB, reflected by an increased albumin ratio and increased concentrations of claudin‐5 in the CSF. In addition, women with eclampsia had increased levels of proinflammatory markers in the CSF compared to both women with preeclampsia and normotensive women.

**Conclusion**: Women with eclampsia demonstrate signs of endothelial distress and disruption of the BBB. This injury and/or altered cell‐signaling over the neurovascular unit might be underlying causes to the increased neuroinflammatory activity and development of cerebral edema and seizures in eclampsia.

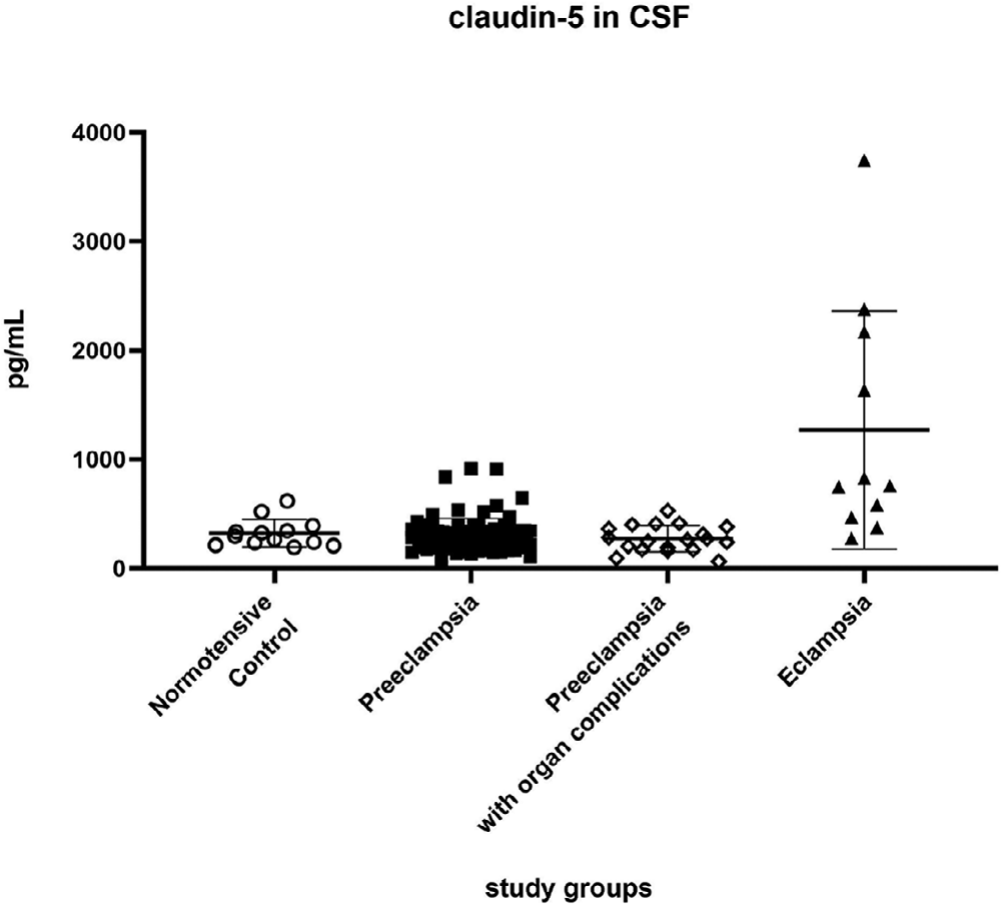

## 10 | Placental growth factor at 24‐28 weeks for aspirin discontinuation: POST‐HOC analysis of stoppre trial

Erika Bonacina^1^, Marta Dalmau^1^, Marta Ricart^2^, Manel Mendoza^1^, Anna Suy^3^, Elena Carreras^4^

^1^Vall d'Hebron University Hospital, Vall d'Hebron Hospital, Catalonia; ^2^Hospital Germans Trias i Pujol, Germans Trias i Pujol, Catalonia; ^3^Vall d'Hebron University Hospital, Barcelona, Catalonia; ^4^Hospital Vall d´Hebron, Hospital Vall d´Hebron, Catalonia

17:00–17:15

**Objective**: This study aims to evaluate the safety of discontinuing aspirin treatment at 24 to 28 weeks in women at high‐risk after first‐trimester combined screening for preeclampsia (PE) and normal placental growth factor (PlGF) levels at 24 to 28 weeks of gestation.

**Study Design**: This is a post hoc analysis of the StopPRE trial, conducted at nine Spanish maternity hospitals from September 2019 to September 2021. In the StopPRE trial, all high‐risk single pregnancies identified during first‐trimester screening for PE were treated with 150 mg daily aspirin. Out of 1,604 eligible women with soluble fms‐like tyrosine kinase‐1 to placental growth factor ratio (sFlt‐1/PlGF) ≤38 at 24‐28 weeks, 968 were randomly assigned in a 1:1 ratio to either continue aspirin until 36 weeks (control group) or discontinue it (intervention group). In this secondary analysis, only women with a PlGF ≥100 pg/mL at 24‐28 weeks were included. As in the StopPRE trial, the non‐inferiority margin was set at a 1.9% difference in preterm PE incidence between groups.

**Results**: Among 13,983 screened pregnant women, 1,984 (14.2%) were deemed high‐risk for preterm PE, of which 397 (20.0%) were ineligible, 636 declined participation, and 32 were excluded. Ultimately, 919 women with PlGF >100 pg/mL were randomized and included in this analysis. Preterm PE occurred in 0.9% of the intervention group (4 out of 465) and 1.5% of the control group (7 out of 454), indicating non‐inferiority of aspirin discontinuation. There were no significant differences between groups in adverse pregnancy outcomes before 37 weeks, or at < 34 weeks, or ≥37 weeks. Minor antepartum hemorrhage incidence was significantly lower in the intervention group (absolute difference, −5.96; 95% CI, ‐ 10.10 to −1.82).

**Conclusion**: Discontinuing aspirin treatment at 24‐28 weeks in women with PlGF ≥100 pg/mL was non‐inferior to continuing until 36 weeks in preventing preterm PE.

## ORAL CONCURRENT SESSION 3 ‐ RISK FACTORS FOR ADVERSE OUTCOMES

Friday, 27 September 2024 09:00–11:15

## 11 | The impact of aspirin on fetal growth in maternal diabetes–secondary analysis of the IRELAND RCT

Catherine Finnegan^1^, Patrick Dicker^2^, Denisa Asandei^3^, Mary Higgins^4^, Neil O'Gorman^5^, Mairead O'Riordan^6^, Fidelma Dunne^7^, Geraldine Gaffney^7^, Christine Newman^7^, Fionnuala M. M. McAuliffe^8^, Vineta Ciprike^9^, Elena Fernandez^10^, Fergal D. Malone^11^, Fionnuala M. Breathnach^3^

^1^RCSI Rotunda, RCSI Rotunda, Dublin; ^2^Royal College of Surgeons Ireland, Dublin, Dublin; ^3^RCSI Rotunda, Dublin, Dublin; ^4^UCD Dublin, Dublin, Dublin; ^5^Coombe Hopsital, Dublin, Dublin; ^6^UCC, Dublin, Dublin; ^7^University Hospital Galway, Dublin, Dublin; ^8^UCD Perinatal Research Centre, University College Dublin, Dublin 2, Dublin; ^9^Our Lady of Lourdes Hospital Drogheda, Dublin, Donegal; ^10^Rotunda Hospital Dublin, Dublin, Dublin; ^11^Royal College of Surgeons in Ireland, Dubiln, Dublin

09:45–10:00

**Objective**: Aspirin treatment during pregnancy is believed to exert a protective effect in the prevention of fetal growth restriction. However, the influence of aspirin on pregnancies prone to fetal overgrowth has not been determined. We sought to examine whether aspirin therapy exerted an influence on the fetal growth trajectory of pregestational diabetes (PGDM) pregnancies.

**Study Design**: The IRELAnD study, involving 126 PGDM patients across 6 centers, compared 150mg aspirin to placebo for a double‐blinded RCT. PGDM patients were randomly assigned aspirin or placebo from 11‐14 weeks’ gestation to 36 weeks. This sub‐study, with 106 participants (aspirin *N* = 54, placebo *N* = 52) and complete sonographic biometry, compared fetal growth trajectories between aspirin and placebo groups, defining macrosomia as AC or EFW >90th centile by the Fetal Medicine Foundation (FMF) standard. Large‐for‐gestational‐age (LGA) at birth was considered where birthweight (BW) for gestation exceeded the 90^th^ centile by the FMF standard. Statistical methods included chi‐square, t‐test, and median test analyses.

**Results**: 426 fetal ultrasound assessments were compared. Fetal macrosomia occurred in 57% and 54% of aspirin and placebo groups, respectively, at a mean (SD) gestation of 27.4 (5.8) weeks. Median [IQR] BW percentile was 92 [64,99] for the entire study group, and LGA BW occurred in 57% of aspirin‐treated pregnancies and 46% of the placebo group (*p* = 0.0282).No significant differences were observed in delivery‐related complications, NICU admission or hypoglycemia between groups. Antenatal detection rates for macrosomia were high >70%.

**Conclusion**: In a prospective RCT of PGDM pregnancies, aspirin‐treated patients exhibited increased rates of macrosomia and LGA, although this trend did not reach statistical significance. Fetal macrosomia is most reliably predicted sonographically between 27‐28 weeks. Aspirin therapy does not appear to substantially influence the fetal growth trajectory where the PGDM‐driven tendency toward fetal overgrowth is high.

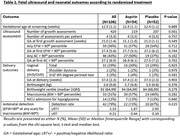

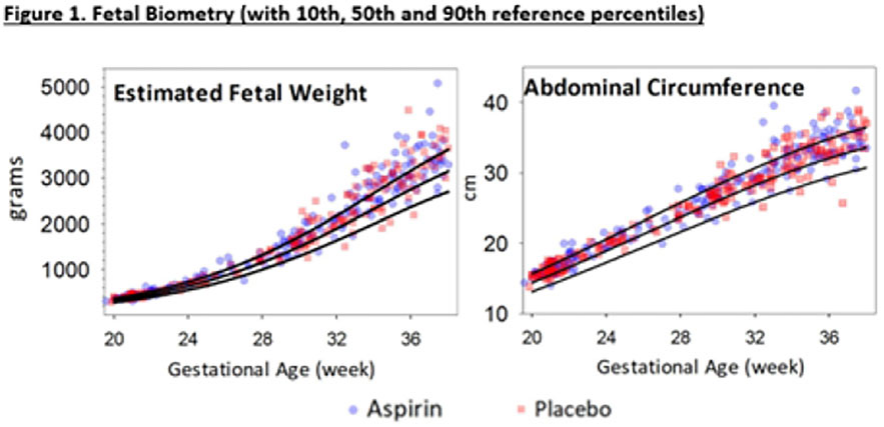

## 12 | Interpregnancy interval following missed abortion and the risk for preterm birth

Gal Bachar^1^, Yousef Abboud^2^, Naama Farago^2^, Naphtali Justman^1^, Nizar Khatib^2^, Yaniv Zipori^1^, Zeev Weiner^2^, Dana Vitner^1^

^1^Rambam Medical Center, Haifa, Hefa; ^2^Rambam Medical Health Center, Haifa, Hefa

10:00–10:15

**Objective**: A short inter‐pregnancy interval (IPI) of  <  18 months following a live birth, has been associated with adverse pregnancy outcome. This study aimed to evaluate whether a short IPI following a medically treated missed abortion (MA) poses similar perinatal risks in a subsequent pregnancy.

**Study Design**: The retrospective analysis included patients with history of an MA at up to 10 weeks of gestation, treated with misoprostol (prostaglandin E1) only, and with a documented subsequent live pregnancy, at a single tertiary medical center between 2010 and 2022. Patients who had surgical evacuation following failure of medical treatment were excluded. Patients were allocated into two groups: IPI ≤ 18 months and IPI > 18 months. The primary outcome was the risk for a spontaneous preterm birth (PTB) before 37 weeks of gestation in the consecutive pregnancy. Secondary outcomes included additional maternal and neonatal adverse outcomes.

**Results**: The cohort included 1,110 pregnancies: 430 (38.74%) patients with IPI  <  18 months and 680 (61.26%) patients with IPI > 18 months. The characteristics of the two groups were not significantly different. The rates of spontaneous PTB at < 37 and < 34 weeks of gestation were significantly higher in the short vs. long IPI cohort (16.28% vs. 7.06% and 6.74% vs. 5.0%, respectively, *p* < 0.05). These patients also had a higher risk for Cesarean delivery (31.63% vs. 23.34%, *p* = 0.005) and postpartum hemorrhage (PPH) (4.42% vs. 2.06%, *p* = 0.029) compared to patients with IPI > 18 months. The observed differences remained statistically significant even after adjusting for potential confounding variables using multiple regression analysis (maternal age, parity and number of PgE1 doses). No other significant differences in neonatal or maternal outcomes were noted.

**Conclusion**: Short IPI (≤ 18 months) following a medical treatment for early‐gestation MA may be associated with an increased risk of PTB, Cesarean delivery and PPH. These findings may be of particular importance when counseling patients who desire another pregnancy following a MA.

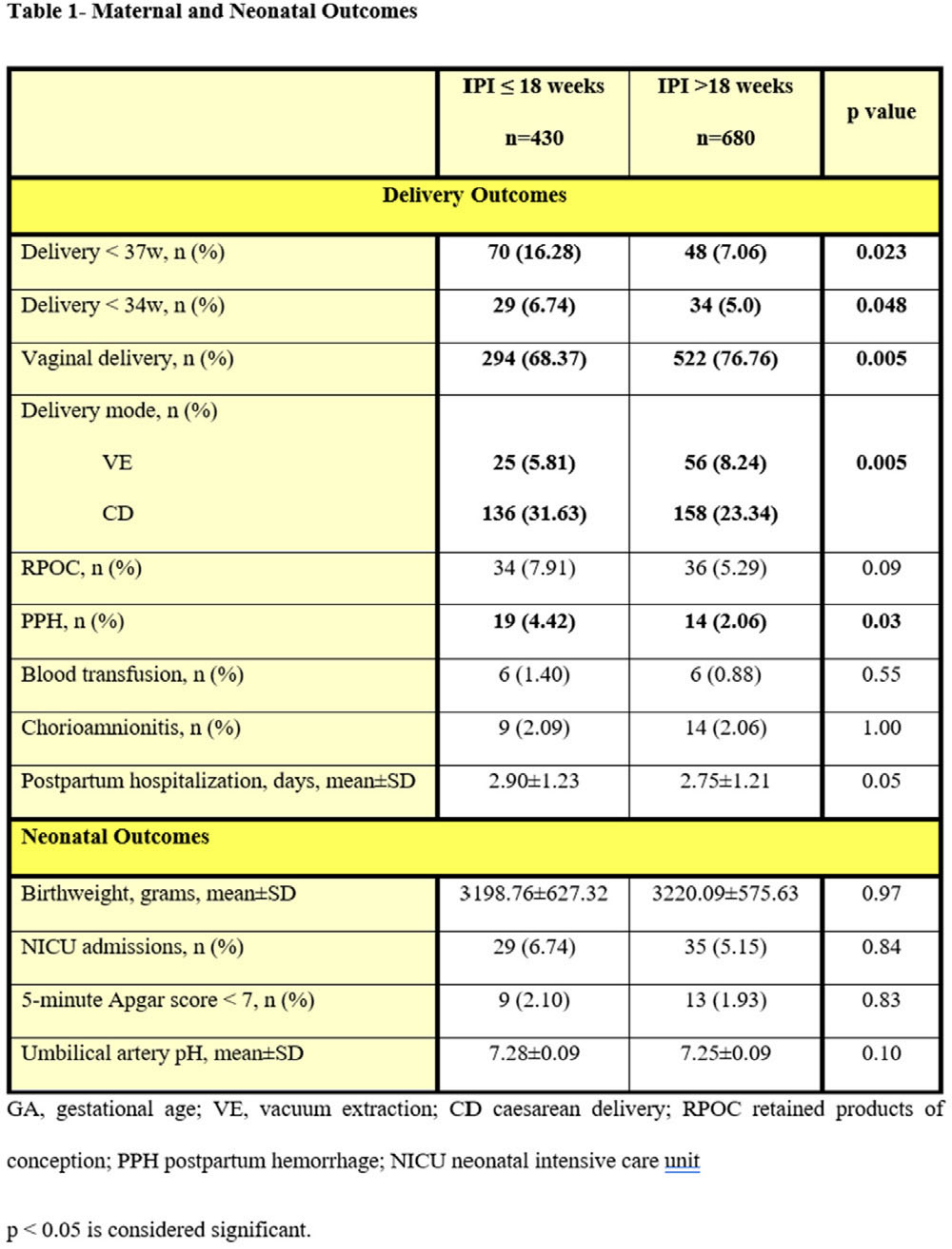

## 13 | Current screening guidelines and their effect on women with late‐onset gestational diabetes

Taylor A. DeAnnuntis^1^, Robert Ehsanipoor^2^, Adriane Burgess^2^

^1^WellSpan Health, York, Pennsylvania; ^2^WellSpan Health, WellSpan Health, Pennsylvania

10:15–10:30

**Objective**: To evaluate the prevalence of gestational diabetes (GDM) in women who have sonographic findings of EFW >90%, AC >95%, and polyhydramnios in the third trimester who were previously not diagnosed with GDM.

**Study Design**: A patient registry was created by the perinatal diabetes team between 2018‐2023 that included pregnant women who had an ultrasound with an MFM sonographer at >28 weeks gestation and were identified as having a fetus with either an abdominal circumference (AC) >95%, estimated fetal weight (EFW) >90% or polyhydramnios (AFI >25 cm) and no prior diagnosis of GDM. These patients were referred for a third‐trimester 3hr GTT or glucose paneling to evaluate for GDM. Primary outcome analyzed was frequency of newly diagnosed GDM. Other outcomes analyzed included mode of delivery, live birth, incidence of shoulder dystocia, respiratory distress syndrome, diagnosis of preeclampsia, neonatal hypoglycemia, and NICU admission.

**Results**: A total of 723 patients were included and 169 (23.4%) tested positive for GDM in the third trimester despite not having a prior diagnosis of GDM. Of those with an EFW >90%, AC >95%, or polyhydramnios, 25.4% (*n* = 123), 24.4% (*n* = 158), and 21.2% (*n* = 22) ruled in for GDM in the third trimester, respectively. Furthermore, 19.5% (*n* = 50) of those with one sonographic criteria, 25.5% (*n* = 104) with two sonographic criteria, and 26.9% (*n* = 14) of those with 3 sonographic criteria had a positive third trimester 3 hr GTT or paneling. There was a statistically significant association between those who ruled in for GDM in the third trimester and neonatal hypoglycemia (30.4%; *n* = 45; *p* < .001), respiratory distress syndrome (10.8%; *n* = 16; *p* = .037) and NICU admission (13.4%; *n* = 20; *p* = .036).

**Conclusion**: When findings of EFW >90%, AC >95%, and polyhydramnios are noted in the third trimester, up to ¼ of women will be found to have newly diagnosed GDM on subsequent evaluation. Increased neonatal morbidity was also noted in this group. This finding suggests that evaluation for GDM should be considered when these sonographic findings are present.

## 14 | Impact of metformin on the fetal lipidome in obese pregnancy

Grace J. Hattersley^1^, Antonia Hufnagel^2^, Denise Fernandez‐Twinn^2^, Samuel Furse^2^, Benjamin Jenkins^2^, Albert Koulman^2^, Rebecca M. Reynolds^3^, Susan E. Ozanne^2^, Catherine E. Aiken^4^, Catherine E. Aiken^4^

^1^School of Clinical Medicine, University of Cambridge, Cambridge, UK, Cambridge, England; ^2^Wellcome‐MRC Institute of Metabolic Science and Medical Research Council Metabolic Diseases Unit, University of Cambridge, Addenbrooke's Hospital, Cambridge, UK, Cambridge, England; ^3^Centre for Cardiovascular Science, Queen's Medical Research Institute, University of Edinburgh, Edinburgh, UK, Edinburgh, Scotland; ^4^Department of Obstetrics and Gynaecology, the Rosie Hospital and NIHR Cambridge Biomedical Research Centre, University of Cambridge, Cambridge, UK, Cambridge, England

10:30–10:45

**Objective**: Metformin is widely used therapeutically during pregnancy, including for treatment of gestational diabetes. It is known that metformin crosses the placenta and enters the fetal circulation, however the specific effects on fetal physiology and metabolism are unclear. Thus, we investigated whether there are changes to the fetal lipidome associated with metformin treatment during pregnancies complicated by maternal obesity.

**Study Design**: Fetal serum was obtained from 10 human pregnancies (mean maternal BMI 44.6kg/m^2^) where women were treated with metformin for gestational diabetes (500‐2000mg/day; average maternal serum concentration 2100 ± 600nM). A complimentary mouse (C57Bl6/J) model of diet‐induced (high sugar/high fat) maternal obesity and glucose intolerance treated with metformin (average maternal serum concentration 1669 ± 568nM) was developed and fetal cardiac tissue obtained at e18.

**Results**: There was no correlation in humans between fetal circulating metformin concentrations and circulating concentrations of free polyunsaturated fatty acids (Fig 1A). In our mouse model, the fetal heart lipidome was not altered with metformin treatment (Fig 1B). There was an increase in fetal cardiac diacylglyercol concentration with metformin treatment, but no differences in any other lipid class (Fig 1C). There was no significant correlation between concentrations of any specific diacylglycerol species in the fetal heart and metformin treatment, but enrichment in the metformin‐treated group was driven primarily by shorter‐chain saturated species (Fig 1D).

**Conclusion**: We show that human fetal exposure to metformin does not impact fetal circulating free polyunsaturated fatty acids. In murine fetal cardiac tissue, diacylglycerols were increased with metformin treatment but the other lipid classes were unaffected. Our results are reassuring with respect to the circulating fetal lipidomic impacts of metformin use in pregnancy, however there are multiple other possible mechanisms by which metformin could impact fetal physiology.

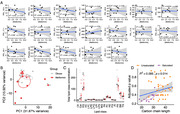

## 15 | Identifying sociodemographic barriers to continuous glucose monitor usage among pregnant patients with type 1 diabetes

Shannon McCloskey^1^, Kali Juracek^2^, John A. Morgan^2^, James D. Toppin^1^, Joseph R. Biggio, Jr^2^, Frank B. Williams^1^

^1^Ochsner Clinic Foundation, New Orleans, Louisiana; ^2^Ochsner Health, New Orleans, Louisiana

10:45–11:00

**Objective**: Continuous glucose monitor (CGM) use among pregnant patients with type 1 diabetes mellitus (T1DM) has been associated with improved glycemic control. Social determinants of health (SDH) are associated with adverse pregnancy outcomes and delayed uptake of CGM. We hypothesize that SDHs are associated with decreased CGM use in pregnancy among patients with T1DM.

**Study Design**: We conducted a cohort study of T1DM pregnancies receiving prenatal care and delivering in a single large healthcare system from 1/2016 to 1/2023. Those with missing delivery information, care presentation after 20 weeks, or unclear CGM status were excluded. Patients were evaluated by public versus private insurer status. Primary outcome was CGM use during pregnancy. Additional SDH characteristics evaluated included home address Area Deprivation Index (ADI) national percentile and rurality status per Rural‐Urban Commuting Area Codes. Regression analyses generating models predicting CGM use included maternal age, baseline body mass index (BMI), diabetes duration, and delivery year.

**Results**: Among 288 pregnancies with T1DM, 144 (50.0%) had public insurance. Public insurance was associated with younger age and shorter T1DM duration. Public payor patients were more likely to be nulliparous, Black, and to have chronic hypertension (Table). Private insurance patients had lower median baseline glycosylated hemoglobin and higher insulin pump use. Controlling for age, duration, and BMI, CGM usage was lower in the public payor group (38.9% vs 61.8%, aOR 0.39, 95% CI 0.23‐0.66). CGM use among publicly insured patients increased over time but lagged privately insured patient proportions by two years. In a model evaluating delivery year and SDH characteristics associated with CGM usage, public insurance (aOR 0.47, 95% CI 0.24‐0.91) and ADI (aOR 0.96, 95% CI 0.95‐0.98) were independently associated with CGM use; rurality was not.

**Conclusion**: Public insurance and neighborhood deprivation are risk factors for lower CGM uptake, potentially representing an indirect and targetable mechanism by which SDH impacts glycemic control for T1DM in pregnancy.

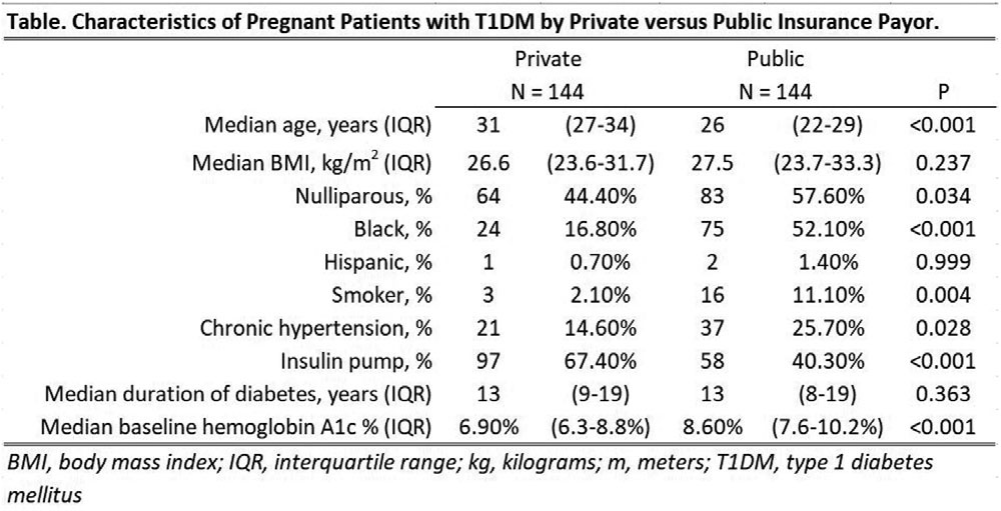

## 16 | Comparison of seatbelt counseling to other risk factor counseling for pregnant individuals

Mary K. Gallaher^1^, Ruyun Jin^2^, Corina Espelien^3^, Jason Forman^3^

^1^University of Virginia Center for Applied Biomechanics, Charlottesville, Virginia; ^2^Division of Biostatics, Department of Public Health Sciences, University of Virginia, University of Virginia, Virginia; ^3^Center for Applied Biomechanics, University of Virginia, Center for Applied Biomechanics, University of Virginia, Virginia

11:00–11:15

**Objective**: The American College of Obstetricians and Gynecologists recommends counseling for seatbelt and substance use during prenatal care, but it remains unclear if counseling is reaching the pregnant population consistently in prenatal visits. This study aims to assess the frequency of seatbelt counseling in the Pregnancy Risk Assessment Monitoring System (PRAMS) national survey and compare it to counseling frequencies for other risk factors during pregnancy.

**Study Design**: Data from the PRAMS dataset for 2009‐2015 was obtained from the Centers for Disease Control and Prevention (CDC). Data were included for states and years if less than 20% of the data were missing for each of the four survey questions of interest. The data was weighted based on survey‐provided weighting factors and percentages of individuals receiving counseling were calculated for each state‐year.

**Results**: The percentage of individuals receiving prenatal counseling about seatbelt use (52%) was lower than the percentages receiving counseling on alcohol (72%), smoking (71%), and illicit drug use (63%). Pairwise correlations show that alcohol use, smoking, and drug use counseling are highly related (correlation coefficient r≥0.92). Alcohol and smoking counseling had the highest correlation (r = 0.96) and smoking and seatbelt use counseling had the lowest correlation (r = 0.53).

**Conclusion**: Motor vehicle collisions (MVCs) are a leading cause of trauma‐related mortality for pregnant individuals in the United States. Seatbelts reduce the likelihood of adverse maternal and fetal outcomes in MVCs. It is important that pregnant individuals wear their seatbelts properly, yet this study shows seatbelt counseling is provided in only about half of prenatal visits, and less frequently than counseling on other risk factors. Given that 75% of pregnant individuals receive early and adequate prenatal care (CDC, 2022), clinicians’ efforts to disseminate seatbelt information, as well as counseling on other risk factors could effectively reach the majority of pregnant individuals during prenatal visits.

## ORAL CONCURRENT SESSION 4 ‐ INNOVATION AND PUBLIC HEALTH

Friday, 27 September 2024 09:00–11:15

## 18 | Cannabis use in the first trimester and congenital anomalies

Torri D. Metz^1^, Amanda A. Allshouse^1^, Gwendolyn A. McMillin^2^, Robert M. Silver^2^

^1^University of Utah, Salt Lake City, Utah; ^2^University of Utah Health, Salt Lake City, Utah

10:00–10:15

**Objective**: To evaluate whether first trimester exposure to cannabis was associated with congenital anomalies overall and by specific organ system.

**Study Design**: Secondary analysis of prospectively collected data from 8 U.S. centers participating in the nuMoM2b cohort. Cannabis exposure was determined by immunoassay for 11‐nor‐9‐carboxy‐delta‐9‐tetrahydrocannabinol (THC‐COOH) using frozen stored urine samples from a study visit between 6w0d and 13w6d; positive results were confirmed with liquid chromatography tandem mass spectrometry (LC‐MS/MS with cutoff 15 ng/mL). The primary outcome was any structural anomaly identified by antenatal ultrasound, or during neonatal hospitalization. Covariates considered for inclusion in modeling included folic acid use, maternal age, pre‐existing diabetes, and nicotine use. Nicotine exposure was determined by LC‐MS/MS for urine cotinine. Odds ratios for the primary outcome were reported from logistic regression modeling using backwards selection, the same model parameterization was used for individual organ systems.

**Results**: Overall 9233 participants were included, and 609 (6.6%) were exposed to cannabis in the first trimester. Maternal age, BMI, race and ethnicity, insurance type, and prevalence of chronic hypertension differed between exposed and unexposed groups, while pre‐gestational diabetes and folic acid use did not.

There was no difference in the incidence of any congenital anomaly between groups (8.9% exposed vs 8.4% unexposed, Table). In a multivariable logistic regression model, cannabis use was not associated with any structural anomaly (aOR 1.16, 0.86‐1.57). However, cannabis exposure was associated with cardiac anomalies [3.3% exposed vs 1.8% unexposed, aOR 2.09 (95% CI 1.28‐3.42)].

**Conclusion**: While cannabis use was not associated with most congenital anomalies, it was associated with cardiac anomalies. A prior birth defect registry study similarly demonstrated an association with ventricular septal defects. Given the high prevalence of cannabis use in the setting of expanding legalization, further work is needed to confirm these observations. 

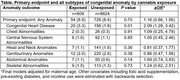

## 19 | Increased cardiovascular mortality in women after spontaneous preterm birth: A national linked cohort study

Sophie M. Welters^1^, Christianne J.M. de Groot^2^, Esmé, Kamphuis^3^, Ben W. Mol^4^, Anita C. Ravelli^2^, Marjon A. de Boer^2^

^1^Amsterdam UMC, Haarlem, Noord‐Holland; ^2^Amsterdam UMC, Amsterdam, Noord‐Holland; ^3^Amsterdam UMC, Amsterdam UMC, Amsterdam, Noord‐Holland; ^4^Monash University, Clayton, Victoria

10:15–10:30

**Objective**: Women with a history of induced preterm birth for preeclampsia and fetal growth restriction have an increased cardiovascular disease (CVD) risk later in life. Recent data also suggest a higher cardiovascular disease risk for women with a history of spontaneous preterm birth (PTB). We assessed whether women with a history of spontaneous PTB have an increased cardiovascular mortality risk.

**Study Design**: We used data‐linkage between the National hospital Birth Registry and the National Death Registry. Data from women with a first birth between 1995 ‐ 2015 were linked with the National Death Registry until 2017 and analyzed for cardiovascular mortality (CVM). First, all women with a PTB, both indicated and spontaneous, in their first singleton pregnancy were analyzed for CVM. We then restricted our analysis to women with a spontaneous PTB  by excluding women with hypertensive pregnancy disorders and/or fetal growth restriction. CVM risk was analyzed in different gestational age groups.

**Results**: The cohort consisted of 1,166,476 women of whom 9.4% had a PTB (*n* = 109,935) and 6.4% (*n* = 74,722) had a spontaneous PTB. Median follow‐up time was 10.1 years. Women with a PTB had a higher CVM risk than women with a term birth in their first pregnancy (HR 1.90; 1.59–2.30). A history of spontaneous PTB in the first pregnancy was associated with an increased CVM risk (aHR 1.38, 95% CI 1.02 to 1.87) compared to women who had a term birth.  In the subgroup analysis, women with a spontaneous PTB 22‐ 32 weeks gestational age (GA) had a higher CVM risk (adjusted hazard ratio (aHR) 3.19; 1.84 to 5.56) compared to women with a first term birth.

**Conclusion**: Women with a history of PTB are at elevated risk for CVM. Also, women with a spontaneous PTB, after excluding women with hypertensive disorders of pregnancy and fetal growth restriction, are at elevated risk for CVM later in life, specifically women with a spontaneous PTB < 32 weeks GA.

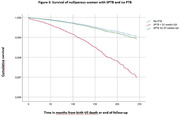

## 22 | Correlates with perceived racial discrimination in healthcare among postpartum individuals

Jonas J. Swartz^1^, Catherine Gervais^2^, Tracy Truong^3^, Evan Myers^2^, Norma Davis^2^, Maria I. Rodriguez^4^, Geeta K. Swamy^2^, Keisha Bentley‐Edwards^2^

^1^Duke University Medical Center, Durham, North Carolina; ^2^Duke University, Durham, North Carolina; ^3^Duke University School of Medicine, Durham, North Carolina; ^4^Oregon Health & Science Unviersity, Portland, Oregon

11:00–11:15

**Objective**: Structural, interpersonal and internalized racism are associated with various adverse maternal and infant outcomes. We sought to assess perceived discrimination in healthcare among individuals hospitalized for obstetric delivery.

**Study Design**: We conducted a prospective, exploratory cross‐sectional survey among postpartum individuals during their delivery hospitalization. In addition to questions about sociodemographics, and social determinants of health, the survey included a validated measure of perceived discrimination in healthcare. Obstetric data was abstracted from the electronic health record.  Perceived discrimination was quantified using the sum of scores across eight domains, the further categorized as reporting (a) No or Few Discrimination Experiences, (b) Several Discrimination Experiences, and (c)  Frequent Discrimination Experiences.

**Results**: We recruited 491 individuals who completed the survey. Racial diversity was reflective of state and health system demographics: most patients identified as White (43.2%) Black (36.5%), or Asian (7.6%), with 10.2% of patients reporting Hispanic ethnicity. Private insurance (59.1%) was the largest payor, followed by Medicaid (33.9%). 66.3% of patients had BMI >30. Overall, 22.3% of patients reported discrimination at least once in at least one domain. Those reporting several or frequent discrimination experiences were disproportionately Black (52.9% several, 55.0% frequent), and we observed a trend toward increasing prevalence of BMI >30 with higher frequency of discrimination (65.0% no or few, 69.0% several, 80.0% frequent). The most frequently reported concerns were that doctors or nurses did not listen to the patient (18.0%) or acted as though the patient was not smart (12.3%).

**Conclusion**: Among a diverse cohort of immediately postpartum individuals, over one in five reported multiple episodes of perceived discrimination in healthcare. The most common perceived racial discrimination occurred during communication between patients and providers. This targeted opportunity for intervention offers a promising avenue to decrease discriminatory behavior.

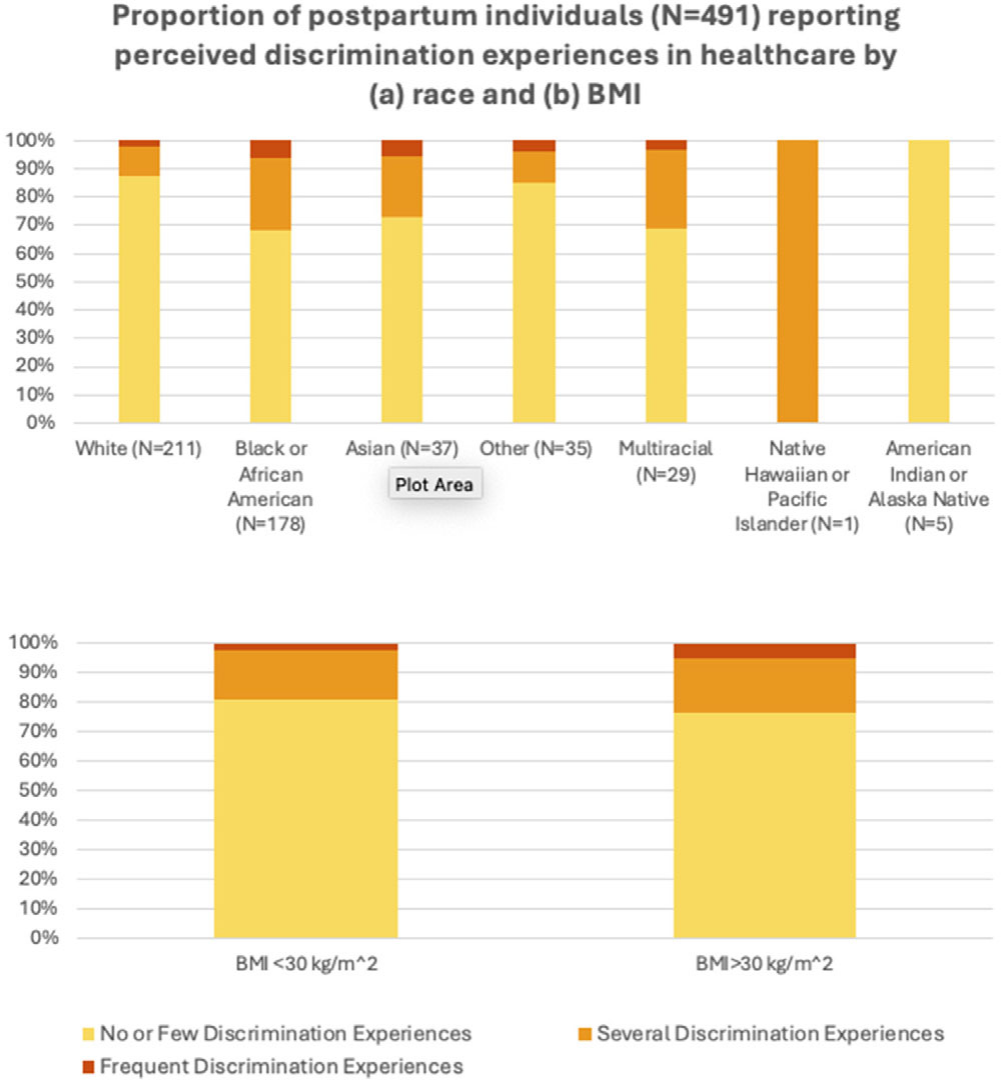

## ORAL CONCURRENT SESSION 5 ‐ LABOR

Friday, 27 September 2024 14:30–16:45

## 23 | External cephalic version in non‐vertex presenting twin – A prospective interventional comparative study

Zvi Ehrlich^1^, Shirley Shapira^2^, Ari Weiss^3^, Alexander Ioscovich^2^, Sorina Grisaru Granovsky^4^, Vladimir Plotkin^2^, Misgav Rottenstreich^5^, Hen Y. Y. Sela^6^

^1^Shaare Zedek Medical Center, Jerusalem, Yerushalayim; ^2^SZMC, Shaare Zedek Medical Center, Yerushalayim; ^3^Shaare Zedek Medical Center, Shaare Zedek Medical Center, Yerushalayim; ^4^Hebrew University, Jerusalem, Yerushalayim; ^5^McMaster University, Hamilton, Ontario; ^6^Shaare Zedek, Jerusalem, Yerushalayim

15:30–15:45

**Objective**: Twin pregnancies with the presenting fetus in a non‐vertex presentation typically result in cesarean delivery (CD). Performing an external cephalic version (ECV) for the presenting twin is rarely reported. This study aimed to assess the feasibility and safety of ECV in twins with a non‐vertex presenting fetus.

**Study Design**: This prospective interventional study included term, multiparous women with no prior CD, having a Dichorionic Diamniotic (DCDA) twin pregnancy with a non‐vertex presenting twin. Between 2020 and 2024, these women were offered ECV during pre‐operative assessment preceding a planned CD. Exclusions encompassed cases with known fetal anomalies, small for gestational age fetuses, and intra‐uterine fetal death of one or both twins. ECV was performed under combined spinal‐epidural anesthesia. Successful ECV led to induction of labor (IOL), while unsuccessful attempts resulted in immediate CD. Maternal and neonatal outcomes of study participants opting for ECV were compared with those choosing a planned CD. The primary outcome was a composite adverse maternal and neonatal outcome. An intention to treat analysis was done. Univariate analysis was followed multivariate logistic regression, adjusting for relevant covariates.

**Results**: Out of 107 included women, 55 opted for ECV prior to CD, while 52 preferred a planned CD without ECV. Labor onset before the planned procedure necessitated unplanned CD in 7.3% of the ECV group and 13.5% of the planned CD group. Overall, 56.9% of the ECV attempts were successful, with 89.7% resulting in vaginal delivery. The incidence of composite adverse maternal and neonatal outcomes did not significantly differ between the groups (18.2% vs. 23.1%, *p* = 0.54, adjusted Odds ratio 0.69; 95% confidence interval 0.26‐1.85), although those undergoing ECV had a shorter hospital stay (3.5 ± 1.9 vs. 4.3 ± 1.7 days, *p* = 0.02).

**Conclusion**: Performing ECV for non‐vertex presenting twins appears feasible and likely safe, with a high subsequent vaginal delivery rate, thus reducing CD rates in this cohort. Larger studies are warranted to assess rare outcomes.

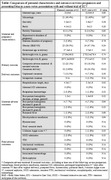

## 24 | Tafoxiparin a new drug candidate for labor priming in women with unripe cervix

Gunvor Ekman Ordeberg^1^

^1^Karolinska Institutet, Danderyd, Stockholms Lan

15:45 ‐ 16:00

**Objective**: Labor induction is increasing to avoid fetal and maternal problems. Tafoxiparin is a new drug candidate with a new mechanism of action inducing both cytokines in human cervical cells and increase Ca‐influx in human myometrial cells. Thus, a positive effect on both the Extra Cellular Matrix (ECM) with cervical ripening and facilitation of labor. Tafoxiparin does not pass placenta. An explorative study showed both positive effect on cervical ripening and a shorter labor time. Successful results from a Phase IIb study will be presented.

**Study Design**: A Randomized, Double‐Blind, Placebo‐Controlled, Parallel‐Group Dose Finding Proof of Concept Study.Nulliparous women (347) with unripe cervix (Bishop score < = 4p) were enrolled. Tafoxiparin/Placebo was daily given subcutaneously up to 7 injections and the cervical state and CTG were checked. In women not starting spontaneously amniotomy and oxytocin were used when a ripe cervix was registered and if an unripe cervix was noted a balloon was applied. Endpoints were, Efficacy of tafoxiparin on cervical ripening, maternal and neonatal safety.

**Results**: The most effective dose was identified associated with a significant cervical ripening rate *p* = 0.006. Moreover 59% had a spontaneous onset/ripe cervix after only tafoxiparin compared to 39% after placebo.  Time from start of treatment until ripe cervix /spontaneous onset of labor was significantly better after tafoxiparin *p* = 0.009. Significant fewer instrumental deliveries were reported after tafoxiparin *p* = 0.025. Operation delivery for fetal distress was 12% with tafoxiparin compared with placebo 23% *p* = 0.07 a reduction close to 50%. No problem with hyperstimulation was reported.

**Conclusion**: Tafoxiparin improves the maternal and fetal outcomes by priming of labor mimicking the natural process, resulting in only 41% of women need further cervical ripening treatment.The safety profile including lack of hyperstimulation supports tafoxiparin for an outpatient setting.

## 25 | The impact of body mass index on misoprostol dosing for labor induction

Taylor M. Bynarowicz^1^, Surya Sruthi Bhamidipalli^1^, Kathleen Flannery^1^, Joanne Daggy^2^, Sara Kay K. Quinney^1^, David M. Haas^1^

^1^Indiana University School of Medicine, Indianapolis, Indiana; ^2^Department of Biostatistics and Health Data Science, Indiana University School of Medicine, Department of Biostatistics and Health Data Science, Indiana University School of Medicine, Indiana

16:00–16:15

**Objective**: To determine if the number of doses to achieve active labor differed across body mass index (BMI) categories for buccal versus vaginal misoprostol during labor induction.

**Study Design**: This is a secondary analysis of the IMPROVE study (NCT 02408315), a triple‐blinded, placebo‐controlled randomized controlled trial of vaginal versus buccal misoprostol in term patients undergoing induction. The primary outcome for this analysis was the number of doses of misoprostol needed for active labor. Participants were grouped into 3 BMI categories (≤ 29/non‐obese, 30‐40/obese, and ≥ 41/morbidly obese). Groups were compared with Wilcoxon and Chi‐square or Fisher's exact test. Generalized linear models were used to adjust for ethnicity, race, dosing route and induction indication.

**Results**: Of 299 participants with complete data, 63 had a BMI ≤ 29 (21%), 165 had a BMI between 30 and 40 (55%), and 71 had a BMI ≥ 41 (23%). The groups did not differ in age, race, gestational age, nulliparity, initial Bishop score or route of dosing. There were significantly more non‐Hispanic participants in the obese categories (*p* = 0.02). A higher proportion of obese or morbidly obese participants had diabetes or a hypertensive disorder as the primary indication for induction (*p* = 0.03). In the univariate analysis, more doses of misoprostol were needed to achieve active labor in the obese and morbidly obese groups when dosed buccally (*p* = 0.01) but not when dosed vaginally (*p* = 0.21).  Rates of vaginal delivery in < 24 hours, cesarean delivery or cesarean for fetal distress did not significantly differ across BMI category or dosing route. Adjusting for induction indication and self‐reported race/ethnicity, obese participants required more doses of misoprostol regardless of dosing route (*p* < 0.01).

**Conclusion**: Obesity (BMI ≥ 30) was associated with more misoprostol doses to achieve active labor. Additionally, buccal dosing was independently associated with a higher number of doses compared to vaginal.

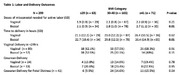

## 26 | Personalized machine learning model for predicting successful vaginal delivery during the active phase of labor

Lee Reicher^1^, Emmanuel Attali^2^, Sharon Napadenski^3^, Nadav Rappoport^3^, Yariv Yogev^4^

^1^Weizmann Instutute Of Science, Weizmann Institute of Science, HaMerkaz; ^2^Lis Hospital for Women's Health, Tel Aviv Sourasky Medical Center, Tel‐Aviv, Tel Aviv; ^3^Ben‐Gurion University of the Negev, beer sheva, HaDarom; ^4^Lis Maternity Hospital, Sourasky Medical Center, Tel Aviv University, Tel Aviv Sourasky Medical Center, Tel Aviv

16:15–16:30

**Objective**: Accurate personalized prediction of delivery mode during the active stage improves resource allocation, reduces unnecessary interventions, and enhances patient‐centered care. However, current machine learning models lack focus on active labor and effective use of labor‐related data. The “black box” problem further impedes understanding, accuracy, and patient safety. Our aim was to develop a personalized model using real‐time data to predict successful vaginal deliveries in the active phase and provide an explanation of the model's decision‐making process.

**Study Design**: Labor records from a tertiary hospital were searched and labeled over a 10‐year period. Three models were trained using both “static features” (e.g demographics) and “dynamic features” (e.g cervical dilatation, efficacy, and newly derived features calculated as ratios between adjusted dynamic measures and time differences). To assess the explainability of the best‐performing model, we employed SHAP (SHapley Additive exPlanations) analysis.

**Results**:

1. A total of 94,386 cases in which a trial of labor was attempted were identified.Of them ∼70,000 admitted to the active phase of labor (6 cm dilatation).
2. We trained three types of classifier models predicting successful vaginal delivery: Random Forest classifier, Histogram‐Based Gradient Boosting classifier, and neural network model **(Figure 1)**. The data split for train∖test was in the ratio of 80:20, respectively.
3. Histogram‐Based Gradient Boosting model achieved the highest scores: ROC AUC score and Precision recall AUC score was 0.87 (95% confidence interval, 0.858‐0.892) and 0.52 (95% confidence interval, 0.511‐0.542) respectively
4. Explainability of the best model was analyzed using SHAP. The features that contributed the most for the model prediction were: rate of fetal descent, cervical opening rate, maternal age and clinical fetal weight estimation.

**Conclusion**: Our model's explainability enhances decision‐making during active labor by providing insights into prediction factors, improving transparency and validation.

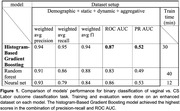

## ORAL CONCURRENT SESSION 6 ‐ STILLBIRTH AND MATERNAL MENTAL HEALTH

Friday, 27 September 2024 14:30–16:45

## 28 | Patterns of placental injury and clinicopathological phenotypes of stillbirth

Ragnheidur I. Bjarnadottir^1^, Thora Steffensen^2^, Alexander K. Smarason^3^, Nikos Papadogiannakis^4^, Karin Pettersson^5^, Johanna Gunnarsdottir^6^

^1^Landspitali University Hospital, Reykjavik, Hofuoborgarsvaoio; ^2^Department of Pathology, Tampa General Hospital, Department of Pathology, Tampa General Hospital, Tampa, Florida, USA, Florida; ^3^University of Akureyri, Sjukrahusið a Akureyri, Norourland Eystra; ^4^Karolinska Institutet, Karolinska Institutet, Stockholms Lan; ^5^Karolinska Institute, Stockholm, Stockholms Lan; ^6^University of Iceland, University of Iceland, Hofuoborgarsvaoio

15:45–16:00

**Objective**: To describe the major patterns of placental injury and clinicopathological phenotypes of singleton stillbirth (SB) in Iceland over quarter of a century.

**Study Design**: Data was collected from medical records of mothers who delivered stillborn singletons from 22+0 weeks of gestation in Iceland 1996‐2021. Placental pathology was reviewed and classified according to the Amsterdam consensus into: maternal vascular perfusion (MVM), chronic villitis of unknown etiology (VUE), and fetal vacular malperfusion (FVM). Association between each pattern of placental injury and the following clinical factors was examined: Gestational age (GA) at diagnosis of stillbirth, hypertensive disorders in pregnancy (HDP), birthweight or placental weight under 10th percentile for gestational age (SGA and small placenta respectively), elevated feto‐placental ratio (FPR) and umbilical cord at risk (see definition below Table 1). Chi‐square tests were applied and *p*‐level of 0.05 considered statistically significant.

**Results**: During the study period, 338 singletons were stillborn and over 96% of the placentas examined (*n* = 326). MVM was found in 57 placentas (16.9%), VUE in 46 (13.6%) and FVM in 70 (20.7%). More than one pattern of placental injury could be found in the same placenta (Table 1). MVM was significantly associated with HDP and SGA. Small placentae and elevated FPR were significantly associated with both MVM and VUE, but the latter was not associated with SGA. However, VUE became significantly more common with higher GA. FVM was associated with umbilical cord at risk and also SGA, which was unexpected.

**Conclusion**: The results support known association between MVM and HDP as well as SGA. VUE, which is neither associated with HDP nor SGA, has a significant association with small placentas and high FPR. Importantly, VUE is most common in stillbirth at term. In view of lack of clinical signs that can be detected before birth, biomarkers for VUE might have a role in reducing term stillbirth.

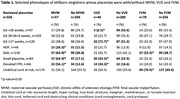

## 30 | The association of war with the risk for postpartum depression, anxiety and impaired maternal‐infant bonding

Hadar klapper‐goldstein^1^, Gali Pariente^2^, Tamar Wainstock^3^, Sharon Dekel^4^, Yair Binyamin^5^, Talya Lanxner Battat^5^, Orit Wissotzky Broder^6^, Tamar Kosef^5^, Eyal Sheiner^3^

^1^soroka hospital, beer sheva, HaDarom; ^2^Soroka University Medical Center, Soroka University Medical Center, HaDarom; ^3^Department of Obstetrics and Gynecology, Soroka University Medical Center, Ben‐Gurion University of the Negev, Beer‐Sheva, Israel., Beer Sheva, HaDarom; ^4^Massachusetts General Hospital, Massachusetts, Massachusetts; ^5^soroka, beer sheva, HaDarom; ^6^Soroka Medical Center, beer sheva, HaDarom

16:15–16:30

**Objective**: War and terrorism can critically undermine the individual mental states. Perinatal populations have been identified as a vulnerable group. Therefore, the aim of this study was to examine the impact of war conditions on maternal mental health outcomes pertaining to depression and anxiety and in adiition on maternal‐infant bonding (MIB).

**Study Design**: A prospective cohort study was performed in women who gave birth in in a tertiary medical center, during (October‐November 2023) and before (March‐May 2020) the Israel‐Hamas War. All participants completed validated self‐reported questionnaires at 1‐3 days postpartum. Mental health was measured using theEdinburgh Postnatal Depression Scale (EPDS) and the State‐Trait Anxiety Inventory (STAI) while maternal‐infant bonding was evaluated using the Postpartum Bonding Questionnaire (PBQ).

**Results**: A total of 502 women were included in the analysis, 230 delivered during the War and 272 delivered before. The rate of postpartum depression risk (EPDS > = 10) was higher in women delivering during the War (26.6% vs. 12.4%, *p* < 0.001, Table). Multivariable regression, accounting for ethnicity, history of perinatal mortality and cesarean delivery, revealed that the risk was two times higher among women delivering during the War (adjusted OR 2.35, 95% CI 1.16‐4.74, *p* = 0.017). Rate of postpartum anxiety risk (STAI >39) was higher in women delivering during the War (34.3% vs 17.0%, *p* < 0.001), and reached a trend towards significance accounting for other risk factors (adjusted OR 2.06, 95% CI 0.97‐4.36, *p* = 0.058). No significant difference in MIB was demonstrated between the groups (Total PBQ, 10.2 ± 14.1 vs. 8.3 ± 6.9, *p* = 0.075. Impaired bonding sub scale, 7.8% vs 5.5%, *p* = 0.363).

**Conclusion**: The study revealed increased risk of postpartum depression and tendency to postpartum anxiety in women who deliver during the War. Postpartum depression and anxiety can progress to permanent mental illness and effect the mother and infant. These should be taken to consideration when developing a supporting system in countries who face War state.

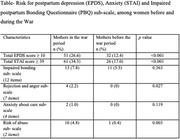

## 31 | Addressing perinatal mood disorders: Scaling up maternal mental healthcare by increasing access to treatment (SUMMIT)

Richard Silver^1^, Samantha Meltzer‐Brody^2^, Laura La Porte^3^, Nour Schoueri‐Mychasiw^4^, Jo Kim^5^, Daisy Singla^6^

^1^Endeavor Health (formerly NorthShore University HealthSystem), Endeavor Health (formerly NorthShore University HealthSystem)/Evanston, Illinois; ^2^UNC School of Medicine, Chapel Hill, North Carolina; ^3^Perinatal Depression Program/Endeavor Health (formerly NorthShore University HealthSystem), Endeavor Health (formerly NorthShore University HealthSystem)/Evanston, Illinois; ^4^Sinai Health/Toronto, Sinai Health/Toronto, Ontario; ^5^Colorado Center for Clinical Excellence/Denver, Colorado Center for Clinical Excellence/Denver, Colorado; ^6^Institute for Mental Health Policy Research/Centre for Addiction and Mental Health (CAMH), Centre for Addiction and Mental Health (CAMH)/Toronto, Ontario

16:30–16:45

**Objective**: Perinatal mood disorders affect 1 in 5 pregnant or postpartum persons, significantly increase the risk of adverse pregnancy outcomes and can be successfully treated with psychotherapy. Unfortunately, only 10% of patients have access to this intervention. Task‐sharing—or training non‐specialists (NSPs, individuals without formal mental health background) to deliver psychotherapy, and telemedicine can further reduce the barriers to care. We report a multi‐site non‐inferiority RCT whose primary aims were to determine whether behavioral activation delivered by NSPs (nurses, midwives and doulas) is non‐inferior to specialists (psychiatrists, psychologists and social workers) and whether telemedicine (TM) is non‐inferior to in‐person (IP) treatment.

**Study Design**: Adult pregnant, and postpartum patients with depressive symptoms (Edinburgh Postnatal Depression Scale, EPDS≥10), recruited in Toronto, Chicago, and Chapel Hill, were randomized to one of four conditions to receive brief behavioral activation from (1) trained NSP or (2) specialist; via either (3) telemedicine or (4) in person. The primary and secondary outcomes were non‐inferiority assessments of depressive and anxiety symptoms (EPDS and Generalized Anxiety Disorder; GAD‐7), respectively, 3‐months post‐randomization.

**Results**: Baseline characteristics for the 4 groups were similar (Table). At randomization, participants reported moderate depressive (EPDS = 15.73, SD = 3.91) and anxiety symptoms (GAD = 11.78, SD = 4.91). Non‐inferiority was met for reduced depression and anxiety when comparing either provider (NSP vs. SP) or modality (TM vs. IP) by intention to treat. There were no significant differences in analyses when accounting for baseline clinical severity, nor between recruitment phases relative to the COVID pandemic.

**Conclusion**: These findings support a global mental health approach of task sharing and telemedicine to increase access to brief, effective psychotherapy to address the significant treatment gap in perinatal depressive and anxiety symptom treatment.

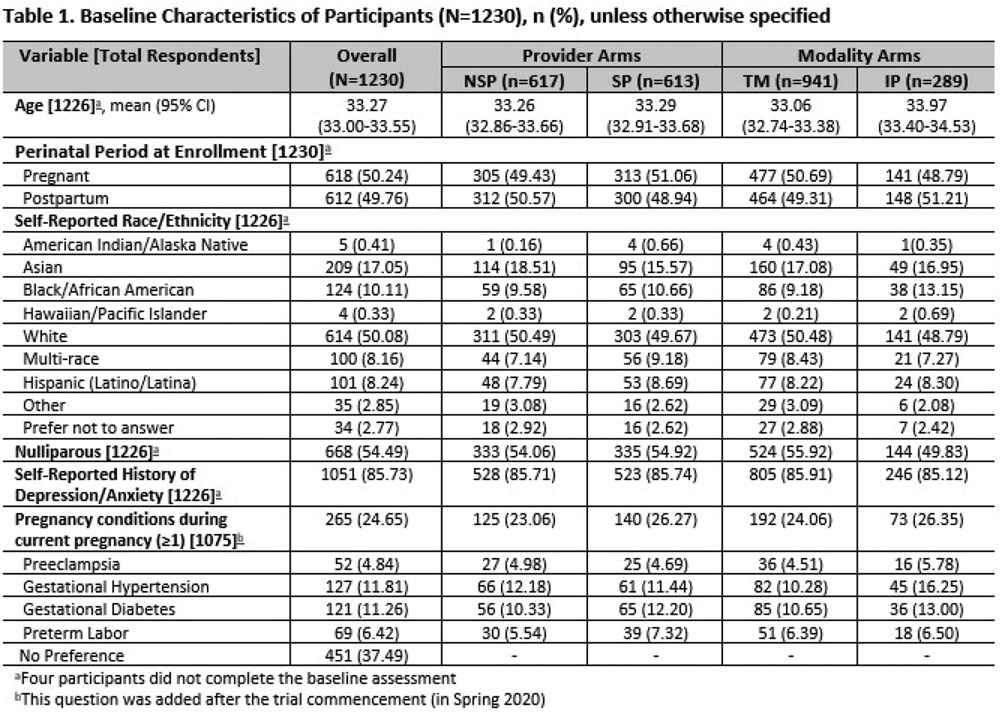

## ORAL CONCURRENT SESSION 7 ‐ PRETERM BIRTH

Saturday, 28 September 2024 09:00–11:15

## 32 | Prospective individual participant data meta‐analysis of pessary trials for preterm birth prevention (PROMPT) in singletons

Ben W. Mol^1^

^1^Monash University, Clayton, Victoria

09:45–10:00

**Objective**: To determine if cervical pessary use in the second trimester compared with no pessary in singleton pregnancies with a short cervix reduces the risk of fetal death or preterm delivery using a prospectively planned Individual Participant Data Meta‐Analysis (IPDMA).

**Study Design**: We performed a planned prospective Individual Participant Data Meta‐Analysis (IPDMA) (PROSPERO ID CRD42018067740) of four randomized trials. This analysis included individuals with a singleton pregnancy and a transvaginal ultrasound cervical length (CL) less than 25 mm at a gestational age (GA) before 24 weeks. Individuals with a previous preterm delivery were excluded. Participants were randomized to cervical pessary or no pessary. The primary outcome was a composite of fetal death or preterm delivery less than 32 weeks gestation (secondary neonatal and maternal outcomes listed in Table 1). Prespecified subgroup analyses were performed for type of pessary, CL, obstetrical history, gestational age at randomization, and randomization period. Log‐binomial or linear regression models were used to estimate relative risks or mean differences and their 95% confidence intervals (CI), as appropriate. Treatment by trial interactions were evaluated and random effects for trial were considered.

**Results**: 1,279 participants were included from the four trials, of which 655 (51%) were randomized to the pessary group and 624 (49%) to the no pessary group. There was no difference in the primary outcome of fetal death or preterm delivery before 32 weeks gestation (aRR, 95% CI: 0.82, 0.37‐1.83). There was a greater risk of genital or urinary tract infection in the pessary group compared with the no pessary group (aRR, 95% CI: 1.49, 1.08‐2.05). Among trials using non‐Arabin pessary, there was a greater risk of cesarean in the pessary group compared with no pessary group (77/226, 34% vs. 53/212, 25%; aRR, 95% CI 1.38, 1.03‐1.84).

**Conclusion**: In this prospectively planned IPDMA in singleton pregnancies with a short cervix, cervical pessary use did not prevent preterm birth or perinatal death.

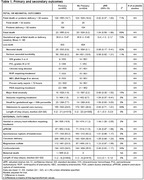

## 33 | Cervical pessary versus vaginal progesterone in multiples with a short cervix: A randomized controlled trial

Charlotte E. van Dijk^1^, Annabelle L. van Gils^1^, Maud D. van Zijl^1^, Bouchra Koullali^1^, Marijke c van der Weide^2^, Eline S.A. S. van den Akker^3^, Brenda J. Hermsen^3^, Henricus Visser^4^, Wilhelmina M van Baal^5^, Joris van Drongelen^6^, Karlijn C C. Vollebregt^7^, Flip W. W. van der made^8^, Sanne J. Gordijn^9^, Yolanda M. M. de Mooij^10^, Martijn A. Oudijk^11^, Marjon A. De Boer^2^, Ben W. Mol^12^, Brenda M. M. Kazemier^1^, Eva Pajkrt^1^

^1^Amsterdam UMC, Amsterdam, Noord‐Holland; ^2^Amsterdam University Medical Center, Amsterdam, Noord‐Holland; ^3^Onze Lieve Vrouwe Gasthuis, Amsterdam, Noord‐Holland; ^4^TerGooi Medisch Centrum, Hilversum, Noord‐Holland; ^5^Flevoziekenhuis, Almere, Flevoland; ^6^Radboud Universitair Medisch Centrum, Nijmegen, Gelderland; ^7^Spaarne Gasthuis, Haarlem, Noord‐Holland; ^8^St. Franciscus Hospital, Rotterdam, Zuid‐Holland; ^9^University Medical Center Groningen, Groningen, Groningen; ^10^Zaans Medisch Centrum, Zaandam, Noord‐Holland; ^11^Amsterdam UMC, location University of Amsterdam, Amsterdam, Noord‐Holland; ^12^Monash University, Clayton, Victoria

10:00–10:15

**Objective**: Multiple pregnancy and a mid‐gestation short cervix are known risk factors for spontaneous preterm birth (sPTB).  We aimed to compare the effectiveness of cervical pessary and vaginal progesterone in multiples without a previous sPTB < 34 weeks of gestation (GA) and with a short cervix for the improvement of perinatal outcome through prevention of PTB.

**Study Design**: We performed an open‐label multi‐centre randomized clinical trial in 20 hospitals in the Netherlands (EUCTR2013‐002884‐24‐NL). Asymptomatic individuals with a multiple (twin or triplet) pregnancy without previous sPTB < 34 weeks of GA and with a mid‐gestation cervical length < 38mm were randomized in a 1:1 ratio to receive either an Arabin® cervical pessary or vaginal progesterone 200mg daily. Primary outcome was a composite of adverse perinatal outcomes. Secondary outcomes were rates of (s)PTB < 24, 28, 32, 34 and 37 weeks. Treatment effect was expressed as relative risk (RR) and 95% confidence intervals (CI). The sample size was set at 332 women and analysis was by intention‐to‐treat. The study was halted for futility based on the results of 237 participants at the 4^th^ interim analyses.

**Results**: Between October 2014 and August 2023, we randomized 277 multiples, including 7 triplets,  (pessary *N* = 138, progesterone *N* = 139) with 531 neonates (pessary *N* = 267, progesterone *N* = 264). The composite adverse perinatal outcome occurred in 19.3% of neonates in the pessary group versus 13.4% of neonates in the progesterone group (crude RR 1.6; 95% CI 0.97–2.5). The rates of (s)PTB were not significantly different between groups. In the predefined subgroup of cervical length ≤ 25mm, the composite perinatal outcome was equally frequent in the pessary and progesterone group (42.1% vs 34.0%, RR 1.4; 95% CI 0.64–3.1).

**Conclusion**: In multiples without a history of previous sPTB < 34 weeks and with a mid‐gestation cervical length < 38 mm or ≤ 25 mm, treatment with a pessary or progesterone did not result in statistically significant differences in the prevention of a composite adverse perinatal outcome.

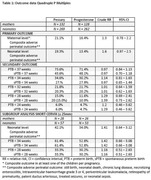

## 34 | Efficacy of aspirin in the prevention of recurrent spontaneous preterm birth based on placental histology

Anna JEMC. Landman^1^, Adelia Gieskes^2^, Laura visser^1^, Patrick van der Voorn^1^, Marieke Sueters^3^, Judith O.E.H. van Laar^4^, Mireille N. Bekker^5^, Christianne J.M. de Groot^2^, Martijn A. Oudijk^6^, Marjon A. de Boer^2^

^1^Amsterdam UMC, Amsterdam UMC, Noord‐Holland; ^2^Amsterdam UMC, Amsterdam, Noord‐Holland; ^3^LUMC, LUMC, Zuid‐Holland; ^4^Máxima Medical Centre, Veldhoven, The Netherlands, Veldhoven, Noord‐Brabant; ^5^University Medical Center Utrecht, Utrecht; ^6^Amsterdam UMC, location University of Amsterdam, Amsterdam, Noord‐Holland

10:15–10:30

**Objective**: Low‐dose aspirin is effective in the prevention of preterm preeclampsia. Recent evidence suggests that aspirin might also be beneficial for the prevention of spontaneous preterm birth (PTB). Important overlap between preeclampsia and spontaneous PTB is seen in placental histology, since signs of maternal vascular malperfusion (MVM) are also found in a large proportion of women with a spontaneous PTB. The APRIL trial showed that low‐dose aspirin did not significantly reduce PTB in women with a previous spontaneous PTB. Here we evaluate the effectiveness of low‐dose aspirin in the prevention of PTB in subgroups based on placental histology.

**Study Design**: This is a secondary analysis of the APRIL study, a multicenter double‐blinded trial in which women with a previous spontaneous PTB were randomized between low‐dose aspirin (80 mg) and placebo. Placental histology reports from the index spontaneous PTB were retrieved from participating centers and histology reports were classified by a pathologist who was blinded for treatment allocation and pregnancy outcome. Participants were grouped based on the presence of MVM.

**Results**: In total 141 (43.1%) placental histology reports of the included participants were available for  analysis. MVM was seen in 35 placentas of the index pregnancies (24.8%). In women with MVM the outcome PTB occurred in 10.5% in the aspirin group compared to 43.8% in the placebo group (OR 0.15; 95% CI 0.03‐0.89, *p* = 0.0036). In women without MVM PTB was reported in 28.3% in the aspirin versus 20.8% in the placebo group (OR 1.51 (0.62—3.68) *p* = 0.061 respectively).There was a significant interaction between allocated treatment and the classification of placental histology (*p* = 0.023).

**Conclusion**: The effect of low‐dose aspirin in the prevention of recurrent preterm birth is mediated by the presence of MVM in the placenta of the index spontaneous PTB. This supports and clarifies the potential role of aspirin in the prevention of spontaneous PTB.

## 35 | External validation of the QUiPP‐App: Study across two independent European cohorts of symptomatic women

Anne Fischer^1^, Kisten Bos^1^, Petra Bakker^1^, Anna Rietveld^1^, Mark Hoogendoorn^2^, Pim Teunissen^3^, Frederik J.R. Hermans^1^

^1^Amsterdam UMC, Amsterdam UMC, Noord‐Holland; ^2^VU University, VU University, Noord‐Holland; ^3^Maastricht University, Maastricht University, Limburg

10:30–10:45

**Objective**: External validation of the QUiPP App v.2 for the prediction of spontaneous preterm birth (sPTB) in two independent cohorts of symptomatic women attending a European tertiary care center. The objective is to assess the applicability of the QUiPP App outside a UK population.

**Study Design**: A prospective European multicenter cohort (*n* = 452) and retrospective single center cohort in the Netherlands (*n* = 572) were used. The cohorts consisted of pregnant women between 23‐34 weeks with symptoms of threatened preterm labor attending a tertiary care hospital between 2013 and 2023. We calculated QUiPP's prognostics using quantitative fetal fibronectin (qfFN) and/or cervical length (CL), and the presence of other risk factors. Discrimination was analyzed by calculating the AUC curve of three QUiPP models (qfFN alone, CL alone, CL+ qfFN) for the risk of sPTB at six time points: within one, two and four weeks after testing and birth before < 30, < 34 and < 37 weeks. Sensitivity and specificity were calculated using a threshold of 5%. Model calibration was assessed to evaluate the agreement between expected and observed outcomes.

**Results**: Premature delivery was observed in 182 (40.3%) cases in the European cohort and in 345 (60.3%) cases in the Dutch cohort. Short‐term predictive performance for birth within one week yielded an AUC of 0.84 (95% CI 0.79‐0.89, European cohort) and 0.75 (95% CI 0.67‐0.83, Dutch cohort). Short‐term outcomes, specifically birth before 30 weeks, demonstrated higher performance compared to longer‐term outcomes (birth before 37 weeks). The highest AUC, 0.91 (95% CI 0.86‐0.95), was achieved for the qfFN and CL model predicting sPTB before 30 weeks in the European cohort. Calibration was excellent for patients with negligible risk; however, for women at greater risk of PTB, the predicted risk deviated from the observed event rate.

**Conclusion**: The combination of CL and qfFN test in the QUiPP app effectively identifies women at short‐term risk of sPTB in a population outside the UK. Conversely, the model relying solely on CL measurement exhibits inferior performance compared to the other models.

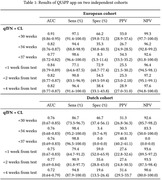

## 36 | Cerclage in low risk pregnancies with short cervix: A randomized controlled trial

Rupsa C. Boelig^1^, Chiara Tersigni^2^, Nicoletta Disimone^3^, Gabriele Saccone^4^, Fabio Facchinetti^5^, Vincenzo Berghella^6^

^1^Sidney Kimmel Medical College, Thomas Jefferson University, Philadelphia, Pennsylvania; ^2^IRCCS Fondazione Policlinico Agostino Gemelli, IRCCS Fondazione Policlinico Agostino Gemelli, Lazio; ^3^Humanitas University, Milan, Lombardia; ^4^University of Naples Federico II, Naples, Campania; ^5^University of Modena and Reggio Emilia, Modena, Emilia‐Romagna; ^6^Sidney Kimmel Medical College of Thomas Jefferson University, Philadelphia, Pennsylvania

10:45–11:00

**Objective**: We aimed to conduct a pragmatic randomized controlled trial to evaluate the additive benefit of cerclage with vaginal progesterone compared to routine care (vaginal progesterone alone) in singletons without a prior spontaneous preterm birth (sPTB) but with a current mid‐trimester transvaginal ultrasound (TVU) short cervical length (CL).

**Study Design**: This is a multicenter international randomized controlled trial from 09/2017 to 09/2023. Singleton pregnancies received TVU CL screening during the mid‐trimester anatomy ultrasound. Inclusion criteria was TVU CL ≤25mm at 18 0/7–23 6/7 weeks. Exclusion criteria included current or planned cerclage, cervical dilation, symptoms of labor, infection, bleeding, or rupture of membranes at screening. Participants were randomized 1:1 to cerclage and vaginal progesterone versus routine care (vaginal progesterone alone), stratified by study site and TVU CL ≤15mm. Primary outcome was PTB < 35 weeks with intention to treat analysis. Secondary outcomes included PTB < 37, 32, 28, 24 weeks and gestational age at delivery. Categorical variables compared with Pearson Chi‐Square and relative risk estimate; continuous with Mann Whitney U test. Planned enrollment was *N* = 206 based on an estimated 0.54 relative risk with cerclage and 34% incidence of preterm birth with standard care.

**Results**: Study was halted in September 2023 due to significantly lagging enrollment. 93 participants were randomized from 2017‐2023, three did not have outcome data. Of 90 included, 40 (44.4%) had a CL≤15mm. There was no significant difference in PTB rate between groups, although those randomized to cerclage with progesterone had reduced rates of PTB at various gestational ages, as well as a significantly later gestational age at delivery (Table).Results were similar in the subgroups of cervical length ≤15mm and >15mm.

**Conclusion**: In singletons without prior sPTB and a TVU CL ≤25mm before 24 weeks, cerclage in addition to progesterone leads to a later gestational age at delivery and may reduce the rate of preterm birth, compared to progesterone alone.

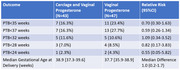

## ORAL CONCURRENT SESSION 8 ‐ PRENATAL GENETICS AND ULTRASOUND

Saturday, 28 September 2024 09:00–11:15

## 37 | Expanded carrier screening for genetic conditions with clinical trials in‐utero therapy

Billie R. Lianoglou^1^, Vivienne Souter^2^, Brittany Prigmore^2^, Madeleine Armer‐Cabral^2^, Elizabeth Repass^2^, Yang Wang^2^, Tippi C. MacKenzie^3^

^1^Center for Maternal‐Fetal Precision Medicine, University of California San Francisco, San Francisco, California; ^2^Natera, Inc., San Carlos, California; ^3^University of California, San Francisco, San Francisco, California

10:00–10:15

**Objective**: Molecular fetal therapies for monogenic conditions are an emerging area of care with the potential to transform outcomes. Identifying pregnancies eligible for treatment is challenging for conditions with no ultrasound manifestations. We evaluated carrier frequency for 10 conditions identifiable through reproductive carrier screening, focusing on conditions with active or planned in‐utero (IU) clinical trials.

**Study Design**: This retrospective observational study included carrier screening tests from female patients, aged 18‐55 years, ordered from a US commercial laboratory (Jan 2020‐Sept 2022). We calculated the proportion of samples that included screening for any of 10 conditions with active or planned IU therapy trials and the gene‐specific carrier frequency for 7 autosomal recessive (AR) conditions and 3 X‐linked (XL) conditions (Table 1). To focus on severe subtypes eligible for IU therapy, we excluded pseudodeficiency variants in *IDUA*, *GAA*, and *ARSB* and the neuronopathic protective variant (p.N409S) of Gaucher disease.

**Results**: Of 822,155 samples screened, 228,720 (28%) were for a panel that included *none* of the conditions and were excluded from the analysis. 15,237 (1.9%) were for a panel with 10 of the relevant conditions with active or planned clinical trials of IU therapy. For the 7 AR conditions, the carrier frequency ranged from 1 in 77 for Pompe disease (*GAA*) to 1 in 1693 for MPS VII (*GUSB*) (Table 1). For the XL conditions, the carrier frequency ranged from 1 in 2325 for hemophilia B to 1 in 19274 for Hunter syndrome (*IDS*).

**Conclusion**: Our study highlights that carriers for these individually “rare” conditions are, collectively, not uncommon. However, many patients have screening with panels that detect *none* of the conditions and few have panels that detect all. As fetal and early postnatal treatment options expand for rare genetic conditions, we should reconsider the paradigm of screening (currently based on carrier frequency) to also include conditions with actionable findings.

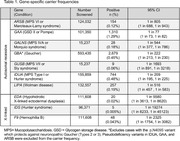

## 38 | Early life course medicine: Epigenome‐wide non‐invasive prenatal testing (the epi‐NIPT‐study), the Rotterdam periconception cohort

Marjolein van Vliet^1^, Ruben Boers^1^, Joachim Boers^1^, Olivier Schaffers^1^, Lotte van der Meeren^1^, Regine Steegers‐Theunissen^1^, Joost Gribnau^1^, Sam Schoenmakers^2^

^1^Erasmus University Medical Center, Erasmus University Medical Center, Zuid‐Holland; ^2^Erasmus University Medical Centre, DEN HAAG

10:15–10:30

**Objective**: Placental originated cell free DNA (cfDNA) could lead to unique opportunities to non‐invasively study (epi)genetic programming of the placenta during pregnancy, but studies investigating genome‐wide methylation of maternal cfDNA are scarce. We aim to investigate the impact of pregnancy on the maternal cfDNA methylome and identify placental‐specific methylation marks in maternal cfDNA.

**Study Design**: The innovative methylated DNA sequencing (MeD‐seq) assay was used to detect differentially methylated regions (DMRs). MeD‐seq is compatible with low amounts of cfDNA and has a high genome‐wide coverage. DMRs were identified between cfDNA from pregnant women (end of first trimester) and non‐pregnant healthy blood donors (HBDs). Overlap of DMRs with DNA methylation profiles of first trimester placental tissues and buffy coats, another major source of maternal cfDNA, was studied to explore the tissue‐of‐origin of identified DMRs. In addition, known placental markers such as hypermethylation of the *RASSF1* promoter region and Y‐chromosomal methylation marks in maternal cfDNA were investigated.

**Results**: We identified >400 DMRs between cfDNA from pregnant women (*n* = 10) and HBDs (*n* = 6). Although >70% of DMRs in maternal cfDNA overlapped with methylation profiles in buffy coats (*n* = 20), we identified 30 DMRs that specifically overlapped with DNA methylation in placental tissues (*n* = 10). These novel DMRs were stronger placental markers in maternal cfDNA than the well‐known hypermethylation of *RASSF1*. Fetal sex could be determined based on Y‐chromosomal DNA methylation in maternal cfDNA.

**Conclusion**: Genome‐wide methylation profiling of first trimester maternal cfDNA using MeD‐seq can detect placental DNA methylation marks and determine fetal sex. The epi‐NIPT study supports future research into epigenome‐wide analyses of cfDNA in early prenatal (epi)genetic testing, and in the context of obstetric health and disease. The combined study of (epi)genetics in maternal cfDNA could lead to the development of epigenome‐wide non‐invasive prenatal testing (epi‐NIPT).

## 40 | Fetal growth restriction and autism spectrum disorder in childhood: Investigating a potential contributory association

Amir Snir^1^, Omri Zamstein^2^, Tamar Wainstock^3^, Eyal Sheiner^3^

^1^Soroka University Medical Center, Omer, HaDarom; ^2^Soroka University Medical Center, Soroka University Medical Center, HaDarom; ^3^Department of Obstetrics and Gynecology, Soroka University Medical Center, Ben‐Gurion University of the Negev, Beer‐Sheva, Israel., Beer Sheva, HaDarom

10:45–11:00

**Objective**: Autism spectrum disorder (ASD) is a prevalent neurodevelopmental disorder in which prenatal factors are hypothesized as risk factors. In light of existing literature suggesting a potential link between fetal growth restriction (FGR) and long‐term neurological abnormalities, our study aimed to investigate the potential association between FGR and ASD during childhood.

**Study Design**: A population‐based cohort analysis was performed, comparing the occurrence of ASD in offspring (data obtained from community settings and hospitalization codes) who were diagnosed with FGR during pregnancy versus those who were not. The database consisted of deliveries that took place from 2005 to 2017 (after which ASD diagnoses decreased due to the time required for a definitive diagnosis). A Kaplan‐Meier survival curve was utilized to evaluate the cumulative incidence of ASD, and a Cox proportional hazards model was developed to account for potential confounding variables.

**Results**: Out of 115,081 pregnancies included in the study, 2,320 (2.0%) were complicated with FGR. These pregnancies exhibited distinct characteristics, including earlier delivery (37.1 vs. 39.0 gestational weeks), a higher likelihood of cesarean deliveries (35.3% vs. 15.1%), and other concerning obstetrical features such as non‐reassuring fetal heart rate tracing and a 5‐minute Apgar score < 7 (*p* < 0.001 for all). The prevalence of ASD was found to be twofold higher among offspring who had experienced FGR (1.0% vs. 0.5%, *p* < 0.001; table). Nevertheless, when considering cumulative ASD incidence, there was no significant difference between the two groups (log‐rank p‐value = 0.969). Likewise, after accounting for various confounders such as maternal age, gestational age, and mode of delivery, the association between FGR and ASD was statistically non‐significant (adjusted HR = 1.04, 95% CI 0.72–1.52, *p* = 0.827; table).

**Conclusion**: Despite previous indications in the literature regarding the possible association between FGR and long‐term neurological abnormalities, our findings suggest that FGR may not be a significant risk factor for the development of ASD.

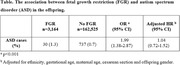

## 41 | Prospective multi‐center international validation of the FMF growth curves in twins

Nadav Kugler^1^, Hamutal Meiri^2^, Nataly Sharon^1^, Ran Svirsky^3^, Richard Brown^4^, Heidy Portillo Rodriguez^4^, Anna Goncé^5^, MAR BENNASAR^6^, Antony Borrell^7^, Julia Ponce^8^, Annegret Geipel^9^, Adeline Walter^9^, Corrina Simonini^9^, Elisa Bevilacqua^10^, Federica Romanzi^11^, Oliver Kagan^12^, Tanja Lennartz^13^, Armin Bauer^14^, Ron Maymon^1^, Kypros NICOLAIDES^15^, Yoram Louzon^16^

^1^Department of Obstetrics and Gynecology, Shamir (Assaf Harofeh) Medical Center, Zerifin, Tel Aviv; ^2^Department of Obstetrics and Gynecology, Shamir (Assaf Harofeh) Medical Center, TeleMarpe, Tel Aviv; ^3^Medical Genetic Unit, Department of Obstetrics and Gynecology, Samson Assuta Ashdod University Hospital, Ashdod, HaMerkaz; ^4^Division of Maternal Fetal Medicine, Department of Obstetrics & Gynaecology, McGill University, McGill University Health Centre, Montreal, Quebec; ^5^Hospital Clínic de Barcelona, Barcelona, Catalonia; ^6^BCNatal ‐ Barcelona Center for Maternal ‐ Fetal and Neonatal Medicine (Hospital Clínic and Hospi tal Sant Joan de Deu), IDIBAPS, University of Barcelona, Center for Biomedical Research on Rare Diseases (CIBER ‐ ER) Barcelona, Spain, Hospital Clínic of Barcelona, Catalonia; ^7^Fetal‐Maternal Medicine Department. BCNatal., Hospital Clinic, University of Barcelona, Barcelona, Catalonia; ^8^BCNatal. Hospital Clínic and Hospital Sant Joan de Déu, Hospital Clínic of Barcelona. Spain, Catalonia; ^9^Department of Obstetrics and Prenatal Medicine, University Hospital Bonn, Bonn, Nordrhein‐Westfalen; ^10^Department of Women, Children, and Public Health Sciences, IRCCS Agostino Gemelli University Polyclinic Foundation, Catholic University of the Sacred Heart, Rome, Lazio; ^11^Fondazione Policlinico Universitario Agostino Gemelli IRCCS, Rome, Italy, Rome, Lazio; ^12^Fetal Medicine Unit, Department of Obstetrics and Gynecology, University Hospital Tuebingen, Tübingen, Baden‐Wurttemberg; ^13^Maternal Fetal Medicine Unit, Department of Obstetrics and Gynecology, University Hospital Tuebingen, Tübingen, Baden‐Wurttemberg; ^14^Maternal Fetal Medicine Unit, Department of Obstetrics and Gynecology, University Hospital Tuebingen, Bonn, Baden‐Wurttemberg; ^15^The Fetal Medicine Research Institute, King's College Hospital, London, England; ^16^Department of Mathematics, Bar Ilan University, Ramat Gan, Tel Aviv

11:00–11:15

**Objective**: 1) To prospectively derive a reference chart of the estimated fetal weight (EFW) with advancing gestational age in monochorionic diamniotic (MCDA) and dichorionic (DC) twins in term pregnancies; 2) To test EFW variability between ethnicities and national women origins, and 3) To examine similarities with the growth charts of the Fetal Medicine Foundation (FMF)

**Study Design**: Gestational age and chorionicity‐specific reference distributions for EFW, were derived from 540 twin pregnancies (385 DC and 154 MCDA) from Montreal (Canada), Bonn and Tubingen (Germany), Barcelona (Spain), Rome (Italy), and Zerifin (Israel). Included were twin pregnancies with 2 normal livebirths at ≥37+0 (DC) and ≥36+0 (MCDA) weeks’ gestation. Pregnancy dating was determined by crown‐rump length of the larger twin in the 1^st^ trimester. The EFW obtained from ultrasound measurements of biparietal diameter (BPD), head circumference (HC), abdominal circumference (AC), and femur length (FL), using Hadlock formula. In each pregnancy, 6 measurements were taken at 11‐13, 20‐22, 24‐26, 28‐30, 32‐34, and 35‐36 weeks’ gestation. The EFW differences between ethnic groups and centres compared by ANOVA on the measurement between respective groups, and at each time point. Prediction of the birth weight estimated from the EFW in the last 3 time points

**Results**: A slower growth of MCDA compared to DC twins started from 28 weeks’ gestation and increased with advancing gestation. The growth patterns in both DC and MCDA twins were similar but mildly slower than those reported by the FMF. There were also small differences in growth between participating centres, with smallest babies in Spain and Italy and largest in Germany (140 grams maximum difference between centres at 36.5 weeks’ gestation), and between ethnicities, with babies of white women being bigger (165 grams for the 50^th^ centile at 36.5 weeks)

**Conclusion**: Our findings of fetal growth in DC and MCDA twins are consistent with the FMF chart and demonstrate small yet significant differences in growth patterns between different countries and ethnicities.

## POSTER SESSION 1

Thursday, 26 September 2024 12:15–13:30 CET

## 42 | Obstetrical outcomes in pregnancies with congenital heart disease: Role of risk stratification and care model

Tehila Avitan^1^, Amiram Nir^2^, Alexander Ioscovich^3^, Daniel Shatalin^2^, Eli Heifetz^2^

^1^Shaare Zedek Medical Center, Jerusalem; ^2^Shaare Zedek Medical Center, jerusalem, Yerushalayim; ^3^SZMC, Shaare Zedek Medical Center, Yerushalayim

12:15–13:30

**Objective**: To compare maternal and neonatal outcomes between pregnant women with maternal congenital heart disease (CHD) receiving routine obstetric care for low‐risk cardiac conditions (WHO class 1‐2, control group) and those receiving multidisciplinary care for more complicated cardiac conditions (WHO class 2‐3, study group).

**Study Design**: A retrospective cohort study evaluated 1640 pregnant women with CHD, consisting of 1476 in the routine care control group (WHO 1‐2) and 164 in the multidisciplinary care study group (WHO 2‐3). Maternal and neonatal outcomes were compared between groups.

**Results**: The routine care control group had significantly higher mean gestational age (38.8 vs 38.2 weeks, *p* = 0.004) and lower rates of nulliparity (18% vs 32%), preterm delivery < 37 weeks (9% vs 18%), induction of labor (13% vs 24%), cesarean delivery (20% vs 28%), instrumental delivery (5% vs 13%), and epidural use (49% vs 66%) compared to the multidisciplinary study group (all *p* < 0.05). Second stage duration was shorter in the control group (27.1 vs 38.8 min, *p* = 0.03). Neonates in the control group had higher mean birth weights (3177.8 g vs 2959.5 g) and lower rates of small for gestational age (9% vs 17%) (both *p* < 0.001). No differences in Apgar scores or NICU admissions were observed.

**Conclusion**: For pregnant women with low‐risk cardiac conditions (WHO 1‐2), routine obstetric care was associated with improved maternal and neonatal outcomes compared to the high risk cardiac pregnancies. However, for high‐risk cardiac cases (WHO 2‐3), multidisciplinary care is needed given the increased risk of complications. Routine care appears appropriate for low‐risk CHD pregnancies, but a specialized, multidisciplinary approach is warranted for more complex cardiac conditions.

## 43 | Rule‐in and rule‐out of pre‐eclampsia using a novel, whole‐blood, point‐of‐care placental growth factor test

Alice Hurrell^1^, James Rogers^1^, Gaayen R. Sahgal^2^, Louisa Samuels^1^, Chileshe Mabula‐Bwalya^3^, Katy Kuhrt^4^, Antonio De Marvao^2^, Paul T. Seed^1^, Andrew Shennan^1^, Kate Bramham^2^

^1^King's College London, King's College London, England; ^2^King's College London, London, England; ^3^King's College London, London, District of Columbia; ^4^King's College London, hartfield, England

12:15–13:30

**Objective**: To determine test performance for prediction of pre‐eclampsia for a novel, whole‐blood, point‐of‐care placental growth factor (PlGF) test–LEPZI ® Quanti PLGF.

**Study Design**: 243 samples from women with suspected or confirmed pre‐eclampsia were analysed from a prospective cohort study.

Serum PlGF concentrations were quantified using the LEPZI ® Quanti PLGF test. Sensitivity, specificity, positive predictive value (PPV), and negative predictive value (NPV) were determined. Test performance was compared to the DELFIA ® Xpress PlGF1‐2‐3.

**Results**: 243 pregnant participants were included; 126 participants received PlGF testing before 34 weeks’ gestation and 117 participants received PlGF testing after 34 weeks’ gestation. For test performance for prediction of pre‐eclampsia within seven days of sampling (all gestations), PlGF < 12 pg/ml had a sensitivity of 66.7% (95% confidence interval (CI) 46.0‐83.5), specificity 81.5% (75.7‐86.4), PPV 31.0% (19.5‐44.5) and NPV 95.1% (91.0‐97.8); PlGF ≥100 pg/ml had a sensitivity of 77.8% (55.7‐91.4), a specificity of 65.7% (59.0‐72.1), PPV 22.1% (14.2‐31.8) and NPV 96.0% (91.4‐98.5). This is comparable to the NPV for the DEFLIA Xpress PlGF test–a threshold of ≥150 pg/ml had a NPV of 92.8% (85.7‐97.0) (before 34 weeks’ gestation).^1‐4^

**Conclusion**: This study demonstrates promising test performance for a whole‐blood, point‐of‐care PlGF test for prediction of pre‐eclampsia, comparable to validated PlGF tests, recommended by national guidance. The promising emerging advent of point‐of‐care PlGF testing means that we are on the tipping point of a diagnostic adjunct become more readily available beyond high‐income settings.

## 44 | Pre‐cerclage cervical dilation and pregnancy outcomes

Kristine E. Brown^1^, Elizabeth Vitaro^2^, Selin Everett^2^, Shai Bejerano^3^, Uma M. Reddy^3^, Mirella Mourad^3^, Maria Andrikopoulou^4^

^1^New York Presbyterian/Columbia, New York, NY; ^2^Columbia University Medical Center, New York, NY; ^3^Columbia University Medical Center, New York, New York; ^4^Columbia University Irving Medical Center, New York, New York

12:15–13:30

**Objective**: Exam‐indicated cerclage (EC) is an important treatment for cervical insufficiency with the goal of preventing preterm birth (PTB). The relationship between the degree of pre‐cerclage cervical dilation and ultimate pregnancy outcomes is unknown.

**Study Design**: A retrospective cohort study was conducted at a single academic center from 2015‐2023. Patients with a dilated cervix < 24 weeks gestation (GA) recommended for EC were included. Multiple gestations, prior PTB, and incomplete delivery data were excluded. Charts were reviewed to identify the preoperative cervical exam and pregnancy characteristics for each patient. The primary outcome was GA at delivery. Secondary outcomes included latency from EC to delivery and rates of PTB, PPROM, intraamniotic infection (IAI), NICU admission and neonatal demise. A spearman correlation analysis was conducted to assess whether there is a correlation between cervical dilation and GA at delivery. A linear regression model with robust standard errors was fit for multivariable analysis of the primary outcome.

**Results**: 125 patients were identified. Median dilation prior to EC was 1 cm and median GA of EC placement was 21.1 weeks. 84 patients received concurrent vaginal progesterone (67%). The median GA at delivery was 37.3 weeks (IQR 31.9‐39.1). There was a trend towards earlier GA at delivery for those with greater preoperative dilation, though this was not statistically significant (ρ = −0.17, *p* = 0.06). These results were similar after adjustment for GA at cerclage placement, progesterone use, and medically‐indicated preterm delivery (*p* = 0.13). Additional pregnancy outcomes are included in table 1. 11 patients were diagnosed with IAI prompting delivery (8.8%). The rate of previable delivery was 9.6% and neonatal demise 10.4%

**Conclusion**: We found no significant correlation between the degree of cervical dilation prior to EC and GA at delivery. However, clinically important pregnancy outcomes such as IAI, previable delivery, and neonatal demise remain elevated in this population. These results may be considered in counseling patients who may undergo exam‐indicated cerclage.

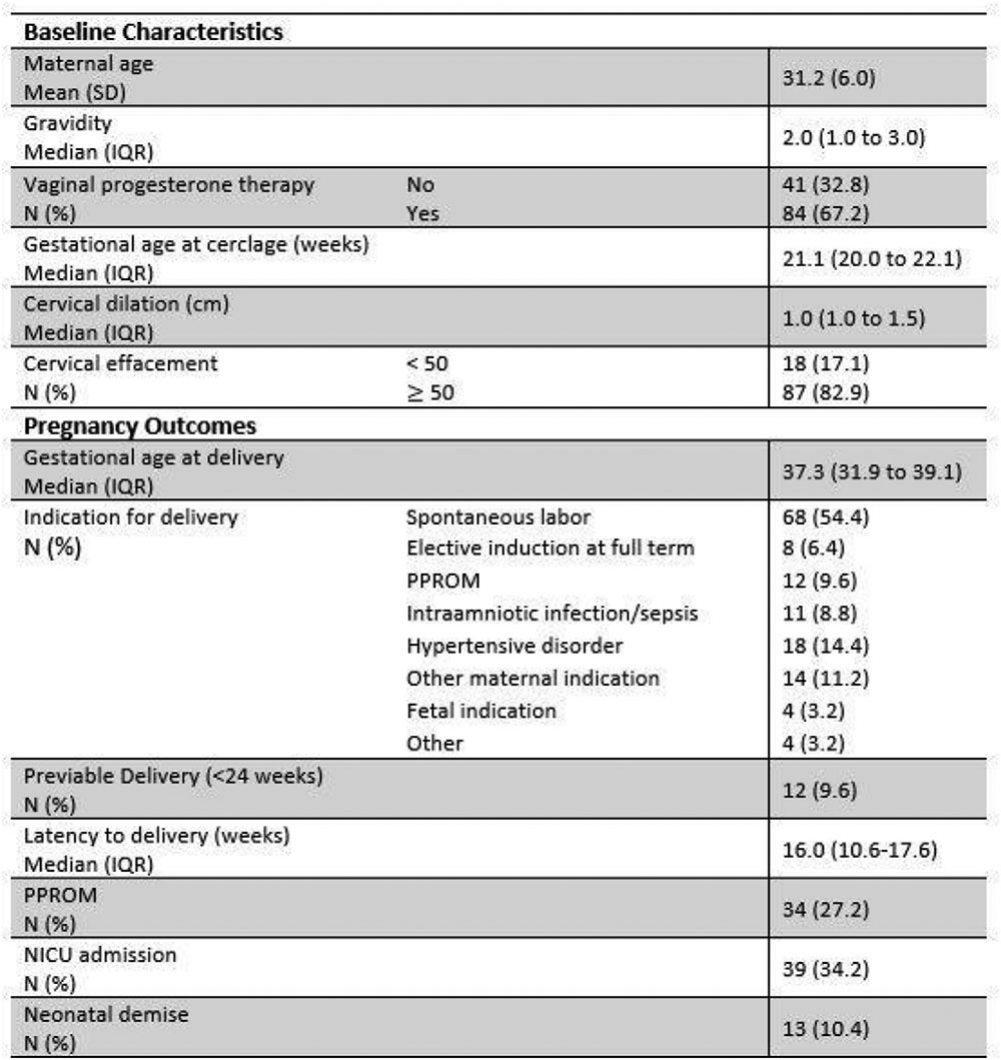

## 46 | Are polygenic risk scores associated with hypertensive disorders of pregnancy in a 2nd pregnancy?

Jenifer A. Akinduro^1^, David M. Haas^2^, Rebecca B. McNeil^3^, Michael C. Honigberg^4^, William A. Grobman^5^, Rafael Guerrero^6^, Janet Catov^7^

^1^Indiana University, Indianapolis, IN; ^2^Indiana University School of Medicine, Indianapolis, Indiana; ^3^Research Triangle International, Durham, North Carolina; ^4^Harvard Medical School, Boston, Massachusetts; ^5^The Ohio State University, Columbus, Ohio; ^6^North Carolina State University, Raleigh, North Carolina; ^7^Magee‐Womens Hospital, Pittsburgh, Pennsylvania

12:15–13:30

**Objective**: Hypertensive disorder of pregnancy (HDP) recurrence across pregnancies is well established, but the contribution of pre‐pregnancy factors such as genetic risk is unknown. The objective of this study was to determine whether a polygenic risk score for HDP is associated with the development of HDP in a 2nd pregnancy, independent of HDP history in the first pregnancy.

**Study Design**: This was a retrospective study of data from the nuMoM2b study, including only those participants who had a pregnancy subsequent to the nuMoM2b pregnancy. We excluded those with pre‐existing chronic hypertension (cHTN) in the nuMoM2b pregnancy. Poisson regression with robust standard errors was used to estimate the association between PRS and developing HDP in a 2^nd^ pregnancy.

**Results**: Ancestry‐adjusted PRS (aaPRS) were available for 5357 study subjects, with 135 of these participants being excluded from this analysis due to pre‐existing cHTN. Of the 5222 individuals in the study population, 2511 had no reported second pregnancy, leaving a final sample size of 2711 for analysis. Among the 2711 participants, 644 (23.7%) had HDP in their nuMoM2b pregnancy and 207 (7.6%) had HDP in their 2^nd^ pregnancy. 205 (7.6%) identified as African American, 249 (9.2%) identified as Hispanic, 6 (0.2%) identified as Asian, 74 (2.7%) as multiracial/multiracial/another self‐reported race, and 2177 (80.3%) identified as White. Figure 1 displays the rates of HDP in the 2^nd^ pregnancy for participants by aaPRS tertile separated by HDP in the nuMoM2b pregnancy. Using Poisson regression, aaPRS was independently associated with developing an HDP in G2 after accounting for HDP and early‐pregnancy BMI in G1 (b = 0.161, CI 0.0302, 0.2918, *p* = 0.0158).

**Conclusion**: Among participants who had an HDP in G1 and had a high‐risk PRS, there is a statistically significant increased risk of developing an HDP in G2. Furthermore, ancestry‐adjusted PRS is independently associated with developing HDP in 2^nd^ pregnancy after controlling for the presence of HDP in the first pregnancy.

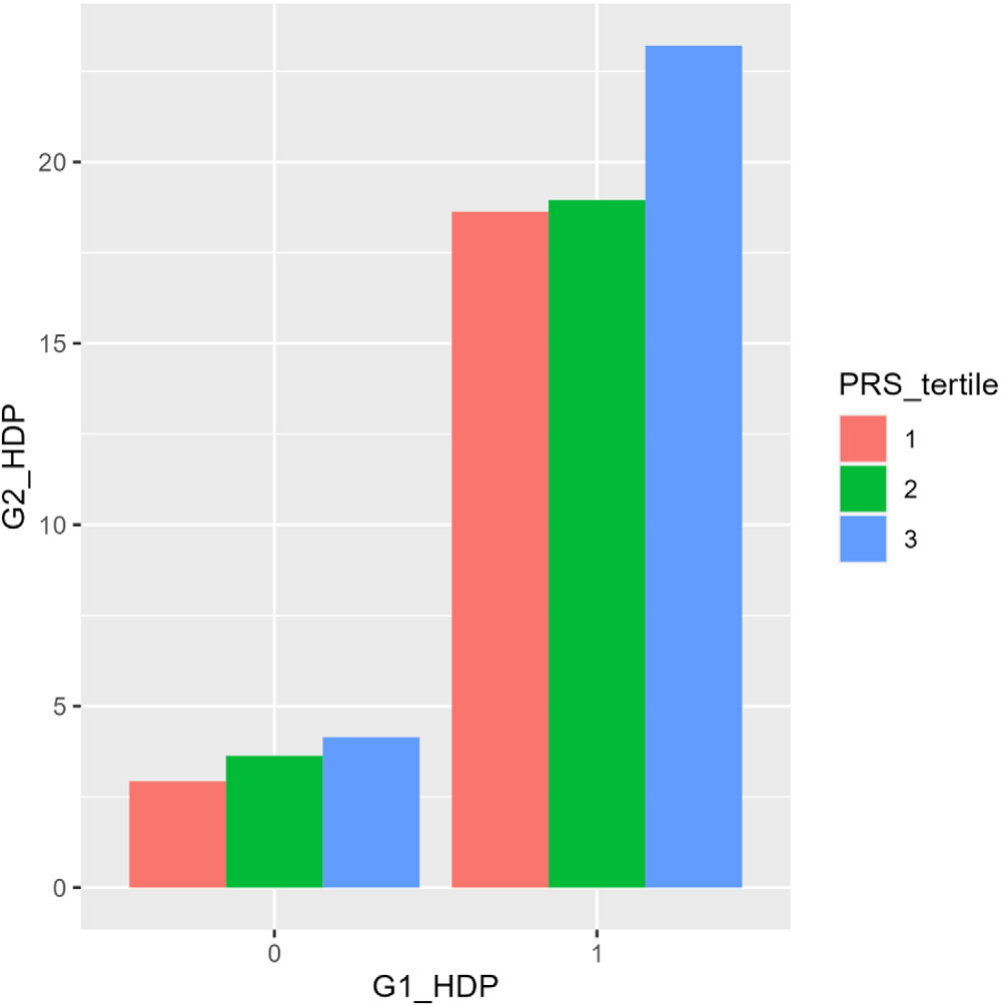

## 47 | Improving breastfeeding rates in low obstetric risk women: A meta‐analysis of mid‐led interventions

Gloria Anderson^1^, Giorgia Gabrielli^1^, Giulia Tedesco^1^, Erika Colagiovanni^1^, Ilaria Pasqua^1^, Maria Luisa Rega^2^, Jessica Preziosi^3^, Michelangela Danza^4^, Francesca Rizzi^1^

^1^Fondazione Policlinico Agostino Gemelli, IRCCS, Rome, Italy, Fondazione Policlinico Agostino Gemelli, IRCCS, Rome, Italy, Lazio; ^2^Università Cattolica del Sacro Cuore, Rome, Italy, Università Cattolica del Sacro Cuore, Rome, Italy, Lazio; ^3^Fondazione Policlinico Agostino Gemelli, IRCCS, Rome, Italy, Fondazione policlinico Agostino Gemelli, Lazio; ^4^Università Cattolica del Sacro Cuore, Rome, Italy, Università Cattolica del Sacro Cuore, Lazio

12:15–13:30

**Objective**: To evaluate the effectiveness of mid‐led or nurse‐led designed to enhance Breastfeeding Self‐Efficacy (BSE) on exclusive breastfeeding rates in women at low obstetric risk.

**Study Design**: We performed a systematic review and meta‐analysis of randomized clinical trials published from 2003 to March 2024, following the Cochrane Handbook for systematic review and the PRISMA guidelines. Studies administering nurse or mid‐led intervention to pregnant or postpartum women at low obstetric risk and without neonatal complications were included. Interventions administered must directly involve a midwife and/or nurse(s). Risk of bias was assessed with the Cochrane's Rob2.

**Results**: We included twenty‐two studies, of which 13 was also included in the meta‐analysis. Most of the studies (86%) were published in the last ten year either in Asia (41%) or in the Middle East (32%). All the articles included in the meta‐analysis except one were rated at low risk of bias. Two‐third of the studies were nurse‐led and only one‐third were mid‐led. 59.1% of the interventions were mono‐component (76.9% educational vs 23.1% counselling), while the remaining (40.9%) were multi‐component (educational + counselling/support). Most of the interventions (77.3%) were performed in two or more sessions during pregnancy or in the immediate post‐partum. We found an increase on exclusive breastfeeding rates in the intervention group compared to usual care at 1 month (OR = 1. 84; 95% CI 1.30;2.60; I2 = 0%, *p* = 0.79) and at 2 months (OR = 2.9; 95% CI 1.73;4.87; I2 = 52.9%, *p* = 0.05). Statistically significant and extremely relevant is the difference in the rate of exclusive breastfeeding at 6 months (OR = 2.77; 95% CI 1.05;7.34; I2 = 70%, *p* = 0.01).

**Conclusion**: Similarly to previous studies, our results confirm the importance of interventions by experienced nurses or midwives toward breastfeeding self‐efficacy in order to improve exclusive breastfeeding rates. Indeed, women who received a midwife‐ and/or nurse‐led intervention were significantly more likely to breastfeed exclusively than women in the control group.

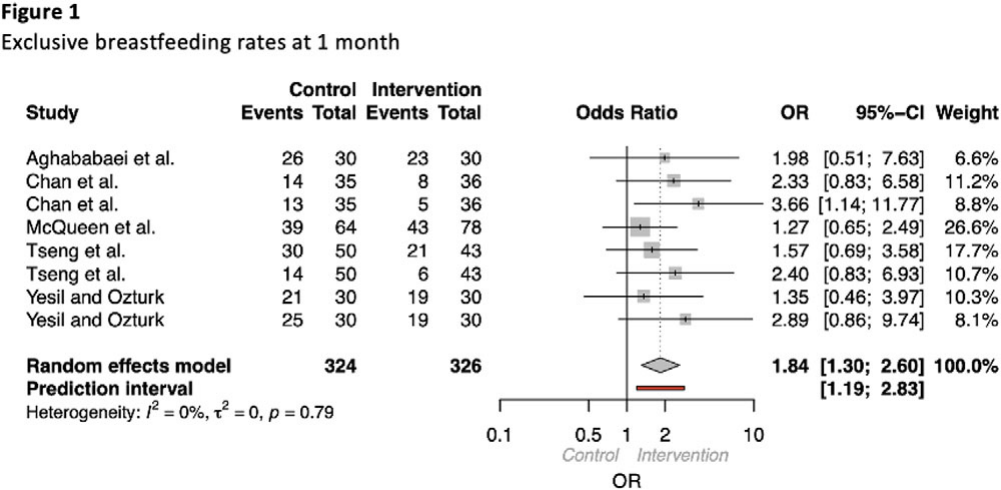

## 48 | Effectiveness and safety of antenatal treatments for Fetal and Neonatal Alloimmune Thrombocytopenia (FNAIT)

Pamela Baker^1^, Paige Meizlik^2^, May Lee Tjoa^3^

^1^Janssen Research & Development, LLC, Spring House, PA; ^2^Janssen Research & Development, LLC, Spring House, Pennsylvania; ^3^Janssen Global Services, LLC, Cambridge, Massachusetts

12:15–13:30

**Objective**: FNAIT is a rare disease caused by maternal alloimmunization to human platelet antigens (HPAs) that can cause bleeding complications, such as intracranial hemorrhage (ICH), in fetuses and neonates. Intravenous immunoglobulin (IVIg) treatment can be used to treat FNAIT, but data are limited. This study evaluated literature on IVIg effectiveness/safety for FNAIT treatment.

**Study Design**: A systematic literature review (SLR) was conducted using key databases (Embase and MEDLINE, MEDLINE In‐Process, The Cochrane Library), gray literature, clinical trial registries, and bibliographic searches. Data were extracted from randomized controlled trials (RCTs), non‐RCTs, and prospective/retrospective observational studies that included women who experienced FNAIT in a previous pregnancy and received any antenatal treatment. Searches were conducted on 15Feb2022.

**Results**: 43 publications met SLR inclusion criteria (4 RCTs, 38 observational studies [79% retrospective], 1 single‐arm trial). There were 14 unique treatment combinations including IVIg (IVIg only: *n* = 38; IVIg+steroids: *n* = 22; IVIg+intrauterine platelet transfer [IUPT]: *n* = 11; IVIg+IUPT+steroids: *n* = 6). Six unique IVIg dosing regimens were reported, with 1 g/kg per week being most common (29 [70%]). Neonatal platelet counts were reported in 29 (67%) studies using varied thresholds; most (*n* = 21) recorded platelet counts < 50×10^9^/L. 28 (65%) studies had neonatal ICH as an outcome; 15 reported no ICH events. Other bleeding outcomes were reported infrequently (hematoma: 2 [5%]; non‐ICH bleeding: 12 [28%]). Safety/tolerability data were limited; 3 (7%) reported AEs and 3 (7%) reported discontinuations. Preterm birth was reported in 12 (28%) studies. Neonatal mortality was reported in half of the studies and rates were < 20%.

**Conclusion**: This SLR of the FNAIT clinical literature suggests that noninvasive treatments (IVIg/steroids) have been widely used to prevent ICH and bleeding‐related fetal/neonatal morbidity and mortality; however, limited and variable reporting of IVIg effectiveness/safety indicates a clear gap in the literature that warrants further research.

## 49 | Comparison of perinatal outcomes among women undergoing induction of labor with and without epidural analgesia

RENANA BEN SHUSHAN AMOR^1^, Adi Y Y. Weintraub^2^, Alla Saban^3^, Yael Baumfeld^4^, Merav Jacobs^4^, Zehava Yohay^5^, Tamar Eshkoli^2^

^1^Faculty of Health Sciences, Ben‐Gurion University of the Negev, Beer Sheba, Israel, ROSH HA'AIN, HaMerkaz; ^2^Soroka University Medical Center, Soroka University Medical Center, HaDarom; ^3^Soroka University Medical Center, Beer Sheva, HaDarom; ^4^Soroka University Medical Center, Beer sheba, HaDarom; ^5^Pain Unit, Soroka University Medical Center, Beer Sheva, Israel, Beer Sheva, HaDarom

12:15–13:30

**Objective**: To investigate pregnancy complications and adverse perinatal outcomes among women who underwent induction of labor (IoL) with and without epidural analgesia (EA).

**Study Design**: In this retrospective population‐based cohort study, all women who underwent IoL and delivered between the years 1996‐2016 in a tertiary medical center were included. The patients were divided into those who did and did not use EA (study and comparison groups, respectively). Multiple pregnancies, gestational age below 34 0/7, congenital malformations, and those lacking prenatal care were excluded from the analysis. Clinical, obstetrical, and demographical characteristics were collected. Pregnancy complications and adverse perinatal outcomes were compared between the groups. Univariate analysis was followed by a multivariate analysis to control for confounders. A *p*‐value of < 0.05 was considered statistically significant.

**Results**: During the study period, 71,173 patients met the inclusion criteria, and 21,091 (29.6%) of them delivered with EA. Women who underwent IoL with EA had higher rates of non‐progressive labor in the first (NPL1) and second (NPL2) stages of labor (5.3% vs. 3.5% for NPLl, 3.9% vs. 2.3% for NPL2; *p* < 0.001), operative deliveries (7.6% vs. 4.2%; *p* < 0.001), and postpartum hemorrhage (PPH) (0.9% vs. 0.7%; *p* = 0.01) than those who underwent IoL without EA. In contrast, neonatal outcomes of women who underwent IoL with EA were favorable with significantly lower rates of non‐reassuring fetal heart rate (NRFHR) tracings (1.5% vs. 2%; *p* < 0.001) and perinatal mortality (0.9 % vs. 1.6%; *p* < 0.001). Using a multivariate analysis, EA was found to be an independent risk factor for operative deliveries (OR 1.25, CI 95% 1.16‐1.34; *p* < 0.001) after controlling for maternal age, nulliparity, hypertensive disorders of pregnancy, and fertility treatments.

**Conclusion**: Among women who underwent IoL, EA was significantly associated with higher rates of maternal complications. However, after controlling for confounders, EA was found to be independently associated with operative deliveries.

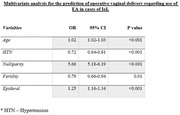

## 50 | Randomized feasibility study of labor induction with baloon catheter, misoprostol or both ‐ IDOM trial

Inna Bleicher^1^, Dafna Ben Yehuda Raz^1^, Dmitry Basov^2^, Mayson Einas Khniefes^3^, Shlomi Sagi^4^, Sharon Einav^5^, Rami sammour^4^

^1^Bnai Zion Medical Center, Haifa, Hefa; ^2^Bnai Zion Medical Center, Bnai Zion Medical Center, Hefa; ^3^Bnai Zion Medical Center, Bnai Zion Medical Center, HaZafon; ^4^Bnai Zion MC, Bnai Zion MC, Hefa; ^5^Shaare Zedek Medical Center, Shaare Zedek Medical Center, Yerushalayim

12:15–13:30

**Objective**: To examine the feasibility of a randomized controlled trial comparing cesarean delivery (CD) rate in patients undergoing induction of labor (IOL) with double balloon for cervical ripening (CRB), oral misoprostol (OM) or both.

**Study Design**: Feasibility trial designed to examine how many patients and centers would be needed for a trial comparing the rate of CD following IOL with CRB, OM or concomitant use of both. We screened patients with a singleton gestation, > 37 gestational weeks, cephalic presentation, Bishop score ≤ 4 and intact membranes. Patients with a maximum vertical pocket < 2, growth restriction < 5^th^ centile, and prior uterine surgery were excluded. After informed consent, patients were randomized to CRB for 6 hours (DB group), 50 mcg OM every 4 hours for maximum of 6 doses (OM group), or concomitant use of both (DOM group). Primary outcome: Rate of CD. Secondary outcomes: Bishop scores, time to delivery, rate of failed induction, maternal and neonatal outcomes, and feasibility parameters.

**Results**: Enrolled were 250 patients per 50 months. Overall, 194 had sufficient data to be analyzed (57‐DB, 67–OM, 70–DOM). CD rates were 10.4%, 17.1% and 19.3% with OM, DB and DOM respectively (NS). DOM patients had a shorter induction to delivery interval, higher 6‐hour Bishop scores than OM patients and lower rates of induction failure than DB patients (Table 1). The average rate of recruitment was 5 patients/month (range 2‐12). Excluded were: 13 protocol violations, 28 patients missing data, 14 patients who withdrew informed consent and 3 with spontaneous labor.

**Conclusion**: Assuming exclusion of one in five randomized patients (56/250), ∼ 600 patients would be needed for a 2‐arm study comparing only OM vs DB. Since the rate of recruitment in a single center was 5 patients/month, 10 medical centers with similar annual delivery rates would have to participate to complete recruitment within a year. In addition, combined mechanical and pharmacological cervical ripening may reduce time to delivery without increasing cesarean delivery rate.

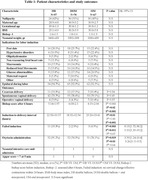

## 51 | Circulating angiogenic factors levels in women with HPD according to the baseline hemodynamic findings

Beatrice Valentini^1^, Elvira Di Pasquo^2^, Andrea Dall'asta^3^, Mariagrazia Capurso^4^, Piernicola D'Amario^4^, Valentina Anna Degennaro^2^, Alessia Casciaro^5^, Tullio Ghi^6^

^1^University of Parma, University of Parma, Emilia‐Romagna; ^2^Azienda Ospedaliero‐Universitaria di Parma, Parma, Emilia‐Romagna; ^3^Department of Medicine and Surgery, Obstetrics and Gynecology Unit, University of Parma, Department of Medicine and Surgery, Obstetrics and Gynecology Unit, University of Parma, Emilia‐Romagna; ^4^University of Parma, Parma, Emilia‐Romagna; ^5^Università di Parma, Università di Parma, Emilia‐Romagna; ^6^Dipartimento di Medicina e Chirurgia, Università di Parma, Parma, Emilia‐Romagna

12:15–13:30

**Objective**: To assess in women with HDP if the predictive value of sFLT‐1/PlGF ratio for adverse outcomes is influenced by the hemodynamic phenotype

**Study Design**: Retrospective study including a cohort of women with new‐onset HDP carrying a singleton viable pregnancy from 22 to 36 gestational weeks. A noninvasive assessment of the main maternal hemodynamic parameters [Cardiac Output (CO), Systemic Vascular Resistance (SVR)] was done upon the hospital admission using USCOM‐1A. The hemodynamic phenotype was classified as “hypodynamic” in case of low CO [ < 5L/min] and/or high SVR [>1400 dynes*s*cm^−5^] or as “non hypodynamic” in case of normal or high CO [>5 L/min] and/or low SVR [ < 1400 dynes*s*cm^−5^]. The values of sFLT‐1 and PlGF were assessed on maternal serum upon hospital admission and their ratio was calculated.

An adverse composite maternal outcome (ACMO) was defined in presence of at least one among the following: severe hypertension or placental abruption or occurrence of end‐organ dysfunction as defined by ISSHP guidelines 2021. A composite of adverse neonatal outcome (ACNO) included birth weight below the 10^th^ percentile (small for gestational age), or fetal/neonatal death

**Results**: Among the 93 women included, 57(61.2%) were categorized as hypodynamic and 36 (38.8%) as non‐hypodynamic. sFLT‐1/PLFG ratio at admission was significantly higher in the former group compare with the latter (301 [93.1‐787] vs. 52.5 [10.0‐257.0]). A significant association between SFLT‐1/PLGF ratio and an ACMO (*p* = 0.02) and an ACNO (*p* = 0.007) was reported only in the group of women defined with a “hypodynamic” profile

**Conclusion**: sFLT1/PlGF ratio is associated with the occurrence of an adverse maternal and neonatal outcome only in women with a hypodynamic profile.

## 52 | The association between abnormal UVF and undetected SGA: Secondary analysis of a multicenter prospective study

Ruben Ramirez Zegarra^1^, Beatrice Valentini^2^, Beatrice Valentini^2^, Ilma Floriana Carbone^3^, Laura Angeli^4^, Francesca M.P. Gigli^5^, Chiara Di Ilio^6^, Olga Barba^7^, Ottavio Cassardo^8^, Enrico M. Ferrazzi^9^, Tullio Ghi^1^

^1^Dipartimento di Medicina e Chirurgia, Università di Parma, Parma, Emilia‐Romagna; ^2^University of Parma, University of Parma, Emilia‐Romagna; ^3^Fondazione IRCCS Ca' Granda Ospedale Maggiore Policlinico, Milano, Lombardia; ^4^Careggi University Hospital, Florence, Toscana; ^5^University of Milan, Milan, Lombardia; ^6^Department of Women and Child Health, Fondazione Policlinico Universitario Agostino Gemelli IRCCS, Roma, Emilia‐Romagna; ^7^University of Parma, Parma, Emilia‐Romagna; ^8^Fondazione IRCCS Ca' Granda Ospedale Maggiore Policlinico, Obstetrics Unit, Department of Woman Child and Neonate, Milan, Italy, Milano, Lombardia; ^9^Fondazione IRCCS Ca' Granda Ospedale Maggiore Policlinico, Obstetrics Unit, Department of Woman Child and Neonate, Milan, Italy, Milan, Lombardia

12:15–13:30

**Objective**: To evaluate the correlation between umbilical vein flow (UVF) measured close to term with neonates born SGA that were not detected prenatally in a cohort of low‐risk pregnancies. We hypothesize that undetected SGA is more common in fetuses with abnormal UVF than in fetuses with normal UVF.

**Study Design**: Secondary analysis of a prospective, multicenter study conducted across two tertiary Maternity units between April 2021 and March 2023. Uncomplicated singleton pregnancies between 35‐38 weeks of gestation were included. Pregnancy at high‐risk of placental insufficiency (EFW < 10^th^ percentile or abnormal Doppler) or with fetal anomalies were excluded. At US examination, the abdominal circumference (AC), UV diameter and peak velocity of the UV were measured. From these variables, we calculated the UVF/AC. The main outcome of the study was undetected SGA, defined as a birthweight below the 10th percentile according to gestational age.

**Results**: Overall, 379 women were included in the study, of whom 16 (4.2%) were born SGA. Undetected SGA fetuses were associated with a lower median UVF/AC (4.8 (IQR 3.9–6.6) ml/min/cm vs 6.1 (IQR 4.6–7.8) ml/min/cm; *p* = 0.02). The UVF/AC yielded an AUC of 0.65 (95% CI 0.53–0.77, *p* = 0.04) for the prediction of undetected SGA. The optimal cut‐off value of the UVF/AC to predict fetal SGA was 4.8 ml/min/cm. This value was associated with a sensitivity of 0.56 (95% CI 0.30–0.80) and a specificity of 0.72 (95% CI 0.67–0.76), and a positive and negative likelihood ratio of 1.98 (95% CI 1.25–3.15) and 0.61 (95% CI 0.35–1.07); respectively. Moreover, while 3/5 (60.0%) cases of undetected SGA fetuses with an abnormal UVF/AC (below 20^th^percentile) suffered from stunted fetal growth, this occurred only in 2/9 (18.2%) with normal UVF/AC (Figure 1).

**Conclusion**: Our results suggest that undetected SGA is associated with lower values of UVF/AC in a low‐risk population. Moreover, if UVF/AC falls below the 20^th^ percentile, fetuses are more likely to present concomitantly abnormal fetal growth patterns.

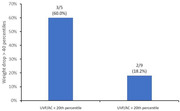

## 53 | Correlation between angiogenetic biomarkers, urinary protein values and ascites in women with HDP

Beatrice Valentini^1^, Elvira Di Pasquo^2^, Andrea Dall'asta^3^, Stefania Fieni^2^, Mariagrazia Capurso^4^, Piernicola D'Amario^4^, Valentina Anna Degennaro^2^, Alessia Casciaro^5^, Tullio Ghi^6^

^1^University of Parma, University of Parma, Emilia‐Romagna; ^2^Azienda Ospedaliero‐Universitaria di Parma, Parma, Emilia‐Romagna; ^3^Department of Medicine and Surgery, Obstetrics and Gynecology Unit, University of Parma, Department of Medicine and Surgery, Obstetrics and Gynecology Unit, University of Parma, Emilia‐Romagna; ^4^University of Parma, Parma, Emilia‐Romagna; ^5^Università di Parma, Università di Parma, Emilia‐Romagna; ^6^Dipartimento di Medicina e Chirurgia, Università di Parma, Parma, Emilia‐Romagna

12:15–13:30

**Objective**: To investigate the correlation between SFLT‐1/PlGF ratio, the 24h urinary protein and the presence of maternal ascites in a population of women with new‐onset Hypertensive Disorders of Pregnancy (HPD).

**Study Design**: Retrospective study including a cohort of women with new‐onset HDP carrying a singleton viable pregnancy between 22 and 36 gestational weeks. At diagnosis, SFLT‐1 and PlGF were assessed using Brahams Kryptor and the ratio was calculated. The amount of proteins on the 24h urine was also investigated and a value ≥5g/24h was used to define massive proteinuria. The presence of ascites was defined in if abdominal free fluid was detected at ultrasound examination.

**Results**: A total of 80 patients were included for the study purpose. A linear correlation was found between the SFLT‐1/PlGF ratio and the 24h urinary‐protein values (R2 = 0.21; *p* < 0.001). A massive proteinuria was detected in 17 women; Sflt‐1/PlGf ratio had an AUC of 0.86 for predicting the presence of massive proteinuria with a cut‐off value of 310.9 (sensitivity 88.2%, specificity 71.0%). Ascites was detected in 6 women; significantly higher values of SFLT‐1/PlGF ratios were found in this group of women compared with those without ascites (1322.0 ± 1083.0 vs. 423.0 ± 734.0).

**Conclusion**: Higher values of SFLT‐1/PLGF ratios are associated with higher levels of urinary protein and higher incidence of maternal ascitis.

## 54 | Machine learning model to predict cesarean surgical site infections

Megan M. Smith^1^, Stephen M. Wagner^2^, Enid Rivera‐Chiauzzi^3^, Michelle Wyatt^4^, Lillian Dyre^5^, Maureen Hamel^6^, Methodius G. Tuuli^7^, Sam Lobel^8^

^1^Brown Univeristy/Women & Infants Hospital of RI, Providence, RI; ^2^Beth Israel Deaconess Medical Center, Boston, Massachusetts; ^3^Mayo Clinic, Rochester, Minnesota; ^4^Essentia Health, Fargo, North Dakota; ^5^Mayo Clinic College of Medicine and Science, Rochester, Minnesota; ^6^Brown University/Women & Infants Hospital of RI, Providence, Rhode Island; ^7^Women & Infants Hospital of Rhode Island and Alpert Medical School of Brown University, Providence, Rhode Island; ^8^Brown University, Providence, Rhode Island

12:15–13:30

**Objective**: To develop a comprehensive machine learning model to identify patients at high risk of cesarean surgical site infections (cSSIs).

**Study Design**: This is a retrospective cohort study based on datasets from two independent level IV academic medical centers to improve generalizability. The first study includes 2,336 cesarean deliveries for analysis with 109 instances of cSSI (4.7%). The second study includes 2,936 cesarean deliveries and 72 instances of cSSI (2.5%). From the first study, we estimated the risk of cSSI by creating a Random Forest Machine Learning algorithm that uses 13 shared features present in both datasets. This first study cohort was utilized for both model training and internal validation, using Bayesian hyperparameter optimization for model selection, with out‐of‐bag area under the ROC curve (AUC) as the validation metric. Feature importance within the model was analyzed using permutation importance. The second study cohort was then used to evaluate testing accuracy (external validation) of this model.

**Results**: The trained model achieved an AUC of 0.77 on the training data and a 0.71 out‐of‐bag AUC. When tested on data from the second study cohort, it achieved an AUC of 0.68. The most predictive features for cSSIs, from feature importance analysis, were: maternal BMI at time of delivery, maternal age, total length of hospital stay, and gestational age at birth. On the testing dataset, the model shows a 7.5% positive predictive value of cSSI among women in the greater‐than‐90th centile risk group, and a 1.5% positive predictive value among women in the less‐than‐50th centile risk group, compared with 2.5% for the study population at large.

**Conclusion**: This machine learning model suggests risk factors for cSSIs including maternal BMI at time of delivery, maternal age, total length of hospital stay, and gestational age at birth, and provides reasonable risk stratifications for patients. This tool may be further refined and used for determining subgroups at higher risk of cSSI and identifying those who may require closer surveillance or clinical intervention.

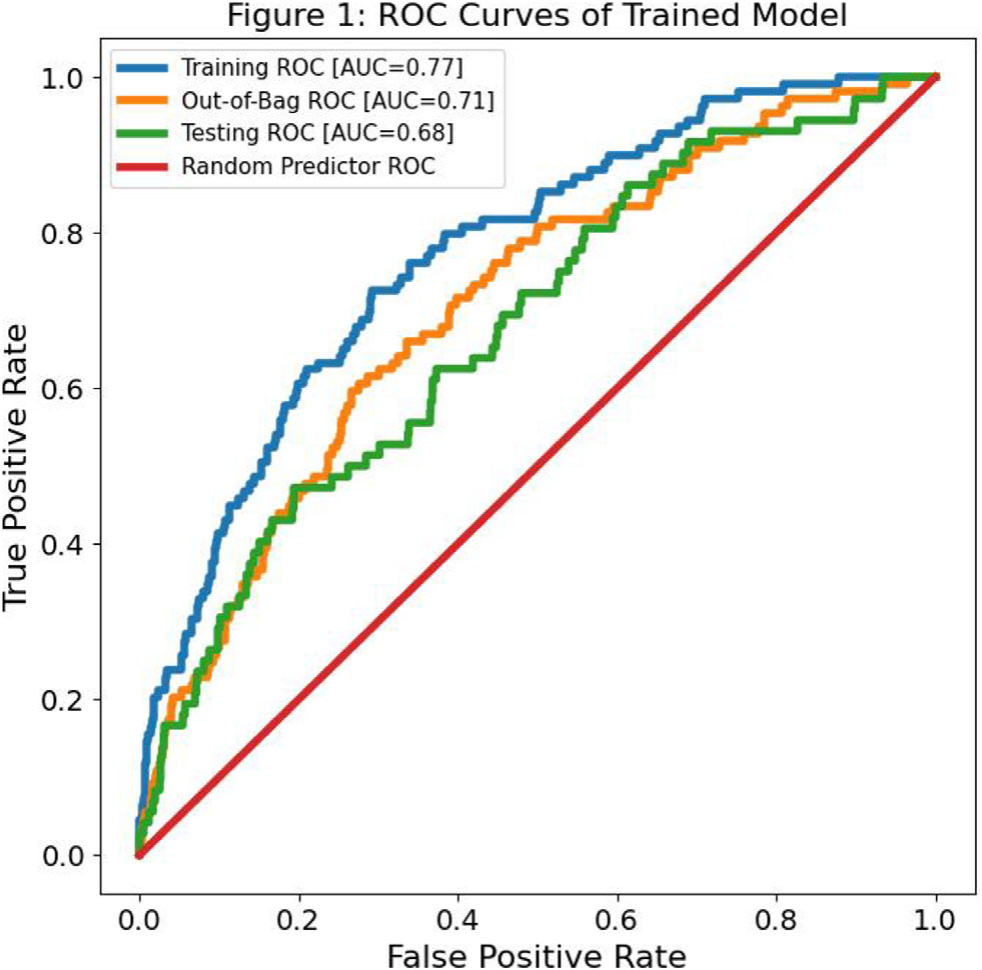

## 55 | Magnesium transporter genes: A potential marker for pregnancy‐related hypertensive disorders

Li Ching Chen^1^

^1^Cathay General Hospital, Taipei, Taipei

12:15–13:30

**Objective**: Emerging evidence suggests that magnesium (Mg) deficiency during the late first trimester may lead to impaired placentation and preeclampsia. While plasma Mg concentration is commonly used to determine Mg status, it may not fully reflect total body Mg, potentially masking deficiencies. Magnesium transporter genes (MgTGs), which regulate Mg metabolism, offer promise as potential markers of Mg status. This study aims to explore the correlation between MgTG expression in the first trimester and subsequent pregnancy‐related hypertensive disorders.

**Study Design**: A prospective cohort study was conducted in Cathay General Hospital, Taipei, Taiwan. Patients between 10 and 13 gestational weeks were recruited, excluding those with multiple pregnancies, type 2 diabetes mellitus, chronic hypertension, or prior complications such as gestational diabetes mellitus or gestational hypertension. Expression levels of five MgTGs (SLC41A1, CNNM2, MAGT1, TRPM6, TRPM7) were determined using quantitative real‐time PCR from maternal peripheral blood. Total plasma calcium, magnesium, and calcium to magnesium ratio were also analyzed.

**Results**: A total of 252 pregnant women were included in the study. Pregnancy‐related hypertensive disorders were classified as borderline pregnancy‐induced hypertension (PIH) or pregnancy‐associated hypertension (PAH). The incidence of hypertensive disorders was 3.97%, with PIH at 1.19% and preeclampsia at 2.78%. Blood pressure at delivery showed a negative correlation with MagT1, TRPM6, and TRPM7 expression levels. Maternal age, serum calcium, magnesium concentrations, and calcium to magnesium ratio did not correlate with MgTG gene expressions. Compared to the normal group, MgTG expression levels were significantly lower in both borderline PIH and PAH groups, particularly MagT1, TRPM6, and TRPM7.

**Conclusion**: Maternal peripheral blood MgTG expression in the late first trimester correlates with pregnancy‐related hypertensive disorders. This highlights the potential utility of MgTGs as markers for predicting and managing hypertensive complications during pregnancy.

## 56 | Cervical stiffness is a better predictor of preterm birth than cervical length in symptomatic women

Dario Colacurci^1^, Gabriele Saccone^2^, Giovanni Nazzaro^1^, Marina Tesorone^3^, Marilena Miranda^1^, Mariavittoria Locci^1^

^1^Università degli studi di Napoli Federico II, Napoli, Campania; ^2^University of Naples Federico II, Naples, Campania; ^3^ASL Napoli Centro 1, Napoli, Campania

12:15–13:30

**Objective**: One of the major challenges in the management of women presenting with symptoms of preterm labor (PTL) is to distinguish between true and false PTL. Over 50% of symptomatic women do not deliver prematurely, and < 10% of women give birth within 7 days of presentation.

This study aimed to evaluate if the cervical stiffness measured with the aspiration‐based device Pregnolia System can differentiate between women with true and those with false PTL.

**Study Design**: Prospective, post‐market single center observational pilot study of 100 symptomatic singleton pregnancies with symptoms of PTL (clinicaltrials.gov: NCT05355649). Cervical Stiffness Index (CSI, in mbar) with the Pregnolia System and cervical length (CL) were measured.

**Results**: Between April 2022 and January 2024, 100 women with signs and symptoms of PTL were recruited. Of these, 35 delivered prematurely (35%). Gestational age (GA) at delivery was 38+5 (37+6,39+2) (median (interquartile range)) for the women delivering at term and 35+4 (34+0,36+1) for the women delivering prematurely. 5 women delivered within 14 days from measurement (5%), of which 1 delivered within 7 days (1%). The median CSI of the term group is 88 mbar (70,102), whereas the CSI for the PTB group is 50 mbar (36,64) (*p* < 0.001). The CL in women delivering at term was 29 mm (24,31), whereas women with a PTB had a CL of 22 mm (18.5,24) (*p* < 0.001).

The Area under the Curve (AUC) for prediction of PTB < 37 weeks are 0.845 (95% CI: 0.763‐0.926) for CSI and 0.779 (0.680‐0.877) for CL; AUC for prediction of PTB < 34 weeks is 0.873 (0.729‐1.000) for CSI and 0.836 (0.563‐1.000) for CL. A model including CSI and CL did not improve the AUC of CSI alone.

AUC for prediction of delivery within 14 days are 0.979 (0.952‐1.000) for CSI and 0.744 (0.369‐1.000) for CL. A CSI threshold of 31 mbar would provide a sensitivity of 100% and a specificity of 95.8% for predicting birth within 14 days.

**Conclusion**: Women presenting with symptoms of PTL and ultimately delivering prematurely have shown a significantly softer cervix than women delivering at term. In our study, CSI was a better predictor than CL.

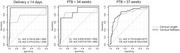

## 57 | Does vaginal progesterone prevent preterm birth in patients with a short cervix after 24 weeks?

Omri Dominsky^1^, Matan Anteby^2^, Roza Berkovitz‐Shperling^3^, Yariv Yogev^4^, Liran Hiersch^5^, Eli Rimon^6^

^1^Tel Aviv Sourasky Medical Center, Tel Aviv Sourasky Medical Center, HaMerkaz; ^2^Lis Maternity Hospital, Sourasky Medical Center, Tel Aviv University, Israel, Tel Aviv, Tel Aviv; ^3^Tel Aviv Sourasky Medical Center, Tel Aviv Sourasky Medical Center, Tel Aviv; ^4^Lis Maternity Hospital, Sourasky Medical Center, Tel Aviv University, Tel Aviv Sourasky Medical Center, Tel Aviv; ^5^Lis Maternity Hospital, Sourasky Medical Center, Tel Aviv University, Israel, Lis Hospital for Women's Health, Tel Aviv Sourasky Medical Center, Tel Aviv; ^6^Lis Hospital for Women's Health, Tel Aviv Sourasky Medical Center, tel aviv, HaMerkaz

12:15–13:30

**Objective**: To determine the efficacy of vaginal progesterone (VP) treatment in the prevention of spontaneous preterm birth (sPTB) among low‐risk patients who were diagnosed with cervical length (CL) < 25 mm after 24^0/7^ weeks of gestation.

**Study Design**: A retrospective cohort study (2011‐2022) including all patients with a singleton pregnancy and no history of sPTB who were diagnosed with a short cervix < 25 mm for the first time at 24^0/7^‐33^6/7^ weeks of gestation. The patients in the study group (490 patients) received VP while the control group (400 patients) did not. The initiation of progesterone treatment was based on physician's preference. Patients with previous sPTB, progesterone treatment prior to diagnosis of short cervix or induction of labor were excluded. The primary outcomes were sPTB < 37, < 34, < 32 and < 28 weeks of gestation.

**Results**: Patients in the study group were diagnosed earlier (29.5 ± 2.8 vs. 31.4 ± 2.4 weeks, *p* < 0.001) and had a shorter CL at diagnosis (17.5 ± 5.3 vs. 19.8 ± 5 mm, *p* < 0.001). In a logistic regression, after adjustment for gestational age (GA) and CL at diagnosis, no significant differences were found in the rates of sPTB < 37, 34, 32 and 28 weeks of gestation. Considering the differences between the groups we performed a 1:1 matching according to CL (± 1 mm) and GA at diagnosis (± 0.5 week), that resulted in 256 pairs. No significant difference was found in the rates of PTB < 37, < 34, < 32 and < 28 weeks as well as in the interval from diagnosis to delivery (49.5 ± 24.6 vs. 48.6 ± 24, days, *p* = 0.574) (Figure). Moreover, a sub‐analysis, comparing the rate of sPTB ( < 37, < 34, < 32, < 28 weeks) according to CL at diagnosis ( < 10 mm, < 15mm, 10‐20mm) and GA at diagnosis (24‐28, 28‐30, 30‐32 and 32‐34 weeks) revealed no significant differences in the rates of sPTB regardless of initial CL and GA at diagnosis.

**Conclusion**: In patients with singleton pregnancy and no history of PTB who were diagnosed with a short cervix at 24^0/7^‐33^6/7^ weeks of GA, vaginal progesterone was not associated with reduction in the rate of sPTB or prolongation of pregnancy.

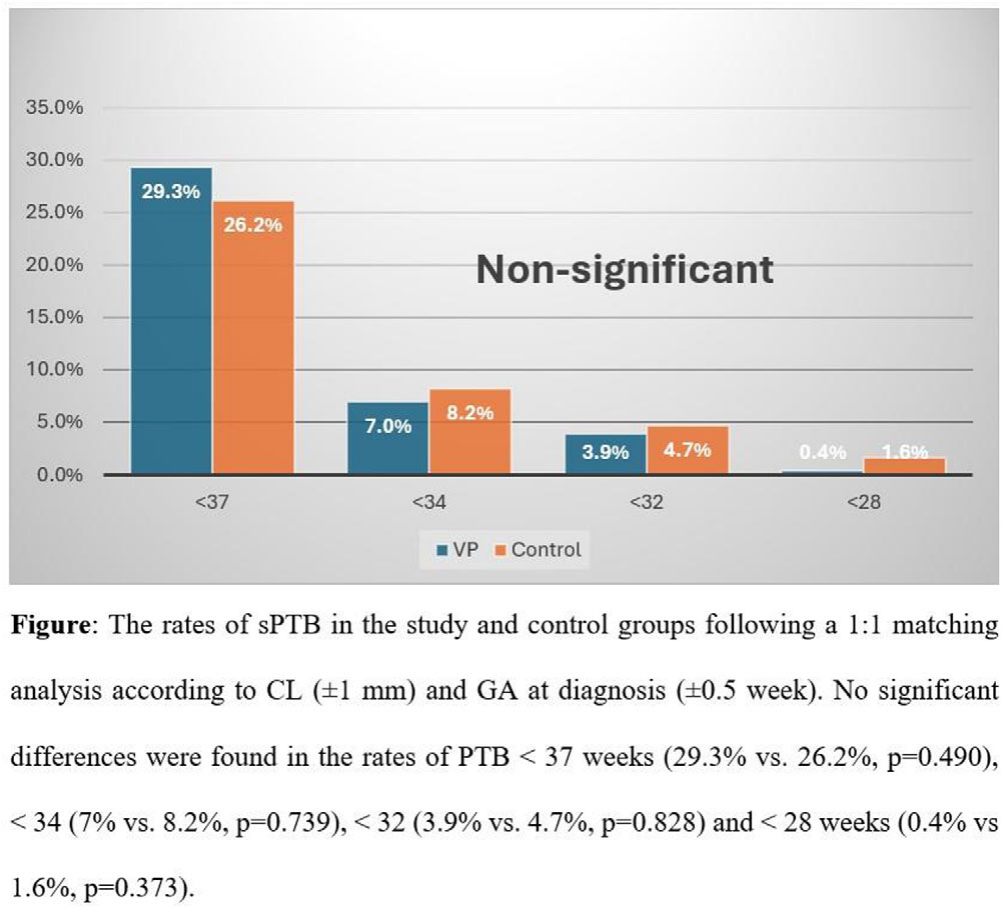

## 58 | Risk factors for cesarean bladder injury: Insights from a retrospective decade‐long tertiary center study

Sara Doroldi^1^, Marina Valeriani^2^, Jacopo Lenzi^3^, Laura Larcher^2^, Elena Contro^2^

^1^Obstetric Unit, IRCCS Azienda Ospedaliero‐Universitaria di Bologna, Bologna, Emilia‐Romagna; ^2^Obstetric Unit, IRCCS Azienda Ospedaliero‐Universitaria di Bologna, Obstetric Unit, IRCCS Azienda Ospedaliero‐Universitaria di Bologna, Emilia‐Romagna; ^3^Department of Biomedical and Neuromotor Sciences, University of Bologna, Department of Biomedical and Neuromotor Sciences, University of Bologna, Emilia‐Romagna

12:15–13:30

**Objective**: Cesarean section (CS) is one of the main causes of iatrogenic bladder injury. This study aimed to identify risk factors and critical surgical steps for this complication.

**Study Design**: An observational case‐control study was conducted at our tertiary center. Patients diagnosed with full‐thickness bladder damage during CS between 2009 and 2021 were included, with each case matched to four controls.

**Results**: Out of 13,710 CSs performed, 25 cases of bladder injury were identified (incidence of 0.2%). In primary CSs, bladder lesions were most frequently observed post‐fetal extraction (4/11 cases) and during peritoneal cavity opening (3/11 cases). In iterative CSs, 50% of bladder lesions occurred during bladder flap creation (7/14 cases).

When compared to a cohort of 100 patients without this complication, the number of previous CSs correlated with bladder injuries (*p* = 0.01), while no differences were observed in BMI, history of abdominal surgery, or endometriosis. Logistic regression identified the number of prior CSs as the sole significant risk factor (OR = 2.26, 95% CI 1.18–4.33, *p* = 0.01).

CSs complicated by bladder damage were associated with intraoperative complications, especially adhesions (13/25 vs. 2/100, *p* < 0.001) and Placenta Accreta Spectrum disorders (2/25 vs. 0/100, *p* = 0.04), as well as postoperative complications, including postpartum hemorrhage (8/25 vs. 5/100, *p* = 0.001), Intensive Care Admission (6/25 vs. 0/100, *p* < 0.001), and infections (3/25 vs. 1/100, *p* = 0.03). Neonatal pH levels were significantly lower in cases of bladder lesions (mean pH level 7,23 vs. 7,33, *p* = 0.002).

**Conclusion**: Bladder injury during CS is rare but can lead to noteworthy intra and postoperative complications. The number of previous CS is a significant risk factor. Bladder flap creation represents a delicate surgical step, meticulous tissue separation is crucial. Peritoneal entry should also be approached with caution, particularly in primary CSs. Awareness of potential surgical complications in repeat CSs is essential when counseling patients for primary cesarean delivery.

## 59 | The impact of ACOG's 39‐week rule on fetal death rates in america: A systematic review

Michael T. Finlan^1^, Alisha Goyal^2^, Yingting Zhang^3^, Vincenzo Berghella^4^, Justin S. Brandt^5^

^1^Rutgers Robert Wood Johnson Medical School, Asbury Park, New Jersey; ^2^Sidney Kimmel Medical College at Thomas Jefferson University, Sidney Kimmel Medical College at Thomas Jefferson University, Pennsylvania; ^3^Rutgers Biomedical and Health Sciences, Piscataway, New Jersey; ^4^Sidney Kimmel Medical College of Thomas Jefferson University, Philadelphia, Pennsylvania; ^5^NYU Langone Health, New York, New York

12:15–13:30

**Objective**: The “39‐week rule,” implemented in August 2009, strongly discouraged early term deliveries before 39 weeks without accepted ACOG indications. In this study, we evaluated fetal death rates before and after the 39‐week rule in the United States (US) by review of published series.

**Study Design**: Systematic literature searches were performed in PubMed, Cochrane Central Register of Controlled Trials, Embase, Cumulative Index to Nursing and Allied Health Literature, Web of Science, and Scopus databases (January 2009‐June 2023). Searches were focused on the 39‐week rule and fetal death. In each concept, we identified keywords, subject headings, synonyms, and other related terms to exhaust search results in accordance with PRESS guidelines. We included observational studies that examined fetal death risk pre‐ and post‐implementation of the 39‐week rule. Articles were excluded if they were non‐English, included non‐US population, and included multiple gestations. Two blinded authors reviewed the titles (and the abstract, when necessary). The study was prospectively registered in PROSPERO.

**Results**: Of 833 articles identified after initial search, 6 peer‐reviewed studies met the inclusion criteria. There were 8,726 fetal deaths/7,267,648 total births (0.12%) post‐implementation of the 39‐week rule, and 8,523 fetal deaths/7,622,439 total births (0.11%) pre‐implementation (*p* < 0.001) (TABLE). Compared to pre‐implementation, the odds of fetal death after the 39‐week rule were 1.07 (95% confidence interval 1.04‐1.11).

**Conclusion**: Implementation of ACOG's 39‐week rule, currently used as a quality indicator, resulted in an 7% increased risk of fetal death, compared to pre‐implementation of the 39‐week rule. Further study is needed to identify other unintended consequences, such as the impact on maternal and neonatal morbidity and mortality, associated with adoption of this policy.

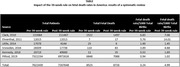

## 60 | Relationship between maternal age and adverse birth outcomes among underserved minority populations in southeastern texas

Irene A. Stafford^1^, Krystal L. Garcia^2^

^1^McGovern Medical School at UTHealth Houston, Houston, Texas; ^2^McGovern Medical School, Dallas, TX

12:15–13:30

**Objective**: Prenatal care is recommended during pregnancy as a method to improve neonatal and maternal outcomes. Prior studies have demonstrated that women of advanced maternal age disproportionately experience adverse perinatal outcomes. Our aim is to determine if there are age‐specific risks that lead to adverse birth outcomes in neonates in Houston, Texas.

**Study Design**: A retrospective cohort study between January 2019 and December 2022 was performed for all pregnant individuals who sought care and delivered in Houston. The association between maternal age and adverse birth outcomes was calculated. A Poisson regression model with robust error variance was used to examine the association between age and adverse birth outcomes while adjusting for potential confounders. Adjusted odds ratios (OR) with 95% confidence intervals were calculated.

**Results**: During the study period, 20,076 patients (mean [SD] age, 28.675 [6.6] years) sought care and delivered at the site, of which 100% had analyzable data. Women 17 years of age or younger demonstrated an increased risk of having a small for gestational age (SGA) newborn (OR = 1.987; 95% CI, 1.512‐2.612). Composite adverse birth outcomes were significantly associated with age. Patients 17 years and younger, and 40 years old and greater had the highest risk for all adverse outcomes (OR = 1.210; 95% CI, 1.004‐1.457, and OR = 1.660; 95% CI, 1.443‐1.910 respectively.

**Conclusion**: Extremes of maternal age were associated with the highest risk of adverse perinatal outcomes in this cohort of underserved minority patients, with adolescents demonstrating the highest risks of neonatal complications. The cause of this increased risk in neonatal complications in adolescence remains unclear and should be a focus for future research. Identification of the key risk factors in adolescents that lead increased neonatal complications can help facilitate the development of interventions that can improve outcomes in neonates and mothers.

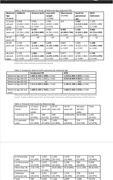

## 61 | Follow up of children born to mothers in Folic Acid Clinical Trial (FACT 4 Child)

Maryam Ghiasi^1^, Shi‐Wu Wen^2^, Ruth Rennicks White^2^, Alysha Dingwall‐Harvey^3^, Kimberly P. Grattan^1^, Marisa Murray^2^, Natalie Rybak^4^, Heather A. Walker^1^, Laura Gaudet^2^, Stephen Robson^5^, William Hague^6^, Donnette Simms‐Stewart^7^, Graeme Smith^8^, Daniel J Corsi^2^, Gary Goldfield^9^, Darine El‐Chaâr^10^, Mark Walker^2^

^1^Ottawa Hospital Research Institute, Ottawa, ON; ^2^Ottawa Hospital Research Institute, Ottawa, Ontario; ^3^Better Outcomes Registry & Network (BORN) Ontario, Ottawa, Ontario; ^4^The Ottawa Hospital, Ottawa, Ontario; ^5^Institute of Cellular Medicine, Newcastle University, Tyne and Wear, England; ^6^Women's and Children's Health, Adelaide, South Australia; ^7^University of the West Indies, Mona, Kingston; ^8^Queen's Perinatal Research Unit, Kingston General Hospital, Department of Obstetrics and Gynecology, Queens University, Kingston, Ontario; ^9^Children's Hospital of Eastern Ontario, Ottawa, Ontario; ^10^The Ottawa Hospital, Ottawa, ON

12:15–13:30

**Objective**: FACT 4 Child is a follow up study of mothers who participated in Folic Acid Clinical Trial (FACT) and their children between 4‐9 years of age. The objective is to assess the impact of high‐dose folic acid supplementation (4.0‐5.1 mg), started between the eighth and sixteenth completed weeks of gestation and continued until delivery, on social impairments associated with Autism Spectrum Disorders (ASDs), deficiencies in executive function, emotional and behavioral problems in young children, and mortality or severe morbidity.

**Study Design**: ***FACT 4 Child*** is an international prospective multi‐centre cohort follow up of mothers and their children born while FACT participants. Mothers who provided informed consented to FACT 4 Child for participation provided self‐reported assessments of social and executive function, and emotional and behavioural problems in their children according to standardized validated questionnaires.

**Results**: From 2578 children born in the FACT trial, 664 participated in FACT 4 Child and completed questionnaires. Among 319 in the high‐dose folic acid group, 43 (13.5%) had abnormal results, while among 345 in the placebo group, 51 (14.8%) had abnormal results.

**Conclusion**: Despite efforts made to enroll all FACT sites and participants, the completed questionnaires did not reach the minimum required to achieve >80% power to detect a 30% relative difference in the proportion of abnormal scores. The challenges of recruiting during the Covid‐19 pandemic and subsequent difficulties with follow‐up contributed to this issue. The results of our study were unable to show evidence that supplementation with high‐dose folic acid will increase the risk of social impairments associated with ASDs, yet may suggest a potential inverse relationship. Further research with larger sample sizes is warranted to confirm these findings and explore potential mechanisms.

## 62 | The role of phospholipid transfer protein in changing high‐density lipoprotein structure and function during pregnancy

Milica M. Miljkovic Trailovic^1^, Tamara Gojkovic^1^, Jasmina Ivanisevic^1^, Jelena Munjas^1^, Daniela Ardalic^1^, Marko Stankovic^1^, Snezana Jovicic^1^, Marija Saric Matutinovic^1^, Aleksandra Zeljkovic^1^, Jelena Vekic^1^, Zeljko Mikovic^1^, Vesna Spasojevic‐Kalimanovska^1^, Aleksandra Stefanovic^1^

^1^Faculty of Pharmacy, University of Belgrade, Belgrade, Vojvodina

12:15–13:30

**Objective**: Phospholipid transfer protein (PLTP) plays a key role in lipoprotein metabolism during pregnancy, especially in the reverse cholesterol transport pathway. It removes excess cholesterol from the surface of triglyceride‐rich lipoprotein particles and transfers it to high‐density lipoproteins (HDL). In this way, there is a change in the size and composition of HDL particles, and thus a change in the function of circulating HDL fractions.

The objective of this work was to examine changes in the concentration and gene expression of PLTP during pregnancy, their relationship with the distribution of HDL subfractions, as well as changes in the concentrations of HDL and apolipoprotein A1 (Apo‐A1).

**Study Design**: The study involved 71 pregnant women who were following during the first, second and third trimester at the Clinic for Gynecology and Obstetrics. Concentrations of lipid status were determined using commercial biochemical tests, while the concentration of PLTP was measured using the ELISA test. Assessment of gene expression of the PLTP gene was obtained using the High Capacity DNA Reverse Transcription Kit.

**Results**: The concentration of PLTP, HDL, Apo‐A1, and the gene expression level of PLTP are statistically significantly higher at the beginning of pregnancy (*p* < 0.05). The correlation analysis showed a statistically significant negative correlation of PLTP concentrations with HDL diameter (ρ = −0.334, *p* < 0.05) and the distribution of HDL 2b (ρ = −0.426, *p* < 0.05), while it showed a statistically significant positive correlation with the distribution of HDL 3a (ρ = 0.0.283, *p* < 0.05), HDL 3b (ρ = 0.418, *p* < 0.05), and HDL 3c (ρ = 0.334, *p* < 0.05) in the first trimester. The relative level of PLTP gene expression in the third trimester had a statistically significant negative correlation with the distribution of HDL3 (ρ = −0.311, *p* < 0.05).

**Conclusion**: The results of our research showed that the increase in PLTP concentration at the beginning of pregnancy plays a key role in the formation of small atheroprotective HDL 3a particles.

## 63 | Post‐partum sonographic measurement of the Inferior Vena Cava (IVC) and correlation with hemoglobin levels

Elvira Di Pasquo^1^, Alessandra Familiari^2^, Beatrice Valentini^3^, Giulia di Marco^4^, Chiara Alfarè^5^, Francesca Felici^6^, Piernicola D'Amario^7^, Antonio Lanzone^8^, Tullio Ghi^9^

^1^Azienda Ospedaliero‐Universitaria di Parma, Parma, Emilia‐Romagna; ^2^University of the Sacred Heart Department of Women and Child Health, Fondazione Policlinico Universitario Agostino Gemelli IRCCS, Università Cattolica del Sacro Cuore, Roma, Lazio; ^3^University of Parma, University of Parma, Emilia‐Romagna; ^4^Policlinico A. Gemelli Roma, Policlinico A. Gemelli Roma, Lazio; ^5^Azienda Ospedaliero‐Universitaria di Parma, Italy, Alabama; ^6^Fondazione Policlinico Universitario Agostino Gemelli IRCCS, Rome, Italy, Italy, Emilia‐Romagna; ^7^University of Parma, Parma, Emilia‐Romagna; ^8^Fondazione Policlinico Agostino Gemelli, IRCSS, Rome Italy, Rome, Lazio; ^9^Dipartimento di Medicina e Chirurgia, Università di Parma, Parma, Emilia‐Romagna

12:15–13:30

**Objective**: The Inferior Vein Cava (IVC) diameter reflects the pressure in the right atrium, which is a measure of cardiac preload; it is sensitive to blood volume and its diameter varies accordingly. The aim of our study was to evaluate the correlation between the IVC diameter, Collapsibility Index (CI) and the Hemoglobin drop 24h after the delivery.

**Study Design**: Prospective cohort study conducted in two tertiary maternity centers. The study cohort consisted of women with singleton pregnancy submitted to an elective Caesarean Delivery at term. IVC diameters were measured by means of abdominal ultrasonography during inspiration (IVCi) and expiration (IVCe) and the CI was calculated as (IVCe‐IVCi)/IVCe two hours after the delivery according to a standardized technique (Figure 1). The total blood loss (BL) was quantified using the quantitative measurement and the total amount of intravenous fluid administrated was recorded. Hemoglobin (Hb) was determined 24 hours after the Caesarean Section (Hbt1) and the Hb drop was calculated as the difference between the Hbt1 value and the pre‐operatory value (HbT0). The primary outcome of the study was to assess the correlation between the CI and a Hb drop ≥ 2g.

**Results**: Overall 76 women were included for the study purpose with a mean maternal age of 35.8 ± 5.3 years and a mean BMI of 23.7 ± 5.5. Mean estimated BL was 422 ± 323 mL and mean CI 2 hours after delivery was 28.2 ± 20.0%. 16 (21.0%) women had a Hb drop ≥ 2g.

After adjusting for the total amount of intravenous fluids administered and BMI no correlation between the CI and a Hb drop ≥ 2g was observed (*p* = 0.50) nor between the IVC diameter and a Hb drop ≥ 2g (*p* = 0.25).

**Conclusion**: IVC diameter and CI assessed 2 hours after the delivery seem to be not associated with the occurrence of an Hb drop ≥ 2g.

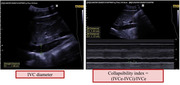

## 64 | The TIMING‐study: Patients' and clinicians' perspectives on reproductive healthcare and Autosomal Dominant Polycystic Kidney Disease

Margriet Gosselink^1^, Robin Mooren^2^, Rozemarijn Snoek^2^, Neeltje Crombag^2^, Paul Vos^3^, Mandy Keijzer‐Veen^2^, Albertien van Eerde^2^, Titia Lely^2^

^1^UMC Utrecht, Utrecht, Utrecht; ^2^UMC Utrecht, UMC Utrecht, Utrecht; ^3^Haga Ziekenhuis, Haga Ziekenhuis, Zuid‐Holland

12:15–13:30

**Objective**: Family planning and reproductive care are essential yet complex aspects of lifecycle management for individuals with Autosomal Dominant Polycystic Kidney Disease (ADPKD), given the potential genetic transmission and pregnancy‐related complications. This qualitative study studied the experiences and perspectives of ADPKD patients and their clinicians to identify areas for potential improvement in reproductive lifecycle care.

**Study Design**: Focus group discussions were conducted in the Netherlands with ADPKD patients, both men and women, who had children through varied reproductive choices, and clinicians, including (pediatric) nephrologists, obstetric gynecologists, geneticists. Thematic analysis, utilizing a grounded theory approach, was performed on verbatim transcriptions of recordings, followed by consensus discussions to finalize themes.

**Results**: Nine focus groups involving 31 participants (16 patients and 15 physicians) identified six key themes. These included the need for timely and comprehensive information dissemination, understanding patient‐specific decision‐making factors, improving tailored psychosocial guidance and communication, the need for systematic efforts to take care of missed (minor) at‐risk patients, addressing inequities in access to care, and improving multidisciplinary collaboration.

**Conclusion**: This study represents the first qualitative study of patient and physician perspectives on reproductive lifecycle care for ADPKD. We present valuable insights into factors influencing patients’ reproductive decision‐making, a comprehensive comparison between the perspectives of patients and clinicians on family planning, and follow‐up care of minors at risk for ADPKD, and recommendations for enhancing overall care quality. Incorporating these insights into clinical care could enhance patient‐centered care and foster interdisciplinary collaborations to further improve the quality of reproductive healthcare services for individuals with ADPKD.

## 65 | Fetal cardiac deformation in persistent left‐sided superior vena cava and suspected coarctation of the aorta

Sara D. Gungor^1^, Sarah McCollum^2^, Meredith Leslie^2^, Ruchika Karnik^2^, Dina Ferdman^2^, Veronika Shabanova^2^, Katherine Kosiv^1^

^1^Yale University, New Haven, CT; ^2^Yale University, New Haven, Connecticut

12:15–13:30

**Objective**: Prenatal prediction of postnatal coarctation of the aorta remains challenging due to the lack of prognostic ultrasound markers and post‐natal circulation changes. Fetal cardiac deformation analysis, which measures changes in cardiac adaptation or strain, has been proposed and tested as a predictive tool for coarctation of the aorta. The fetal echocardiogram in isolated persistent left‐sided superior vena cava (PL‐SVC) mimics suspected coarctation of the aorta because of shared left ventricular to right ventricular size discrepancy. We sought to describe cardiac deformation in fetuses with PL‐SVC and suspected coarctation of the aorta and hypothesized that cardiac strain would be different in fetuses with suspected coarctation compared to PL‐SVC.

**Study Design**: This is a single‐center, retrospective study of fetuses with PL‐SVC and suspected coarctation by fetal echocardiography from 2014‐2023. Fetuses with other forms of congenital heart disease were excluded. Fetal cardiac deformation analysis was performed offline on a 4‐chamber clip from the last fetal echocardiogram prior to delivery using a vendor non‐specific software (TomTec Arena). The reviewer performing the analysis was blinded to the fetal diagnosis. The primary variable was global longitudinal strain (GLS) and secondary variables included left ventricular ejection fraction (LVEF), left ventricular end‐systolic volume (LVESV), and left ventricular end‐diastolic volume (LVEDV).

**Results**: Fifty‐nine subjects were included (29 with isolated PL‐SVC and 30 with suspected coarctation) in the analysis. Cardiac strain (*p* = 0.65), LVEF (*p* = 0.54), LVEDV (*p* = 0.24) and LVESV (*p* = 0.16) were similar in both PL‐SVC and suspected coarctation groups. LVESV and LVEDV was slightly higher in the suspected coarctation group. Compared to normal values, GLS, LVEF, and LVESV significant differed in PL‐SVC (*p* < 0.0001).

**Conclusion**: GLS is in fetuses with PL‐SVC and suspected coarctation of the aorta is similar. LV volume measurements may provide a meaningful difference between the groups, however further investigation is warranted.

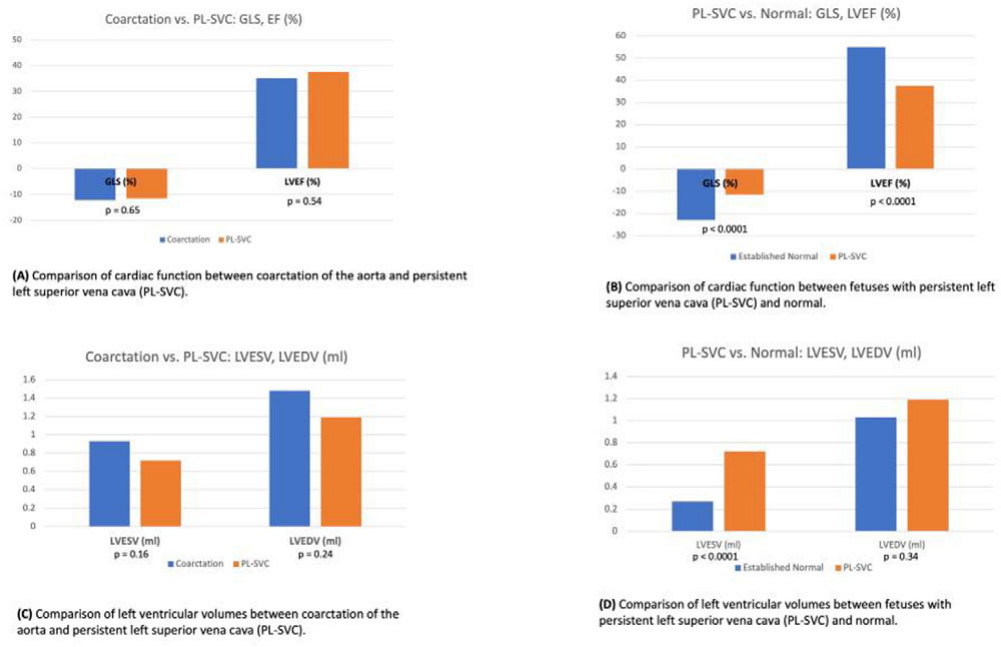

## 66 | Impact of an educational video on knowlege of vaccines during pregnancy among pregnant women

Maria Isabel Hawayek^1^, Daniel Perez^2^, Alexandra Quintana^2^, Aniris Roman^3^, Josefina Romaguera^2^

^1^University of Puerto Rico Medical Sciences Campus, San Juan, PR; ^2^University of Puerto Rico Medical Sciences Campus, University of Puerto Rico Medical Sciences Campus, Puerto Rico; ^3^University of Puerto Rico Rio Piedras, University of Puerto Rico Medical Sciences Campus, Puerto Rico

12:15–13:30

**Objective**: Vaccination during pregnancy has been reported as the first line of defense against many common diseases for pregnant women and developing fetuses. However, lower rates of vaccination have been described among pregnant women in recent years. Educational material has been shown to be an effective intervention for increasing knowledge and vaccination among pregnant women. This study aims to showcase the effect of an educational video on the knowledge of vaccination during pregnancy among pregnant women living in Puerto Rico.

**Study Design**: Surveys were conducted among the population using Survey Monkey regarding demographic data, general misconceptions, and knowledge base of pregnant women regarding vaccines during pregnancy. These were then shown an educational video debulking many common misconceptions and answers retaken to evaluate if their thoughts and perceptions had changed.

**Results**: Participants were between the ages of 21 and 44 (*n* = 200). When asked if “Vaccination against Influenza, Pertussis and COVID‐19 can reduce the rate of hospitalizations in infants?”, few participants replied “False” (7.5%) while others replied that they did not know (29%). After the educational video was presented, the group that originally answered “Don't know” decreased to only 5% of participants. Before the video, there were as many participants that answered “Don't know” to if The COVID‐19 vaccine could cause a miscarriage as those who replied false to this statement 46.5%. After being shown the video, most then replied “false” (81%) and only 4% replying “Don't know”. People were also asked if they believed vaccines could cause autism. Before the video 35% answered that they were not sure, however after the video this percentage decreased to 18.

**Conclusion**: This study shows the importance and impact educational material can have on patients. Due to increasingly available materials on social media and other platforms that are easily accessible, it is important that the information comes from reliable sources, especially in a population such as that of pregnant women, so that they may make informed decisions.

## 67 | Impact of gestational diabetes mellitus on neonatal outcomes in small for gestational age infants

Ayala Hirsch^1^, Tzuria Peled^2^, Hen Y. Y. Sela^3^, Ari Weiss^2^, Sorina Grisaru Granovsky^4^, Misgav Rottenstreich^5^

^1^Shaare zedek Medical Center, Jerusalem, Yerushalayim; ^2^Shaare Zedek Medical Center, Shaare Zedek Medical Center, Yerushalayim; ^3^Shaare Zedek, Jerusalem, Yerushalayim; ^4^Hebrew University, Jerusalem, Yerushalayim; ^5^McMaster University, Hamilton, Ontario

12:15–13:30

**Objective**: To evaluate obstetric and perinatal outcomes among small for gestational age (SGA) infants born to patients diagnosed with Gestational diabetes mellitus (GDM).

**Study Design**: A multicenter retrospective cohort study between 2005 and 2021. The perinatal outcomes of SGA infants born to patients with singleton pregnancy and GDM were compared to SGA infants born to patients without GDM. The primary outcome was a composite adverse neonatal outcome. Infants with known structural/genetic abnormalities or infections were excluded. A univariate analysis was conducted followed by a multivariate analysis (adjusted odds ratio [95% confidence interval]).

**Results**: During the study period, 11,662 patients with SGA infants met the inclusion and exclusion criteria. Of these, 417 (3.6%) SGA infants were born to patients with GDM, while 11,245 (96.4%) were born to patients without GDM. Overall, the composite adverse neonatal outcome was worse in the GDM group (53.7% vs 17.4%, *p* < 0.01). Specifically, adverse neonatal outcomes such as a 5‐minute Apgar score < 7, meconium aspiration, seizures, and hypoglycemia were independently associated with GDM among SGA infants. In addition, patients with GDM and SGA infants had higher rates of overall and spontaneous preterm birth, unplanned cesarean, and postpartum hemorrhage. In a multivariate logistic regression assessing the association between GDM and neonatal outcomes, GDM was found to be independently associated with the composite adverse neonatal outcome (aOR 4.26 [3.43‐5.3]), 5‐minute Apgar score < 7 (aOR 2 [1.16‐3.47]), meconium aspiration (aOR 4.62 [1.76‐12.13]), seizures (aOR 2.85 [1.51‐5.37]) and hypoglycemia (aOR 16.16 [12.79‐20.41]).

**Conclusion**: Our study demonstrates that GDM is an independent risk factor for adverse neonatal outcomes among SGA infants. This finding underscores the imperative for tailored monitoring and management strategies in those pregnancies.

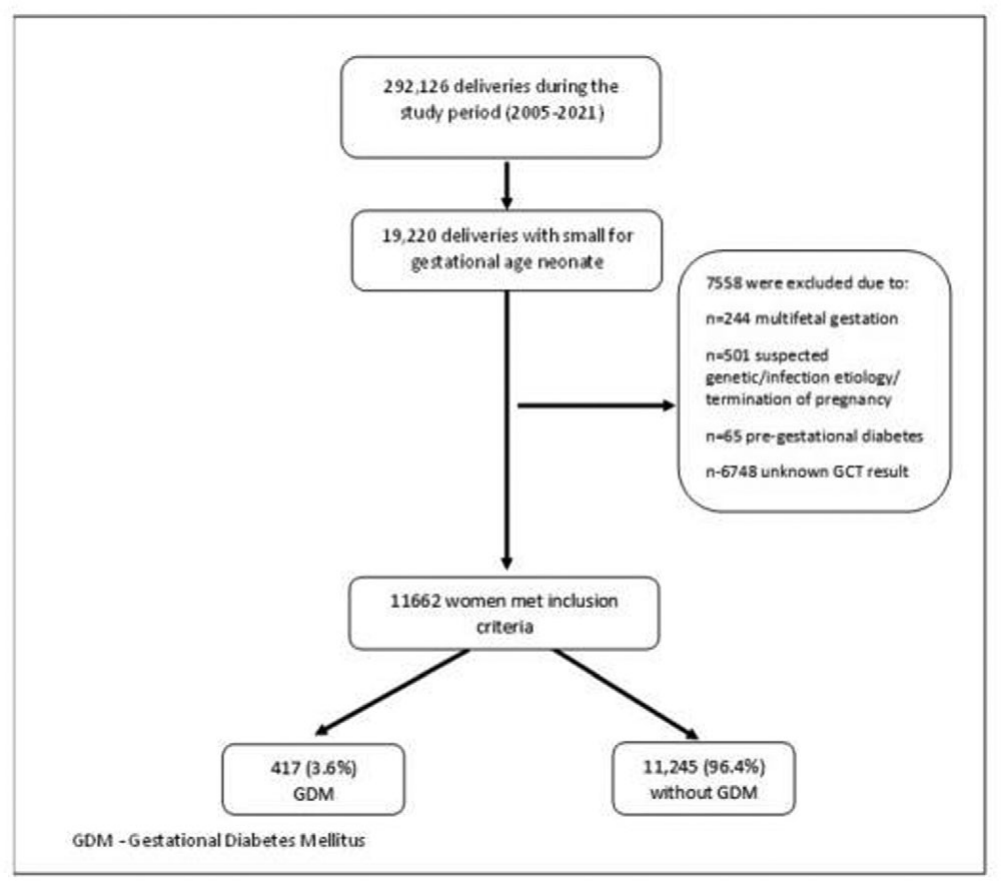

## 68 | Oral antihypertensives in hypertensive pregnancy and the association with fetal growth: A population‐based cohort study

Rosalie Hup^1^, Martijn van Ling^2^, Eline Houben^3^, Jens Bos^4^, Martine Depmann^5^, Titia Lely^2^

^1^UMC Utrecht, Amsterdam, Utrecht; ^2^UMC Utrecht, UMC Utrecht, Utrecht; ^3^PHARMO Institute for Drug Outcomes Research, PHARMO Institute for Drug Outcomes Research, Utrecht; ^4^IADB.nl, IADB.nl, Groningen; ^5^UMC Utrecht, Utrecht, Utrecht

12:15–13:30

**Objective**: Of the oral antihypertensive agents (OAA) available in pregnancy (methyldopa, labetalol, nifedipine), methyldopa alone is registered ‘safe in pregnancy’. A possible association between labetalol and fetal growth restriction (FGR) and insufficient data available on nifedipine hamper safety registration. Over 45 years of research yielded data on less than 6000 pregnancies in 58 trials could not state OAA safety. As such, we propose a novel approach. Using registration data on OAA use and pregnancy outcomes, we will assess if exposure to OAA affects fetal growth.

**Study Design**: In this retrospective cohort study, linking pregnancy registry data to pharmacy dispensing records, we included pregnancies exposed to OAA monotherapy from 2000‐2019. Risk of small for gestational age (SGA, birth weight < 10th percentile) was calculated via multivariate logistic regression, adjusted for maternal age, parity, diabetes, socio‐economic status, hypertension type and comedication. We analyzed nifedipine use in chronic hypertension (CH) alone to exclude a tocolytic indication.

**Results**: 11.033 pregnancies with dispensing data were identified. Monotherapy was prescribed in 76.0% (methyldopa 73.9%, labetalol 17.4%, nifedipine 8.7%). Mean maternal age (SD) was 32.2 (4.7) for methyldopa, 32.6 (4.7) for labetalol and 30.7 (5.4) for nifedipine. Mean GA in weeks + days and birth weight in grams (SD) was 38+0 (2+3) weeks and 3057 (736) for methyldopa, 38+3 (2+2) and 3134 (695) for labetalol and 37+3 (2+4) and 2922 (707) for nifedipine. No significant difference in risk for SGA was observed for labetalol versus methyldopa and for nifedipine versus methyldopa or labetalol in CH subgroup (Table 1).

**Conclusion**: In this cohort study, no effect of type of OAA on the occurrence of FGR was observed. Preference for methyldopa, labetalol or nifedipine should be determined on medication effectiveness and maternal side effects. Large‐scale observational studies using Electronic Patient Files with elaborate data will allow for analysis on maternal outcomes and other fetal side effects such as neonatal admission or hypoglycemia.

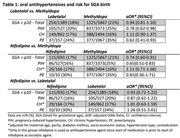

## 70 | Preeclampsia with severe features before 34 weeks: Comparing outcomes for expectant management versus immediate delivery

Josef Jackson^1^, Alison N. Goulding^2^, Felixnando Rubio^3^, Jeevitha Patil^4^, Steven Clark^2^, Mary C. Tolcher^5^

^1^Baylor College of Medicine, Houston, TX; ^2^Baylor College of Medicine, Houston, Texas; ^3^University of Colorado, Denver, Colorado; ^4^Roswell OBGYN, LLC, Roswell, Georgia; ^5^Baylor College of Medicine, Baylor College of Medicine, TX

12:15–13:30

**Objective**: To describe characteristics and clinical outcomes of patients diagnosed with preeclampsia with severe features before 34 weeks comparing those delivered immediately to those managed expectantly.

**Study Design**: Retrospective cohort study of two hospitals in our academic institution from 2015 to 2019. Patients were included if preeclampsia with severe features was diagnosed between 24 0/7 and 33 6/7 weeks’ gestation. High order multiples, rupture of membranes, labor, intrauterine fetal demise, and fetal anomalies were excluded. Baseline characteristics and outcomes were compared based on whether expectant management was pursued.

**Results**: 404 patients met inclusion criteria. 220 (54%) were expectantly managed and 184 (46%) were delivered immediately. In the expectantly managed group median gestational age at diagnosis was earlier (30 2/7 vs 31 4/7 weeks, *p* < 0.001) and fetal growth restriction was more likely (46 vs 31%, *p* = 0.002). Severe hypertension was the defining “severe” feature in 93% of all patients and was more common in the expectantly managed group (95 vs 90%, *p* = 0.03). Headache (22 vs 33%, *p* = 0.01), vision changes (5 vs 13%, *p* = 0.004), right upper quadrant pain (5 vs 13%, *p* = 0.004), and transaminitis (8 vs 24%, *p* < 0.001) were all more likely in the immediate delivery group. Despite a median latency of 3 days, gestational age at delivery was lower in the expectantly managed group (31.0 vs 32.0 weeks *p* = 0.03). Neonatal outcomes differed only by slightly lower birthweight in the expectantly managed group (1402 vs 1515 grams, *p* = 0.02).

**Conclusion**: Patients with symptoms or transaminitis were more likely to be delivered immediately. Lower median gestational age at delivery and correspondingly lower neonatal birth weight in expectantly managed patients likely represents a lower threshold for delivery at more advanced gestational ages. Delivery planning based on traditional classifications of disease severity does not appear to yield improved outcomes in patients managed conservatively, suggesting that such classifications are poorly predictive of true disease severity or impact on maternal‐fetal health.

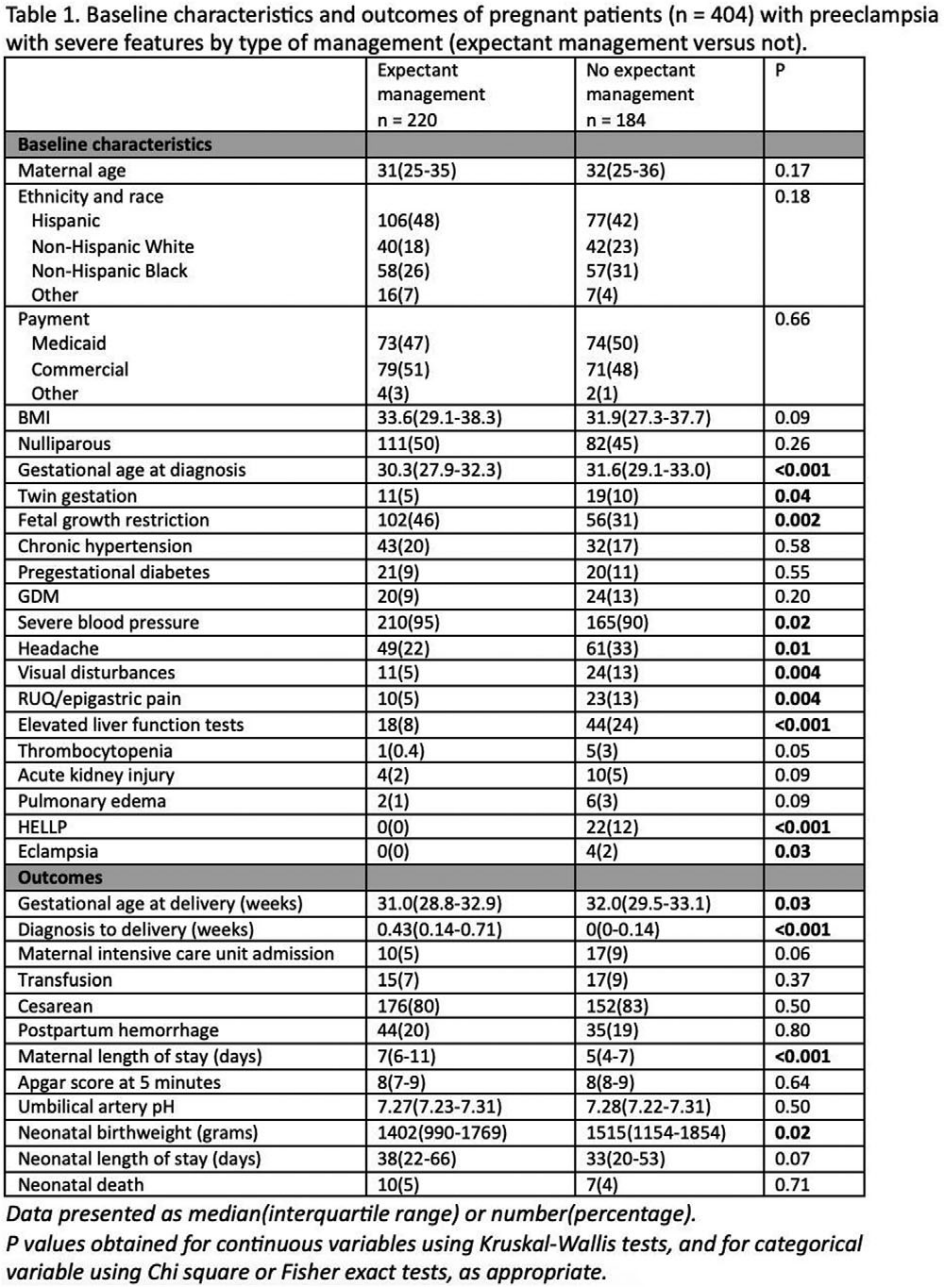

## 72 | A cesarean birth during labor increases the risk of placenta accreta in a subsequent pregnancy

Lior Kashani Ligumsky^1^, Miriam Lopian^2^, Ariel Many^3^, Avshalom Elmalech^4^, Deborah Krakow^1^, Yalda Afshar^1^

^1^University of California, Los Angeles, Los Angeles, California; ^2^Mayanei Hayeshua Medical Center, Mayanei Hayeshua Medical Center, Tel Aviv; ^3^Department of Obstetrics and Gynecology, Mayanei Hayeshua Medical Center, bnei brak, HaMerkaz; ^4^Bar Ilan University, Givat Shmuel, Israel, Givat Shmuel, HaMerkaz

12:15–13:30

**Objective**: Placenta accreta spectrum (PAS) disorders are associated with significant morbidity. Cesarean delivery (CD) is a clinical risk factor for PAS. The lower uterine segment milieu and hysterotomy of a labored CD versus a planned non‐labored CD are significantly different. We investigate the association between a labored versus unlabored primary CD and subsequent risk of PAS.

**Study Design**: Retrospective cohort study at a single hospital between 2011‐2022. Data was collected from the electronic records. This hospital serves a distinctive population characterized by a very high order parity and a low rate of CD (9%). Inclusion criteria was singleton pregnancies with a FIGO diagnosis of PAS. PAS participants were 1:1 matched by parity, previous CDs, and VBACs. Association between labored and non‐labored CD with PAS was estimated using multivariable regression models, adjusting for placenta previa, IVF, history of hemorrhage, and a history of dilatation and curettage.

**Results**: 128,485 births were recorded during the study period, with 45 PAS diagnoses (0.04%) matched with 45 pregnancies with similar clinical risk factors without PAS. Median parity was 5.0, with 44% (*n* = 20) having undergone ≥3 previous CDs. A primary CD during labor was a significant risk factor for PAS (aOR 6.3, 95% CI 1.7‐23.3, *p* < 0.05). Consistent with known risk factors, placenta previa and history of hemorrhage were associated with PAS (aOR 16 95% CI 3.5‐81.1 and aOR 9.9 95% CI 1.6‐61.8, respectively). No significant differences were demonstrated in maternal and neonatal outcomes between pregnant people who underwent a labored versus unlabored primary CD (*p* > 0.05).

**Conclusion**: Labored CD versus an unlabored primary CD increases the risk of PAS in subsequent pregnancies by 6‐fold. Translation studies are needed to understand the molecular changes in the decidua during labor (vs non‐labor) that modulate this risk for PAS following a labored CD.

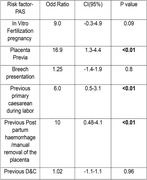

## 73 | Interval between cerclage removal and delivery in women who deliver term

Maya Kochhar^1^, Laura CB. van der Krogt^2^, Natalie Suff^3^, Andrew Shennan^1^

^1^King's College London, King's College London, England; ^2^King's College London, London, England; ^3^Kings College London, London, England

12:15–13:30

**Objective**: The purpose of this study was to determine the time between cerclage removal and spontaneous delivery, in women who delivered at term in a pregnancy with transvaginal cerclage.

**Study Design**: This was a retrospective cohort study of women who received a transvaginal cerclage and delivered subsequently at term. Demographic data, indication for cerclage insertion, gestation at cerclage removal (which routinely occurred around 37 weeks’ gestation) and gestation at delivery were collected. The interval between removal and delivery was calculated.

**Results**: An initial cohort of 39 women with transvaginal cerclage and term delivery were identified, of which 8 women were delivered by an elective caesarean section and 10 were induced. Therefore, the remaining 21 women, who went into spontaneous labour, were included for analysis.

Of these women, 48% (*n* = 10) had a history‐indicated cerclage, 48% (*n* = 10) had an ultrasound indicated cerclage and 4% (*n* = 1) had an emergency rescue cerclage.

The mean interval between cerclage removal and delivery was 15 days. 10% of women delivered within 48 hours of cerclage removal and 20% delivered within 7 days, whilst 48% delivered within 14 days and 76% delivered within 21 days.

There was no difference in interval to delivery between ultrasound‐indicated/emergency rescue cerclage and history‐indicated cerclage–difference of the means −1.89 days (confidence interval −9.35 to 5.57; p value 0.62).

**Conclusion**: The mean interval between cerclage removal and delivery is 15 days. Only 10% of women deliver within 48 hours of cerclage removal. 80% of women will deliver after one week. There was no significant difference in interval to delivery between ultrasound‐indicated/rescue cerclage and history‐indicated cerclage. This small retrospective cohort study adds to the existing literature about term cerclage removal, which can be used to counsel patients.

## 74 | Adverse perinatal outcomes in small for gestational age fetuses: An Italian multicenter retrospective cohort study

Laura La Milia^1^, Sara Consonni^2^, Isabella Crippa^3^, Eloisa Maria Mariani^3^, Irene Cameroni^3^, Stefania Dell'Oro^1^, SIlvia Alongi^4^, Valeria Fibioli^4^, Giulia Liberati^4^, Anna Locatelli^3^, Sara Ornaghi^3^

^1^Department of Obstetrics and Gynecology, Lecco Hospital, ASST Lecco, Lecco, Lombardia; ^2^Department of Obstetrics and Gynecology, Carate Hospital, ASST Brianza, Carate, Lombardia; ^3^Department of Obstetrics and Gynecology, Unit of Obstetrics, Fondazione IRCCS San Gerardo dei Tintori, Monza, Lombardia; ^4^University of Milan‐Bicocca School of Medicine and Surgery, Monza, Lombardia

12:15–13:30

**Objective**: To predict adverse perinatal outcomes (APOs) before delivery in late‐onset small for gestational age (SGA) fetuses.

**Study Design**: A multicenter retrospective cohort study carried out at three Italian maternity units of the same academic network including singleton pregnancies with a SGA fetus (AC or EFW < 10^th^ centile not meeting Delphi consensus criteria for fetal growth restriction, FGR) diagnosed after 32 weeks, from 06.2011 to 12.2022. Cases with last ultrasound examination (US) performed >2 weeks before delivery were excluded. APOs included operative delivery for non‐reassuring fetal status, 5‐minute Apgar score < 7, umbilical artery cord pH < 7.1, or base excess > −12 mmol/L. A multivariate analysis adjusted for confounders was performed to build the predictive model (R Open Source Software).

**Results**: During the study period, 288 SGA fetuses were diagnosed. APOs occurred in 47 (16.3%) of them. SGA fetuses experiencing APOs more commonly had nulliparous mothers (78.7% vs 61.4%, *p* = 0.036), MCA PI < 5^th^ centile and mean UtA PI >95^th^ centile at last US (18.2% vs 7.2%, *p* = 0.046 and 55.6% vs 26.5%, *p* = 0.001, respectively). Multivariate analysis for APOs prediction showed nulliparity and mean UtA PI >95^th^ centile as significantly associated with APOs (aOR 2.17, 95%CI, 1.03‐4.93; 4.46, 95% CI 2.40‐9.15, respectively). The formula calculating the probability of having an APOs in a late‐onset SGA fetus is shown in Figure 1. The AUC is 0.73, with a specificity of 78%, sensitivity of 60%, and accuracy of 75%.

**Conclusion**: This study provides a fairly performant, simple‐to‐use model to predict APOs within 2 weeks before delivery in late‐onset SGA fetuses, that could be useful in planning fetal monitoring and timing of delivery. Our study supports the pivotal role of UtA in discriminating SGA fetuses at risk of APOs.

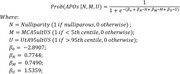

## 75 | Biomarker analysis of amniotic fluid to facilitate genomic sequencing variant interpretation in mucopolysaccharisdoses

Billie R. Lianoglou^1^, Juan Gonzalez^1^, Sarah Young^2^, Michael Gelb^3^, Zackary Herbst^3^, Emma Canepa^4^, Akos Herzeg^5^, Deeksha Bali^2^, Jennifer Cohen^2^, Priya Kishnani^2^, Beltran Borges^4^, Paul Harmatz^6^, Tippi C. MacKenzie^7^

^1^Center for Maternal‐Fetal Precision Medicine, University of California San Francisco, San Francisco, California; ^2^Duke, Durham, North Carolina; ^3^University of Washington, Seattle, Washington; ^4^University of California, San Francisco Center for Maternal Fetal Precision Medicine, San Francisco, California; ^5^UCSF, San Francisco, California; ^6^University of California, San Francisco Center for Maternal Fetal Precision Medicine, Oakland, California; ^7^University of California, San Francisco, San Francisco, California

12:15–13:30

**Objective**: Advances in fetal therapy for monogenic disease necessitate improved diagnostics to accurately identify eligible pregnancies. Variants of uncertain significance (VOUS) identified by genomic sequencing limit fetal diagnosis and warrant additional tools. We sought to assess the measurement of glycosaminoglycans (GAGs) as a biomarker in amniotic fluid (AF) to aid in the diagnosis of pregnancies affected by mucopolysaccharidoses.

**Study Design**: The University of California, San Francisco has an ongoing Phase I in‐utero enzyme replacement therapy (IUERT) clinical trial (NCT04532047). AF samples were obtained at each IUERT procedure from a fetus with MPS I (three collections, 25.9 to 32.3 weeks) and a fetus with MPS II (five collections, 25.1 to 34.7 weeks), and compared with AF from unaffected controls (median gestational age: 24.9 weeks, range: 18.0‐35.1, *n* = 74). We evaluated GAGs using two methods, the first by quantifying Heparan sulfate (HS), dermatan sulfate (DS), and chondroitin sulfate (CS) in AF as uronic acid‐hexosamine dimers produced by methanolysis and the second measuring MPS type specific endogenous non‐reducing ends (NREs). Both methods then apply liquid chromatography‐tandem mass spectrometry to quantify the GAG‐derived biomarker.

**Results**: Both approaches were able to clearly distinguish GAG values in the AF of affected pregnancies compared to controls. By methanolysis, HS levels were elevated relative to controls in both affected fetuses. DS was not elevated relative to controls for either patient at any time point but trended in the high‐normal range. NREs specific for MPS I and MPS II clearly differentiated between affected pregnancies and controls.

**Conclusion**: These data demonstrate that evaluation of GAGs in AF can differentiate between fetuses with MPS I and II and unaffected controls. Evaluation in additional affected pregnancies will aid in demonstrating the utility of these assays in confirming a prenatal diagnosis of MPS in the setting of genomic VOUS. Changes in HS levels during and following prenatal therapy may be useful for monitoring therapeutic response during IUERT.

## 77 | Safety of tranexamic acid in obstetrics: A systematic review and metaanalysis of clinical trials

Amairani Méndez Vionet^1^, Layra Mena García^1^, Angélica Daniela Hidalgo Maldonado^1^, Ana Lucía Fernández Macías^1^, María Emilia Garza‐Galván^2^, Mariana Moncada Madrazo^3^, Paul Arthur Bain^4^, Ana S. Ferrigno^5^, Mario Isaac Lumbreras Márquez^1^

^1^Epidemiology and Public Health Division, Universidad Panamericana School of Medicine, Mexico City; ^2^Escuela de Medicina y Ciencias de la Salud, Tecnológico de Monterrey, Monterrey., Monterrey, Nuevo Leon; ^3^Inova Fairfax Hospital., Inova Fairfax Hospital., Virginia; ^4^Countway Library, Harvard Medical School, Countway Library, Harvard Medical School, Massachusetts; ^5^Department of Medicine, Yale University School of Medicine, New Haven, USA., Department of Medicine, Yale University School of Medicine, New Haven, USA., Connecticut

12:15–13:30

**Objective**: To determine the safety profile of tranexamic acid (TXA) in treating and preventing postpartum hemorrhage (PPH) during vaginal or cesarean delivery.

**Study Design**: Systematic review and meta‐analysis of clinical trials where TXA or placebo/no intervention were randomly assigned as part of the study protocol in vaginal or cesarean deliveries. The primary aim was the incidence of thromboembolic events (i.e., composite outcome including arterial thrombosis, superficial and deep vein thrombosis, pulmonary embolism, visceral thrombosis, myocardial infarction, and stroke). Secondary outcomes included maternal mortality and mild adverse effects (i.e., composite outcome including nausea/vomiting, dizziness, diarrhea, urticaria, dermatitis, blurred or other changes in vision). Respective weights of the selected trials and pooled effect size were calculated using the inverse‐variance method using a random‐effects model with a continuity correction for studies with zero cell counts. Bias risk assessment was performed using the Risk of Bias 2 tool, and the quality of evidence was appraised using the GRADE criteria.

**Results**: A total of 34 randomized controlled trials with 44,016 women were included in the primary analysis. The meta‐analysis showed no significant differences between TXA and placebo in the incidence of thromboembolic events (odds ratio [OR] 0.95; 95% confidence interval [CI] 0.83, 1.10; I^2^ = 0%; *p* = 0.486; low‐quality evidence; Figure). Notably, TXA was associated with a reduction in maternal mortality (OR 0.88; 95% CI 0.84, 0.92; I^2^ = 0%; *p* < 0.001; low‐quality evidence). Furthermore, exposure to TXA was associated with an increased risk of mild adverse events (OR 1.71; 95% CI 1.16, 2.53; I^2^ = 87%; *p* = 0.010; low‐quality evidence).

**Conclusion**: In this systematic review and meta‐analysis, the use of TXA to prevent or treat postpartum hemorrhage was not associated with an increased risk of thromboembolic events. Moreover, a significant reduction in maternal mortality in women exposed to TXA was observed. However, the use of TXA is associated with an increase in mild adverse effects.

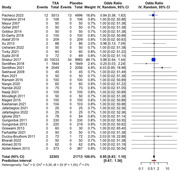

## 78 | Cervical length changes for prediction of spontaneous onset of labor at term: A longitudinal study

Puntabut Warintaksa^1^, Roberto Romero^2^, Adi L. Tarca^3^, Dereje Gudicha^4^, Supakorn Chaiyakarn^1^, Piya Chaemsaithong^5^

^1^Faculty of Medicine Ramathibodi Hospital, Mahidol University, ratchathewi, Krung Thep; ^2^Pregnancy Research Branch, Division of Obstetrics and Maternal‐Fetal Medicine, Division of Intramural Research, Eunice Kennedy Shriver NICHD, NIH, DHHS, Bethesda, Maryland, USA, Bethesda, Maryland; ^3^Center for Molecular Medicine and Genetics, Wayne State University School of Medicine, Detroit, Michigan; ^4^Perinatology Research Branch, NICHD, NIH, Mihigan, MD, United States., Detroit, Michigan; ^5^Faculty of Medicine Ramathibodi Hospital, Mahidol University, Bangkok, Krung Thep

12:15–13:30

**Objective**: The diagnosis of labor is the greatest challenge in clinical obstetrics as it is retrospective diagnosis. Cervical sonography provides objective assessment of cervical length (effacement/shortening) for definition of effacement. The objective of this study was to determine whether changes in cervical length can be detected prior to the onset of spontaneous labor at term.

**Study Design**: A longitudinal study was conducted to serially measure cervical length at every 2‐3 days intervals after term, starting at the 37 weeks of gestation in patients with uncomplicated pregnancy (*n* = 46). Spontaneous onset of labor was defined as spontaneous cervical dilation ≥4 cm and ≥80% effaced or spontaneous rupture of membranes with subsequent delivery within 24 hours of the onset of true labor. Linear mixed effect modeling was used to account for dependence due to serial cervical length measurements. Difference in cervical length between parous and nulliparous was adjusted for maternal characteristics (maternal weight and height) and gestational age at cervical measurement.

**Results**: 1) The median (range) cervical length examination was 6 times (1‐12) per participant; 2) women with cervical length percentile ≤10 had delivery within 2 days; and they were 3.34 times more likely to deliver than those with percentile >10 percentile; 3) nulliparous women had longer cervical length compared to parous women in the last 3 weeks prior to spontaneous and this difference was significant even after adjusting for maternal characteristics (Figure 1); 4) however, nulliparous women had subsequently reduction of cervical length more rapidly than multiparous women (i.e., faster reduced 2 mm per day compared to multiparous women)

**Conclusion**: Cervical length shortening, a component of the common pathway parturition, can be detected 3weeks prior to the onset of labor at term. Changes in cervical length differ according to parity status. Cervical length measurement might be used as an index of onset of labor at term.

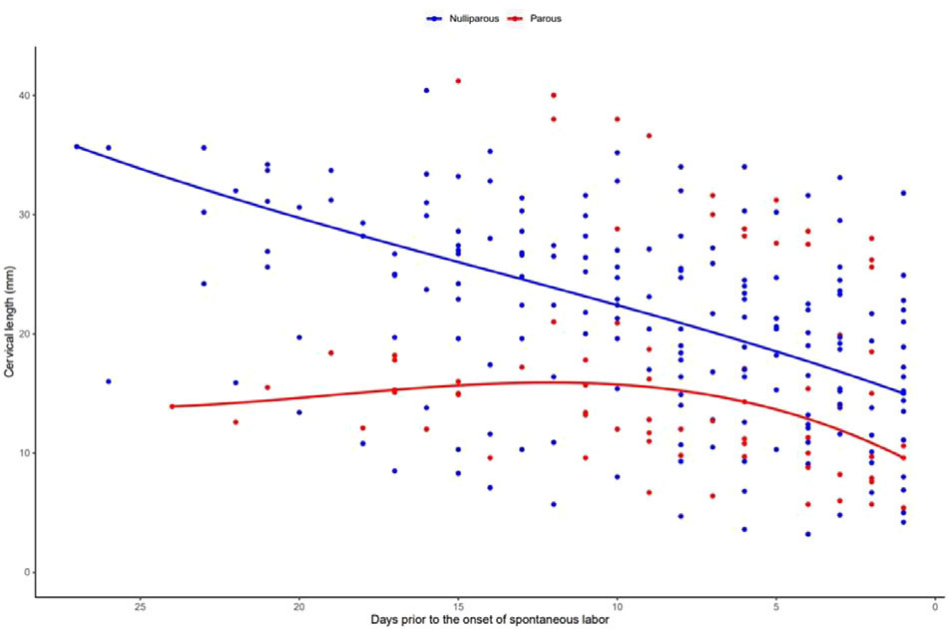

## 79 | Brain signal for spontaneous onset of labor at term: A CSF proteomics study

Puntabut Warintaksa^1^, Roberto Romero^2^, Jirawan Weeraphan^1^, Thanasikan Limpiti^1^, Lisa Sangkum^1^, Pakjira Sombatthaveekul^1^, Tossapol Homthain^1^, Chuthit Keesakul^1^, Kanyaphat Kotchompoo^1^, Wararat Chiangjong^1^, Piya Chaemsaithong^3^

^1^Faculty of Medicine Ramathibodi Hospital, Mahidol University, ratchathewi, Krung Thep; ^2^Pregnancy Research Branch, Division of Obstetrics and Maternal‐Fetal Medicine, Division of Intramural Research, Eunice Kennedy Shriver NICHD, NIH, DHHS, Bethesda, Maryland, USA, Bethesda, Maryland; ^3^Faculty of Medicine Ramathibodi Hospital, Mahidol University, Bangkok, Krung Thep

12:15–13:30

**Objective**: To determine brain signal for spontaneous onset of labor by the examination of cerebrospinal fluid (CSF) proteomics profile in women presenting with spontaneous onset of labor at term.

**Study Design**: This is a cross‐sectional study including 10 women at term gestation with and without spontaneous onset of labor (*n* = 5 each). CSF was obtained prior to Cesarean delivery. Cells and debris in CSF were removed by centrifugation. The proteins in CSF were dissolved in reducing buffer before total protein amount measurement by Bradford’ assay. Equal protein amount in each sample was reduced, alkylated, and digested before performing label‐free quantification using SWATH‐proteomic and protein bioinformatic analyses (STRING protein‐protein interaction and Reactome pathway analysis).

**Results**: Twelve CSF protein analytes increased in abundance in women with spontaneous onset of labor compared to those without labor (fold change >1.5 and unpaired student's t‐test at *p* < 0.05). The most upregulated protein was centrosome‐associated protein ALMS1 followed by amyloid beta precursor like protein‐1and prostaglandin‐H2 (fold changes 2.5, 2.4 and 2.3, respectively). Moreover, the protein‐protein interaction revealed that 9 in 12 proteins involving extracellular exosome, i.e., C3, APOE, ALB, APOD, FAM3C, PTGDS, CST3, TF, and NPC2. Furthermore, the proteins changing with labor were enriched in those associated with biological processes including protein phosphorylation, insulin‐growth factor transport, lipid remodeling and platelet degranulation (Figure 1). These may imply that protein signal release in form of extracellular exosome into CSF at the time of labor.

**Conclusion**: 1) The CSF proteome increased at the time of labor; 2) proteins involved in lipoprotein remodeling and platelet degranulation were differentially upregulated expressed; 3) in the future, intersecting the maternal plasma and CSF proteome could identify biomarker for the diagnosis of labor.

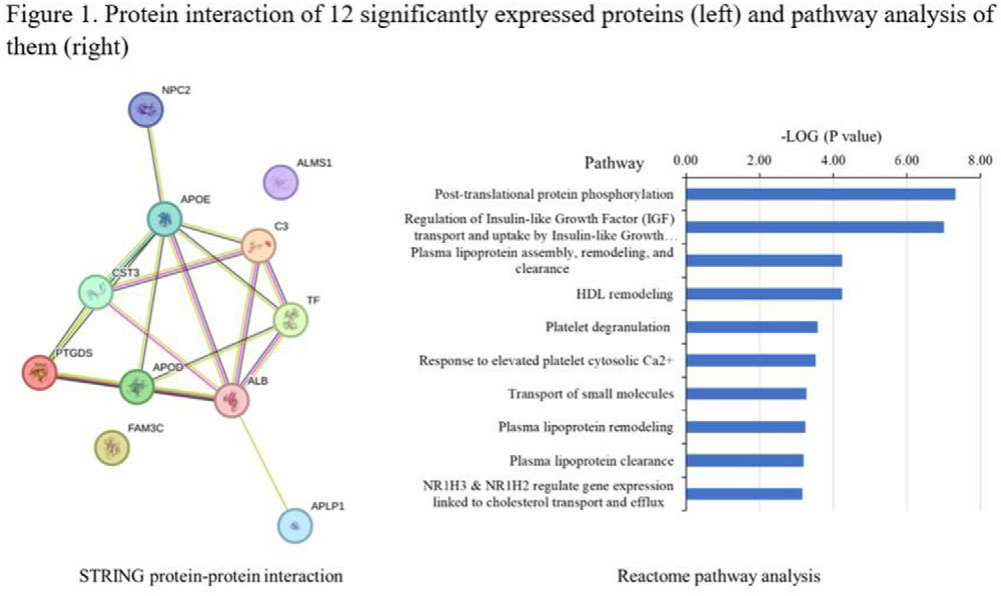

## 80 | Does advanced cervical dilation imply fast approaching delivery in a preterm gestation?

Miguel Bonilla Moreno^1^, Lanbo Yang^2^, Bhaskari Burra^3^, Alison Key^4^, Vidda Simpson^5^, Johanna Higdon^6^, Pratibha Rayapati^4^, Irene Stafford^7^, Chi Dola^8^

^1^Tulane University School of Medicine Department of OBGYN, New Orleans, LA; ^2^Tulane University School of Medicine, Tulane University School of Medicine, Louisiana; ^3^Mountain Area Health Education Center, Asheville, North Carolina; ^4^Tulane University School of Medicine Department of OBGYN, Tulane University School of Medicine/ New Orleans, Louisiana; ^5^Tulane University School of Medicine Department of OBGYN, East Jeff General Hospital/New Orleans, Louisiana; ^6^Tulane University School of Medicine Department of OBGYN, Eugene, Oregon; ^7^LSU Department of OBGYN, LSU/New Orleans, Louisiana; ^8^Tulane University School of Medicine Department of OBGYN, New Orleans, Louisiana

12:15–13:30

**Objective**: To assess the likelihood of imminent delivery in preterm labor with advanced cervical dilation and identify factors linked to a prolonged latency period from admission to delivery.

**Study Design**: A retrospective cohort study of singleton gestations at two New Orleans hospitals was conducted. Patients with preterm labor (defined as cervical dilation of ≥ 3 cm with contractions) were included. Information on maternal and neonatal demographics, complications, and outcomes were collected. Student t‐test, Chi‐square test, and multiple regression analysis were performed to assess for associations.

**Results**: Two hundred and twenty‐nine patients met inclusion criteria. This cohort had several risk factors for preterm delivery: history of preterm delivery (27.9%), high BMI (29.5 ± 7.1), tobacco use (14.4%), illicit drug use (10.5%), GBS carrier status (19.6%), and other urogenital infections (28.4%). Moreover, 46.7% had medical and pregnancy‐related comorbidities. Mean gestational age on admission was 33.5 ± 3.9 weeks with mean cervical dilation of 4.7 ± 2.1 cm and mean cervical effacement of 72 ± 21.6%. Mean gestational age at delivery was 34.2 ± 3.7 weeks. While most (70.4%) progressed to delivery within 24 hours, 29.6% delivered > 24 hours after admission. Furthermore, 23.5% delivered > 48 hours, and 15.2% delivered > 7 days after admission. Of note, 10% of patients delivered within 24 hours of admission due to a decision to augment labor or proceed to Cesarean delivery based solely on advanced cervical dilation on admission.

**Conclusion**: Our findings suggest advanced cervical dilation may not be associated with imminent delivery in patients presenting in preterm labor. Approximately 1/3 of preterm patients with advanced cervical dilation remained pregnant 24 hours after admission, 1/4 beyond 48 hours after admission, and almost 1/5 remained pregnant greater than 7 days after admission. Further research is needed to assess for optimal predictors of imminent delivery in the setting of preterm labor which may include gestational age, degree of cervical dilation and effacement, and regularity of contractions.

## 81 | Low lying and placenta previa: Determining the need for follow‐up ultrasounds

Elizabeth Mercer^1^, Rylie Wada^2^, Courtney Ip^2^, Dena Towner^2^

^1^University of Hawaii, University of Hawaii, HI; ^2^University of Hawaii, University of Hawaii, Hawaii

12:15–13:30

**Objective**: Our objective was to determine the need for follow‐up ultrasounds to evaluate placenta previa or low lying placentas based on the distance from the placental edge to the internal os at the time of the second trimester ultrasound.

**Study Design**: The study population was identified from the singleton obstetrical ultrasounds performed at a single institution from January 2013 to December 2018. Studies with a diagnosis code for placenta previa or low lying placenta and gestational age of 18 to 24 weeks were identified. The distance from the placenta edge to the internal os was extracted from the report and images. Subsequent third trimester ultrasounds were analyzed for resolution. Of the 914 patients who met the search criteria, 716 met the study criteria and were analyzed. 198 patients were excluded due to not having a low lying or placenta previa after transvaginal ultrasound, no follow‐up ultrasound, multiple gestations, placenta accreta, or vasa previa. The data was analyzed based on location of placenta: posterior and anterior. The percentage of resolution (more than 20mm from the os) and the likelihood ratios based on the distance the placenta was from the internal os during the second trimester ultrasound were calculated.

**Results**: The rate of placenta previa and low lying placenta resolution at the last performed ultrasound was 85.3% (611/716). Seventy‐four percent (530/716) had posterior placentas. For posterior placentas, 96.2% (172/179) of the low lying placentas that were 10mm or more from the internal os resolved. For anterior placentas, 100% (68) of the low lying placentas that were 10mm or more from the internal os resolved.

**Conclusion**: Given recommendations that a trial of labor is appropriate for placentas 10mm or more from the os and the high rate of resolution for both anterior and posterior placentas at this distance away, it is reasonable to consider not repeating third trimester ultrasounds for patients with low lying placentas that are 10mm or more away from the internal os. In this study population, these recommendations would have reduced 287 follow‐up ultrasounds.

## 82 | Novel microarray analyzer to predict major obstetric syndromes using surface markers of plasma‐derived extracellular vesicle

Marei Sammar^1^, Malene Moller Jorgensen^2^, Rikke Bæk^3^, Manu Vatish^4^, Jenni Sloth^5^, Rami sammour^6^, Hamutal Meiri^7^

^1^Braude College of Engineering, Braude College of Engineering, Karmiel, HaZafon; ^2^Department of Clinical Immunology, Aalborg University Hospital, Aalborg, Department of Clinical Immunology, Aalborg University Hospital, Aalborg, Nordjylland; ^3^University Hospital, Aalborg, Aalborg University Hospital, Aalborg, Nordjylland; ^4^Nuffield Department of Women's & Reproductive Health, University of Oxford, Nuffield Department of Women's & Reproductive Health, University of Oxford, Oxford, United Kingdom, England; ^5^Department of Clinical Immunology, Aalborg University Hospital, Department of Clinical Immunology, Aalborg University Hospital, Nordjylland; ^6^Bnai Zion MC, Bnai Zion MC, Hefa; ^7^Department of Obstetrics and Gynecology, Shamir (Assaf Harofeh) Medical Center, TeleMarpe, Tel Aviv

12:15–13:30

**Objective**: Evaluate a novel Multiple MicroArray in discovering the surface markers of plasma extracellular vesicle (EVs) predicting preterm delivery (PTD) and preeclampsia (PE) over matched term delivery controls (TD)

**Study Design**: Pregnant women, gestational weeks 24‐40 with PTD (*n* = 16), PE (*n* = 19) and matched TD controls (*n* = 15) were triaged at Bnai Zion Medical Cener, Haifa, Israel following plasma samples tested in the microarray using glass slides printed with 17 antibodies to EV surface receptors (selected of initial 85 tested). PBS and whole human Ig were used as control. Slides were then incubated with raw plasma samples, detected by a cocktail of biotinylated antibodies specific to EVs biomarkers or to placental EV (PEV), and labelled by cyanine 5–streptavidin. The fluorescent signal was measured as the ratio to negative control, and log 2 transformed. Results verified by comparison with pure EVs from standard purification procedures, validated by nanoparticle size tracking analysis.

**Results**: Heatmaps separate surface profiles of PE, PTD, and TD receptors of total EVs and PEVs. Similar results obtained with purified EV and raw plasma EVs. Univariate analyses scored markers predicting PTD and PE over TD control with area under the receiver operation characteristic curve (AUC of ROC) >0.6 and sensitivity >50% at 80% specificity. Best one combined in multi‐variate analysis. AUC = 0.89 (95% CI: 0.72‐1.0) for PE prediction over TD with 90% sensitivity at 90% specificity obtained with surface receptors of inflammation (TNF alpha), relaxation (PP13), and immune‐modulation (LFA1). PTD prediction over TD had AUC = 0.97 (0.94‐1.0), 84% sensitivity at 90% specificity marked by cell adhesion (ICAM), and EV general markers (CD81, CD82 and Alix). PE prediction over PTD had AUC = 0.91 (0.79‐0.99) with 80% sensitivity and 90% specificity for the complement activation cascade (C1q) and autoimmunity.

**Conclusion**: A new, simple, fast, robust EV microarray analyzer is a new diagnostic device that reveal novel surface markers of major obstetrics syndromes.

## 83 | Seasonal patterns of spontaneous and iatrogenic preterm birth in the netherlands

Annabelle L. van Gils^1^, Anita C. Ravelli^1^, Brenda M. M. Kazemier^1^, Eva Pajkrt^1^, Martijn A. Oudijk^2^

^1^Amsterdam UMC, Amsterdam, Noord‐Holland; ^2^Amsterdam UMC, location University of Amsterdam, Amsterdam, Noord‐Holland

12:15–13:30

**Objective**: Preterm birth (PTB) is subject to seasonal periodicity, dependent on geographical, meteorological and environmental factors. This study aims to assess whether seasonal periodicity of PTB can be found in the Netherlands, using three different approaches of which a fetus‐at‐risk approach.

**Study Design**: We performed a population‐based cohort study including singleton births between 24^+0^–42^+6^ weeks’ gestation between January 1^st^ 2010–December 31^st^ 2019. Cases of intra uterine fetal demise or congenital anomalies were excluded. Primary outcome was PTB < 37 weeks. Secondary outcomes were total, spontaneous and iatrogenic PTB < 28, 28‐32 and 32‐37 weeks GA. PTB incidences were calculated as a proportion of births per month/season of birth, per month/season of conception and using fetus‐at‐risk approach taking into account the number of ongoing pregnancies at risk for PTB. A trend line of PTB per fetus‐at‐risk was plotted for the ten year study period. Multivariate logistics regression analysis was used to estimate odds ratios and the 95% confidence interval (CI).

**Results**: In total 1,598,554 singleton pregnancies were included. The mean incidence of PTB < 37 was 5.16%. The incidence of PTB < 37 weeks per month of birth was highest in December (5.65%) and the lowest in September (4.68%). PTB < 37 weeks per month of conception was highest for conception in April (5.49%). Out of fetuses‐a‐risk, the highest incidences of PTB were found in the winter months December and January (respectively 1.40% and 1.38%) and the lowest incidence was found in April (1.23%). The ten year trend line of PTB incidences using the fetus‐at‐risk approach shows a recurring unimodal pattern, with highest incidences of PTB at the end of each year and lowest incidences in spring months. (Figure 1).

**Conclusion**: Fetuses are highest at risk for PTB in the winter months and lower risk in spring. Seasonal trends of PTB should be assessed in the light of the timelines of conception, ongoing pregnancy and moment of birth. A fetus‐at‐risk approach seems to be more comprehensive in evaluating the continuous risk that fetuses encounter.

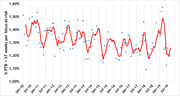

## 84 | Universal mid‐pregnancy maternal placental growth factor screening for the risk of early preterm birth

Rachel A. Gladstone^1^, Sumaiya Ahmed^2^, Ella Huszti^3^, Kellie Murphy^4^, John C. Kingdom^5^

^1^Department of Obstetrics & Gynaecology, University of Toronto, Toronto, ON; ^2^Mount Sinai Hospital, Department of Obstetrics & Gynaecology, Mount Sinai Hospital, Ontario; ^3^Biostatistics@UHN, Toronto, Ontario; ^4^University of Toronto, Toronto, Ontario; ^5^Dept OBGYN Mount Sinai Hospital University of Toronto, Toronto, Ontario

12:15–13:30

**Objective**: Early preterm birth (PTB) < 34 weeks confers a high risk for adverse health outcomes. Currently, no universal screening strategy exists for early PTB, preventing targeted delivery of effective interventions. We aimed to evaluate the association between mid‐pregnancy placental growth factor (PlGF) and early PTB in the general obstetric population.

**Study Design**: We performed a prospective cohort study among unselected, singleton pregnant people. Participants underwent a placental growth factor (PlGF) test at the time of routine gestational diabetes mellitus (GDM) screening, typically at 24‐28 weeks. The primary outcome was all PTB < 34 weeks.

**Results**: Among 9,037 independent pregnancies, 156 (1.7%) experienced PTB < 34 weeks (spontaneous *n* = 52, iatrogenic *n* = 104). Low PlGF was predictive of PTB < 34 weeks (area under the curve [AUC] 0.80, 95% CI 0.75‐0.85) at an optimal threshold of 290.5 pg/mL; however, time to birth from PlGF test was significantly lower among patients with PlGF < 100 pg/mL, for whom >50% delivered within 50 days of testing (aRR PTB < 34 weeks 48.23, 95% CI 36.55‐63.64). The association was unaffected by gestational age of testing, race, or pre‐pregnancy weight. The AUC for iatrogenic PTB < 34 weeks was 0.90 (95% CI 0.85‐0.94). Stillbirth occurred in 36 (0.4%) pregnancies. The relative risks for stillbirth (aRR 36.78, 95% CI 18.63‐72.60) and preeclampsia (aRR 11.01, 95% CI 8.85‐13.69) were significantly increased if PlGF < 100 pg/mL.

**Conclusion**: Low PlGF, identified at the time of routine GDM screening, is an effective clinical tool to identify pregnant people at risk of early PTB, especially in the majority that is iatrogenic. Strategic redirection of tertiary healthcare resources to this high‐risk group could improve maternal and perinatal outcomes.

## 85 | Fetal predictors of left ventricular dysfunction following pulmonary valve balloon dilation in critical pulmonary stenosis

Patrizio Moras^1^, Luciano Pasquini^2^, Cosimo Marco Campanale^1^, Marco Masci^1^, Maria Carolina Colucci^1^, Serena Ventrella^1^, Alessandra Toscano^1^

^1^Bambino Gesù Pediatric Hospital, Roma, Lazio; ^2^Policlinico Agostino Gemelli, Policlinico Agostino Gemelli, Lazio

12:15–13:30

**Objective**: Critical pulmonary stenosis (CPS) is a cyanotic congenital heart defect characterized by complete obstruction to the right ventricular (RV) outflow tract and is associated with various degrees of RV hypoplasia. Left ventricular (LV) systolic dysfunction is a complication occurring in up to 35% of patients after transcatheter pulmonary valve balloon dilation (PVBD). To date, no study has examined the fetal risk factors of this complication, which plays a crucial role in post‐procedural intensive management.

**Study Design**: The aims of this study were to determine whether fetal echocardiographic examination can identify risk factors for LV dysfunction after PVBD in patients diagnosed with CPS. Twenty‐one neonates with a prenatal diagnosis of CPS between 2017 and 2023 were retrospectively analyzed. Fetal diameters of the atrioventricular and semilunar valves, RV anatomy and RV systolic and diastolic function were assessed in the second and third trimester. Neonates were divided into two groups based on the development of post‐PVBD LV dysfunction. The newborns who did not develop it were assigned to Group 1, while those who did to Group 2.

**Results**: Compared to group 1 fetuses, those who developed post‐PVBD LV dysfunction (group 2) had significantly higher tricuspid valve (TV) annulus z‐score (‐1.2 ± 1.4 vs. 0.2 ± 0.9, *p* = 0.022) and a lower mitral valve/tricuspid valve ratio (1.2 ± 0.2 vs 0.8 ± 0.1, *p* = 0.030). Group 2 fetuses also had a higher prevalence of a tripartite right ventricle (1 (8.3%) vs. 11 (91.7%), *p* = 0.002, OR 9.3, 95% CI 1.3‐62.9) while group 1 fetuses had a higher prevalence of a hypoplastic infundibulum (7 (70%) vs. 3 (30%), *p* = 0.008, OR 3.0, 95% CI 1.2‐7.9). Similar associations were found at the third trimester examination.

**Conclusion**: Most patients with indices of large and tripartite right ventricles developed LV systolic dysfunction following PVBD. The assessment of RV anatomy during fetal examination provides crucial insights into the immediate post‐PVBD period for both parents and clinicians.

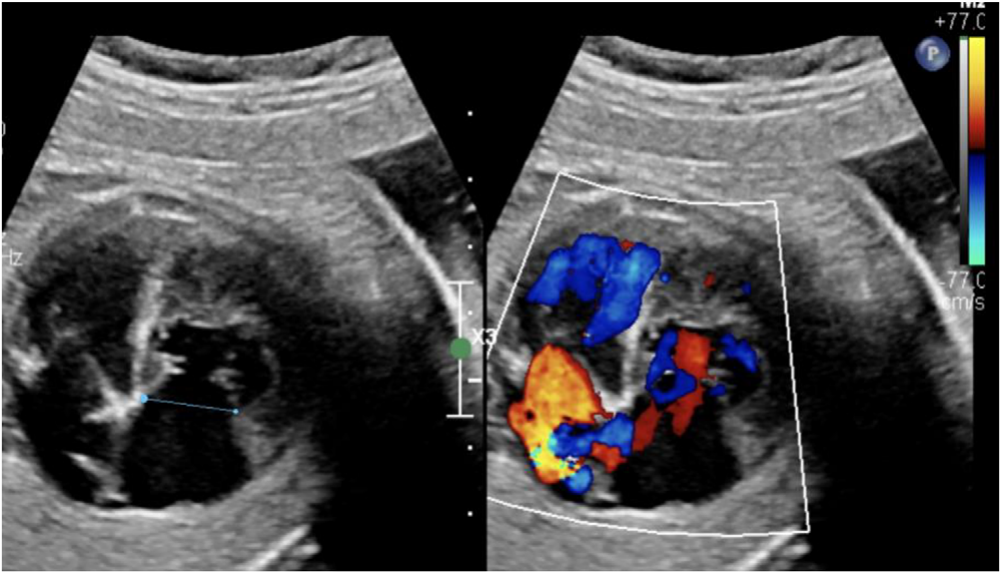

## 86 | Fetal massive subdural hematoma with midline shift: In‐utero hematoma evacuation with favorable outcomes: Case series

Ahmed Amdihun. Essa^1^, Amsalu Worku. Mekonnen^1^, Daniel Misikir. Hailu^2^, Waltenigus Terefe^2^

^1^Bahir Dar University teaching Hospital, BAHIR DAR, Amara; ^2^BAHIR DAR UNIVERSITY, BAHIR DAR, Amara

12:15–13:30

**Objective**: To describe the clinical characteristics and outcomes of fetal massive subdural hematoma cases managed with in‐utero hematoma evacuation following intrauterine transfusion at a Maternal‐Fetal Medicine Center in Ethiopia.

**Study Design**: We retrospectively analyzed three cases of fetal subdural hematoma managed at our university hospital. Data on clinical presentation, in‐utero neurological intervention, fetal follow up and neonatal outcomes were collected through chart review. We performed individual case‐analysis and description of the clinical course and management outcomes of individual cases.

**Results**: There were 3 cases of fetal massive subdural hematoma cases detected antenatally using ultrasound. Three them presented at 29‐34 weeks of gestation. Fetal subdural hematoma cases were associated with significant midline shift. Sessions of intrauterine transfusion followed by fetal surgery (in‐utero needle hematoma evacuation) were carried out by Fetal medicine sub‐specialist. All procedures were done safely. there was no recollection of hematoma. All fetuses had Showen significant improvement in the midline shift, in secondary ventriculomegaly and in Biophysical profiles. All fetuses were delivered alive at term. The immediate neonatal outcomes were favorable in all the cases.

**Conclusion**: Being the first case series on in‐utero intervention (needle evacuation of fetal massive subdural hematoma). It was done safely, and recollection were not seen following needle evacuation. Our study implies that this prenatal intervention improves neonatal outcome by relieving compression and midline shift, which is a known poor prognostic factor. Immediate neonatal outcomes were favorable in three of the cases.

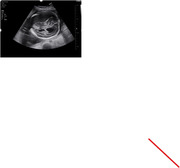

## 87 | Trial of labor after cesarean in individuals with and without diabetes: A population cohort study

Sarah A. Nazeer^1^, Han‐Yang M. Chen^2^, Hector M. Mendez‐Figueroa^3^, Ghamar Bitar^2^, Baha M. Sibai^2^, Michal Fishel Bartal^4^, Suneet P. Chauhan^5^

^1^The University of Texas Health Science Center at Houston (UTHealth), Houston, Texas; ^2^McGovern Medical School at UTHealth Houston, Houston, Texas; ^3^McGovern Medical School at UTHealth, Houston, Texas; ^4^UTH Houston & Sheba Medical Center Israel, Houston, Texas; ^5^Delaware Center of Maternal‐Fetal Medicine at Christiana Care, Delaware, Delaware

12:15–13:30

**Objective**: The primary purpose was to assess the rate of failed trial of labor cesarean delivery (TOLAC), among individuals with and without diabetes (DM); the secondary purpose, compare the adverse outcomes with TOLAC (irrespective of the eventual route of delivery) in the two groups.

**Study Design**: Our population‐based cohort study used the U.S. Vital Statistics data set (2016‐21) and evaluated individuals with singleton, no hypertensive disorders, who attempted TOLAC at 37 to 41 wks. The exposure variable was DM. The primary outcome was failed TOLAC. The secondary outcomes were composite neonatal adverse outcome (CNAO) and the maternal adverse outcome (CMAO). The sensitivity analysis examined whether the association of outcomes persisted if we stratified individuals by type of diabetes (no DM, pregestational DM, gestational DM). Multivariable Poisson regression models were used to estimate adjusted relative risks (aRR) and 95% confidence intervals (CI).

**Results**: Of the 22,685,620 live births during the time frame, 577,925 (2.5%) met the inclusion criteria. Of those pregnancies that underwent a TOLAC, 46,181 (8.0%) had DM, and 531,744 (92%) did not DM. The rate of failed TOLAC in those with DM compared to those without was significantly higher (40.3% versus 34.2%; aRR 1.05, 95% CI 1.04‐1.06). The CNAO was significantly higher among individuals with DM who had TOLAC compared to those without (47.6% versus 36.2%; aRR 1.24, 95% CI 1.19‐1.30). The CMAO was higher among individuals with DM who had TOLAC compared to those without (9.9% versus 8.5%; aRR 1.18, 95% CI 1.07‐1.30). A sensitivity analysis indicated that compared to individuals without DM, the risk of failed TOLAC was higher among those with pregestational DM (aRR = 1.22, 95% CI 1.19‐1.26) and in those with gestational DM (aRR = 1.03, 95% CI 1.02‐1.04; Table).

**Conclusion**: Among individuals with DM, versus those without, who attempted TOLAC, the likelihood of failure was 5% higher, and the composite neonatal and maternal adverse outcomes were 24% and 18% higher, respectively. Our findings may be useful for counseling individuals and for policymakers.

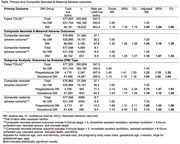

## 88 | Continuous glucose monitoring in individuals with diabetes in the postpartum period: A prospective study

Sarah A. Nazeer^1^, Rafael Bravo Santos^2^, Ghamar Bitar^3^, Claudia Pedroza^2^, Sean C. Blackwell^3^, Antonio F. Saad^4^, George R. Saade^5^, Suneet P. Chauhan^6^, Baha M. Sibai^3^, Michal Fishel Bartal^7^

^1^Division of Maternal‐Fetal Medicine, Department of Obstetrics, Gynecology and Reproductive Sciences, McGovern Medical School at UTHealth Houston, Houston, Texas; ^2^The University of Texas Health Science Center at Houston (UTHealth), Houston, Texas; ^3^McGovern Medical School at UTHealth Houston, Houston, Texas; ^4^Inova Health, Falls Church, Virginia; ^5^Macon & Joan Brock Virginia Health Sciences Eastern Virginia Medical School at Old Dominion University, Norfolk, Virginia; ^6^Delaware Center of Maternal‐Fetal Medicine at Christiana Care, Delaware, Delaware; ^7^UTH Houston & Sheba Medical Center Israel, Houston, Texas

12:15–13:30

**Objective**: The data on blood sugars during the postpartum period for individuals with diabetes is limited; practice depends on clinician preferences. Continuous glucose monitoring (CGM) provides personalized glycemic profiles. Our aim was to investigate CGM metrics in the immediate postpartum period.

**Study Design**: This was a secondary analysis of a multi‐center, prospective study (11/21 −12/22) of individuals with pregestational (DM) and gestational diabetes (GDM) at ≥34 weeks’ gestation. Participants had a blinded CGM (Dexcom G6 PRO) placed throughout their labor and delivery admission. CGM metrics evaluated included the mean glucose and time in range (TIR; range 70–180 mg/dL), time above the target range (TAR; >180mg/dL), and time below range (TBR; < 70mg/dL) were expressed as % of all CGM readings. Maternal outcomes evaluated were rates of postpartum hemorrhage, blood transfusion, endometritis, wound complications, new diagnosis of postpartum hypertension, breastfeeding at discharge and at 6‐week postpartum visit and 6‐week postpartum visit compliance. Based on the pilot study (PMID: 37088275), we compared outcomes among those with TAR ≥10% versus < 10%. Pearson's Chi‐squared and Fisher's exact test were calculated; p value of < 0.05 was considered as a statistically significant value.

**Results**: During the study period, 202 (10.5%) individuals met inclusion criteria and 112 (55.4%) participants were enrolled. Postpartum data was available for 82 (73.2%) participants: 47 (57.3%) had GDM and 35 (42.7%) had pregestational DM. The mean glucose using CGM was 120.4 mg/dL (IQR 108.3‐130.4). The average TIR was 95.5% (87.9, 99.1), average TAR was 0.9% (0.0, 10.3) and TBR was 0.2% (0.0, 2.7). There was no statistically significant difference in maternal outcomes in the immediate postpartum period for individuals with TAR ≥ 10% versus < 10% (Table).

**Conclusion**: In this analysis of individuals with diabetes during the postpartum period, there was no association between maternal adverse outcomes and TAR ≥10%. Further studies are needed to examine the appropriate thresholds for glycemic control in the postpartum period.

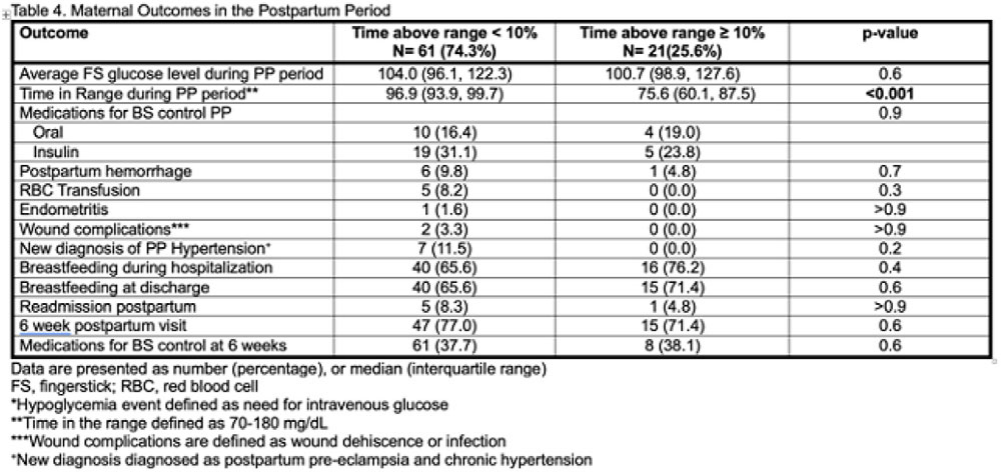

## 89 | Association between different single nucleotide polymorphisms and hyperglycemia in pregnancy in Ho Chi Minh City

Quan Duc Minh. Nguyen^1^, Vinh Nguyen. Dao^2^, Hoa Giang^2^, Thang Nhat. Tran^3^

^1^University Medical Center, Ho Chi Minh City, Ho Chi Minh City, Ho Chi Minh; ^2^Medical Genetics Institute, Medical Genetics Institute, Ho Chi Minh; ^3^University Medical Center, Ho Chi Minh City, University Medical Center, Ho Chi Minh City, Ho Chi Minh

12:15–13:30

**Objective**: We investigated the relationship between hyperglycemia in pregnancy (HIP) and different single nucleotide polymorphisms (SNPs) using non‐invasive prenatal testing (NIPT) data.

**Study Design**: This is a nested case‐control study, performed on a cohort of pregnant women, who had both an NIPT and an oral glucose tolerance test (OGTT). Women with pre‐existing type 2 diabetes (T2D) or a positive OGTT were defined as having HIP.

We reviewed the literature to designed a high priority SNPs panel, which includes 203 SNPs associated with GDM and 1057 SNPs associated with T2D. For the association test, we pooled sequencing data from the case and control groups to create 2 genomic samples. The SNPs prevalence between the 2 pooled samples were compared both directly and after STICH imputation. The associated SNPs were used to create a polygenic risk score (PRS) model.

**Results**: From 12/2017 to 07/2020, we found records of 351 eligible women. There were 110 cases, including 105 women with GDM and 5 women with T2D, and 241 control women. In the high priority SNPs panel tests, we found 31 SNPs associated with HIP. Among these, rs6235 and rs13179048 in PCSK1 were priorly linked to GDM, but not T2D. The remaining 29 SNPs were previously associated with T2D in large‐scale GWAS. Also, 4 of these variants, rs11709077, rs9348441, rs10962, and rs824248, were associated with both GDM and T2D.

The PRS distributions of cases and controls were characterized by normal distributions, with the PRS of cases significantly lower than that of controls (*p* = 2.2e‐16). The AUC of the PRS for predicting GDM was 0.85.

Due to our limited sample size, the PRS have not yet been validated independently. Also, our sequencing data was shallow as the tests were performed for NIPT purpose and we had to employ STITCH imputation model, of which accuracy at these high priority SNPs remains uncertain.

**Conclusion**: This study identified 31 SNPs associated with hyperglycemia in pregnancy. We also developed a PRS model which showed promises in stratifying risk of GDM for pregnant women and also for identifying individuals at high risk for T2D post‐pregnancy.

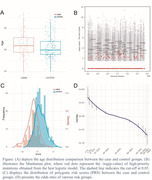

## 90 | Antenatal physical activity for improving pregnancy outcomes: A systematic review and meta‐analysis

Amanda J. Poprzeczny^1^, Andrea R. Deussen^2^, Laura Slade^3^, Megan Mitchell^2^, Jennie Louise^4^, Jodie Dodd^5^

^1^Women's and Children's Hospital, North Adelaide, Adelaide, South Australia; ^2^The University of Adelaide, Department of Obstetrics and Gynaecology and the Robinson Research Institute, Adelaide, South Australia; ^3^Women's and Children's Hospital, Adelaide, South Australia; ^4^Women's and Children's Hospital Research Centre, Adelaide, South Australia; ^5^Women's and Children's Hospital and The University of Adelaide, Department of Obstetrics and Gynaecology and the Robinson Research Institute, Adelaide, South Australia

12:15–13:30

**Objective**: Guidelines recommending regular physical activity in pregnancy for improving pregnancy outcomes are informed by published meta‐analyses. We sought to assess the validity of these recommendations by focusing on trial quality.

**Study Design**: This analysis formed part of a prospectively registered (PROSPERO ID CRD42022324220) systematic review and meta‐analysis investigating the effect of dietary and physical activity interventions on pregnancy outcomes, specifically evaluating antenatal physical activity interventions only. Trials were classified as at negligible, minimal, moderate or substantial risk of bias due to departures from the ‘Intention to Treat’ principle. Sequential meta‐analyses were undertaken to determine the impact on findings with inclusion of less rigorous trials.

**Results**: Only 6 trials were assessed as no/negligible risk of bias as result of departures from the ‘Intention to Treat’ principle. When considering only those trials at no/negligible risk of bias, there was no significant effect of physical activity interventions on infant birthweight (MD 20.97g; 95% CI −85.45, 127.40), or gestational weight gain (MD −0.60kg; 95% CI −2.17, 0.98). There was no significant effect of physical activity on the occurrence of preeclampsia, caesarean birth or large for gestational age infants. The inclusion of trials with increasing risk of bias moved the estimate of effect further from the null, but increased the width of confidence intervals producing a less precise result.

**Conclusion**: When considering only trials at no/negligible risk of bias, antenatal physical activity interventions were not associated with improved pregnancy outcomes. Most trials of antenatal physical activity interventions were not methodologically rigorous, and incorporation of such meta‐analyses into pregnancy care guidelines may result in inaccurate recommendations.

## 91 | Visit‐to‐visit blood pressure variability associated with development of maternal and neonatal adverse events in preeclampsia

I Gusti Bagus Mulia Agung Pradnyaandara^1^, Ryan Saktika Mulyana^1^

^1^Faculty Of Medicine, Udayana University, Faculty of Medicine, Udayana University, Bali

12:15–13:30

**Objective**: Blood pressure variability (BPV) is the result of complex interaction between behavior, environment, autonomic regulation, as well as the cardiovascular system. BPV is particularly important in hypertensive patients and is often associated with organ damage. In preeclampsia the role of BPV remains unclear. This study aims to evaluate the association of BPV in preeclampsia with maternal and neonatal outcomes.

**Study Design**: This cohort retrospective study enrolled 40 patients who visited maternal fetal medicine unit with diagnosis of preeclampsia with severe features. Patients were grouped according to BPV status, and maternal and neonatal outcomes were compared. Mean arterial pressure (MAP) was measured every outpatient counseling, and BPV was calculated as the coefficient of variation of MAP (MAP_CV_). MAP_CV_ values above the median were defined as high BPV and below as low BPV.

**Results**: Out of 40 patients, 50% had high BPV. The mean age in high BPV group was 34.15 ± 6.67 and low BPV was 33.83 ± 3.63 (*p* = 0.06). High BPV was associated with higher preterm birth (80% vs 20%; *p* < 0.001), and premature rupture of membrane (90.9% vs 9.1%; *p* = 0.005). High BPV was also associated with longer hospitalization time (6 ± 3.5 vs 4 ± 1.75; *p* = 0.001). In terms of neonatal outcomes, high BPV was associated with higher neonatal adverse event (76.5% vs 23.5%; *p* = 0.01), and admission to neonatal intensive care unit (90.9% vs 9.1%; *p* = 0.005). Infants born in the high BPV group also had lower birth weight (1.74 ± 1.47 vs 2.92 ± 1.37; *p* < 0.001), and shorter birth length (43.5 ± 9.75 vs 47.5 ± 4.75; *p* = 0.003). BPV is an independent variable that increases the risk of maternal adverse events (PR 13.65; 95% CI 2,26‐82,42; *p* = 0.004).

**Conclusion**: BPV has proven to play an important role in pregnancy. Considering BPV as a regular pregnancy screening parameter has the potential to improve maternal and neonatal outcomes.

## 92 | Trends and perinatal outcome of adolescent pregnancies: Not only an obstetric challenge

Jessica Preziosi^1^, Stefania Giacalone^2^, Gloria Anderson^3^, Michelangela Danza^2^, Antonio Lanzone^4^, Silvia Salvi^5^

^1^Fondazione Policlinico Agostino Gemelli, IRCCS, Rome, Italy, Fondazione policlinico Agostino Gemelli, Lazio; ^2^Università Cattolica del Sacro Cuore, Rome, Italy, Università Cattolica del Sacro Cuore, Lazio; ^3^Fondazione Policlinico Agostino Gemelli, IRCCS, Rome, Italy, Fondazione Policlinico Agostino Gemelli, IRCCS, Rome, Italy, Lazio; ^4^Fondazione Policlinico Agostino Gemelli, IRCSS, Rome Italy, Rome, Lazio; ^5^Fondazione Policlinico Agostino Gemelli, IRCSS, Rome, Italy, Fondazione Policlinico Agostino Gemelli, Lazio

12:15–13:30

**Objective**: Worldwide, 11% of the births are provided by adolescents between 15 and 19 years. This represents a major obstetric and social challenge, potentially affecting not only the perinatal wellbeing, but also mothers’ education, job opportunities and economic wellbeing. Thus, this study aims to describe the number and the trend of deliveries in adolescent girls giving birth in a primary referral centre of Rome, and to investigate their perinatal outcome.

**Study Design**: We undertook a retrospective study, analysing the data of all the pregnant women who had delivered in the last ten years at Fondazione Policlinico A. Gemelli‐IRCSS. Data on adolescents’ admission, pregnancy and discharge were obtained by inpatient medical records recorded electronically.

**Results**: Overall, a total of 43.201 women delivered from January 2013 to December 2023. Of all these deliveries, 0.6% (281) occurred in adolescents. The median maternal age was of 18.9 (IQR: 18‐19.5). Only seven girls (0.02%) had more than one delivery in the time range analysed. We observed a decreasing trend in the deliveries of adolescents (median 24, IQR 20‐31). Most of these pregnancies ended with vaginal delivery (228, 82%), while only 46 had a caesarean section (CS) (16%). The CS rate was lower than the overall national CS rate in the same period and with a declining trend (β = −0.006, *p* = 0.39). 22% (*n* = 61) had received an episiotomy, while 18% (*n* = 52) had spontaneous lacerations. Analysing the trend of episiotomies, the rates decrease of the 77.7%. Regarding obstetrical complications, the most frequent were pre‐partum anaemia (*n* = 26.9%), intrauterine growth restriction (IUGR) (*n* = 15.5%) and post‐partum haemorrhage (*n* = 6,2%). Only one intrapartum death was registered in a woman with psychiatric known disorder.

**Conclusion**: Our results suggest that, in Italy, adolescent pregnancies are not associated with worst perinatal outcome; these data refer to a high economic country with a free national system guaranteeing access to care for all and are therefore only applicable in that context.

## 95 | Cerclage efficacy following cervical caesarean damage

Hannah Rosen O'Sullivan^1^, Hannah Rosen O'Sullivan^1^, Laura CB. van der Krogt^1^, Agnieszka A. Glazewska‐Hallin^2^, Lisa Story^3^, Natalie Suff^4^, Andrew Shennan^5^

^1^King's College London, London, England; ^2^Guy's And St Thomas' NHS Foundation Trust, King's College London/Guy's and St Thomas' NHS Foundation trust, England; ^3^King's College London, King's College London/Guy's and St Thomas' NHS Foundation trust, England; ^4^Kings College London, London, England; ^5^King's College London, King's College London, England

12:15–13:30

**Objective**: Emergency caesarean sections (EMCS) are increasing worldwide. They also increase risk of spontaneous preterm birth (sPTB) and mid‐trimester loss (MTL) secondary to cervical damage sustained at caesarean. Recurrence is common with an absolute risk of > 50% even with vaginal cerclage, and 6‐fold risk of recurrent loss < 24 weeks.

Transvaginal cerclage (TVC) has been shown to be a less effective intervention following cervical caesarean damage (CCD), and is 10 times more likely to fail in this group than in those with other preterm birth risk factors. Transabdominal cerclage (TAC) has been demonstrated to be effective after full dilation EMCS.

This study compares outcomes of pregnancies with TAC or TVC in women with a history of any EMCS and subsequent sPTB or MTL.

**Study Design**: Retrospective cohort study of women with a history of MTL or sPTB following a previous term delivery by EMCS, receiving either TVC or TAC in one or more subsequent pregnancies. The primary outcome measure was live baby at discharge. Secondary outcome measures were delivery < 30 and delivery < 24 weeks.

**Results**: 25 pregnancies with TAC and 64 with TVC were included. 37% had EMCS before full dilatation. 100% of pregnancies with TAC resulted in a live baby at discharge compared with only 59% of pregnancies with TVC (chi‐square *p* < 0.001). None of the TAC group had a late miscarriage < 24 weeks, compared with 31% of the TVC group (chi‐square *p* < 0.001). Results were similar for FDCS vs those women with earlier EMCS in labour (55% vs 64% had live births respectively). Figure 1 demonstrates the survival curve of TAC vs TVC. In the 64 women with TVC, 45% delivered < 30 weeks.

**Conclusion**: Cervical caesarean damage is a common unrecognised cause of recurrent pregnancy loss and early preterm birth where transvaginal cerclage has a high failure rate. This data suggests abdominal cerclage maybe the treatment of choice in this very high risk group. Prospective trails are underway (ABOVE trial).

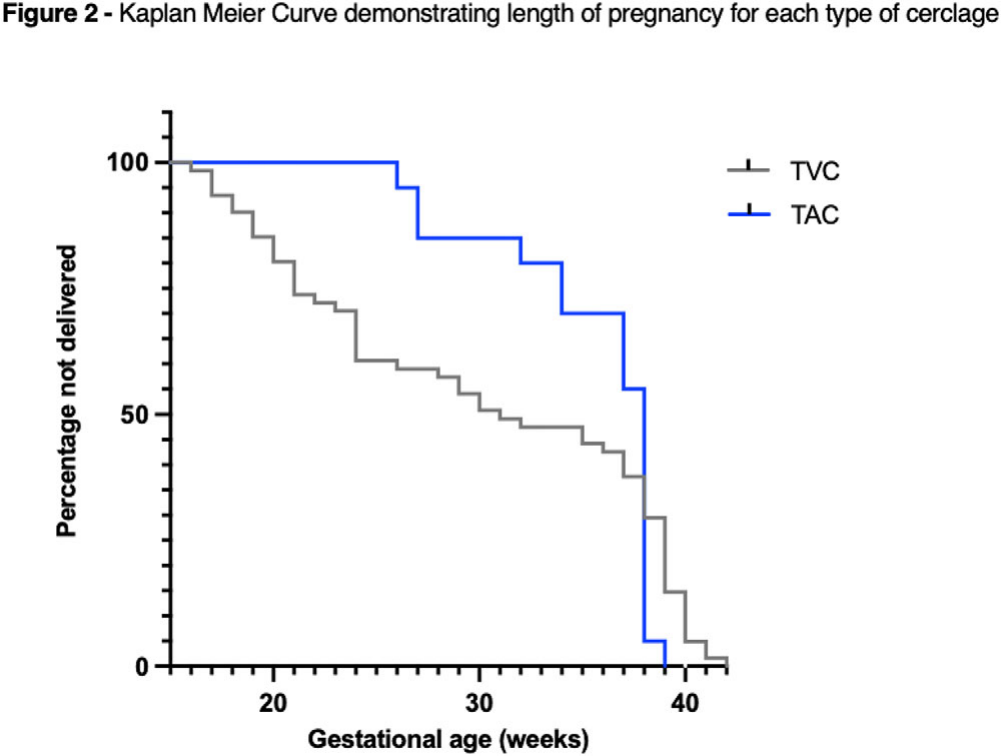

## 96 | Peripartum coronaviruses disease‐19 infection increases the risk for obstetric anal sphincter injuries

Alla Saban^1^, Noa Leybovitz Haleluya^2^, Reli Hershkovitz^3^, Yael Geva^3^, Lior Yahav^4^, Adi Y Y. Weintraub^5^

^1^Soroka University Medical Center, Beer Sheva, HaDarom; ^2^Soroka Medical Center, Meitar, HaDarom; ^3^Soroka medical center, Soroka medical center, HaDarom; ^4^Soroka Medical Center, Beer Sheva, HaDarom; ^5^Soroka University Medical Center, Soroka University Medical Center, HaDarom

12:15–13:30

**Objective**: The coronavirus disease 2019 (COVID‐19) has had a significant impact on healthcare systems around the world. The effect on obstetric outcomes is not yet completely understood, especially on obstetric anal sphincter injuries (OASIS) during childbirth. We aimed to investigate whether COVID‐19 infection during the peripartum period was associated with an increased risk for OASIS.

**Study Design**: A retrospective cohort study was conducted, including all singleton vaginal deliveries and cesarean deliveries due to failed vacuum extraction, between June 2020 to January 2022 at a large tertiary medical center. During the study period, COVID‐19 was universally screened by polymerase chain reaction (PCR) test from oral and nasal swab samples for all women before hospitalization. OASIS complication during childbirth was compered between women with and without peripartum diagnosis of COVID‐19, defined as a as a positive PCR test obtained within one week prior to delivery and up to three days after delivery. A logistic regression model was constructed to adjust for confounders.

**Results**: The study population included 22,911 women, among them 468 (2.0%) tested positive for COVID‐19, compared to 22,443 women without diagnosis of COVID‐19. After adjusting for confounding variables, peripartum infection with COVID‐19 was found to be independently associated with OASIS (adjusted OR 4.38, *p* < 0.001, CI 2.00‐9.61).

**Conclusion**: Infection with COVID‐19 during the peripartum period increases the risk for OASIS in more than fourfold. These finding emphasize the importance of understanding the impact of COVID‐19 on birth complications, such as OASIS, to improve public health measures and enhance obstetric outcomes during pandemics.

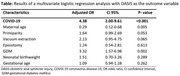

## 97 | Placenta accreta spectrum and fetal congenital anomalies in a Tertiary Center, Riyadh Saudi Arabia

Bahauddin I. Sallout^1^, Enas Wedad^2^, Eman Alsanie^3^, Mohammed Mira^4^

^1^King Fahad Medical city, King Fahad medical City, Ar Riyad; ^2^Maternal and Fetal Medicine, Women's specialized hospital, King Fahad medical city, Maternal and Fetal Medicine, Women's specialized hospital, King Fahad medical city, Ar Riyad; ^3^Maternal and Fetal Medicine, Women's specialized hospital, King Fahad medical city, Maternal and Fetal Medicine, Women's specialized hospital, King Fahad medical city, Ar Riyad; ^4^Maternal and Fetal Medicine, Women's specialized hospital, King Fahad medical city, Maternal and Fetal Medicine, Women's specialized hospital, King Fahad medical city, Ar Riyad

12:15–13:30

**Objective**: The incidence of placenta accreta spectrum is rising sharply, placenta accreta spectrum is frequently associated with severe maternal and fetal complications, including structural abnormalities, prematurity, and neonatal death. This study aimed to determine the relationship between placenta accreta spectrum and congenital abnormalities in King Fahad Medical City, Riyadh, Saudi Arabia.

**Study Design**: This is a retrospective study including all patient diagnosed as placenta accreta spectrum and managed in King Fahad Medical City, Riyadh, Saudi Arabia, over three years’ period, from Jan 2021 to Dec 2023.

**Results**: During the three years’ study period, we managed 132 pregnancies complicated with placenta accrete spectrum. and two pregnancies were twin gestation. We diagnosed 18 cases of fetal major congenital anomalies. The prevalence of fetal congenital anomalies in our study group was 13.6% in comparison global prevalence of 3%. The prevalence of IUGR was 15.9 % (21 out of 132 pregnancy). There are 4 cases of major congenital anomalies were terminated before 22 weeks of gestation. The majority of the patients (82.1%) delivered preterm, cesarean hysterectomy was performed for 108 patients (81.8%).

Four fetuses were stillbirth. 126 fetuses born alive; out of those, there were 6 cases of early NND and two infant deaths at 6 weeks and 10 weeks of life. 44 from the 126 life born (34.9%) were admitted to the normal newborn nursery, including two newborns with congenital anomalies; Cross fused renal ectopia and Polydactyly, the rest were normal. The remaining 82 (65.1%) required NICU admission, including 8 newborns with congenital anomalies; 4 with cardiac anomalies, 2 with omphalocele, one with holoprosencephaly, and one renal anomaly. Respiratory complications (RDS or TTN) were the most common during neonatal period affecting 48 newborns.

**Conclusion**: We found that there is a positive and significant association between placenta accrete spectrum and congenital malformations.

## 98 | Should we induce labor earlier in twin pregnancy complicated with hypertensive disoreders?

Yoav Siegler^1^, Naphtali Justman^2^, Gal Bachar^2^, Naama Farago^1^, Dana Vitner^2^, Zeev Weiner^3^, Ron Beloosesky^3^

^1^Rambam Health Care Center, Rambam Health Care Center, HaZafon; ^2^Rambam Medical Center, Haifa, Hefa; ^3^Rambam Medical Health Center, Haifa, Hefa

12:15–13:30

**Objective**: Preeclampsia (PE) is a major health concern affecting 2–8% of pregnancies, with twin pregnancies facing a 2–3 times higher risk compared to singletons. Existing obstetrics guidelines do not differentiate between singleton and twin pregnancies. Our study compares the antepartum and postpartum outcomes of preeclampsia in singleton and twin pregnancies, aiming to identify potential differences that should results in change of guidelines.

**Study Design**: A retrospective cohort case control study. We included pregnancies complicated with hypertensive disorders after 24 0/7 weeks of gestation delivered at a single tertiary care center between 2008‐2023. We compared charectraristics and outcomes of twin pregnancies wuth those of singleton pregnancies. Primary outcome was gestational age at the time of mild and severe PET. Secondary outcomes included clinical and laboratory charictaristics.

**Results**: A total of 403 twins and 3096 singleton pregnancies were included in the study. Both mild and severe PET appeared significantly earlier in twins compared to singletons (35.72 ± 2.1 vs. 38.12 ± 2.6 weeks, *p* < 0.001, and 35.53 ± 2.3 vs. 37.5 ± 3.2 weeks, *p* < 0.001, respectively). Rates of severe PET were significantly higher in twins compared to singletons (53.3% vs. 40.2%, *p* < 0.001). Both Hemoglobin and platlets levels were lower in twin pregnancies compared to singleton while aminotransferase level was higher in the twin group (9.54 ± 1.7 vs. 10.05 ± 1.5 gr/dL, *p* < 0.001; 156.2 ± 6 vs. 187.4 ± 6 10^3^/ul, *p* < 0.001 and 57.4 ± 88.6 vs. 46.7 ± 72.5 units/L respectively). The need for multiple antihypertensive agents treatment was significantly higher among twins compared to singletons (17.4% vs. 13.8%, *p* < 0.001). Postpartum hemorrhage rates was also higher in twin pregnancy group (31% vs. 17%, *p* < 0.001).

**Conclusion**: Twin pregnancies often exhibit hypertensive disorders at an earlier stage and with more severe symptoms. This finding highlights the need to reconsider existing clinical guidelines regarding the timing of labor induction for singleton versus twin pregnancies.

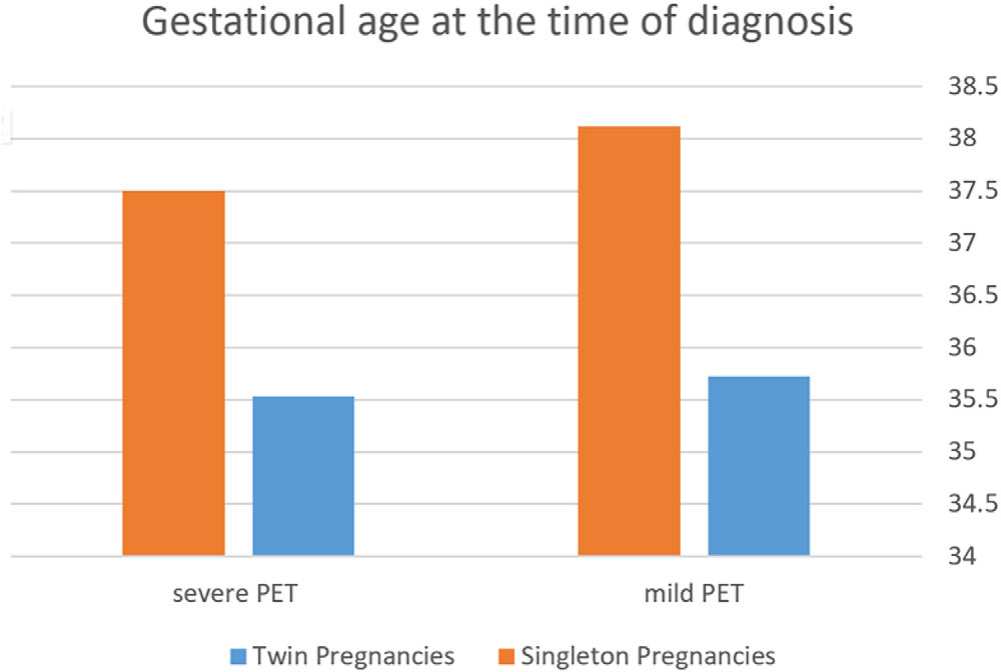

## 99 | Long term cardiovascular risk after pregnancy complicated by Systemic Lupus Erythematosus

Serena Simeone^1^, Alessandra Poli^2^, Caterina Serena^3^, Federico Toscano^2^, Chiara Biagiotti^2^, Paola Palazzo^2^, Silvia Vannuccini^4^, Marianna P. Rambaldi^3^, Serena Ottanelli^3^, Sara Zullino^3^, Laura Angeli^3^, Giacomo Bruscoli^3^, Calogero Lino Cirami^3^, Felice Petraglia^4^, Federico Mecacci^4^

^1^Careggi University Hospital, Careggi University Hospital, Toscana; ^2^University of Florence, Florence, Toscana; ^3^Careggi University Hospital, Florence, Toscana; ^4^Department of Biomedical, Experimental and Clinical Sciences ‘Mario Serio’, University of Florence, Florence, Toscana

12:15–13:30

**Objective**: Systemic Lupus Erythematosus (SLE) may significantly affect pregnancy. Preeclampsia and SGA/FGR, frequently observed in SLE pregnancies, are risk factors for cardiovascular disease (CVD) later in a woman's life. The aim of our study was to investigate the long term cardiovascular outcome in women with SLE who had experienced preeclampsia/FGR/SGA during pregnancy.

**Study Design**: Secondary analysis of a single center prospective cohort of pregnancies affected by SLE between 2007 and 2022. Years after pregnancy, we recalled the cohort included in an Ultrasonic Cardiac Output Monitor (USCOM) prospective project and collected data on the occurrence of CVD, thrombosis/stroke, fasting glucose, cholesterol, metabolic syndrome, tryglicerides and renal function (creatinine >1.20mg/dL and proteinuria). The association between these variables and the history of preeclampsia/FGR/SGA was analyzed both with ANOVA and linear regression analysis.

**Results**: A cohort of 110 participants was enrolled, 60 accepted to be recalled and 6 were excluded for incomplete dataset, resulting in a final cohort of 54 participants), between 13 and 2 years after pregnancy. CVD prevalence was 22.2%. Chronic hypertension affected 26% of women, presenting after pregnancy in 9 cases (pPH, 64%).

Linear regression showed a significant association between CVD vs FGR/SGA (18.5%, *p* = 0.025) and CVD vs post pregnancy hypertension, pPH (*p* = 0.001). pPH was also associated with preeclampsia (13%, *p* = 0.005), and CVD with the risk of thrombosis/stroke (13%, *p* = 0.001). Renal function, as abnormal serum creatinine, was associated with CVD in ANOVA (*p* = 0.03) but not in the regression analysis (*p* = 0.4).

**Conclusion**: SLE is a known risk factor for CVD later in life, but particularly in women who have experienced preeclampsia and FGR/SGA in pregnancy. Preeclampsia is associated with the development of hypertension in pregnancy and the consequent risk of CVD/thrombosis.

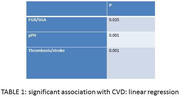

## 101 | Expanded targeted neonatal screening for congenital cytomegalovirus infection–is it needed?

Lital Brilant^1^, Micky Osovsky^2^, Joseph Pardo^3^, Jacob Amir^3^, Keren Tzadikevitch Geffen^4^

^1^Bar Ilan University, Bar Ilan University, HaZafon; ^2^Helen Schneider Hospital for Women, Rabin Medical Center, Helen Schneider Hospital for Women, Rabin Medical Center, Tel Aviv; ^3^Helen Schneider Hospital for Women, Rabin Medical Center, Petach Tikvah, Israe, Helen Schneider Hospital for Women, Rabin Medical Center, Petach Tikvah, Israe, Tel Aviv; ^4^Rabin Medical Center, Petach Tikva, HaMerkaz

12:15–13:30

**Objective**: Congenital Cytomegalovirus (cCMV) is the most common congenital infection, affects 0.2‐5% of all neonates worldwide. Only 10‐15% of nfants with cCMV are symptomatic at birth. Sensorineural hearing loss is the most frequent neurodevelopmental disability presented in cCMV, which might be present at birth or develop later in life. Asymptomatic infants will benefit from close follow up and early treatment if neurodevelopmental disabilities present later in life. Universal screening of infants for cCMV is not the common practice in most countries. The yield of targeted screening is being explored in recent studies. Our study aimed to explore the yield of targeted screening and assess the indications for targeted screening in addition to failed newborn hearing screen (FNHS), a well‐established indication for targeted screening for cCMV.

**Study Design**: Retrospective observational study at a tertiary center, from August 2018 to September 2022. Inclusion criteria were all newborns who underwent testing for cCMV infection.

**Results**: During the study period 36,958 neonates were born. Among them, 235 neonates were screened for cCMV. Positive cCMV based on urine PCR tests were detected in 112 infants, representing 0.3% of all births, which is within the predicted range of cCMV. One positive cCMV infant was excluded from the analysis due to missing data.

The highest prevalence of cCMV was found in neonates tested due to maternal infection 81% (95/117). Followed by failed newborn hearing screen (FNHS) 17% (7/41). And clinical or laboratory findings 13.8% (9/65).

Maternal infection was the indication for screening 85.5% (95/111) of the 111 positive neonates. Of these 95 positive for cCMV neonates, only 14 failed the newborn hearing screen test.

**Conclusion**: Expanded criteria for targeted screening including FNHS, maternal infection, and Clinical or laboratory finding is needed in order to improve early diagnosis of cCMV particularly in asymptomatic neonates.

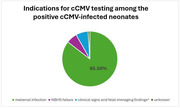

## 103 | Home, Sweet Home: An evaluation of the relationship between food environment and diabetes in pregnancy

Symone McClain^1^, Tiffany Clinton^1^, Alicia Speak^1^, Jessica Flint^2^, Allison Fly^3^, Tylar Louise Marie Dickson^4^, Briana Marin^5^, Miranda Goodson^6^, Sun Kwon Kim^1^, Raminder Khangura^1^, D'Angela S. Pitts^1^

^1^Henry Ford Health, Detroit, Michigan; ^2^Corewell Dearborn, Detroit, Michigan; ^3^Michigan State University College of Osteopathic Medicine, Lansing, Michigan; ^4^Michigan State University College of Medicine, Lansing, Michigan; ^5^Benedictine University, Chicago, Illinois; ^6^Wayne State Medical School, Detroit, Michigan

12:15–13:30

**Objective**: Access to healthy and nutritious food is a basic human right, although it has become a critical social determinant of health. Lack of healthy food access has been linked to poorer health outcomes and chronic preventable health conditions, especially in our most vulnerable populations. Previous research has suggested mixed results with food insecurity and type 2 diabetes (T2DM) and gestational diabetes (GDM). This study's objective is to explore the impact of living in a low food access environment (LAFE) on the incidence of T2DM and GDM in pregnant patients

**Study Design**: This study was a retrospective chart review from 2014‐2019 of patients with pregnancies complicated by T2DM and GDM who delivered a viable neonate. Patient census tracts were compared to the USDA Access Resource Atlas to identify patients who live in low food access neighborhoods. We defined a low access area as characterized by the number of housing units in the area without access to a vehicle that is more than 0.5 mile from a supermarket. Multivariable logistic regression models were used for data analysis.

**Results**: 3897 patients were included in this study with 1377 (35.3%) residing in a LAFE and 2520 (64.7%) lived in high access food environments. In our univariate comparison analysis for food environment status, 4.6% (*n* = 117) of patients in the high food access environment had T2DM, whereas 4.8% (*n* = 66) with T2DM resided in a LAFE (*p* = 0.832). There were 13.7% (*n* = 345) of patients in a high food access environment had GDM, whereas 11.3% (*n* = 155) in a LAFE (*p* = 0.030) had GDM. However, after controlling for confounding variables in the multivariate regression model, neither T2DM nor GDM were significant. Black race (OR 3.7), other race (OR 1.8), younger maternal age, and drug use (OR 1.6) were statistically significant predictors of a low food access group.

**Conclusion**: Our results suggest that T2DM and GDM are not significantly associated with residing in a low food access environment. More studies need to be done examining the role of other environmental and genetic factors that could predispose patients to these health conditions.

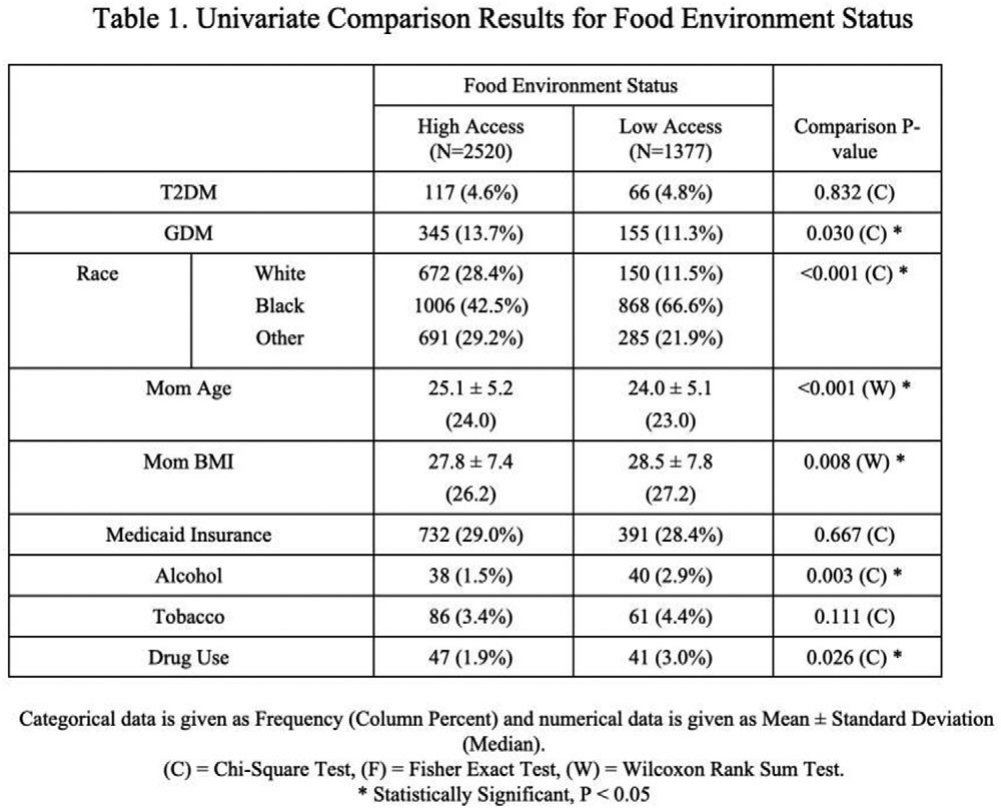

## 104 | The association between cervical cerclage and long‐term infectious morbidity of the offspring among twin pregnancies

Orit Wissotzky Broder^1^, Gali Pariente^2^, Tamar Wainstock^2^, Eyal Sheiner^2^

^1^Soroka Medical Center, beer sheva, HaDarom; ^2^Department of Obstetrics and Gynecology, Soroka University Medical Center, Ben‐Gurion University of the Negev, Beer‐Sheva, Israel., Beer Sheva, HaDarom

12:15–13:30

**Objective**: In Twin pregnancy, albeit the association with preterm birth, the use of cerclage remains controversial. Placing a cervical cerclage, beyond carrying a mechanical effect on the cervix, may have an effect on vaginal flora and microbiome, and thus may influence the plausibility for an infection of the offspring. We aimed to study the association between cervical cerclage and long‐term infectious morbidity of the offspring among twin pregnancies.

**Study Design**: A population‐based retrospective cohort study was conducted, comparing the long‐term infectious morbidity among twins, born after pregnancy with and without cervical cerclage, between the years 1991‐2021 in a tertiary medical center. The study groups were followed until 18 years of age for infectious morbidity. Data for the diagnosis of infectious morbidity of the offspring were extracted from community‐based clinics and hospitalization records. A Kaplan‐Meier survival curve was used to compare cumulative incidence rates, and a Cox proportional hazard model was used to adjust for confounders.

**Results**: The study included 11,986 twins of which 180 (1.5%) following placement of cervical cerclage. Although crude incidence of infectious morbidity was lower in offspring of pregnancies with a cervical cerclage (45.6% vs. 54.2%, *p* = 0.021), the cumulative incidence of long‐term infectious morbidity was comparable in offspring with and without cerclage (Kaplan‐Meier survival curve, Log‐Rank *p* = 0.640; Figure). Likewise, In the Cox proportional hazard model, adjusted for gestational age at birth, cervical cerclage was not found as a risk factor for long‐term infectious morbidity among twin pregnancies (adjusted HR 0.92, 95% CI 0.74‐1.14, *p* = 0.476).

**Conclusion**: Cervical cerclage placement in twin pregnancies is not associated with an increased risk of long‐term infectious morbidity of the offspring.

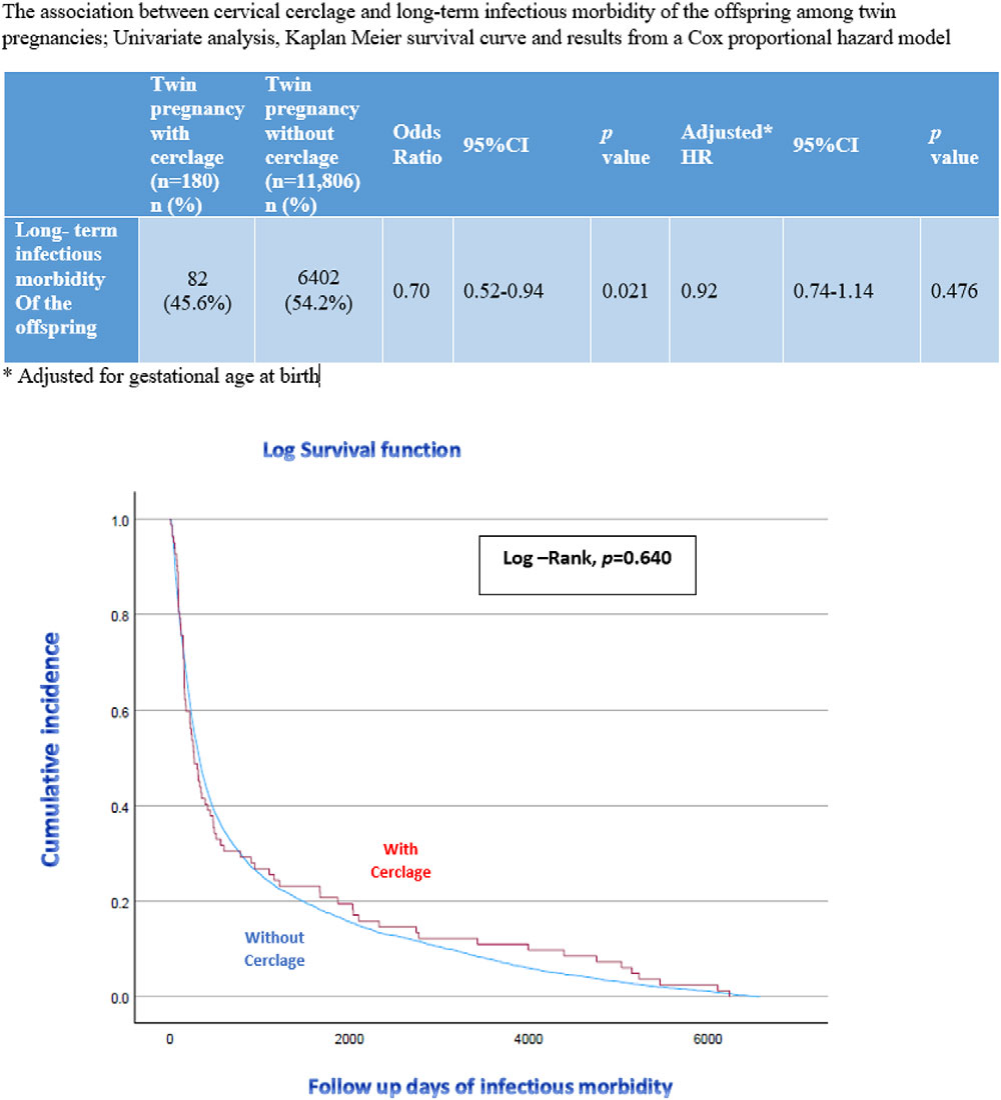

## 105 | Development and validation of an ultrasound‐based estimated fetal weight reference for chinese twin pregnancy

Jing Yang^1^, Yangyu zhao^2^

^1^Department of Obstetrics and Gynecology, Peking University Third Hospital, BeiJing, Beijing; ^2^Obstetrics & Gynecology Department, Peking University Third Hospital, Beijing, Beijing

12:15–13:30

**Objective**: To develop and validate a chorionicity‐specific growth chart of ultrasound estimated fetal weight (EFW) for Chinese twin pregnancies.

**Study Design**: This retrospective cohort study included all twin pregnancies who delivered two live fetuses with gestational age ≥34 weeks. The participants were divided into a development cohort (delivered before December 2017) and a validation cohort (delivered after January 2018). The chorionicity‐specific growth charts were created using a three‐level mixed model based on the development cohort. The fetuses from the validation cohort were classified into three groups: fetal growth restriction (FGR) indicated by both the newly established twin charts and the Hadlock singleton chart currently used for twin pregnancies in China, suspected FGR indicated by only the singleton chart, and no FGR indicated by either chart. The incidence of neonatal outcomes among the three groups was then compared accordingly.

**Results**: This retrospective cohort study included all twin pregnancies who delivered two live fetuses. The participants were divided into a development cohort (delivered before December 2017) and a validation cohort (delivered after January 2018). The chorionicity‐specific growth charts were created using a three‐level mixed model based on the development cohort. The fetuses from the validation cohort were classified into three groups: fetal growth restriction (FGR) indicated by both the newly established twin charts and the Hadlock singleton chart currently used for twin pregnancies in China, suspected FGR indicated by only the singleton chart, and no FGR indicated by either chart. The incidence of neonatal outcomes among the three groups was then compared accordingly.

**Conclusion**: The fetal growth trajectories differed by chorionicity, with a lower EFW for MCDA twins beginning at 28 weeks. The establishment of chorionicity‐specific growth reference could reduce overdiagnosis of FGR and improve fetal growth monitoring of twin pregnancies.

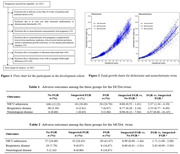

## 106 | Characteristics of cochrane reviews in the pregnancy and childbirth group: A comprehensive analysis

Emily Zitkovsky^1^, Silas Monje^2^, Julia Sroda Agudogo^3^, Anna R. Whelan^4^, Stephen M. Wagner^3^, Suneet P. Chauhan^5^, Megha Gupta^6^

^1^Beth Israel Deaconess Medical Center, Providence, RI; ^2^Warren Alpert Medical School Brown University, Providence, Rhode Island; ^3^Beth Israel Deaconess Medical Center, Boston, Massachusetts; ^4^University of Massachusetts Chan School of Medicine, Grafton, Massachusetts; ^5^Delaware Center of Maternal‐Fetal Medicine at Christiana Care, Delaware, Delaware; ^6^Beth Israel Deaconess Medical Center, Boston, MA

12:15–13:30

**Objective**: The Cochrane Pregnancy and Childbirth Group (CPCG) is one of the largest review groups within the Cochrane Library. This study aims to analyze the characteristics of Cochrane reviews published by the CPCG and their incorporation into guidelines from the American College (ACOG), the Royal College (RCOG), and the Royal Australian and New Zealand College of Obstetricians and Gynaecologists (RANZCOG).

**Study Design**: We conducted a cross‐sectional study of all Cochrane reviews published by the CPCG as of February 1, 2023. Data were abstracted from the Cochrane Library database for each review, including the year of publication, number of studies, total patients included, type of intervention, and timing of intervention. We also assessed the incorporation of these reviews into guidelines from ACOG, RCOG, and RANZCOG.

**Results**: Of 654 Cochrane reviews under CPCG, 4 were withdrawn, and 85 had no studies included, leaving 565 Cochrane reviews for final analysis. The most common categories of reviews were medical complications during pregnancy (14.0%), preterm labor (8.3%), and care during childbirth (8.0%). The majority (71.2%) of reviews were published between 2010 and 2019, included 1‐10 studies (63.0%), and involved both US and international studies (54.1%). The number of patients in the reviews varied, with 38.1% including less than 1,000 patients. Interventions were most frequently conducted during the antepartum period (46.0%), and 98.2% of reviews focused on interventions, with drugs being the most common type (41.1%). More than half of the reviews (53.3%) reported both maternal and fetal/neonatal primary outcomes. The citation of Cochrane reviews in guidelines varied significantly: ACOG (25.5%), RCOG (20.0%), and RANZCOG (9.4%; *p* < 0.001; Table 1).

**Conclusion**: This analysis describes the focus of CPCG reviews and their variable incorporation into national guidelines. The significant differences in citation of Cochrane among the three organizations highlight the need for increased transparency and organizations to better inform clinical practice and guide future research.

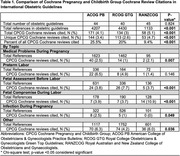

## 107 | The impact of the indication for emergency cervical cerclage on pregnancy outcomes

Chen manor^1^, Ettie Piura^1^, Yair Daykan^1^, Zvi Klein^1^, Tal Biron‐Shental^2^, Michal Kovo^1^, Nissim Arbib^1^

^1^Meir Medical Center, kfar saba, HaMerkaz; ^2^Meir Medical Center, Meir Medical Center, HaMerkaz

12:15–13:30

**Objective**: Emergency cervical cerclage (ECC) is placed in cases of ultrasound‐indicated cerclage (USIC) and physical examination‐indicated cerclage (PEIC) to reduce the risk of preterm birth (PTB). Despite reported success rates ranging from 60% to 80%, with an average prolongation of pregnancy by 4 weeks, disparities in pregnancy outcomes between these indications remain unclear. This study aims to elucidate such differences.

**Study Design**: This retrospective cohort study investigates singleton pregnancies that underwent ECC using the McDonald's technique between 18 and 23.6 weeks of gestation within the period of 2017‐2023 at a single tertiary healthcare center. The cohort was stratified based on ECC indication: USIC and PEIC. The comparative analysis includes rates of PTB < 34 weeks, time intervals between cerclage and birth, and overall pregnancy outcomes.

**Results**: Maternal characteristics, maternal age, gravidity, and parity did not differ between the groups. The USIC group (*n* = 32) exhibited a significantly longer cervical length pre‐procedure compared to the PEIC group (*n* = 12): 13.5 ± 7.9 mm vs. 6.8 ± 4.8 mm, respectively, *p* = 0.008. Prior hysteroscopy was more prevalent in the PEIC group (*p* = 0.049). Gestational age (GA) at the time of the procedure and prolongation of pregnancy were similar between the USIC and PEIC groups: 20.5 ± 1.9 weeks vs. 21.2 ± 1.8 weeks, respectively, *p* = 0.329; 14.8 ± 4.2 weeks vs. 13.3 ± 6.2 weeks, respectively, *p* = 0.391. The majority (81.8%) delivered after 34 weeks, No significant differences were observed in gestational week at delivery between the groups (*p* = 0.99).

**Conclusion**: Emergency cervical cerclage effectively extends pregnancy duration and reduces the risk of early PTB. However, the indication for ECC appears to have no discernible impact on pregnancy outcomes.

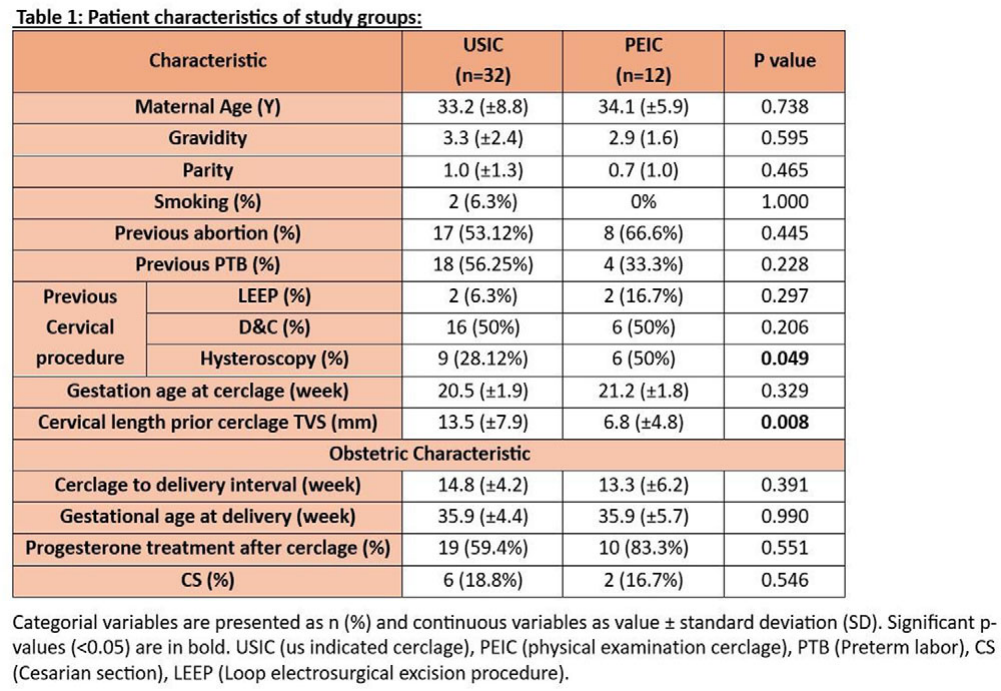

## 108 | Electrocardiogram utility for chest pain in pregnancy in a contemporary united states cohort

Nathan A. Keller^1^, Frank I. Jackson^1^, Insaf Kouba^2^, Matthew J. Blitz^1^, Luis A. Bracero^1^

^1^Northwell, New Hyde Park, New York; ^2^Northwell, St. Petersburg, Florida

12:15–13:30

**Objective**: To determine if electrocardiograms (EKGs) performed for chest pain during pregnancy are more likely to yield abnormal results than those performed for routine screening.

**Study Design**: Retrospective cohort study of EKGs performed during pregnancy within a large healthcare system in New York, United States, between January 2018 and December 2022. EKG indications were determined by ICD‐10 codes. Heart disease screening was defined as any EKG performed for a non‐acute indication and was used as the reference group. Odds ratios of abnormal EKGs were calculated for each indication. Statistical analyses were performed in R 4.3.1.

**Results**: A total of 21,357 EKGs were performed and 6,233 (29.2%) had abnormal results. Chest pain was reported in 10.5%. The most common remaining indications were routine screening (66.6%) and tachycardia (11.5%). EKGs performed for chest pain were no more likely to be abnormal than those performed for routine screening. EKG indications of heart failure, cardiac arrhythmia, tachycardia, and other all had significantly higher odds of an abnormal EKG result. An EKG indication of syncope and collapse had a lower odds of an abnormal result than the reference group. The indications, percentages, and odds ratios are shown in the Table.

**Conclusion**: The diagnostic value of EKGs performed for the indication of maternal chest pain remains uncertain. This may be a potential opportunity to reduce unnecessary testing and healthcare costs during pregnancy.

* Abnormal rhythm includes any of the following: premature ventricular contraction, premature atrial contraction, right bundle branch block, left bundle branch block, long QT, or atrial fibrillation

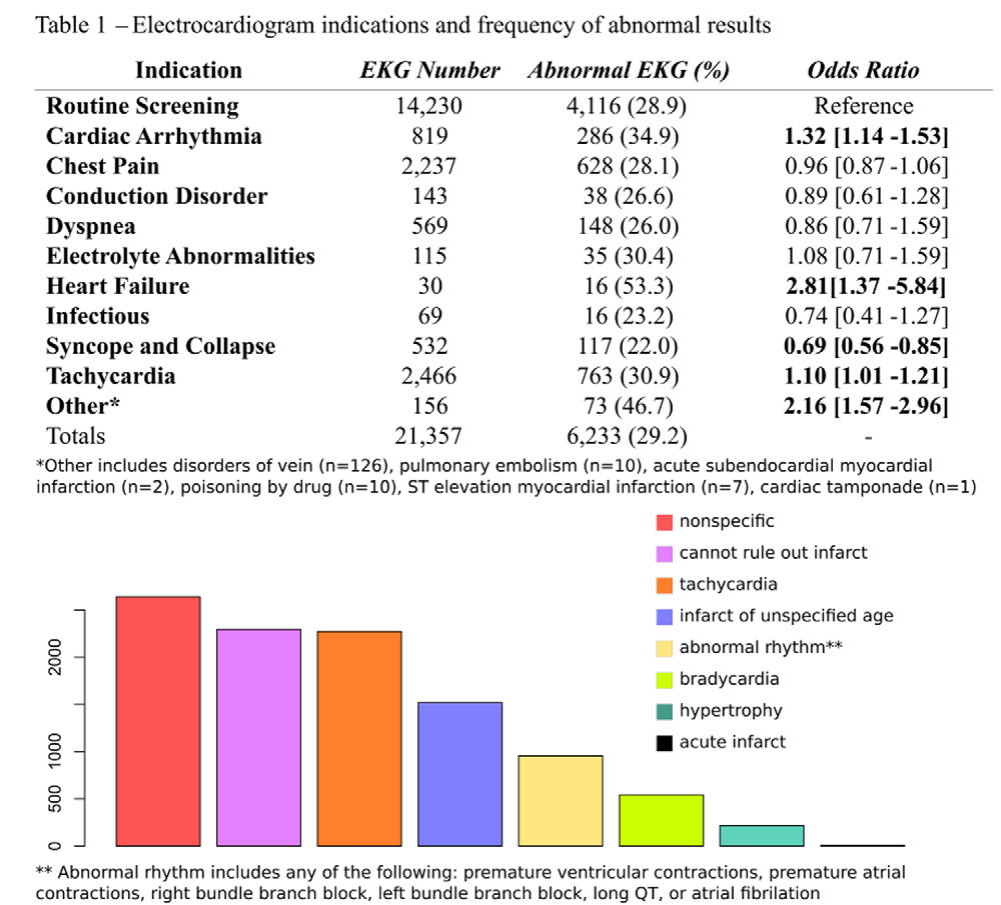

## 109 | Adverse pregnancy outcomes in resolved early fetal growth restriction

Frank I. Jackson^1^, Nathan A. Keller^1^, Insaf Kouba^2^, Matthew J. Blitz^1^, Luis A. Bracero^1^

^1^Northwell, New Hyde Park, New York; ^2^Northwell, St. Petersburg, Florida

12:15–13:30

**Objective**: To determine whether fetal growth restriction (FGR) diagnosed at the time of comprehensive fetal anatomy ultrasound which later resolves during the 3rd trimester is associated with increased odds of adverse pregnancy outcomes compared to pregnancies with normal fetal growth throughout gestation.

**Study Design**: Retrospective cohort study of all patients with both a second and third trimester ultrasound who delivered at seven hospitals within a large healthcare system in New York, United States between 2018 and 2023. FGR was defined as an estimated fetal weight or abdominal circumference < 10th percentile for gestational age. FGR diagnosed before 24 weeks persisting to greater than 28 weeks was considered persistent FGR and those cases which did not persist were considered resolved FGR. Multivariable logistic regression was used to compare the rate of preterm birth ( < 37 weeks), NICU admission, and neonatal acidosis (umbilical artery pH < 7.20) among patients with resolved FGR to those who did not have FGR. All statistical analyses were performed in R 4.3.1.

**Results**: In total 45,297 pregnancies were included: 39,856 (88%) without FGR, 4,706 (10.4%) with FGR diagnosed after 28 weeks, and 735 (1.6%) diagnosed with FGR before 24 weeks. Among FGR pregnancies diagnosed < 24 weeks, 405 (55.1%) had persistent FGR and 330 (44.9%) had resolution of FGR after 28 weeks. Of the 330 pregnancies with resolved FGR there was a significantly increased risk of PTB and NICU admission but not neonatal acidemia compared to pregnancies with no FGR (Table). FGR diagnosed after 28 weeks and persistent FGR diagnosed before 24 weeks were associated with a PTB rate of 14.2% and 33.8% and a NICU admission rate of 25.0% and 48.1% respectively.

**Conclusion**: Pregnancies complicated by a diagnosis of FGR in the second trimester that resolves during the 3rd trimester are at increased risk of PTB and NICU admission compared to pregnancies with normal fetal growth throughout gestation.

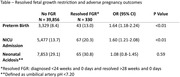

## 110 | Adverse pregnancy outcomes in resolved large for gestational age fetuses

Nathan A. Keller^1^, Frank I. Jackson^1^, Insaf Kouba^2^, Matthew J. Blitz^1^, Luis A. Bracero^1^

^1^Northwell, New Hyde Park, New York; ^2^Northwell, St. Petersburg, Florida

12:15–13:30

**Objective**: To determine whether large for gestational age (LGA) fetuses diagnosed at less than 24 weeks of gestation with consequent resolution have increased adverse pregnancy outcomes.

**Study Design**: Retrospective cohort study of all patients with both a second and third trimester ultrasound delivering within a large healthcare system in New York, United States between January 2018 and December 2023. LGA was defined as an estimated fetal weight (EFW) > 90% for gestational age. LGA diagnosed before 24 weeks and 0 days persisting to greater than 28 weeks and 0 days was considered persistent LGA and those not persisting as resolved LGA. LGA diagnosed after 28 weeks and 0 days was classified as late LGA. Multivariable logistic regression was used to compare rates of preterm birth ( < 37 weeks), NICU admission, and neonatal acidosis among patients with resolved LGA, persisting LGA, late LGA, and appropriate for gestational age (AGA) fetuses. All statistical analyses were performed in R 4.3.1.

**Results**: In total 45,297 pregnancies were reviewed with 39,666 (87.6%) AGA and 5,631 (12.4%) diagnosed with LGA during pregnancy. LGA was diagnosed after 28 weeks in 2,658 (5.8%) of patients and before 24 weeks in 2,973 (6.6%) of patients. Among those diagnosed before 24 weeks 1,998 (67.2%) resolved and 975 (32.8%) persisted after 28 weeks. Pregnancies with early LGA with consequent resolution experienced a significantly higher rate of preterm birth (12.1%) than AGA, late diagnosed LGA, and persistent early LGA (Table 1). The rates of NICU admission and neonatal acidosis did not differ between groups.

**Conclusion**: Fetuses with an LGA diagnosis before 24 weeks of gestation with subsequent resolution after 28 weeks experienced a significantly increased rate of preterm delivery. Such pregnancies may encompass fetuses with an inherently higher baseline growth potential with impaired growth or placental dysfunction later in gestation.

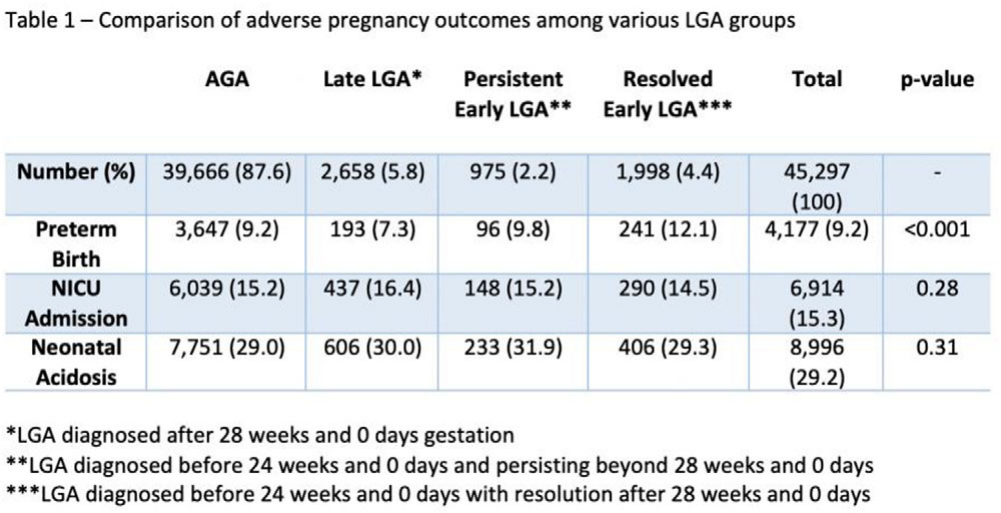

## 111 | Complications resulting from gestational diabetes screening in patients with history of bariatric surgery

Frank I. Jackson^1^, Nathan A. Keller^1^, Sarika Arora^1^, Alexis Palmer^1^, Heather L. Tier^2^, Insaf Kouba^3^, Jolene C. Muscat^2^, Matthew J. Blitz^1^, Luis A. Bracero^1^

^1^Northwell, New Hyde Park, New York; ^2^Northwell Health, Bay Shore, New York; ^3^Northwell, St. Petersburg, Florida

12:15–13:30

**Objective**: To assess complications resulting from gestational diabetes (GDM) screening in patients with a history of bariatric surgery.

**Study Design**: Retrospective cohort of patients with a history bariatric surgery who received prenatal care within a large healthcare system in New York, United States between January 2019 and June 2023. Patients were grouped based on surgical procedure into the following categories: Gastric Sleeve, Gastric Band, Roux‐en‐Y Gastric Bypass, and Biliopancreatic Diversion with Duodenal Switch (BPDDS). Manual chart review was performed to obtain patient clinical characteristics, surgical history, GDM screening methods, and related complications, which were classified into the following categories: hypoglycemia, nausea and vomiting, and inability to tolerate (incomplete test results). All statistical analyses were performed in R 4.3.1.

**Results**: A total of 322 patients were included for analysis: 214 (66%) had an oral glucose test (50g, 75g, or 100g) in a subsequent pregnancy. In our cohort, 242 (75.2%) had a sleeve gastrectomy, 53 (16.5%) had a Roux‐en‐Y gastric bypass, 23 (7.1%) had a gastric band, and 4 (1.2%) had a BPDDS. In total, complications were identified in 26% of pregnancies in which an oral glucose test was performed. Patients with Roux‐en‐Y gastric bypass experienced the highest rate of complications (42%), followed by gastric sleeve (27%), and gastric band (5%). One BPDDS patient had oral glucose testing and experienced a complication. The most common complication experienced in all groups was hypoglycemia ( < 65 mg/dL), which occurred in 17% of patients. Patients with gastric sleeves who had oral glucose testing experienced an inability to tolerate the drink in 11% and nausea and vomiting in 5% of cases.

**Conclusion**: We observed a high rate of complications after oral glucose testing was performed during pregnancy following both malabsorptive and restrictive bariatric surgery procedures. It may be advisable to discontinue the practice of oral glucose testing in patients with a history of bariatric surgery.

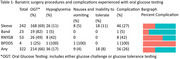

## 112 | Sleep apnoea among third trimester gravidae by maternal sleep position

Kathleen M. Antony^1^, Jordan McIntyre^1^, Shania Kumar^1^, John M.D. Thompson^1^, Sonja Woodall^1^, Hanna Fontinha^1^, Wendy Burgess^1^, Robin Cronin^1^, Andrew Veale^2^, Edwin A. Mitchell^1^, Peter R. Stone^1^

^1^University of Auckland, Auckland, Auckland; ^2^New Zealand Respiratory and Sleep Institute, Auckland, Auckland

12:15–13:30

**Objective**: The objective of this analysis was to evaluate the apnoea‐hypopnea index by maternal sleep position during the third trimester.

**Study Design**: This was a secondary analysis of a prospective observational study. The primary study examined the physiology of maternal sleep and sleep position in the third trimester. Participants had level 2 PSG tests at their own home using the Embletta MPR PT with ST+Proxy ambulatory unit. Sleep position was registered by the PSG device, validated by an accelerometer scored by three investigators with consensus meetings when blinded assessments did not align. Sleep tests were scored by sleep specialists. The apnoea‐hypopnea index (AHI) was calculated as the total apnoeas plus hypopnoeas per hour of sleep. OSA was defined as present if the AHI was 5 or greater, or absent if the AHI was < 5. Sleep position was characterized as left, supine, right, or unknown.

**Results**: 160 gravidae completed PSGs. Of the 134 participants with reliable sleep position information, 64 (47.8%) had OSA. The predominant amount of sleep time was spent in a lateral position. The median AHI for supine position was 5 (interquartile range (IQR) 2.3‐9.3); and was significantly higher than left lateral position 2.8 (IQR 1.1‐5.4), (*p* < 0.001); and right lateral position 3.4 (IQR 1.5‐5.9)(*p* < 0.001). The AHI in the right and left lateral positions was not statistically significantly different. Participants with OSA spent 17.6% (IQR .05‐41%) of the night in left lateral position compared to 32.2% (IQR 8.6‐47.8%) of the night in left lateral for gravidae who did not meet criteria for OSA (*p* = 0.05).

**Conclusion**: Supine sleep position was associated with a higher AHI than non‐supine. Supine sleep position has been associated with adverse pregnancy outcomes. These findings offer a potential biological explanation, but larger studies are needed.

## 113 | Sleep apnoea among a convenience sample of third trimester pregnant people in Auckland, Aotearoa/New Zealand

Kathleen M. Antony^1^, Jordan McIntyre^1^, Shania Kumar^1^, John M.D. Thompson^1^, Hanna Fontinha^1^, Sonja Woodall^1^, Robin Cronin^1^, Wendy Burgess^1^, Edwin A. Mitchell^1^, Andrew Veale^2^, Peter R. Stone^1^

^1^University of Auckland, Auckland, Auckland; ^2^New Zealand Respiratory and Sleep Institute, Auckland, Auckland

12:15–13:30

**Objective**: To estimate the prevalence of obstructive sleep apnoea (OSA) among a population of third trimester gravidae undergoing polysomnography(PSG).

**Study Design**: Secondary analysis of a prospective observational study. Primary study examined the physiology of maternal sleep and sleep position in the third trimester among 5 groups of participants: 1. “normal” (no medical or obstetric comorbidities, normal body mass index (BMI)), 2.having obesity (BMI > = 30), 3.small for gestational age or growth restricted fetus (SGA), 4.reduced fetal movement (RFM), or 5.symptoms of supine hypotension (SH). Participants had level 2 PSG tests at their own home. Tests were scored by sleep specialists. OSA was defined as present if the apnoea‐hypopnea index (AHI) was 5 or greater, or absent if the AHI was < 5. OSA severity was defined as mild, moderate, or severe as per international criteria. OSA diagnosis was evaluated overall, by the predesignated groups, and by ethnicity.

**Results**: 160 gravidae completed PSGs, of which 153 were usable. PSGs were performed between 33+6 and 38+5 weeks gestation. The overall prevalence of OSA was 45.8% (70/153). The majority of OSA was mild, present in 38.6% (59/153) of participants. Moderate and severe OSA was present in 5.9% (9/153) and 1.3% (2/153) of participants, respectively. 23 gravidae were of indigenous Māori or of Pasifika descent and the prevalence of OSA was 52.2%. Among group 1, the prevalence of OSA was 45.2% (14/31). The prevalence of OSA was 60% (18/30), 41.9% (13/31), 34.2% (13/38), and 52.2% (12/23) for groups 2, 3, 4, and 5, respectively. The prevalence of OSA did not differ statistically by group or ethnicity in this sample.

**Conclusion**: The prevalence of OSA in this sample of third trimester gravidae was high, even among participants without comorbidities (45%). These findings are novel in Aotearoa/New Zealand. This study uniquely includes participants of Māori or Pasifika descent, who are not represented in many other countries. Overall, the sample size was small for analysis by ethnicity; additional studies and studies at different time points during pregnancy are indicated.

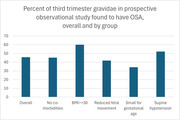

## 114 | Sleep quality and architecture in the third trimester of pregnancy

Kathleen M. Antony^1^, Jordan McIntyre^1^, Shania Kumar^1^, John M.D. Thompson^1^, Sonja Woodall^1^, Hanna Fontinha^1^, Robin Cronin^1^, Wendy Burgess^1^, Andrew Veale^2^, Edwin A. Mitchell^1^, Peter R. Stone^1^

^1^University of Auckland, Auckland, Auckland; ^2^New Zealand Respiratory and Sleep Institute, Auckland, Auckland

12:15–13:30

**Objective**: The objective of this analysis was to describe sleep quality and architecture in the third trimester of pregnancy.

**Study Design**: This was a secondary analysis of a prospective observational study. The primary study examined the physiology of maternal sleep and sleep position in the third trimester in 5 groups of participants: 1. “normal” (no medical or obstetric comorbidities, normal body mass index (BMI)), 2. having obesity (BMI > = 30), 3. small for gestational age or growth restricted fetus (SGA), 4. reduced fetal movement (RFM), or 5. symptoms of supine hypotension (SH). Participants had level 2 PSG tests at their own home.

**Results**: 160 people were enrolled. The average sleep period across all gravidae was 464 mins (7.7 hours) and was similar between groups; mean 449 mins in the RFM group to 481 mins in the SHP group; no statistically significant differences were found. Sleep latency was shortest in the “normal” group (median of 11 minutes, range 11‐20 minutes) and was found to be significantly longer in the group with obesity (*p* = 0.01) and the group with RFM (*p* = 0.02) compared to “normal”. The total minutes actually asleep ranged from 349 to 381 minutes. Sleep efficiency in all groups was also similar ranging from 78.2% to 81.9%.

The sleep architecture was similar across all groups and was dominated by N2 (40.6%). A notable proportion of time was spent awake (19.7%). REM sleep was 21% of total sleep time, and 17% of the total sleep period. The median time to first REM was 88 minutes. The length of first period of REM was 8 mins (IQR 3‐ 14) and subsequent periods of REM were of similar length 8 min (IQR 4‐16). The period of time between REM periods was 34 mins (IQR 9‐ 86). The average lengths of time in sleep states were short with 4.9 mins in N2, 6.1 minutes in N3 and 11.3 mins in REM, with a mean of 109 sleep state changes over the period from sleep onset to waking.

**Conclusion**: Here, we describe the sleep patterns and architecture in the third trimester of pregnancy. Slight sleep disturbances were noted, even in pregnancies unaffected by obesity or obstetric complications.

## 115 | Implementation and performance of first‐trimester referral ultrasound following theintroduction of the guidelines of SIEOG

Grazia Volpe^1^, Grazia Volpe^1^, Laura Sarno^2^, Elena mantovani^3^, Daniele Di Mascio^4^, Valeria D'ambrosio^5^, Tiziana Fanelli^6^, Ilaria Fantasia^6^, Giampiero Minnella^7^, Paola Quaresima^8^, Enrico Corno^9^, Andrea Dall'asta^10^

^1^Fondazione IRCCS Ca Granda, Ospedale Maggiore Policlinico, Fondazione IRCCS Ca Granda, Ospedale Maggiore Policlinico, Lombardia; ^2^Università degli Studi di Napoli Federico II, Rome, Lazio; ^3^Department of Obstetrics and Gynecology, Azienda Ospedaliera Universitaria Integrata Verona, Department of Obstetrics and Gynecology, Azienda Ospedaliera Universitaria Integrata Verona, Veneto; ^4^Università Roma La Sapienza, Roma, Lazio; ^5^Department of Maternal and Child Health and Urological Sciences, Umberto I Hospital, Sapienza, Department of Maternal and Child Health and Urological Sciences, Umberto I Hospital, Sapienza, Lazio; ^6^Fetal Medicine Unit, “Di Venere” Hospital, Bari, Puglia; ^7^Azienda Ospedaliera Universitaria Policlinico Paolo Giaccone, Azienda Ospedaliera Universitaria Policlinico Paolo Giaccone, Sicilia; ^8^Department of Obstetrics and Gynaecology, Magna Graecia University of Catanzaro, Department of Obstetrics and Gynaecology, Magna Graecia University of Catanzaro, Calabria; ^9^University of Parma, University of Parma, Emilia‐Romagna; ^10^Department of Medicine and Surgery, Obstetrics and Gynecology Unit, University of Parma, Department of Medicine and Surgery, Obstetrics and Gynecology Unit, University of Parma, Emilia‐Romagna

12:15–13:30

**Objective**: To evaluate the implementation of first trimester expert referral ultrasound (US) and its accuracy in discriminating between anomalous and non‐anomalous fetuses compared with referral ultrasound performed in the second trimester.

**Study Design**: Multicenter, retrospective study of prospectively collected data conducted in nine referral Fetal Medicine units in Italy including singleton pregnancies identified at risk for structural anomaly following first trimester screening US. The definition of “risk for structural anomaly” was based on either nuchal translucency ≥3.5 mm or one or more fetal structural anomaly suspected at first trimester screening US. Only cases undergoing referral US with a standardized protocol at one of the participating centers between 11 +0 and 13 +6 weeks of gestation were included in the study. The reference to evaluate the performance of first trimester US was represented by second trimester referral US.

**Results**: 344 cases were referred between 11 +0 and 13 +6 weeks at the participating centers over the study period and 322 (93.6%) had referral sonographic assessment within 13 +6 weeks of gestation. After excluding cases undergoing miscarriage and termination of pregnancy, 136 cases having referral US at 11 +0 −13 +6 and at 19‐21 weeks and 207 cases having referral US at 11 +0 −13 +6 and at 14‐16 weeks were included. Overall, the agreement per anatomical regions between 11 +0 −13 +6 weeks’ and 19‐21 weeks’ referral sonographic assessment was 85.3% (116/136) and the sensitivity, specificity, PPV and NPV were 82.0%, 88.0%, 84.7% and 85.7%, respectively. The agreement between 11 +0 −13 +6 and 14‐16 weeks referral US was 91.3% (189/207) and sensitivity, specificity, PPV and NPV were 90.7%, 92.1%, 93.9% and 88.2%, respectively.

**Conclusion**: Our data suggest excellent implementation of the SIEOG guidelines with respect to first‐trimester referral ultrasound. The expert assessment of fetal anatomy before 14 weeks is feasible when adopting a standardized protocol and allows an early diagnosis in most cases at risk for fetal anomaly following first trimester screening ultrasound.

## 116 | Repeat PlGF testing in women with suspected pre‐eclampsia: A cost‐effectiveness analysis of the PARROT‐2 trial

Alice Hurrell^1^, Lucy C. Chappell^2^, Rachael Hunter^3^

^1^King's College London, King's College London, England; ^2^King's College London, London, United Kingdom; ^3^University College London, London, England

12:15–13:30

**Objective**: To evaluate cost‐effectiveness of repeat placental growth factor (PlGF)‐based testing for suspected pre‐eclampsia, compared with usual care.

**Study Design**: This was a within trial cost‐effectiveness analysis, in 22 maternity units in England, Scotland, and Wales, participating in the PARROT‐2 trial of repeat revealed PlGF‐based testing, compared to usual care (ISRCTN85912420, 25/11/2019), including women receiving an initial revealed PlGF‐based test as part of clinical management of suspected preterm pre‐eclampsia. This was a health economic evaluation, describing health resource use and associated cost per woman, infant, and maternal‐infant dyad, according to randomised allocation to repeat revealed PlGF‐based testing or usual care with repeat concealed testing. Additional analysis was stratified according to initial PlGF‐based test result (normal, abnormal, or very abnormal).

**Results**: Costs per woman, infant, or maternal‐infant dyad were similar between the repeat revealed testing group and the group receiving usual care with repeat concealed testing. In the revealed group compared to the usual care group, there were significantly greater costs associated with neonatal admissions for special care (mean difference £1,169 95% CI 398‐1,939), but not neonatal intensive care or high‐dependency care. Costs correlated with initial PlGF‐based test result, with highest costs per mother and infant dyad in the group with a very abnormal initial test result (£38,284 SD 35,997) and lowest costs in the group with a normal initial test result (£13,156 SD 9,568).

**Conclusion**: There is no evidence of an overall significant difference in cost associated with a policy of universal, routine repeat testing, compared to usual care, in women receiving an initial PlGF‐based test for suspected pre‐eclampsia.

## 117 | Correlation between maternal BMI and duration of second stage of labor: A prospective monocentric study

Andrea Dall'asta^1^, Stefania Fieni^2^, Ariane Kiener^3^, Biancamaria Mastrandrea^3^, Gabriella Maria Celora^3^, Alissa Valenti^3^, Sara Sorrentino^3^, Giovanni Morganelli^2^, Letizia Galli^2^, Valentina Anna Degennaro^2^, Elvira Di Pasquo^2^, Beatrice Valentini^4^, Tullio Ghi^5^

^1^Department of Medicine and Surgery, Obstetrics and Gynecology Unit, University of Parma, Department of Medicine and Surgery, Obstetrics and Gynecology Unit, University of Parma, Emilia‐Romagna; ^2^Azienda Ospedaliero‐Universitaria di Parma, Parma, Emilia‐Romagna; ^3^University of Parma, Parma, Emilia‐Romagna; ^4^University of Parma, University of Parma, Emilia‐Romagna; ^5^Dipartimento di Medicina e Chirurgia, Università di Parma, Parma, Emilia‐Romagna

12:15–13:30

**Objective**: To investigate the relationship between maternal body mass index (BMI) recorded in early pregnancy and the duration of the second stage of labor in an unselected cohort of women achieving spontaneous vaginal delivery

**Study Design**: Retrospective analysis of data prospectively collected over a 12‐month period at a single referral tertiary maternity unit In Italy. Singleton pregnancies with cephalic presenting fetus at term gestation were eligible for the purposes of the study. The exclusion criteria were represented by maternal age < 18 years, delivery prior to 37+0 weeks of gestation, twin gestation and obstetric intervention for all indications. The correlation between early pregnancy BMI and second stage duration was investigated, furthermore the mean duration of the second stage was compared across the different BMI categories.

**Results**: Overall, 2555 deliveries were recorded, and 2605 fetuses were delivered over the study period. 438 cesarean deliveries and 166 preterm deliveries were recorded, respectively, and there were 119 instrumental deliveries leaving 1782 cases for data analysis. Of them, 196 (11.0 %) patients had BMI >30 kg/m^2^; 68 (3.8 %) had BMI >35 kg/m^2^; 20 (1.1%) had BMI > 40 kg/m^2^. A negative correlation between BMI and second stage duration was observed, and a longer second stage duration was recorded in obese compared to non‐obese women (33 ± 51 vs 49 ± 60 minutes, *p* < 0.01). Such association was retained after adjusting for potential confounders (*p* = 0.02). Shorter second stage duration was also recorded when comparing women with BMI above vs below 35 kg/m^2^(30 ± 50 vs 48 ± 59 minutes, *p* = 0.13). Overall, a shorter second stage duration was observed across the different BMI categories (*p* < 0.01) and when considering the different degrees of obesity (*p* < 0.01).

**Conclusion**: The findings from our large single‐center study demonstrate shorted duration of the second stage of labor with increasing BMI, and particularly a shorter mean duration in obese compared to non‐obese

## 118 | Maternal and neonatal outcomes in twin pregnancies in patients with history of prior cesarean section

Ariana Spiegel^1^, Julie Gomez^2^, Kavisha Khanuja^1^, Rodney A. McLaren, Jr., Jr.^1^

^1^Thomas Jefferson University Hospital, Philadelphia, Pennsylvania; ^2^Thomas Jefferson University, Philadelphia, PA

12:15–13:30

**Objective**: ACOG states that twins with one prior cesarean delivery, who are otherwise candidates for trial of labor may be offered trial of labor after cesarean delivery (TOLAC). The objective of this was to evaluate adverse maternal and neonatal outcomes between twin TOLAC and twin repeat cesarean without TOLAC in a US modern population cohort.

**Study Design**: A retrospective study using National Vital Statistics System birth data from 2015–2022 with twin births > 28 weeks gestation of patients who had twin pregnancy and a history of one cesarean delivery. Those who had a nonvertex twin A birth were excluded. Adverse maternal and neonatal outcomes were compared between twin births that underwent trial of labor after cesarean delivery and those who had a repeat cesarean without a trial. Univariable and multivariable analyses were performed.

**Results**: There were 39,034 births that met inclusion criteria from 2015 to 2022. Regarding adverse maternal outcomes, repeat cesarean delivery without trial of labor did not differ in uterine rupture, maternal transfusion, and unplanned hysterectomy compared to those who had a TOLAC, but were more likely to require ICU admission (Table 1). Regarding adverse neonatal outcomes, immediate assisted ventilation use, and at >6 hours, occurred more frequently in twins who had a repeat cesarean delivery without TOLAC than those who had a TOLAC, however those who had a TOLAC had a higher risk of 5‐minute APGAR score < 3. (Table 1)

**Conclusion**: Twin TOLAC did not differ in several adverse maternal and neonatal outcomes in the US compared to a repeat cesarean delivery without TOLAC.

## 119 | Association between breastfeeding and neurodevelopment at age 6 in the French PELAGIE Birth Cohort

Marion MONPERRUS^1^, Rémi BERANGER^2^, Ronan GARLANTEZEC^3^, Maela LE LOUS^3^

^1^University Hospital Rennes, CHU Rennes, Bretagne; ^2^University Hospital Rennes, University Hospital Rennes, Bretagne; ^3^University Hospital, University Hospital Rennes, Bretagne

12:15–13:30

**Objective**: Breastfeeding has been shown to be associated with improved child cognitive performance, but the causality of this association is still debated, as it tends to disappear when accounting for maternal cognitive performance and socio‐economic status. We aimed to explore the relationship between breastfeeding and cognitive performance of children in the French in a French general population.

**Study Design**: From the PELAGIE mother‐child cohort, which included pregnant women between 2002 and 2006 in Brittany (France), 286 children were evaluated using the Wechsler Intelligence Scale for Children (WISC‐IV) and the Developmental NEuroPSYchological Assessment (NEPSY) scales at age 6. Associations between breastfeeding and cognitive performance were assessed using multivariable linear regression models adjusted for maternal verbal cognitive performance and education level, familial stimulation and environment (HOME scale), and the Rey's social deprivation index (contextual indicator). In addition, we performed Structural Equation Modeling (SEM) to investigate the complex interrelation between these variables.

**Results**: The WISC Verbal Comprehension Index (WISC‐VCI) was significantly higher for children breastfed for at least four months than that for those never breastfed or breastfed < 15 days (β_adjusted_: +4.95 points, 95% CI: 0.54, 9.37). Among the 193 breastfed children, the duration of breastfeeding, in particularl during the first four months, was increasingly associated with the WISC‐VCI score (per log10 increase, in month, *n* = 193: β_adjusted_ = 8.01 [3.31, 12.71]). We also observed statistically significant associations between breastfeeding or the duration of breastfeeding and better performance on several NEPSY subsets, including visual attention, design copying, arrows and narrative memory. SEM analysis confirmed these associations. No statistical association was observed with the WISC Working Memory Index or other NEPSY subtests.

**Conclusion**: These data support current national and WHO recommendations for Europe promoting breastfeeding for at least four months.

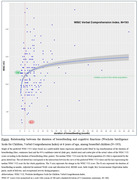

## 121 | Doula care: Help or hindrance? Perinatal outcomes in over 99,000 deliveries

Ella Segal^1^, Adi Ashkenazi Katz^2^, Lior Kashani Ligumsky^3^, Ariel Many^4^, Miriam Lopian^5^

^1^Sheba Medical Center, Tel Aviv University, HaMerkaz; ^2^Tel Aviv University, Tel Aviv University, Tel Aviv; ^3^University of California, Los Angeles, Los Angeles, California; ^4^Department of Obstetrics and Gynecology, Mayanei Hayeshua Medical Center, bnei brak, HaMerkaz; ^5^Mayanei Hayeshua Medical Center, Mayanei Hayeshua Medical Center, Tel Aviv

12:15–13:30

**Objective**: Doula care has become increasingly recognized as an effective strategy for improving maternal health equity. This study aims to extend the investigation of Doula care's impact by evaluating its effects on a broader demographic, including patients of higher socioeconomic status.

**Study Design**: We conducted a retrospective cohort study at a single medical center between 2012‐2023. Outcomes of pregnant women receiving Doula care were compared to those that didn't. Doula care was self‐funded and included > three prenatal visits and support during labor, delivery and the postpartum. Patients with a previous cesarean delivery (CD), planned CD, non‐vertex presentation, multiple pregnancy and known fetal abnormalities were excluded. The primary outcome was intrapartum cesarean delivery. Secondary outcomes included delivery characteristics and composite adverse neonatal outcome (CANO), comprised of umbilical artery pH < 7.1, Apgar score < 7 at 5 minutes, neonatal intensive care unit (NICU) admission, respiratory and infectious morbidity, and hypoglycemia.

**Results**: Perinatal outcomes of 856 women receiving Doula care were compared to 98, 833 women who didn't. Patients receiving Doula care were more likely to be nuliparous (25.6% vs 21.6% *p* = 0.002) undergo induction of labor (26.4% vs 20.1% *p* < 0.001), receive epidural analgesia (77.9% vs 55.8%, *p* < 0.001) and augmentation of labor (29.4% vs 18.7% *p* < 0.001). Multivariate logistic regression analysis was performed to adjust for confounders. Patients receiving Doula care were not at increased risk of CD (5% vs 4.6% adjusted Odds Ratio (aOR) 1.1, 0.8‐1.5, *p* = 0.3), postpartum hemorrhage, blood transfusion or CANO (16.9% vs 15.3% aOR 1.1, 0.9‐1.4, *p* = 0.1) but at increased risk of operative vaginal delivery (12.7% vs 7.3%, aOR, 1.4, 1.1‐1.7, *p* = 0.002) perineal tears (37.5% vs 29.9% aOR 1.4, 1.2‐1.6 *p* < 0.0) and postnatal readmission (1.4% vs 0.7% aOR 2.0, 1.1‐3.5 *p* = 0.009).

**Conclusion**: In non‐deprived populations, doula care may offer limited benefits and might be associated with an increased risk of obstetric intervention without an improvement in perinatal outcomes.

## 122 | Does epidural analgesia increase the risk of emergency delivery for fetal compromise in small‐for‐gestational‐age neonates?

Adi Ashkenazi Katz^1^, Ella Segal^2^, Lior Kashani Ligumsky^3^, Ariel Many^4^, Miriam Lopian^5^

^1^Tel Aviv University, Tel Aviv University, Tel Aviv; ^2^Sheba Medical Center, Tel Aviv University, HaMerkaz; ^3^University of California, Los Angeles, Los Angeles, California; ^4^Department of Obstetrics and Gynecology, Mayanei Hayeshua Medical Center, bnei brak, HaMerkaz; ^5^Mayanei Hayeshua Medical Center, Mayanei Hayeshua Medical Center, Tel Aviv

12:15–13:30

**Objective**: To assess the impact of intrapartum epidural analgesia (EA) on the risk of emergency operative delivery for suspected fetal compromise in small‐for‐gestational age (SGA) neonates.

**Study Design**: A retrospective cohort study was conducted at a single medical center between 2013‐2023. The study group was comprised of patients who delivered SGA neonates (birthweight (BW) < 10^th^ centile). Outcomes were compared between women that did and did not receive intrapartum EA. Patients with BW > 10^th^ centile, previous cesarean delivery (CD), planned CD, multiple gestation, fetal death or abnormalities were excluded. The primary outcome was operative delivery for suspected fetal compromise. Secondary outcomes included CD for other indications, composite adverse maternal outcomes (CAMO) defined as postpartum hemorrhage, blood product transfusion, and postpartum readmission and composite adverse neonatal outcomes (CANO), defined as APGAR < 7 at 5 mins, umbilical artery pH < 7.1 and neonatal intensive care unit (NICU) admission. Multivariate logistic regression analysis was used to adjust for potential confounders.

**Results**: Outcomes of 1,833 (57.3%) women receiving EA were compared to 1,363 (42.6%) women who did not. There were no clinically significant between‐group differences in maternal age, gestational age at delivery or neonatal birthweight (2550g vs 2545g). Patients receiving EA were more likely to be nulliparous (50% vs. 24.6% *p* < 0.001), undergo induction of labor (43.2% vs 24.6%) and have hypertensive disorders (5.3% vs 2.0%, *p* < 0.001). After adjusting for confounders, intrapartum EA use significantly increased the risk of emergency operative delivery for suspected fetal compromise (20.3% vs 8.8% adjusted odds ratio (aOR) 1.7, 1.3‐2.1, *p* < 0.001) but not CD for other indications (6.3 vs 5.2% aOR 0.8, 0.6‐1.1, *p* = 0.2) CAMO (1.2% vs. 0.9 % aOR 1.1, 0.5‐2.3 *p* = 0.8) or CANO (6.7% vs 7.5 aOR 1.0, 0.7‐1.5, *p* = 0.8).

**Conclusion**: Epidural analgesia increases the risk of emeregency operative delivery for suspected fetal compromise in SGA neonates, but not maternal and neonatal adverse outcomes or CD for other indications.

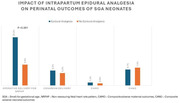

## 123 | Does intrapartum epidural analgesia increase the risk of emergency operative delivery for suspected fetal compromise?

Miriam Lopian^1^, Elad Miron^2^, Ella Segal^3^, Adi Ashkenazi Katz^2^, Lior Kashani Ligumsky^4^, Ariel Many^5^

^1^Mayanei Hayeshua Medical Center, Mayanei Hayeshua Medical Center, Tel Aviv; ^2^Tel Aviv University, Tel Aviv University, Tel Aviv; ^3^Sheba Medical Center, Tel Aviv University, HaMerkaz; ^4^University of California, Los Angeles, Los Angeles, California; ^5^Department of Obstetrics and Gynecology, Mayanei Hayeshua Medical Center, bnei brak, HaMerkaz

12:15–13:30

**Objective**: To assess the impact of intrapartum epidural analgesia (EA) on the risk of operative delivery for suspected fetal compromise.

**Study Design**: A retrospective cohort study was conducted at a single university‐affiliated medical center between 2013‐2023. The study group was comprised of patients who received EA during labor. Outcomes were compared to those who did not. Patients with a previous cesarean delivery (CD), planned CD, non‐vertex presentation, multiple gestation, intrauterine fetal death and fetal abnormalities were excluded. The primary outcome was emergency operative delivery for suspected fetal compromise. Secondary outcomes included CD for other indications, composite adverse maternal outcomes (CAMO) defined as postpartum hemorrhage, blood product transfusion, and postpartum readmission and composite adverse neonatal outcomes (CANO), defined as APGAR < 7 at 5 mins, umbilical artery pH < 7.1 and neonatal intensive care unit (NICU) admission. Multivariate logistic regression analysis was used to adjust for potential confounders.

**Results**: Outcomes of 50,667 (56.9%) women who received EA were compared to 38,405 (43.1%) women who did not receive EA. There were no clinically significant between‐group differences in maternal age, gestational age at delivery or neonatal birthweight. Patients who received EA were more likely to be nulliparous (32.5% vs. 11.0% *p* < 0.001) and undergo induction of labor (26.8% vs 13.5%, *p* < 0.001) than controls. After adjusting for confounders, patients receiving EA had an increased risk of emergency operative delivery for suspected fetal compromise (12.4% vs 8.6% adjusted odds ratio (aOR) 2.3, 2.1‐2.4, *p* < 0.001), CAMO (2.2% vs. 1.4% aOR 1.3, 1.1‐1.4, *p* < 0.001) and CANO (2.4% vs 1.9 aOR 1.2, 1.2‐1.4, *p* < 0.001) but no difference in the risk of intrapartum cesarean delivery for other indications (2.8% vs 2.3% aOR 1.03, 0.9‐1.2, *p* = 0.6).

**Conclusion**: Intrapartum epidural analgesia is associated with an increased risk of operative delivery for non‐reassuring fetal heart rate patterns as well as maternal and neonatal adverse outcomes.

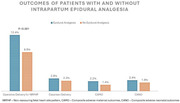

## 124 | Does epidural analgesia increase the risk of emergency delivery for fetal compromise in a TOLAC?

Ella Segal^1^, Adi Ashkenazi Katz^2^, Lior Kashani Ligumsky^3^, Ariel Many^4^, Miriam Lopian^5^

^1^Sheba Medical Center, Tel Aviv University, HaMerkaz; ^2^Tel Aviv University, Tel Aviv University, Tel Aviv; ^3^University of California, Los Angeles, Los Angeles, California; ^4^Department of Obstetrics and Gynecology, Mayanei Hayeshua Medical Center, bnei brak, HaMerkaz; ^5^Mayanei Hayeshua Medical Center, Mayanei Hayeshua Medical Center, Tel Aviv

12:15–13:30

**Objective**: To assess the impact of intrapartum epidural analgesia (EA) on the risk of emergency delivery for suspected fetal compromise in patients undergoing a trial of labor after caesarean (TOLAC).

**Study Design**: A retrospective cohort study was conducted at a single medical center between 2013‐2023. The study group comprised patients undergoing TOLAC. Outcomes were compared between women who did and did not receive EA in labor. Patients with contraindications to TOLAC, planned ccesarean delivery (CD), multiple gestation, intrauterine fetal death or fetal abnormalities were excluded. The primary outcome was emergency delivery for suspected fetal compromise. Secondary outcomes included uterine rupture, composite adverse maternal outcomes (CAMO) defined as uterine rupture, postpartum hemorrhage, blood product transfusion, and postpartum readmission and composite adverse neonatal outcomes (CANO), defined as APGAR < 7 at 5 mins, umbilical artery pH < 7.1 and neonatal intensive care unit (NICU) admission. Multivariate logistic regression analysis was used to adjust for potential confounders.

**Results**: Outcomes of 3, 600 (57.9%) women undergoing TOLAC who received EA were compared to 2623 (42.2%) women who did not receive EA. There were no clinically significant between‐group differences in maternal age, gestational age at delivery, hypertensive disorders of pregnancy or neonatal birthweight. Patients who received EA were less likely to have had a previous vaginal delivery (79% vs 91% < 0.001) and more likely to undergo inductionof labor (23% vs. 11.5% *p* < 0.001). After adjusting for confounders, intrapartum EA increased the risk of emergency delivery for suspected fetal compromise (16.0% vs 6.7% adjusted odds ratio (aOR) 1.9,1.6‐2.3, *p* < 0.001) but not the risk of uterine rupture (0.4% vs 0.2%, aOR 1.8, 0.7‐4.6, *p* = 0.25) CAMO (2.8% vs 2.0% aOR 1.2, 0.8‐1.6, 0.4) or CANO (3.1% vs 2.4% aOR 1.4 1.0‐1.9, *p* = 0.07).

**Conclusion**: Epidural analgesia use during a TOLAC is associated with an increased risk of emergency delivery for non‐reassuring fetal heart rate patterns but not maternal or neonatal adverse outcomes.

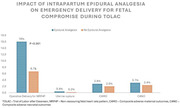

## 125 | When a screening test becomes diagnostic — Predicting one abnormal OGTT value using GCT cut‐off

Lee Reicher^1^, Guy Lutsker^2^, Elad Barkan^2^, Emmanuel Attali^3^, Daniel Gabbai^3^, Michal Rosenberg‐Friedman^4^, Yariv Yogev^5^, Sharon Maslovitz^6^

^1^Weizmann Instutute Of Science, Weizmann Institute of Science, HaMerkaz; ^2^Weizmann Instutute Of Science, Rehovot, HaMerkaz; ^3^Lis Hospital for Women's Health, Tel Aviv Sourasky Medical Center, Tel‐Aviv, Tel Aviv; ^4^ichilov, ichilov, Tel Aviv; ^5^Lis Maternity Hospital, Sourasky Medical Center, Tel Aviv University, Tel Aviv Sourasky Medical Center, Tel Aviv; ^6^tel aviv medical center, tel aviv, Tel Aviv

12:15–13:30

**Objective**: The 50 g‐glucose challenge test (GCT) is a widely used screening method for gestational diabetes (GDM). If elevated (>130‐140), an oral glucose tolerance test (OGTT) is performed to confirm the diagnosis. Traditionally, GDM has been diagnosed by GCT > 200 mg/dL or by two high values on the OGTT. However, a recent meta analysis has shown that individuals with only one abnormal value on the OGTT face similar risks as those with two abnormal values and should be managed and treated as such. Hence, the GCT threshold that obviates OGTT should be lowered. We assessed the positive predictive values (PPV) for one abnormal OGTT value at different cut‐off points of GCT and used a machine learning algorithm to predict the occurrence of one abnormal value on OGTT.

**Study Design**: Retrospective cohort study was conducted during 2017‐2022 in a tertiary center. The study group included women with GCT of >140 mg/dl who underwent OGTT. The PPV of GCT for one abnormal value on OGTT (Carpenter and Coustan criteria) was assessed using different GCT cut‐off values. Additionally, we fitted a regression model that predicts one abnormal value on OGTT based on GCT and maternal characteristics.

**Results**:

1. Our study consisted of 440 women (age 30.2 ± 7.4) with elevated GCT (159 ± 11.78) who underwent OGTT (median of one abnormal value).
2. At a GCT cut‐off of 157 and 170 mg/dl, the PPV of GDM was 90% and 100%, respectively **(Figure 1)**.
3. We trained a Gradient Boosting classifier predicting one abnormal OGTT value based on GCT, first trimester fasting glucose, gestational weight gain, BMI and maternal obstetric history parameters. The model had an area under the curve of 0.894 (95% confidence interval, 0.891‐0.899)

**Conclusion**: Our study highlights the potential for improved screening and diagnosis of GDM by considering lower cut‐off values for GCT that obviate OGTT thus sparing this inconvenient test. Moreso, these findings may contribute to more efficient and accurate identification of pregnant women at risk of developing GDM, allowing for early intervention and optimizing the diagnosis to glucose control interval.

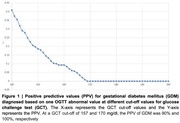

## 126 | Pravalip in unexplained recurrent pregnancy loss: Impact on pregnancy outcomes and implications for further research

Lee Reicher^1^, Emmanuel Attali^2^, Daniel Gabbai^2^, Michal Rosenberg‐Friedman^3^, Yariv Yogev^4^, Sharon Maslovitz^5^

^1^Weizmann Instutute Of Science, Weizmann Institute of Science, HaMerkaz; ^2^Lis Hospital for Women's Health, Tel Aviv Sourasky Medical Center, Tel‐Aviv, Tel Aviv; ^3^ichilov, ichilov, Tel Aviv; ^4^Lis Maternity Hospital, Sourasky Medical Center, Tel Aviv University, Tel Aviv Sourasky Medical Center, Tel Aviv; ^5^tel aviv medical center, tel aviv, Tel Aviv

12:15–13:30

**Objective**: Unexplained recurrent pregnancy loss (RPL) are treated empirically with a combination of progesterone, micropirin and LMWH (triple therapy, TT) with little evidence of efficacy. Pravastatin has a potentially beneficial effect on angiogenesis through enhancement of placental vascularity by improving the ratio of s‐flt/PLGF in several previous studies. Here, we will describe pregnancy outcomes among women with RPL who failed under TT and were treated with Pravastatin in the subsequent pregnancy.

**Study Design**: Women with at least 4 previous consecutive miscarriages (between 5‐10 weeks with documented gestational sac) who visited the RPL clinic prior to conception and initiated Pravalip 20 mg once daily during the current pregnancy (not later than 6w6d) included in this retrospective study. The women had at least two previous pregnancy failures under TT.

To rule out any other pathology, the workup prior to pregnancy included: Hysteroscopy and 3‐D ultrasound, parental karyotyping, diabetes (fasting glucose, HBA1C), thyroid malfunction (TSH), APLA (LAC, ACL, B2GP) and inherited thrombophilia (FVL, PROTHROMBIN, AT3, PROTEIN S, PROTEIN C).

**Results**:

1. Our study consisted of 58 women with median of 5 previous miscarriage with median of 3 previous unsuccessful pregnancies under TT
2. The mean maternal age was 33.8 ± 5 and BMI 28.1 ± 4
3. Previous placental complications: 43 women had no previous complications, 8 had previous IUGR, 4 had PET and 4 had IUGR and PET
4. Pravastatin treatment was initiated in 17 women after 5 weeks, 35 women after 6 weeks, and 6 women after 7 weeks
5. Current pregnancy outcomes: 4 had early miscarriage and 49 had term delivery. The gestational age at delivery was 38 ± 1.1 weeks, fetal weight was 2772.8 ± 314 gr.
6. Complications: we observed 7 SGA, 1 PET and 8 GDM.
7. There was one fetus with SUA, one with VSD and one with ARSA.

**Conclusion**: The findings suggest that Pravastatin could positively impact pregnancy outcomes, as a notable number of women achieved term deliveries.

## 127 | Personalized machine learning model for predicting time to delivery in active labor

Lee Reicher^1^, Emmanuel Attali^2^, Sharon Napadenski^3^, Nadav Rappoport^3^, Yariv Yogev^4^

^1^Weizmann Instutute Of Science, Weizmann Institute of Science, HaMerkaz; ^2^Lis Hospital for Women's Health, Tel Aviv Sourasky Medical Center, Tel‐Aviv, Tel Aviv; ^3^Ben‐Gurion University of the Negev, beer sheva, HaDarom; ^4^Lis Maternity Hospital, Sourasky Medical Center, Tel Aviv University, Tel Aviv Sourasky Medical Center, Tel Aviv

12:15–13:30

**Objective**: Prolonged labor has significant associations with an increased rate of operative delivery and adverse maternal and neonatal outcomes. To optimize resource management, physicians should assess and predict the course of labor. To address this issue, machine learning methods have been applied in numerous medical fields to develop personalized prediction models. Our aim was to develop a personalized model using real‐time data to predict time to delivery in the active phase of labor.

**Study Design**: Labor records from a tertiary hospital were searched and labeled over a 10‐year period. We trained logistic regression models using both “static features” (e.g demographics) and “dynamic features” (e.g cervical dilatation, efficacy, and newly derived features calculated as ratios between adjusted dynamic measures and time differences) to predict time to delivery (min). To assess the explainability of the model, we employed SHAP (SHapley Additive exPlanations) analysis.

**Results**:

1. A total of 94,386 cases in which a trial of labor was attempted were identified. Of them ∼70,000 admitted to the active phase of labor (6 cm dilatation).
2. Histogram‐Based Gradient Boosting regressor trained on the dataset with the demographic, static, dynamic, and aggregative features achieved higher results in terms of overall MAE and R2 metrics than a baseline of Lasso linear regression model: The model scores were mean absolute error of 62.64, and R2 0.51.
3. Next, we present a residual box plot **(Figure 1.)** focusing on the time range between 0‐120 minutes to labor.
4. Explainability of the best model was analyzed using SHAP. The features that contributed the most for the model prediction were: current cervical opening rate, maximal cervical opening rate and clinical fetal weight estimation.

**Conclusion**: Our personalized machine learning model holds promise in predicting time to delivery during active labor, aiding physicians in making informed decisions and potentially improving outcomes.

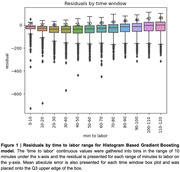

## 128 | Molecular and physiological profile of women with a history of Gestational Diabetes Mellitus

Lee Reicher^1^, Nastya Godneva^2^, Michal Rein^3^, Adina Weinberger^2^, Eran Segal^2^, Yariv Yogev^4^

^1^Weizmann Instutute Of Science, Weizmann Institute of Science, HaMerkaz; ^2^Weizmann Institute of Science, Rehovot, HaMerkaz; ^3^Weizmann Instutute Of Science, Rehovot, HaMerkaz; ^4^Lis Maternity Hospital, Sourasky Medical Center, Tel Aviv University, Tel Aviv Sourasky Medical Center, Tel Aviv

12:15–13:30

**Objective**: Previous studies showed that gestational diabetes mellitus (GDM) can be a risk factor for subsequent cardiovascular and metabolic diseases. However, there is a paucity of information regarding diverse cardiovascular, metabolic and multi‐omic measurements in elderly women after GDM. In the current study, we examined the molecular and physiological profile of women with a history of GDM.

**Study Design**: Data collected as part of large‐scale, prospective, longitudinal study with more than 10,000 healthy adults aged 40–70 years, between the years 2019 and 2022. The participants underwent physiological assessments through special medical tests and wearables, molecular and multi‐omic profiling, along with the collection of medical records and lifestyle questionnaires **(Figure 1)**. We included women who reported at least one live birth and compared the profiles of women with a history of GDM to control subset, matched by the number of pregnancies and the time elapsed since the GDM pregnancy.

**Results**:

1. Our study consisted of 300 and 800 women in GDM and the control group, respectively. Women with a history of GDM displayed notable findings compared to the control group:
2. Higher sonographic liver attenuation, which is indicative of fatty liver.
3. Higher cholesterol, triglycerides and ALT and lower HDL
4. Higher values across 20 glucose control and variability indexes of continuous glucose monitoring including the Continuous Overall Net Glycemic Action that measures intra‐day glycemic variability
5. Body composition obtained by a DXA scan showed higher visceral fat percentage and higher waist circumference
6. Sleep monitoring indicated a higher rate of sleep apnea (Oxygen Desaturation Index)
7. Microbiome: lower bacterial richness (α‐diversity) and higher relative abundance of Fusobacterium and Firmicutes were higher

**Conclusion**: GDM has lasting effects on multiple physiological systems relating to metabolic syndrome. The insights provided can assist in implementing essential long‐term monitoring and targeted interventions to mitigate the increased risk of cardiovascular and metabolic diseases associated with GDM.

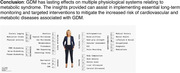

## 129 | Cost‐effectiveness of exome sequencing and CMA in pregnancy with major central nervous system structural anomalies

Michal Rosenberg‐Friedman^1^, Sharon Maslovitz^2^, Moshe Leshno^3^, Yariv Yogev^4^, Lee Reicher^5^

^1^ichilov, ichilov, Tel Aviv; ^2^tel aviv medical center, tel aviv, Tel Aviv; ^3^Tel Aviv university, TAU, Tel Aviv; ^4^Lis Maternity Hospital, Sourasky Medical Center, Tel Aviv University, Tel Aviv Sourasky Medical Center, Tel Aviv; ^5^Weizmann Instutute Of Science, Weizmann Institute of Science, HaMerkaz

12:15–13:30

**Objective**: The central nervous system (CNS) is affected in 9% of fetal malformations but accounts for as much as one‐third of all pregnancy terminations due to fetal anomaly. When applying Chromosomal microarray (CMA) to the diagnosis of fetal CNS anomalies, the estimated diagnostic yield remains low.demonstrated that Recent study in fetuses with major CNS anomalies, Exome sequencing (ES) has an additional diagnostic yield of 44% over the 10% provided by CMA only. We aimed to determine the cost‐effectiveness of ES in major CNS anomalies.

**Study Design**: We modeled costs, utility and quality adjusted life years (QALYs) for CMA versus CMA+ES in prenatal testing. Two strategies were compared using Markovian process decision analysis model: (1) CMA only, abnormal result culminating in termination of pregnancy (TOP) (2) ES after a negative CMA, for positive result a TOP is conducted. The outcomes included health quality of life after TOP or having a disabled child, the cost of CMA test, ES test and the health expenses of a disabled child. The time horizon of the model was 20 years. Sensitivity analysis including probabilistic sensitivity analysis of all parameters was conducted.

**Results**: In the base model the cost was $91,059 for CMA test and the average health expansion for disable child and $3,101 for CMA+ES. The QALY with time horizon of 20 years were 11.84 and 13.84 for CMA and CMA+ES strategies respectively, thus the strategy CMA+ES dominates the strategy CMA alone, higher QALY value and cost saving. Sensitivity analysis revealed that the results are robust and for all sensitivity analysis CMA+ES dominated the CMA with cost saving.

**Conclusion**: We suggest that Exome sequencing has the potential to be cost‐effective and should be considered as a first‐tier test in the prenatal diagnosis of major CNS anomalies. The substantial increase in diagnostic yield offered by ES not only provides valuable information for the current pregnancy but also holds significant relevance for future pregnancies. These findings are pivotal in facilitating timely and evidence‐based decision‐making.

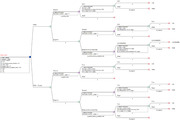

## 130 | Disordered crosstalk between the EGF and FGF2 pathways is implicated in dysregulated trophoblast invasion

Colleen Sinnott^1^, Viktoriia Kolesnyk^2^, Olga Grechukhina^2^, Yang Yang‐Hartwich^2^

^1^Yale University, New Haven, Connecticut; ^2^Yale School of Medicine, New Haven, Connecticut

12:15–13:30

**Objective**: Trophoblast over‐invasion into the decidua results in placenta accreta spectrum (PAS) while trophoblast under‐invasion has been implicated in severe preeclampsia (PEC); however, the mechanism of trophoblast invasion remains poorly understood. Our preliminary data indicated that epidermal growth factor (EGF) and fibroblast growth factor‐2 (FGF2) are differentially elevated in the blood of patients with abnormal placentation. We hypothesized that disordered crosstalk between the EGF and FGF2 pathways plays a role in dysregulated trophoblast invasion.

**Study Design**: Serum samples were obtained from patients with healthy pregnancies, PEC, and PAS. Cytokine analysis was performed on 43 samples (Eve Technologies). HTR‐8/SVneo first‐trimester trophoblasts were used for *in vitro* assays. Scratch and cell proliferation assays were performed after treatment with FGF2, EGF, and both FGF2 + EGF. Quantitative polymerase chain reaction (QPCR) was performed to assess mRNA levels of the receptors FGFR1, FGFR2, and FGFR4 after the same treatment conditions. Levels of FGFR1 and FGFR4 in trophoblasts treated with EGF alone were assessed with Western Blot (WB).

**Results**: Cytokine analysis of patient samples found higher levels of EGF in patients with PAS, while FGF2 was elevated in PEC patients when compared to controls. Trophoblast exposure to EGF resulted in increased cell migration and proliferation, but these effects were dampened after treatment with EGF and FGF2 together. QPCR demonstrated down‐regulation of FGFR1, FGFR2, and FGFR4 mRNA after treatment with EGF. Down‐regulation of FGFR1 and FGFR4 at the protein level after treatment with EGF was confirmed with WB.

**Conclusion**: EGF down‐regulates key FGF2 receptors at mRNA and protein levels in first‐trimester trophoblasts. FGF2, in turn, inhibits the typical migratory and proliferative effects of EGF on trophoblasts. Both FGF2 and EGF pathways seem to play key roles in trophoblast function, and there is interaction between these two pathways in trophoblasts. Future study should aim to understand this crosstalk and its specific role in abnormal placental invasion.

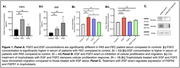

## 131 | Placenta accreta spectrum disorder may protect against hypertensive disorders of pregnancy

Colleen Sinnott^1^, Khadija Alshowaikh^2^, Jennifer F. Culhane^2^, Lisbet S. Lundsberg^2^, Viktoriia Kolesnyk^2^, Caitlin Partridge^2^, Sonya Abdel‐Razeq^2^, Olga Grechukhina^2^

^1^Yale University, New Haven, Connecticut; ^2^Yale School of Medicine, New Haven, Connecticut

12:15–13:30

**Objective**: Placenta accreta spectrum (PAS) is thought to result from over‐invasion of the placenta into the myometrium, while hypertensive disorders of pregnancy (HDP) are thought to arise in part from trophoblast under‐invasion. These proposed biologic mechanisms suggest that PAS and HDP represent opposite ends of a placental invasion spectrum. We investigated whether there is clinical evidence to suggest that PAS is protective against HDP.

**Study Design**: All PAS cases delivered at a single academic institution were identified by ICD‐10 codes and confirmed by individual chart review. The control group was generated by random selection of 4,000 deliveries in the same time period with no PAS. Cases of HDP were identified via ICD‐10 codes. Crude and multivariate logistic regression were performed to calculate the effect of PAS on development of HDP. All blood pressure values were obtained from the electronic medical record and used to calculate mean arterial pressure (MAP) for all patients in each trimester. Mean MAPs were compared by ANOVA.

**Results**: A total of 187 PAS cases and 4,000 control deliveries were included. The groups did not differ in race/ethnicity, insurance type, body mass index, rates of chronic hypertension, or gestational and pre‐gestational diabetes. PAS patients were older, delivered at earlier gestational ages (GA), and more likely to take aspirin and anti‐hypertensive medications. After controlling for these differences, PAS patients had an aOR 0.52 (95% CI [0.32, 0.83], *p* = 0.007) for developing any HDP and an aOR of 0.54 (95% CI [0.31, 0.96], *p* = 0.037) for severe HDP (preeclampsia, eclampsia, and HELLP syndrome). In the first and second trimesters, PAS patients and controls had similar MAPs, but in the third trimester PAS patients had significantly lower MAP than controls (82.7 ± 7.9 *vs* 85.5 ± 7.6, *p* < 0.0001).

**Conclusion**: PAS patients are less likely to develop HDP and have significantly lower MAP values in the third trimester compared to controls. These findings support the plausibility of a biologic mechanism in which trophoblast over‐invasion in PAS protects against development of HDP.

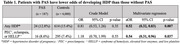

## 132 | Evaluating the association between COVID‐19 vaccination and adverse neonatal outcome

Lior Yahav^1^, Noa Leybovitz Haleluya^2^, Alla Saban^3^, Adi Y Y. Weintraub^4^, Reli Hershkovitz^5^, Tamar Eshkoli^4^

^1^Soroka Medical Center, Beer Sheva, HaDarom; ^2^Soroka Medical Center, Meitar, HaDarom; ^3^Soroka University Medical Center, Beer Sheva, HaDarom; ^4^Soroka University Medical Center, Soroka University Medical Center, HaDarom; ^5^Soroka medical center, Soroka medical center, HaDarom

12:15–13:30

**Objective**: Prior research had not documented any negative impacts of COVID‐19 vaccination on fertility or pregnancy outcomes. Our study aimed to examine neonatal outcomes among pregnant women who received the COVID‐19 vaccine, in comparison to those who were not vaccinated. This analysis was conducted on a cohort of women who remained COVID‐19 negative throughout the peripartum period–spanning from one week prior to hospital admission to up to three days after delivery.

**Study Design**: We conducted a population‐based cohort study at a tertiary medical center, including all women with singleton pregnancies who delivered between 2020 and 2022 and remained uninfected with COVID‐19 throughout the peripartum period. Exclusion criteria included incomplete medical records, multiple gestations, and fetal malformations. The study group comprised women who had received the COVID‐19 vaccine, whereas the control group included women who had not been vaccinated. We examined neonatal adverse outcomes between both groups. A multivariable logistic regression analysis was used to adjust for potential confounders.

**Results**: The study population included a total of 26,250 women who met the inclusion criteria. Among them, 3,379 women had received the COVID‐19 vaccination. Receiving the COVID 19 vaccination was associated with a reduced risk of meconium presence, perinatal mortality, small for gestational age, and Apgar scores below 7 at 1 and 5 minutes. Using a multivariable logistic regression analysis, after adjusting for potential confounders, the association between COVID −19 vaccination and a reduce d risk of meconium presence and low Apgar score at 1 minute remained statistically significant (adjusted OR 0.64, 95% CI 0.45‐0.92; *p* = 0.016; adjusted OR 0.49, 95% CI 0.31‐0.75; *p* = 0.001, respectively).

**Conclusion**: COVID‐19 vaccination in pregnant women was associated with improved neonatal outcomes, showing a reduced risk of meconium presence and lower rates of Apgar scores below 7 at 1 minute.

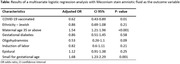

## 133 | The impact of obstetric anal sphincter tears on interpregnancy interval and complications in future deliveries

Lior Yahav^1^, Talia Birenstock^2^, Atar Ben Shmuel^3^, Zehava Yohay^4^, Adi Y Y. Weintraub^5^, Tamar Eshkoli^5^

^1^Soroka Medical Center, Beer Sheva, HaDarom; ^2^Department of Public Health, Faculty of Health Sciences, Ben‐Gurion University of the Negev, Beer‐Sheva, Israel., Beer Sheva, HaDarom; ^3^Soroka medical center, Beer‐Sheva, HaDarom; ^4^Pain Unit, Soroka University Medical Center, Beer Sheva, Israel, Beer Sheva, HaDarom; ^5^Soroka University Medical Center, Soroka University Medical Center, HaDarom

12:15–13:30

**Objective**: Obstetric anal sphincter injuries (OASIS) can lead to long‐term complications for affected patients, however, there is a limited data available on the potential impact of OASIS on the time between pregnancies, known as interpregnancy interval (IPI). Our primary objective was to investigate the IPI of women who had experienced OASIS during delivery, as well as to compare various factors, such as patient and delivery characteristics, pregnancy complications and adverse perinatal outcomes, bew = tween those with a longer vs. shorter IPI in their subsequent birth following OASIS.

**Study Design**: This retrospective cohort study analyzed the medical records of women who gave birth at a tertiary medical center between 2015 and 2019 and experienced obstetric anal sphincter injuries (OASIS) during their first delivery, and subsequently had a second pregnancy. Data was retrieved from patient computerized medical records. Kaplan‐Meier curves and cox regression analysis were used to evaluate the IPI, which was defined as the duration from the index delivery to the last menstrual period preceding the subsequent delivery.

**Results**: The study found no significant difference in the mean interpregnancy interval (IPI) between women who had experienced obstetric anal sphincter injuries (OASIS) during their first delivery and those who had not. Women were significantly more likely to have a repeat perineal tear (34.5%, *p* < 0.001), and an episiotomy (11.3%, *p* < 0.001) during a subsequent vaginal birth. Moreover, a greater proportion of women with OASIS had an elective or an emergency caesarean section in their second pregnancy (42.3%, *p* < 0.001). Additionally, known risk factors for OASIS were proven. There was no difference in baseline characteristics or pregnancy outcomes among women following OASIS who had a long versus short IPI.

**Conclusion**: The occurrence of obstetric OASIS does not seem to deter women from planning or having subsequent childbirth. However, women who have had OASIS are more likely to experience perineal tears, require episiotomies or undergo caesarean delivery in their subsequent pregnancy.

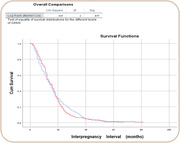

## 134 | Assessing aspartate aminotransferase‐to‐platelet ratio index as a predictor for complications in preeclampsia with severe features

Lior Yahav^1^, Alla Saban^2^, Noa Leybovitz Haleluya^3^, Adi Y Y. Weintraub^4^, Reli Hershkovitz^5^, Tamar Eshkoli^4^

^1^Soroka Medical Center, Beer Sheva, HaDarom; ^2^Soroka University Medical Center, Beer Sheva, HaDarom; ^3^Soroka Medical Center, Meitar, HaDarom; ^4^Soroka University Medical Center, Soroka University Medical Center, HaDarom; ^5^Soroka medical center, Soroka medical center, HaDarom

12:15–13:30

**Objective**: Pregnancy‐related hypertension disorders, especially preeclampsia‐toxemia (PET), present a substantial risk to maternal and fetal well‐being. Aspartate aminotransferase to platelet ratio index (APRI) is a blood test that enables the detection of early inflammatory processes and markers for liver fibrosis and inflammation. Emerging evidence suggests a connection between APRI and outcomes like HELLP syndrome, yet its association with PET remains uncertain. We aimed to assess the predictive ability of APRI in PET with severe features, comparing those with an APRI below the median to those with an APRI above.

**Study Design**: In this retrospective cohort study, we analyzed medical records of women diagnosed with PET with severe features, who gave birth at a tertiary medical center between 2020 and 2022. Blood tests assessed complete blood count and liver enzyme levels. The study included 201 women with singleton pregnancies, with data retrieved from their computerized medical records. Women were categorized into two groups based on their APRI value: below the mean (*n* = 169) and equal to or above the mean (*n* = 32).

**Results**: The study demonstrated a significant increase in placental abruption and longer maternal hospital stays among women diagnosed with PET with severe features and an APRI equal to or above the mean. Except for a statistically significant difference in gestational age at delivery (36.2 weeks for APRI below the mean and 35 weeks for APRI equal to or above the mean), baseline characteristics showed no significant differences between the two groups. In a multivariate analysis, APRI was found as a predictor for placental abruption (OR = 20.5, *p* = 0.001) and longer maternal hospitalization (OR = 4.23, *p* = 0.011), adjusting for maternal age, ethnicity, gestational age and non‐reassuring fetal heart rate.

**Conclusion**: APRI score was found as a predictor for placental abruption and longer maternal hospitalization among women diagnosed with severe PET features. Further research with larger patient cohorts is required to validate and strengthen our findings.

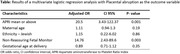

## 135 | Nulliparous women's experience after cervical ripening according to the method: A prospective observational study

Lea DELALANDRE^1^, lucie PLANCHE^1^, Guillaume DUCARME^1^

^1^Centre Hospitalier Departemental, LA ROCHE SUR YON, Pays de la Loire

12:15–13:30

**Objective**: To assess whether method is associated with altered immediate postpartum experience among nulliparous women with cervical ripening at term.

**Study Design**: A prospective observational study included all nulliparous women with a live singleton fetus in cephalic presentation and cervical ripening beyond 37 weeks for maternal and/or fetal disease using cervical ripening balloon (CRB), dinoprostone vaginal insert (PG), oral misoprostol (M) or repeated methods (R). An auto‐administered questionnaire assessed the maternal experience of ripening and delivery in immediate postpartum period. Primary outcome was feelings and experience of cervical ripening using Likert scales from 0 to 10 (very satisfied) according to the method. Secondary endpoints were the evaluations of pain, vaginal discomfort during ripening, and the overall experience of childbirth according to the method.

**Results**: 340 women were included (CRB 33.8%; PG 32.7%; M 3.8%; R 29.7%). The overall vaginal rate was 79.1% and was similar between groups (CRB 77.4%; PG 87.4%; M 69.2%; R 73.3%; *p* = 0.15). The experience of ripening was significantly better with CRB, M or PG, compared to R (6.7 ± 2.5; PG 7.2 ± 2.6; M 6.8 ± 3.6; R 5.2 ± 2.8; *p* < 0.001). The maximum pain during ripening was significantly higher with PG (7.9 ± 2.5; CRB 7.2 ± 2.4; M 7.0 ± 3.9; R 7.8 ± 2.4; *p* = 0.02). The experience of childbirth was more negative in R group (6.1 ± 2.7; CRB 6.9 ± 2.6; PG 7.2 ± 2.4; M 7.4 ± 3.1; *p* = 0.02). After multivariate analysis with adjustment (method, mode of birth, maternal and neonatal morbidities), repeated methods were significantly associated with a worse experience of cervical ripening (aOR = ‐1.3, 95% CI −2.0–‐ 0.66; *p* < 0.001) and childbirth (aOR = −0.73, 95% CI −1.4–−0.07; *p* = 0.03), compared to PG, CRB or M used alone. Maternal experience and childbirth were similar between each method used alone.

**Conclusion**: Nulliparous women with repeated cervical ripening methods at term had a significantly worse experience of cervical ripening and childbirth at immediate postpartum period, compared to women who had PG, CRB or M alone.

## 136 | Carbetocin versus oxytocin following vaginal delivery in high‐risk women: A before‐after study

Margot SAUVEE^1^, lucie PLANCHE^1^, Guillaume DUCARME^1^

^1^Centre Hospitalier Departemental, LA ROCHE SUR YON, Pays de la Loire

12:15–13:30

**Objective**: To compare the effectiveness of prophylactic carbetocin with oxytocin for preventing postpartum hemorrhage (PPH) following vaginal delivery in high‐risk women.

**Study Design**: In a before‐and‐after cohort study between 2021‐2022 in a tertiary maternity hospital, all high‐risk women with vaginal births were included, with two groups compared: prophylactic oxytocin in 2021 and prophylactic carbetocin in 2022. In February 2022, protocol for PPH prevention immediately after vaginal delivery changed for high‐risk women: oxytocin (5 IU) IV was replaced by carbetocin (100µg) IV. High‐risk women were defined by at least one of the following criteria: previous PPH (blood loss >500mL), antenatal suspicion of macrosomia (estimated fetal weight >90^th^ p), twin pregnancy, repeated cervical ripening methods, polyhydramnios, multiparity (≥4), and rapid labor ( < 2 hours) without analgesia. The primary outcome was PPH. Secondary outcomes included severe PPH (blood loss≥1000mL), need for sulprostone and second‐line therapies (Bakri balloon, uterine compression sutures, uterine artery embolization, and peripartum hysterectomy) and maternal morbidity (defined by at least one of the following criteria: blood transfusion, Δ hemoglobin pre‐postpartum >4g/dL, Δ hematocrit pre‐postpartum >10%, and intensive care unit admission).

**Results**: 754 women were included (377 women in each group). Different high‐risk criteria, maternal and labor characteristics among included women were similar between groups. The incidence of PPH was not different between groups (7.4% after carbetocin *vs*. 9.3% after oxytocin; *p* = 0.36). The incidence of severe PPH was similar in both groups (2.9% *vs*. 2.7%; *p* = 0.83). Others secondary outcomes including maternal morbidity did not also differ between groups.

**Conclusion**: Prophylactic carbetocin is similar to oxytocin at preventing post‐partum hemorrhage due to atony after vaginal delivery in high‐risk women.

## 137 | Safety and clinical impact of blood pressure self‐monitoring in high‐risk pregnancies: The multicenter SAFE@home‐II study

Shinta Moes^1^, Martine Depmann^2^, Arie Franx^3^, Elles in't Anker^4^, Flip W. W. van der made^5^, Jacques Dirken^6^, Leonoor van Eerden^7^, Lindy Santegoets^8^, Marc Spaanderman^9^, Roel de heus^10^, Sanne J. Gordijn^11^, Steven Koenen^12^, Titia Lely^13^, Mireille N. Bekker^14^

^1^UMC Utrecht, Amsterdan, Noord‐Holland; ^2^UMC Utrecht, Utrecht, Utrecht; ^3^Erasmus University Medical Center, Zuid‐Holland; ^4^Bravis Ziekenhuis, Bergen op Zoom, Utrecht; ^5^St. Franciscus Hospital, Rotterdam, Zuid‐Holland; ^6^Jeroen Bosch Ziekenhuis, Den Bosch, Noord‐Brabant; ^7^Maasstad Ziekenhuis, Rotterdam, Zuid‐Holland; ^8^Reinier de Graaf Gasthuis, Delft, Zuid‐Holland; ^9^MUMC+, Maastricht, Limburg; ^10^St. Antonius Ziekenhuis, Utrecht, Utrecht; ^11^University Medical Center Groningen, Groningen, Groningen; ^12^Elisabeth‐TweeSteden Ziekenhuis, Tilburg, Noord‐Brabant; ^13^UMC Utrecht, UMC Utrecht, Utrecht; ^14^University Medical Center Utrecht, Utrecht

12:15–13:30

**Objective**: Hypertensive disorders of pregnancy (HDP) affect 10% of pregnancies causing serious adverse maternal and neonatal outcomes. Pregnancies complicated by (high risk of) HDP require frequent monitoring of blood pressure and symptoms. This burdens healthcare resources and patients. Globally, obstetric care is facing issues of accessibility, affordability, and sustainability. A former pilot study indicated that home monitoring of patients with (high risk of) HDP using a digital platform (SAFE@home) is a promising solution. It showed fewer hospital visits, lower costs and high appreciation by patients. In this study we investigated safety and clinical impact in a large‐scale setting.

**Study Design**: We performed a multicenter before‐after study in 11 Dutch hospitals. Pregnant women with a singleton pregnancy and (a high risk of) HDP monitored at home using the SAFE@home platform were compared with a control group receiving usual care. Primary outcome was a composite of adverse maternal and neonatal morbidity as well as mortality. Logistic regression analyses were performed to test non‐inferiority of SAFE@home for clinical effectiveness.

**Results**: In 1212 patients, a significantly lower incidence of the primary composite outcome in the SAFE@home group (17.2%) was observed compared to the usual care group (20.3%) with an adjusted odds ratio (aOR) of 0.63 (95% CI 0.44‐0.90). Of the composites, severe hypertension was significantly lower in the SAFE@home group (12.7%) compared to usual care (16.3%) (OR 0.61 (95% CI 0.41–0.90). Significantly more patients started oral antihypertensives in the SAFE@home group compared to usual care (31% vs. 25%, *p* = 0.01) and fewer iatrogenic preterm births < 34weeks, occurred in SAFE@home patients (3.6%) compared to usual care (5.6%) (aOR 0.49 (95% CI 0.26–0.91)).

**Conclusion**: This study shows that telemonitoring in high‐risk pregnancies is not only safe but is also significantly associated with a lower rate of adverse perinatal outcomes as compared to hospital care. Our research shows the clinical benefits and transformative potential of telemonitoring in managing high‐risk pregnancies.

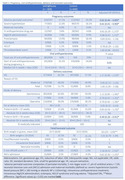

## 138 | Novel risk factors associated with retained placenta after vaginal birth

Basel Habib. Nasser^1^, Jimmy Jadaon^2^, Nibal Khamaisy^2^, Luna Abu‐Alfol^3^, Israel Hendler^4^

^1^Nazareth Hospital, affiliated with the Azrieli Faculty of Medicine at Bar Ilan University, Israel, Nazareth hospital, HaZafon; ^2^Nazareth Hospital, Israel, HaZafon; ^3^Nazareth Hospital, Nazareth Hospital, HaZafon; ^4^Nazareth Hospital, Tel Aviv

12:15–13:30

**Objective**: Retained placenta is a serious complication of vaginal birth. The ability to predict retained placenta may have positive effect on timely management. We aimed to evaluate the maternal and obstetric risk factors associated with retained placenta after singleton live vaginal birth

**Study Design**: A retrospective cohort of women who had retained placenta after singleton live vaginal birth ≥ 24 weeks, compared in 1:2 ratio with women who had normal vaginal delivery without complications. Study and control group were matched for maternal age, gestational age and parity.

Multivariate regression analysis was performed to evaluate the potential risk factors for retained placenta including maternal and obstetrical characteristics

**Results**: Fifteen thousand two hundred sixty women underwent vaginal delivery at our medical center between 2015‐2022. One hundred seventy women (1.1%) were diagnosed with retained placenta. Ninety‐nine women (0.65%) who met the inclusion criteria, were matched with 198 women (1.3%) as controls.

Multivariate logistic regression revealed various potential risk factors not previously described as associated with retained placenta including: IVF pregnancy (OR 3.8, 95% CI [1.3‐ 11.7], P 0.018), preeclampsia (OR 4.5, 95% CI[1.1‐ 17.5], P 0.0315), women with large for gestational age fetus (OR 28.2, 95% CI [5.4‐ 148.5], P 0.0298), labor Induction (OR 21.8, 95% CI [5.5‐ 86.8], *p* < 0.001), vacuum assisted vaginal delivery (OR 2.3 .95% CI [1.2‐ 4.5], P 0.011), and Duration of second stage > 3 hours (OR 3.9,95% CI [1‐ 15.1], *p* < 0.001)

**Conclusion**: Our study highlights unreported risk factors associated with retained placenta such as macrosomia, in vitro fertilization and endometriosis. This emphasis on early risk identification and the exploration of possible preventive measures holds promise for enhancing antenatal care practices, ultimately improving outcomes for both mothers and infants.

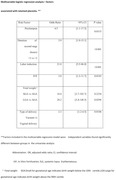

## 139 | From Cesarean to VBAC: Assessing prostaglandin‐induced labor induction

Shanny kolp asis^1^, Elad Miron^2^, Limor Vaknin Geron^2^, Olena Minich^3^, Adi Ashkenazi Katz^2^

^1^Tel Aviv university, Tel aviv, HaMerkaz; ^2^Tel Aviv University, Tel Aviv University, Tel Aviv; ^3^Tel Aviv University, Kfar Saba, HaMerkaz

12:15–13:30

**Objective**: Labor induction in women with a previous cesarean section carries risks such as uterine rupture, impacting delivery outcomes. This study evaluates the safety and effectiveness of prostaglandins compared to other methods for inducing labor in this group, aiming to define their role in post‐cesarean deliveries.

**Study Design**: In this retrospective study at a University‐Affiliated medical center, we assessed the safety of labor induction using prostaglandins compared to oxytocin and balloon catheters in women aged 17‐45 with a singleton pregnancy at ≥36 weeks gestation and a prior cesarean section. A sub‐analysis was conducted on second deliveries to eliminate the impact of parity and VBACs (Vaginal Birth After Cesarean). The primary goal was to evaluate the safety of prostaglandins, particularly in terms of uterine rupture and vaginal delivery success. Data extracted from medical records.

**Results**: The study analyzed 8,460 labors in women with prior cesarean sections, finding a 0.4% overall uterine rupture risk. Spontaneous labor had the lowest cesarean (5.2%) and rupture rates (0.3%). Active inductions showed higher risks, with oxytocin leading at 18.9% for cesareans and 2.2% for ruptures. Balloon catheters resulted in 18.4% cesarean and 0.5% rupture rates, while prostaglandins had 13.1% and 0.6% rates, respectively, the latter not significantly different from balloon catheters.

**Conclusion**: This study highlights the effectiveness of prostaglandins for inducing labor in women with prior cesarean sections, increasing vaginal birth rates without raising uterine rupture risks significantly. Due to the limitations of retrospective analysis, more randomized controlled trials are needed to confirm these findings.

## 140 | The paradox of parity: Dissecting uterine rupture risks in post‐cesarean births

Shanny kolp asis^1^, Elad Miron^2^, Oshrit Shtossel^3^, Adi Ashkenazi Katz^2^, Olena Minich^4^, Limor Vaknin Geron^2^, Ariel Many^5^, bari kaplan^2^

^1^Tel Aviv university, Tel aviv, HaMerkaz; ^2^Tel Aviv University, Tel Aviv University, Tel Aviv; ^3^bar ilan University, bar ilan University, HaMerkaz; ^4^Tel Aviv University, Kfar Saba, HaMerkaz; ^5^Department of Obstetrics and Gynecology, Mayanei Hayeshua Medical Center, bnei brak, HaMerkaz

12:15–13:30

**Objective**: Uterine rupture is a major obstetric complication impacting both mother and child, with significant risk factors including grand multiparity and a history of cesarean delivery (CS). This study assesses whether the risk of uterine rupture is greater in grand multiparous women with prior CS compared to those without grand multiparity.

**Study Design**: This retrospective study, conducted at a university‐affiliated medical center from November 2011 to January 2024, analyzed 126,952 singleton births, excluding non‐labor cesareans. We identified 48 cases of uterine rupture and evaluated the risk associated with parity in women with and without previous CS. The study compared risks across induction methods—prostaglandins, balloon, and oxytocin—versus spontaneous labor and examined factors such as maternal age, birth weight, prior miscarriages, and ectopic pregnancies, with data verified through extensive medical records review.

**Results**: Analysis revealed that uterine rupture risk decreases with increasing parity in women with a previous CS, from 0.8% at first parity to less than 0.3% at seventh parity (*p* < .001). Conversely, in women without a cesarean history, the risk increases from 0% at first parity to over 0.06% at eighth parity, although it remains significantly lower than in women with prior cesareans. Additionally, the number of VBACs inversely correlates with rupture risk, decreasing from 0.6% with no previous VBACs to 0.2% with 4 VBACs (*p* < .001). Birth induction risks vary by method, Oxytocin posed the highest rupture risk at 0.13%, followed by prostaglandins at 0.11%, and balloon at 0.07%, with spontaneous labor at the lowest at 0.02% (*p* < .001). No correlation was found between uterine rupture risk and maternal age, previous miscarriages, ectopic pregnancies, or birth weight.

**Conclusion**: Our findings suggest that the risk factors for uterine rupture in grand multiparous women with a cesarean history are not cumulative. Indeed, such women have a lower risk of rupture than what might be expected, challenging the assumption that grand multiparity and previous cesarean are synergistic risk factors.

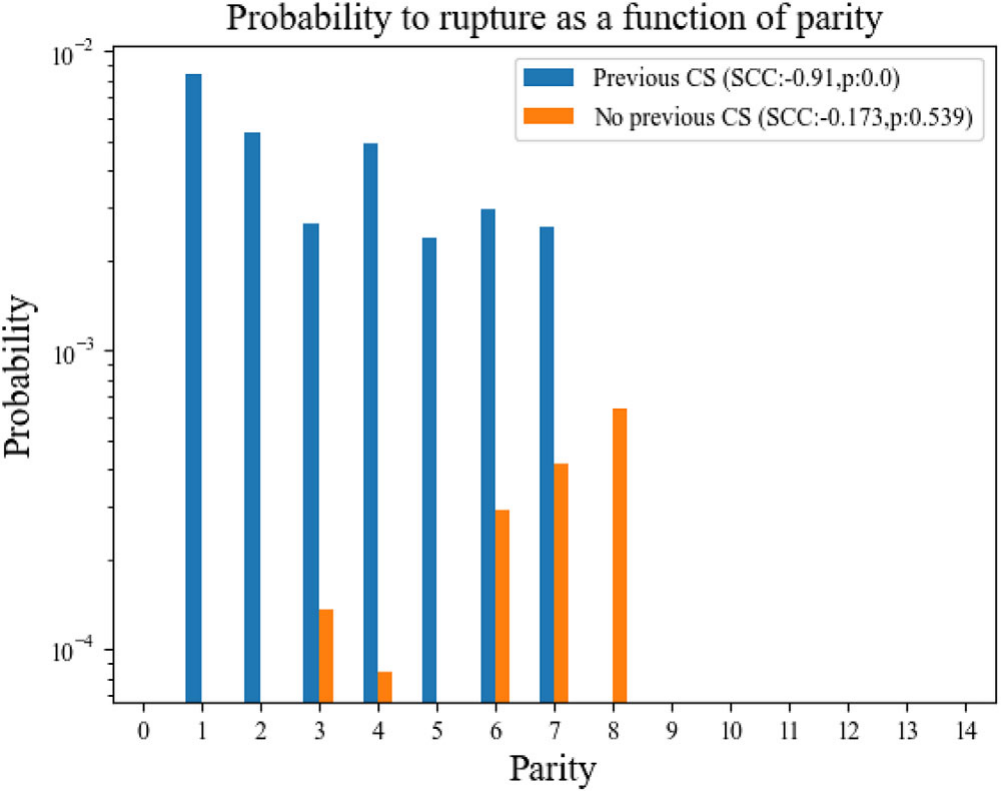

## 141 | The effect of a multidisciplinary preoperative counseling clinic for scheduled cesarean delivery on breastfeeding rates

Or Marom^1^, Liat Mor^2^, Eran Weiner^1^, Yael Ganor Paz^2^, Irit Segman^1^, Ilia Kleiner^3^, Tamar Shyldkrot^4^, Moran Dan Shmilovitz^1^, Zvia Hiaev^1^, Racheli Shalom Turgeman^1^, Noa Gonen^5^

^1^Wolfson Medical Center, Wolfson Medical Center, Tel Aviv; ^2^Edith Wolfson Medical Center, Holon, HaMerkaz; ^3^Edith Wolfson Medical Center, Edith Wolfson Medical Center, Tel Aviv; ^4^Wolfsom Medical Center, Wolfsom Medical Center, Tel Aviv; ^5^Sheba Medical Center, Ramat gan, HaMerkaz

12:15–13:30

**Objective**: Infants born by cesarean delivery (CD) are less likely to be breastfed than vaginally delivered ones. In September 2022 we initiated a novel clinic dedicated to medical and holistic counseling to patients scheduled for CD. We aimed to investigate whether this clinic increases breastfeeding rates (BR) within initial 24 hours, 3 months postpartum, and other aspects of recovery and satisfaction.

**Study Design**: We compared 2 cohorts of patients undergoing scheduled CD at our tertiary center in two periods: before (P1) and after (P2) the inauguration of our clinic. The clinic comprised of a senior obstetrician counseling followed by a midwife meeting in which a birth plan was developed. Outcomes were compared between groups and a questionnaire assessing breastfeeding 3 months postpartum and patient satisfaction was conducted. Primary outcome was BR within initial 24 hours after CD. Secondary outcomes were skin to skin and zero separation during surgery, pain level 24 h post‐op, patient satisfaction, and BR 3 months postpartum. A sample size of 492 patients was calculated to demonstrate a 25% difference in the primary outcome.

**Results**: Overall, 492 patients were included in the study, 246 in each group. There were no between‐groups differences in demographics, surgical and neonatal outcomes. The rate of BR during first 24h, and during surgery was significantly higher in P2 (59.7% vs 41.1%; and 38.6% vs 5.6%, both *p* =  < 0.001), in addition to higher rates of skin‐to‐skin (75.6% vs 59.3%), and zero separation during CD (33.3% vs 5.6%), and in the maternity ward (30% vs 10.1%, *p* =  < 0.001 for all). Patients in P2 reported significantly lower pain levels 24 hours after surgery (4.0 ± 3.2 vs 5.4 ± 3.3, *p* =  < 0.001), higher satisfaction (9.5 ± 1.0 vs 9.3 ± 1.2, *p* = 0.025) and increased BR 3 months post‐partum (40.2% vs 24.3%; *p* =  < 0.001), compared to P1.

**Conclusion**: Implementing a specialized clinic for scheduled CD increases BR during surgery, within 24 hours postpartum, and 3 months after. Furthermore, it reduced post‐op pain, increased skin‐to‐skin contact, zero separation, and patient satisfaction.

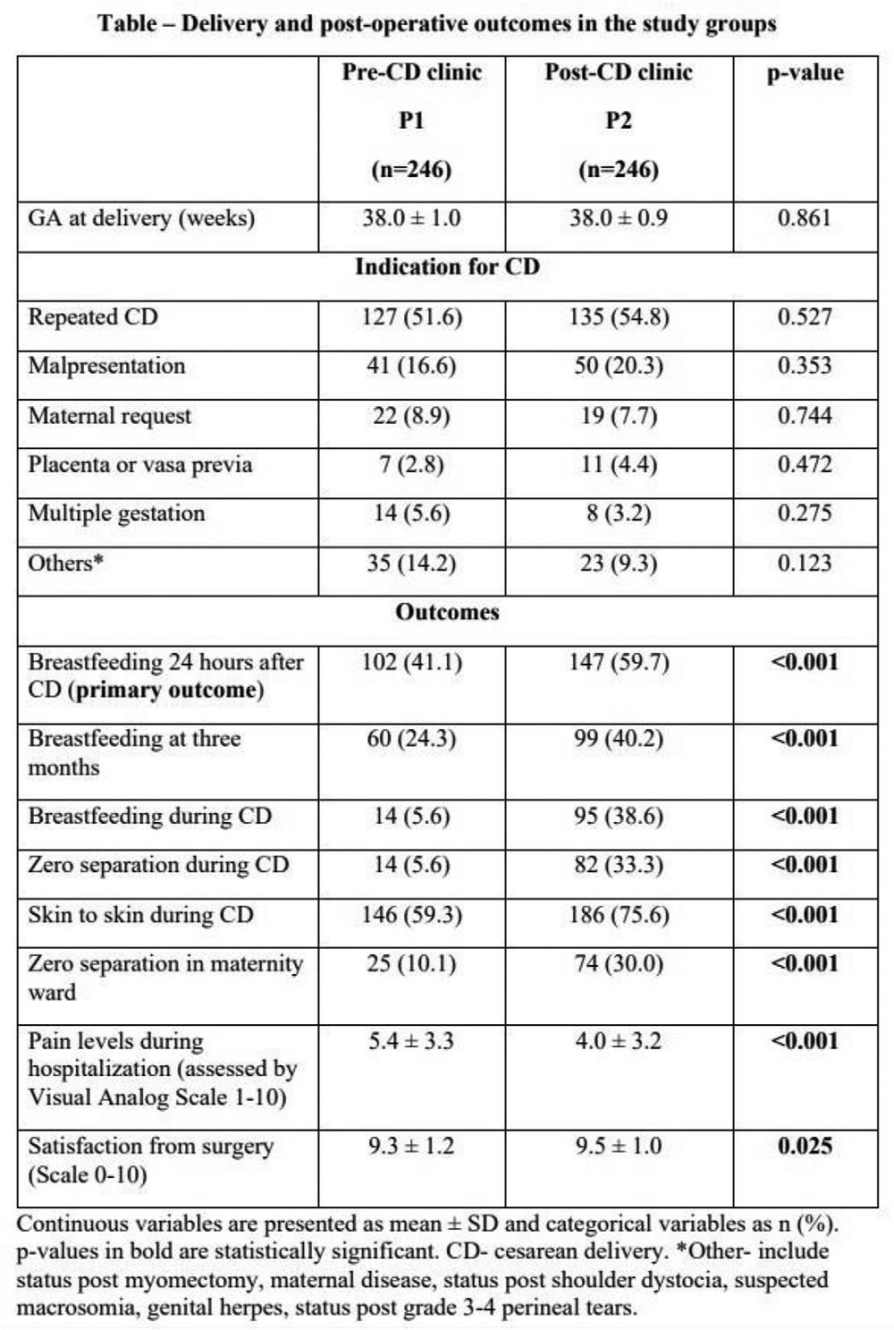

## POSTER SESSION 2

Thursday, 26 September 2024 17:15–18:30

## 143 | Person‐centered prenatal care in low‐and middle‐income settings: Scale development and validation in Ghana

Patience A. Afulani^1^, Hawa Malechi^2^, Moro Ali^3^, Daniel Enos Sekwo^4^, Odiase Osamuedeme^5^, Monica Getahun^5^, Raymond Aborigo^4^

^1^University of California, San Francisco, San Francisco, CA; ^2^Tamale Teaching Hospital, Tamale Teaching Hospital, Northern; ^3^Navrongo Health Research Center, Navrongo Health Research Center, Upper West; ^4^Navrongo Health Research Center, Navrongo Health Research Center, Upper East; ^5^University of California, San Francisco, University of California, San Francisco, California

17:15–18:30

**Objective**: Person‐centered prenatal care (PCPC)–prenatal care that is respectful of and responsive to people's preferences, needs, and values–is important to achieving optimal pregnancy outcomes. Yet, there are no validated tools to comprehensively measure PCPC in low‐and middle‐income countries (LMICs). We aimed to develop and validate a tool to comprehensively measure the PCPC experiences of women in LMICs.

**Study Design**: We followed standard procedures for scale development. This included literature review to adapt items from a prior scale developed in the US and to generate new items relevant to LMICs; expert reviews with maternal health experts, health care providers, and women with lived experiences (currently pregnant or previously pregnant) to assess content validity; and cognitive interviews with pregnant and postpartum women to assess clarity, appropriateness, and relevance of the questions. Questions were iteratively revised at each stage and finally administered in a survey to 299 pregnant and 300 postpartum women (given birth within the last 6 months) in the Upper East Region of Ghana. Data from 594 respondents were used in psychometric analysis to assess construct and criterion validity and reliability.

**Results**: Exploratory factor analysis reduced the number of items from 55 to 36, with one dominant factor, and three factors with eigenvalues of greater than one. Items were grouped into three conceptual domains representing subscales for “dignity and respect,” “communication and autonomy,” and “responsive and supportive care.” The Cronbach alpha for the full scale is 0.90, and that for the subscales are each >0.8. The summative PCPC scores are correlated with global measures of prenatal care satisfaction suggesting good criterion validity.

**Conclusion**: The PCPC scale has high validity and reliability in a sample of prenatal and postpartum women in Ghana. This scale will facilitate efforts to measure and improve respectful of and responsive prenatal care.

## 144 | Is inpatient opioid usage after cesarean delivery associated with patient‐reported pain two weeks postpartum?

Kali Stewart^1^, Rachel Paul^2^, Ashley Veade^3^

^1^Washington University in St. Louis–Barnes Jewish Hospital, SAINT LOUIS, MO; ^2^Washington University School of Medicine, St. Louis, Missouri; ^3^Washington University in St. Louis, Washington University in St. Louis, Missouri

17:15–18:30

**Objective**: Postpartum pain experience and patient satisfaction are closely tied. Identifying objective measures to predict the postpartum pain experience after cesarean delivery (CD) may help guide postpartum counseling and support for patients. We evaluated the association between inpatient opioid usage, measured in morphine milligram equivalents (MME) and patient‐reported post‐CD pain.

**Study Design**: We conducted a prospective, observational study with patients undergoing CD at a single academic hospital from February to October 2021. Patients with opioid use disorder, wound complication, peripartum hysterectomy or readmission were excluded. Inpatient opioid consumption from 24 hours post‐CD to hospital discharge was extracted from the medical record. Patients were contacted by telephone two weeks post‐discharge and asked to rate their pain on a 5‐point Likert scale; we collapsed responses to “well‐controlled” and “poorly‐controlled” for analysis. We used Wilcoxon rank sum and chi‐square to compare the two pain groups.

**Results**: We collected inpatient opioid usage for 143 patients; 109 (76.2%) completed the two‐week call and were included in the analysis. Two weeks postpartum, 68 (62.4%) reported well‐controlled pain and 41 (37.6%) reported poorly‐controlled pain. Patients reporting well‐controlled pain were more likely to have private insurance (66.2% vs 34.2%, *p* = 0.001) and less likely to have had a prior CD (32.4% vs 53.7%, *p* = 0.03). Well‐controlled patients consumed less inpatient MME than poorly‐controlled patients (well‐controlled: median 60.1 (IQR 18.8‐93.8); poorly controlled: 83.5 (60.0‐105.0), *p* = 0.01).

**Conclusion**: Patients undergoing CD with higher MME usage during their inpatient stay were more likely to report poorly controlled pain at two weeks postpartum. Utilizing an inpatient MME consumption metric could identify patients who may benefit from additional counseling and support during admission to optimize their postpartum pain experience.

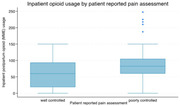

## 145 | Subjective versus objective pain assessment in the postpartum period: Improving pain control after vaginal delivery

Kali Stewart^1^, Lauren Holroyd^2^, Rebecca Rimsza^3^, Ashley Veade^4^

^1^Washington University in St. Louis–Barnes Jewish Hospital, SAINT LOUIS, MO; ^2^Washington University School of Medicine, St. Louis, Missouri; ^3^University of Nebraska Medical Center, Omaha, Nebraska; ^4^Washington University in St. Louis, Washington University in St. Louis, Missouri

17:15–18:30

**Objective**: The numerical pain rating scale (NPRS) is a validated measure to objectively quantify the experience of acute pain. However, the utility of NPRS after vaginal delivery is unclear. Poorly controlled postpartum pain can lead to increased rates of complications, therefore patient satisfaction with pain control is paramount. We investigated how subjective pain relates to objective pain in the postpartum period.

**Study Design**: A prospective, observational study at a single academic hospital included patients with vaginal deliveries from February‐October 2021. Women with opioid use disorder, wound complication, peripartum hysterectomy, or readmission were excluded. Subjective pain assessment and NPRS were completed 2 weeks post‐discharge. Subjective pain was recorded as ranging from “well controlled” to “poorly controlled” and NPRS was scored from 0‐10 and was further subdivided into categories: none (0), mild (1‐3), moderate (4‐6), and severe (7‐10).

**Results**: 238 patients were included and 174 (73%) followed up. On subjective assessment 120 (69%) reported pain as well controlled and 54 (31%) reported pain as poorly controlled. Both groups were similar in mode of delivery, laceration and use of adjuncts (*p* > 0.05 for all). Patients who used oxycodone while admitted were significantly more likely to report poorly controlled pain (8.3% vs 20.4%, *p* = 0.02). Poorly controlled pain was associated with a higher median NPRS score [5 (IQR 4‐6)] vs 2 (IQR 0‐3), *p* < 0.001]. Notably, among patients who reported poorly controlled pain, 11 (20.4%) had objectively none to mild pain and of those who reported well controlled pain, 22 (18.3%) had objectively moderate to severe pain.

**Conclusion**: While objective pain scores were higher for postpartum patients with poorly controlled pain and lower for those with well controlled pain, this was not universal. It is critical to accurately assess and respond to postpartum pain and for nearly 1/5th of patients their subjective experience did not correlate with their objective score. Titrating pain control to both subjective and objective goals may improve patient experience and outcomes.

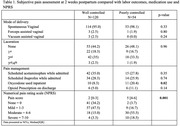

## 146 | The association of vacuum assisted delivery and offspring neurodevelopmental outcomes; a nationwide population‐based study

Ida Björk^1^, Gunilla Ajne^2^, Ängla Mantel^3^

^1^KI/Clintec, Dep of obstetrics and Gynecology, Karolinska Institute/Stockholm, Stockholms Lan; ^2^Karolinska Institute, KI/Stockholm, Stockholms Lan; ^3^KI, KI/Stockholm, Stockholms Lan

17:15–18:30

**Objective**: Ensuring a safe delivery is of utmost importance and selecting the optimal method of delivery presents a challenge. Studies indicate a higher risk of severe neonatal complications associated with vacuum assisted delivery (VAD) performed at mid/low station compared to emergency caesarean delivery (ECD), but research on the long‐term outcomes for offspring is scarce.

**Study Design**: This population‐based study evaluated the association between delivery mode and long‐term neurodevelopment disorder in childhood combining Swedish health registers. The cohort was identified in The Swedish Medical Birth Register, which cover 98% of the births in Sweden, from1997 to 2014 and followed until 2021. Data is prospectively collected. Singleton children born at term gestation to primiparous women were eligible and the inclusion years was defined based on the minimal attained age of 7 years at end of follow‐up. Exclusions were forceps‐assisted birth, breech presentations or congenital anomalies. Exposed cohort was VAD, further stratified into outlet and mid/low station, and compared to ECD. Spontaneus vaginal delivery was defined as an external reference group. Cox proportional Hazards regression models was used to calculate adjusted Hazard Ratios (aHRs) with 95% CI. Outcome variables were attention deficit hyperactivity disorder (ADHD), autism spectrum disorder (ASD), intellectual disability (ID), cerebral palsy (CP) and epilepsy (EP).

**Results**: In total 630 985 deliveries were included. VAD at outlet station was calculated a lower aHR for all studied neurodevelopment disorders compared to ECD, except CP with no difference. There was no calculated difference between low/mid VAD and ECD, except a lower aHR for ID.

**Conclusion**: ECD and low/mid VAD exhibit the same risk for long‐term neurodevelopment disorders, except intellectual disability with a lower risk after VAD. The method of delivery with fetal head at low/mid station may not matter regarding long‐term neurodevelopment. The use of a vacuum cup at outlet station is safe.

## 148 | Hypertensive disorders of pregnancy and childhood neurodevelopment: A meta‐analysis of over 27 million children

Jessica A. Atkinson^1^, Hannah Gordon^2^, Stephen Tong^3^, Sue Walker^4^, Parinaz Mehdipour^5^, Anthea Lindquist^5^, Roxanne Hastie^5^

^1^The University of Melbourne, Heidelberg, Victoria; ^2^University of Melbourne, Victoria; ^3^Department of Obstetrics, Gynaecology and Newborn Health, University of Melbourne, Heidelberg, Victoria, Victoria; ^4^Department of Obstetrics, Gynaecology and Newborn Health, University of Melbourne, Melbourne, Victoria; ^5^University of Melbourne, University of Melbourne, Victoria

17:15–18:30

**Objective**: Hypertensive disorders of pregnancy may be associated with an increased risk of adverse health outcomes for exposed offspring. However, there has been no comprehensive review of neurodevelopmental outcomes among these children. Thus, we undertook a systematic review and meta‐analysis investigating the association between hypertensive disorders of pregnancy and childhood neurodevelopment.

**Study Design**: We searched MEDLINE, CINAHL, Web of Science, and PsychInfo for studies reporting neurodevelopmental outcomes of children born following hypertensive disorders of pregnancy compared with those born following normotensive pregnancy. Studies reporting similar outcomes were pooled using random effects meta‐analysis and reported as odds ratios (OR) or mean difference (MD) with corresponding 95% confidence intervals (CI).

**Results**: After screening 12,580 records, 108 studies reporting the outcomes of 27,369,463 offspring were included. Compared with unaffected pregnancies, hypertensive disorders were associated with an increased likelihood of childhood autism (OR 1.49 [95% CI 1.41‐1.57]; *n* = 26,229,760; 42 studies); attention‐deficit/hyperactivity disorder (OR 1.24 [1.19‐1.29]; *n* = 12,062,933; 15 studies); intellectual disability (OR 1.80 [1.38‐2.35]; *n* = 10,448,230; 10 studies); global developmental delay (OR 1.65 [1.40‐1.94]; *n* = 2,529,908; 36 studies); poor educational outcomes (OR 1.36 [1.27‐1.46]; *n* = 1,248,644; 4 studies); and decreased mean intelligence scores (MD −2.32 [‐3.95, −0.68]; *n* = 1,092,851; 14 studies). When conducting subgroup analyses by prematurity, no differences in likelihood of poor neurodevelopment were seen among preterm children ( < 37 weeks).

**Conclusion**: Hypertensive disorders of pregnancy are associated with an increased likelihood of poor neurodevelopment among offspring. These findings were not present when correcting for prematurity. Our findings suggest that morbidity attributed to hypertensive disorders of pregnancy may be in part due to the need for early birth among affected pregnancies.

## 149 | Fetal fraction in noninvasive testing to predict adverse pregnancy outcomes: Nationwide study of 56,110 pregnancies

Ellis Becking^1^, Ewoud Schuit^2^, Jens Henrichs^3^, Caroline Bax^3^, Erik Sistermans^3^, Lidewij Henneman^3^, Peter Scheffer^4^, Mireille N. Bekker^5^

^1^Wilhelmina Children's Hospital, University Medical Center Utrecht, The Netherlands, Wilhelmina Children's Hospital, University Medical Center Utrecht, The Netherlands, Utrecht; ^2^University Hospital Centre Utrecht, University Hospital Centre Utrecht, Utrecht; ^3^Amsterdam UMC, Amsterdam UMC, Noord‐Holland; ^4^University Medical Centre Utrecht, University Medical Centre Utrecht, Noord‐Holland; ^5^University Medical Center Utrecht, Utrecht

17:15–18:30

**Objective**: Fetal fraction, defined as the proportion of placenta‐originated cell‐free DNA in the maternal circulation, is routinely measured as a quality parameter in noninvasive prenatal testing (NIPT). The objective of this study was to assess the prognostic value of fetal fraction in the prediction of adverse pregnancy outcomes.

**Study Design**: We assessed the prognostic value of fetal fraction in a cohort of 56,110 pregnant women. A model was constructed that included known clinical parameters from current first trimester prediction models (base model) and fetal fraction was added to this base model (fetal fraction model). The prognostic value was assessed by comparing measures of goodness of fit and predictive performance between the base model and the fetal fraction model.

**Results**: Adding fetal fraction to the base model significantly improved model fit (likelihood ratio test *p* < 0.05) for hypertensive disorders of pregnancy (HDP), birthweight < p10, birthweight < p2.3, spontaneous preterm birth (sPTB), and diabetes, but not for congenital anomalies (Table 1). For HDP, the area under the curve (AUC) changed from 0.67 to 0.68 by adding the fetal fraction to the base model (*p* = 0.14) and the integrated discrimination index (IDI) was 0.0018 (*p* < 0.0001). For birthweight < p10, the AUC improved from 0.65 to 0.66 (*p* < 0.0001) and the IDI was 0.0023 (*p* < 0.0001). For birthweight < p2.3, the AUC improved from 0.67 to 0.69 (*p* < 0.0001) and the IDI was 0.0011 (*p* < 0.0001). For all sPTB, the AUC was similar for models with and without fetal fraction (AUC 0.63, *p* = 0.021) and the IDI was 0.00028 (*p* = 0.0023). For diabetes, the AUC was similar (AUC 0.72, *p* = 0.35) and the IDI was 0.00055 (*p* = 0.00015).

**Conclusion**: This is the first large‐scaled study investigating the potential of fetal fraction in NIPT as biomarker to predict adverse pregnancy outcomes. We found that fetal fraction has a statistically significant but limited prognostic value in the prediction of adverse pregnancy outcomes. Future studies should focus on adding fetal fraction to clinically validated prediction models.

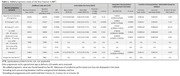

## 150 | Risks associated with IVF pregnancies conceived following male infertility

Alexandra Berezowsky^1^, Adi Borovich^2^, Eran Hadar^3^, Avital Wertheimer^4^

^1^University of Toronto, Toronto, Ontario; ^2^Tel Aviv University, Tel Aviv University, New York; ^3^Rabin Medical Center, Petach Tikva, HaMerkaz; ^4^Tel Aviv University, Tel Aviv University, Tel Aviv

17:15–18:30

**Objective**: We aimed to evaluate placental related adverse outcomes (as IUGR, preeclampsia) associated with an IVF pregnancy, indicated solely by a male infertility (MF), in healthy nulliparous women.

**Study Design**: This was a retrospective case control study of nulliparous women conceived by IVF d/t MF, carrying a singleton gestation between 2007 and 2020. Cases were matched to controlled by age and delivery date in a ratio of 1:10. Pregnancies complicated by multiple gestations, major fetal anomalies or history of chronic medical condition were excluded. The following composite outcomes were defined: 1) Pregnancy related (delivery prior to 34 weeks, gestational hypertension, pre‐eclampsia, oligohydramnios, abnormal placentation); 2) Maternal (PPH, blood product transfusion) 3) Fetal composite (admission to NICU at term, SGA, asphyxia, or seizure). Outcomes were compared.

**Results**: Overall 1,826 women were included in the study. Of those 166 were in the study group and 1660 in the control group. Women in the study group had a slightly higher BMI than those in the control group (21.48 vs. 23.43, *p* = 0.01), otherwise women were comparable in baseline characteristics. Those in the study group had slightly higher rate of preterm deliveries, prior to 32+0 weeks gestation (1.80% vs. 0.48%, *p* = 0.03). There was no difference in mode of delivery between the groups, with a rate of cesarean deliveries at 19% of both groups. Primary and secondary outcomes were comparable between the groups, including the pregnancy (12.65% vs. 8.3% *p* = 0.06) and maternal (10.24% vs. 7.5% *p* = 0.21) and composite outcomes. Initially fetal composite outcome rates were higher in the study group (19.28% vs. 13.19%, *p* = 0.02), but this difference become insignificant after controlling for maternal age, BMI and gestational age at delivery.

**Conclusion**: Adverse outcomes are comparable between healthy, nulliparous women conceiving by an IVF technology for the solely indication of male infertility, when compared to women who conceived spontaneously at the same maternal age.

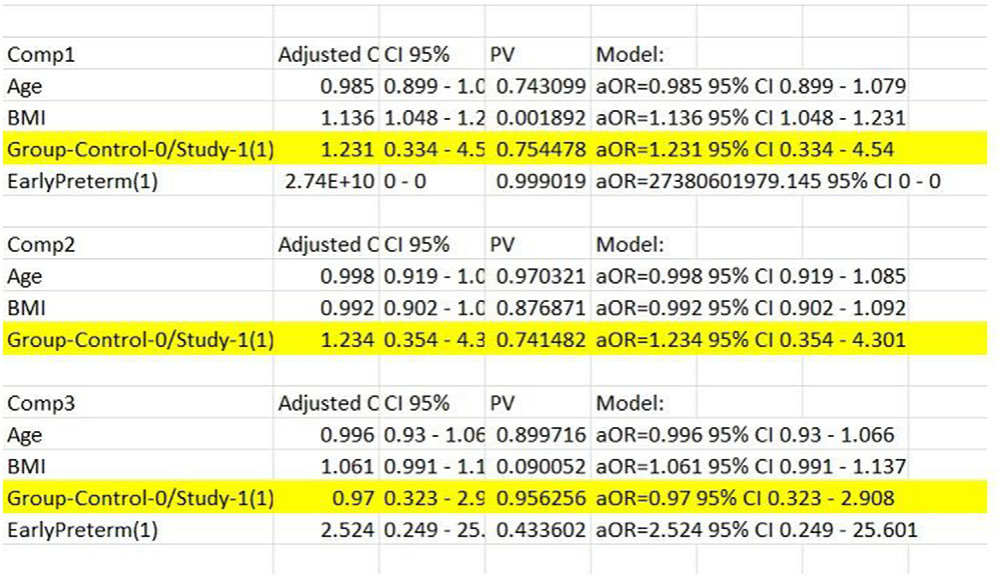

## 155 | Universal survival of “lethal” osteogenesis imperfecta: Rethinking perinatal management from diagnosis to delivery

Megan B. Raymond^1^, Joanna D. Costa^2^, Ricki S. Carroll^2^, Andrea J. Schelhaas^2^, Cristina McGreal^2^, Sarah Little^2^, Jeanne Franzone^2^, Daria E. Willis^3^, Jason K. Baxter^1^, Margaret R. Chou^4^

^1^Thomas Jefferson University, Philadelphia, Pennsylvania; ^2^Nemours Children's Health, Wilmington, Delaware; ^3^Sidney Kimmel Medical College at Thomas Jefferson University, Philadelphia, Pennsylvania; ^4^Nemours Children's Health, Wilmington, DE

17:15–18:30

**Objective**: Patients with a diagnosis of osteogenesis imperfecta (OI) are often counseled that the condition will be life‐limiting or lethal due to skeletal deformity and lung hypoplasia. Advances in delivery planning, neonatal intensive care, pain management, bisphosphonate therapy, and eventual orthopedic interventions for moderate to severe OI offer survival with a meaningful quality of life, challenging established objective ultrasound predictors of lethality. We describe perinatal management of “lethal” OI within a multidisciplinary skeletal dysplasia program.

**Study Design**: In 2019‐2024, we iterated process improvement cycles in delivery planning/technique for suspected or confirmed fetal OI. Clinical outcomes were abstracted from the medical record.

**Results**: 5 of 9 delivered patients presented on referral with a diagnosis of “lethal,” “likely lethal,” or “type II” fetal OI based upon sonographic or genetic data; 8 of 9 fetuses had fetal growth restriction with normal umbilical artery dopplers, 5 had estimated fetal weight (EFW) < 3%ile. EFW within 14 days of birth underestimated birthweight by 25%. There were no fetal demises. 6 of 9 presentations were breech. Shared decision‐making determined timing and mode of delivery. All had scheduled, early‐term cesarean deliveries with surgical technique aimed at minimizing intrapartum atlanto‐occipital and other fractures. No infant required intubation or chest compressions at delivery; all survived (age range 1 week to 4.5 years). Of 7 discharged infants, median length of stay was 46 days (range 14‐366, mean 88), 3 were on room air, 1 on nasal cannula, 2 on continuous positive airway pressure, 1 required tracheostomy; 2 inpatients are on room air.

**Conclusion**: Given advances in medical and surgical care of individuals with OI, counseling for a prenatal diagnosis of “lethal” or severe OI should be nuanced, non‐directive, and concordant with individualized goals of care. In addition to the options of termination or comfort care, the option for multidisciplinary antenatal counseling with thoughtful perinatal care and life‐sustaining neonatal care should be offered.

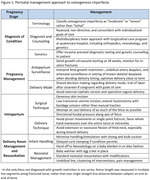

## 156 | Does Amnioinfusion for intrapartum variable decelerations reduce the total deceleration area?

Gal cohen^1^, Nofar Bar Noy Traub^2^, Hanoch Schreiber^2^, Tal Biron‐Shental^3^, Hila Shalev‐Ram^3^, Michal Kovo^2^, Dorit Ravid^2^

^1^Meir Hospital, Meir Hospital, HaMerkaz; ^2^Meir Medical Center, Kfar Saba, HaMerkaz; ^3^Meir Medical Center, Meir Medical Center, HaMerkaz

17:15–18:30

**Objective**: Data regarding the efficiency of amnioinfusion to ameliorate variable decelerations (VD) is limited. Total deceleration area (TDA) has been shown to be a useful indicator of the intrapartum fetal acid‐base status. We aimed to evaluate the effectiveness of amnioinfusion during labor, for reducing TDA in deliveries complicated with repeated VD.

**Study Design**: Our departmental protocol indicates to perform amnioinfusion after 30 minutes of repeated VD, if decelerations continue despite position changes. Electronic fetal monitoring of deliveries with repeated VD that underwent amnioinfusion, during a period of one year, were analyzed. TDA was calculated, for each patient, both 30 minutes before and after the initiation of amnioinfusion. TDA was calculated as the sum of the area under the curve for each deceleration using the formula: (heart rate change in BPM*time in seconds)/2.

**Results**: A total of 131 deliveries were included for analysis, 44.3% resulted in vaginal deliveries, 22.1% in vacuum extractions and 33.6% in cesarean deliveries (CD). Patients who had CD had higher rates of nulliparity (*p* = 0.004), meconium (*p* = 0.017) and fever (*p* = 0.034). Median TDA before amnioinfusion was 8,430 (5,305–14,321), compared to 3,591 (1,297–7,371) after amnioinfusion, *p* < 0.001. Amnioinfusion led to a 57.4% reduction in median TDA, regardless of the presence of cord around the neck/body. In a subgroup analysis of patients with normal amniotic fluid index upon admission to labor (*n* = 120), amnioinfusion decreased TDA as well [median TDA 9,189 (5,322–14,774) before amnioinfusion and 3,621 (1,321–7,581) after, *p* < 0.001]. There was a notable trend of lower rates of meconium, CD and umbilical cord pH < 7.1 in patients with a larger TDA change following amnioinfusion.

**Conclusion**: Amnioinfusion effectively reduces TDA in labors complicated with repeated VD and can be used to alleviate intrapartum fetal distress. Larger cohorts are needed to evaluate the correlation between TDA change following amnioinfusion and obstetric outcomes.

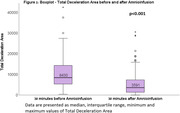

## 157 | Second stage duration >4 hours‐ does it matter? An evaluation of nulliparous with epidural anesthesia

Gal cohen^1^, Tal Biron‐Shental^2^, Hila Shalev‐Ram^2^, Gil Shechter Maor^3^, Michal Kovo^3^, Hanoch Schreiber^3^

^1^Meir Hospital, Meir Hospital, HaMerkaz; ^2^Meir Medical Center, Meir Medical Center, HaMerkaz; ^3^Meir Medical Center, Kfar Saba, HaMerkaz

17:15–18:30

**Objective**: The current guidelines allow a duration of up to 3 hours for the second stage of labor without epidural analgesia and 4 hours with epidural analgesia for nulliparous, provided there are satisfactory maternal and fetal conditions and documented labor progress. We aimed to evaluate the obstetric outcomes of nulliparous with epidural who exceeded the 95^th^ percentile duration of 4 hours.

**Study Design**: This retrospective cohort study included all term singleton deliveries of nulliparous with epidural analgesia who had a second stage duration of >3 hours, between 2014‐2021. Maternal and neonatal outcomes were evaluated by comparing two second stage durations: 3‐4 hours vs >4 hours.

**Results**: A total of 2,798 deliveries were included, with 2,273 in the 3‐4 hours group (mean duration of 3.42 ± 0.28 hours) and 525 in the >4 hours group (mean duration 4.38 ± 0.42 hours). Compared to the 3‐4 hours group, the >4 hours group had lower rates of vaginal delivery (80.4% vs 93%, *p* < 0.001), relatively higher rates of vacuum assisted delivery (VAD) (56.0% vs 42.4%, *p* < 0.001) and higher rates of cesarean delivery (CD) (19.6% vs 7.0% respectively, *p* < 0.001). The >4 hours group had higher rates of macrosomia and large for gestational age birthweights (7.0% vs 4.0%, *p* < 0.003 and 11.8 vs 8.9%, *p* = 0.039 respectively). Shoulder dystocia, neonatal subgaleal hematoma and failed VAD were more common in the >4 hours group (2.7% vs 1.1%, 9.0% vs 3.3% and 4.4% vs 1.0% respectively, *p* < 0.01 for all). Neonatal Apgar scores, cord pH and NICU admission rates were similar between the groups. PPH was more common among the >4 hours group (*p* = 0.002). Multivariable logistic regression analysis adjusted for confounders including obesity, hypertensive disorders, diabetes, macrosomia and mode of delivery, revealed that a duration of >4 hours was associated with increased risks for shoulder dystocia, subgaleal hematoma and failed VAD.

**Conclusion**: Most women achieve vaginal delivery even after a duration of >4 hours. However, it is associated with increased risks of neonatal birth trauma and failed VAD.

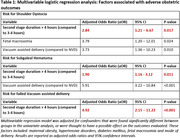

## 158 | Mode of conception and long‐term infectious morbidity of the offspring; a sibling analysis

Noam Dadon^1^, Eyal Sheiner^2^, Tamar Wainstock^2^, Gil Gutvirtz^3^, atif zeadna^4^, Naama Steiner^2^, Dvora Kluwgant^5^, Gali Pariente^2^

^1^Soroka University Medical Center, Beer‐sheva, Israel, soroka medical center, Beer‐sheva, HaDarom; ^2^Department of Obstetrics and Gynecology, Soroka University Medical Center, Ben‐Gurion University of the Negev, Beer‐Sheva, Israel., Beer Sheva, HaDarom; ^3^Soroka University Medical Center, Metar, HaDarom; ^4^IVF Unit, Department of Obstetrics and Gynecology, Soroka University Medical Center, Ben‐Gurion University of the Negev, Beer‐Sheva, Israel, Beer‐sheva, HaDarom; ^5^NYPBrooklyn Methodist Hospital–‐ Brooklyn, NY, Brooklyn, New York

17:15–18:30

**Objective**: The aim of the current study was to investigate the association between mode of conception, i.e. using fertility treatments vs. spontaneously conception, and long‐term infectious morbidity of the offspring while employing sibling matched analysis to maximize confounder control.

**Study Design**: A retrospective population‐based cohort study was performed, including all sibling deliveries between 1991 and 2021 at a tertiary medical center. Offspring were followed until the age of 18. A Kaplan–Meier survival curve was used to compare the cumulative incidence of childhood infectious morbidity, and a multivariable Cox survival hazards regression model was used to control for confounders.

**Results**: During the study period, 14,336 siblings met the inclusion criteria, of which 7,711 (53.8%) were conceived through fertility treatments and 6,625 (46.2%) were conceived spontaneously. A significant association was found between siblings born following fertility treatments and childhood infectious morbidity as compared with those conceived spontaneously (29.9% vs. 27.8%, *p* = 0.014, *Table*). Nevertheless, the Kaplan Meier survival curve did not demonstrates significantly higher cumulative incidence rates of long‐term infectious morbidity off the offspring born following fertility treatment (Log‐Rank *p* = 0.614). Likewise, using a Cox proportional hazards model, adjusted for maternal age, gestational age at birth and follow‐up time, being born following fertility treatments was not independently associated with long‐term infectious morbidity of the offspring (adjusted HR 0.92; 95% CI 0.82‐1.00; *p* = 0.063).

**Conclusion**: Using a sibling analysis, fertility treatment does not appear to be an independent risk factor for long‐term infectious morbidity of the offspring up to 18 years of age.

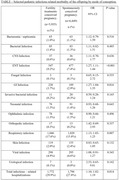

## 159 | Oxytocin vs Carbetocin for the prevention of post‐partum haemorrhage: An individual participant data meta‐analysis

Madeline F. Flanagan^1^, Ben W. Mol^2^, Malitha patabendige^2^, Arsheeya Rattan^2^, George Attilakos^3^, Agnes Fenix^4^, Helen Van Der Nelson^5^, Luis Torres‐Gomez^6^, Sofus Rabow^7^, Tripop Lertbunnaphong^8^, Osvaldo Reyes^9^, Carole‐Anne Whigham^10^, Leiv Arne Rosseland^11^, Wentao Li^2^

^1^Department of Obstetrics and Gynaecology, Monash University, St Kilda, Victoria; ^2^Monash University, Clayton, Victoria; ^3^Directorate of Women's and Children's Health, Southmead Hospital, North Bristol NHS Trust, Bristol, England; ^4^Obstetrics and Gynecology, Cardinal Santos Medical Center, Calamba, Laguna; ^5^2 Directorate of Women's and Children's Health, Southmead Hospital, North Bristol NHS Trust, Bristol, England; ^6^4 Servicio de Ginecología y Obstetricia, Hospital General de Subzona 13, Instituto Mexicano del Seguro Social, Acuna, Coahuila de Zaragoza; ^7^Institution of Clinical Sciences Malmo, Department of Obstetrics and Gynaecology, Skane University Hospital, Lund University, Malmo and Lund, Skane Lan; ^8^Department of Obstetrics and Gynaecology, Faculty of Medicine Siriraj Hospital, Mahidol University, Bangkok, Krung Thep; ^9^Department of Obstetrics and Gynaecology, Saint Thomas Maternity Hospital, Panama City, Panama; ^10^Department of Obstetrics and Gynaecology, Monash Health, Clayton, Victoria; ^11^8 Faculty of Medicine, Institute of Clinical Medicine, University of Oslo, Oslo, Oslo

17:15–18:30

**Objective**: To compare the efficacy of oxytocin versus carbetocin for the prevention of PPH through individual participant data (IPD) meta‐analysis (MA). To evaluate the quality and trustworthiness of Randomised Controlled Trials (RCTs) comparing oxytocin and carbetocin for the prevention of PPH with the Trustworthiness in RAndomised Controlled Trials (TRACT) tool. To compare the results of IPD MA with aggregate data MA of RCTs ranked as low versus high risk for data integrity concerns.

**Study Design**: This study used IPD‐MA for all trial data received. Aggregate data MA was used when trial data was unavailable or trialists were uncontactable. We searched randomised controlled trials(RCTs) comparing systemic oxytocin and systemic carbetocin used to prevent PPH in labouring women. Authors of eligible trials were invited to contribute their IPD. The main outcomes were estimated blood loss(EBL) and occurrence of PPH(EBL ≥500mls). For RCTs that did not share IPD, we assessed trustworthiness using the reason not to share data and the TRACT data integrity checklist.

**Results**: We identified 49 RCTs, of which 16 contributed IPD(*n* = 3093 oxytocin; *n* = 3093 carbetocin). From the 33 RCTs that did not share IPD, we classified 8 as ‘trustworthy’. Studies sharing IPD and ‘trustworthy’ studies not sharing IPD showed carbetocin decreased EBL by 40mls[55.26, 24.62], and in RCTs that did not meet ‘trustworthy’ criteria carbetocin decreased EBL by 86 mls[96.73, 74.81]. Regarding PPH≥500mls, IPD and ‘trustworthy’ studies showed that carbetocin did not decrease the risk of PPH(OR = 0.99, [0.94,1.05]), whereas RCTs that did not meet the ‘trustworthy’ criteria demonstrated that carbetocin decreased the risk of PPH by 36%(OR = 0.64, [0.49, 0.82]).

**Conclusion**: We conclude from studies sharing IPD and trustworthy studies not sharing IPD that oxytocin and carbetocin are equivalent in preventing PPH. The fact that studies not sharing IPD that did not fulfill the trustworthiness criteria showed different results should be a reason for concern.

## 160 | Oxytocin vs Misoprostol for the prevention of postpartum haemorrhage: An individual participant data meta‐analysis

Madeline F. Flanagan^1^, Ben W. Mol^2^, Malitha patabendige^2^, Arsheeya Rattan^2^, Shiva Sridhar^2^, Ester Atukunda^3^, Ritwik Samanta^4^, Daljit Sahota^5^, Enrique Teran^6^, Vanita Jain^7^, Munir‐deen Ijaiya^8^, Wentao Li^2^

^1^Department of Obstetrics and Gynaecology, Monash University, St Kilda, Victoria; ^2^Monash University, Clayton, Victoria; ^3^Mbarara University of Science and Technology, Mbarara, Mbarara; ^4^3 Department of Obstetrics and Gynaecology, National Health Mission, DHFW&S, West Bengal, West Bengal; ^5^The Chinese University of Hong Kong, Hong Kong, Not Applicable; ^6^Colegio de Ciencias de Salud, Universidad San Francisco de Quito, Quito, Pichincha; ^7^6 Department of Obstetrics and Gynecology, Postgraduate Institute of Medical Education and Research, Chandigarh, Chandigarh; ^8^7 Department of Obstetrics and Gynecology, Federal Medical Centre, Lokoja, Kogi

17:15–18:30

**Objective**: To compare the effectiveness of oxytocin and misoprostol for the prevention of PPH through individual participant data(IPD) meta‐analysis. To examine the trustworthiness of RCTs comparing oxytocin and misoprostol.

**Study Design**: We used an Independent Participant Data (IPD) and aggregate data Meta‐Analysis (MA) study design. We searched randomised controlled trials(RCTs) comparing systemic oxytocin and systemic misoprostol used to prevent PPH in labouring women. Authors of eligible trials were invited to contribute their IPD. The main outcome was PPH(EBL ≥500mls) and severe PPH(EBL≥1000mls). Secondary outcomes were EBL, haemoglobin (Hb) drop and requirement for blood transfusion. For RCTs that did not share IPD, we assessed trustworthiness using the reason not to share data and the TRACT data integrity checklist and performed aggregate data MA.

**Results**: We identified 79 RCTs, of which 10 contributed IPD(*n* = 1110 oxytocin; *n* = 1113 misoprostol). From the 69 RCTs that did not share IPD, we classified 26 as ‘trustworthy’. Studies sharing IPD showed oxytocin decreased average EBL by 32mls[20.22, 44.10], while in trustworthy studies not sharing IPD misoprostol decreased EBL by 45mls[‐100.06, 9.14], and in RCTs that did not meet ‘trustworthy’ criteria misoprostol increased EBL by 54 mls[26.82, 81.11]. Regarding PPH ≥500mls, IPD and ‘trustworthy’ studies showed oxytocin decreased the risk of PPH by 40 and 48%, respectively(RR = 1.4[1.13,1.73]; RR = 1.48[1.38,1.60]), versus a 6% decrease in RCTs that did not meet ‘trustworthy’ criteria(RR = 0.94[0.82,1.08].

**Conclusion**: We conclude from studies sharing IPD and trustworthy studies not sharing IPD that oxytocin is more effective than misoprostol in preventing PPH. The fact that studies not sharing IPD that did not fulfill the trustworthiness criteria showed different results should be a reason for concern.

## 161 | A positive association between postpartum electrohysterography parameters and blood loss after vaginal delivery

Marion Frenken^1^, Daisy van der Woude^1^, Bettine van Willigen^2^, Jeanne Dieleman^1^, S. Guid Oei^1^, Judith O.E.H. van Laar^3^

^1^Máxima Medical Centre, Veldhoven, Noord‐Brabant; ^2^Máxima Medical Centre, Eindhoven, Noord‐Brabant; ^3^Máxima Medical Centre, Veldhoven, The Netherlands, Veldhoven, Noord‐Brabant

17:15–18:30

**Objective**: The postpartum period can be complicated by hemorrhage, frequently caused by uterine atony. Electrohysterography, allowing continuous monitoring of uterine activity, may be a promising alternative for early detection of uterine atony, and thereby contribute to the prevention of postpartum hemorrhage. Associations between electrohysterographic parameters postpartum and total blood loss were studied.

**Study Design**: In this prospective explorative study, women were included with a vaginal delivery between 36+0 and 42+0 weeks of gestation. Linear regression analysis was used to describe the association between electrohysterographic parameters (i.e. area under the contraction curve (AUC) total (in arbitrary units), AUC from baseline (in arbitrary units), maximum amplitude (in arbitrary units) and baseline tone (in arbitrary units)) and total blood loss (in mL) during the first 30 minutes postpartum.

**Results**: In total, 25 women were included for analysis, of whom three had postpartum hemorrhage. A moderate positive linear correlation was found between total blood loss and the AUC total (r = 0.44, *p* = 0.03), AUC from baseline (r = 0.43, *p* = 0.03) and baseline tone (r = 0.43, *p* = 0.03). There was no significant linear correlation between total blood loss and maximum amplitude. Explorative analysis revealed a significantly higher AUC total, AUC from baseline and baseline tone in women with postpartum hemorrhage compared to women without postpartum hemorrhage. (*p* = 0.02, *p* = 0.02 and *p* = 0.03, respectively).

**Conclusion**: There is a moderate positive correlation between total blood loss and the AUC total, AUC from baseline and baseline tone. Also, these parameters were all significantly higher in women with postpartum hemorrhage compared to women without postpartum hemorrhage.

## 162 | Risk of cesarean section following immediate vs. delayed induction of labor after external cephalic version

Rachel L. Friedlander^1^, Nathan Gill^2^, Susan Gerber^2^

^1^Northwestern Feinberg School of Medicine, Chicago, Illinois; ^2^Northwestern University Feinberg School of Medicine, Northwestern Feinberg School of Medicine, Illinois

17:15–18:30

**Objective**: In the United States, approximately 3‐4% of term pregnancies are in breech presentation, and the vast majority are delivered by cesarean section. While external cephalic version (ECV) reduces the risk of cesarean section when successful, there is variability in practice regarding timing of delivery following a successful ECV. This study aimed to assess the difference in cesarean section rates between patients whose labors were induced immediately following ECV vs. those whose labors were induced at a delayed interval.

**Study Design**: A retrospective analysis from a single high‐volume academic institution was conducted, including all pregnant people with singleton, viable, non‐anomalous gestations, and successful ECV followed by induction of labor at term. A multivariable logistic regression analysis was used to model the odds of a c‐section given immediate vs. delayed IOL following ECV. Delayed IOL was defined as IOL that began >24 hours after ECV. The model adjusted for parity (nulliparous vs. multiparous), reason for induction (medical vs. elective), BMI, maternal age, and birthweight (AGA, SGA, macrosomia).

**Results**: 393 patients were included in the study, 252 underwent delayed IOL after ECV, and 141 underwent immediate IOL. Overall, the population had a mean age of 34.4 years, parity of 0.9, and BMI of 30.8. Baseline characteristics between groups did not differ clinically. The majority of neonates were AGA (84.0%). 24.8% of those who underwent immediate IOL delivered via c‐section versus 23.4% in the delayed group. There was a 78% increase in the odds of undergoing c‐section associated with immediate IOL vs. elective IOL after controlling for covariates, however this was not statistically significant (OR 1.78, *p* = 0.06, 95% CI (0.99, 3.25)).

**Conclusion**: Immediate IOL following successful ECV was independently associated with a trend towards increased cesarean section risk when compared to delayed IOL following ECV. Further research should be performed on how ECV may affect labor mechanics and possible labor dystocia.

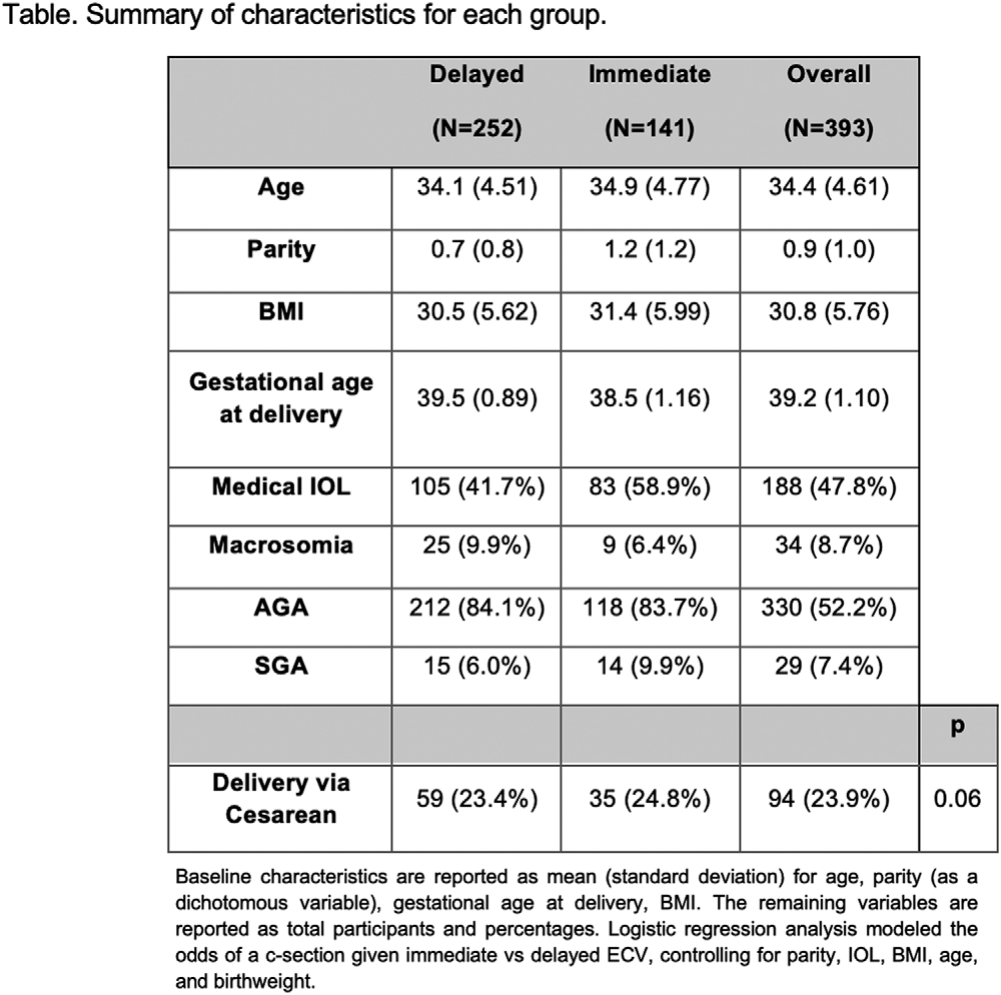

## 163 | A comparison of complications and surgery outcomes in skin closure methods following cesarean sections

Daniel Gabbai^1^, Chen Jacoby^2^, Itamar Gilboa^1^, Lee Reicher^3^, Anat Lavie^4^, Yariv Yogev^5^, Emmanuel Attali^1^

^1^Lis Hospital for Women's Health, Tel Aviv Sourasky Medical Center, Tel‐Aviv, Tel Aviv; ^2^Lis Hospital for Women's Health, Tel Aviv Sourasky Medical Center, Tel Aviv University, Tel Aviv, Israel., Tel Aviv, HaMerkaz; ^3^Weizmann Instutute Of Science, Weizmann Institute of Science, HaMerkaz; ^4^Tel Aviv Sourasky Medical Center, Tel Aviv Sourasky Medical Center, Tel Aviv; ^5^Lis Maternity Hospital, Sourasky Medical Center, Tel Aviv University, Tel Aviv Sourasky Medical Center, Tel Aviv

17:15–18:30

**Objective**: To assess and compare complication rates of three different methods for skin closure in the cesarean section.

**Study Design**: We conducted a retrospective cohort study, in a single tertiary university‐affiliated medical center between (January 2011‐April 2022). In 2020, a new technique for skin closures using absorbable subcutaneous staples was implemented in our center. A comparison was made between three groups of skin closures: non‐absorbable staples (NAS), absorbable subcutaneous staples (ASSt), and absorbable subcutaneous sutures (ASSu).

**Results**: 1) During the study period, 31,660 cesarean sections (CS) were performed in our center. The data of 30848 CS were available for analysis.

2) The most common technique used for skin closures was non‐absorbable staples 22,121 women (71.71%) followed by absorbable subcutaneous staples 7,333 women (23.77%) and absorbable subcutaneous sutures). in 1394 women (4.52%)

3) ASSt was associated with a significantly shorter surgery time in comparison to NAS and ASSu (52 min vs 53 min vs 60 min, *p* < 0.001).

4) No differences were found in rates of wound infections, wound‐related surgery, or any surgical site surgery in the 45 days following CS.

4) In a multivariate analysis: the use of ASSt was associated with a significantly lower risk for prolonged hospitalization > 5 days (OR 0.57, *p* < 0.001) and re‐admission within 45 days (OR 0.723, *p* = 0.002).

**Conclusion**: Absorbable subcutaneous staples for skin closure during CS are associated with a shorter surgery time and a lower risk for prolonged hospitalization and readmission within 45 days.

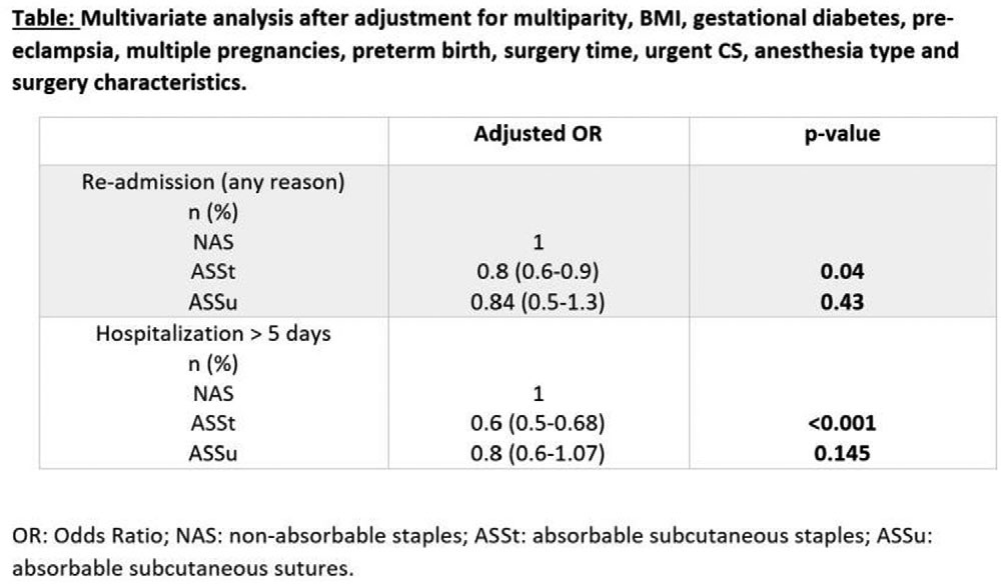

## 164 | Change in delivery outcomes following the introduction of a novel electronic partogram board control

Daniel Gabbai^1^, Anat Lavie^2^, Yoav Baruch^3^, Sharon Maslovitz^4^, Lee Reicher^5^, Yariv Yogev^6^, Emmanuel Attali^1^

^1^Lis Hospital for Women's Health, Tel Aviv Sourasky Medical Center, Tel‐Aviv, Tel Aviv; ^2^Tel Aviv Sourasky Medical Center, Tel Aviv Sourasky Medical Center, Tel Aviv; ^3^Lis Maternity Hospital, Sourasky Medical Center, Tel Aviv University, Israel, Tel Aviv, Tel Aviv; ^4^tel aviv medical center, tel aviv, Tel Aviv; ^5^Weizmann Instutute Of Science, Weizmann Institute of Science, HaMerkaz; ^6^Lis Maternity Hospital, Sourasky Medical Center, Tel Aviv University, Tel Aviv Sourasky Medical Center, Tel Aviv

17:15–18:30

**Objective**: In 2019, we introduced an electronic board control, incorporating modified bishop score parameters, amniotic fluid status (including color), oxytocin infusion rate, and second stage duration. We assessed the impact of this introduction on obstetrical practices and perinatal outcomes.

**Study Design**: In a retrospective cohort study at a tertiary university‐affiliated medical center with 17 active delivery rooms, we compared two groups: the pre‐introduction group (01/2012‐06/2019) and the post‐introduction group (01/2021‐12/2022). We excluded planned cesarean deliveries and births that occurred between 7/2019‐12/2020, considered as implantation period.

We assessed differences using univariate and multivariate analyses including the Cochrane‐Armitage test for trend examination. We used the years 2012‐2019 as predictors applied to the complete dataset to evaluate predicted probabilities versus actual rates. Results were visually presented through probability graphs.

The primary outcome was the time interval from transfer to the delivery room until delivery. Secondary outcomes were defined as rate of intrapartum fever, prolonged second stage of labor, operative deliveries, perineal laceration including obstetric anal sphincter injury; and 5‐minute APGAR score < 7.

**Results**: (1) During the study period, 90,530 women attempted labor, with 78.34% (70,922) before the board's introduction and 21.66% (19,608) after.

(2) After multivariate analysis, the post‐introduction group had positive outcomes: reduced time in the delivery ward (32.9% vs. 38%, adjusted Odd Ratio (aOR) 0.67 [0.6‐0.7], *p* < 0.001), lower rates of prolonged second stage (5.4% vs. 7.1%, aOR 0.64 [0.5‐0.7], *p* < 0.0001), and fewer perineal injuries (28.6% vs. 34.2%, aOR 0.8 [0.7‐0.9], *p* < 0.001). No differences in operative delivery and 5‐minute APGAR Score < 7 were found.

**Conclusion**: An electronic continuous partogram board is an efficient tool for active labor management, optimizing labor ward utilization time, and reducing prolonged second stage of labor and perineal injuries, without increasing operative deliveries or adverse neonatal outcomes.

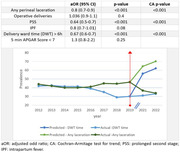

## 165 | Risk factors for prolonged hospitalization following vacuum‐assisted delivery

Daniel Gabbai^1^, Lee Reicher^2^, Itamar Gilboa^1^, Anat Lavie^3^, Yariv Yogev^4^, Emmanuel Attali^1^

^1^Lis Hospital for Women's Health, Tel Aviv Sourasky Medical Center, Tel‐Aviv, Tel Aviv; ^2^Weizmann Instutute Of Science, Weizmann Institute of Science, HaMerkaz; ^3^Tel Aviv Sourasky Medical Center, Tel Aviv Sourasky Medical Center, Tel Aviv; ^4^Lis Maternity Hospital, Sourasky Medical Center, Tel Aviv University, Tel Aviv Sourasky Medical Center, Tel Aviv

17:15–18:30

**Objective**: We aimed to determine risk factors for prolonged hospitalization following vacuum‐assisted delivery (VAD).

**Study Design**: A retrospective cohort study, in a single, university‐affiliated tertiary medical center with approximately 12,500 deliveries annually (2012‐2022) was conducted Standard practice in our department entails a post‐vaginal delivery hospital stay of 48 to 72 hours. Length of stay following a VAD was analyzed and parturients who underwent VAD were categorized into two groups by length of hospitalization (more and less than 7 days). Maternal and neonatal characteristics were compared. Risk factors for prolonged hospitalization were examined using univariate and multivariate logistic regression.

**Results**:

1. Overall, 133,035 deliveries occurred during the study period. Of them, 9,440 (7.1%) were by VAD.
2. The post‐partum hospitalization period was distributed as follows: 9134 (96.76%) women were hospitalized for up to 7 days, and 306 (3.34%) were hospitalized for more than 7 days.
3. Using multivariate analysis: preterm delivery (delivery prior to 37 weeks) (RR 5.4 95% CI [1.6‐17], *p* = 0.005); preeclampsia (RR 6.0 95% CI [1.9‐19], *p* = 0.002), induction of labor (RR 3.4 95% CI [1.8;6.3], *p* < 0.001), obstetric anal sphincter injury (RR 3.2 95% CI [1.2‐8], *p* = 0.013), postpartum hemorrhage which required blood transfusion (RR 3.3 95% CI [1.3‐8.4], *p* = 0.012), and Apgar Score under 7 at minute 5 (RR 3.4 95% CI [1.4‐39], *p* = 0.015), were identified as independent risk factors for prolonged hospitalization [Table]

**Conclusion**: Risk factors associated with prolonged hospitalization following a VAD can be identified, with preterm delivery and Apgar score < 7 at minute 5 being the most prominent.

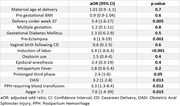

## 166 | Expectant management of preeclampsia with severe features: Comparing singleton and twin pregnancies

Alison N. Goulding^1^, Josef Jackson^1^, Felixnando Rubio^2^, Jeevitha Patil^3^, Steven Clark^4^, Mary C. Tolcher^5^

^1^Baylor College of Medicine, Houston, TX; ^2^University of Colorado, Denver, Colorado; ^3^Roswell OBGYN, LLC, Roswell, Georgia; ^4^Baylor College of Medicine, Houston, Texas; ^5^Baylor College of Medicine, Baylor College of Medicine, TX

17:15–18:30

**Objective**: To compare baseline characteristics and outcomes for singleton and twin pregnancies expectantly managed for preeclampsia with severe features diagnosed prior to 34 weeks.

**Study Design**: Retrospective cohort study from two hospitals within our academic institution including pregnant people admitted for preeclampsia with severe features between 24 0/7 and 33 6/7 weeks gestation from January 2015 to December 2019. Exclusion criteria included higher order multiples, preterm premature rupture of membranes, labor, intrauterine fetal demise, or major fetal anomaly. Baseline characteristics and clinical outcomes were compared between singleton and twin pregnancies using standard statistical methods.

**Results**: 407 deliveries were identified as preeclampsia with severe features, 220 (54%) of which were expectantly managed and 30 (7%) of which were twins. Twins were less likely to be expectantly managed compared to singletons (37 vs 56%, *p* = 0.04). Baseline clinical characteristics did not differ significantly between twin and singleton pregnancies, aside from twin pregnancies having lower median hemoglobin levels at time of preeclampsia diagnosis (10.4 vs 11.8 grams/deciliter, *p* = 0.001), and twin pregnancies being more likely to present with right upper quadrant or epigastric pain (18 vs 4%, *p* = 0.03). Among outcomes, only postpartum hemorrhage was more likely among twin pregnancies (55 vs 18%, *p* = 0.003); other clinical outcomes did not differ significantly.

**Conclusion**: Despite similar baseline characteristics for singleton and twin pregnancies diagnosed with preeclampsia with severe features prior to 34 weeks, twin pregnancies were significantly less likely to be managed expectantly. Those twin pregnancies that were managed expectantly had similar clinical outcomes, aside from known increased risk of postpartum hemorrhage associated with twin physiology. These data suggest that unconscious bias involving a subjective discomfort with twin pregnancies makes clinicians less likely to manage twin pregnancies conservatively in the setting of preeclampsia with severe features.

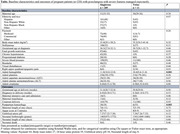

## 167 | Risk factors for relaparotomy after cesarean delivery

Natav Hendin^1^, Liron Seidman^2^, Yossi Geron^1^, Gil Zeevi^3^, Eran Hadar^1^, Asnat Walfisch^4^, Ohad Houri^1^

^1^Rabin Medical Center, Petach Tikva, HaMerkaz; ^2^Rabin medical center, Rabin medical center, Israel, HaMerkaz; ^3^Rabin Medical Center, Rabin Medical Center, Tel Aviv; ^4^Helen Schneider Hospital for Women, Rabin Medical Center, Petah Tikva, HaMerkaz

17:15–18:30

**Objective**: To comprehensively identify and analyze risk factors associated with relaparotomy following cesarean delivery (CD), focusing on obstetric and surgical parameters.

**Study Design**: Retrospective study conducted at a high‐volume tertiary obstetric center. All women who underwent CD between 2013‐2023 were included. Patient data were systematically reviewed to identify potential risk factors contributing to the need for relaparotomy.

**Results**: Out of 11,465 cases, 59 participants (0.5%) required relaparotomy after the primary CD. While maternal characteristics like age, BMI, parity, previous CD, number of CD and preeclampsia showed no differences (Table 1), the relaparotomy group exhibited significantly higher rates of preterm delivery (37.29% vs. 19.46%, *p* < 0.001) and specific CD indications, including placenta previa (6.78% vs. 1.77%, OR 4.48, 95% CI 1.69‐11.87, *p* = 0.002) and multiple gestations (16.95% vs. 6.44%, OR 3.08, 95% CI 1.57‐6.02, *p* = 0.001).

Moreover, factors associated with the nature and course of the CD itself, such as emergency CD (52.54% vs. 30.99%, OR 2.46, 95% CI 1.48‐4.09, *p* = 0.0005) and surgeries exceeding one hour (18.64% vs. 8.18%, OR 2.57, 95% CI 1.34‐4.92, *p* = 0.004) were significantly correlated with relaparotomy. Higher incidence of adhesions (44.07% vs. 12.06%, OR 5.77, 95% CI 3.46‐9.63, *p* < 0.0001) and excessive bleeding >1 liter (15.25% vs. 1.92%, OR 10.08, 95% CI 4.95‐20.54, *p* < 0.0001), as well as additional intraoperative bilateral tubal ligation (6.78% Vs. 2.35%, OR 3.36, 95% CI 1.27‐8.87, *p* = 0.014) and the necessity for additional sutures during uterine incision closure (89.83% Vs. 73.60%, OR 4.95, 95% CI 1.68‐14.61, *p* = 0.004) were noted in cases requiring relaparotomy.

**Conclusion**: Our study underscores the multifactorial nature of relaparotomy after cesarean delivery, emphasizing the significance of considering a broad array of risk factors. By identifying and understanding these factors, clinicians can optimize patient care.

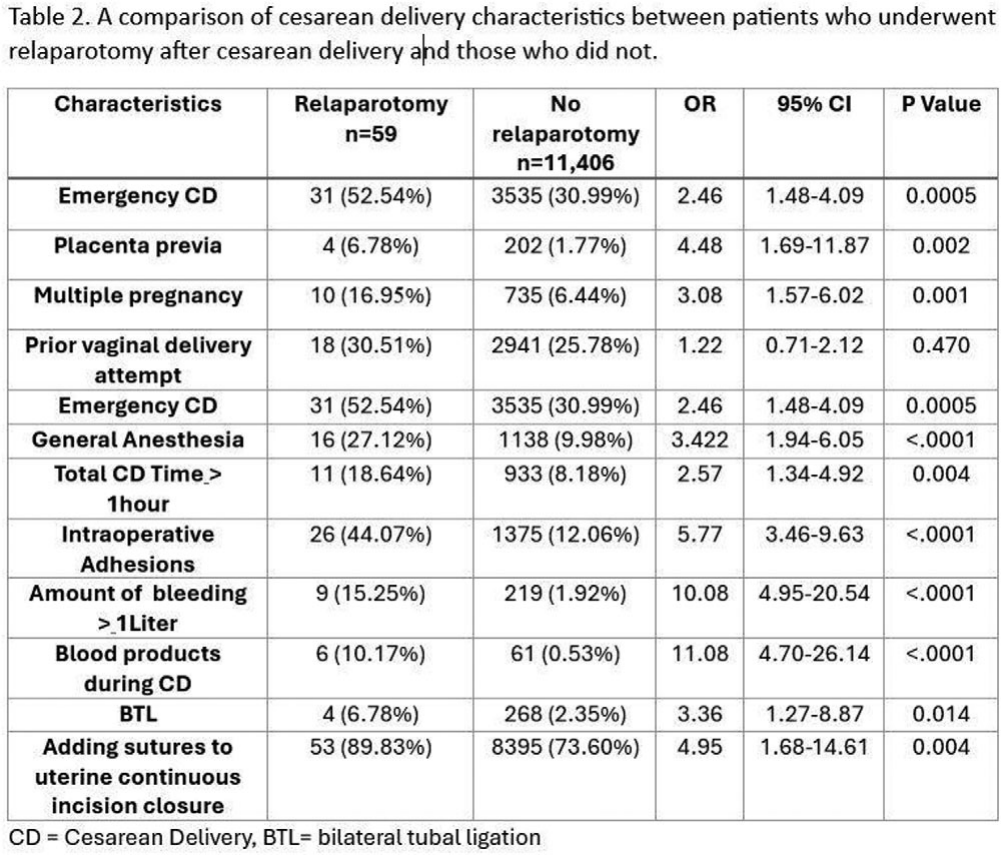

## 168 | Clinical characteristics and pregnancy outcomes in pregnancies at‐risk of HDFN in Sweden, Finland, and Denmark

Kelvin Ho Man Kwok^1^, Shengxin Liu^2^, Mette Østergaard Thunbo^3^, Mika Gissler^4^, Malin Almgren^2^, Agneta Wikman^5^, Lars Henning Pedersen^3^, May Lee Tjoa^6^, Elizabeth C. Hsia^7^

^1^Real‐world Evidence, Schain Research AB, Stockholm, Stockholms Lan; ^2^Real‐world Evidence, Schain Research AB, Stockholm, Sodermanlands Lan; ^3^Department of Clinical Medicine, Aarhus University, Aarhus, Midtjylland; ^4^Finish Institute for Health and Welfare, Helsinki, Uusimaa; ^5^Clinical Immunology and Transfusion Medicine, Karolinska University Hospital, Stockholm, Sodermanlands Lan; ^6^Janssen Global Services, LLC, Cambridge, Massachusetts; ^7^Janssen Research & Development, LLC, Spring House, PA

17:15–18:30

**Objective**: Maternal alloimmunization against fetal red blood cells may result in hemolytic disease of fetus and newborn (HDFN), a potentially life‐threatening condition. This study characterized maternal clinical characteristics and pregnancy outcomes in pregnancies at risk of HDFN in Sweden, Finland, and Denmark.

**Study Design**: Using nationwide population‐based registers from 2000‐2021 in Sweden and Finland and 1997‐2018 in Denmark, women who had ≥1 pregnancy that was monitored or treated for alloimmunization (ICD‐10 O36.0‐O36.2) or birthed ≥1 child with a postnatal diagnosis of HDFN‐related conditions (P55.0, P55.8‐P55.9, P56.0, P57.0) were identified. Complete, singleton pregnancy records of these women were included in this study, except when the maternal age at delivery was below 15 years. The pregnancies with an alloimmunization code or child with postnatal diagnosis code of HDFN were categorized as HDFN pregnancies, while the others were categorized as non‐HDFN pregnancies. Descriptive analyses were performed to compare maternal characteristics and pregnancy outcomes in HDFN versus non‐HDFN pregnancies.

**Results**: The study included a total of 14,732 singleton pregnancies from Sweden, 5,863 from Finland, and 11,964 from Denmark. Of these pregnancies, 7,391 (50%) in Sweden, 2,885 (49%) in Finland, and 6,150 (51%) in Denmark were HDFN pregnancies. As expected, a higher proportion of HDFN pregnancies were multiparous (≥2), which was consistent across countries. Both emergency and elective cesarean delivery were more frequent in HDFN pregnancies. No difference in stillbirth rate was observed between HDFN and non‐HDFN pregnancies. Maternal age and perinatal complications appeared similar in HDFN and non‐HDFN pregnancies (**Table 1**).

**Conclusion**: Apart from maternal parity and cesarean delivery, no maternal characteristics were identified to be strongly associated with HDFN. Future studies are warranted to investigate if there are other maternal characteristics associated with pregnancy outcomes in HDFN.

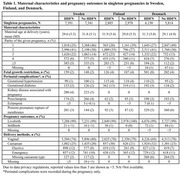

## 169 | Retrospective analysis of neonatal outcomes in pregnancies at‐risk of HDFN in Sweden, Finland, and Denmark

Kelvin Ho Man Kwok^1^, Shengxin Liu^2^, Mette Østergaard Thunbo^3^, Mika Gissler^4^, Malin Almgren^2^, Agneta Wikman^5^, Lars Henning Pedersen^3^, May Lee Tjoa^6^, Elizabeth C. Hsia^7^

^1^Real‐world Evidence, Schain Research AB, Stockholm, Stockholms Lan; ^2^Real‐world Evidence, Schain Research AB, Stockholm, Sodermanlands Lan; ^3^Department of Clinical Medicine, Aarhus University, Aarhus, Midtjylland; ^4^Finish Institute for Health and Welfare, Helsinki, Uusimaa; ^5^Clinical Immunology and Transfusion Medicine, Karolinska University Hospital, Stockholm, Sodermanlands Lan; ^6^Janssen Global Services, LLC, Cambridge, Massachusetts; ^7^Janssen Research & Development, LLC, Spring House, PA

17:15–18:30

**Objective**: Maternal alloimmunization against fetal red blood cell antigens may lead to severe HDFN, a potentially life‐threatening condition that may require invasive treatments in the fetus and neonate. This study characterized neonatal outcomes in pregnancies at risk of HDFN in Sweden, Finland, and Denmark.

**Study Design**: Using nationwide, population‐based registers from 2000‐2021 in Sweden and Finland, and 1997‐2018 in Denmark, women who had ≥1 pregnancy with alloimmunization diagnosis (ICD‐10 O36.0‐O36.2) or birthed ≥1 child with a postnatal diagnosis of HDFN‐related conditions (P55.0, P55.8‐P55.9, P56.0, P57.0) were identified. Complete singleton pregnancy records were included except when maternal age at delivery was < 15 years. Pregnancies with an alloimmunization code or child with postnatal HDFN diagnosis code during follow‐up were categorized as HDFN pregnancies, while the others were categorized as non‐HDFN pregnancies. Neonatal outcomes and pre‐/postnatal treatments received were examined in liveborn neonates from HDFN and non‐HDFN pregnancies. Neonates with HDFN were grouped by the most burdensome treatment (ie, IUT > blood/exchange transfusion > phototherapy or unknown).

**Results**: 7,289 infants in Sweden, 2,849 in Finland, and 6,076 in Denmark had HDFN (**Table 1**). Of these, 27% in Sweden, 38% in Finland, and 12% in Denmark received treatments (prenatal IUT, transfusion, phototherapy); IUT was not reported in Finland. Higher percentages of preterm birth, lower mean birth weights/lengths, and higher neonatal unit admission rates were observed in the IUT and transfusion groups. The transfusion group had the lowest mean 5‐minute Apgar scores and highest neonatal unit admission rate. These patterns were consistent across countries.

**Conclusion**: These population‐based data suggest that neonates with HDFN in Sweden, Finland, and Denmark, especially those treated with transfusion(s), have poorer outcomes compared to neonates without HDFN. These results support that there is still unmet need in the management of HDFN, leading to potentially severe neonatal outcomes.

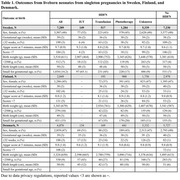

## 170 | Low Fetal Fraction: A risk factor for adverse pregnancy outcome

Timothy J. Immonen^1^, Joseph Levy^2^, Ann‐Cathrin Lutterloh^3^, Lauren Robb^3^, Bernard Gonik^4^

^1^Detroit Medical Center, Detroit Medical Center, MI; ^2^University of Missouri‐Kansas City, Kansas City, Missouri; ^3^Detroit Medical Center, Detroit, Michigan; ^4^Wayne State University, Detroit, Michigan

17:15–18:30

**Objective**: The aim of this study was to determine if FF is affected in pregnancies complicated by preeclampsia, preterm birth (PTB), and small for gestational age (SGA).

**Study Design**: This was a retrospective cohort study who underwent NIPT for genetic screening at a single institution between 2017 and 2023. Three study groups were included: 1) patients with normal fetal fraction (*n* = 439); 2) patients with low FF on first draw (*n* = 110); 3) patients who had repeat draw performed following low FF result (*n* = 62). The primary outcome was a composite of preelcampsia, PTB < 37 weeks, SGA or birthweight < 10 percentile for gestational age, stillbirth >20 weeks after an NIPT result of low FF. NIPT was performed using Panorama from Natera Inc. and low FF was determined by a proprietary algorithm.

**Results**: In this cohort, 569 pregnancies were eligible for inclusion in the analysis. Cases afflicted with aneuploidy were excluded from the final analysis. Low FF occurred in 110 (20%) of pregnant patients. A repeat sample was obtained in 62 (52%) participants with recurrent low FF in 10 (16%) of those patients. FF increased with gestational age (r = 0.593) and decreased with rising body mass index (BMI) (r = 0.28) in pregnancies with normal fetal fraction. Low FF was associated with high BMI, early gestational age at blood draw, a history of chronic hypertension and having ever smoked cigarettes. Preeclampsia is associated with lower FF (*p* = 0.02). Pregnancies with low FF were more likely to develop the composite outcome when compared to normal FF (*p* < 0.001). The increase was primarily driven by hypertensive disorders of pregnancy (*p* = 0.001), more specifically, preeclampsia (*p* = 0.005). Low FF was more indicative of preterm preeclampsia (*p* = 0.041) than term preeclampsia (*p* = 0.064).

**Conclusion**: FF increased as gestational age advanced and decreased as a function of BMI. Pregnant patients with a low FF are at increased risk for experiencing an adverse outcome, particularly hypertensive disorders of pregnancy. Clinicians should be aware of these increased risks and consider close follow‐up if a low FF is identified during routine NIPT.

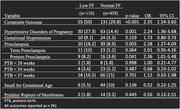

## 171 | Induction of labor with misoprostol in grand‐multiparous women with an unscared uterus – Is it safe?

Gal Issakov^1^, Ron Rosenberg^1^, Tzippy Shochat^2^, Amir Weintraub^1^, Salim Kees^1^, Hila Hochler^1^, Suzi Oberman Farhi^1^, Matan Elami‐Suzin^1^, Nir Duvdevani^1^, Danit Aviv^1^, Lee Ravid Arbel^1^, Khaled Massalha^1^, Yael Melamed Yekel^1^

^1^Obstetrics Gynecology and IVF Department, Laniado Medical Center, Netanya, HaMerkaz; ^2^Rabin Medical Center, Petach Tikva, HaMerkaz

17:15–18:30

**Objective**: Grand multiparity is considered a risk factor for uterine rupture even in women with an unscarred uterus, therefore induction of labor in this population is performed with extra caution. The aim of the study was to compare obstetric and neonatal outcomes in grand multiparous women who underwent induction with misoprostol to those induced with oxytocin, as well as to women who experienced spontaneous labor.

**Study Design**: We conducted a retrospective cohort study on 8,352 grand multiparous women (labor 6‐8) with an unscarred uterus who gave birth in our institute from 2005 to 2022. We compared the safety and efficacy of induction of labor with misoprostol (*n* = 193) (study group) to oxytocin (*n* = 579) and spontaneous onset of labor (*n* = 7,580) (control groups). The primary outcome was mode of delivery, and secondary outcomes included length of induction, uterine rupture, postpartum hemorrhage, placental abruption, pH < 7.1, five‐minute Apgar score < 7, and NICU admissions.

**Results**: Induction of labor with misoprostol compared to oxytocin in grand multiparous women did not increase the rate of cesarean delivery (2.59% vs. 3.45% *p* = 0.64) or instrumental delivery (1.55% vs. 2.42% *p* = 0.58). There were no cases of uterine rupture or advanced perineal tears in any of the groups. There were no differences in the maternal and neonatal complications. The use of epidural analgesia was significantly lower in the misoprostol group (69.95% vs. 83.94% *p* < .001). Mean time from induction to delivery was longer in the misoprostol group by less than one day compared to oxytocin induction (45 hours vs. 33 hours *p* < .0001).

**Conclusion**: Misoprostol is a safe and effective induction method in grand multiparous women with an unscarred uterus.

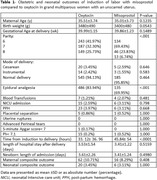

## 172 | The relationship between cardiac disease and postpartum emergency department visits and hospital readmission

Noor Joudi^1^, Jonathan A. Mayo^1^, Stephanie A. Leonard^1^, Kelly F. Darmawan^2^, Suzan L. Carmichael^1^, Danielle M. Panelli^1^, Abha Khandelwal^3^, Katherine Bianco^1^

^1^Stanford University, Palo Alto, California; ^2^Stanford University, Stanford University, California; ^3^Stanford University, Stanford, California

17:15–18:30

**Objective**: Cardiovascular disease (CD) is associated with increased hospital re‐presentation at 2 weeks and 6 weeks postpartum, primarily for indications associated with SMM, non‐transfusion SMM, and cardiac SMM. Targeted postpartum intervention strategies that focus on postpartum cardiovascular risks for re‐presentation may be warranted.

**Study Design**: This was a population‐based cohort study using linked birth certificate and hospital discharge data for births in California from 2007‐2018. Patients with cardiac disease were identified using diagnosis codes for congenital cardiac disease, acquired cardiomyopathy, ischemic heart disease, valvular disease, arrhythmias, cardiomyopathy and heart failure. We excluded hypertensive disorders. Multivariable logistic regression models were conducted to determine the association of CD with postpartum emergency department (ED) visits and hospital readmissions.

**Results**: In this cohort of 5,199,040 deliveries, people with CD had increased odds of ED visits and hospital readmission within 2 weeks [aOR 1.70 (CI 1.60‐1.80)] and within 6 weeks [aOR 1.73 (CI 1.65‐1.82)] post‐discharge compared to people without CD (Table 1). Among people with hospital re‐presentation 6 weeks post‐discharge, severe maternal morbidity (SMM), non‐transfusion SMM, and cardiac SMM as admission diagnoses were significantly higher in people with CD (1.75% vs 0.26%, *p*‐value < 0.0001; 1.62% vs 0.19%, *p*‐value < 0.0001; and 0.98% vs 0.03%, respectively, *p*‐value < 0.0001).

**Conclusion**: Cardiovascular disease with is associated with increased hospital re‐presentation at 2 weeks and 6 weeks postpartum, primarily for indications associated with SMM, non‐transfusion SMM, and cardiac SMM. Targeted postpartum intervention strategies that focus on postpartum cardiovascular risks for re‐presentation may be warranted.

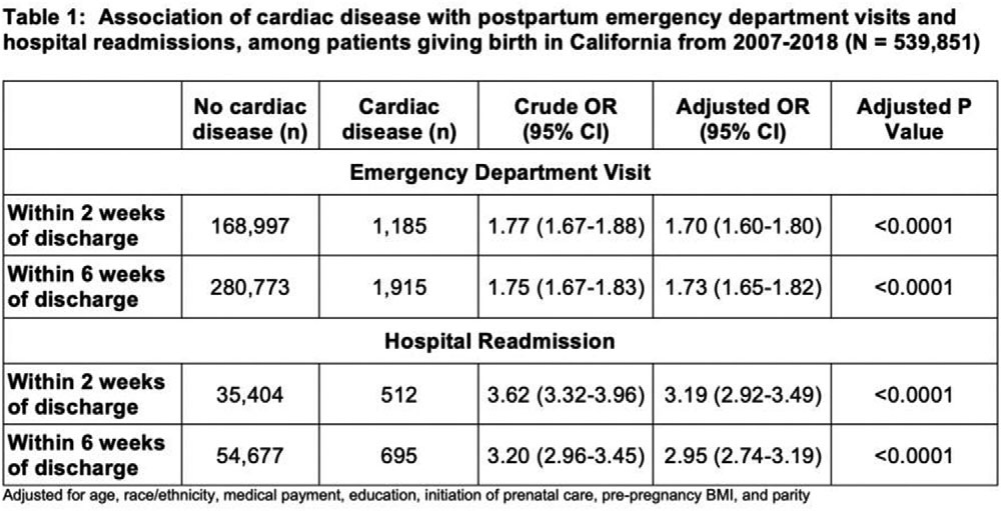

## 173 | Neurologic pediatric morbidity of small for gestational age offspring of short stature mothers

Noa Leybovitz Haleluya^1^, Tamar Wainstock^2^, Eyal Sheiner^2^

^1^Soroka Medical Center, Meitar, HaDarom; ^2^Department of Obstetrics and Gynecology, Soroka University Medical Center, Ben‐Gurion University of the Negev, Beer‐Sheva, Israel., Beer Sheva, HaDarom

17:15–18:30

**Objective**: Small for gestational age (SGA) definition does not distinguish SGA infants that achieves their normal growth potential (i.e constitutional) from small infants with growth restriction. SGA newborns in general are considered to be at an an increased risk for neurologic morbidity. We studied the association between SGA infants born to short‐stature mothers (assuming constitutional), and long‐term neurologic pediatric morbidity, compared to SGA infants born to non‐short mothers. Our hypotheses was that constitutionally small infants are not at an increased risk for long‐term neurologic morbidity.

**Study Design**: A population‐based retrospective cohort study including all SGA infants born between the years 1991‐2021 at a tertiary medical center was conducted. Long‐term pediatric neurologic morbidity among SGA offspring of short‐statured mothers was compared with SGA offspring of non‐short mothers. A Kaplan‐Meier survival curve was used to compare the cumulative neurologic morbidity incidence, and a Cox proportional hazards model was constructed to adjust for confounders. Infants with congenital malformations and multiple gestations were excluded from the analysis.

**Results**: The study population included 16,210 SGA newborns; 1088 (6.7%) were born to short‐stature mothers. SGA offspring of short‐stature mothers had lower rates of long‐ term neurologic related hospitalizations as compared to SGA offspring of non‐short mothers, although it did not reach statistical significance (9.1% vs. 9.5%, *p* = 0.697; Kaplan‐Meier survival curve *p* = 0.121; table, figure). However, while using a Cox proportional hazard model controlling for gestational age, maternal age, diabetes and hypertension, an independent significant negative association was shown between SGA of short‐stature mothers and long‐term neurologic morbidity (Adjusted HR = 0.91, 95% CI 0.85‐0.96, *p* = 0.002).

**Conclusion**: SGA offspring of short stature mothers (assuming constitutional) has significantly **lower** risk of long‐term pediatric neurologic morbidity in comparison to SGA offspring of non‐short mothers.

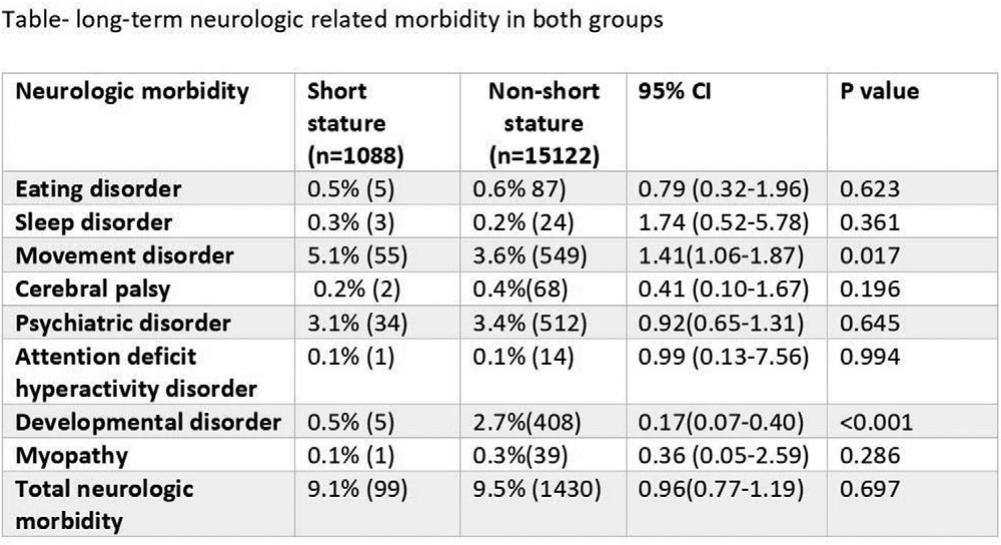

## 174 | Selective small for gestational age in twin pregnancies and the risk of long‐term neurologic morbidity

Noa Leybovitz Haleluya^1^, Tamar Wainstock^2^, Gali Pariente^2^, Eyal Sheiner^2^

^1^Soroka Medical Center, Meitar, HaDarom; ^2^Department of Obstetrics and Gynecology, Soroka University Medical Center, Ben‐Gurion University of the Negev, Beer‐Sheva, Israel., Beer Sheva, HaDarom

17:15–18:30

**Objective**: Small for gestational age (SGA) singletons are at increased risk for neurodevelopmental abnormalities. Scarce data exist regarding the long‐term implications of SGA in twins. As twins share the same intra‐ uterine environment and same maternal characteristics, assessing the association between selective SGA of on e twin and pediatric neurological morbidity, will allow us to further clarify the nature of this association. Therefore, we opted to study the association between SGA of one twin and long‐ term neurologic related morbidity

**Study Design**: A population‐based retrospective cohort study including consecutive dichorionic diamniotic twins, born between the years 1991‐2021 at a tertiary medical center. Total and subtypes of neurologic related pediatric hospitalizations among SGA versus non‐SGA twins were compared. A Kaplan‐Meier survival curve was used to compare the cumulative neurologic morbidity incidence, and a Cox proportional hazards model was constructed to adjust for confounders.

**Results**: The study population included 4,222 newborns; 180 (4.3%) were SGA. Rate of long‐ term neurologic related hospitalizations was comparable between the two groups (3.7% vs. 7.8%, *p* = 0.572; Kaplan‐Meier survival curve Log‐ rank *p* = 0.972; Table, Figure). Using a Cox proportional hazards model, controlling for gender and birth order, no association was found between SGA and the risk for subsequent neurologic pediatric morbidity of the offspring (Adjusted HR = 1.0, 95% CI 0.6‐1.8,* p* = 0.973).

**Conclusion**: SGA is not associated with an increased risk for long‐term pediatric neurologic morbidity in dichorionic diamniotic twins

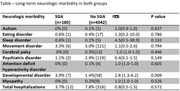

## 175 | Aspartate Aminotransferase to platelet ratio‐ predictor of small for gestational age newborns in pre‐eclamptic women

Noa Leybovitz Haleluya^1^, Lior Yahav^2^, Alla Saban^3^, Adi Y Y. Weintraub^4^, Reli Hershkovitz^5^, Tamar Eshkoli^4^

^1^Soroka Medical Center, Meitar, HaDarom; ^2^Soroka Medical Center, Beer Sheva, HaDarom; ^3^Soroka University Medical Center, Beer Sheva, HaDarom; ^4^Soroka University Medical Center, Soroka University Medical Center, HaDarom; ^5^Soroka medical center, Soroka medical center, HaDarom

17:15–18:30

**Objective**: Hypertensive disorders of pregnancy, especially preeclampsia toxemia (PET), can lead to significant maternal and neonatal adverse outcomes. Currently, there are no established and effective tests with a high predictive value for PET severity and its associated adverse outcomes. The aspartate aminotransferase‐to‐platelet ratio index (APRI) is linked to early inflammatory processes, but there is limited data available regarding its association with PET. Hence, our study aimed to investigate the potential correlation between the APRI parameter and the risk of small for gestational age (SGA) newborns in women diagnosed with PET.

**Study Design**: A population‐based cohort study at a tertiary medical center was conducted, including all women with singleton gestations who delivered between the years 2020 and 2022, and who were diagnosed with PET. Women with incomplete medical records, multiple gestations, and fetal malformations were excluded. The median APRI index of the cohort was used as a cut‐off point, allowing us to compare the risk of SGA between the low and high APRI index groups. A multivariable logistic regression was used to adjust for potential confounders, such as parity and gestational age

**Results**: The study population included 513 women with PET who met the inclusion criteria. The median APRI index value of the cohort was used as a cut‐off value, resulting in 255 women with an APRI index < 0.26 and 258 women with an APRI index ≥0.26. A higher APRI index was significantly associated with an increased risk of SGA newborn (Table 1, 24.4% vs. 14.9%; *p* = 0.008). In a multivariable logistic regression model, after adjusting for gestational age at delivery and parity, the association between SGA and APRI index of ≥0.26 remained significant (adjusted OR 1.60, 95% CI 1.01‐2.55; *p* = 0.047).

**Conclusion**: An APRI index ≥ 0.26 can be used as a predictor for SGA newborns in women diagnosed with PET.

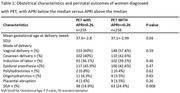

## 176 | Can Aspartate Aminotransferase to platelet ratio predict outcomes in Preeclamptic women with normal laboratory results?

Noa Leybovitz Haleluya^1^, Lior Yahav^2^, Alla Saban^3^, Adi Y Y. Weintraub^4^, Reli Hershkovitz^5^, Tamar Eshkoli^4^

^1^Soroka Medical Center, Meitar, HaDarom; ^2^Soroka Medical Center, Beer Sheva, HaDarom; ^3^Soroka University Medical Center, Beer Sheva, HaDarom; ^4^Soroka University Medical Center, Soroka University Medical Center, HaDarom; ^5^Soroka medical center, Soroka medical center, HaDarom

17:15–18:30

**Objective**: Hypertensive disorders during pregnancy has significant adverse effects. Preeclampsia‐toxemia (PET) is a multisystemic disease and its underlying mecha nisms involve inflammatory processes, endothelial dysfunction and intravascular platelet activation. The Aspartate aminotransferase‐to‐platelet ratio index (APRI) is associated with inflammatory processes. There is limited data on the association between APRI and PET. Our study aimed to investigate the correlation between the APRI parameter and obstetrical outcomes in women with PET and normal laboratory results.

**Study Design**: We conducted a cohort study at a tertiary medical center, including all women with singleton gestations who delivered between 2020 and 2022 and were diagnosed with PET. Exclusion criteria included women with an incomplete medical records, multiple gestations, and fetal malformations. We exclude women with elevated liver enzymes (Aspartate aminotransferase and Alanine transaminase) and low platelet count < 150,000. The cohort was divided into two groups based on the median APRI index, allowing for comparison of adverse outcomes between two groups. A multivariable logistic regression analysis was used to adjust for confounders, including maternal age, small for gestational age (SGA), early preterm delivery < 34 weeks and chronic hypertension

**Results**: A total of 357 women with PET, meeting the inclusion criteria were included in the study population. The cohort was divided based on the median APRI index value, resulting in 166 women with an APRI index < 0.21 and 191 women with an APRI index >0.21. An APRI index above 0.21 was significantly associated with an increased risk of early preterm delivery (Table 1, 13.1% vs. 5.4%; *p* = 0.010). Using a multivariable logistic regression analysis, after adjusting for maternal age, SGA and chronic hypertension, the association between early preterm delivery and an APRI index of ≥0.21 remained significant (adjusted OR 2.67, 95% CI 1.17‐6.06; *p* = 0.010).

**Conclusion**: In woman with PET and normal aminotransferases and platelets, an APRI index ≥ 0.21 is predictive of early preterm delivery before 34 week

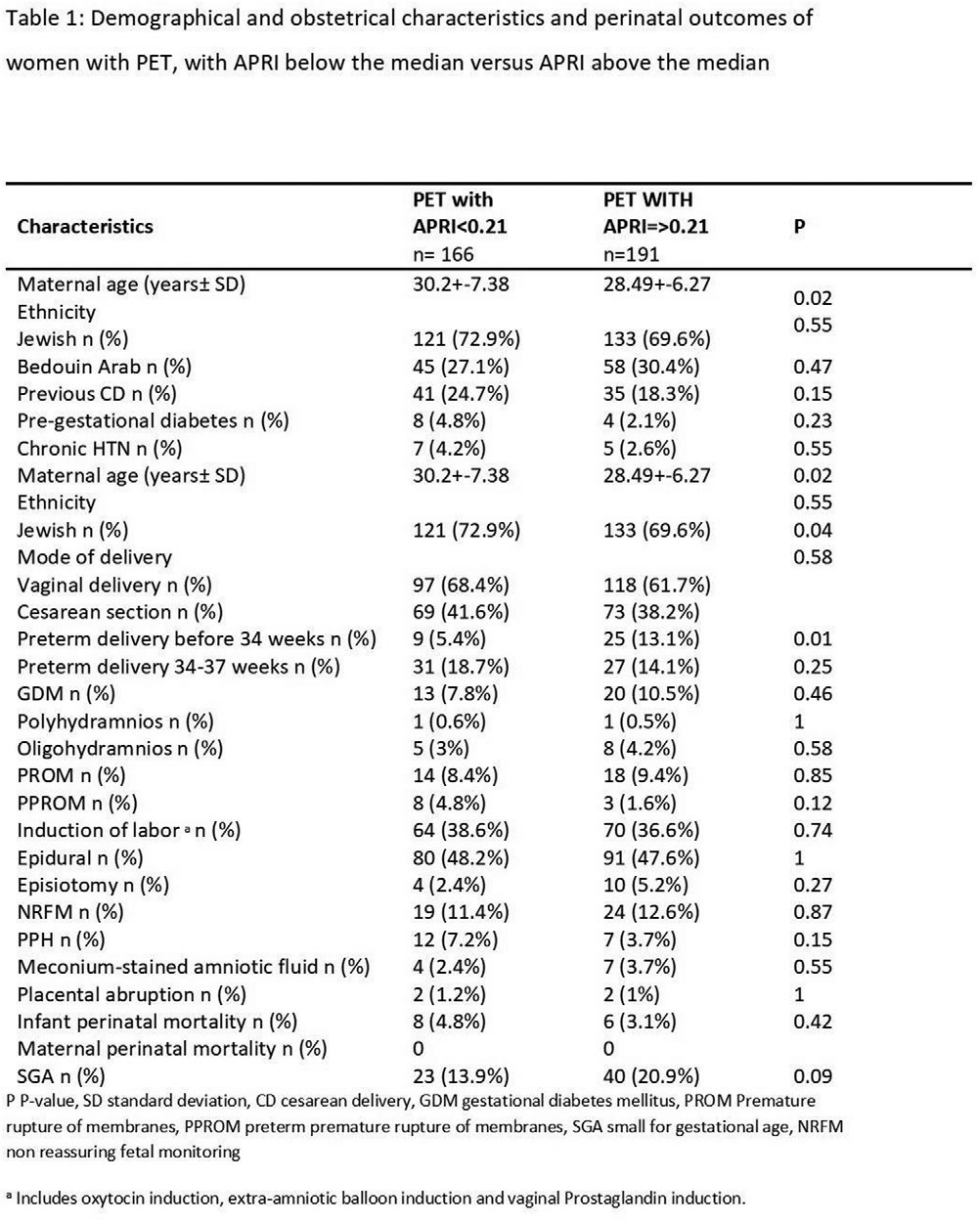

## 177 | Risk of term preeclampsia in first‐trimester screen‐positive women: A cohort study

CHRISTINA AMMARI^1^, Sara Hillman^2^, Davide Casagrandi^3^, Raffaele Napolitano^4^, Diane Nzelu^3^

^1^University College London, LONDON, England; ^2^Elizabeth Garrett Anderson Institute for Women's Health, University College London, London, England; ^3^University College London Hospital, University College London Hospital, England; ^4^University College London Hospital, University College London, England

17:15–18:30

**Objective**: This study aimed to evaluate the risk of preeclampsia (PE) among women who initially screen positive for preterm PE in the first trimester yet exhibit normal fetal growth and blood pressure at 36 weeks. It analyzed how the timing of delivery (before or after 40 weeks) and the screening method influence the risk.

**Study Design**: In this observational study, we analyzed 1802 singleton pregnancies screened for PE using the National Institute for Health and Care Excellence (NICE) method. We retrospectively applied the first trimester Fetal Medicine Foundation (FMF) PE screening method to the same cohort. The inclusion criteria required delivery post‐36+6 weeks without signs of preterm PE and with normal fetal growth assessment by ultrasound in the third trimester.

The cohort was categorized into two groups: those who delivered between 37 and 39+6 weeks (group 1), and those who delivered at 40 weeks or later (group 2). We compared the incidence of term PE between these groups, sub‐grouped based on the screening method, employing the chi‐square test for statistical analysis.

**Results**: Women in group 2 who had screened positive in the first trimester based on the FMF‐screening approach, showed a statistically significant increase in the risk of term preeclampsia by 14.6%, with a *p*‐value of 0.008, compared to group 1. The number needed to treat to prevent one case of PE in this cohort was calculated to be 7, if the delivery was to take place prior to 40 weeks. Conversely, the risk rise for term PE in group 2 screened by NICE was smaller and not statistically significant, amounting to only 1.2%.

**Conclusion**: Our study reveals that using the FMF‐first trimester screening method for PE women who test positive and have uncomplicated pregnancies until 37 weeks face a significantly higher risk of developing term PE if they deliver after 40 weeks. These findings support the recommendation of offering routine induction before 40 weeks to women who screen positive, as a preventive measure against the onset of term PE.

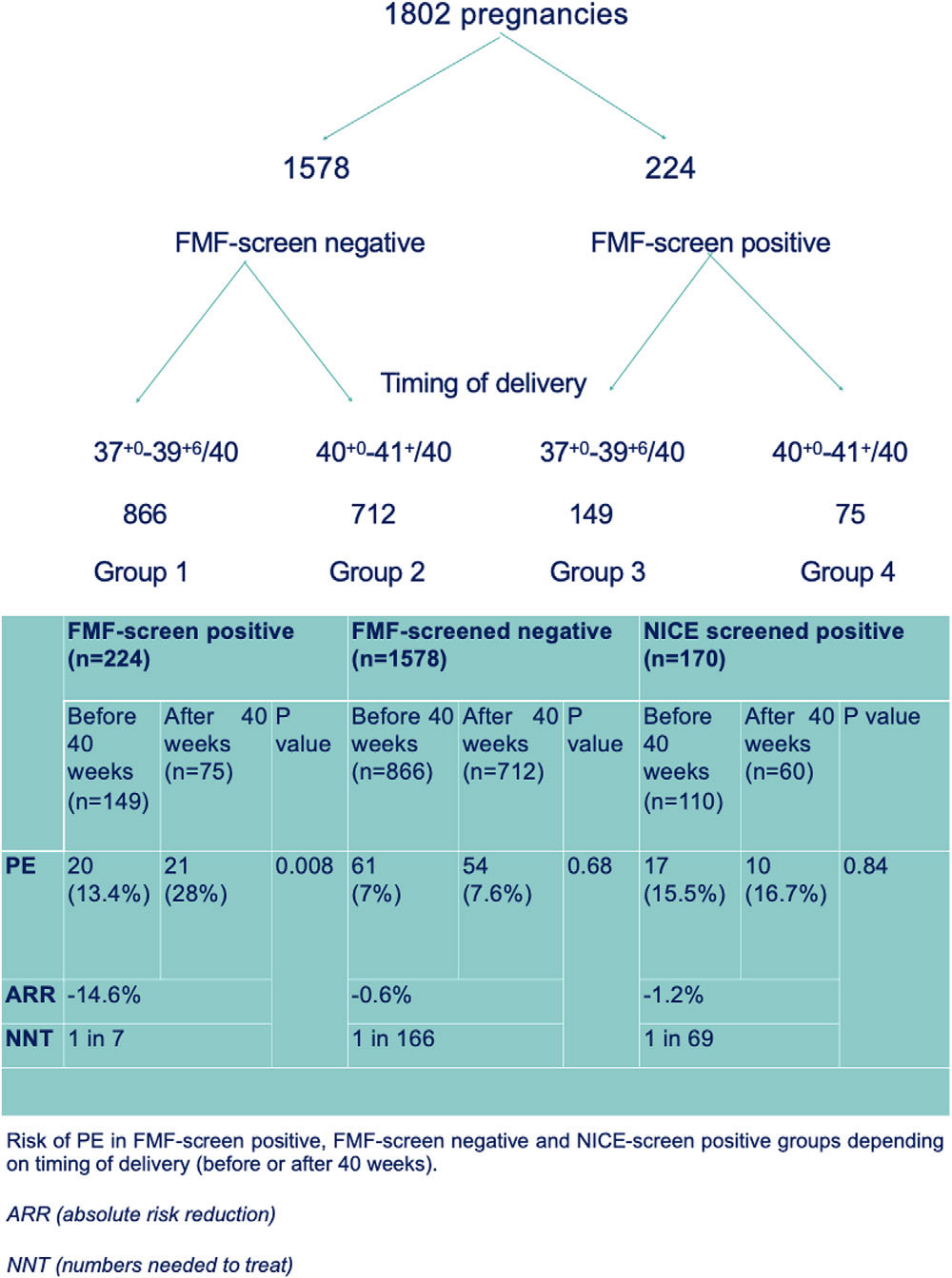

## 178 | A systematic review of national and international clinical practice guidelines on preeclampsia prediction and prevention

CHRISTINA AMMARI^1^, Diane Nzelu^2^, Sara Hillman^3^, Davide Casagrandi^2^, Raffaele Napolitano^4^

^1^University College London, LONDON, England; ^2^University College London Hospital, University College London Hospital, England; ^3^Elizabeth Garrett Anderson Institute for Women's Health, University College London, London, England; ^4^University College London Hospital, University College London, England

17:15–18:30

**Objective**: Our study systematically reviewed global clinical practice guidelines (CPGs) on combined screening for preeclampsia (PE) to evaluate current practices and changes post‐introduction.

**Study Design**: Our systematic review was registered on PROSPERO before the literature review. We included CPGs that focused on PE prediction and prevention in the first trimester. We assessed screening methods, risk factors, aspirin dosage, safety discussions, pharmacological measures, and non‐pharmacological interventions.

**Results**: We reviewed 31 CPGs published since 2017. Although there is an increase in adopting combined screening, 81% of guidelines continue to recommend risk factor‐based screening. There is variability in defining maternal risk factors; however, all guidelines recognize previous PE and medical comorbidities as risks. Recommendations on low‐dose aspirin (LDA) dosage varied, with 48% suggesting a range from 75/80mg to 150/162mg, and specific dose selection criteria were often lacking. Discussions on LDA risks during pregnancy were infrequent, which could affect adherence. Protocols for initiating and stopping aspirin therapy also varied, potentially complicating adherence. While all guidelines acknowledged the benefits of aspirin, only a quarter addressed its side effects. Half of the guidelines recommended calcium supplementation, though dosage and timing were inconsistent. There is an increasing endorsement of non‐pharmacological interventions, such as exercise and dietary modifications, for reducing PE risk.

**Conclusion**: Despite a trend towards combined screening, most CPGs still support risk factor‐based screening for PE. All CPGs recommend LDA, with calcium supplementation being the second most cited preventative strategy, particularly for women at high risk of PE with low dietary calcium. Non‐pharmacological interventions are also gaining recognition. Further research is needed to explore the feasibility of different screening approaches in various economic settings and healthcare infrastructures. This will aid in establishing more uniform international guidelines for predicting and preventing PE.

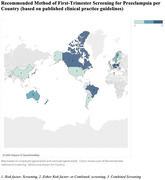

## 179 | Reproducibility of uterine artery pulsatility index following implementation of combined screening for pre‐eclampsia at UCLH

CHRISTINA AMMARI^1^, Diane Nzelu^2^, Georgios Christodoulou^3^, Shukria Omer^3^, Yesim Yildiz^3^, Davide Casagrandi^2^, Raffaele Napolitano^4^, Sara Hillman^5^

^1^University College London, LONDON, England; ^2^University College London Hospital, University College London Hospital, England; ^3^University College London, University College London Hospital, England; ^4^University College London Hospital, University College London, England; ^5^Elizabeth Garrett Anderson Institute for Women's Health, University College London, London, England

17:15–18:30

**Objective**: Our aim was to assess the reproducibility of uterine artery (UtA) blood flow measurements by sonographers following the implementation of combined screening for preeclampsia (PE) at University College London Hospital (UCLH) in February 2023. We sought to evaluate both intra‐ and inter‐observer reproducibility to ensure consistency in measurements. If discrepancies were identified, further training would be arranged for the sonographers.

**Study Design**: We collected prospective data on uterine artery (UtA) blood flows from 143 patients with singleton pregnancies over a period of 6 months. We compared both the intra‐ and the inter‐ observer reproducibility by assessing intraclass correlation coefficient (ICC) with 2‐way random effects model and absolute agreement. Results were analysed with Bland‐Altman plots revealing limits of agreement (LoA).

**Results**: A total of 569 Ut A PI comparisons were made for the intra‐observer and the inter‐observer reproducibility. The mean difference between repeated measurements by the same sonographer was 0.0116 (95% CI: −0.002 to 0.026), with 95% LoA of −0.001 to 0.034 and an ICC of 0.974 (95% CI: 0.97‐0.978, *p*‐value < 0.001) for intra‐observer comparisons. For inter‐observer comparisons, the mean difference in UtA PI values was −0.026 (95% CI: −0.048 to −0.0043), with 95% LoA of −0.077 to 0.025 and an ICC of 0.94 (95% CI: 0.927‐0.948, *p*‐value < 0.001).

**Conclusion**: Our study demonstrates that UtA PI measurements by trained sonographers following the implementation of first‐trimester screening for PE at UCLH are reproducible. Individualized training, including one‐to‐one teaching, feedback provision, and completion of online learning prior to accreditation by the Fetal Medicine Foundation (FMF), contributed to excellent reproducibility. This underscores the importance of tailored training for sonographers before the introduction of new services to ensure the reliability of combined screening for PE.

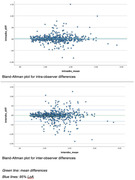

## 181 | Metformin and the risk of pre‐eclampsia: A two‐country analysis

Hannah Gordon^1^, Noor Ahmed Salim^2^, Sue Walker^3^, Ann Doust^4^, Stephen Tong^5^, Catherine Anne. Cluver^6^, Lauren Sutherland^4^, Anthea Lindquist^7^, Lina Bergman^8^, Susanne Hesselman^2^, Roxanne Hastie^7^

^1^University of Melbourne, Victoria; ^2^Uppsala University, Department of Women's and Children's Health, Uppsala University, Uppsala Lan; ^3^Department of Obstetrics, Gynaecology and Newborn Health, University of Melbourne, Melbourne, Victoria; ^4^University of Edinburgh, MRC Centre for Reproductive Health, University of Edinburgh Institute for Regeneration and Repair, Scotland; ^5^Department of Obstetrics, Gynaecology and Newborn Health, University of Melbourne, Heidelberg, Victoria, Victoria; ^6^Stellenbosch University, Stellenbosch University, Western Cape; ^7^University of Melbourne, University of Melbourne, Victoria; ^8^Department of Obstetrics and Gynecology, Sahlgrenska Academy, University of Gothenburg, Gothenburg, Vastra Gotaland

17:15–18:30

**Objective**: Metformin is an oral hypoglycaemic drug that has been proposed to treat or prevent preeclampsia. Combining data from two countries, Sweden and Scotland, we investigated whether metformin used during pregnancy was associated a reduced risk of hypertensive disorders of pregnancy, or preeclampsia.

**Study Design**: We combined data from population‐based cohorts obtained from Sweden (births between 2007‐2019) and Scotland (2012‐2018). We included nulliparous women with gestational diabetes or type 2 diabetes and linked birth outcomes with medication prescribed. Inverse probability weighted regression analyses were used to characterise the association between metformin prescription and any hypertensive disorder of pregnancy or only preeclampsia. We also explored neonatal outcomes. Results from both countries were combined in a meta‐analyse using a random effects model.

**Results**: We examined 7,771 women with diabetes in Sweden, where 19.3% (*n* = 1,498) were prescribed metformin during pregnancy, and 3,859 from Scotland where 30.8% (1,187) were prescribed metformin. Compared with non‐users, metformin was not associated with an altered risk of any hypertensive disorder of pregnancy (Sweden adjusted relative risk (aRR) 1.08 [95% CI 0.86, 1.37]; Scotland aRR 0.88 [95% CI 0.66‐1.19]) or preeclampsia (Sweden aRR 1.00 [95% CI 0.72, 1.39]; Scotland aRR 1.02 [95% CI 0.66‐1.60]). Combining adjusted results from both countries in a meta‐analysis revealed similar findings; a pooled RR of 0.98 (95% CI 0.79‐1.18) for any hypertensive disorder and RR 1.01 (95% CI 0.73‐1.28) for preeclampsia. In pooled adjusted analyses across both countries, metformin was not associated with adverse neonatal outcomes, including preterm birth < 37 weeks (RR 1.00 [95% CI 0.89‐1.13]), birthweight >4500g (RR 0.89 [95% CI 0.64, 1.24]), birthweight < 10th centile (RR 0.82 [95% CI 0.60‐1.13]) and birthweight < 3rd centile (RR 0.78 [95% CI 0.41‐1.48]).

**Conclusion**: Metformin use in pregnancy was not associated with an altered risk of developing any hypertensive disorder of pregnancy in either Sweden or Scotland.

## 182 | Long‐term outcomes after prenatal aspirin exposure: A 4‐year follow‐up of a randomized controlled trial

Emilie V.J. van Limburg Stirum^1^, Janneke van't Hooft^1^, Anna JEMC. Landman^1^, Martijn Finken^1^, Anita C. Ravelli^2^, Aleid G. Leemhuis^1^, Eva Pajkrt^2^, Martijn A. Oudijk^3^, Marjon A. de Boer^2^

^1^Amsterdam UMC, Amsterdam UMC, Noord‐Holland; ^2^Amsterdam UMC, Amsterdam, Noord‐Holland; ^3^Amsterdam UMC, location University of Amsterdam, Amsterdam, Noord‐Holland

17:15–18:30

**Objective**: Aspirin is increasingly prescribed to prevent pregnancy complications. With this study we assessed the long‐term effects of in utero exposure to aspirin on child development.

**Study design**: This study was a follow‐up of a randomized controlled trial (the APRIL study) which compared aspirin 80mg daily to placebo in women with a previous spontaneous preterm birth in singletons. In this follow‐up study children were assessed at four years corrected age using the Ages and Stages Questionnaire 3rd edition (ASQ‐3) and the Strength and Difficulties Questionnaire (SDQ) for (neuro)development and behavioral outcomes. Data on mortality, growth, general health, and a composite of abnormal child outcome or mortality were also collected. Analyses were performed by intention‐to‐treat reporting Odds Ratio (OR) and 95% Confidence Intervals (CI).

**Results**: Eight children were deceased during the original trial (*n* = 6 in aspirin and *n* = 2 in placebo group). Developmental and behavioral outcomes were collected for 231/379 (60.9%) children; 111/231 (48.1%) in the aspirin group and 120/231 (51.9%) in the placebo group. Total ASQ‐3 score was higher in the aspirin group (mean 259.3 versus 248.3, *p*‐value 0.03). The rate of children with an ASQ‐3 score of 1SD or more below the normative data did not differ between aspirin and placebo group (35.2% versus 47.4%; OR 0.60, 95% CI 0.33‐1.09), nor did a score on the SDQ ≥80 centile (18.3% versus 14.7%; OR 1.30, 95% CI 0.64‐2.66). Numbers of children with abnormal growth, general health and the composite outcome of abnormal child development or mortality did not significantly differ between groups.

**Conclusion**: We found no harmful effects of daily prenatal exposure to 80mg aspirin and potential beneficial effects on (neuro)developmental outcomes at four years corrected age. Our findings are in line with existing literature on safety of aspirin use in pregnancy.

## 183 | Pelvic and Sexual Health Outcomes after cesarean hysterectomy for placenta accreta spectrum

Austin Oberlin^1^, Mirella Mourad^2^, Meghan Angley^2^, Fady Khoury Collado^3^, Helai Hesham^3^

^1^Yale University, New York, NY; ^2^Columbia University Medical Center, New York, New York; ^3^Columbia University, Columbia University, New York

17:15–18:30

**Objective**: Literature on the long‐term outcomes after surgery for placenta accreta spectrum (PAS) is limited. The aim of this study was to explore the incidence of urge/stress urinary incontinence (UUI/SUI), pelvic organ prolapse (POP), and sexual dysfunction in a cohort of women who have undergone hysterectomy for PAS.

**Study Design**: Patients who underwent a cesarean hysterectomy for PAS between January 2018 and August 2023 at a single urban academic institution were recruited to complete a survey. Patients were included if they had surgery at least 6 months prior to the survey by the dedicated PAS team. The survey consisted of validated questionnaires (Pelvic Floor Distress Inventory, and Pelvic Organ Prolapse/Urinary Incontinence Sexual Questionnaire) and clinical data were abstracted from the medical record.

**Results**: During the study period, 100 patients underwent a hysterectomy for PAS, of which 64 agreed to participate in the survey. Participants had a median age of 36 at time of surgery, and a median of 2 prior cesarean deliveries. The majority of cases were scheduled (68.8%) and underwent a supracervical hysterectomy (67.5%). Cystotomy occurred in 12.5% of cases. Approximately 32% of participants had at least one symptom of POP, 39.3% had symptoms of SUI, 23% had symptoms of UUI and 12.5% had incomplete bladder emptying (Table 1). Symptoms of prolapse, SUI, UUI, incomplete bladder emptying, pelvic pain and dyspareunia were all higher amongst those who had a cystotomy, with the difference in SUI and pelvic pain being statistically significant. There was no difference in outcomes when comparing type of hysterectomy or the urgency of the case.

**Conclusion**: In this cohort of patients who underwent hysterectomy for PAS, there was a high rate of symptoms of POP, UI, pelvic pain and dyspareunia, especially in those with intra‐operative bladder injury. These results should be used to counsel patients prior to surgery. PAS centers of excellence should consider early referral to urogynecology post‐operatively.

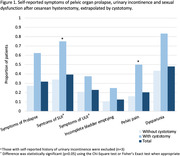

## 184 | Rates of depression, anxiety and PTSD after cesarean hysterectomy for placenta accreta spectrum

Austin Oberlin^1^, Fady Khoury Collado^2^, Catherine E. Monk^3^, Kristina D'Antonio^4^, Helai Hesham^2^, Meghan Angley^5^, Mirella Mourad^5^

^1^Yale University, New York, NY; ^2^Columbia University, Columbia University, New York; ^3^Columbia University Irving Medical Center, New York, New York; ^4^Columbia University Women's Mental Health Division, New York, New York; ^5^Columbia University Medical Center, New York, New York

17:15–18:30

**Objective**: Medically complicated pregnancies and deliveries can lead to adverse mental health outcomes, including post‐traumatic stress disorder (PTSD), postpartum. However, few studies have explored mental health outcomes after surgery for placenta accreta spectrum (PAS). Our aim was to understand factors that contribute to anxiety, depression, and PTSD in patients undergoing surgery for PAS.

**Study Design**: Patients who underwent a cesarean hysterectomy for PAS between January 2018 and August 2023 at a single urban academic institution were recruited to complete a survey. All cases were performed by a dedicated PAS team. Patients were included if they had surgery at least 6 months prior to the survey. The survey consisted of validated questionnaires (Patient Health Questionnaire [PHQ‐9], General Anxiety Disorder [GAD‐7], and the National Stressful Events Survey short scale [NSESSS] for symptoms of PTSD) and clinical data were extrapolated from the medical record.

**Results**: During the study period, 100 patients underwent a hysterectomy for PAS, of which 61 completed the survey. Fourteen percent of patients reported a past diagnosis of depression, 18% reported anxiety and 10% reported PTSD. After surgery, 13% of patients scored a ten or greater on the PHQ‐9, indicating symptoms of major depression, while 16% had a score on the GAD‐7 indicating symptoms of anxiety. Although for both depression and anxiety, most patients scored in the moderate range, a high proportion indicated difficulty coping related to these symptoms. Twenty‐one percent of patients experienced symptoms of PTSD. The rate of PTSD was higher in those who underwent an emergent surgery, although this was not statistically significant.

**Conclusion**: In patients who undergo cesarean hysterectomy for PAS, nearly a quarter may experience symptoms of PTSD, higher than the general postpartum population. Since surgical factors such as urgency of the case or complications were not associated with symptoms of PTSD, mental health services should be incorporated into routine postpartum care for these patients.

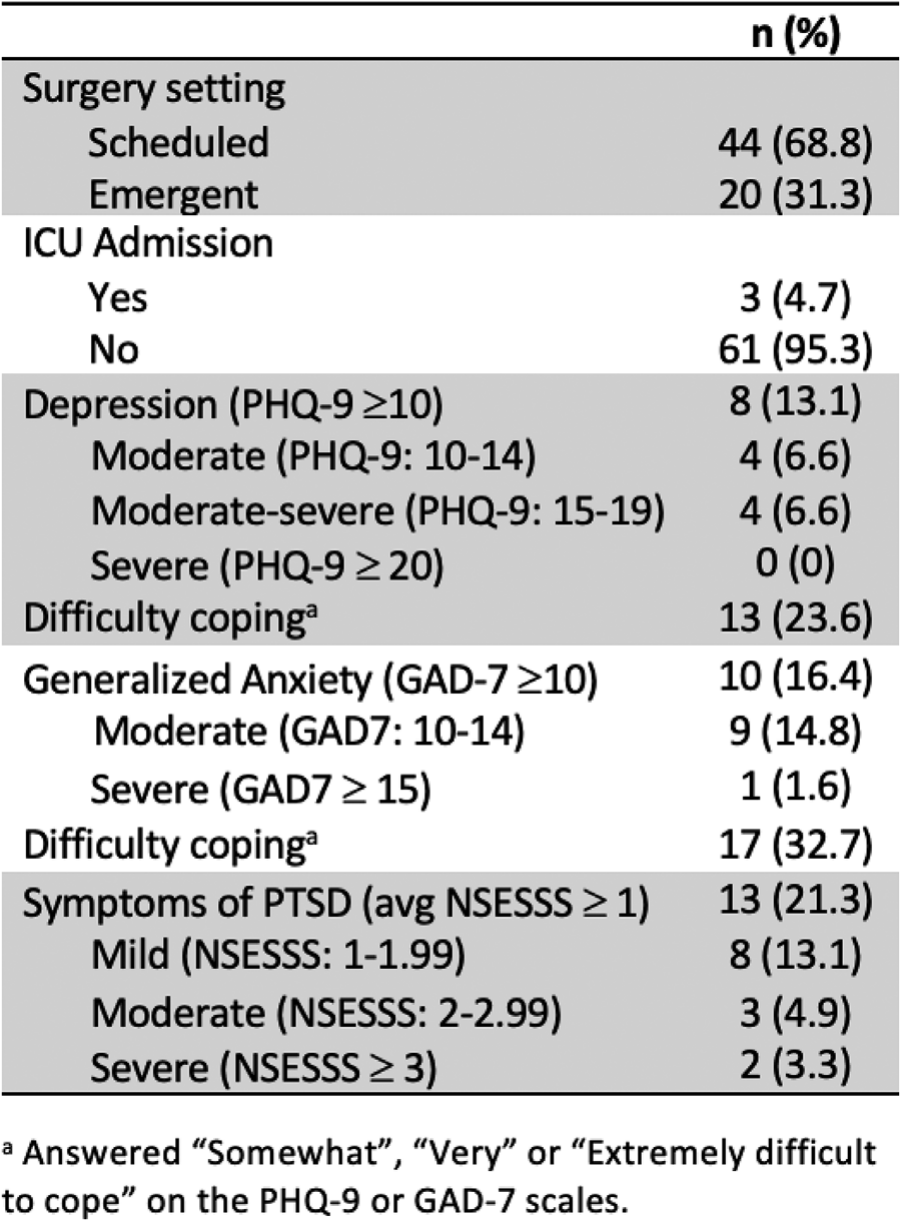

## 186 | Birthweight to placenta weight ratio in clinically unexpected stillbirths

Laura A. Lens^1^, Mauritia C. Marijnen^2^, Maddy Smies^2^, Wessel Ganzevoort^3^, Sanne J. Gordijn^4^

^1^Amsterdam UMC, Amsterdam, Noord‐Holland; ^2^Amsterdam UMC, AMC, Noord‐Holland; ^3^Amsterdam University Medical Centers, Amsterdam, Groningen; ^4^University Medical Center Groningen, Groningen, Groningen

17:15–18:30

**Objective**: In an extreme form placental insufficiency can result in stillbirth, but more often it results in fetal growth restriction. Unexpected stillbirths are preceded by apparently uncomplicated pregnancies with normal growth and routine examinations. We hypothesized that at least a part of these unexpected stillbirths had an undiagnosed placental insufficiency and abnormal birthweight/placenta weight ratio (BWPW‐ratio) as well as low placenta weight at pathology examination.

**Study Design**: We conducted a retrospective cohort study of stillbirths that occurred in the AMC hospital between January 2000 and December 2017. The stillbirth cohort was divided into three groups: known placental insufficiency, unexpected stillbirths, or due to another condition diagnosed prior to the stillbirth. The BWPW‐ratio was calculated, and the proportion of abnormal ratios was determined in every group. The proportion of low birthweight and low placental weight was also determined in every group. Subsequently, a total of 184 placenta's were histologically scored for primary, secondary or combined pathological lesions according to amended criteria of Freedman and Ernst.

**Results**: Between 2000 and 2017, we found 295 cases of stillbirths. Of the unexpected stillbirths, 71.3% had an abnormal BWPW‐ratio, which did not differ between the defined groups. Low placental weight was seen in 69.8% of the unexpected stillbirths, and in 91.7% of the placenta group, but significantly lower in the other causes‐group (42.1%). The most common primary placental lesion was maternal vascular malperfusion (MVM), which occurred in 53.7% of the cases. In 57.0 % there was only one lesion, whereas 35.7% showed a combination of lesions.

**Conclusion**: The greater part of unexpected stillbirths had a high BWPW‐ratio and low placenta weight. These proportions were the same or (in case of low placenta weight) higher in the ‘placental’ stillbirths but significantly lower in the ‘other’ stillbirths. This suggests that an undetected placental insufficiency contributes to a large proportion of the unexpected stillbirths.

## 187 | High‐versus low‐dose oxytocin regimens for labor augmentation: A systematic review and meta‐analysis

Teresa C. Logue^1^, Fabrizio Zullo^2^, Fiamma van Biema^3^, Moeun Son^4^, Anthony C. Sciscione^1^, Daniele Di Mascio^5^, Suneet P. Chauhan^6^

^1^Christiana Care Health System, Newark, Delaware; ^2^University of Rome La Sapienza, Rome, Lazio; ^3^Hackensack Meridian School of Medicine, Hackensack Meridian School of Medicine, New Jersey; ^4^Weill Cornell Medicine, New York, New York; ^5^Università Roma La Sapienza, Roma, Lazio; ^6^Delaware Center of Maternal‐Fetal Medicine at Christiana Care, Delaware, Delaware

17:15–18:30

**Objective**: ACOG supports either high‐ or low‐dose oxytocin regimens during labor. Trials of high‐ versus low‐dose strategies have not shown differences in neonatal outcomes but were limited by sample size. We sought to assess whether high‐ versus low‐dose regimens for labor augmentation are associated with differential risk for low Apgar score, neonatal acidosis, and other adverse labor outcomes.

**Study Design**: We conducted a systematic review and meta‐analysis of randomized and quasi‐randomized trials of nulliparous or multiparous parturients undergoing labor augmentation (CRD42024500197). Included trials compared high‐ (starting at 4+ mU/min, increasing by 3‐6 mU/min) to low‐dose (starting at 0.5‐2 mU/min, increasing by 1‐2 mU/min) protocols. Our co‐primary outcomes were Apgar score < 7 at 5 minutes and umbilical arterial pH < 7.00. Secondary outcomes included cesarean delivery (CD), chorioamnionitis (CO), and postpartum hemorrhage (PPH). We performed random‐effect head‐to‐head meta‐analyses to compare high‐ versus low‐dose strategies, reporting summary risk ratio (RR) with 95% confidence interval (CI). Subgroup analyses were conducted for the primary outcomes by parity and high quality‐studies by revised Cochrane risk‐of‐bias tool.

**Results**: Of 2,233 studies screened, 10 met inclusion criteria with 2864 individuals randomized to high‐ and 2648 to low‐dose oxytocin regimens. Baseline characteristics were balanced. There was no difference in risk for the primary outcomes of Apgar < 7 at 5 min [RR 0.94 (0.60, 1.46); *p* = 0.77] or pH < 7.00 [RR 0.77 (0.50, 1.20); *p* = 0.25]. Likelihood of CD [RR 0.83 (0.67, 1.02); *p* = 0.08] and PPH [RR 1.06 (0.85, 1.31); *p* = 0.62] were similar. High‐dose oxytocin was associated with significantly lower risk of CO [RR 0.68 (0.56, 0.83); *p* < 0.001]. These findings persisted in subgroup analyses.

**Conclusion**: High‐dose oxytocin did not significantly affect risk for low Apgar score, neonatal acidosis, CD or PPH though it decreased risk for CO. Clinicians and policy makers should be cognizant of the risk reduction of chorioamnionitis with high‐dose oxytocin, without apparent risk of fetal harm.

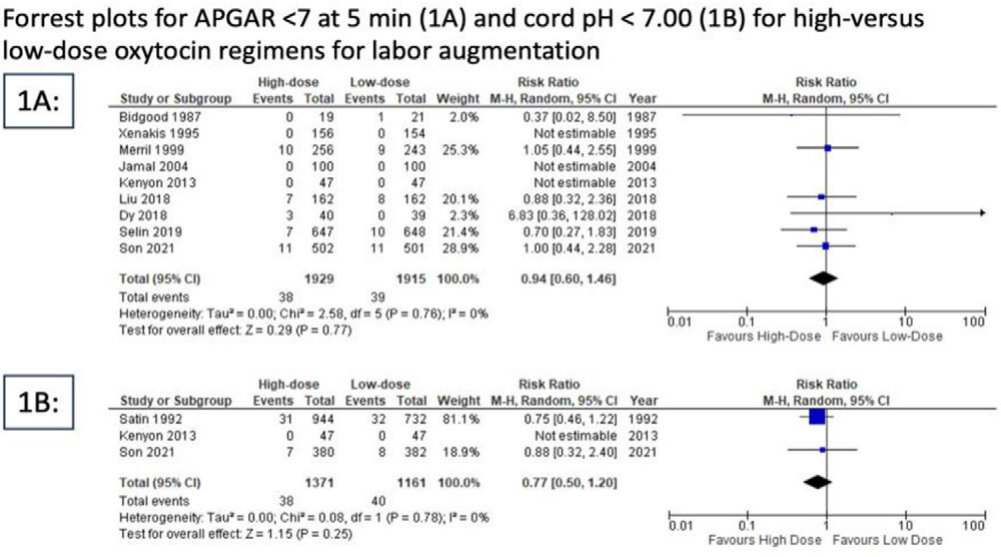

## 188 | Fertility treatment as a risk factor for McDonald's cerclage in twin pregnancies

Noam Shema^1^, Gali Pariente^2^, Gil Gutvirtz^3^, Tamar Wainstock^2^, Eyal Sheiner^2^

^1^soroka medical center, soroka medical center, HaDarom; ^2^Department of Obstetrics and Gynecology, Soroka University Medical Center, Ben‐Gurion University of the Negev, Beer‐Sheva, Israel., Beer Sheva, HaDarom; ^3^Soroka University Medical Center, Metar, HaDarom

17:15–18:30

**Objective**: The rising incidence of fertility treatments has led to an increase in the rate of twin pregnancies, which are associated with an increased risk for adverse perinatal outcomes, specifically preterm delivery. The use of cervical cerclage in non‐singleton pregnancies to prevent preterm delivery is controversial. This study was conducted to evaluate whether fertility treatment is a risk factor for cerclage placement in twin pregnancies.

**Study Design**: This is a population‐based cohort study of twin pregnancies that were delivered in a single tertiary referral medical center for 30 years. Rates of cerclage placement were compared between twin pregnancies conceived through fertility treatment and spontaneously conceived twin pregnancies. A generalized estimated equation (GEE) multivariable model was constructed to control for possible confounders

**Results**: The study included 11,986 twin pregnancies of which 3410 (28.4%) were conceived through fertility treatments. A cervical cerclage was performed in 113 (3.3%) pregnancies conceived through fertility treatments compared to 67 (0.8%;) of spontaneously conceived twin pregnancies (OR = 4.35 95% CI 3.20‐5.90, *p* < 0.001; Table). In the GEE model which adjusted for maternal age and gravidity, this association remained significant (adjusted OR = 6.22, 95% CI 3.36‐11.51, *p* < 0.001; Table).

**Conclusion**: In this study, we observed a notably higher occurrence of cerclage placement in twin pregnancies resulting from fertility treatments in contrast to spontaneously conceived twin pregnancies. Our findings suggest that fertility treatment independently elevates the risk of cerclage placement in twin pregnancies.

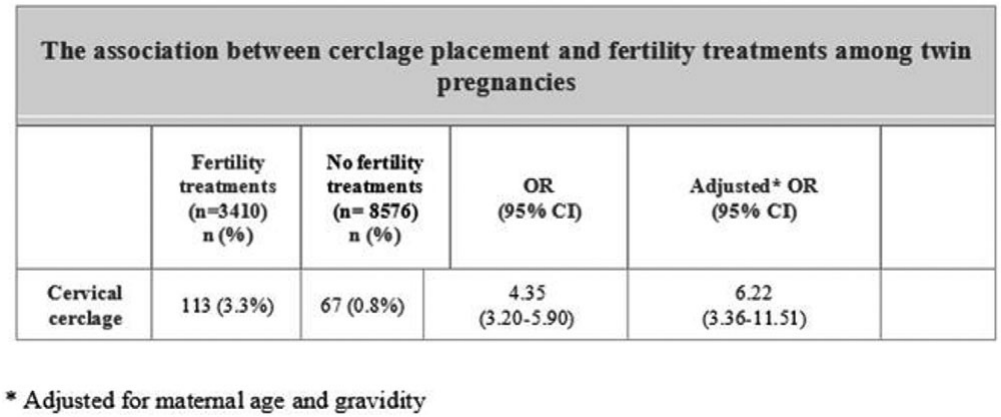

## 189 | Slowing fetal growth signifies perinatal mortality and morbidity risk: A retrospective study of 24,395 pregnancies

Teresa M. MacDonald^1^, Chloe Jamieson‐Grigg^1^, Pawel Kalinowski^2^, Stephen Tong^3^, Sarah Banting^1^, Sue Walker^1^

^1^Department of Obstetrics, Gynaecology and Newborn Health, University of Melbourne, Melbourne, Victoria; ^2^Melbourne Dementia Research Centre, University of Melbourne, Melbourne, Victoria; ^3^Department of Obstetrics, Gynaecology and Newborn Health, University of Melbourne, Heidelberg, Victoria, Victoria

17:15–18:30

**Objective**: Slowing fetal growth is an important sign of placental dysfunction, but uncertainty regarding what constitutes poor growth has hindered clinical translation. We aimed to investigate slowing fetal growth and adverse perinatal outcomes (APO) including perinatal mortality. We also aimed to ascertain a threshold of slowing growth associated with increased perinatal mortality risk.

**Study Design**: This single‐centre retrospective cohort study included all singleton pregnancies from 2009 with 2 assessments of fetal biometry, ≥ 2 weeks apart, from 18‐38 weeks. For each pregnancy, fetal growth rates (estimated fetal weight (EFW) centile/week; abdominal circumference (AC) centile/week) were calculated between the first and last scans included.

Logistic regression assessed the relationships between the fetal growth velocity parameters and perinatal mortality, and APO. Thresholds of slowing growth and perinatal mortality were examined by Fisher's exact test.

**Results**: 24395 pregnancies were included with a median 12^+3^ weeks between first and last scans. The mid‐trimester morphology scan was the first growth assessment in 62% of pregnancies. EFW centile change/week was strongly inversely associated with perinatal mortality and APO. Each centile/week reduction in EFW increased the odds [95% confidence interval] of perinatal mortality by 8.8% [1.7%‐15.6%], *p* = 0.01; and of APO by 8.4% [7.3%‐9.6%], *p* < 0.0001. AC centile/week growth rate was not significantly associated with perinatal mortality, but each centile/week reduction in AC increased the odds of APO by 6.8% [5.8%‐7.8%], *p* < 0.0001.

EFW centile/week cut‐offs were tested to identify a clinically useful threshold. Perinatal mortality was significantly increased once the EFW fell at a rate of > 2.5 centiles/week, with a stepwise increase as growth velocity progressively slowed.

**Conclusion**: This large investigation confirms that a slowing fetal growth rate signifies increased risk of important adverse perinatal outcomes, including mortality. Assessment of fetal growth rate could assist clinicians to better identify at‐risk fetuses, and to plan care to reduce this risk.

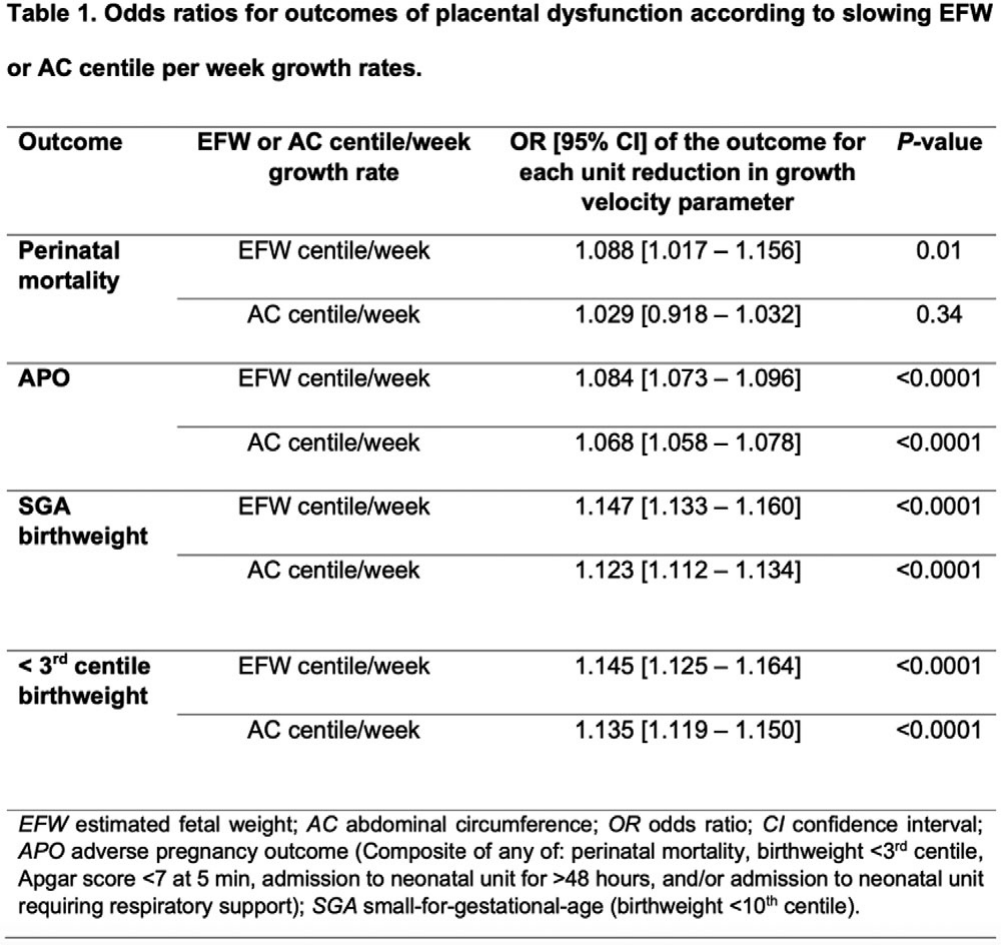

## 190 | Outcomes of fetal ovarian cysts better following in utero aspiration than expectant management

Laurent Mandelbrot^1^, Violaine Peyronnet^2^, Laure Tudal^2^, Charles Egloff^2^, Cergika Velupilai^3^, Catherine Noel^4^, Nathalie Tordjeman^5^, Valérie Mairovitz^6^, Chloé, Lieng^2^, mikael Tassin^2^, Olivier Picone^2^

^1^Hôpital Louis Mourier, APHP, Université Paris Cité, Colombes, Ile‐de‐France; ^2^Assistance Publique/Hôpitaux de Paris, Université Paris Cité, Hôpital Louis Mourier, Colombes, Ile‐de‐France; ^3^Hopital NOVO, PONTOISE, Ile‐de‐France; ^4^Hôpital Eaubonne Montmorency, eAUBONNE, Ile‐de‐France; ^5^CH d'Argenteuil, Argenteuil, Ile‐de‐France; ^6^Institut Franco‐Britannique, LEVALLOIS PERRET, Ile‐de‐France

17:15–18:30

**Objective**: There is longstanding uncertainty regarding in utero therapy for large prenatally diagnosed ovarian cysts. This study was performed to evaluate whether perinatal outcomes were better following in utero aspiration vs no prenatal intervention.

**Study Design**: A retrospective study of all fetal ovarian cysts diagnosed in 4 centers in Paris, France. The decision to perform in utero therapy for anechogenic cysts > 3 cm diameter was a shared decision on a case‐by‐case basis. The primary outcome studied was the occurrence of a complication in the prenatal or neonatal period. Complications were defined by the appearance of hyperechogenic changes in the cyst, suggesting torsion or hemorrhage. The secondary outcome was the incidence of postnatal surgical treatment.

**Results**: Of 65 ovarian cysts which were uncomplicated at presentation, 18 had transabdominal aspiration under ultrasound guidance and 47 were managed expectantly. Mean gestational age at the time of diagnosis was 33 WG (IQR 32‐33.4) with no difference between groups. The mean maximum diameter was greater in the aspiration than in the expectant group, 45 mm (IQR: 40‐55) vs 39 mm (32‐45), *p* < 0.01. Mean gestational age at intervention was 34.2 WG (33.5‐36.1). There was one case of premature rupture of membrane following intervention (1/18, 5.6%). Complications, defined as hyperechogenic changes, were observed by prenatal and/or postnatal ultrasound in 1 case (5.6%) in the intervention group, vs 21 (44.7%) in the observation group, *p* = 0.003. Postnatal surgery was required in 1 case (5.6%) vs 5 (11.4%), respectively (*p* = 0.66).

**Conclusion**: Our findings suggest that in utero aspiration can prevent complications in large prenatal ovarian cysts. Definitive evidence would require neonatal surgical exploration in all infants with ovarian cysts, which is not warranted.

## 191 | Digital therapeutics for birthing people with opioid and substance use disorder

Maria Manriquez^1^, Kristyn Piñeda^2^, Erika Flores^3^, Pooja Rangan^3^

^1^University of Arizona College of Medicine–Phoenix, University of Arizona College of Medicine Phoenix, Arizona; ^2^University of Arizona College of Medicine–Phoenix, Phoenix, Arizona; ^3^University of Arizona College of Medicine Phoenix, University of Arizona College of Medicine Phoenix, Arizona

17:15–18:30

**Objective**: The goal of this retrospective cohort study was to determine if pregnant or postpartum people showed higher engagement in addiction medicine treatment with digital therapeutic (DTx) intervention.

**Study Design**: A two‐site retrospective cohort study enrolled 23 pregnant or postpartum people aged 18 to 50 years with an opioid use disorder (OUD) or substance use disorder (SUD) diagnosis who were seeking addiction medicine treatment and consented to be in the program. The 23 control subjects received care at one of the two sites during the same time frame of enrollment. Participants were given access to a HIPAA‐compliant mobile application designed for pregnant and postpartum people with OUD/SUD. The platform allowed the personalization of goals, objectives, and module activities.

**Results**: Of the 46 individuals, most participants who used DTx were younger. Most patients had Medicaid insurance in the participants (*n* = 30, 87%) and the study group (*n* = 17, 74%). The median (Interquartile range) number of substances used was similar (*p* = 0.478) in both participants (3[2‐3]) vs controls (3[2‐4]). Participants had no missed appointments during the study period as compared to controls (median [IQR]: 3[1‐6], *p* =  < 0.0001). There was no difference in the relapse or preterm rates and NICU admissions between the groups. The incidence of NOWS/NAS was lower in participants (*n* = 4, 17%) compared to controls (*n* = 11, 48%). This difference showed a trend towards statistical significance (*p* = 0.057). On multivariate analysis, after controlling for age group, number of substances used, and Medicaid status, the NOWS/NAS incidence (aOR: 0.18, 95% CI: (0.04 to 0.81, *p* = 0.026) and the number of appointments missed (aβ: −2.8, 95% CI: −3.96 to −1.58, *p* =  < 0.001) was significantly lower in participants.

**Conclusion**: This study provides encouraging preliminary evidence for the benefits of DTx in pregnant/postpartum people with OUD/SUD. Further research is warranted to confirm these findings and optimize the intervention to improve maternal and neonatal health outcomes in this vulnerable population.

## 192 | Concordance between patient‐reported and Electronic Medical Record (EMR)‐abstracted data among high‐risk pregnant patients

Sarah Heerboth^1^, Emily Gascoigne^2^, Lauren Kucirka^3^, Hadley Hartwell^1^, Alison M. Stuebe^4^, Rebecca Fry^1^, Tracy A. Manuck^1^

^1^University of North Carolina, Chapel Hill, North Carolina; ^2^UNC Chapel Hill School of Medicine, Carrboro, North Carolina; ^3^University of North Carolina, Chapel Hill, Chapel Hill, North Carolina; ^4^UNC School of Medicine, Chapel Hill, North Carolina

17:15–18:30

**Objective**: Accurate medical and OB history is crucial to optimize care, but discrepancies between patient‐reported history and the EMR are common. We sought to evaluate patient‐reported and EMR abstracted concordance and determine factors associated with discordance.

**Study Design**: Prospective, longitudinal, observational cohort of patients at high‐risk for adverse pregnancy outcomes at a tertiary Univ. hospital, 2017‐2022. At enrollment, participants completed surveys assessing medical and OB hx; some also completed postpartum surveys. Medical and OB hx data were manually abstracted from the EMR. Patient:EMR concordance was evaluated for 9 characteristics–gravida, medical hx (anxiety ± depression, diabetes, HTN) and OB hx (gestational age of the earliest prior birth; hx of IUGR, abruption, HTN disorders of pregnancy, PTB). All discordant patient:EMR findings underwent a second EMR review by a MFM physician. Patient:EMR concordance was evaluated using Cohen's kappa. Factors associated with discordance (defined as patient:EMR disagreement on ≥3 of 9 characteristics) were evaluated using logistic regression.

**Results**: 381 individuals participated; 38 (10%) had 0, 168 (44%) had 1, 108 (28%) had 2, and 67 (18%) had ≥ 3 discordant characteristics (and were classified as discordant). Concordance was imperfect for all 9 characteristics. Cohen's kappa and percent agreement varied widely by characteristic, *Table*. Concordance was lower for medical compared to OB hx characteristics (average Cohen's kappa 0.419 vs. 0.559). In logistic regression models, Medicaid/self‐pay (aOR 2.60, 95% CI 1.53‐5.28), maternal age (aOR 1.13, 95% CI 1.07‐1.20), and gestational age of the initial survey (aOR 0.96, 95% CI 0.93‐0.99)–but not maternal race or education–were associated with patient‐EMR discordance.

**Conclusion**: We found significant patient:EMR discordance for multiple medical and OB hx factors among individuals at high‐risk for adverse pregnancy outcomes. These findings have implications for both clinical care and research, underscoring the importance of patient communication and accurate medical documentation.

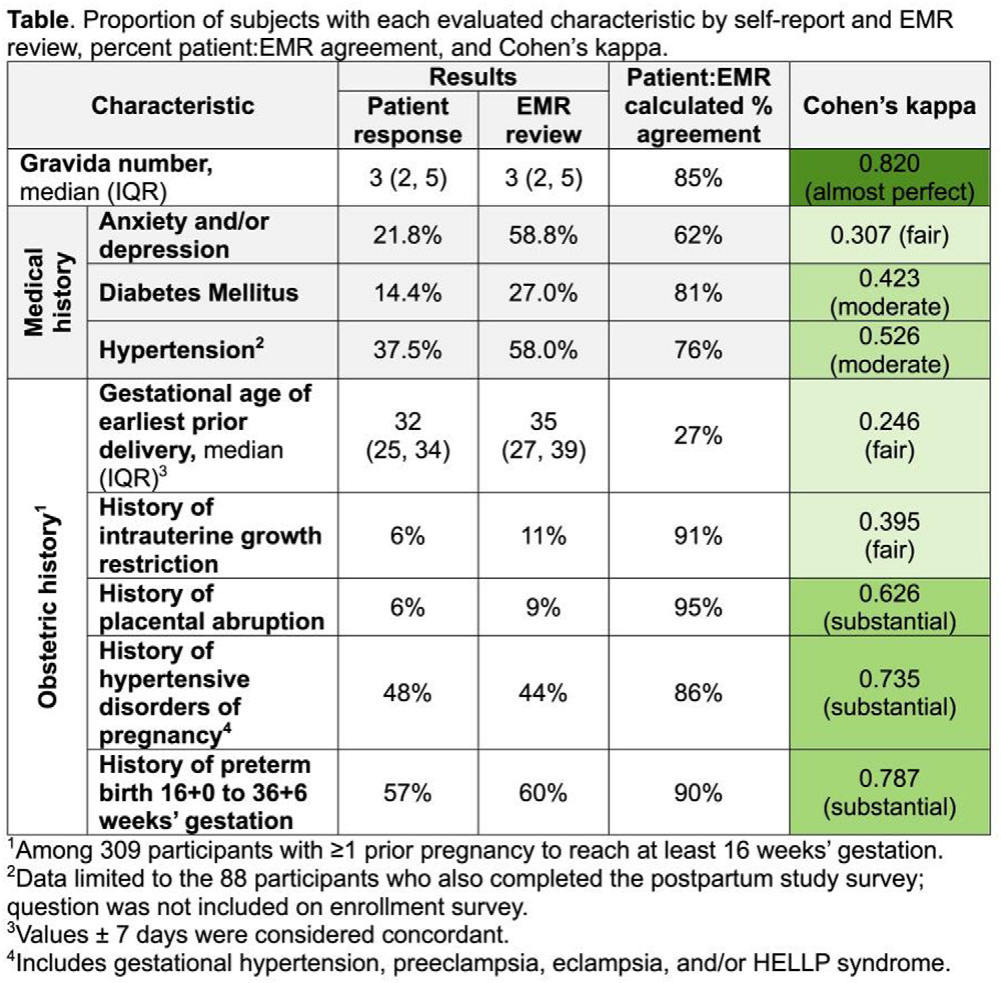

## 193 | Discrimination due to personal characteristics and adverse pregnancy outcomes

Sarah Heerboth^1^, Emily Gascoigne^2^, Joy McNeal^1^, Ebony Carter^1^, Hadley Hartwell^1^, Rebecca Fry^1^, Tracy A. Manuck^1^

^1^University of North Carolina, Chapel Hill, North Carolina; ^2^UNC Chapel Hill School of Medicine, Carrboro, North Carolina

17:15–18:30

**Objective**: Racial discrimination (discrim) is consistently associated with adverse pregnancy outcomes. It is less certain if discrim based on other personal characteristics carries similar risk. We sought to quantify whether perceived reasons for discrim are associated with adverse pregnancy outcomes.

**Study Design**: Secondary analysis of a prospective pregnancy cohort enriched for those at high *a priori *risk of early PTB. Participants identifying as Black, White, and/or Hispanic, with singleton, non‐anomalous gestations were recruited < 22 weeks, 2017‐2022. Participants completed the Krieger Scale to assess discrimin in 9 domains (e.g., school, work, public); for each, if discrim was reported, respondents completed a followup question re: the perceived discrim reason (options = (1) race, ethnicity, ancestry, ± skin color; (2) education ± income; (3) gender; (4) age, religion, height/weight, sexual orientation, ± disability; (5) ‘other.’ The primary outcomes were PTB < 35 weeks and a diagnosis of plac‐comps (preeclampsia, gestational hypertension, birthweight < 10% for gestational age and sex, ± placental abruption). Secondary outcomes included PTB < 32 & < 37 weeks and delivery gestational age.

**Results**: 435 individuals (43% Black, 38% White, 19% Hispanic) were included; 108 (25%) delivered < 37, 58 (13%) < 35, and 31 (7%) < 32 weeks; 71 (16%) had plac‐comps. Surveys were completed at a mean of 17.1 (IQR 16.0‐19.7) weeks. Discrim was more common among those with PTB < 37 (48% vs. 35%, *p* = 0.02) and < 35 (56% vs. 36%, *p* = 0.003) but not < 32 weeks (50% vs. 38%, *p* = 0.18) and among those with plac‐comps (57% vs. 35%, *p* < 0.001). Discrim was most common for ‘other’ reasons and race/ancestry/skin color, *Table*. Distinct patterns in reasons for discrim were noted for PTB & delivery GA outcomes (associated with discrimin due to ‘other’ factors) and plac‐comps (associated with overall amounts of discrim and discrim due to race and education ± income).

**Conclusion**: The reasons why an individual has experienced discrimination are important when considering risk for various adverse pregnancy outcomes.

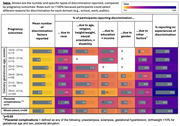

## 194 | Postpartum health self‐evaluation and use and barriers to medical care

Sarah Heerboth^1^, Emily Gascoigne^2^, Alison M. Stuebe^3^, Hadley Hartwell^1^, Rebecca Fry^1^, Tracy A. Manuck^1^

^1^University of North Carolina, Chapel Hill, North Carolina; ^2^UNC Chapel Hill School of Medicine, Carrboro, North Carolina; ^3^UNC School of Medicine, Chapel Hill, North Carolina

17:15–18:30

**Objective**: The postpartum period is critical for maternal health, but postpartum health status and healthcare access/utilization vary widely. We sought to assess self‐evaluation of health, medical care utilization, and to identify barriers to receiving care among individuals at high risk for adverse pregnancy outcomes.

**Study Design**: Planned primary analysis of a prospective observational cohort of patients at high‐risk for adverse pregnancy outcomes at a tertiary University hospital, 2017‐2021. Participants were followed longitudinally throughout pregnancy and completed a pp survey including questions regarding overall health (5‐point Likert scale), plans to improve their health (if average, fair, or poor overall health self‐rated), and medical care use and barriers. Data were analyzed by t‐test, chi‐square, and Fisher's exact. Regression models were used to identify factors associated with fair/poor health self‐eval.

**Results**: 122 participants (29% Black, 61% White, 11% Hispanic) completed the pp survey at a median 21 weeks pp (IQR 9, 56). 2% rated their health as fair, 17% as poor, 47% as average, 29% as very good, and 5% as excellent. Those with fair/poor health were more likely to have chronic HTN, had higher systolic and diastolic BPs during pregnancy, and were more likely to be anxious/depressed compared to those with ≥ average health, Table. Difficulty scheduling and financial concerns were the leading barriers to receiving medical care but did not differ by reported health status. In logistic regression models, HTN (aOR 4.9, 95% CI 1.5, 16), anxiety ± depression (aOR 5.9, 95% CI 1.7, 21.0), and pre‐pregnancy BMI (aOR 1.07, 95% CI 1.01‐1.12) were significantly associated with fair/poor health self‐eval.

**Conclusion**: In this high‐risk cohort, despite frequent medical co‐morbidities, high obesity, and relatively high pp visit utilization, only 38% of participants were planning to try to improve their health in the next month. Nearly half reported ≥1 barrier to medical care. Identifying tangible solutions to improve OB and primary care access and motivation to change is crucial.

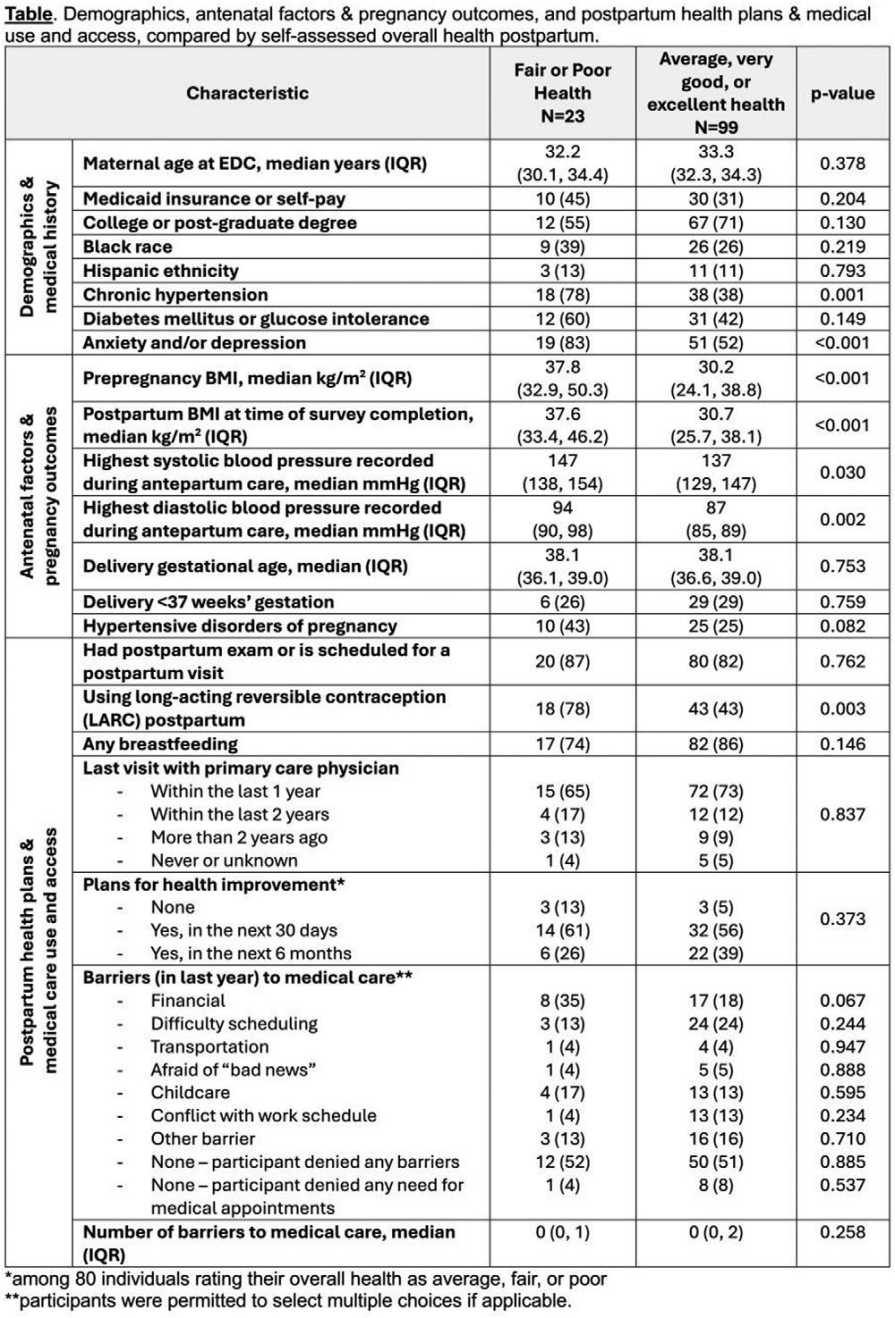

## 195 | Antepartum maternal hemodynamic assessment and Perinatal outcomes

Beatrice Melis^1^, Anna Luna Tramontano^2^, Martina Benuzzi^1^, Giuseppe Chiossi^1^, Isabella Neri^1^, Antonio Ragusa^3^, Fabio Facchinetti^4^, Antonio La Marca^5^, Francesca Monari^1^

^1^University of Modena, Modena, Emilia‐Romagna; ^2^University of Napoli, Napoli, Campania; ^3^University of Bologna, Bologna, Emilia‐Romagna; ^4^University of Modena and Reggio Emilia, Modena, Emilia‐Romagna; ^5^Azienda Ospedaliero–Universitaria Policlinico di Modena, Obstetrics and Gynaecology Unit, Modena, Emilia‐Romagna

17:15–18:30

**Objective**: Pregnancy requires adaptive changes of the cardiovascular system. Appropriate adaptation favors Vaginal Delivery (VD). The UltraSonic Cardiac Output Monitor (USCOM) is a non‐invasive method providing clinically relevant information on maternal cardiocirculatory health. The aim of the study is to investigate possible correlations between maternal hemodynamic status at term and operative delivery, either cesarean section in labour or operative vaginal delivery (CS/OVD).

**Study Design**: Singleton, at term pregnancies were recruited between Jan 23 and Mar 24, at two University Hospitals, in Italy. Women planned for cesarean section were excluded. Assessment of maternal hemodynamic status with USCOM occurred antepartum, between 37+0 and 41+0 weeks of gestation. Obstetric‐neonatal outcomes were acquired from the electronic medical records. Continuous variables were expressed as mean and standard deviation (SD), and categorical variables were expressed as total count and percentage (%). A multivariate logistic regression was performed.

**Results**: Three‐hundreds and forty‐eights women were recruited. Their features, cardiovascular parameters and Perinatal Outcomes are shown in Table 1. ART and analgesia in labor were prevalent in women who delivered with CS/OVD. These latter also showed higher Cardiac Index (CI) that VD group. At multivariate analysis, ART (*p* = 0.01) and CI < 2.9 (*p* = 0.007) were independent risk factors for predicting CS∖OVD, once adjusted for analgesia in labor and ethnicity.

**Conclusion**: A low cardiac index (CI), the ratio of cardiac output from left ventricle in one minute to body surface area, predicts operative delivery, in addition to ART. Larger studies are warranted to hypothesize that such hemodynamic marker would become a predictive tool for labors at increased hypoxic‐ischemic risk.

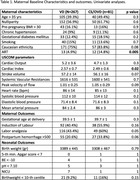

## 196 | Neuroimaging in monochorionic gestation: Is there any marker for worse neurological prognosis?

Nieves Mendez sanchez^1^, Raul Arenal^2^, Beatriz Herrero Ruiz^2^, Laura Sotillo^2^, Marta Campillo^2^, Roberto Rodríguez^2^, Jose Luis Bartha Rasero^2^, Eugenia Antolin Alvarado^2^

^1^Hospital Universitario La Paz, Madrid, Madrid; ^2^Hospital Universitario La Paz, Hospital Universitario La Paz, Madrid

17:15–18:30

**Objective**: It is widely known that monochorionic twin pregnancies carry a higher risk of complications during gestation, primarily due to the hemodynamic imbalances that can occur as both fetuses share the same placenta. This leads to increased perinatal mortality and morbidity, especially neurological, and adds to the inherent risk of prematurity associated with twin pregnancies. Our objetives were to evaluate if there are differences in certain neuroimaging parameters (NSG and MRI) among newborns (NB) based on their chorionicity and to explore the added value of fetal magnetic resonance in the assessment of the central nervous system.

**Study Design**: A prospective observational study was conducted, recruiting dichorionic and monochorionic twin pregnancies with and without complications. Neurosonography and magnetic resonance imaging were performed around 32 weeks of gestation. Both the presence of lesions and the quantitative assessment of different structures of the central nervous system were evaluated.

**Results**: Out of a total of 100 pregnancies, 66 were monochorionic, and among them, 26 presented some complication. Neurosonographic study detected two fetuses (1.1%) with central nervous system lesions. Magnetic resonance imaging was pathological in 5 fetuses (3.3%), three of whom had normal neurological examination and transfontanellar ultrasound after birth. All fetuses with central nervous system lesions corresponded to complicated monochorionic pregnancies.

Regarding the biometric study of different central nervous system structures, cephalic biometrics and corpus callosum length were lower in donor and growth restriction fetuses. The fetuses from uncomplicated monochorionic pregnancies showed a larger cisterna magna.

**Conclusion**: Although variations in the dimensions of some structures of the central nervous system have been described in fetuses of complicated monochorionic pregnancies, their clinical significance is yet to be determined. Albeit magnetic resonance imaging appears to increase the detection rate of lesions, these lesions seem to have little clinical impact.

## 198 | Repeatability of measurements obtained in fetalHQ assessment by sonographers with limited experience with the method

Jakub Mlodawski^1^, Anna Zmelonek‐Znamirowska^2^, Grzegorz Swiercz^1^, Marta Mlodawska^1^

^1^Jan Kochanowski University in Kielce, Kielce, Swietokrzyskie; ^2^Jan Kochanowski University in Kielce, Jan Kochanowski University in Kielce, Swietokrzyskie

17:15–18:30

**Objective**: The purpose of this study was to assess interobserver variability in the analysis of FetalHQ by two observers using previously obtained 2D four‐chamber‐view loops.

**Study Design**: Two experienced sonographers, each with more than five years of experience in conducting midtrimester screenings but only three months of experience with FetalHQ analysis, undertook this evaluation on 2D four‐chamber‐view loops previously acquired by one of them (excluding further patient involvement) in accordance with the study's methodology. Each evaluator measured the global strain index (GSI), global longitudinal strain for both the right and left ventricles (GLS‐RV, GLS‐LV), and fractional area change for the right and left ventricles (FAC‐RV, FAC‐LV).

**Results**: This investigation encompassed 20 patients between 19 and 22 weeks of gestation, with a median gestational age of 20 weeks and 2 days. All analyses were adjudged suitable for evaluation by the investigators. In 50% of the analyzed cases, the heart's apex was oriented perpendicularly to the ultrasound beam, 25% were oblique, and in the remainder, the apex was upward. The global strain index (GSI) exhibited good reproducibility with an interclass correlation coefficient (ICC) of 0.82 (95% CI = 0.78–0.94). Moderate reproducibility was observed for GLS‐LV (ICC = 0.72, 95% CI 0.61‐0.79) and FAC‐LV (ICC = 0.70, 95% CI = 0.67–0.73), whereas poor reproducibility was noted for GLS‐RV (ICC = 0.48 95% CI = 0.42–0.54) and for FAC‐RV (ICC = 0.41 95% CI = 0.39–0.49).

**Conclusion**: The utilization of FetalHQ software for the analysis of cardiac function and morphology by sonographers with minimal experience in its application demonstrates limited repeatability, especially regarding parameters pertinent to the right ventricle. Conversely, the assessment of global cardiac morphology via GSI showcases high repeatability.

## 199 | Trends in severe maternal morbidity and obstetric comorbidities among Illinois births from 2016‐2023

Mugdha V. Mokashi^1^, Lynn M. Yee^2^, Joe M. Feinglass^2^

^1^Northwestern Medical Center, Chicago, IL; ^2^Northwestern University Feinberg School of Medicine, Chicago, Illinois

17:15–18:30

**Objective**: The objective was to analyze trends in severe maternal morbidity (SMM) and route‐specific complications of delivery in Illinois hospitals from 2016 to 2023. We also evaluated International Classification of Diseases, 10th Revision (ICD‐10) coding for chronic and pregnancy‐related comorbidities over this time period to model the association of risk factors with complication incidence.

**Study Design**: This retrospective population‐based cohort study analyzed delivery admission data from 127 hospitals using the Illinois Hospital Association Comparative Health Care and Hospital Data Reporting Services database from January 2016 to June 2023. Primary outcomes were incidence of SMM and route‐specific delivery complications. Secondary outcomes included incidence of chronic and pregnancy‐related comorbidities. Poisson regression was used to estimate incidence rate ratios for SMM, logistic regression was used to estimate odds ratios for route‐specific delivery complications, and model standard errors were adjusted for clustering by hospital.

**Results**: In this cohort of 988,480 deliveries, the overall SMM rate was 1.6%; SMM rose from 1.6% in 2016 to 2.0% in 2023. Vaginal delivery complications (7.3% overall) increased 24% and cesarean complications (10.9% overall) increased 39% (Figure 1a). Hypertensive disorders of pregnancy (HDP) and anemia, both of which increased over the study period, were significant risk factors for SMM and delivery complications. Over the study period, there were also increases in ICD‐10 coded obesity (7.8% to 22.3%), anxiety (3.1% to 10.4%), gestational diabetes (4.2% to 5.5%), depression (2.5% to 6.6%), and other chronic comorbidities (4.7% to 7.4%) (Figure 1b). Non‐Hispanic Black patients had over double the SMM rate (2.6%) compared to non‐Hispanic White patients (1.1%).

**Conclusion**: From 2016 to 2023, the rates of SMM and delivery complications in Illinois increased alongside increases in coding for chronic and pregnancy‐specific comorbidities. Public health and perinatal quality initiatives must address increasingly prevalent comorbidities upstream of SMM to improve maternal health.

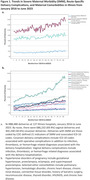

## 200 | Cervical length as a predictor of spontaneous preterm birth: Individual Participant Data (IPD) meta‐analysis

Kelly M. Hughes^1^, Mason Aberoumand^2^, Lene Seidler^3^, Rui Wang^1^, Ben W. Mol^4^

^1^Monash University, Monash University, Victoria; ^2^University of Sydney, University of Sydney, New South Wales; ^3^NHMRC Clinical Trials Centre, University of Sydney, Sydney, New South Wales; ^4^Monash University, Clayton, Victoria

17:15–18:30

**Objective**: Spontaneous preterm birth is the leading cause of perinatal and early childhood mortality worldwide. Studies have shown conflicting results on the ability of mid‐trimester transvaginal sonographic cervical length to predict spontaneous preterm birth (SPTB). Aggregate data meta‐analyses are limited by shortcomings in primary literature. The purpose of this individual participant data (IPD) meta‐analysis was to quantify how well mid‐trimester cervical length can predict spontaneous preterm birth in asymptomatic women with singleton pregnancy, and to assess whether other factors may aid in prediction.

**Study Design**: Medline, Embase, Cochrane, and LILACS were searched to identify eligible studies examining the association between mid‐trimester transvaginal sonographic cervical length and SPTB in asymptomatic with singleton pregnancy. The last search was performed on 30/9/2020; two reviewers screened all studies for inclusion. The primary outcome was STPB < 37 weeks. Risk of bias was assessed with QUIPS by two reviewers. A two‐stage IPD meta‐analysis in a Cox proportional hazard model was performed using cervical length as a continuous variable.

**Results**: The search yielded 3884 unique records; 462 underwent full‐text screening. 22/72 eligible studies were obtained and analysed (*n* = 88 498). Each additional millimetre of cervical length was associated with a 7% (5‐8) decreased hazard of SPTB < 37 weeks’ gestation, and a 10% (9‐12) decrease in odds of SPTB < 34 weeks. When cervical length was 25mm at 20 weeks’ gestation, there was a 13% chance of delivering before 37 weeks, 4.1% before 34 weeks, 3% before 32 weeks and 1% before 28 weeks.

**Conclusion**: The prognostic value of cervical length for spontaneous preterm birth in singleton pregnancy is in a continuous scale. SPTB is not predicted by a single significant cervical length cut‐off, nor is risk eliminated by a longer cervix. Clinical cut‐offs should be determined by an expected treatment effect.

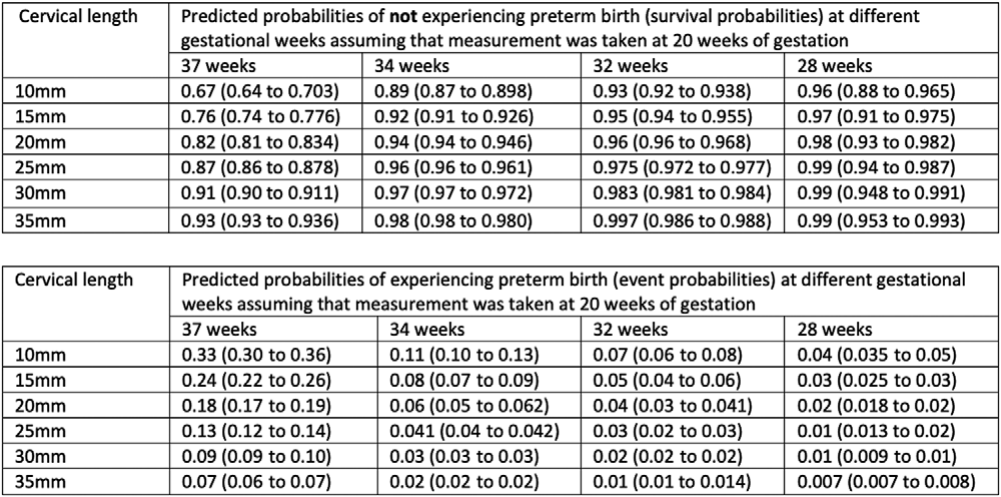

## 201 | Publishers' post‐publication response to concerns regarding false data in women's health

Ben W. Mol^1^, Siddharth Shivantha^2^, Jeremy Nielsen^3^, Jim Thornton^4^

^1^Monash University, Clayton, Victoria; ^2^Monash University, PRAHAN, Victoria; ^3^Monash University, Monash University, Victoria; ^4^Nottingham University, Nottingham University, England

17:15–18:30

**Background**: There is increasing concern about the integrity of clinical research. The post‐publication review process allows the assessment of problematic papers after publication, but the effectiveness and efficiency of this assessment (Committee on Publication Ethics (COPE)) has not been assessed.

**Objective**: To evaluate the effectiveness and efficiency of the post‐publication review system in papers published in women's health.

**Study Design**: Cohort study.

**Methods: **Between 2017‐2023, we wrote to editors and publishers of women's health journals about potential problematic papers and requested an investigation according to the criteria established by the Committee of Publication Ethics (COPE). For each potentially problematic paper, we tabulated the trial characteristics (type of study, publication year, etc), the timeline of e‐mail correspondence with journals and the outcome of the process [retraction, expression of concern (EOC), correction and no wrongdoing found].

**Results**: We wrote to editors and publishers of 732 precarious papers ((58% randomised clinical trials; 42% cohort studies) published in 185 journals. Until October 2023, 183 (25%) of 732 papers received an outcome (95 papers being retracted, 64 papers received EOC, four corrections, 20 no wrongdoing found) (total retraction/EoC *n* = 159, 87%), mostly for problems attributed to containing false data (78%). The response rate per journal to issue these outcomes varied between 0% to 100%. One journal retracted 18 articles out of the 19 completed investigations, with the remaining one being challenged for obvious problems (mainly even numbers) It took a median time of 32 months for editors and publishers to issue any outcome. The median time to retraction was 40 months, and the median time taken to issue an EOC was 52 months.

**Conclusion**: Concerns regarding integrity in clinical research are much more widespread than initially assumed. The post‐publication assessment process guided by COPE has many shortcomings, including absent timelines. This has immediate consequences for guidelines, meta‐analyses and patient safety.

## 202 | Effects of maternal stress on placental 11βHSD2 mRNA and protein in Fetal Growth Restriction

Francesca Carasi‐Schwartz^1^, Elizabeth A. Morgan^2^, Perrie O'Tierney‐Ginn^1^, Tomoko Kaneko‐Tarui^1^, Sharon Geerts^3^, Chloe Benichou^3^

^1^Tufts Medical Center, Boston, Massachusetts; ^2^University of Connecticut, EAST LONGMEADOW, MA; ^3^Baystate Medical Center, Springfield, Massachusetts

17:15–18:30

**Objective**: To determine if higher maternal stress levels, assessed by the Perceived Stress Scale (PSS) questionnaire and maternal hair cortisol, correlate with decreased 11βHSD2 in the placenta.

**Study Design**: PSS questionnaire, maternal hair samples, and placenta samples of 18 control and 6 FGR participants were collected. Cortisol was quantified in maternal hair samples by ELISA. RNA and total protein were extracted from biopsies across the villous surface of the placenta, and expression was evaluated using RT‐qPCR and a capillary‐based automated Western blot system (WES), respectively. Gene and protein expression between groups was analyzed using Mann‐Whitney t‐tests. 11βHSD2 gene and protein expression were tested for association with clinical and demographic variables using Spearman's rank correlation tests and multivariate linear regression analyses.

**Results**: FGR placentas had significantly greater 11βHSD2 mRNA expression (*p* = 0.0004), and an inverse correlation to neonatal birthweight (*p* = 0.0071). 11βHSD2 protein expression was not significantly different between the groups, but was associated with birthweight when gestational age and neonatal sex were adjusted for (*p* = 0.0196) by multivariate linear regression analysis. There was a significant positive correlation between PSS and maternal hair cortisol (*p* = 0.012), however, there was no significant relationship between PSS or maternal hair cortisol and 11βHSD2 mRNA or protein expression.

**Conclusion**: Placental 11βHSD2 mRNA and protein levels are differently related to birthweight, suggesting a compensatory genetic response to protein defects or a potential negative feedback mechanism whereby low protein levels trigger increased gene expression. Sample size may be a limitation for detectability of associations between 11βHSD2, maternal stress, and FGR. Future research should incorporate fetal cortisol for a better understanding of maternal cortisol's effects on fetal growth.

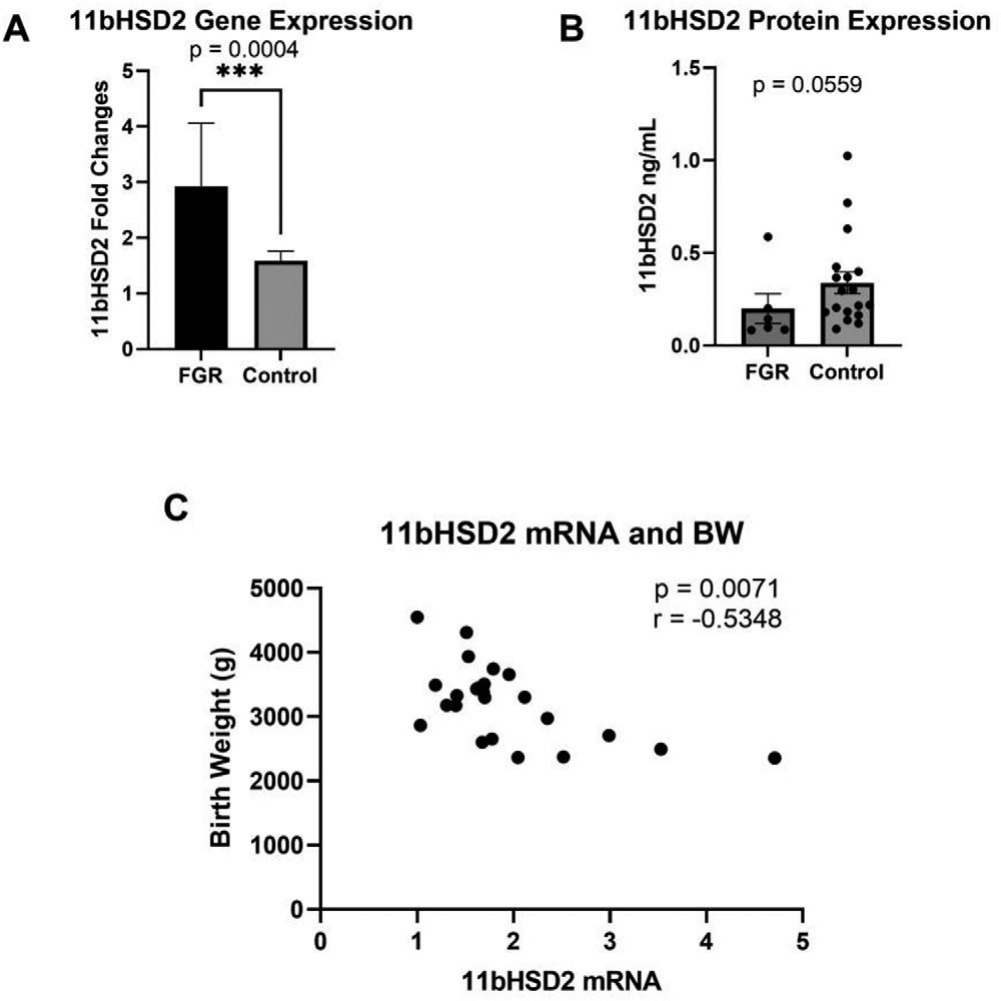

## 203 | COVID‐19 exposure in pregnancy and newborn anthropometric measurements

Kristen L. Moriarty^1^, Kelsey Manfredi^1^, Pascale Carrel^1^, Emma Kryzanski^1^, David A. Schwartz^2^, Andrea D. Shields^3^

^1^UCONN Health, Farmington, Connecticut; ^2^Perinatal Pathology Consulting, Atlanta, Georgia, Farmington, Connecticut; ^3^University of Connecticut Health, Avon, Connecticut

17:15–18:30

**Objective**: COVID‐19 is inconsistently linked to fetal growth restriction and small for gestational age (SGA). Viral infections like Zika virus infection during pregnancy have been linked to abnormal newborn anthropometric measurements, specifically microcephaly without SGA or low birth weight. There is limited data on the impact of COVID‐19 on specific anthropometric measurements. This study investigates the relationship between trimester‐specific SARS‐CoV‐2 infection and growth metrics.

**Study Design**: A retrospective cohort enrolled 276 patients. Inclusion criteria: a) singleton pregnancy; b) singleton birth occurring in our institution between April 28, 2020, and December 31, 2022; and c) maternal COVID‐19 infection diagnosed via PCR. Exclusion criteria: < 12 years of age, major fetal anomalies, and fetal loss < 15 weeks. Outcomes: comparison of newborn anthropometric measurements (length, weight, and head circumference (HC) at birth), Ponderal Index, and development of SGA between SARS‐CoV‐2‐infected and uninfected patients. Maternal and neonatal characteristics were descriptively summarized, and multivariate analyses and linear regression models were performed.

**Results**: Baseline maternal demographics did not significantly differ among the uninfected and infected cohorts. Compared to the uninfected cohort, infection with COVID‐19 was associated with smaller infant HC percentile (z scores) after adjusting for gestational age at the anatomy scan (*p* = 0.023). COVID‐19 diagnosed in the third trimester was associated with a lower neonatal HC compared to newborns of uninfected patients (β = −0.38 [0.38 SD lower], 95% CI −0.65 to −0.10, *p* = 0.024). There was no significant difference among the cohorts for birth length, weight, or diagnosis of SGA.

**Conclusion**: We found that COVID‐19 infection in the third trimester was associated with a lower neonatal head circumference without associated SGA. The cause underlying this association is unknown. Further research to determine the risk of neurotropic fetal infection by SARS‐CoV‐2, like ZIKA's effect on the fetal immune system leading to microcephaly, is urgently needed.

## 204 | Racial disparities in COVID‐19 symptomatology, severity and treatment in pregnancy

Kristen L. Moriarty^1^, Kelsey Manfredi^1^, Emma Kryzanski^1^, Pascale Carrel^1^, Lucas Godoy^1^, Chia‐Ling Kuo^1^, Andrea D. Shields^2^

^1^UCONN Health, Farmington, Connecticut; ^2^University of Connecticut Health, Avon, Connecticut

17:15–18:30

**Objective**: This study investigates racial disparities in COVID‐19 vaccination, symptomology, hospitalization rates, and treatment in pregnancy.

**Study Design**: A retrospective cohort study with inclusion criteria: 1) COVID‐19 during pregnancy and ongoing pregnancy beyond 14 weeks, 2) initiation of prenatal care between February 2020 and August 2022, 3) and age 12 or older. Exclusion criteria: 1) major fetal anomalies and delivery at another hospital. Primary outcomes: COVID‐19 vaccination status, symptoms and hospitalization at diagnosis, and COVID‐19 treatment by race. Secondary measures: adverse maternal, fetal and neonatal outcomes. Descriptive statistics were performed and were analyzed using appropriate analyses.

**Results**: 147 COVID‐19 pregnancies. When baseline demographics were stratified by race, African American (31.7 ± 9.0) and “Other” (31.1 ± 9.0) races had higher pre‐pregnancy body mass indexes (*p* = 0.021). African American (29.2 ± 5.3) and “Other” (28.2 ± 5.2) races were younger at delivery compared to the total cohort (*p* = 0.006). The African American cohort was also more likely to have a baseline history of type 2 diabetes (4, 10.5%, *p* = 0.075). Overall, the rate of severe maternal COVID‐19 infection requiring ICU admission was 5.0%. When stratified by race (Black, Asian, Other, White), we found that White (58%, *n* = 35) and Asian (80%, *n* = 8) women were more likely to be vaccinated than Black women (37%, *n* = 14) (*p* = 0.013). Black women were more likely to report cough than other races (*p* = 0.024). Although not significant, Black women were less likely to receive COVID‐19 treatments than White women (89% vs. 75%, *p* = 0.154). Maternal co‐morbidities were similar between races.

**Conclusion**: Our data compares to nationally reported ICU admission rates. Compared to prior health disparities reported in COVID‐19 in non‐pregnant patients, our study found a difference in vaccination rates and symptomology reported between races. A trend was observed between COVID‐19 treatment utilization amongst races, with White and “Other” races more likely to receive treatment.

## 205 | Outcomes and prognostic factors of very early Preterm Premature Rupture of Membrane

HYE RIM OH^1^, Sun Min Kim^2^, Byoung Jae Kim^3^

^1^Seoul Metropolitan Boramae Medical Center/ SEOUL, Seoul Metropolitan Boramae Medical Center/ SEOUL, Seoul‐t'ukpyolsi; ^2^101 Daehak‐ro, Jongno‐gu, Seoul, Seoul‐t'ukpyolsi; ^3^Seoul Metropolitan Government Seoul National University Boramae Medical Center, Seoul, Seoul‐t'ukpyolsi

17:15–18:30

**Objective**: Preterm premature rupture of membrane (PPROM) that develops extremely earlier gestational age is one of the devastating obstetric complications that can lead to serious adverse perinatal outcomes. The aim of this study was to evaluate the natural course and prognostic factors of early PPROM with conservative management before the first 20 weeks of pregnancy.

**Study Design**: This was a retrospective cohort study with singleton pregnant women who were diagnosed of PPROM between 10^th^ and 19^th^ weeks of gestation. After diagnosis, the patients were hospitalized and received conservative management with antibiotics. Perinatal and maternal outcomes were collected, and the data were analyzed by dividing the patients into two groups: those with adequate remained amniotic fluid (AF) after PPROM and those with oligohydramnios.

**Results**: A total of 30 patients were enrolled, and 20 patients maintained adequate AF even after PPROM, and 10 patients were diagnosed with oligohydramnios. The gestational age at delivery was higher, and the interval from diagnosis of PPROM to delivery was also longer in the adequate AF group than in those with oligohydramnios (38.0 vs 18.5 weeks of gestation and 153.5 vs 8 days, *p*‐value < 0.001 each). Excluding the 3 who did not deliver at our institution, in 12 out of 17 patients (70.6%) whose AF was maintained adequately, evidence of ROM was disappeared after conservative treatment with antibiotics and full‐term delivery without complication was achieved. On the other hand, the majority with oligohydramnios after PPROM had poor prognosis. Among the 10 patients, five underwent abortion, three were discharged against medical advice, and only two experienced preterm delivery at 24 and 28 weeks of gestation each.

**Conclusion**: The remained AF volume after diagnosis of PPROM could be an important prognostic factor for perinatal outcomes. It is also noteworthy that more than two‐thirds women with normal AF were delivered at term even though they had PPROM at extremely early gestational age.

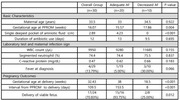

## 206 | The impact of war on adverse pregnancy and neonatal outcomes

Sharon Oron^1^, Shayna MIODOWNIK^2^, Tamar Wainstock^3^, Eyal Sheiner^3^

^1^Soroka University Medical Center, beer sheva, HaDarom; ^2^soroka medical center, beer sheva, HaDarom; ^3^Department of Obstetrics and Gynecology, Soroka University Medical Center, Ben‐Gurion University of the Negev, Beer‐Sheva, Israel., Beer Sheva, HaDarom

17:15–18:30

**Objective**: Conflict and war may have a dramatic effect on the health and well‐being of the population at‐risk; it therefore provides a unique opportunity to study the impact of a universally stressful exposure on pregnancy. Following the outbreak of the “Iron Swords” war in Israel on October 7, 2023, the population is experiencing significant levels of acute stress. Studies regarding the influence of war‐related stress on pregnancy outcomes are limited. This study aims to investigate the influence of the “Iron Swords” war on adverse pregnancy and neonatal outcomes among the population in Southern Israel.

**Study Design**: Pregnancy and neonatal outcomes of women who delivered during the “Iron Swords” war were compared to women who delivered during the same period, one year previously. As the war is currently ongoing, we collected data from its onset, October 7, 2023, up to November 11, 2023. The study data was retrieved from primary tertiary Medical Center located in Southern Israel.

**Results**: During the exposure period, the “Iron Swords” war, there were 1682 deliveries, which were compared to 1804 deliveries which took place during the same period in 2022. The rates of women presenting with premature rupture of membranes (PROM) (OR 1.84, 95% CI 1.09‐3.09, *p* = 0.021), meconium‐stained amniotic fluid (OR 3.92, 95% CI 1.58‐9.71, *p* = 0.003), and low 5‐minute Apgar scores (less than 7; OR 1.69, 95% CI 1.01‐2.87, *p* = 0.049), were significantly higher among those who delivered during the war. Background characteristics were comparable between the two periods (Table).

**Conclusion**: Acute war stress is a risk factor for adverse perinatal outcomes, including increased rates of PROM, meconium‐stained amniotic fluid and low 5‐minute Apgar scores, which has reportedly been associated with increased fetal morbidity. This report further supports the fact that, in the realm of public health, it is essential to recognize pregnant women as a distinct group requiring special attention, particularly during emergency situations.

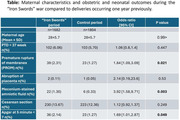

## 207 | The association between obstetric delivery volume on neonatal and pregnancy outcomes

Ciaran S. Phibbs^1^, Katy Backes Kozhimannil^2^, Stephanie A. Leonard^3^, Elliott K. Main^1^, Molly Passarella^4^, Scott Lorch^4^

^1^Stanford University, Stanford, California; ^2^University of Minnesota, Minneapolis, Minnesota; ^3^Stanford University, Palo Alto, California; ^4^Children's Hospital of Philadelphia, Philadelphia, Pennsylvania

17:15–18:30

**Objective**: Numerous clinical fields including surgical care and high‐risk infants exhibit volume‐outcome associations. There is little information on the association between obstetric delivery volumes and adverse pregnancy outcomes, which can inform optimal perinatal care systems. We examined the association of annual hospital delivery volume and neonatal and pregnancy outcomes.

**Study Design**: Retrospective cohort analysis of all 2000‐2020 birth certificates linked to death certificates and maternal and infant hospital discharge records in 5 US states (*n* = 12,086,389). Outcome was a delivery with a significant maternal or infant complication. Multivariable logistic regression determined the association of annual hospital delivery volume with the dyad outcome, adjusting for sociodemographic or medical factors, year, and hospital as a fixed effect.

**Results**: The risk‐adjusted rates of an adverse pregnancy outcome in either the birth parent or infant were higher among hospitals with obstetric volume below 4000 deliveries (OR < 1000 births 1.04, 95 % CI 1.02‐1.06). Similar associations were seen for adverse parental or infant outcomes separately (Figure 1). Risk‐adjusted rates of adverse outcomes were highest with volumes < 1250 deliveries, with a lower risk of adverse outcomes until 6000 deliveries, after which rates increased.

**Conclusion**: Adjusting for patient casemix and hospital fixed effects, lower obstetric delivery volume was associated with higher rates of adverse maternal or infant outcomes, especially for the smallest hospitals. While smaller delivery services can be consolidated in urban areas, this isn't possible in rural areas. Effective strategies are needed to support low volume hospitals in rural areas to optimize the health of pregnancy patients and infants.

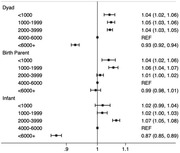

## 209 | Pregnancy outcome in women with thyrotoxicosis following radioactive iodine therapy or anti‐thyroid drug

Prasanta kumar. Pradhan^1^, Sangeeta Yadav^2^, Neeta Singh^2^

^1^SGPGIMS, Lucknow, Uttar Pradesh; ^2^Sanjay Gandhi Post Graduate Institute of Medical Sciences, Sanjay Gandhi Post Graduate Institute of Medical Sciences, Uttar Pradesh

17:15–18:30

**Objective**: To analyze maternal and fetal outcome in women receiving radioactive iodine or medical therapy for Graves’ disease.

**Study Design**: Pregnant women with known case of Graves’ disease attending the antenatal clinic between January 2009 to Dec 2023 were included in this study. Group I included those who had received radio‐active iodine and group II were those with antithyroid drug treatment before pregnancy. Presence of thyroid receptor antibody (TRAb), thyroid peroxidase antibody (TPO), TSH and fT4, Course of pregnancy and perinatal outcome were compared between both the groups.

**Results**: There were 132 pregnancies in 124 women with Graves, disease during this period. Three women were diagnosed during pregnancy and 11 had surgical treatment and were excluded. The cause of hyperthyroidism was Graves’ disease in 84.6 %, AFTN in 5.4% and TNG in 9%. Treatment with RAI therapy was in 53 women (Gr I) and medical therapy in 57 women (Group II). Age, presence of TRAb and TPO was comparable in both the groups (Table). Overall, 83% women had autoantibodies. Missed abortion, congenital malformation, preterm birth, GDM, pre‐eclampsia, fetal growth restriction was noted similar in both groups. One woman, a known case of Graves’ disease presented with hypothyroidism and fetal hydrops at 28 weeks and was on levothyroxine for replacement. She had high TRAb titer. She had spontaneous preterm delivery and had neonatal death. In her subsequent pregnancy, she was treated with levothyroxine and antithyroid drugs following diagnosis of fetal hyperthyroidism from 12 weeks onwards. Doses were increased with advancing gestation. She was delivered at 36 weeks and newborn was treated with anti‐thyroid drug for 4 weeks. The child at 1 year follow up is euthyroid with normal growth and milestones.

**Conclusion**: There is no difference in pregnancy outcome in women treated with RAI or medical therapy for thyrotoxicosis. Rarely women can become hypothyroid with circulating TRAb which may cross placenta and cause fetal thyrotoxicosis which can be managed by both levothyroxine and propyl‐thiouracil with maternal and fetal monitoring.

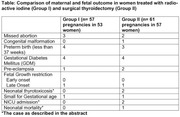

## 212 | The impact of fetal biometry and maternal subpubic angle in large for gestational age fetuses

Ilma F. Carbone^1^, Rasha Kamel^2^, Francesca M.P. Gigli^3^, Valentina Romagnoli^3^, Enrico Iurlaro^1^, Vittorio Parodi^3^, Benedetta Gallicola^3^, Giuseppe Cappuccio^3^, Giovanna Esposito^3^, Enrico M. Ferrazzi^4^

^1^Unit of Obstetrics, Department of Mother and Child Area, Fondazione IRCCS Ca' Granda Ospedale Maggiore Policlinico, Mangiagalli Center, Milan, Italy, Milan, Lombardia; ^2^Department of Obstetrics & Gynecology, Maternal‐Fetal Medicine unit, Cairo University, Cairo, Egypt, Al Qahirah, Al Qahirah; ^3^University of Milan, Milan, Lombardia; ^4^Fondazione IRCCS Ca' Granda Ospedale Maggiore Policlinico, Obstetrics Unit, Department of Woman Child and Neonate, Milan, Italy, Milan, Lombardia

17:15–18:30

**Objective**: We hypothesized that the evaluation of the relationship between fetal biometry (the passenger) and maternal pelvimetry (the passage), could allow for the identification of pregnant women who could benefit from induction of labor at early term in a population at high risk of fetopelvic disproportion.

**Study Design**: This was a prospective multicenter study on pregnant women with a singleton pregnancy, with an estimated fetal weight above the 85^th^ percentile, at 35‐37 weeks of gestation, not affected by gestational diabetes.

Induction of labor was scheduled between 38+4 and 39+4 weeks in the case of an estimated fetal weight >95^th^ percentile, or in the case of an estimated fetal weight > 85^th^ percentile and at least one of the following unfavorable indices: maternal height < 155 cm, subpubic angle < 100°.

The whole cohort was analyzed to sort out which clinical condition or ultrasound index was significantly associated with unplanned operative delivery. The ratio between estimated fetal weight and subpubic angle and the ratio between head circumference and subpubic angle were investigated and compared between women who underwent uneventful vaginal delivery and those who underwent unplanned operative birth.

**Results**: The rate of unplanned operative births, was higher in women who underwent induction of labor than in the expectant management group (51.6% *versus* 9.8%; *p* < 0.01). Of the whole 227 patients included in this analysis, uneventful vaginal delivery occurred in 127 (56.0%). At multivariate analysis, an EFW/SPA ratio ≥ 34.67 and a HC/SPA ratio ≥ 3.29 remained significantly associated with a higher risk of unplanned operative birth, and the ORs were 2 to 3 times higher than in cases below these cut‐offs.

**Conclusion**: This study has proved the association of unplanned operative delivery of large fetuses for labor arrest with the ratios between fetal biometry and maternal subpubic angle. This novel approach, has the potential to improve our ability to identify women for whom an early planned delivery may be beneficial compared to those in which an expectant management can be considered.

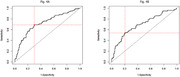

## 213 | Association between time from uterine incision to delivery and maternal and neonatal outcomes

Adam Schettler^1^, Emily Labruna^2^, Lauren Gilgannon^2^, Gabrielle Smith^2^, Donald Dudley^2^

^1^University of Virginia, Charlottesville, VA; ^2^University of Virginia, Charlottesville, Virginia

17:15–18:30

**Objective**: To assess the association between time from uterine incision to delivery > = 3 minutes with 5‐minute neonatal APGAR score < 7, NICU admission, and operative quantitative blood loss (QBL) at an academic teaching institution.

**Study Design**: Patients who underwent cesarean delivery of a singleton live birth at our institution from January 2022–July 2023 were included. Medical records were abstracted for clinical variables, including history of cesarean delivery (CD), scheduled/unscheduled CD, QBL, 5‐minute APGAR scores, and NICU admission. Means and SDs were calculated for all continuous variables. Univariate chi‐squared tests were performed for 5‐minute neonatal APGAR score > = 7/ < 7, NICU admission, and QBL.

**Results**: Our sample included 1387 couplets. The mean time from uterine incision to delivery was 1.72 minutes (SD+‐1.35). Of the 1387 births, 16 required NICU admission (1.2%) and 144 infants received a 5‐minute APGAR score of < 7 (10.3%). The average QBL during delivery in our sample was 1010ml (SD+/‐542ml). Time from incision to delivery > = 3 minutes was not associated with APGAR score < 7 (*p* = 0.079) or NICU admission (*p* = 0.41). The QBL during delivery was likewise not associated with time from incision to delivery > = 3 minutes (*p* = 0.68). Scheduled and unscheduled CDs had similar rates of deliveries with time from incision to delivery > = 3 minutes (*p* = 0.61), while repeat CD were more likely than primary CD to have times > = 3 minutes (*p*‐0.0006).

**Conclusion**: Time from hysterotomy to delivery had no impact on 5‐minute neonatal APGAR scores, NICU admission, or operative blood loss. Longer durations of time to delivery are not associated with harm to the infant or mother as reflected by these criteria, which provides reassurance to learners, teaching faculty, and patients.

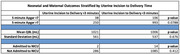

## 214 | Standardizing post‐cesarean analgesia regimens is insufficient at decreasing racial disparities in postoperative pain management

Carrie Sibbald^1^, Amy Godecker^2^, Tiffany Green^3^, Kathleen M. Antony^4^

^1^University of Wisconsin Madison, Madison, WI; ^2^University of Wisconsin Hospitals and Clinics, Madison, Wisconsin; ^3^University of Wisconsin Madison, Madison, Wisconsin; ^4^University of Auckland, Auckland, Auckland

17:15–18:30

**Objective**: This study sought to evaluate the effects of an institution‐wide implementation of a post‐cesarean standardized analgesia protocol on racial disparities previously identified in a single birth center. Our hypothesis was that a standardized protocol would decrease the disparities previously observed, improving the postoperative cares for patients undergoing cesarean section.

**Study Design**: This study was a retrospective cohort of 2340 cesarean deliveries at a single institution. Two periods of time were compared: July 1, 2017 to April 8, 2018 and June 19, 2018 to April 30, corresponding to periods before and after implementation of a standardized post‐operative analgesia protocol. Delivery during the rollout period, opioid use disorder, and incomplete data were excluded. The primary outcome was average pain score 24 hours postoperative and secondary outcome was cumulative dose of morphine milligram equivalents (MME) before and after protocol implementation.

**Results**: 2009 patients were included in the study. Pain disparities pre‐protocol demonstrated higher pain scores for Black and Hispanic patients compared to White patients. Average MME use was not different. Asian race used significantly less MME, a trend that persisted after the protocol. Post‐protocol, Black patients continued to have higher pain scores without a difference in MME used and the change in the racial difference post‐protocol was insignificant. Hispanic patients no longer demonstrated a significantly higher pain score when compared to White patients, but the change in the difference was not significant. Average MME use between Hispanic and White patients was also not significantly changed by protocol implementation. These results are summarized in table 4.

**Conclusion**: This study demonstrated that standardized postoperative analgesia regimens are insufficient to decrease racial disparities in post‐cesarean pain control. These results highlight the complexity of maternal racial disparities and the need for expansive protocol changes and multifaceted approaches to improving postpartum care for racial minorities in the US.

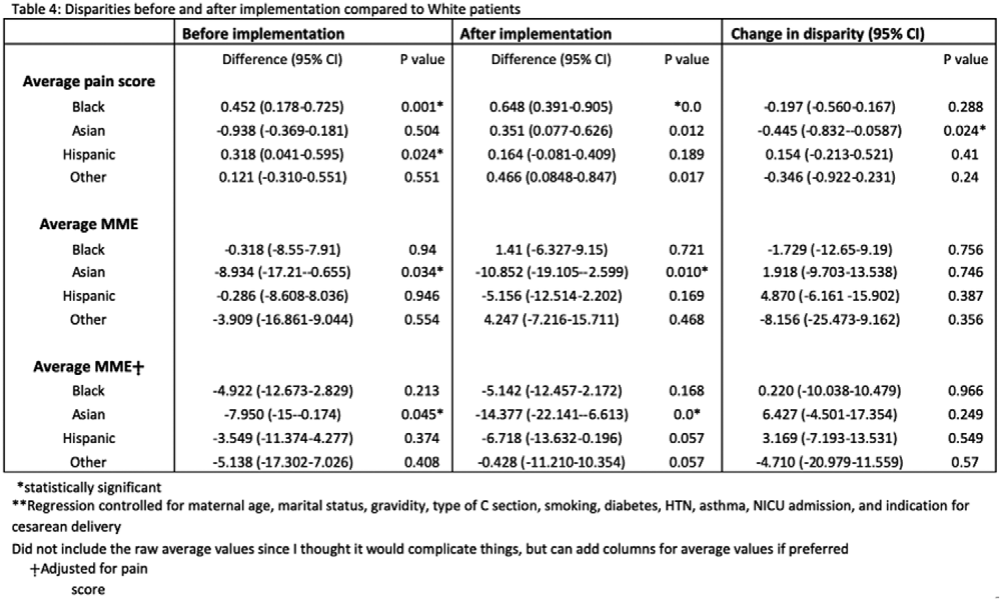

## 215 | Comprehensive analysis of scRNA‐seq and bulk RNA‐seq reveals transcriptional signatures of macrophages in ICP

Mi Tang^1^, Jianghui Cai^2^, Liling Xiong^2^, Xuejia Gong^3^, Li Fan^2^, Xiaoyu Zhou^2^, shasha Xing^2^, Xiao Yang^2^

^1^Chengdu Women and Children's Central Hospital, University of Electronic Science and Technology of China, Chengdu, Sichuan; ^2^Chengdu Women and Children's Central Hospital, Chengdu Women and Children's Central Hospital, Sichuan; ^3^University of Electronic Science and Technology of China, University of Electronic Science and Technology of China, Sichuan

17:15–18:30

**Objective**: Intrahepatic cholestasis of pregnancy (ICP) is a disorder that characterized by maternal pruritus, abnormal liver function, and an elevation in total bile acid concentrations during pregnancy. Immune factors have been recognized as playing a vital role in the mechanism of ICP. However, the underlying mechanisms regulating dysfunctional immune cells and immune genes remain to be fully elucidated.

**Study Design**: Single‐cell RNA sequencing and bulk RNA sequencing data of the placenta were downloaded from the SRA database. The AUCell package, Monocle package and SCENIC package were utilized to explored immune cell activity, cell trajectory and transcription factor, respectively. GO, KEGG, and GSEA were employed to explore potential biological mechanisms. Cell‐cell communications were further investigated using the CellChat package. RT‐PCR, and western blot were used to verify the analysis.

**Results**: In placenta cells, macrophages were found to be significantly increased in ICP. Additionally, macrophages exhibited the highest immune gene score and were divided into four subclusters (MF1‐4). Our analysis revealed significant elevations in MF2, associated with LPS response and antigen presentation, and MF4, associated with TNF and cytokine production. MF3 displayed an anti‐inflammatory phenotype. MF1, closely related to ribosomes and proteins, exhibited a sharp decrease. Although ICP maintained an anti‐inflammatory state, macrophage trajectories showed a gradual progression toward inflammation. Subsequently, we confirmed that cytokine‐ and chemokine‐related signaling pathways were emphasized in macrophages. Within the CXCL signaling pathway, the increased expression of CXCL1 in macrophages can interact with CXCR2 in neutrophils, potentially inducing macrophage infiltration and leading to an inflammatory response and cellular damage.

**Conclusion**: In conclusion, we firstly revealed the transcriptional signatures of macrophages in ICP and discovered a tendency toward an inflammatory state. This study also provides new evidence that the CXCL1‐CXCR2 axis may play an important role in the pathogenesis of ICP.

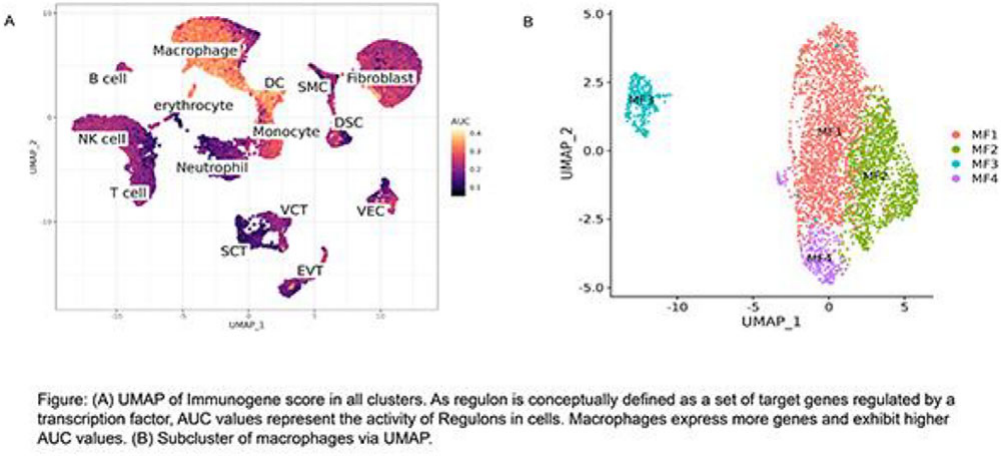

## 216 | Low circulating SPINT1 at 36 weeks gestation predicts fetal growth restriction and poor neonatal outcomes

Stephen Tong^1^, Roxanne Hastie^2^, Sue Walker^3^, Danica Idzes^4^, Teresa M. MacDonald^3^, Ping Cannon^2^, Vi Nguyen^2^, Parinaz Mehdipour^2^, Lucy Bartho^5^, Alexandra Roddy Mitchell^2^, Tu'uhevaha Kaitu'u‐Lino^5^

^1^Department of Obstetrics, Gynaecology and Newborn Health, University of Melbourne, Heidelberg, Victoria, Victoria; ^2^University of Melbourne, University of Melbourne, Victoria; ^3^Department of Obstetrics, Gynaecology and Newborn Health, University of Melbourne, Melbourne, Victoria; ^4^Department of Obstetrics, Gynaecology and Newborn Health, University of Melbourne, University of Melbourne, Mercy Perinatal, Victoria; ^5^Department of Obstetrics, Gynaecology and Newborn Health, University of Melbourne, University of Melbourne, Victoria

17:15–18:30

**Objective**: Fetal growth restriction (FGR, birthweight < 3^rd^ centile) has a 10+ fold risk of perinatal death. Current management, fundal height measurement and selective ultrasound, only identifies 14‐32% of fetuses < 3^rd^ centile weight. We did a large cohort study to examine whether low circulating SPINT1 at 36 weeks gestation predicts fetal growth restriction and poor neonatal outcomes.

**Study Design**: We measured SPINT1 in 2900 plasma samples prospectively collected at 36 weeks’ gestation. We measured SPINT1 using an ultra‐sensitive assay we generated. Using a cut‐off of SPINT1 levels < 10^th^ centile to define a positive test, we examined whether low SPINT1 predicts: 1) neonates < 3^rd^ birthweight centile or 2) a composite of poor neonatal outcomes (see table). We also examined whether SPINT1 makes ultrasound more accurate.

**Results**: Compared to higher levels, a low SPINT1 ( < 10^th^ centile) at 36 weeks gestation was associated with a 4.8 fold risk of a neonate < 3^rd^ centile birthweight. This cut off identified 42.7% of all < 3^rd^ birthweight centile infants (42.7% sensitivity, 95% CI 40.9‐44.5%). Low SPINT1 had a 2.5 fold risk of a composite of poor neonatal outcomes. Significantly, the positive predictive value was 26%, meaning 1 in 4 had a poor neonatal event.

The table shows the risk of adverse outcomes at different levels of SPINT1. There is a 21‐fold difference in risk of a birthweight < 3^rd^ centile between highest and lowest levels of SPINT1 (14.1% vs 0.6%).

An estimated fetal weight < 10^th^ centile on ultrasound and low SPINT1 ( < 10^th^ centile) identified fetuses at extremely high‐risk of placental insufficiency: 17.9 fold risk (95% CI 7.5‐42.7) of a neonate < 3^rd^ centile and a 52.6% positive predictive value (95% CI 4.9‐61.6%) of poor neonatal outcomes (*n* = 1407, sub‐cohort who had third trimester ultrasounds).

**Conclusion**: Low SPINT1 ( < 10^th^ centile) at 36 weeks gestation identified pregnancies at high risk of FGR and adverse neonatal outcomes. It has promise as a universal screening test that flags fetuses at risk, where monitoring and timed birth may reduce perinatal deaths and poor neonatal outcomes.

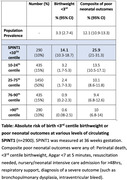

## 218 | Vaginal Cerclage, we must standardise technique

Graham S. Tydeman^1^, Laura Stirrat^2^, megan hall^3^, Natalie Suff^3^, lea hansen^4^, Andrew Shennan^5^, florence tydeman^3^, julie Glavind^4^

^1^NHS Fife, NHS Fife, Scotland; ^2^Edinburgh Royal Infirmary, Edinburgh Royal Infirmary, Scotland; ^3^Kings College London, London, England; ^4^Aarhus University Hospital, Aarhus, Midtjylland; ^5^King's College London, King's College London, England

17:15–18:30

**Objective**: Preterm birth is the biggest cause of neonatal mortality & morbidity. Despite being the most common procedure to prevent preterm labour, vaginal cerclage remains contentious. We aimed to establish: variation in vaginal cerclage by experts on a simulator; whether exposure to an information package altered performance & to determine best practice

**Study Design**: Two observational studies on a simulator, measuring suture height, depth & tension by CT; a randomised trial of 53 experts at a European conference & a Delphi Consensus. Our first (published) study of 28 UK experts showed huge variation in height of suture, tension & depth of bites. Higher placement with small needle diameter (*p* = 0.05).

**Results**: At a European congress, randomisation to information package showed a lower chance of entering the cervical canal (*p* = 0.03) with more thread left on the surface (*p* = 0.02) but no effect on height or tension. 29 practitioners at a Nordic conference showed equal variation. Delphi consensus concluded sutures should be as high as possible, not enter cervical canal & tension to result in 30% reduction in area. Combining all 3 studies: 134 procedures by 110 practitioners from 22 Countries with 26(20%) achieving < 5% reduction in area (41% was achieved by some), 18(13%) had one or more bite deep enough to enter the cervical canal and 33 (25%) achieved a mean height of suture less than half way up the cervix.

**Conclusion**: By using a validated simulator, biological variation is removed so differences must be due to differences in technique. Higher sutures are likely to be more effective & given the agreement that avoiding the cervical canal is important as well as some tension in the suture is necessary, it is a concern that so many procedures failed to achieve these simple objectives. If transferrable to real women, many thousands may, at best, have ineffective and, at worst, damaging procedures. What represents a perfect cerclage is not determined but before we undertake more research studies on real women we must at least establish that best practice is achievable on a simulator

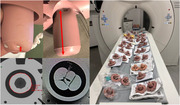

## 219 | Daytime variations in urinary protein to creatinine ratio among pregnant women with and without hypertention

Esther Maor‐Sagie^1^, Cami lehavi^2^, Mordechai Hallak^3^, Rinat Gabbay‐Benziv^3^

^1^Hille Yaffe medical center, Hillel Yaffe medical canter, Hefa; ^2^Hillel Yaffe Medical Center, Rappaport Faculty of Medicine, Technion‐ Israel Institute of Technology, Hefa; ^3^Hillel Yaffe Medical Center, Hillel Yaffe medical center, Hefa

17:15–18:30

**Objective**: Physiological proteinuria occurs in approximately 2% of normal pregnancies, attributed to heightened Glomerular Filtration Rate and tubular reabsorption. Around 30% of pregnancies are prone to hypertensive disorders, potentially leading to adverse pregnancy outcomes. Traditionally, proteinuria was quantified through 24‐hour urine collection. Recently, Urine Protein Creatinine Ratio (Upcr) gained popularity for its ease of use and strong correlation with 24‐hour collection. However, Upcr measurement involves random sampling, susceptible to diurnal variations in urinary protein excretion. Hence, our study aims to evaluate and compare the diurnal variability of Upcr in healthy and hypertensive pregnancies.

**Study Design**: A retrospective analysis of all Upcr evaluations during 1.1.2021 −1.3.2024. Evaluations were sent as part of routine assessment of proteinuria in the Maternity ER or FetoMaternal department after positive urine stick for protein in a single center In Israel. Individuals with pregnancies that were eventually complicated by any hypertensive disorders were compared to Individuals with pregnancies without hypertensive disorders. Every woman was counted once, at her first testing only. Kruskall Walis test was used to compare Upcr levels according to the time of the day.

**Results**:–During the study, 13,054 deliveries occurred, with 10% undergoing Upcr evaluations. Among them, 35.8% were diagnosed with hypertensive disorders. Daytime tests outnumbered nighttime tests (1121 vs. 196). As anticipated, Individuals with hypertensive disorders had higher BMI and Upcr. Demographic factors showed no significant differences between day and night testing. However, those with hypertensive disorders had notably higher daytime Upcr values compared to nighttime (0.75 vs. 0.39, *p* = 0.02), while no difference was observed in those without hypertensive disorders.

**Conclusion**: Upcr has diurnal variability which is significant among women with hypertensive disorder in pregnancy.

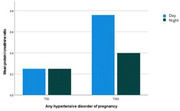

## 220 | Fetal sex and the development of future Type 2 Diabetes — A 5‐year cohort study

Esther Maor‐Sagie^1^, Liron Livny^2^, Mordechai Hallak^3^, Amir Naeh^3^, Yoel toledano^4^, Rinat Gabbay‐Benziv^3^

^1^Hille Yaffe medical center, Hillel Yaffe medical canter, Hefa; ^2^Hillel Yaffe mediacal center, Hillel Yaffe medical center, Hefa; ^3^Hillel Yaffe Medical Center, Hillel Yaffe medical center, Hefa; ^4^Meuhedet HMO, Meuhedet HMO, Hefa

17:15–18:30

**Objective**: Gestational Diabetes (GDM) poses a recognized risk for subsequent Type 2 Diabetes (T2DM) development. Emerging evidence suggests potential variations in pregnancy outcomes based on fetal sex. Some studies indicate that individuals carrying male fetuses exhibit diminished insulin sensitivity, reduced β‐cell function, and elevated glucose levels in oral glucose tolerance tests compared to those carrying females. However, mechanisms remain unknown, and findings are inconclusive. Therefore, our study aimed to assess the long‐term risk of T2DM after GDM according to fetal sex in up to five years postpartum.

**Study Design**: Retrospective analysis of pregnant individuals with up to 5 years of post‐pregnancy follow‐up. Data sourced from Meuhedet HMO's computerized laboratory system, cross‐tabulated with the Israeli National Diabetes Registry. Inclusion criteria were singleton pregnancy, and GDM diagnosis based on 2 abnormal values at GTT or GCT above 200mg/dl. Women with multifetal pregnancy or prior diagnosis of diabetes were excluded. Maternal characteristics, obstetrics data, and T2DM were stratified by fetal sex. Univariant analysis was followed by survival analysis adjusted to confounders.

**Results**: 1734 patients entered the analysis, of whom 829 carried male and 808 carried female fetuses. No statistically significant variances were observed between these groups in terms of Body Mass Index (BMI), Glucose Challenge Test (GCT), and Glucose Tolerance Test (GTT) values. Additionally, the incidence of T2DM did not exhibit a significant discrepancy between the male and female fetus groups, with rates of 7.1% and 6.2% respectively (*p* = 0.48). Multivariate analysis indicated that maternal age, Hyperlipidemia, and fetal sex did not significantly influence the likelihood of developing future T2DM.

**Conclusion**: The subsequent risk of developing T2DM following GDM showed no variation based on fetal sex.

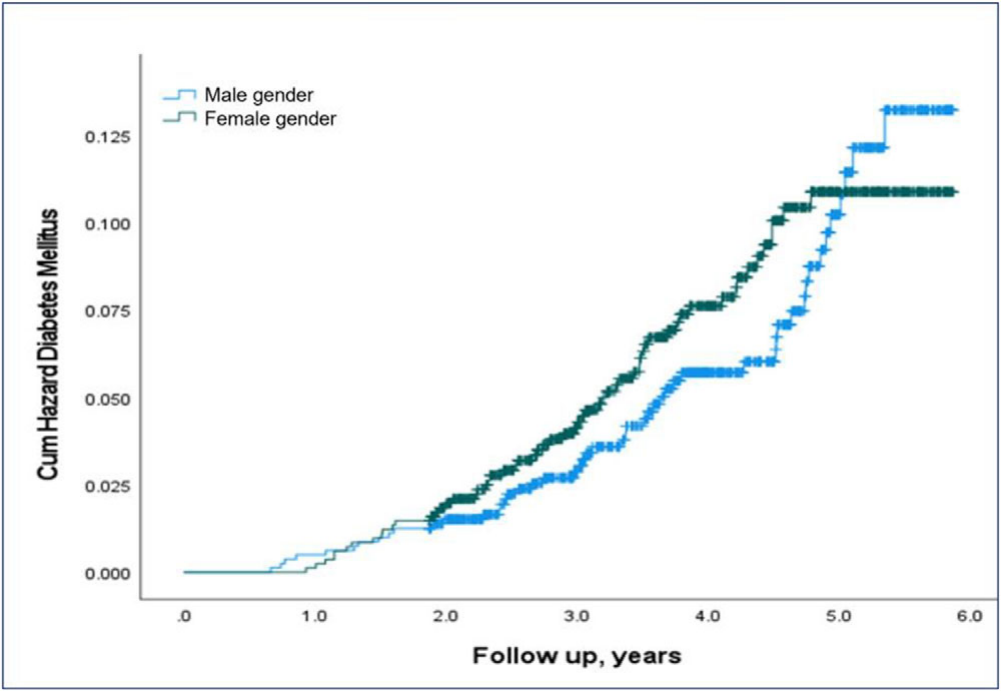

## 221 | Lipid profile before, during and after pregnancy–time to normality

Esther Maor‐Sagie^1^, Mordechai Hallak^2^, Yoel toledano^3^, Rinat Gabbay‐Benziv^2^

^1^Hille Yaffe medical center, Hillel Yaffe medical canter, Hefa; ^2^Hillel Yaffe Medical Center, Hillel Yaffe medical center, Hefa; ^3^Meuhedet HMO, Meuhedet HMO, Hefa

17:15–18:30

**Objective**: Hypercholesterolemia is one of the main risk factors for atherosclerosis. Physiological hypercholesterolemia occurs during pregnancy following the weeks of gestation, becoming 40–50% greater than the initial value at the end of pregnancy to meet the growing fetus's high cholesterol demands. However, data about postpartum maternal lipid profile normalization is scarce. Thus, in this study, we aimed to evaluate the maternal lipid profile before, during, and up to 18 months after delivery in order to evaluate its time to normalization.

**Study Design**: A retrospective analysis of parturients between 1.1.2017 to 31.12.2020 from Mehuedet HMO–one of four mandatory national public health organizations in Israel. Lipid profiles measured 6 months before, during, and 18 months after pregnancy were compared. Statistical analysis included frequency analysis and paired samples T‐test to assess for differences before and after delivery.

**Results**: 88,611 parturients were included. LDL, HDL, triglycerides, and total cholesterol levels were increased during pregnancy and gradually decreased after it: LDL–93.4(79‐111.5), 104(85‐123), 95.4(78.6‐113.8), and 94.4(79‐114.4); HDL–53(46‐62), 56(48‐65), 53(46‐62), and 52(45‐61); Triglycerides‐ 70(53‐97), 74(54‐104), 72(52‐104) and 75(55‐108); Total cholesterol 164(146‐186), 178(155.3‐202), 166(147‐188) and 164(146‐191); at 6 months before, and at 6, 12 and 18 months after pregnancy, mg/dL, median (IQR).

Paired samples t‐test demonstrated the return of LDL and HDL levels to their pre‐pregnancy state within 1 year after delivery (*p*‐value < 0.05 for 6 and 12 months postpartum). Levels of total cholesterol and triglycerides remained high even after 18 months postpartum compared to their pre‐pregnancy state (*p* < 0.001 for all).

**Conclusion**: Maternal hypercholesterolemia persists in the postpartum period. LDL and HDL levels return to the pre‐pregnancy state at least a year after delivery while the triglycerides and total cholesterol levels remain higher than the pre‐pregnancy state even 18 months postpartum.

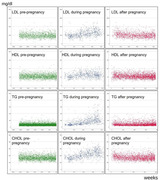

## 222 | Lipid profile in multifetal vs singleton pregnancy

Esther Maor‐Sagie^1^, Mordechai Hallak^2^, Amir Naeh^2^, Yoel toledano^3^, Rinat Gabbay‐Benziv^2^

^1^Hille Yaffe medical center, Hillel Yaffe medical canter, Hefa; ^2^Hillel Yaffe Medical Center, Hillel Yaffe medical center, Hefa; ^3^Meuhedet HMO, Meuhedet HMO, Hefa

17:15–18:30

**Objective**: Physiological hypercholesterolemia occurs during pregnancy following gestational weeks, becoming 40–50% greater than the initial value at the end of pregnancy. In Multifetal pregnancy, both the placenta and the physiological demands of the pregnancy increase. However, data regarding the lipid profile in multifetal pregnancy is scarce. We aimed to evaluate and compare lipid profiles before, during and after pregnancy between singleton to multifetal pregnancy.

**Study Design**: A retrospective analysis of parturients between 1.1.2017 to 31.12.2020 from Mehuedet HMO–one of four mandatory national public health organizations in Israel. Lipid profiles including low‐density lipoprotein (LDL), high‐density lipoprotein (HDL), triglycerides and cholesterol levels measured six months before, during, and eighteen months after pregnancy were compared between singleton and multifetal pregnancies. Statistical analysis included univariate followed by multivariate analysis with adjustment for maternal age, BMI, gestational weight gain, and gestational diabetes status.

**Results**: 88,611 parturients were included, 1298 were multifetal pregnancies and 87,322 were singleton. Parturients in the multifetal group were older and gained more weight during pregnancy. In univariate analysis, triglycerides and cholesterol were higher in multifetal compared to singleton pregnancies. LDL was higher only after the 2^nd^ trimester and HDL was lower only in the 3^rd^ trimester. In the multivariate analysis, only cholesterol and LDL levels in the 2^nd^ trimester were found to be significantly higher in multifetal pregnancy.

**Conclusion**: Multifetal pregnancy doesn't necessarily induce higher lipid profile compared to singleton. Whether it is a physiological mechanism to provide the higher fetal mass and what are the implications of increased maternal lipid levels during pregnancy on future maternal morbidity are yet to be evaluated in prospective research.

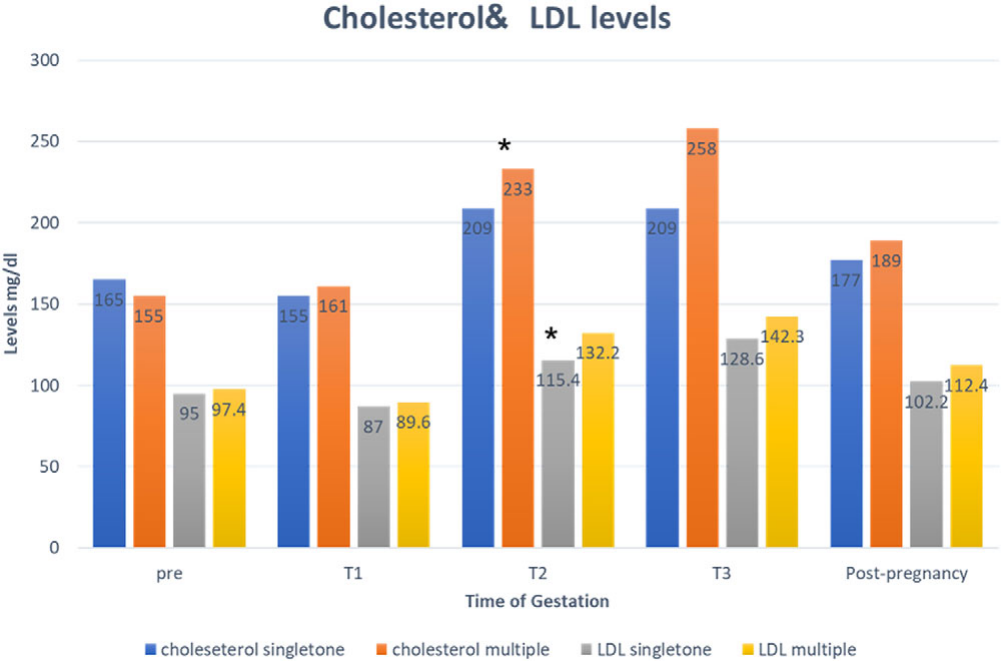

## 223 | Impact of prenatal engagement in a virtual maternity program on perinatal healthcare utilization

Ian J. Hooley^1^, Marta Bralic Kerns^2^, Charissa A. Qian^2^, Isabelle Von Kohorn^2^

^1^Pomelo Care Inc, Pomelo Care, NJ; ^2^Pomelo Care, Jersey City, New Jersey

17:15–18:30

**Objective**: To compare perinatal risk and healthcare utilization between patients who engaged prenatally in Pomelo Care's 24/7 virtual maternity care program and those who did not.

**Study Design**: This retrospective study included patients (*n* = 534) if they: (A) were enrolled in one specific health plan contracted with Pomelo Care in the US, (B) delivered a liveborn infant between 10/20/22 and 9/22/23, (C) had linkable mom‐baby medical claims. Mother and baby medical claims that occurred between 280 days before birth and 60 days after birth were analyzed for emergency department (ED) visits, inpatient stays not related to birth, and neonatal intensive care unit (NICU) admissions. The study compared patients who utilized Pomelo Care's services at least four weeks before birth with those who did not. HHS‐HCC risk adjustment factors (RAF) were calculated for mothers and compared across groups. The study used bivariate analyses to estimate significance of differences. IRB waiver of informed consent was obtained.

**Results**: Patients in the prenatally engaged with Pomelo Care cohort (*n* = 74) had higher risk than those in the comparator cohort (*n* = 460) (HHS‐HCC RAF 4.41 vs 4.38, difference = 1%, *p* = 0.858), significantly lower ED utilization (378 vs 696 visits per 1,000 births, reduction = 46%, *p* = 0.003), significantly lower NICU admissions (81 vs 193 per 1,000 births, reduction = 58%, *p* = 0.020), and lower non‐birth inpatient stays (95 vs 107 per 1,000 births, reduction = 11%, *p* = 0.781) (see Table).

**Conclusion**: The results suggest that engagement in Pomelo Care's virtual maternity program is associated with significantly lower emergency department utilization and NICU admissions, and may be associated with a reduction in non‐birth related inpatient stays, in the setting of an equivalent or higher average patient risk profile. This suggests virtual maternity care can be an approach to perinatal health management and outcomes improvement. Future studies could benefit from employing a matched cohort design or randomized trials to further validate these findings.

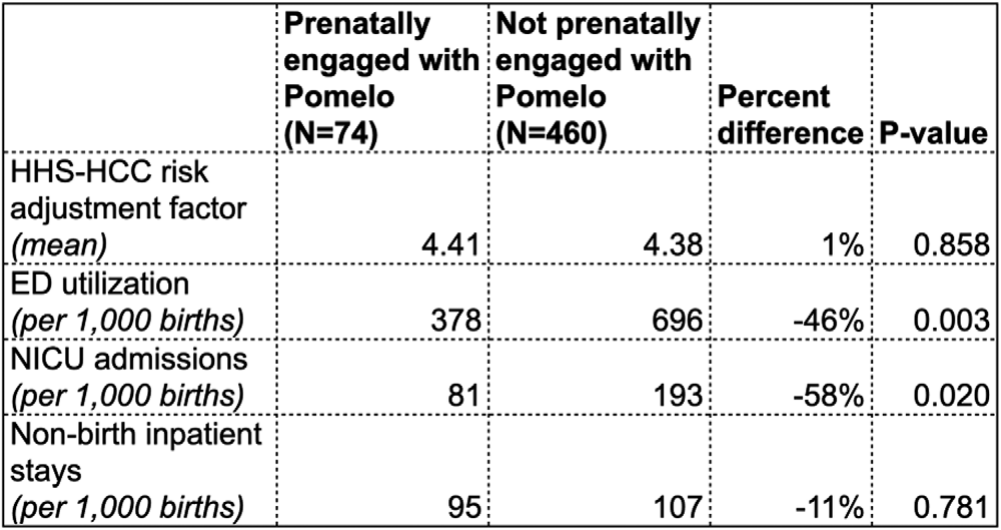

## 224 | The value of coagulation profiles in suspected placental abruption: A retrospective chart review

Mariana McCune^1^, Anjali Mishra^2^, Chase Michael Irwin^3^, Emily Anne Galloway^3^, RE Garfield^3^, Linda R. Chambliss^4^

^1^University of Arizona College of Medicine Phoenix, tucson, AZ; ^2^Creighton University School of Medicine, Omaha, Nebraska; ^3^University of Arizona College of Medicine Phoenix, Phoenix, Arizona; ^4^Valleywise Medical Center, Phoenix, Arizona

17:15–18:30

**Objective**: To investigate if coagulation panels (PT, aPPT, and fibrinogen) are predictive of placental abruption.

**Study Design**: At two tertiary care centers in Phoenix, AZ, ICD‐10 codes were used to identify charts of adult pregnant patients admitted to the ED for trauma. Primary endpoints included PT, aPPT, fibrinogen, and blood product administration. Secondary endpoints were need for eminent fetal delivery, route of delivery, and Apgar score at 5 minutes. Lab results were categorized as normal/abnormal based on institutional reference ranges.

Patients were stratified by whether their record contained ultimate diagnosis of abruption or receipt of blood transfusion and the frequency and proportion of normal versus abnormal lab values reported. Fisher's Exact test with Benjamini & Hochberg's adjustment for multiple comparisons were applied to determine if the proportion of abnormal labs significantly differed across strata. Secondary endpoints were narratively reported due to inconsistent chart documentation.

**Results**: Of 421 patients, mean age was 28.79 ± 6.61 and predominant race and ethnicity was Hispanic (69.58%, 70.52%). No significant differences were found in normal/abnormal values between abruption (*n* = 30) and non‐abruption (*n* = 391) groups for aPPT (*p* = 0.99), PT (*p* = 1.0), or fibrinogen (*p* = 0.33). Among those receiving blood transfusions, fibrinogen levels differed significantly (*p* < 0.05), while PT (*p* = 0.11) and aPTT (*p* = 0.94) did not. Abnormal/normal results were not significantly associated with higher likelihood of eminent delivery or difference in 5 minute Apgar scores.

**Conclusion**: No significant difference was found for abnormal coagulation panels between groups with or without placental abruption, or for those who received blood products and those who did not. This implies limited utility of these panels in diagnosing or managing placental abruption. Notably, abnormal laboratory findings persisted even in cases without abruption, casting doubt on their diagnostic value for this condition. Limitations of our study include retrospective design, reliance on chart documentation, and limited generalizability.

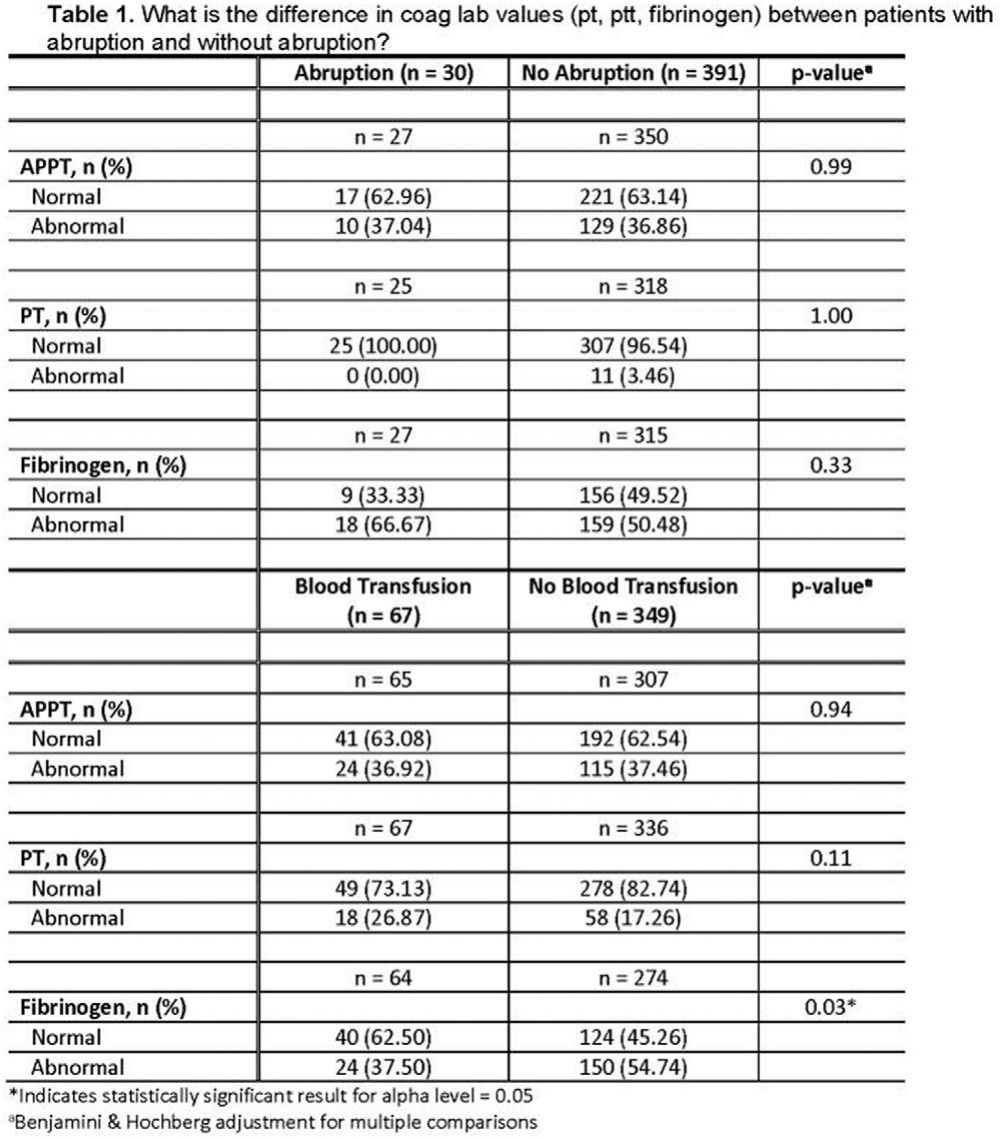

## 225 | Changes in fetal and maternal parameters in early‐onset fetal growth restriction from diagnosis to delivery

Mette van de Meent^1^, Wessel Ganzevoort^2^, Sanne J. Gordijn^3^, Elizabeth M. W. Kooi^4^, Wes Onland^5^, Johannes J. J. Duvekot^6^, René, F. Kornelisse^6^, Salwan Al‐Nasiry^7^, Reint Jellema^8^, H. Marieke M. Knol^9^, Gwendolyn T. R. Manten^9^, Susanne M. Mulder‐deTollenaer^9^, Jan B. Derks^1^, Floris Groenendaal^1^, Mireille N. Bekker^10^, Ewoud Schuit^11^, Titia Lely^1^, Judith Kooiman^1^

^1^UMC Utrecht, UMC Utrecht, Utrecht; ^2^Amsterdam University Medical Centers, Amsterdam, Groningen; ^3^University Medical Center Groningen, Groningen, Groningen; ^4^Universitair Medisch Centrum Groningen, University Medical Center Groningen, Groningen; ^5^Amsterdam UMC, Amsterdam UMC, Noord‐Holland; ^6^Erasmus MC, Erasmus MC, Zuid‐Holland; ^7^Maastricht UMC+, Limburg; ^8^Maastricht UMC, Maastricht UMC, Limburg; ^9^Isala, Isala, Overijssel; ^10^University Medical Center Utrecht, Utrecht; ^11^University Hospital Centre Utrecht, University Hospital Centre Utrecht, Utrecht

17:15–18:30

**Objective**: Early‐onset fetal growth restriction (FGR) frequently requires iatrogenic, preterm birth to prevent stillbirth or severe neurological damage. Timing of delivery is complex in early‐onset FGR and the time sequence of changes in fetal and maternal parameters is not uniformly. Nevertheless, for clinicians it is of value to be informed of these changes to optimize clinical care. This study therefore aimed to study the changes in fetal and maternal parameters in a large, retrospective cohort of early‐onset FGR pregnancies.

**Study Design**: A multicenter, retrospective cohort study was performed in six level III hospitals between 2012‐2021 including pregnancies complicated by early‐onset FGR (consensus‐based definition Gordijn et al.). Repeated measures on fetal and maternal parameters were assessed routinely from diagnosis to delivery. Mixed‐effects models were used to determine the probability of abnormality over time for each parameter, stratified according to gestational age at delivery ( < and >32 weeks).

**Results**: A total of 1,453 patients were included, of whom 1,025 delivered before 32 weeks and 428 after 32 weeks. As can be appreciated from Figure 1, the pulsatility index (PI) of the umbilical artery (UA) was first to become abnormal, followed by the PI of the middle cerebral artery. However, the last days preceding delivery the end‐diastolic velocity in the UA had an increased probability to become absent/reversed. With regard to maternal parameters, an increased use of antihypertensive agent(s) was seen the last days preceding delivery in the group that delivered before 32 weeks.

**Conclusion**: This large cohort of recent early‐onset FGR pregnancies provides a time sequence of fetal and maternal parameters, compliant with previous results (Hecher, 2001). This knowledge could aid clinical practice in timely transfer of patients to hospitals with a neonatal intensive care facility for fetal monitoring and antenatal corticosteroid administration. Future analyses should elaborate the predictive value of each parameter for adverse perinatal outcomes.

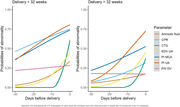

## 226 | OPtimal TIming of antenatal COrticosteroid in early‐onset fetal growth REstriction: The OPTICORE study

Mette van de Meent^1^, Wessel Ganzevoort^2^, Sanne J. Gordijn^3^, Elizabeth M. W. Kooi^4^, Wes Onland^5^, Johannes J. J. Duvekot^6^, René, F. Kornelisse^6^, Salwan Al‐Nasiry^7^, Reint Jellema^8^, H. Marieke M. Knol^9^, Gwendolyn T. R. Manten^9^, Susanne M. Mulder‐deTollenaer^9^, Jan B. Derks^1^, Floris Groenendaal^1^, Mireille N. Bekker^10^, Ewoud Schuit^11^, Titia Lely^1^, Judith Kooiman^1^

^1^UMC Utrecht, UMC Utrecht, Utrecht; ^2^Amsterdam University Medical Centers, Amsterdam, Groningen; ^3^University Medical Center Groningen, Groningen, Groningen; ^4^Universitair Medisch Centrum Groningen, University Medical Center Groningen, Groningen; ^5^Amsterdam UMC, Amsterdam UMC, Noord‐Holland; ^6^Erasmus MC, Erasmus MC, Zuid‐Holland; ^7^Maastricht UMC+, Limburg; ^8^Maastricht UMC, Maastricht UMC, Limburg; ^9^Isala, Isala, Overijssel; ^10^University Medical Center Utrecht, Utrecht; ^11^University Hospital Centre Utrecht, University Hospital Centre Utrecht, Utrecht

17:15–18:30

**Objective**: Early‐onset fetal growth restriction (FGR) often requires preterm delivery. Antenatal corticosteroids (CCS) reduce neonatal morbidity and mortality, most effectively when administered 2‐7 days preceding delivery. Timing of antenatal CCS is complex in early‐onset FGR, as the exact onset and course of fetal hypoxia are unpredictable. This study aimed to compare two main timing strategies of antenatal CCS in the Netherlands: administration when (A) the umbilical artery shows a pulsatility index above the 95th centile versus (B) absent or reversed end‐diastolic velocity.

**Study Design**: A multicenter, retrospective cohort study was performed including singleton pregnancies complicated by early‐onset FGR (consensus‐based definition Gordijn et al.) in six level III hospitals with either strategy A or B in the Netherlands between 2012 and 2021 (Figure 1). Primary outcome was a composite of perinatal and in‐hospital mortality of the infant. Mixed‐effects model analysis was performed to compare timing strategies, adjusted for birthweight Z‐score and gestational age at birth.

**Results**: A total of 1,453 pregnancies were included. Median values of birthweight and gestational age at delivery were 1050 grams (IQR 800‐1345) and 30.6 weeks (IQR 28.6‐32.4), respectively, and did not differ between the strategies. Time intervals between CCS and delivery were 6 (strategy A; IQR 3‐18) and 5 (strategy B; IQR 3‐16). Only 39.6% (A) and 38.1% (B) of patients delivered within the CCS therapeutic window of 2‐7 days. The composite primary outcome of perinatal and in‐hospital mortality occurred in 7.2% (A) and 9.8% (B), adjusted OR strategy B vs A 1.47; 95% CI 0.97‐2.22.

**Conclusion**: Rates of perinatal and in‐hospital mortality did not differ significantly between the timing strategies. Importantly, timing of antenatal CCS was suboptimal in the majority of patients. Future studies should focus on explaining differences in neonatal outcomes and ultimately the development of a dynamic prediction model for the need of medical delivery as an alternative timing strategy of antenatal CCS.

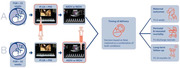

## 227 | School performance after induction of labor versus expectant management for late PROM: PPROMEXIL follow‐up

Larissa I. van der Windt^1^, Martijn A. Oudijk^1^, Anita C. Ravelli^2^, Noor E. Simons^2^

^1^Amsterdam UMC, location University of Amsterdam, Amsterdam, Noord‐Holland; ^2^Amsterdam UMC, Amsterdam, Noord‐Holland

17:15–18:30

**Objective**: Late preterm prelabor rupture of membranes (PPROM) from 34‐37 weeks of gestation can be managed by expectant management (EM) or induction of labor (IoL), balancing the risks of infection and preterm birth. In the randomized PPROMEXIL trials interventions were compared and showed no benefit for IoL. In its ten year follow‐up study, including 35% of all children, outcomes were comparable. However, children with adverse outcomes seem less likely to partake in follow‐up research potentially leading to self‐selection bias. The current study aims to assess educational outcomes at 12 years of age of children born after pregnancies complicated by late PPROM managed by EM compared to IOL.

**Study Design**: This follow‐up study included singletons born between 2006 and 2009 to participants of the PPROMEXIL trial. Data retrieved in the trial were linked with the national perinatal registry and national data on standardized school achievement testing (rating 501–550) and positive school advise for senior general or pre‐university secondary education for around the age of 12. Children exposed to IOL versus EM will be compared.

**Results**: Outcomes were attainable for 105 children. Mean education score was 533.6 in the EM group (61 children) and 533.9 in the IoL (44 children) (p 0.89). Also positive school advise did not significantly differ. When outcomes of children were compared based on participation in the 10 year follow‐up study, education score was 533.1 in non‐participants (533.3 EM vs 532.9 IOL) and 534.7 in the participating group (534.2 EM vs 535.3 IOL) (p 0.48).

**Conclusion**: Children born after IOL as management for late PPROM have similar education scores as those in the EM group, which is in line with the previous follow‐up study. Participants of the long‐term follow‐up study have slightly higher educational scores. It is therefore of added value to link trial data to national registries for long‐term outcomes. However, outcomes have to be interpreted with caution since outcomes were attainable for only limited amount of children.

## 228 | Increased postpartum severe maternal morbidity associated with stillbirth compared to full‐term livebirth deliveries

Dante F. Varotsis^1^, Jordan Beacham^2^, Julie Gomez^3^, Megan B. Raymond^4^, Rupsa C. Boelig^5^, Vincenzo Berghella^6^, Moti Gulersen^7^

^1^Thomas Jefferson University, Thomas Jefferson University/Philadelphia, PA; ^2^Sidney Kimmel Medical College at Thomas Jefferson University, Philadelphia, PA; ^3^Thomas Jefferson University, Philadelphia, PA; ^4^Thomas Jefferson University, Philadelphia, Pennsylvania; ^5^Sidney Kimmel Medical College, Thomas Jefferson University, Philadelphia, Pennsylvania; ^6^Sidney Kimmel Medical College of Thomas Jefferson University, Philadelphia, Pennsylvania; ^7^Division of Maternal‐Fetal Medicine, Thomas Jefferson University, Philadelphia, Philadelphia, Pennsylvania

17:15–18:30

**Objective**: Rates of severe maternal morbidity (SMM) in the United States continue to increase. Data exploring the association between stillbirth and postpartum SMM are limited and without detailed pregnancy characteristics. Therefore, we aimed to evaluate the association between postpartum SMM and stillbirth compared to full‐term livebirths.

**Study Design**: Multicenter retrospective cohort study within our hospital system from 2017‐2023. All stillbirths, defined as delivery of a fetus showing no signs of life ≥ 20 weeks gestation, were eligible for inclusion. The control group included livebirths between 39 0/7–41 6/7 weeks that were matched at an attempted 1:2 ratio controlling for hypertensive disorders and diabetes. Multiple gestations, maternal age < 18 years old, duplicate records, and elective terminations were excluded. The primary outcome of composite SMM, derived from the Centers for Disease Control and Prevention list of indicators, was compared between the two groups. Multivariable logistic regression was performed to adjust for potential confounders. Data were presented as adjusted Odds Ratios (aOR) with 95% confidence intervals (CI). Statistical significance was set at *p* < 0.05.

**Results**: 29,060 deliveries occurred during the study period, of which 129 (0.44%) stillbirths were included for analysis. Rates of alloimmunization (2.3% vs. 0%, *p* = 0.0007), placental abruption (2.3% vs. 0%, *p* = 0.0007), and substance use (5.4% vs. 1.0%, *p* = 0.0004) were significantly higher in the stillbirth group compared to full‐term. The odds of composite SMM were significantly higher in stillbirths compared to full‐term livebirths (17.6% vs. 5.8%, aOR 3.39, 95% CI 1.72‐6.66) (Table). Postpartum hemorrhage, blood transfusion, uterine rupture, ICU admission, preeclampsia, and sepsis were significantly increased in the stillbirths compared to full‐term livebirths (Table).

**Conclusion**: Stillbirth is associated with an increased risk of SMM compared to full‐term livebirths. Increased precautions should be taken regarding hemorrhage management, uterine rupture, sepsis, and postpartum preeclampsia for stillbirth deliveries.

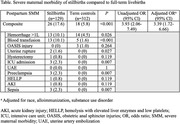

## 229 | Increased postpartum severe maternal morbidity associated with stillbirth compared to gestational‐age matched livebirth deliveries

Dante F. Varotsis^1^, Jordan Beacham^2^, Julie Gomez^3^, Megan B. Raymond^4^, Rupsa C. Boelig^5^, Vincenzo Berghella^6^, Moti Gulersen^7^

^1^Thomas Jefferson University, Thomas Jefferson University/Philadelphia, PA; ^2^Sidney Kimmel Medical College at Thomas Jefferson University, Philadelphia, PA; ^3^Thomas Jefferson University, Philadelphia, PA; ^4^Thomas Jefferson University, Philadelphia, Pennsylvania; ^5^Sidney Kimmel Medical College, Thomas Jefferson University, Philadelphia, Pennsylvania; ^6^Sidney Kimmel Medical College of Thomas Jefferson University, Philadelphia, Pennsylvania; ^7^Division of Maternal‐Fetal Medicine, Thomas Jefferson University, Philadelphia, Philadelphia, Pennsylvania

17:15–18:30

**Objective**: Reducing the rate of severe maternal morbidity (SMM), which has been substantially higher than in other high‐income countries, is a priority in the United States. There is limited data exploring the association of SMM with stillbirths. Furthermore, many of these studies only compare to term livebirths. Therefore, we aimed to evaluate the association of postpartum SMM and stillbirth compared to gestational age (GA) matched livebirths.

**Study Design**: Multicenter retrospective cohort study within our hospital system from 2017‐2023. All stillbirths, defined as delivery of a fetus showing no signs of life ≥ 20 weeks gestation, were eligible for inclusion. GA‐matched livebirths were matched in an attempted 1:1 ratio based on the presence of hypertensive disorders and diabetes. Multiple gestations, maternal age < 18 years old, duplicate records, and elective terminations were excluded. The primary outcome of composite SMM, derived from the Centers for Disease Control and Prevention list of indicators, was compared between the two groups. Multivariable logistic regression was performed to adjust for potential confounders. Data were presented as adjusted Odds Ratios (aOR) with 95% confidence intervals (CI). Statistical significance was set at *p* < 0.05.

**Results**: Of the 29,060 between 2017‐2023, 129 (0.44%) stillbirths were included. There were significantly fewer cesarean deliveries in stillbirths compared to GA‐matched livebirths (13.2% vs. 29.2%, *p* = 0.001). The odds of composite SMM were significantly higher in stillbirth deliveries compared to GA‐matched livebirths (19.4% vs. 8.0%, aOR = 4.35, 95%, CI 1.75‐10.84) (Table). Significantly more blood transfusions were needed in the stillbirth group (10.1% vs. 2.9%, *p* = 0.017) (Table). There were no significant differences in the other SMM components (Table).

**Conclusion**: Stillbirth is associated with an increased risk of SMM, specifically blood transfusions, compared to GA‐matched livebirth deliveries. Increased precautions should be taken with this patient population, including a low threshold for prophylactic and active management of postpartum hemorrhage.

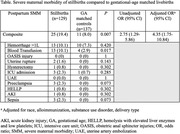

## 230 | Maternal body mass index and cumulative oxytocin dose in woman with induced labor

Nathalie Vilhelmsson^1^, Anna Ramö, Isgren^2^, Marie Blomberg^3^, Sara Carlhäll^3^

^1^Dept of Obstetrics and Gynecology and Dept of Biomedical and Clinical Sciences, Linköping University, Linköping, Sweden, Linköping University, Ostergotlands Lan; ^2^Linköping University, Linköping University, Ostergotlands Lan; ^3^Dept of Obstetrics and Gynecology and Dept of Biomedical and Clinical Sciences, Linköping University, Linköping, Sweden, Linköping University, Linköping, Ostergotlands Lan

17:15–18:30

**Objective**: Obese women have higher risk for failed induction of labor (IOL). Guidelines for oxytocin dosage are not adjusted for body mass index (BMI). We evaluated if the cumulative oxytocin dose and the maximum oxytocin infusion rate during the first stage of labor in women with IOL are associated with BMI.

**Study Design**: A Swedish retrospective observational multicenter study, from 2017 to 2019, including 1394 nulliparous women with IOL and a term singleton pregnancy. The cumulative oxytocin dose was measured from the start of oxytocin infusion until birth in 892 women. Information on maximum oxytocin infusion rate in the first stage of labor was collected by electronical medical record review and available for 1144 women. The study population was categorized according to BMI: normal weight (BMI 18.5‐24.9, *n* = 660), overweight (BMI 25.0‐29.9, *n* = 419), obesity class I (BMI 30.0‐34.9, *n* = 193) and morbid obesity (BMI ≥ 35.0, *n* = 122). Outcomes were compared between the BMI groups. Kaplan Meier and Cox regression analyses were performed to measure the association between BMI and the cumulative oxytocin dose and the maximum oxytocin infusion rate respectively. Adjustments were made for maternal age.

**Results**: The mean cumulative oxytocin dose differed between the BMI groups (normal weight 4233 mU, overweight 5436 mU, obesity class I 6587 mU, morbid obesity 8238 mU) (p ≤ 0.001). The mean maximum oxytocin infusion rate differed between the BMI groups (normal weight 14.1 mU/min, overweight 16.1 mU/min, obesity class I 18.4 mU/min, morbid obesity 23.2 mU/min) (p ≤ 0.001). The chance for birth at a certain total oxytocin dose decreased significantly with increasing BMI group compared to normal weight women (overweight adjusted Hazard Ratio (aHR) = 0.83, obesity class I aHR = 0.72 and morbidly obese aHR 0.62).

**Conclusion**: Obese women with IOL received higher cumulative oxytocin dose and maximum oxytocin infusion rate compared to normal weight women. Future guidelines for IOL with oxytocin infusion may need to be individualized and oxytocin dosage adjusted according to maternal BMI.

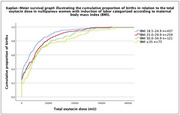

## 231 | Impact of immediate cerclage placement on pregnancy outcomes in cases of extremely short cervix

Elizabeth Vitaro^1^, Kristine E. Brown^2^, Shai Bejerano^3^, Uma M. Reddy^3^, Mirella Mourad^3^, Maria Andrikopoulou^4^

^1^Columbia University Medical Center, New York, NY; ^2^New York Presbyterian/Columbia, New York, NY; ^3^Columbia University Medical Center, New York, New York; ^4^Columbia University Irving Medical Center, New York, New York

17:15–18:30

**Objective**: Management of an extremely short cervix ( < 10mm) in pregnancy remains a clinical challenge, with cerclage placement being a potential intervention. The benefit of cerclage placement in such cases remains controversial.

**Study Design**: A retrospective cohort study conducted at a single tertiary care center from 2015 to 2023 included patients with a cervical length < 10mm. Patients with multiple gestations, history of spontaneous preterm birth, or who underwent a D&E were excluded. Patients were categorized as receiving immediate cerclage (within 72 hours of a short cervix diagnosis) or expectant management with vaginal progesterone. The primary outcome was gestational age (GA) at delivery. Secondary outcomes included latency from cerclage placement to delivery and pregnancy outcomes. Binary and categorical variables were analyzed with Fisher's exact test or chi‐square tests. Continuous variables were analyzed with either Welch's t‐test or Wilcoxon rank‐sum tests. A robust linear regression model with standard errors, adjusted for GA and cervical dilation at diagnosis, was conducted for the primary outcome.

**Results**: A total of 92 patients were included with 77 patients in the immediate placement group and 15 in the expectant group. Of these patients, 4 had a cerclage placed later in pregnancy and 11 received expectant management. The median GA at delivery was 36.0 weeks in the immediate group and 36.1 in the expectant group. This difference was not statistically significant (*p* = 0.90), even after adjusting for potentially confounding variables (β = 2.72, *p* = 0.27). There was a trend of longer latency period from diagnosis to delivery in the immediate group, 15.0 weeks, compared to the expectant group, 11.0 weeks; though, not statistically significant (*p* = 0.06). There was also no difference seen in the other secondary outcomes.

**Conclusion**: In low‐risk patients diagnosed with extremely short cervix, there was no difference in GA at delivery between patients treated with immediate cerclage versus expectant management. These results suggest the role of cerclage placement in such cases requires further exploration.

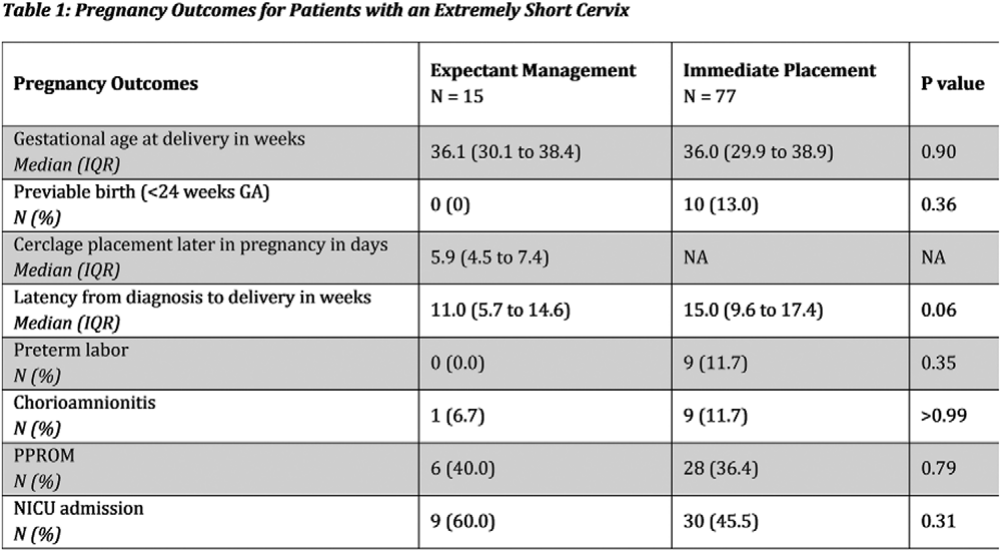

## 232 | Weight and risk adjusted peri‐operative bundle for prevention of cesarean section associated surgical site infections

Heather A. Walker^1^, Alexa C. Bowie^2^, Sara C. Scremin Souza^2^, Darine El‐Chaâr^3^, Megan Gomes^4^

^1^Ottawa Hospital Research Institute, Ottawa, ON; ^2^Ottawa Hospital Research Institute, Ottawa, Ontario; ^3^The Ottawa Hospital, Ottawa, ON; ^4^The Ottawa Hospital, Ottawa, Ontario

17:15–18:30

**Objective**: Surgical site infections (SSI) following Cesarean Section (CS), especially in patients with high body mass index (BMI), significantly increase maternal morbidity risks. While individual interventions to reduce SSI risk after CS have been identified, their collective impact when bundled together has been less studied. This quality improvement initiative aimed to evaluate the effectiveness of the Weight and Risk Adjusted Peri‐operative (WRAP) bundle, a comprehensive care protocol designed to reduce the incidence of CS‐associated SSI by integrating multiple evidence‐based practices such as intra‐operative thermoregulation, antibiotic use and negative pressure wound dressings.

**Study Design**: This initiative was conducted at The Ottawa Hospital using a before‐after quasi‐experimental design over a two‐year period. It included all patients undergoing CS, focusing on those with a BMI ≥ 40 kg/m^2^ or with a BMI ≥ 35 kg/m^2^ and additional SSI risk factors. The control group was comprised of patients who underwent CS before the WRAP bundle implementation, while the intervention group included those who received peri‐operative care tailored to their risk category post‐implementation. Post‐CS phone calls by clinical reviewers were made 30‐42 days postpartum to identify SSI cases.

**Results**: Data from 4424 patients have been analyzed. In the high‐BMI group, the SSI rate decreased from 15.9% to 8.5% (*p*‐value 0.029). Across all BMI categories, the overall SSI rate dropped from 10.3% to 6.2% (*p*‐value < 0.001). We also identified that the accurate SSI rate reported by patients via post‐CS phone calls is higher than the SSI rate outlined only through medical record review. A gap in systematic surveillance of post‐operative outcomes was identified for CS in the Canadian context.

**Conclusion**: Tailored bundled interventions, such as the WRAP bundle, are effective in reducing CS‐associated SSI, particularly in high‐risk individuals. Continuous monitoring and reporting of post‐CS SSI are vital for quality improvement efforts to optimize postpartum outcomes.

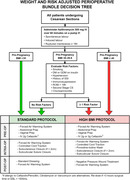

## 233 | Polycystic ovary syndrome and ferroptosis: A systematic review and meta‐analysis

Tianjing Wang^1^, Leyi Fu^2^, Ningning Xie^2^, Feng Yun^2^, Jinlan Piao^2^, jing lin^2^, Jue Zhou^2^, Danqing Chen^2^, Fan Qu^2^, Fangfang wang^2^

^1^WOMEN'S HOSPITAL SCHOOL OF MEDICINE ZHEJIANG UNIVERSITY, Hangzhou; ^2^WOMEN'S HOSPITAL SCHOOL OF MEDICINE ZHEJIANG UNIVERSITY, Hangzhou, Zhejiang

17:15–18:30

**Objective**: Ferroptosis, tightly regulated by iron metabolism, may be linked to polycystic ovary syndrome(PCOS). This review aimed to comprehensively explore the potential relationship between ferroptosis and PCOS, by investigating the differences in iron metabolism among women with PCOS in comparison to control, and narrating the past researches on PCOS and ferroptosis association.

**Study Design**: A systematic search of the literature was performed using PubMed, Embase, Web of Science from inception up to December 2022. Search terms relating to assisted PCOS, ferroptosis and iron metabolism were used. RevMan 5.4 was utilized for conducting the meta‐analysis, wherein the investigated outcomes included iron ferritin, iron, transferrin saturation and hepcidin. Narrative synthesis was performed to explore the correlation between PCOS and ferroptosis.

**Results**: In the meta‐analysis comprising a total of 16 studies, significant differences in serum ferritin levels (15 studies, standardized mean difference (SMD): 0.41, 95% CI: 0.22 to 0.59, *p* < 0.01) and transferrin saturation levels (3 studies, mean difference (MD): 4.39, 95% CI: 1.67 to 7.11, *p* < 0.01) between PCOS and control group were observed. Regarding serum iron (6 studies, SMD: 0.05, 95% CI: −0.24 to 0.33, *p* = 0.75) and serum hepcidin (4 studies, SMD: −0.44, 95% CI: −1.41 to 0.52, *p* = 0.37), no significant difference observed between PCOS and control group. Other studies have found that ferroptosis is involved in the occurrence and development of PCOS, offering valuable insights for guiding potential treatment measures and prognosis evaluation of PCOS. In addition, ferroptosis is involved in the miscarriage of PCOS‐like rat, suggesting that interventions targeting ferroptosis may hold promise for improving pregnancy outcomes in PCOS.

**Conclusion**: The observation of our analysis may suggest an underlying disturbance in iron metabolism in PCOS, potentially induce the activation of ferroptosis. Further research is imperative to elucidate the underlying pathophysiology, providing insights for potential preventive measures and therapeutic strategies.

## 234 | The effect of glycosylated hemoglobin variation index on PPROM in GDM women

Dan Wang^1^

^1^Chongqing Southeast Hospital, Chongqing, Chongqing

17:15–18:30

**Objective**: To explore the effect of glycated hemoglobin variation index (HGI) on preterm premature rupture of membranes (PPROM) in pregnant women with diabetes mellitus (GDM).

**Study Design**: Collect case data of 160 GDM patients from Chongqing Southeast Hospital from January 2022 to July 2023 for research. On the day of diagnosis, the fasting blood glucose and glycated hemoglobin values of all patients were counted, and the predicted glycated hemoglobin values were calculated using a linear regression equation to obtain the HGI. Using the median HGI (‐0.257) as the critical value, 160 GDM patients were divided into two groups: a high‐level group (glycated hemoglobin variation index ≥‐0.257, 80 cases) and a low‐level group (glycated hemoglobin variation index < −0.257, 80cases). Analyze the occurrence of PPROM in all patients, and investigate the impact of HGI on PPROM occurrence in GDM patients through point binary correlation analysis and logistic regression analysis.

**Results**: The HGI in 160 patients with GDM was (‐5.24)∼5.98; There is a statistical difference between two groups of glycated hemoglobin and predicted glycated hemoglobin (*p* < 0.05); Among 160 GDM patients, 25 (15.63%) had PPROM. The proportion of women aged ≥ 30 and with a history of alcohol consumption who experience GDM is higher than that of women without GDM (*p* < 0.05). The proportion of women with high HGI was significantly higher than that of the group without PPROM (*p* < 0.05). Logistic regression analysis suggests that the glycated hemoglobin variability index can affect the occurrence of PPROM in GDM patients (OR = 1.624, 95% CI 1.116‐2.291, *p* = 0.004).

**Conclusion**: The HGI is related to the occurrence of PPROM in GDM patients. The higher the HGI, the greater the likelihood of PPROM occurrence in GDM patients.

## 235 | Rates of delivery complications leading to postpartum hemorrhage by BMI and mode of delivery

Nastassia Anna. Yammine^1^, Claire D. Sundjaja^2^, Cece Cheng^3^, Michaela Y. Lee^1^, Natasha Paul^1^, Margaret T. Klausmeyer^1^, Bryce T. Munter^4^, Barbara Kate. Neuhoff^5^, Patrick S. Ramsey^4^, John J. Byrne^4^

^1^UTHSCSA, San Antonio, Texas; ^2^University of Texas Health San Antonio, San Antonio, Texas; ^3^UTHSCSA, San Antonio, TX, Texas; ^4^University of Texas Health Science Center San Antonio, San Antonio, Texas; ^5^UT Health Science Center San Antonio, San Antonio, Texas

17:15–18:30

**Objective**: Body mass index (BMI) is associated with an increased risk of delivery complications. We sought to evaluate if there was an association between BMI and delivery complications that leads to postpartum hemorrhage (PPH).

**Study Design**: We performed a single‐institution, retrospective cohort study on patients whose deliveries were complicated by PPH from 8/2020‐11/2021. Inclusion criteria included quantitative blood loss (QBL) of 1000 ml within 24 hours of delivery. Patients were divided into four cohorts based on BMI–non‐obese ( < 30 kg/m^2^), Class 1 obese (30‐34.9 kg/m^2^), Class 2 obese (35‐39.9 kg/m^2^) and Class 3 obese (40 kg/m^2^). Comparisons were made based on the mode of delivery–vaginal (VD) versus cesarean (CD). The primary outcome was the composite rate of delivery complications that led to PPH including uterine atony, retained products of conception, manual removal of the placenta, uterine inversion, vaginal lacerations for patients undergoing VDs, and hysterotomy extensions for patients undergoing CDs.

**Results**: Of the 4298 patients who delivered during the study period, 534 (12%) experienced a PPH. Of these patients, 218 (41%) delivered vaginally and 316 (59%) delivered by CD. Patients with a non‐obese BMI were significantly more likely to self‐identify as Asian, while patients with Class 3 obesity were more likely to be delivered by CD (*p* = 0.02) and have higher rates of chronic hypertension (*p* = 0.004) and pregestational diabetes (*p* < 0.0001). All other demographics were similar between BMI classes. Patients with Class 3 obesity who delivered vaginally were more likely to experience uterine atony but less likely to have vaginal lacerations. There were no differences in delivery complication rates in patients who delivered via CD; however, the hysterotomy extension trend was higher in the Class 3 obesity cohort but did not reach significance (*p* = 0.05, Table).

**Conclusion**: Class 3 obesity may be a risk factor for uterine atony during VD and hysterotomy extension during CD. Further investigation is needed to properly identify and counsel patients with higher BMIs on the risks associated with delivery.

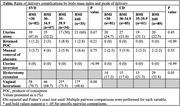

## 236 | Characteristics of COVID‐19 vaccine hesitancy among pregnant women in the Southern US

Lanbo Yang^1^, Miguel Bonilla Moreno^2^, Maya Roth^3^, Eva Kruger^3^, Heather Barnes^3^, Alexandra Kruse^3^, Marissa Steinberg^3^, Chi Dola^4^

^1^Tulane University School of Medicine, Tulane University School of Medicine, Louisiana; ^2^Tulane University School of Medicine Department of OBGYN, New Orleans, LA; ^3^Tulane University School of Medicine, New Orleans, Louisiana; ^4^Tulane University School of Medicine Department of OBGYN, New Orleans, Louisiana

17:15–18:30

**Objective**: To evaluate factors contributing to vaccine hesitancy and uptake and understand risk perceptions of influenza, Tdap (tetanus‐diphtheria‐pertussis) and COVID‐19 vaccination among pregnant and postpartum women in a metropolitan city in the Deep South region of the US.

**Study Design**: Retrospective cohort study between April and July 2021 at outpatient clinics affiliated with an academic medical center who received COVID‐19 vaccine counseling during regular antenatal visits.

**Results**: Among a racially diverse cohort of 262 women (44% Black, 29% Hispanic, 22% white and 5% Asian/other races), the mean age was 28 ± 6 years old. The mean gestational age at which vaccine counseling occurred was 24.7 ± 9.91 weeks. 29% of the women were obese (BMI≥30). 93% of the women had never been diagnosed with COVID‐19 prior to pregnancy and 3.2% were infected with COVID‐19 while pregnant. Prior to counseling, 21.2% had already received the COVID‐19 vaccine and 31% did not plan on receiving the vaccine. Perceptions of influenza, Tdap and COVID‐19 vaccinations are shown in Table 1. Sources of COVID‐19 vaccine information included: TV, radio and the news (37.8%), friends and family (30.5%), social media (21.0%), doctor's office (17.2%) and CDC or government (6.1%); 16.0% used multiple sources. After counseling about the COVID‐19 vaccine, 12.9% planned to receive the vaccine during pregnancy, 16.3% considered receiving the vaccine postpartum and 10.2% would consider receiving the vaccine in the future; 33.3% of the women declined. No differences in vaccine attitudes were observed between racial and ethnic groups.

**Conclusion**: Our study, the first to study COVID‐19 vaccine attitudes in pregnancy in the Deep South region of the US, demonstrates that pregnant women decline COVID‐19 vaccine at high rates despite adequate counseling on safety and benefits by a healthcare provider. Unreliable and inconsistent sources of information along with regional attitudes may contribute to poor vaccine literacy. Further efforts are necessary to increase COVID‐19 vaccine uptake and prevent maternal morbidity and mortality.

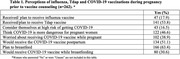

## 238 | Assessing the impact of anesthetic techniques during cesarean delivery on autism spectrum disorder risk

Omri Zamstein^1^, Tamar Wainstock^2^, Eyal Sheiner^2^

^1^Soroka University Medical Center, Soroka University Medical Center, HaDarom; ^2^Department of Obstetrics and Gynecology, Soroka University Medical Center, Ben‐Gurion University of the Negev, Beer‐Sheva, Israel., Beer Sheva, HaDarom

17:15–18:30

**Objective**: Research indicates that certain prenatal and perinatal interventions might influence the risk of autism spectrum disorder (ASD) development. Considering the potential impact of anesthesia exposure in early life on a child's neurological development, our study focuses on assessing whether the anesthetic techniques applied during cesarean delivery (CD) may affect the likelihood of ASD occurrence.

**Study Design**: A population‐based cohort study, including singleton births, was carried out at a tertiary referral hospital to examine how the type of anesthesia used for CD—neuraxial anesthesia (NA) versus general anesthesia (GA)— might relate to the later diagnosis of ASD in offspring, including data from both hospital and community settings. Kaplan‐Meier survival curve was utilized to analyze the cumulative morbidity, and a Cox proportional hazards model was employed to adjust for confounders.

**Results**: The study encompassed 17,481 CDs, with 3,854 (22%) utilizing NA and the rest GA. Compared to those given NA, patients undergoing GA for their CD were generally younger (30.1 years vs. 31.4 years), had a higher likelihood of preterm delivery (17.4% before 37 weeks of gestation vs. 9.7%), and had newborns with lower birth weights (15.9% under 2,500 grams vs. 10.0%; *p* < 0.001 for all). Out of the cohort, 172 children were diagnosed with ASD during the follow‐up, with slightly higher rates in the NA group than in the GA group (1.3% vs. 0.9%, *p* = 0.016). However, the cumulative incidence of ASD did not differ significantly between the two groups (log‐rank *p*‐value = 0.532; Figure). After adjusting for variables such as diabetes mellitus, hypertension, birth week, maternal age, gender, ethnicity, and birth year in a Cox proportional hazards model, no association was documented between the anesthetic method and ASD risk (aHR = 1.02, 95% CI 0.72–1.44, *p* = 0.92).

**Conclusion**: The type of anesthesia used during cesarean delivery, neuraxial or general, has no significant impact on the risk of autism spectrum disorder development.

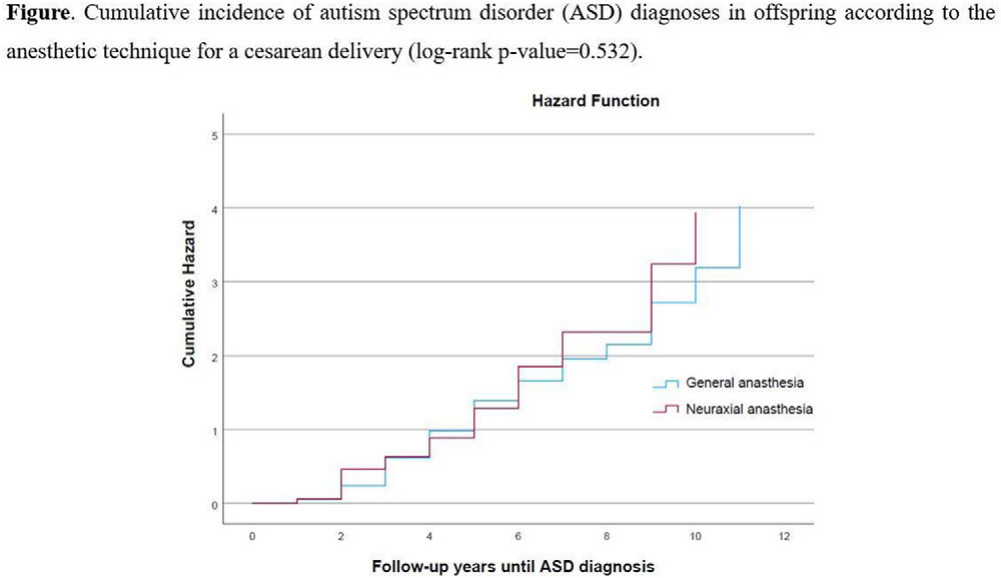

## 239 | Examining the influence of cesarean delivery on autism spectrum disorder risk

Omri Zamstein^1^, Tamar Wainstock^2^, Eyal Sheiner^2^

^1^Soroka University Medical Center, Soroka University Medical Center, HaDarom; ^2^Department of Obstetrics and Gynecology, Soroka University Medical Center, Ben‐Gurion University of the Negev, Beer‐Sheva, Israel., Beer Sheva, HaDarom

17:15–18:30

**Objective**: Evidence indicates that various perinatal conditions could elevate the risk of autism spectrum disorder (ASD). With cesarean delivery (CD) accounting for a substantial proportion of births, understanding its potential impact on ASD is imperative. This study seeks to investigate the relationship between CD and ASD development within the context of known factors that affect the onset of ASD.

**Study Design**: A population‐based cohort study was conducted at a tertiary referral center, focusing on singleton births. The study aimed to compare the occurrence of ASD in children, considering both hospital and community‐based diagnoses, in relation to the mode of delivery–vaginal delivery (VD), elective CD, or emergency CD. A Kaplan‐Meier survival curve was employed to assess the cumulative incidence of ASD. A Cox proportional hazards model was used to account for confounding variables.

**Results**: Among 115,081 included births, 17,481 (15.1%) were CD, with the remainder being VD. Emergency CDs accounted for 32.3% of all CDs, with the rest being elective. Conditions associated with high‐risk pregnancies, such as diabetes mellitus, hypertensive disorders, and preterm births, were notably more common in the CD group (*p* < 0.001 for all; Table). During follow‐up, 767 children were diagnosed with ASD: 0.6% from VD, 1.0% from emergency CDs, and 1.0% from elective CDs (*p* < 0.001). However, no significant difference was observed in the cumulative ASD diagnosis rates across these groups (log‐rank *p*‐value = 0.896). Additionally, after adjusting for various factors including pregnancy comorbidities, birth week, maternal age, child's gender, ethnicity, and birth year in Cox proportional hazards models, no significant association was found between the method of delivery or the urgency of CDs and ASD risk (aHR for delivery mode = 1.07, 95% CI 0.89–1.28, *p* = 0.48; aHR for CS urgency = 95%0.95, CI 0.68–1.34, *p* = 0.78; Table).

**Conclusion**: Cesarean delivery, whether elective or emergency, does not significantly affect the likelihood of autism spectrum disorder development.

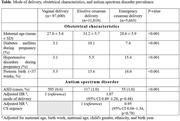

## 240 | Early diabetes screening in pregnancy: Differentiating between pregestational diabetes and early gestational diabetes

Alexandra Zetye^1^, Ashli Gibb^2^, Malek Ghandour^3^, Bernard Gonik^3^

^1^Wayne State University, Detroit Medical Center, Ferndale, MI; ^2^Detroit Medical Center/Wayne State University, Detroit, Michigan; ^3^Wayne State University, Detroit, Michigan

17:15–18:30

**Objective**: Controversy exists regarding testing for pregestational diabetes (DM) during pregnancy. Since the majority of data suggests that early screening before 24w0d for gestational diabetes (GDM) does not improve outcomes, ACOG advises that patients at risk for DM should be screened using ADA nonpregnant criteria when evaluating for DM early in pregnancy. As a part of the needs assessment component of a quality improvement project at our institution, we examined patients screened early in pregnancy for assessment of compliance with these recommendations.

**Study Design**: Patients were identified to be at risk for DM based on ICD‐10 codes. Included patients received prenatal care between September 2021‐December 2023 at our institution and had screening via a Hemoglobin A1c (HbA1c), 2‐hour 75g oral glucose tolerance test (2h GTT), or 1‐hour 50g oral glucose challenge test (early 1h). Correct diagnosis of DM was determined by HbA1c ≥ 6.5%, fasting blood glucose > 125 mg/dL, or 2h GTT ≥ 200 mg/dL. A blood glucose ≥ 200 mg/dL at 2 hours on 3h 100g GTT was considered diagnostic for DM, despite higher glucose load. The primary outcome was the screening test ordered before 24w0d. The secondary outcome was accuracy of diagnosis from positive early testing.

**Results**: Of 957 patients identified, 366 patients met inclusion criteria. Screening was completed with only HbA1c in 200 (54.6%), only early 1h in 93 (25.4%), and both in 73 (19.9%). No patients were screened with 2h GTT. Overall, 11 patients (3.0%) met the criteria for DM. Of these, 6 (54.5%) were correctly diagnosed and 5 (45.5%) were incorrectly diagnosed with GDM. Criteria for GDM were met by 6 patients (1.6%). Of these, 4 (66.7%) were correctly diagnosed and 2 (33.3%) were incorrectly diagnosed with DM. There was no difference in the rate of incorrect diagnoses between any of the groups.

**Conclusion**: These results demonstrated a lack of compliance with current ACOG guidelines for the diagnosis of pre‐gestational DM early in pregnancy and support the planned QI project which will be based on sharing of these data and educational modules to improve compliance with guidelines.

## 241 | Impact of aspirin dose on development of pre‐eclampsia in twin pregnancy: A multicentre retrospective study

PIERPAOLO ZORZATO^1^, Dominique A. Badr^2^, Eleonora Torcia^3^, Andrew Carlin^2^, Alessandra Familiari^4^, Erich Cosmi^5^, Silvia Visentin^6^, Elisa Bevilacqua^7^, Jacques C. Jani^2^

^1^Azienda Ospedale Università Di Padova, Padova, Veneto; ^2^University Hospital Brugmann, Université Libre de Bruxelles, Brussels, Belgium, Bruxelles, Brussels Hoofdstedelijk Gewest; ^3^Fondazione Policlinico Universitario Agostino Gemelli IRCCS, Rome, Italy, ROMA, Lazio; ^4^University of the Sacred Heart Department of Women and Child Health, Fondazione Policlinico Universitario Agostino Gemelli IRCCS, Università Cattolica del Sacro Cuore, Roma, Lazio; ^5^Università degli studi di Padova, Università degli studi di Padova/Padova, Veneto; ^6^Università Degli Studi di Padova, Padova, Veneto; ^7^Department of Women, Children, and Public Health Sciences, IRCCS Agostino Gemelli University Polyclinic Foundation, Catholic University of the Sacred Heart, Rome, Lazio

17:15–18:30

**Objective**: This study aimed to assess the efficacy of various aspirin prophylaxis dosages in the prevention of preeclampsia and hypertensive disorders of pregnancy (HDP) in twin pregnancies.

**Study Design**: This was an international multicentre retrospective cohort study that was conducted in three European centers. Patients included were categorized into three groups: no aspirin, daily 80‐100mg aspirin, and daily 160mg aspirin. Primary outcomes were the incidence of preeclampsia and HDP, whereas secondary outcomes were small‐for‐gestational age, postpartum hemorrhage >1000 mL, antenatal bleeding of obstetrical origin, thrombocytopenia, miscarriage, intrauterine fetal demise (IUFD), neonatal death, and gastritis. Propensity score matching and multivariate analyses were conducted to assess outcomes in order to balance the three groups of study. Cox regression models were done for each outcome after matching to compare the three groups. A *p*‐value < 0.05 was considered statistically significant.

**Results**: A total of 1907 twin pregnancies were included. After using propensity score matching, the three groups were adequately balanced (absolute standardized difference [ASD] < 15%), except for age and thrombophilia (ASD 22.1% and 16.4% respectively). Aspirin 80‐100mg was insufficient to decrease the rate of HDP (HR: 1.72, 95%CI: 1.06‐2.77, *p* = 0.027) and preeclampsia (HR: 1.92, 95%CI: 0.98‐3.77, *p* = 0.058), while aspirin 160mg significantly reduced HDP (HR: 0.56, 95%CI: 0.34‐0.93, *p* = 0.025) but not preeclampsia (HR: 0.63, 95%CI: 0.34‐1.14, *p* = 0.125). No significant increase for aspirin‐related complications, such as bleeding or thrombocytopenia, or other obstetrical outcomes was observed with the high dose of aspirin.

**Conclusion**: The use of 160 mg aspirin for the prevention of hypertensive disorders of pregnancy, encompassing preeclampsia, may offer superior outcomes in twin pregnancies, with no discernible rise in complications when compared to aspirin doses ranging from 80‐100 mg. Further research should explore long‐term impacts and refine dosage strategies for optimal outcomes in twin pregnancies.

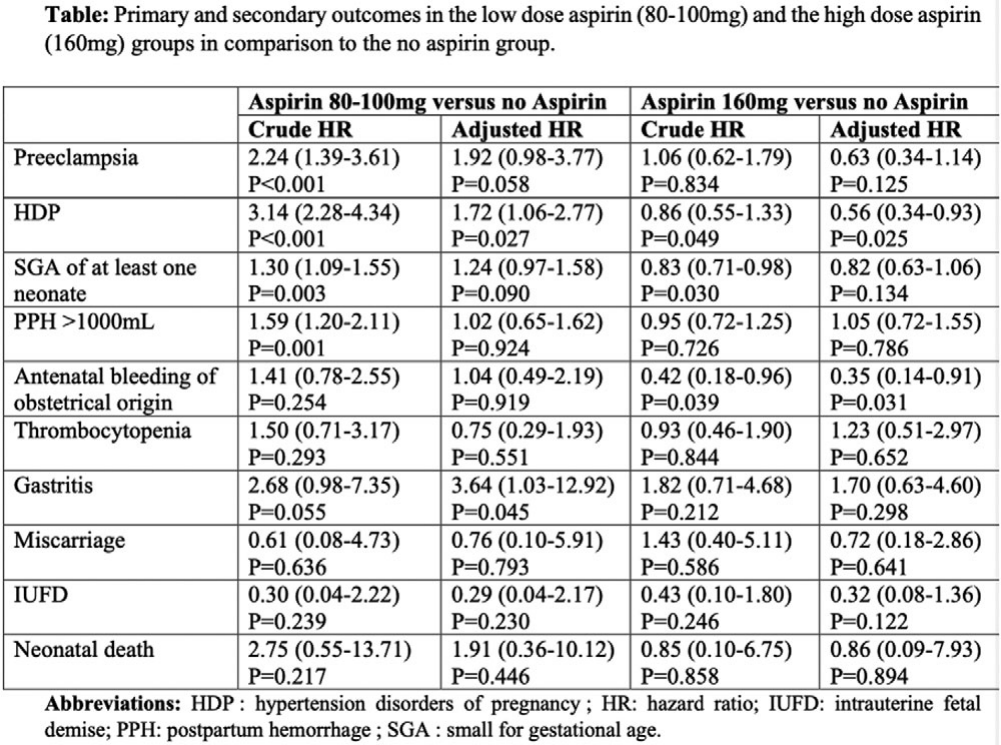

## POSTER SESSION 3

Friday, 27 September 2024 11:45–13:00

## 242 | Quantitative Blood Loss: The correlation with hemoglobin drop and need for transfusion

Fareeza Afzal^1^, Samantha Perovski^2^, Muhammad Aslam^2^, Simran Sherwal^2^, Sadie Ellenson^2^

^1^Ascension St. John, Troy, MI; ^2^Ascension St. John, Detroit, Michigan

11:45–13:00

**Objective**: Postpartum hemorrhage (PPH) is defined as a blood loss of 1,000 mL or more, and is the leading cause of death that occurs on the day of birth in the United States. In an attempt to accurately determine blood loss, institutions are adopting the use of quantitative blood loss (QBL). We sought to determine if QBL was accurate in determining blood loss by comparing QBL to the change in postpartum hemoglobin from pre‐delivery hemoglobin (delta hemoglobin), the need for blood or iron transfusion, and changes in pre‐delivery and postpartum vital signs.

**Study Design**: This was a historical cohort study that was done by a retrospective chart review of women (ages 18 years and older) who had a vaginal or Cesarean delivery between January 1, 2022 to December 31, 2022. QBL, hemoglobin, vital signs (heart rate and blood pressure), and the need for an iron or blood transfusion were collected from the inpatient medical record. Paired analysis, repeated measures analysis of variance and multiple pairwise comparisons using the Bonferroni correction of the *p*‐value were used for analysis.

**Results**: 346 patients were analyzed during the study period. A negative correlation was found between QBL and change in pre‐delivery to post‐partum hemoglobin (r = −0.37, *p* < 0.001). The patients that required transfusion had a higher median QBL of 1000 mL (753.0, 1316.0) compared to those who did not with a median of 274.0 mL (125.0, 509.0, *p* < 0.001). Only the delta diastolic blood pressure for one hour (r = −0.14, *p* = 0.01) and the delta heart rate at 24 hour post‐delivery (r = 0.31, *p* < 0.001) was significant.

**Conclusion**: Based on the results of this study, preemptive transfusion of iron or blood products could be considered in patients with a QBL of 1000 mL or greater. Utilization of preemptive transfusion would not only reduce delays in care for acute blood loss anemia, but it could also reduce instances of postpartum symptomatic anemia.

## 243 | Toward the identification and prediction of maternal biopsychosocial risk

Temitope O. Ajileye^1^, Sandra Nwokeoha^2^, Sangeeta Agnihotri^3^

^1^Ellescope Ltd, oxford, England; ^2^Ellescope Ltd, London, England; ^3^Barts Health NHS Trust, London, England

11:45–13:00

**Objective**: The ‘Saving Babies Lives’ 2023 Report [1] highlighted that the UK has worse maternal mortality metrics than other major European countries, with 40% of maternal deaths owed to multiple mental or social disadvantages [2]. Pre‐birth risk factors collected during GP visits are often not readily accessible to hospital clinicians. Our study aims to evaluate the impact of pre‐existing biopsychosocial (BPS) risk factors on maternal outcomes and assess the viability of querying EPR data. The objective was to develop a clinical tool that identifies and triages maternal risks, equipping clinical teams with the insights they require to appropriately tailor the antenatal care of a patient.

**Study Design**: A retrospective cohort study assessed 116413 pregnancy records across 2018‐2023 obtained from an NHS Integrated Care Board. The records included patients’ demographics, 60 categories of BPS factors and 20 categories of adverse outcomes. Records spanned primary and secondary care and were clustered using supervised classification models. We analysed the correlations between risk factors and adverse outcomes alongside a control group. We further enriched the data by linking the deprivation indexes of the patient's GP practices in our dataset with the 2019 IMD.

**Results**: Patients with a history of BPS risk factors were twice as likely (22%) to develop adverse outcomes than patients with no historical risk factors. Mental health (MH) related risk factors correlated strongly with the onset of adverse outcomes: Mental Disorder (40%), Insomnia (25%), Anxiety and Depression (24%). A disproportionate distribution of adverse outcomes across ethnic groups was found; patients identifying as “mixed” being twice as likely as other patients to experience adverse MH related outcomes. A similar trend was detected along the IMD scale: MH outcomes impacted deprived areas disproportionately.

**Conclusion**: The analytical tool we have developed provides essential support toward the personalisation of maternal care. Early evidence of its actionable use was demonstrated across a group of UK GP practices (published case study available).

## 245 | Timing of oxytocin initiation during the second stage of labor among multiparous patients

Gal Bachar^1^, Shaked Greenhut^2^, Yoav Siegler^3^, Yaniv Zipori^1^, Nizar khatib^4^, Zeev Weiner^2^, Dana Vitner^1^

^1^Rambam Medical Center, Haifa, Hefa; ^2^Rambam Medical Health Center, Haifa, Hefa; ^3^Rambam Health Care Center, Rambam Health Care Center, HaZafon; ^4^Rambam Medical Health Campus, Rambam Medical Health Campus, Hefa

11:45–13:00

**Objective**: To investigate the optimal timing for oxytocin administration during the second stage of labor in multiparous women, aiming to optimize vaginal delivery rates and achieve favorable maternal and neonatal outcomes.

**Study Design**: This retrospective cohort study analyzed data from multiparous women with singleton pregnancies who received oxytocin solely during the second stage of labor (2014‐2021), at a single tertiary medical center. Women were categorized based on the timing of oxytocin initiation: within 1 hour, 1‐2 hours, or greater than 2 hours after complete cervical dilation. The primary outcome measure was the cesarean delivery (CD) rate. Secondary outcomes encompassed other adverse maternal and neonatal outcomes.

**Results**: A total of 250 pregnancies were included. Early oxytocin administration (within 1 hour) was observed in 17.6% (*n* = 44), followed by initiation between 1‐2 hours (66.8%, *n* = 167), and after 2 hours (15.6%, *n* = 39). Compared to the late initiation group (>2 hours), women receiving oxytocin within 2 hours exhibited a significantly lower CD rate (3‐5% vs. 18%, *p* < 0.05). This association remained statistically significant after adjusting for confounding variables. No statistically significant differences were identified in other maternal or neonatal complications between groups.

**Conclusion**: Initiating oxytocin early during the second stage of labor in multiparous women might be associated with a reduced CD rate and a shorter second stage duration, without compromising maternal or neonatal well‐being.

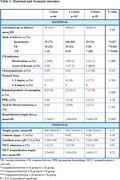

## 246 | Timing of oxytocin initiation in the second stage of labor among primiparous women

Gal Bachar^1^, Shaked Greenhut^2^, Naama Farago^3^, Naphtali Justman^1^, Yaniv Zipori^1^, Nizar khatib^4^, Zeev Weiner^2^, Dana Vitner^1^

^1^Rambam Medical Center, Haifa, Hefa; ^2^Rambam Medical Health Center, Haifa, Hefa; ^3^Rambam Health Care Center, Rambam Health Care Center, HaZafon; ^4^Rambam Medical Health Campus, Rambam Medical Health Campus, Hefa

11:45–13:00

**Objective**: To evaluate the association between timing of oxytocin initiation in the second stage of labor and vaginal delivery rates, alongside maternal and neonatal outcomes in nulliparous women.

**Study Design**: This retrospective cohort study analyzed data from nulliparous women with singleton pregnancies undergoing a trial of vaginal delivery between 2014 to 2021, at a single tertiary medical center. Women received oxytocin only after reaching the second stage. We compared outcomes for women receiving oxytocin within 1 hour, 1‐2 hours, or >2 hours of full cervical dilation. The primary outcome was cesarean delivery (CD) rate.

**Results**: A total of 2054 pregnancies were included. Oxytocin initiation occurred within 1 hour, 1‐2 hours, or >2 hours after full dilation in 181 (8.81%), 1210 (58.91%), and 663 (32.28%) of women, respectively. Baseline and delivery characteristics were similar across groups. The CD rate was significantly lower in women receiving oxytocin within 2 hours of full dilation compared to >2 hours (8.68% vs. 11.61% vs. 31.23%, *p* < 0.05). Early oxytocin administration ( < 1 hour) showed a lower rate of CD due to arrest of descent (57% vs. 79‐86%, *p* < 0.05). Additionally, early administration was associated with a lower rate of third/fourth‐degree perineal tears (1.6‐1.8% vs. 3%, *p* < 0.05) compared to a >2‐hour delay. These associations remained significant after adjusting for confounders in multivariable regression analysis. No significant differences in other adverse maternal or neonatal outcomes were observed between groups.

**Conclusion**: Initiating oxytocin early during the second stage of labor is associated with a lower CD rate, particularly those due to arrest of descent, and a lower rate of advance perineal lacerations, without compromising neonatal well‐being.

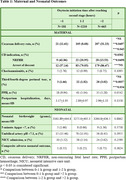

## 247 | Neutrophil‐to‐lymphocyte ratio as a predictor for peritoneal adhesion formation after cesarean delivery

Shani Binyamin^1^, Adi Y Y. Weintraub^2^, Reut Rotem^3^, Alla Saban^4^, Zehava Yohay^5^, Tamar Eshkoli^2^

^1^shaare Zedek Medical center ISRAEL, shaare Zedek medical center israel, Yerushalayim; ^2^Soroka University Medical Center, Soroka University Medical Center, HaDarom; ^3^Shaare Zedek medical center, Sharre Zedek Medical center, Yerushalayim; ^4^Soroka University Medical Center, Beer Sheva, HaDarom; ^5^Pain Unit, Soroka University Medical Center, Beer Sheva, Israel, Beer Sheva, HaDarom

11:45–13:00

**Objective**: Postoperative adhesions are a common complication after cesarean deliveries (CD), particularly after repeated surgeries. So far, only a few studies have been published to support the use of hematological indices (HI) in the prediction of adhesions formation. We aimed to investigate the role of HI in the prediction of adhesions.

**Study Design**: A retrospective case‐control study was conducted comparing HI in the first CD between women with and without adhesions diagnosed at the second CD, attributable to the first CD. Matching was done for CD indication, and strict exclusion criteria were applied to avoid including cases with adhesions attributed to other factors. The initial analysis was performed using descriptive statistics and advanced analytical statistics. Additionally, an analysis of the area under the receiver operating curves (ROC) was constructed for selected indices.

**Results**: Overall, 176 women were found eligible. Of those, 88 women who developed adhesions at the second CD were matched with 88 controls. Non‐progressive labor during the first CD (31% vs. 13.6%, *p* < 0.01) and gestational diabetes mellitus (GDM) during the pregnancy of the first CD (10.2% vs. 2.3% *p* = 0.03) were more prevalent among women with adhesions. Mean white blood cell counts as well as the neutrophil‐to‐lymphocyte ratio (NLR) taken pre‐operatively and during the postpartum period (PPP) were significantly higher in the study group. In a multivariable analysis controlling for maternal age, maternal smoking, and GDM during the first CD, NLR during the PPP was found to be significantly associated with adhesion formation during the second CD (aOR = 1.15, CI 1.09‐1.25, *p* < 0.01).

**Conclusion**: Our study suggests that NLR taken postoperatively was associated with the development of intra‐peritoneal adhesions in a subsequent CD. A complete blood count is routinely taken before and after CD; thus, NLR may be an effective, inexpensive, and reliable predictive tool for intra‐peritoneal adhesions in a subsequent CD.

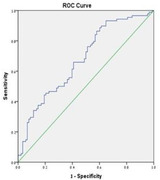

## 248 | The association between preconception diet and severe maternal morbidity

Courtney M. Bisson^1^, Isabella Sciacca^2^, Sunitha Suresh^3^

^1^University of Chicago/Endeavor, University of Chicago, IL; ^2^HCA Medical City Healthcare UNT‐TCU, Dallas, Texas; ^3^Endeavor Health, Evanston, Illinois

11:45–13:00

**Objective**: Severe maternal morbidity (SMM) is increasing in the United States, with a large component attributable to blood transfusions. Prior data has suggested that deficiency in various micronutrients or overall dietary quality may be associated with adverse pregnancy outcomes. The primary objective of this study was to assess dietary quality measured by the Alternative Healthy Eating Index 2010 (AHEI2010) with transfusion‐related SMM and non‐transfusion‐related SMM.

**Study Design: Methods**: This was a retrospective secondary analysis of the nuMom2B dataset that included 6,540 patients with complete AHEI2010 dietary data. The primary outcomes were a composite of transfusion‐related SMM and non‐transfusion‐related SMM. Univariate analysis was performed using chi squared and ANOVA for categorical and continuous variables respectively. Multivariable logistic and linear regression was used to assess the independent association between SMM and dietary quality, adjusting for education, BMI, race, income, diabetes, and chronic hypertension.

**Results**: Majority of the patients were non‐Hispanic White (60.1%) and non‐Hispanic Black (14.8%) and between the ages of 18‐34 (95.0%). Unadjusted models demonstrated no association between non‐transfusion related SMM (odds ratio [OR] 0.996, 95% confidence interval [CI] 0.989–1.003; *p* = 0.248) and transfusion‐related SMM (OR 1.000, 95% CI 0.9832–1.02); *p* = 0.999) with dietary quality. Adjusted models showed no association between dietary quality and transfusion ‐related morbidity (adjusted OR 1.00 [95% CI (0.983–1.012)]; *p* = 0.999). The adjusted model for non‐transfusion related SMM also illustrated no association between dietary quality and non‐transfusion related SMM (adjusted OR 0.996 [95% CI (0.989–1.00)]; *p* = 0.22).

**Conclusion**: Our results illustrated no association between dietary quality and transfusion‐related SMM and non‐transfusion‐related SMM. The findings in this study suggest further work should be completed to better assess modifiable risk factors for SMM.

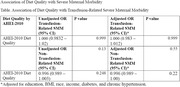

## 250 | Kleihauer Betke test and minor abdominal trauma in pregnancy: To do or not to do?

Olena Minich^1^, Sabina Razdolsky^2^, shanny kolp asis^3^, Elad Miron^4^, Adi Ashkenazi Katz^4^, Loren Elbaz^4^, Ariel Many^5^

^1^Tel Aviv University, Kfar Saba, HaMerkaz; ^2^Mayanei Hayeshua Medical Center, Mayanei Hayeshua Medical Center, Tel Aviv; ^3^Tel Aviv university, Tel aviv, HaMerkaz; ^4^Tel Aviv University, Tel Aviv University, Tel Aviv; ^5^Department of Obstetrics and Gynecology, Mayanei Hayeshua Medical Center, bnei brak, HaMerkaz

11:45–13:00

**Objective**: The Kleihauer‐Betke (KB) test, a vital diagnostic tool in obstetrics, serves to discern fetal‐maternal hemorrhage (FMH) following abdominal trauma during pregnancy. KB may be used in pregnancy after trauma to rule out any FMH and fetal anemia. Furthermore, in Rh‐negative pregnant women this test may be useful to assess the extent of fetal blood in maternal circulation. But is there any benefit for this test during minor trauma in pregnancy?

**Study Design**: This is a retrospective cohort study. conducted at a single university‐affiliated medical center with approximately 12,000 deliveries per year. The study group included all pregnancies >24wks gestation who were admitted due to minor abdominal trauma during a 2‐year period. Minor trauma was defined as any incident of fall or car accident with low impact and without direct abdominal trauma.

**Results**: Overall 297 pregnant women were included. The mean gestational week at admission was 32 wks. The mean gestational week at delivery was 37 wks. Four pregnancies (1.3%) were complicated with clinical placental abruption during labor. The average getstational weight was 3050 mg with 12 (4%) fetal growth restriction. There was no neonatal anemia required therapy. Table 1

**Conclusion**: According to our study there is no justification for the use of KB test during the management of minor abdominal trauma. Further research is warranted to validate these results.

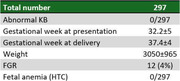

## 251 | Angiogenic biomarkers at mid pregnancy for prediction of termpreeclampsia in an antenatal care setting

Stina Björkman^1^, Marie Bladh^2^, Sara Bergstrand^3^, Sofia Sederholm Lawesson^4^, Elisabeth Aardal^5^, Martin Overgaard^6^, Caroline Lilliecreutz^7^

^1^Department of Obstetrics and Gynaecology in Norrköping, and Department of Biomedical and Clinical Sciences, Linköping University, Linköping, Sweden, Linköping, Ostergotlands Lan; ^2^Department of Obstetrics and Gynaecology, and Department of Biomedical and Clinical Sciences, Linköping University, Linköping, Sweden, Linköping, Ostergotlands Lan; ^3^Department of Health, Medicine and Caring Sciences, Linköping University, Linköping, Sweden, Linköping, Ostergotlands Lan; ^4^Department of Cardiology, and Department of Health, Medicine and Caring Sciences, Linköping University, Linköping, Sweden, Linköping, Ostergotlands Lan; ^5^Department of Clinical Chemistry, and Department of Biomedical and Clinical Sciences, Linköping University, Linköping, Sweden, Linköping, Ostergotlands Lan; ^6^Department of Clinical Biochemistry, Odense University Hospital, and Department of Clinical Research, University of Southern Denmark, Odense, Denmark, Odense, Syddanmark; ^7^Department of Obstetrics and Gynaecology and Department of Biomedical and Clinical Sciences, Linköping University, Linköping, Sweden, Linköping, Ostergotlands Lan

11:45–13:00

**Objective**: Improved risk stratification for preeclampsia (PE) is desirable to reduce adverse outcomes and to allocate resources. First trimester screening can identify women at risk of preterm PE ( < gestational week (GW) 37). This method is less effective for term PE and another approach is needed. The use of the angiogenic biomarkers; soluble Fms‐like tyrosine kinase receptor 1 (sFlt‐1), Placental Growth Factor (PlGF), and the sFlt‐1/PlGF‐ratio for mid‐pregnancy screening of term PE is not fully elucidated and different methods have been proposed. The purpose was to evaluate sFlt‐1, PlGF and the sFlt‐1/PlGF‐ratio in GW 24‐29 for prediction of term PE, in an antenatal care setting.

**Study Design**: This was a retrospective case‐control study using serum from a pregnancy research biobank taken at GW 24‐29. Women who later developed PE and twice as many normotensive pregnant controls, matched for parity and age, comprised the study population. sFlt‐1 and PlGF were analysed on BRAHMS Kryptor Gold (Thermo Scientific) and their concentrations were compared between women with term PE and controls. ROC‐curves and cut‐offs for prediction of term PE were computed.

**Results**: The mean time from sampling to PE diagnosis was 91.0 (SD ± 17.2) days. The sFlt‐1/PlGF‐ratio and PlGF levels were higher in women who later developed PE (*n* = 92) than controls (*n* = 374) (*p* < 0.001). ROC‐curves for the sFlt/PlGF‐ratio suggested a cut‐off at 3.92 (sensitivity 0.61, specificity 0.73) with an area under the curve (AUC) 0.70 (95% CI 0.63‐0.76). The corresponding numbers for PlGF were: cut‐off 224.85 pg/ml (sensitivity 0.56, specificity 0.84) with an AUC 0.76 (CI 95% 0.70‐0.82). sFlt‐1 alone did not add any value in identifying women that developed term PE (Figure 1).

**Conclusion**: Evaluating the sFlt‐1/PlGF‐ratio or PlGF alone at GW 24 to 29, in women participating in standard antenatal care, might help to identify women at risk of developing term PE. Although, the predictive performance of the biomarkers might not be sufficient to use alone. The most appropriate method for risk evaluation for term PE is yet to be defined.

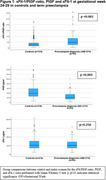

## 252 | Low PAPP‐A ( < 0.3MoM) at first trimester screening and structural abnormalities in euploid fetuses

Mariachiara Bosco^1^, Tiziana Fanelli^2^, Ilaria Fantasia^2^, Valentina De Robertis^2^, Paolo Volpe^2^

^1^Unit of Obstetrics and Gynecology, Department of Surgery, Dentistry, Pediatrics and Gynecology, University of Verona, AOUI Verona, University of Verona, Veneto; ^2^Fetal Medicine Unit, “Di Venere” Hospital, Bari, Puglia

11:45–13:00

**Objective**: To investigate the proportion of structural abnormalities in fetuses with a low PAPP‐A ( < 0.3MoM) and normal karyotype and array CGH.

**Study Design**: Retrospective cohort study including all singleton pregnancies with maternal plasma PAPP‐A < 0.3MoM at 1st trimester screening. An invasive procedure for fetal karyotype and array CGH was offered when indicated (high risk for aneuploidies or structural anomalies). Cases with an abnormal genetic test were excluded. All pregnancy were followed‐up with regular scans until 36 weeks’ gestation. Data on pregnancy outcomes were collected from our database.

**Results**: From 2011 to 2023, 581 singleton pregnancies with low PAPP‐A were identified. 39 cases were excluded because of incomplete pregnancy information or loss at follow‐up. 109 were confirmed to have abnormal genetic findings. 433 cases had negative genetic test results; intrauterine demise was observed in 2 cases, while 37 fetuses developed FGR. Of the total included (394) cases, 292 (66%) had a normal outcome with adequate fetal growth. US follow‐up showed at least one structural anomaly in 102 cases (34%), of these, 14 cases had multiple anomalies (3.2%). The encountered anomalies were: congenital heart diseases (CHD) in 40 cases (9%) [12 conotruncal anomalies and 5 minor CHD], of the central nervous system in 19 (4.3%), skeletal in 8 (1.8%), urogenital in 7 (1.5%), vascular in 7 (1.5%) [4 aberrant right subclavian artery, 3 ductus venosus agenesis], orofacial in 3 (2.9%), gastrointestinal in 3 (2.9%), congenital airway obstruction (CHAOS) in 1 (0.9%).

**Conclusion**: Among fetuses with low PAPP‐A and negative genetic test results, 34% had at least one structural anomaly found at US follow‐up. Patients who present with a low PAPP‐A in the first trimester not only benefit from serial US scans to assess fetal growth but might also represent a group of women who might benefit from a careful assessment for fetal structural anomalies, cardiac in particular. The implementation of exome sequencing analysis could provide insight into the association between low PAPP‐A in the 1st trimester and structural malformations.

## 254 | Diagnosis, management and outcomes of right aortic arch: From fetal life to long‐term follow‐up

Cosimo Marco Campanale^1^, Luciano Pasquini^2^, Marco Masci^1^, Adriano Carotti^3^, Duino Meucci^3^, Patrizio Moras^1^, Maria Carolina Colucci^1^, Alessandra Toscano^1^

^1^Bambino Gesù Pediatric Hospital, Roma, Lazio; ^2^Policlinico Agostino Gemelli, Policlinico Agostino Gemelli, Lazio; ^3^Ospedale Pediatrico Bambino Gesù, Ospedale Pediatrico Bambino Gesù, Lazio

11:45–13:00

**Objective**: The aims ot this study are (1) to report our experience with right aortic arch (RAA) and its variants, association with complete vascular ring (CVR), congenital heart disease (CHD), extracardiac malformations and (2) to describe the long‐term follow‐up in terms of symptoms and surgery for CVR.

**Study Design**: We collected clinical and instrumental data from 502 patients with RAA referred at our tertiary center.

**Results**: 157 patients (31.3%) had fetal diagnosis (medium follow‐up 7.6 years). 330 (65%) had CHD and 89 (17%) genetic anomalies, with DiGeorge syndrome the most frequent (9.5%). Genetic disorders were significantly more frequent in those with CHD (23% versus 7.5%, *p* < 0.001). 58 (11%) had extracardiac anomalies. Among the RAA not forming CVR, left anterior ductus arteriosus with mirror‐image (MI) branching pattern of epiaortic vessels (RAA/LADA/MI) was present in 29, 100% associated with CHD (conotruncal in 72%). Right ductus arteriosus (RAA/RDA/MI) was found in 8, 4 with complex CHD. CVR was present in 246 (49%): the most frequent were RAA, left posterior ductus arteriosus with aberrant left subclavian artery (RAA/LPDA/ALSA–163 cases, 66%) and double aortic arch (DAA–66 cases, 27%), Left posterior ductus arteriosus (LPDA) with MI branching (RAA/LPDA/MI–12 cases, 5%) and aberrant left innominate artery (RAA/LPDA/ALIA–5 cases, 2%) were rare. CVR were most frequently associated with minor CHD (26% versus 17% major CHD). At CT mean tracheal compression was 46% (57% for DAA, 43% for RAA/LPDA/MI. 109 (45%) were operated (median age 31.2 months). Three among 16 pts were re‐operated for persistent symptomatic compression, the majority being DAA.

**Conclusion**: RAA is commonly associated with CVR, asymptomatic in 2/3 of cases but requiring surgery in 50%. Symptoms resolves almost completely and reoperations are reserved to the DAA. RAA is present along with major CHD in half cases and these patients are at higher risk of genetic syndromes, however not frequent.

## 255 | Neurodevelopmental outcomes in infants with left heart obstructions and transposition of the great arteries

Cosimo Marco Campanale^1^, Silvia Bucci^2^, Marco Masci^1^, Patrizio Moras^1^, Maria Carolina Colucci^1^, Angela Rossi^2^, Chiara De Marchis^2^, Luciano Pasquini^3^, Alessandra Toscano^1^

^1^Bambino Gesù Pediatric Hospital, Roma, Lazio; ^2^Ospedale Pediatrico Bambino Gesù, Ospedale Pediatrico Bambino Gesù, Lazio; ^3^Policlinico Agostino Gemelli, Policlinico Agostino Gemelli, Lazio

11:45–13:00

**Objective**: Children with congenital heart disease (CHD) are at risk of neurodevelopmental disorders, decreased functional status and quality of life outcomes. Risk factors as severity of CHD, fetal oxygenated blood flow impairment to the brain, chromosomal anomalies and post‐natal little understood yet. Data about hypoplastic left heart syndrome (HLHS) and transposition of the great arteries (TGA) children have been already published but few data are available about children with non‐HLHS left heart obstructions such as aortic coarctation (CoAo) simple or associated to other lesions. We studied neurodevelopmental outcomes in CoAo and TGA patients compared with controls.

**Study Design**: Children with CoAo (simple, complex and shone syndrome) (*n* = 39), children with TGA (*n* = 54) and healthy controls (*n* = 24) aged 12 months who were admitted to Bambino Gesù Children's Hospital were included. To avoid bias in the interpretation of results, only children with isolated congenital heart disease without associated genetic syndromes were considered.

**Results**: Children with TGA presented significantly lower mean scores on the cognitive (M = 98.89 ± 7.99; *p* < .001), language (M = 95.92 ± 7.05; *p* < .001) and motor scales (M = 86.34 ± 10.26; *p* < .001) compare to children with CoAo (Cognitive M = 103.97 ± 7.96; language M = 100.18 ± 7.12; Motor M = 94.21 ± 6.91) and healthy controls (Cognitive M = 107.04 ± 7.34; language M = 105.18 ± 6.44; Motor M = 97.65 ± 8.93). However all group had mean scores within the normal range. Children with CoAo did not differ in their development from healthy children. Within the group of children with CoAo, no significant differences were found according to disease severity (simple CoAo; complex CoAo; Shone syndrome). Two children with TGA showed developmental delay (Cognitive < 85); 17 children with TGA and 3 children with CoAo showed delay in the area of motor skills.

**Conclusion**: Our results show that at 1 year infants with CoAo have neurodevelopmental scores comparable to controls while TGA patients are a significantly lower level, although within the normal ranges. However longer follow‐up is needed.

## 257 | Fetal tachycardia as a result to umbilical peripheral mechanic stimulation: Novelty from the EXTEND model

Maria Antonietta Castaldi^1^, Alan W. Flake^2^

^1^The Children's Hospital of Philadelphia, Philadelphia, PA; ^2^The Children's Hosspital of Philadelphia, The Children's Hosspital of Philadelphia, Pennsylvania

11:45–13:00

**Objective**: Intrapartum fetal monitoring is one of the most important challenges to prevent fetal hypoxia/acidemia. Although modern CTG interpretation of Fetal Heart Rate (FHR) is mainly established, it is common in clinical practice to find several circumstances in which a FHR pattern falls outside the usual frame of reference.

We hypothesize that some abnormal cardiotocographic patterns, activated by mechanical stimulus i.e. stretching a short cord, may be the trigger for remote preconditioning of trauma (RPCT) in the foetus and that tachycardia is the epiphenomenon. We tested this hypothesis on the EXTEND (Extra‐Uterine Environment for Neonatal Development) model.

Aim of the present study was to show that fetal tachycardia can be a result to umbilical peripheral mechanical stimulation: stretching the cord.

**Study Design**: To obtain a raise of at least 20bpm in FHR, we performed a controlled traction of the umbilical cord in 2 animals in both early‐pregnancy (day 99) and later‐pregnancy (day 112) conditions in the EXTEND model.

Each lamb was gently pushed to stretch the cord. FHR, Blood Pressure, Blood Flow, O2 delivery were recorded. A Related‐Samples Wilcoxon Signed Rank Test was performed. Statistical significance was set at *p* < .05.

**Results**: Experiments were repeated 15 times. A significant rise in FHR was present in all the experiments performed in later‐pregnancy lamb (day 112, equal to human 34 weeks). Median rise of FHR was 32,5bpm [21‐43 95%CI], p:.005. No difference in FHR was observed in the early‐pregnancy lamb (day 99, equal to 25 weeks) (**Figure 1**), neither in Blood Flow, Pressure and Oxygen delivery were observed.

**Conclusion**: When assessing intrapartum fetal monitoring, not only cardiovascular direct response to hypoxia/acidemia should be considered. IAs our study on the EXTEND model clearly shows, different physiological responses to mechanical stimuli and biomechanical interferences, such as umbilical cord stretching, should be pondered as possible factors able to modify FHR in very specific ways, such as triggering fetal tachycardia.

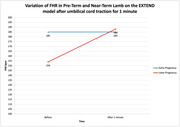

## 260 | Main cerebral fissures depth in fetuses affected by Congenital Heart Disease

Isabella Fabietti^1^, Milena Viggiano^1^, Maria Carolina Colucci^2^, Marco Masci^2^, Chiara Vassallo^3^, Cosimo Marco Campanale^2^, Elena Nicastri^4^, Patrizio Moras^2^, Leonardo Caforio^1^, Alessandra Toscano^2^

^1^Ospedale Pediatrico Bambino Gesù, ROMA, Lazio; ^2^Bambino Gesù Pediatric Hospital, Roma, Lazio; ^3^Ospedale Pediatrico Bambino Gesù, Ospedale Pediatrico Bambino Gesù, Lazio; ^4^Università degli Studi di Roma Tor Vergata, Università degli Studi di Roma Tor Vergata, Lazio

11:45–13:00

**Objective**: Reduced brain fissures have been already described in Left Heart Obstructive lesions (LHO) by two‐dimensional US. This study aimed to investigate brain fissures comparing different type of congenital cardiac anomalies.

**Study Design**: We retrospectively analyzed cerebral ultrasound data from pregnancies complicated by CHD, recording the main cerebral fissures at 26 and 30 weeks of gestational age. The collected data were indexed per biparietal diameter and compared according to pathophysiology into Right Heart Obstructive lesions (RHO), Left Heart Obstructive lesions (LHO), pathologies with Pulmonary Overflow Voume (OV), Fetal Heart failure (HF) and Transposition of Great Arteries (TGA).

**Results**: We recorded data of 28 (37.3%) fetuses affected from RHO, 1(1%) from LHO, 9 (12%) from OV, 10(13%) and 27(36%) from TGA. As displayed in the table, we found a reduced depth of insular lobe at 26 weeks of gestational age in fetuses with RHO being this result not confirmed at 30 weeks of gestational age. Otherwise, fetuses affected by OV showed a reduced depth of the parieto‐occipital fissure at both gestational age.

**Conclusion**: Reduced brain fissures were detected by two‐dimensional US in fetuses in RHO. Such informations would be helpful to evaluate the maturation progress improving counseling parents.

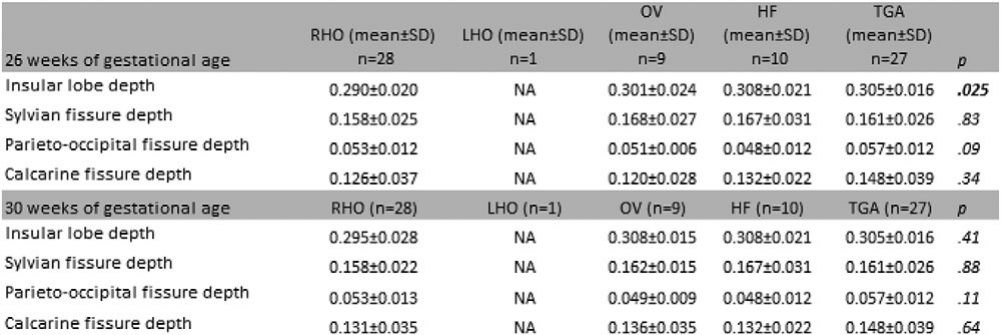

## 261 | Placental vascular malperfusion in fetuses with congenital heart diseases: Distinction based on pathophysiology

Alessandra Toscano^1^, Marco Masci^1^, Maria Carolina Colucci^1^, Alessandra Stracuzzi^2^, Rita Alaggio^2^, Cosimo Marco Campanale^1^, Milena Viggiano^3^, Patrizio Moras^1^, Isabella Fabietti^3^, Luciano Pasquini^4^

^1^Bambino Gesù Pediatric Hospital, Roma, Lazio; ^2^Ospedale Pediatrico Bambino Gesù, Ospedale Pediatrico Bambino Gesù, Lazio; ^3^Ospedale Pediatrico Bambino Gesù, Roma, Lazio; ^4^Policlinico Agostino Gemelli, Policlinico Agostino Gemelli, Lazio

11:45–13:00

**Objective**: Literature suggests a correlation between placental malperfusion (PM) and CHD, highlighting the importance of this relationship for early diagnosis, monitoring, and management of CHD. Therefore, our objective was to characterize placental anomalies in pregnancies complicated by CHD.

**Study Design**: From March 2021 to October 2023, 95 CHD‐affected fetal placentas were analyzed. A pathologist categorized placental pathology as maternal placental malperfusion (MPM), fetal placental malperfusion (FPM), and combined maternal‐fetal placental malperfusion (MFPM). Placentas were categorized according to fetal pathophysiology into Right Heart Obstructive lesions (RHO), Left Heart Obstructive lesions (LHO), pathologies with Pulmonary Overflow Voume (OV), Fetal Heart failure (HF) and Transposition of Great Arteries (TGA).

**Results**: In our population, newborns with the highest birth weight were affected by OV (in centiles, 66.4 ± 27.4, *p* = 0.004), with higher BW/PW in LHO and lower in TGA (RHO:0.2 ± 0.4;LHO:0.2 ± 0.4;OV: 0.4 ± 0.5;HF:0.2 ± 0.4;TGA: 0.1 ± 03.; *p* = 0.18), along with a higher prevalence of LGA placentas in the TGA group (54.3%, *p* = 0.07). PM was more prevalent in patients with OV and to a lesser extent in TGA (RHO:75.9%; LHO:72.7%; OV:87.5%; HF:75%; TGA:60%; *p* = 0.6). The graph illustrates the distribution of PM signs.

**Conclusion**: An association between placental vascular anomalies and fetal CHD is confirmed. The higher prevalence of PM in patients with OV may be biased by the high number of comorbidities. Meanwhile, a higher BW/PW in LHOs may be justified by a reduction in combined ventricular output in more severe forms.

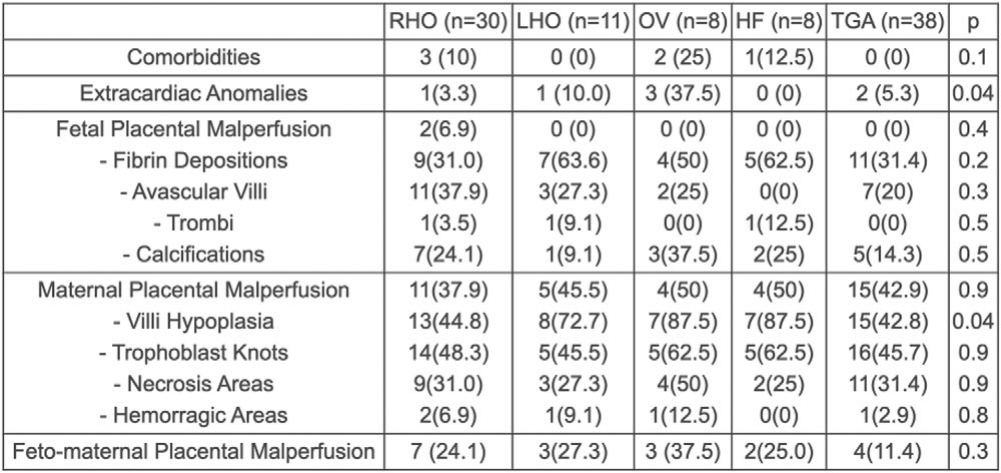

## 262 | Prenatal diagnosis of coarctation of the aorta: Ventricular disproportion in presence of ventricular septal defects

Maria Carolina Colucci^1^, Luciano Pasquini^2^, Marco Masci^1^, Cosimo Marco Campanale^1^, Patrizio Moras^1^, Alessandra Toscano^1^

^1^Bambino Gesù Pediatric Hospital, Roma, Lazio; ^2^Policlinico Agostino Gemelli, Policlinico Agostino Gemelli, Lazio

11:45–13:00

**Objective**: Prenatal diagnosis of coarctation of the aorta (CoAo) is challenging, often raised from intrauterine ventricular disproportion but only confirmed postnatally. We aimed to investigate if association with a ventricular septal defect (VSD) weakens predictive criteria.

**Study Design**: We retrospectively analyzed fetal echocardiographic data in pregnancies complicated by suspected CoAo from January 2019 to October 2023, indexing data according to validated z‐scores and dividing patients into VSD+ (undergoing corrective surgery in the first year of life) and VSD‐.

**Results**: We examined 129 fetuses suspected of CoAo, with 26.4% VSD+ and 73.6% VSD‐. Subaortic defects were present in 52.9%, and muscular defects in 20.6%. The table presents indexed valve measurements and ratios compared between the two subgroups, with notable differences in the size of the aortic isthmus. Neonates with VSD had a significantly lower false positive rate (VSD+:20.6%; VSD‐:66.3%, *p* = 0.01)

**Conclusion**: Our data suggest that prenatal diagnosis of VSD+ CoAo has significantly fewer false positives. Additionally, VSD is associated with greater isthmic hypoplasia, likely aiding prenatal diagnosis.

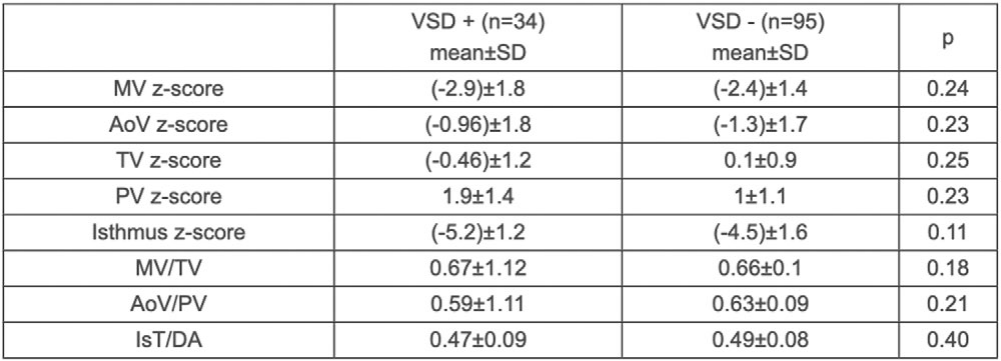

## 263 | Impact of Diabetes Mellitus on fetal cardiac morphology and functions

Anna Conte^1^, Fabiana Savoia^2^, Marco La Verde^3^, Maddalena Morlando^2^

^1^Università degli Studi della Campania Luigi Vanvitelli, arzano, Campania; ^2^Università degli Studi della Campania Luigi Vanvitelli, Università degli Studi della Campania Luigi Vanvitelli, Campania; ^3^Università degli Studi della Campania Luigi Vanvitelli, Univerità degli Studi della Campania Luigi Vanvitelli, Campania

11:45–13:00

**Objective**: Maternal diabetes impacts the fetal heart. Fetal echocardiography is used to detect cardiacs defects. The methods provide only morphological evaluation. Myocardial strain measured by two‐dimensional speckle tracking echocardiography has demonstrated greater sensitivity and specificity to assess cardiac fuction. The objective is to evaluate the differences in echocardiographic indices obtained from speckle‐tracking imaging in women suffering from diabetes and in physiological pregnancies.

**Study Design**: Prospective case‐control study.80 patients between in third trimester were recruited (53 control group and 27 case group). Exclusion criteria were: multiple pregnancies, no CRL dating, structural fetal anomalies, IUGR, maternal/obstetrical pathology, inadequate 4C cine‐loop. Fetal heart assessment was performed offline, after the acquisition of cine clips of the 4‐chamber view during third trimester ultrasound scan. The following indices were evaluated:Sphericity index (SI), Fractional area change (FAC), Global longitudinal strain (GLS), Inter‐ventricular septum thickness (IVST). These indices were compared in the case group and in the control group.

**Results**: There was no significant difference between the groups, except for BMI (30.6 ± 5.96 vs 27.0 ± 4.71, *p* = 0.021). We found a decrease in the FAC in group 1, as a result in a decrease in right ventricle contractility and a lower cardiac strain due to reduced cardiac deformation:FAC Z score RV: −3.7 ± 1.939 vs −2.357 ± 2.049, *p* = 0.008;FAC % RV: 20.744 ± 10.593 vs 27.115 ± 10.343, *p* = 0.012; GLS % RV: ‐11.0778 ± 7.166 vs 13.836 ± 7.089, *p* = 0.105. We found remodelling of the fetal heart in diabetic mothers in women in insulin treatment, from elliptical to spheroid:SI % LV: 1.98 ± 0.4237 vs 1.6412 ± 0.2451, *p* = 0.014;SI % RV: 18.67 ± 10.1578 vs 36.3941 ± 25.8933, *p* = 0.005.

**Conclusion**: The speckle tracking offers the potential to assess myocardial function. The small sample size is a significant limitation. Further evidences are needed to define the role of speckle‐tracking imaging to improve the management of pregnancies complicated by diabetes.

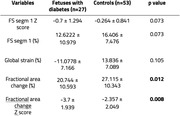

## 264 | Impact of maternal risk factors on periconception glycated hemoglobin and risk of major fetal anomalies

Shelby A. Crants^1^, Aleksandra Polic^2^, Lynsa Nguyen^3^, Etoi Garrison^2^, Amelie Pham^2^

^1^Mass General Brigham, Boston, MA; ^2^Vanderbilt University Medical Center, Nashville, Tennessee; ^3^Valley Children's Hospital, Madera, California

11:45–13:00

**Objective**: Elevated periconception glycated hemoglobin (A1c) in the setting of pre‐gestational diabetes mellitus (pre‐G DM) is a known risk factor for major fetal anomalies (MFA). Non‐Hispanic Black (NHB) race has also been associated with suboptimal periconception glycemic control. This study aims to further characterize the impact of these maternal risk factors on the association between periconception A1c and risk of MFA.

**Study Design**: This retrospective cohort study at a major academic referral center queried all singleton deliveries between 22‐44 weeks from 2010‐2021 with a periconception A1c (90 days before and up to 14 weeks after estimated conception). We excluded anomalous fetuses with known cause. Maternal and infant data were extracted. Primary outcome was any MFA, defined by the 2018 European surveillance of congenital anomalies criteria of pre‐specified ICD 9 and 10 codes. Poisson regression modeling adjusted for a priori selected covariates.

**Results**: Of 6,604 deliveries, 192 (3%) were complicated by pre‐G DM. A total of 726 infants (11%) had any MFA, of which 32% (235) were congenital heart defects. The baseline prevalence of MFA in mothers with A1C < 6.5% was 11% (681/6420) compared to 24% for those with A1C ≥ 6.5% (45/184). NHBs had higher mean A1c when compared to non‐Hispanic Whites (NHW) (5.4 ± 0.9 vs. 5.1 ± 0.6, *p* < 0.01). Increasing A1c was associated with a significantly higher risk of any MFA (adjusted incidence rate ratio (aIRR) 1.27, *p* < 0.01), regardless of pre‐G DM history (see Table). In pregnancies with A1c < 6.5%, NHB race appeared to be an independent risk for any MFA (aIRR 1.23, *p* = 0.03) compared to NHW. But in a small subgroup with A1C ≥ 6.5%, NHB race was no longer a significant factor (NHB vs NHW, *p* = 0.84).

**Conclusion**: Periconception A1c was independently associated with increased risk of MFA, regardless of other risk factors, in this high‐risk cohort. Universal periconception A1c screening rather than risk‐based screening should be used to identify fetuses at risk for MFA. Additional data on social determinants of health is needed to explore the impact of race on MFA risk.

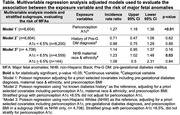

## 265 | Maternal bladder anomalies in pregnancy: Risk of genitourinary tract infectious complications and pregnancy outcomes

Shelby A. Crants^1^, Jennifer Johnson^2^, Nicole A. Smith^2^

^1^Mass General Brigham, Boston, MA; ^2^Brigham and Women's Hospital, Boston, Massachusetts

11:45–13:00

**Objective**: Bladder exstrophy (BE) and neurogenic bladder (NB) are conditions predisposing to urinary tract infections (UTIs) and complicating delivery planning. This population is at risk of multi‐drug resistant infection with antibiotic overuse. Limited data exist to counsel patients on risks and best practices in pregnancy. This study reviews genitourinary (GU) complications and outcomes for pregnant patients with BE and NB.

**Study Design**: This case series conducted at a major academic center queried all patients with a pregnancy from 2004‐2024 with a diagnosis of BE or NB seen by the perinatologist specializing in this population, with most co‐managed by infectious disease (ID). Patients were identified by ICD‐10 codes. Data was extracted by chart review. T‐tests were used for *p*‐value calculations.

**Results**: 15 pregnancies were reviewed from 9 patients, 3 with BE (33.3%) and 6 with NB (66.7%). 14 pregnancies (93.3%) resulted in live birth, with 12 (85.7%) at term. 5 deliveries (33.3%) were vaginal, 5 were cesarean due to bladder anomalies, and 5 were cesarean for other indications. 1 patient with NB had a pregnancy complicated by urosepsis and fetal demise prior to care in this center. Antibiotic suppression was prescribed in 6 pregnancies (42.9%), and patients on suppression had a mean of 1.2 hospitalizations for infectious complications (vs 0.13 in those not on suppression, *p* = 0.08). Number of UTIs did not differ by suppression status (3.2 vs 1.75, *p* = 0.26). Hospitalizations and number of UTIs also did not differ by anomaly (see Table). Number and timing of pregnancy urine cultures obtained varied widely but were most frequently obtained for symptoms, not surveillance.

**Conclusion**: BE and NB are rare conditions for which there are no clear strategies to guide obstetric management. Risk of complex UTI is increased in this population. Close surveillance resulted in live births in all affected pregnancies at this center. Weighing risks and benefits of suppression and avoidance of treating GU colonization to minimize antibiotic exposure requires a thoughtful multidisciplinary approach by MFM and ID specialists.

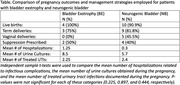

## 266 | Use of tranexamic acid after vaginal delivery with episiotomy: A double‐blind randomized placebo control trial

Atara De porto^1^, Elad Preuss^2^, alona doron^3^, Vadim Sheiman^4^, noa glick^4^, Josef Tovbin^3^, tal ben valid^4^, oshri barel^3^, eran barzilay^4^

^1^assuta ashdod, gan yavne, HaDarom; ^2^assuta ashdod, ashdod, HaDarom; ^3^assuta ashdod, assuta asdod, HaDarom; ^4^assuta ashdod, assuta ashdod, HaDarom

11:45–13:00

**Objective**: To evaluate the effect of prophilactic tranexamic acis on the decline in hempglobin levels folowing vaginal delivery with episiotomy

**Study Design**: a single center, double blind, randomized placebo ‐controlled trail was conducted at samson assuta ashdod hospital from july 2022 to augost 2023. 150 patiants aged 18‐45, undergoing vaginal delivery with episiotomy, were randomized into TA (*n* = 73) or placebo (*n* = 75)groups. TA or placebo was administrated post delivery and Hb levels were measured at 24‐72 hours post delivery.

**Results**: No significant differences were observed between the TA and placebo groups in terms of Hb level decrease (2.43 ± 1.36 vs. 2.35 ± 1.36 gr/dl, *p* = 0.723), estimated blood loss, incidence of post‐partum hemorrhage, or rates of post‐partum anemia and hematocrit. Additional outcomes including blood transfusion, post‐partum iron supplementation, and hospitalization duration were also similar between the groups.

**Conclusion**: Prophylactic administration of TA did not reduce blood loss or the risk of PPH following vaginal delivery with episiotomy. Therefore, its use as a prophylactic agent in low‐risk deliveries is not recommended.

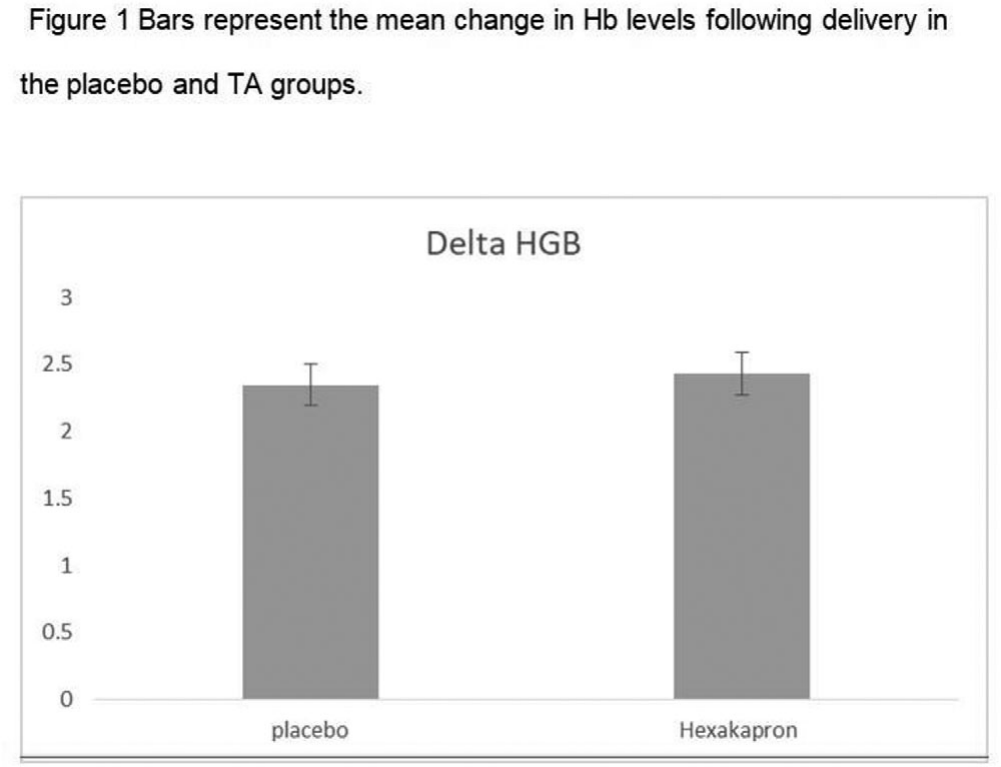

## 267 | Maternal and fetal outcomes in pregnant women with mechanical heart valves: Tertiary center case series

Camilla Dionisi^1^, Sara Doroldi^2^, Giuliana Simonazzi^1^, Gianluigi Pilu^1^, Elisa Montaguti^1^

^1^Obstetric Unit, IRCSS Azienda Ospedaliero Universitaria Di Bologna, Obstetric Unit, IRCSS Azienda Ospedaliero Universitaria di Bologna, Emilia‐Romagna; ^2^Obstetric Unit, IRCCS Azienda Ospedaliero‐Universitaria di Bologna, Bologna, Emilia‐Romagna

11:45–13:00

**Objective**: Patients with mechanical heart valves require lifelong anticoagulation to prevent thromboembolic complications. Pregnancy induces a hypercoagulable state, increasing the risk of thromboembolic events. Recent practice involves replacing vitamin K antagonists (VKA) with heparin (LMWH) to reduce fetal loss and anomalies, albeit with a potential rise in maternal complications.

This study aims to compare outcomes of pregnant patients with mechanical heart valves referred to our center with those reported in the literature.

**Study Design**: A retrospective observational study spanning fifteen years (2008‐2024) included eleven pregnant patients with mechanical heart valves. Primary outcomes encompassed maternal mortality and thromboembolic complications, while primary fetal outcomes involved live births and anticoagulant‐related fetal anomalies. Secondary outcomes comprised maternal bleeding, cardiac events (arrhythmias, heart failure, non‐thrombotic valvular dysfunction), postpartum hemorrhage, fetal intracranial bleeding, small for gestational age, miscarriage and fetal loss.

**Results**: Among our patients, three (27%) received VKA peri‐conceptionally, two (18%) received VKA throughout pregnancy, two (18%) received VKA until voluntary termination of pregnancy, three (27%) started LMWH preconceptionally. One patient had combinatory therapy: LMWH in first trimester and VKA after. Thromboembolic complications occurred in two patients receiving LMWH: one experienced valvular thrombosis and one non‐valvular thrombosis. Seven pregnancies resulted in live births, three in termination of pregnancy and one ended in miscarriage. One fetal subdural hematoma occurred in a patient on combined therapy at 26 weeks of pregnancy. No other fetal anomalies related to anticoagulants were reported.

**Conclusion**: Our findings align with existing literature, indicating a higher incidence of maternal complications in women with mechanical heart valves receiving LMWH. Limited data on patients treated with VKA during pregnancy preclude definitive conclusions regarding its safety.

## 268 | Retrospective study: Prevalence and timing of changes in antibody screening during pregnancy

Elchanan dreyfuss^1^, vered yahalom^2^, irit ambar^3^, Anat Pardo^4^, Anat Shmueli^5^, Eran Hadar^6^, Shiri Barabash Hazan^5^

^1^Helen Schneider Hospital for Women, Rabin Medical Center, Petach Tikva, ariel, HaMerkaz; ^2^Rabin Medical Center, Petach Tikva, Petach tikva, HaMerkaz; ^3^Helen Schneider Hospital for Women, Rabin Medical Center, Petach Tikva, Petach tikva, HaMerkaz; ^4^The Helen Schneider Hospital for Women at Rabin Medical Center, Petach Tikwa, HaMerkaz; ^5^Helen Schneider Hospital for Women, Rabin Medical Center, petach tikwa, HaMerkaz; ^6^Rabin Medical Center, Petach Tikva, HaMerkaz

11:45–13:00

**Objective**: During pregnancy, the coexistence of a fetus and mother can trigger red blood cells alloimmunization. For high‐risk pregnancies where surgery might be necessary, a “3‐day rule” mandates frequent blood type and antibody screening to ensure valid and up to date antibody status, which allows prompt provision of compatible blood if needed, despite the increased risk of anemia and added workload in the Blood Bank Laboratory.

We aimed to evaluate the percentage of high‐risk pregnant women developing clinically significant alloantibodies within 3 days of a previous negative antibody screen.

**Study Design**: Retrospective analysis of 9,155 pregnant women (2012‐2020) with an initial negative antibody screen, undergoing at least two screenings. RhD negative women were excluded from the study. This cohort was compared with 1,811 healthy male blood donors over nine months.

**Results**: Among 8,659 RhD‐positive pregnant women with initial negative screens, only 56 (0.6%) tested positive during pregnancy, with 15 (0.2%) showing clinically significant alloantibodies. Of these, only a single woman converted within 3 days, while the other 14 women did so after 5‐90 days (average 41, median 25). Two of these 15 women received transfusions (5‐8 days) before positive screens. None of the male donors showed conversion.

**Conclusion**: Only one of 8,659 pregnant women became positive within 3 days of repeated negative antibody screen, comparable to healthy males. Extending the 3‐day rule timeframe between screenings appears safe for pregnant women without transfusions.

## 269 | The missing link: Connecting placental pathology reports and clinical databases

Robin Ducharme^1^, Kasim Abdulaziz^2^, Dina El‐Demellawy^3^, Shannon Bainbridge‐Whiteside^4^, Steven Hawken^5^

^1^Ottawa Hospital Research Institute, Ottawa Hospital Research Institute, ON; ^2^BORN Ontario, Ottawa, Ontario; ^3^Children's Hospital of Eastern Ontario, Ottawa, Ontario; ^4^University of Ottawa, Ottawa, Ontario; ^5^Ottawa Hospital Research Institute, Ottawa, Ontario

11:45–13:00

**Objective**: It is well established that placental dysfunction is associated with adverse pregnancy outcomes including hypertensive disorders of pregnancy (HDP). To facilitate research involving placenta mediated diseases, we linked placental pathology findings for births from a large Canadian hospital to a population‐based birth registry database. The objective of our first study utilizing this data was to assess the associations between placental lesions and clinically documented HDP.

**Study Design**: This retrospective cohort study included deliveries at The Ottawa Hospital from 2013 to 2018 for which a placenta was collected for examination by a perinatal pathologist. An evidence‐based, validated synoptic data abstraction form was used, documenting 35 distinct placental lesions with grade of severity. We linked this pathology data to clinical data from the Better Outcomes Registry and Network (BORN). We used multivariable logistic regression to assess associations between the clinical diagnosis of HDP and placental lesions. The placental lesions assessed here include Maternal Vascular Malperfusion (MVM) and Maternal Decidual Vasculopathy (MDV).

**Results**: Pathology data were available for *n* = 2693 patients over the 5‐year study period, of which 829 (30.8%) had documented MVM, and 78 (2.9%) had MDV (Table). Clinically documented HDP was present in 953 (35.4%) patients. After adjusting for covariates, all grades of MVM were significantly associated with increased odds of HDP diagnosis, with grades 3+ being associated with the highest odds of HDP diagnosis (Odds Ratio (OR), 95% Confidence Interval (95%CI): 6.38 (3.81‐10.67). Documented MDV of Grade 1 or higher was also significantly associated with increased odds of HDP diagnosis (OR 4.85, 95% CI 2.88‐8.17).

**Conclusion**: In this study, we linked placental pathology findings to clinical data from our provincial birth registry. Our models demonstrated strong associations between MVM/MDV and HDP. These findings demonstrate the validity and potential of using this linked resource to better understand the role of placental dysfunction in maternal fetal adverse health outcomes.

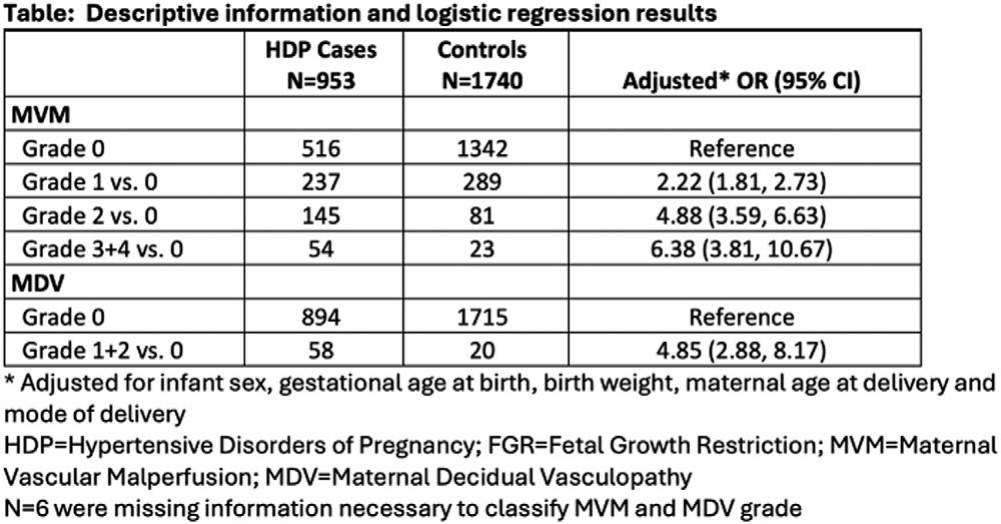

## 270 | Circulating maternal leukocytes during and after pregnancy show many dysregulated RNAs in preeclampsia

Monica Frøystad^1^, Tove Lekva^1^, Thor Ueland^2^, Marie Cecilie Paasche Roland^1^

^1^Oslo University Hospital, Oslo, Oslo; ^2^Oslo University Hospital, University of Oslo, Oslo, Oslo

11:45–13:00

**Objective**: Preeclampsia is a serious pregnancy complication affecting 2‐8 % of pregnancies worldwide. The etiology of the disease has not been fully elucidated, making early detection challenging. Early diagnosis and follow‐up can improve prognosis and potentially decrease future cardiovascular risk. We examined if mRNAs and long non‐coding RNAs (lncRNAs) in peripheral blood mononuclear cells (PBMC) were dysregulated in preeclampsia compared to normal pregnancy during pregnancy and at 5‐year follow‐up.

**Study Design**: We investigated RNA expression in PBMCs from normal pregnancy (*n* = 7) and from women developing late‐onset preeclampsia (*n* = 9), from the STORK study, at gestational weeks 22‐24 and 36‐38, as well as 5 years postpartum. Isolated RNAs from PBMCs were submitted for rRNA depletion and RNA sequencing (RNA‐seq). We used the DESeq2 package in R for differential gene expression analysis and g:Profiler for pathway analysis.

**Results**: Many RNAs were significantly differentially regulated in preeclampsia compared to normal pregnancy. At weeks 22‐24, we found 1399 upregulated RNAs (34 % lncRNA) and 12 downregulated RNAs (16 % lncRNA). At weeks 36‐38, 1759 RNAs were upregulated (2 % lncRNA) and 1210 were downregulated (27 % lncRNA). At the 5‐year follow‐up, 62 upregulated (37 % lncRNA) and 68 downregulated RNAs (31 % lncRNA) were found. Pathway analysis of downregulated RNAs at 22‐24 weeks showed programmed cell death and interleukin‐1 response as major pathways, while upregulated genes yielded developmental pathways and ECM interactions. At 36‐38 weeks the analysis yielded many infection pathways for both up‐ and downregulated RNAs. Fewer RNAs were differentially regulated at 5‐year follow‐up and only general transport pathways were found.

**Conclusion**: The presence of dysregulated RNAs at all time points might indicate that RNAs from the maternal circulation may be used to detect preeclampsia prior to clinical manifestation (22‐24 weeks) and be utilized as progression markers (36‐38 weeks). Future studies will investigate these important findings further.

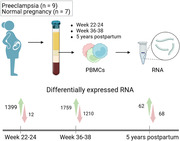

## 271 | Early onset versus late onset gestational diabetes mellitus in non‐obese women: A retrospective comparative study

Martina Garassino^1^, Libera Troìa^2^, Libera Troìa^2^, Valentino Remorgida^2^

^1^University Hospital Maggiore della carità–Università del Piemonte Orientale, Novara, Piemonte; ^2^Università degli Studi del Piemonte Orientale, Novara, Piemonte

11:45–13:00

**Objective**: Gestational Diabetes Mellitus (GDM) poses significant risks to both mothers and infants, however benefits of early diagnosis during pregnancy remain unclear.

**Study Design**: This retrospective cohort study examined the impact of early (16‐18 weeks) versus late (24‐28 weeks) diagnosis of GDM in non‐obese pregnant women (*n* = 320) at a tertiary care centre in Northern Italy. Women were screened for GDM at 16‐18 weeks using a 75‐g, 3‐hour glucose tolerance test (OGTT), if the screening was negative they were rescreened at 24‐28 weeks. GDM was diagnosed using IADPSG criteria.

**Results**: Women diagnosed early (E‐group *n* = 72) tended to be older, more frequently nulliparous and with a history of previous GDM compared to the late onset group (L‐group *n* = 278) (31.9% vs 0.3%, *p* = 0.0001). Pre‐pregnancy BMI on E‐group was higher. However, BMI at delivery was similar between the two groups because women in the E‐group had lower gestational weight gain. Women in E‐group had higher fasting blood glucose levels in the first trimester. They also had a higher likelihood of requiring insulin treatment (45.8% vs 29.4%, *p* = 0.0018) and the average daily dosage of slow insulin was higher. E‐group experienced earlier gestational age at delivery, with a higher rate of preterm birth and caesarean section (CS 36.1% vs 23%, *p* = 0.0322). While neonatal birth weight, LGA, macrosomia, Apgar score, NICU admission were comparable between groups, infants born to mothers with early‐onset GDM showed worse base excess (BE) at birth, indicating potential metabolic implications.

**Conclusion**: The incidence of adverse outcomes was more pronounced in the early‐diagnosed group. These findings emphasize the importance of monitoring fasting blood glucose levels in the first and second trimesters for timely intervention. Early diagnosis of GDM may warrant closer monitoring and tailored interventions to mitigate adverse outcomes for both mother and child.

## 272 | Does the use of Cervidil impact delivery outcomes in women with parity above three births?

Hadar Gluska^1^, Gal cohen^2^, chen manor^3^, Hanoch Schreiber^3^, Dorit Ravid^3^

^1^Department of Obstetric and Gynecology, Meir Medical Center, Raanana, HaMerkaz; ^2^Meir Hospital, Meir Hospital, HaMerkaz; ^3^Meir Medical Center, kfar saba, HaMerkaz

11:45–13:00

**Objective**: Vaginal PGE2 also known as Cervidil is an acceptable form for labor induction. It is believed that women with more than 3 previous births should refrain from the use of Cervidil, since it may potentially cause rupture of uterus. However, this assumption has not yet been validated by clinical studies. Our aim was to examine the association between parity above 3 and adverse maternal and neonatal outcomes, among women who underwent induction of labor with Cervidil.

**Study Design**: This retrospective cohort study included all pregnant women who went through induction of labor with Cervidil between the years 2014 to 2020 and gave birth at our department. The study cohort was stratified by number of past births: up to 3 (Parity ≤ 3 group) and above 3 (Parity ≥ 4 group). Maternal characteristics and pregnancy outcomes were compared between these groups.

**Results**: A total of 3295 women underwent induction of labor with Cervidil were included in the study. Among them 3235 where in the Parity ≤ 3 group, and 60 where in the Parity ≥ 4 group. Women in the Parity ≥ 4 group were older than their counterpart s (34.8 ± 4.1 vs. 30.9 ± 5.6, *p* < 0.001), and had higher BMI (28.5 ± 5.0 vs. 25.6 ± 5.9, *p* = 0.020). Gestation age at delivery was slightly higher in the Parity ≤ 3 group (40.0 ± 1.4 vs. 39.5 ± 1.4, *p* = 0.002). Women in the Parity ≥ 4 group had more successful vaginal births (54(90%) vs. 2321(71.7%), *p* = 0.002(, with less vacuum extraction 3.3%)2() vs. 309(12.3%), *p* < 0.028) and less cesarian sections (4(6.7%) vs. 513(15.9%), *p* = 0.050). There were no differences between the groups in meconium aspiration, blood loss during labor, neonatal birth weight, neonatal Apgar score < 5, cord pH < 7.1 and neonatal composite outcome. In both groups none of the women had uterine rupture.

**Conclusion**: The study findings suggest no inferiority in the use of Cervidil in women with parity > 3 comparing to women with up to 3 births with no differences in adverse maternal or neonatal outcomes. If corroborated by further studies these findings may reassure clinicians when choosing to perform induction of labor with PGE2 in women with more than 3 births.

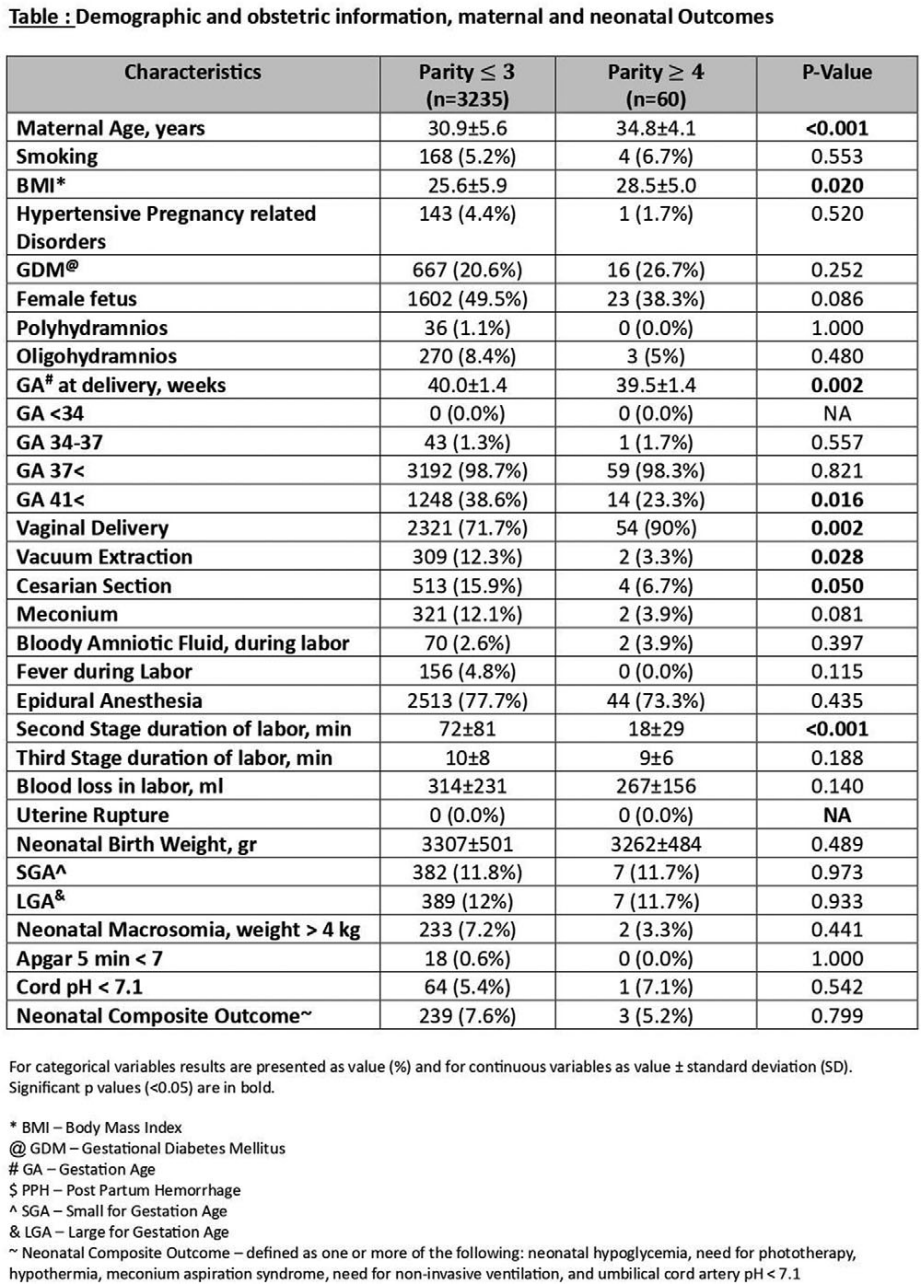

## 273 | Impact of revised criteria set on electronic early‐warning infection alerts: A multi‐site pilot study

Isaac Michaels^1^, Lejdisa Stanaj^2^, Brianne Genow^2^, Baruch Fertel^1^, Jason Adelman^3^, Benjamin Ranard^4^, Dena Goffman^5^

^1^NewYork Presbyterian, NewYork Presbyterian, New York; ^2^NewYork Presbyterian, NewYork Presbyterian/NY, New York; ^3^Columbia University Irving Medical Center/NewYork Presbyterian, New York, New York; ^4^Columbia University Irving Medical Center, New York, New York; ^5^Columbia University Medical Center, New York, New York

11:45–13:00

**Objective**: To evaluate the impact of implementing Modified Systemic Inflammatory Response Syndrome (SIRS) with heart‐rate threshold of 120 beats/minute (revised criteria set), compared to adult SIRS (baseline criteria set) on the rate of electronic early‐warning alerts for sepsis risk during delivery visits.

**Study Design**: A multi‐site pilot study was conducted at a large, urban, academic hospital system. The revised criteria set, identified through simulation studies, was implemented in the electronic health record system. Incidence rates of alerts firing during a pre‐pilot period (2022‐03‐13 through 2022‐11‐13) and during the pilot period (2023‐07‐01 through 2024‐03‐01) were compared. Clinician chart reviews were performed on pilot‐period delivery encounters with a sepsis diagnosis recorded, to identify false negatives in which sepsis onset without the alert firing beforehand.

**Results**: There were 48,971 delivery encounters during the pre‐pilot period, and 48,471 during the pilot period. The baseline criteria set triggered at least one alert in 7.5% (*n* = 3,695) of the pre‐pilot period encounters. The revised criteria set triggered at least one alert in 1.9% (*n* = 923) of the pilot‐period encounters. Clinician chart reviews identified zero false negatives during the pilot period.

**Conclusion**: Implementation of the revised criteria set led to a substantial decrease in the incidence rate of early‐warning alerts for sepsis risk during delivery encounters. These findings suggest that the revised criteria set has better specificity for sepsis than the baseline criteria set–while maintaining high sensitivity. Further studies are warranted to assess the long‐term sustainability and generalizability of these results.

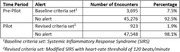

## 274 | A retrospective comparison of twin specific growth charts with singleton growth charts for MCDA twins

Edward Golob^1^, Nouran Elbarabry^2^, Ramesh Ganapathy^3^

^1^Epsom and St Helier NHS trust, Epsom General Hospital London, England; ^2^St George's Hospital, London, England; ^3^Epsom and St Helier NHS trust, London, England

11:45–13:00

**Objective**: Fetal growth follows a normal distribution but twin fetuses are relatively smaller at birth than singletons. Small for gestational age (SGA) fetuses are a heterogenous group but some will be affected by fetal growth restriction (FGR). Growth charts for singletons classify a disproportionate number of twin fetuses as < 10th centile. Twin specific growth charts potentially reflect the distribution of growth in twins better, detecting higher rates of FGR in those < 10th centile. The objective of this study was to compare the rates of SGA and neonatal outcomes between singleton and twin specific growth charts.

**Study Design**: Retrospective data was obtained from 145 monochorionic diamniotic (MCDA) twin pregnancies over a period of 13 years. The final growth scan performed prior to delivery was available with fetal weight calculated according to a singleton growth centile. From this data the equivalent centile on a twin specific chart was derived to analyse how it correlated with outcomes and whether it would have impacted management.

**Results**: Growth centiles according to twin charts more closely matched a normal distribution whilst singleton charts had the effect of shifting centiles to the left. On singleton charts more than 31% of pregnancies were classified as having one or both fetuses being SGA whilst on twin specific charts this was 13%. Rates of iatrogenic prematurity and neonatal admissions were higher in the SGA group according to twin centiles. In the group where one or both fetuses were below the 10th centile on a singleton chart the rate of neonatal unit admission was 25/45 (55%) and the rate of iatrogenic prematurity was 13/45 (28.8%). In the group where one or both twins was found to be below the 10th centile on a twin chart the rate of neonatal admission was 15/20 (75%) and the rate of iatrogenic prematurity was 12/20 (60%).

**Conclusion**: Twin centiles more accurately reflect a normal distribution. SGA according to twin centiles likely included more fetuses affected by FGR. This would have been unlikely to alter management as delivery of all MCDA twins was advised < 37 weeks.

## 275 | Performance of the sFlt‐1/PLGF ratio in the short term diagnosis of preeclampsia

Edward Golob^1^, sarah Davie^2^, Ramesh Ganapathy^3^

^1^Epsom and St Helier NHS trust, Epsom General Hospital London, England; ^2^Kingston Hospital, London, England; ^3^Epsom and St Helier NHS trust, London, England

11:45–13:00

**Objective**: In 2022 the United Kingdom's National Institute for Health and Care Excellence (NICE) approved the use of angiogenic biomarkers for the prediction of preeclampsia. We have been using the Elecsys® (Roche) sFlt‐1/PlGF immunoassay in our centre since April 2022 to assess patients with suspected preeclampsia. The objective of this retrospective study was to assess the performance of the test in the short term prediction of preeclampsia.

**Study Design**: An electronic database of all samples sent for sFlt‐1/PLGF testing is maintained by the performing laboratory. A retrospective analysis of samples (*n* = 276) collected between April 2022‐ October 2023 was performed. The results of all samples were then cross referenced with electronic maternity notes (Badgernet) in order to determine outcomes for each case. The short term prediction of preeclampsia according to a threshold suggested in NICE guidance was assessed. A ratio of < 38 was used to rule out a diagnosis of preeclampsia for 1 week, whilst a ratio >38 was used to rule in preeclampsia within 4 weeks.

**Results**: 276 samples were performed on 216 pregnancies. 6 patients were lost to follow up and excluded. 270 samples were analysed, including 60 repeat tests. The predictive performance was assessed for each test according to the above criteria.

212 samples had a ratio < 38, ruling out preeclampsia for 1 week. In this group the incidence of preeclampsia within 1 week was 6/212 (2.8%). 58 samples returned a ratio >38. The incidence of preeclampsia within 4 weeks was (72.4% *n* = 42), 12 cases did not develop preeclampsia within 4 weeks (20.6%), 4 cases delivered in less than 4 weeks and had not developed preeclampsia at this point. These 4 cases were not included in analysis of accuracy as they did not remain pregnant for 4 weeks. For the 266 cases analysed the AUC was 0.91 (Sensitivity: 87.5% Specificity: 94.5%).

**Conclusion**: The accuracy of the sFlt‐1/PLGF ratio for the short term prediction was preeclampsia was high. The challenge for clinicians is to translate this diagnostic accuracy into improvements in care, reductions in adverse events and cost savings.

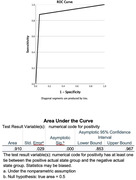

## 276 | Trends of trial of labor in twin gestations after cesarean delivery in the United States

Julie Gomez^1^, Ariana Spiegel^2^, Kavisha Khanuja^2^, Rodney A. McLaren, Jr., Jr.^2^

^1^Thomas Jefferson University, Philadelphia, PA; ^2^Thomas Jefferson University Hospital, Philadelphia, Pennsylvania

11:45–13:00

**Objective**: ACOG states that twins with a prior cesarean delivery who are candidates for a twin vaginal delivery are candidates for trial of labor after cesarean (TOLAC). Whether this practice is performed in patients with a prior cesarean delivery for twin delivery is not well known. Thus, this study's objective is to evaluate the trend of TOLAC among twin deliveries who are candidates for TOLAC.

**Study Design**: A retrospective cross‐sectional study was performed using the National Vital Statistics System birth data from 2015 to 2022. Data from patients with twin gestation and one prior cesarean delivery were included. Data from patients were excluded for delivery prior to 28 weeks gestation, known fetal anomaly, intrauterine fetal demise of either twin, or if fetal lie of presenting twin was not vertex. Rates of TOLAC in twin pregnancies following one prior cesarean delivery were evaluated over time. In addition, twin vaginal birth after cesarean delivery (VBAC) were calculated among those who had a TOLAC. Cochrane‐Armitage trend analysis was performed to evaluate trends in TOLAC and VBAC.

**Results**: There were 39,186 total candidates for TOLAC between 2015 and 2022. The rate of TOLAC in twin gestations following one prior cesarean delivery has increased from 11.0% in 2015 to 12.2% in 2022, but was not statistically significant (*p* = 0.056). The rate of successful VBAC has increased from 62% in 2015 to 69% in 2022 (*p* < 0.001).

**Conclusion**: Rates of twin VBAC have significantly increased from 2015 to 2022. However, the rates of TOLAC remain overall low in twin gestations following one prior cesarean delivery. Further investigation is needed to determine why TOLAC rates have remained low in the United States so we can continue to support TOLAC in patients with twin gestation after one cesarean delivery.

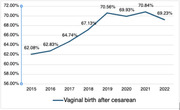

## 277 | AI‐enabled ultrasound in prenatal screening for fetal cardiac abnormalities: A systematic review

Elena D'Alberti^1^, Olga Patey^2^, Netzahualcoyotl Hernandez‐Cruz^2^, J Alison Noble^2^, Aris Papageorghiou^2^

^1^Università di Roma Sapienza, Roma, Lazio; ^2^University of Oxford, Oxford, England

11:45–13:00

**Objective**: To examine the performance of artificial intelligence (AI) in the detection of fetal cardiac defects.

**Study Design**: A systematic search of PubMed, EMBASE and CINAHL was performed up to February 2024. We included studies that undertook validation of two‐dimensional (2D) fetal cardiac ultrasound image recognition using AI models from 16 weeks of gestation. Adherence to reporting standards and risk of bias were assessed using TRIPOD and QUADAS‐2 tools, respectively. As some studies also reported training of models within the same publication, only validation data were assessed.

**Results**: Eleven studies met the inclusion criteria, analyzing 20,910 records (images or videos) using AI‐recognition of normal vs abnormal fetal hearts. Video analysis was used in 2 studies and automatic cardiac biometry in another 2; cardiac Doppler was not investigated. An important source of heterogeneity was the views collected: 4 studies used the four‐chamber view alone, 5 used all five axial views recommended by AIUM and ISUOG, and 2 analyzed sagittal views. Seven studies pooled cases as “any heart defect” while 4 assessed specific defects. Seven articles compared AI with clinical screening programs: AI performed better than operators with lower expertise and at least comparable to experts, but human comparators (median 9, IQR 4‐23 clinicians) were usually non‐blinded. Meta‐analysis was not possible due to lack of data for 2x2 tables. All studies were from tertiary or university hospitals, and 9/11 studies were from high‐income countries. The overall risk of bias and adherence to reporting standards were moderate (> 70% of adherence for 19/29 TRIPOD items).

**Conclusion**: Despite their promise, few studies currently exist on AI detection of fetal heart defects. The paucity of studies from community‐screening centers and low/middle income countries limits the generalizability of the findings to settings where AI might be of greatest benefit. We call on researchers to follow reporting standards to reduce the risk of bias and sources of heterogeneity.

## 278 | The impact of institutional culture on the implementation of a Maternal Equity Safety Bundle

Anna K. Daoud^1^, Elysia Larson^2^, Tonia Janae Rhone^3^, Heather Olden^4^, Kali Vitek^5^, Hafsatou Diop^6^, Ndidiamaka Amutah‐Onukagha^7^, Audra R. Meadows^8^

^1^NewYork‐Presbyterian/Weill Cornell Medical Center, New York, NY; ^2^Department of Obstetrics and Gynecology, Beth Israel Deaconess Medical Center, Boston, Massachusetts; ^3^UT Southwestern Medical Center, Dallas, Texas; ^4^Harvard T.H. Chan School of Public Health, Boston, Massachusetts; ^5^The Perinatal Neonatal Quality Improvement Network of Massachusetts (PNQIN), Boston, Massachusetts; ^6^Massachusetts Department of Public Health, Boston, Massachusetts; ^7^Tufts University School of Medicine, Boston, Massachusetts; ^8^University of San Diego Medical Center, La Jolla, California

11:45–13:00

**Objective**: Culture is a modifiable organizational factor that impacts quality, outcomes, and care delivery processes. This study describes the impact of institutional culture on the experience and success of implementing an Equity Maternal Safety Bundle (MSB) aimed at reducing severe maternal morbidity, improving patient experience, and eliminating racial inequities.

**Study Design**: Focus group discussions (FGDs) were conducted in Fall 2022 and Fall 2023, before and after Equity MSB implementation among obstetric nurses, residents, and attending physicians. FGDs were facilitated using a semi‐structured guide developed using the Consolidated Framework for Implementation Research (CFIR). A codebook was developed using CFIR for deductive coding and inductive codes were added as appropriate. Transcripts were independently coded by two analysts using NVivo 14. Themes were generated through an iterative process and compared across study time points.

**Results**: Fifteen clinicians participated in FGDs at each time point with similar distributions across race, ethnicity, gender, and profession. Across time points, participants discussed needing culture change for this bundle, rather than solely practice change. Participants used the terms individualized, patient‐oriented, acknowledging inequities, and the gold standard to define a culture of equity. Participants described administration, residents, and nurses as responsible for establishing culture, though nurses were most acknowledged as culture drivers. Leadership pushed for culture change by prioritizing equity. Department cultures resistant to change and embedded with implicit biases were implementation barriers.

**Conclusion**: Participants named cultures of equity, accountability, and quality improvement as facilitators when implementing equity‐focused projects. A culture of equity was defined as individualized, gold‐standard care that accommodates all patient needs and preferences while acknowledging existing inequities and providing a sense of belonging. Leadership priorities and staff turnover over the last three years resulted in a culture shift towards equity.

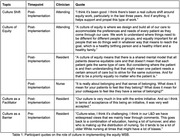

## 279 | Effect of aspirin discontinuation at 24‐28 weeks on the trajectories of biophysical and biochemical markers

Marta Dalmau^1^, Ester del Barco^1^, Mireia Armengol‐Alsina^1^, Erika Bonacina^1^, Manel Mendoza^1^, Anna Suy^2^, Elena Carreras^1^

^1^Vall d'Hebron University Hospital, Vall d'Hebron Hospital, Catalonia; ^2^Vall d'Hebron University Hospital, Barcelona, Catalonia

11:45–13:00

**Objective**: Discontinuation of aspirin in women at an increased risk of preeclampsia (PE) during first‐trimester screening, with normal angiogenic factors at 24‐28 weeks of gestation, does not heighten the risk of preterm PE. Moreover, aspirin does not affect mean arterial blood pressure (MAP), mean uterine artery pulsatility index (UtAPI), or biomarkers trajectories after 20 weeks of gestation. Our aim was to investigate the impact of aspirin discontinuation at 24‐28 weeks on the trajectories of biophysical and biochemical markers.

**Study Design**: This is a longitudinal secondary analysis of the Detection of False Positives From First‐trimester Preeclampsia Screening at the Second‐trimester of Pregnancy (StopPRE) trial, using repeated measures of MAP, UtAPI, placental growth factor (PlGF), and soluble fms‐like tyrosine kinase‐1 (sFlt‐1). In the trial, 936 women at high risk for PE during the first‐trimester screening received aspirin 150 mg daily from screening to randomization. At 24‐28 weeks, 463 women were randomly allocated to continue aspirin until 36 weeks, while 473 were assigned to discontinue aspirin. All markers were assessed at baseline (24‐28 weeks) and follow‐up visits at 28‐32, 32‐36, and after 36 weeks. Generalized estimating equations procedure with treatment by gestational age interaction were used to explore the effect of aspirin on markers' trajectories over time.

**Results**: Among all participants, there were 3,193 MAP, 2,954 UtAPI, and 3,437 biomarker measurements. Trajectories of MAP, UtAPI, PlGF, sFlt‐1, and sFlt‐1/PlGF raw values and percentiles or multiples of the median (MoM) showed no significant differences between the groups (*p*‐values for raw analysis: 0.804, 0.353, 0.211, 0.852, and 0.802, respectively).

**Conclusion**: In women at an increased risk of preterm PE, discontinuation of aspirin at 24‐28 weeks of gestation does not affect the trajectories of MAP, UtAPI, PlGF, sFlt‐1, and sFlt‐1/PlGF. This finding may help explain why discontinuing aspirin did not lead to an increased risk of preterm PE in the StopPRE trial.

## 280 | Sustainability of maternal and newborn health programs: What can we learn from Tanzania?

Florina Serbanescu^1^, Ahmad M. Makuwani^2^, Abdulaziz Msuya^3^, Sunday A. Dominico^3^, Alicia Ruiz^4^, Carrie Shapiro‐Mendoza^4^

^1^Centers for Disease Control and Prevention, Atlanta, GA; ^2^Division of Reproductive, Maternal and Child Health; Tanzania Ministry of Health, Dodoma, Dodoma; ^3^Tanzania Ministry of Health, Dar es Salaam, Dar es Salaam; ^4^Centers for Disease Control and Prevention, Atlanta, Georgia

11:45–13:00

**Objective**: Reducing maternal mortality is a global priority. During 2013–2018, a donor‐supported Program to Reduce Maternal Deaths was successfully implemented in Kigoma, Tanzania. The program increased access to emergency obstetric and reproductive health care, resulting in 43% maternal and 29% neonatal mortality declines. Because health benefits may diminish or disappear after donor funding ends, program sustainability is unknown. To understand sustainability, we compared the status of maternal and newborn health services and outcomes in 2018 with the status 4 years after the program ended

**Study Design**: The sustainability assessment used multiple data collection methods. They included facility assessments, pregnancy outcomes monitoring, and Rapid Ascertainment Process of Institutional Death methodology to extract, verify, and triangulate data on maternal deaths from all available sources (patient records, registers, nurses’ notebooks, morgue) in 202 health facilities. Z‐tests were used to assess significance of change

**Results**: From 2018 to 2022, the number of facility births rose from 85,187 to 88,421. The number of facilities providing all elements of emergency obstetric and newborn care (EmONC) rose from 21 to 27; partial EmONC was available in an additional 56 facilities. The population cesarean section rate increased by 69% (from 4.5% to 7.6%). The proportion of women with childbirth complications treated in EmONC facilities rose by 26% (from 48.3% to 60.9%). Case‐fatality rate fell by 21% (from 1.4% to 1.1%). Maternal mortality in all facilities continued to decline (17%, from 174 to 145 maternal deaths per 100,000 live births) as did pre‐discharge neonatal mortality (25%, from 7.6 to 5.7 neonatal deaths per 1,000 live births).

**Conclusion**: A sustainability evaluation found that maternal and newborn health services and outcomes continued to improve in Tanzania after the Program to Reduce Maternal Deaths ended, demonstrating that program gains can be sustained. Lessons learned from Tanzania can inform program sustainability where similar approaches are used to reduce preventable maternal and newborn deaths.

## 281 | Investigation of Methacrylated Carboxymethyl Chitosan as a tissue engineering material for fetal therapy

Hyun Sun Ko^1^, Ahyoung Kim^2^, Jeong Ha Wie^2^, Hyeon Soo Kim^2^, Heung Jae Chun^2^, Dae Hyeok Yang^2^

^1^Department of Obstetrics and Gynecology, Catholic University College of Medicine/ Seoul, Catholic University College of Medicine/ Seoul, Seoul‐t'ukpyolsi; ^2^The Catholic University of Korea, The Catholic University of Korea, Seoul‐t'ukpyolsi

11:45–13:00

**Objective**: The intrauterine environment for fetal therapy necessitates a specific biomaterial tailored to treat congenital diseases. This material needs to effectively isolate the lesion from the surrounding amniotic fluid in a waterproof manner and remain flexible to accommodate changes in the skin surface area during fetal growth, as well as facilitate the regeneration of the defect area. In the previous study, we reported that a visible light‐curing system is useful as a preparation tool for injectable hydrogel systems because it can avoid tissue damage resulting from excessive exposure to ultraviolet light and visible light‐curable methacrylated glycol chitosan (MGC) hydrogel patches suggested the therapeutic potential for the prenatal treatment of fetal myelomeningocele. The objective of this study was to investigae CMCSMA hydrogel as a tissue engineering material for fetal therapy.

**Study Design**: We prepared visible light‐curable MGC and the methacrylation of carboxymethyl chitosan (CMCSMA) hydrogel patches, which had been suggested as a valuable scaffold for restoring cortical and cancellous bone defects, for the prenatal treatment of fetal gastroschisis, in a rat model.

**Results**: 6 w/v% MGC hydrogel and 7 w/v% CMCSMA hydrogel demonstrated comparable decrease of abdominal wall defects and epithelial regeneration. When decidual stem cells (DSC) were added in 7 w/v% CMCSMA hydrogel, reduction of the defect and epithelization were significantly improved, compared to MGC hydrogel and CMCSMA hydrogel only.

**Conclusion**: Visible light‐curable CMCSMA hydrogel, which is more stable, less expensive, and easily accessible, compared to MGC hydrogel, may be a possible candidate for fetal therapy. (supported by the National Research Foundation of Korea (NRF) grant funded by the Korea government (MSIT) (No. 2020R1A2C1007588)) and the Ministry of Trade, Industry and Energy (MOTIE) (No. 20018324, 20017645, 20014222, 20017939, and 20004627) of Republic of Korea.

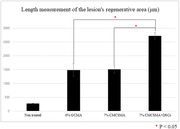

## 282 | Circulating angiogenic factors for stratifying the risk of preeclampsia with severe features

Anders Berg^1^, Martha Bautista^1^, Ge Guo^2^, Martin Hund^3^, S Ananth Karumanchi^1^

^1^Cedars‐Sinai Medical Center, Los Angeles, California; ^2^Roche Diagnostics Operations, Inc., Indianapolis, Indiana; ^3^Roche Diagnostics International, Rotkreuz, Zug

11:45–13:00

**Objective**: Prior single center studies in women with suspected preeclampsia indicated that the ratio of serum soluble fms‐like tyrosine kinase 1 (sFlt‐1) to placental growth factor (PlGF) measured on Elecsys platform at a cut‐off of 38 predicted preeclampsia with severe features (sPE) within two weeks. We sought to validate the Elecsys® sFlt‐1/PlGF cut‐off of 38 for the prediction of sPE in the prospective PRAECIS cohort that recruited patients with a hypertensive disorder of pregnancy (HDP; gestational hypertension, chronic hypertension, de novo and superimposed preeclampsia) across 18 tertiary and community hospitals in the US.

**Study Design**: We measured sFlt‐1/PlGF ratio using the Roche Elecsys platform in archived, frozen serum samples of the PRAECIS study that recruited hospitalized pregnant women with a HDP between 23 and 35 weeks of gestation. The primary study outcome was prediction of sPE within two weeks after testing. We also evaluated time to delivery in women whose sFlt‐1/PlGF ratio was above the cut‐off of 38 compared to those whose sFlt‐1/PlGF ratio was at or below that cut‐off.

**Results**: In the PRAECIS validation cohort of 556 women with a HDP, the Elecsys sFlt‐1/PlGF cut‐off ratio >38 demonstrated 66.9% positive predictive value (95% CI, 60.8 to 72.7) and 94.7% negative predictive value (95% CI, 91.5 to 96.9) for the progression to sPE within two weeks. The sensitivity was 91.4% (95% CI 86.4% to 95.0%) and specificity was 77.3% (95% CI 72.7% to 81.5%) for the primary outcome. The sFlt‐1/PlGF ratio performed better than standard‐of‐care clinical measures, with an area under the receiver‐operating characteristic curve of 0.92 for sFlt‐1/PlGF ratio versus 0.74 for standard‐of‐care tests. Among the women who delivered within two weeks of presentation 68.9% had an sFlt‐1/PlGF ratio >38 compared to 31.1% with a ratio ≤38 (hazard ratio 3.1 for delivery within two weeks; 95% CI 2.5 to 3.7, *p* < 0.001).

**Conclusion**: The Elecsys sFlt‐1/PlGF ratio >38 is useful to predict development of sPE within two weeks in pregnant women between 23 and 35 weeks of gestation being hospitalized with a HDP.

## 283 | Blood pressure in pregnancy and hypertension 10‐to‐14 years after delivery

Kartik K. Venkatesh^1^, William A. Grobman^1^, Jiqiang Wu^1^, Maged M. Costantine^1^, Mark B. Landon^1^, Denise Scholtens^2^, William Lowe^2^, Nilay S. Shah^3^, Natalie A. Cameron^4^, Sadiya S. Khan^5^

^1^The Ohio State University, Columbus, Ohio; ^2^Northwestern University, Northwestern University/Chicago, Illinois; ^3^Northwestern University, Chicago, Illinois; ^4^Northwestern, Northwestern/Chicago, Illinois; ^5^Northwestern University Feinberg School of Medicine, Chicago, Illinois

11:45–13:00

**Objective**: To examine the association between blood pressure (BP) in the early third trimester and hypertension 10‐14 years after delivery.

**Study Design**: This is a secondary analysis from the prospective Hyperglycemia and Adverse Pregnancy Outcome (HAPO) Follow‐Up Study of individuals without a clinical diagnosis of chronic hypertension in pregnancy or HDP. The exposure was BP in the early third trimester, and the outcome was BP 10‐to‐14 years after delivery. At both timepoints, BP was measured using a standardized research protocol, and categorized per American College of Cardiology (ACC)/American Heart Association (AHA) thresholds: normal (B*p* < 120/ < 80 mmHg, reference), elevated (BP 120‐129/ < 80 mmHg), and hypertension (stage 1 or 2 [BP ≥130/≥80 mmHg], clinical diagnosis, or antihypertensive treatment). Ordinal logistic regression was used to estimate the association between pregnancy and postpartum BP categories, and adjusted for baseline covariates.

**Results**: We assessed 4,693 pregnant individuals without chronic hypertension nor HDP at a median gestational age of 27.9 weeks and median age of 30.6 years. At 10‐to‐14 years after delivery (median age of 42.1 years), 17.4% of individuals with normal BP in the early third trimester had hypertension, while 38.9% of individuals with elevated BP and 52.5% with stage 1 or higher BP in pregnancy had hypertension (*p* < 0.001). In adjusted analyses, compared with normal BP, BP in the early third trimester that met incrementally higher ACC/AHA thresholds of elevated BP (aOR: 2.80; 95% CI: 2.13, 3.67) and hypertension (aOR: 3.92; 95% CI: 3.15, 4.90) were associated with greater risks of higher BP 10‐to‐14 years after delivery.

**Conclusion**: Pregnant individuals with elevated BP in the third trimester without a clinical diagnosis of hypertension were at greater risk of hypertension 10‐to‐14 years after delivery. These results highlight the potential impact of using ACC/AHA criteria to define BP thresholds in pregnancy.

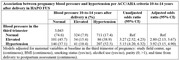

## 284 | Dysglycemia in pregnancy and predicted risk of Long‐Term Postpartum Cardiovascular Disease

Kartik K. Venkatesh^1^, William A. Grobman^1^, Jiqiang Wu^1^, Maged M. Costantine^1^, Mark B. Landon^1^, Denise Scholtens^2^, William Lowe^2^, Nilay S. Shah^3^, Sadiya S. Khan^4^

^1^The Ohio State University, Columbus, Ohio; ^2^Northwestern University, Northwestern University/Chicago, Illinois; ^3^Northwestern University, Chicago, Illinois; ^4^Northwestern University Feinberg School of Medicine, Chicago, Illinois

11:45–13:00

**Objective**: To examine whether dysglycemia in pregnancy is associated with a higher long‐term predicted risk of atherosclerotic cardiovascular disease (ASCVD) postpartum.

**Study Design**: This is a secondary analysis of data from the Hyperglycemia and Adverse Pregnancy Outcome (HAPO) Follow‐up Study. The exposure was glucose level defined by a summary or mean z‐score incorporating the fasting, 1‐, and 2‐hour values of a 75‐gram oral glucose tolerance test (OGTT) at ∼28 weeks gestation; and, secondarily, each z‐score at the three timepoints assessed separately. The primary outcomes were estimates of the predicted 10‐ and 30‐year ASCVD risk (composite of fatal and non‐fatal coronary heart disease and stroke) as continuous measures. Linear regression models adjusted for covariates measured at the baseline pregnancy visit when the OGTT was performed: study field center, age, body mass index, smoking and alcohol use, parity, and time from delivery to postpartum assessment.

**Results**: Among 4,545 assessed pregnant individuals, the median age was 30.6 years and median gestational age was 27.9 weeks at enrollment. At 10‐14 years after delivery at a median age of 41.1 years, the median predicted risk of ASCVD over the following 10 years was 1.8% (IQR: 1.2%, 2.7%) and 30 years was 5.0% (IQR: 3.3%, 7.7%). In adjusted analyses, for each 1‐unit higher glycemia summary z‐score, the estimated 10‐year ASCVD risk was higher by 0.30% (95% CI: 0.23 to 0.37), and the estimated 30‐year ASCVD risk was higher by 0.89% (95% CI: 0.70 to 1.08). These associations between glycemia z‐score and 10‐ and 30‐year predicted risk of ASCVD persisted when assessed separately at fasting, 1‐hour, and 2‐hour timepoints (TABLE).

**Conclusion**: There is a dose‐response relationship between pregnancy dysglycemia in the early third trimester and the predicted 10‐and 30‐year risk of ASCVD postpartum.

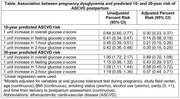

## 285 | Choosing for a homebirth during the COVID‐19 lockdown in the Netherlands: Who and why?

Benjamin Y. Gravesteijn^1^, Nienke Boderie^2^, Roseriet Beijers^3^, Loes Bertens^2^, Thomas van den Akker^4^, Jeroen van dillen^5^, Arie Franx^6^, marion van den heuvel^7^, Ank de Jonge^8^, Brenda M. M. Kazemier^9^, Igna Kwint‐Reijnders^10^, Ben W. Mol^11^, Sylvia Oberman^10^, Lilian Peters^12^, Stefania Vacaru^3^, Carolina de Weerth^3^, Sam Schoenmakers^13^, Christianne J.M. de Groot^9^, Jasper Been^2^

^1^Amsterdam UMC, Amsterdam UMC, Noord‐Holland; ^2^Erasmus MC, Erasmus MC, Zuid‐Holland; ^3^Radboud University Medical Center, Radboud University Medical Center, Gelderland; ^4^Leiden University Medical Center, LUMC/Leiden, Zuid‐Holland; ^5^Radboud UMC, Radboud UMC, Nijmegen, Gelderland; ^6^Erasmus University Medical Center, Zuid‐Holland; ^7^Tranzo Scientific Centre for Care and Wellbeing, Tilburg University, Tranzo Scientific Centre for Care and Wellbeing, Tilburg University, Noord‐Brabant; ^8^Amsterdam UMC, Amsterdam UMC, Noord‐Brabant; ^9^Amsterdam UMC, Amsterdam, Noord‐Holland; ^10^Care4Neo, Care4Neo, Zuid‐Holland; ^11^Monash University, Clayton, Victoria; ^12^UMC Groningen, UMC Groningen, Groningen; ^13^Erasmus University Medical Centre, DEN HAAG

11:45–13:00

**Objective**: During the first lockdown in the Netherlands, homebirth rate increased from 27% to 37% among women in primary care. This study explores characteristics and motivations of women that chose for a homebirth instead of a hospital birth.

**Study Design**: We used the COPE study, a nationwide online survey distributed during the first lockdown in the Netherlands. We compared characteristics of pregnant women who report to have changed their intended birth location from a hospital to home birth to those that did not change their intended location of birth. Moreover, we compared anticipatory worries, anxiety levels, depression levels, and birth experiences in relation to COVID‐19 and mitigation measures.

**Results**: We analyzed data from 1206 pregnant women. The most reported change in planned location of birth was from a hospital to a home birth (13%; 84% reported no change) which was primarily experienced as a choice rather than compulsory (81%). Women who report changing their preferred location of birth to a homebirth were mainly different to women that did by having less self‐reported risk factor during pregnancy (3% versus 16%). They also had significantly higher depression (*p* = 0.004) anxiety (*p* = 0.027) and COVID‐19 related worries (*p* < 0.001); the most important worries were whether friends and family could visit post‐partum and whether chosen support would be available during labor.

**Conclusion**: During the COVID‐19 pandemic in the Netherlands, mainly women with no risk factors during pregnancy chose to give birth at home. This may have been motivated by COVID‐19 related worries pertaining to a hospital birth.

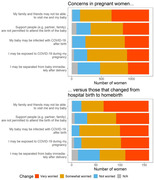

## 286 | COVID‐19 lockdown and maternity care and maternal outcome in the Netherlands: A national quasi‐experimental‐study

Benjamin Y. Gravesteijn^1^, Nienke Boderie^2^, Thomas van den Akker^3^, Loes Bertens^2^, Kitty bloemenkamp^4^, Lizbeth Burgos Ochoa^5^, Ank de Jonge^6^, Brenda M. M. Kazemier^7^, Peter Paul Klein^8^, Igna Kwint‐Reijnders^9^, Jeremy Labrecque^2^, Ben W. Mol^10^, Sylvia Oberman^9^, Lilian Peters^11^, Anita C. Ravelli^7^, Ageeth Rosman^12^, Jasper Been^2^, Christianne J.M. de Groot^7^

^1^Amsterdam UMC, Amsterdam UMC, Noord‐Holland; ^2^Erasmus MC, Erasmus MC, Zuid‐Holland; ^3^Leiden University Medical Center, LUMC/Leiden, Zuid‐Holland; ^4^University Medical Center Utrecht, University Medical Center Utrecht, Utrecht; ^5^Tilburg University, Tilburg University, Noord‐Brabant; ^6^Amsterdam UMC, Amsterdam UMC, Noord‐Brabant; ^7^Amsterdam UMC, Amsterdam, Noord‐Holland; ^8^RIVM, RIVM, Noord‐Holland; ^9^Care4Neo, Care4Neo, Zuid‐Holland; ^10^Monash University, Clayton, Victoria; ^11^UMC Groningen, UMC Groningen, Groningen; ^12^Perined, Perined, Zuid‐Holland

11:45–13:00

**Objective**: The COVID‐19 pandemic and associated lockdowns disrupted healthcare worldwide. High‐income countries observed a decrease in preterm births, but maternal pregnancy‐related outcomes were also likely affected. This study investigates the effect of the first COVID‐19 lockdown in the Netherlands on provision of maternity care and maternal pregnancy‐related outcomes.

**Study Design**: Multiple linked national registries were used; all births from a gestational age of 24 weeks in 2010‐2020 were included. In births starting in midwife‐led primary care, we assessed the effect of lockdown on provision of care. In the general pregnant population, the impact on labour onset, mode of birth, obstetric anal sphincter injury (OASI), episiotomies, and post‐partum haemorrhage (>1000ml) was assessed. A difference‐in‐regression‐discontinuity design was used to estimate causal effects.

**Results**: A total of 1,039,728 births were included. During lockdown, births among women who started labour in midwife‐led primary care (49%) more often ended at home (27% pre‐lockdown, +10%[95% CI:+7%; +13%]). A small decrease was seen in referrals towards obstetrician‐led care during labour (46%, −3%[‐5%;‐0%]). In the general population, no change was seen in induction of labour (27%, +1%[‐1%; +3%]). We found no significant changes in the incidence of emergency caesarean section (9%, −1%[‐2%; +0%]), OASI (2%, +0%[‐0%; +1%]), episiotomy (21%, −0%[‐2%; +1%]), or post‐partum haemorrhage (6%, −0%[‐1%; +1%]).

**Conclusion**: During the first COVID‐19 lockdown in the Netherlands, a substantial increase in homebirths was seen. Registered maternal outcomes and mode of birth remained largely unchanged, suggesting that a maternity care system with a strong midwife‐led primary care system may flexibly and safely adapt to external disruptions.

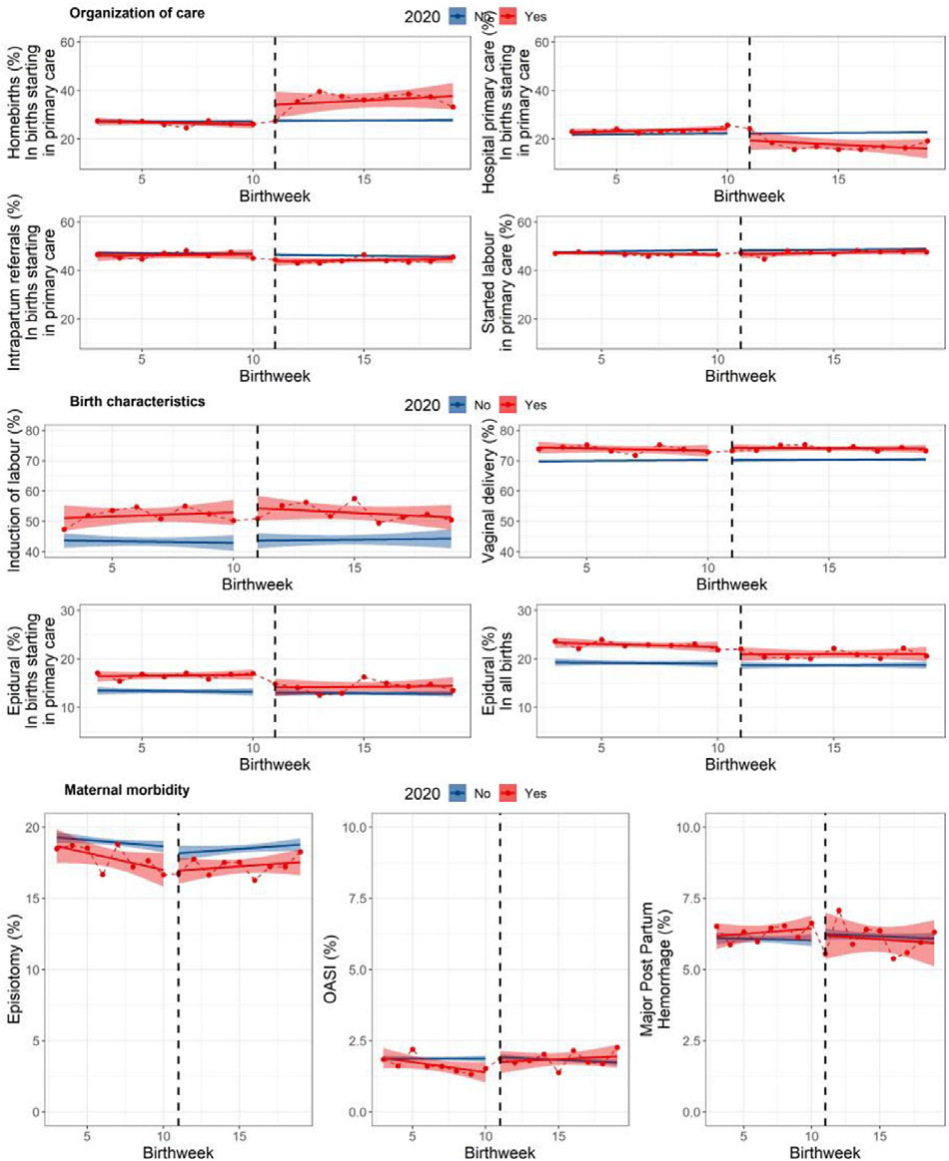

## 287 | Manifestations of placental insufficiency while under external stress–a quasi‐experimental analysis of COVID‐19 lockdown

Benjamin Y. Gravesteijn^1^, Sam Schoenmakers^2^, Nienke Boderie^3^, Thomas van den Akker^4^, Kim vanden Auweele^5^, Loes Bertens^3^, Jeroen van dillen^6^, Ank de Jonge^7^, Brenda M. M. Kazemier^8^, Jeremy Labrecque^3^, Ben W. Mol^9^, Sylvia Oberman^10^, Martijn A. Oudijk^11^, Tom van Ourti^12^, Lilian Peters^13^, Zainulareb Zohra^3^, Jasper Been^3^, Christianne J.M. de Groot^8^

^1^Amsterdam UMC, Amsterdam UMC, Noord‐Holland; ^2^Erasmus University Medical Centre, DEN HAAG; ^3^Erasmus MC, Erasmus MC, Zuid‐Holland; ^4^Leiden University Medical Center, LUMC/Leiden, Zuid‐Holland; ^5^HELLP stichting, HELLP stichting, Overijssel; ^6^Radboud UMC, Radboud UMC, Nijmegen, Gelderland; ^7^Amsterdam UMC, Amsterdam UMC, Noord‐Brabant; ^8^Amsterdam UMC, Amsterdam, Noord‐Holland; ^9^Monash University, Clayton, Victoria; ^10^Care4Neo, Care4Neo, Zuid‐Holland; ^11^Amsterdam UMC, location University of Amsterdam, Amsterdam, Noord‐Holland; ^12^Erasmus University Rotterdam, Erasmus University Rotterdam, Zuid‐Holland; ^13^UMC Groningen, UMC Groningen, Groningen

11:45–13:00

**Objective**: Increased psychosocial stress might negatively impact on placenta function and therefore increase the risk of hypertensive disorders and small‐for‐gestational‐age (SGA) in newborns, but the potential for unmeasured confounding in traditional designs hampers the evaluation of their causal effect. The COVID‐19 lockdown period can be regarded as a natural experiment to evaluate this potentially modifiable cause. This study therefore evaluates the effect of the COVID‐19 lockdown per pregnancy trimester on the occurrence of hypertensive disorders and the incidence of SGA.

**Study Design**: Singleton births of nulliparous women between 2016‐2020 in the Dutch national perinatal register above 24 weeks gestation were included. We regressed the outcomes on gestational age at march 9 (the date on which the lockdown started in 2020), allowing for interaction with the lockdown year (2020), and included year of birth to adjust for underlying long‐term time trends. We modelled non‐linearity of gestational age with restricted cubic splines.

**Results**: The analysis included 250,876 pregnancies. Entering the lockdown in the first trimester was associated with an increase in hypertension from 5.2% with 1.0 percentage points (95%CI: 0.1%‐1.9%), while entering the lockdown in the third trimester with an increase from 5.4% with 0.8% (0.1%‐1.5%). Entering the lockdown in the first trimester was also associated with more pre‐eclampsia. There was no significant association between entering the lockdown in the second trimester and hypertension, nor with entering the lockdown in any period and the incidence of SGA.

**Conclusion**: Hypertensive disorders during pregnancy increased upon exposure to lockdown in the first trimester, possibly due to maternal stress. This increase did not translate into a higher incidence of SGA babies.

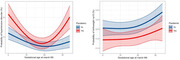

## 290 | Personal history of cancer complicating pregnancy and obstetric outcomes in the United States, 2017‐2020

Rachel K. Harrison^1^, Anna Palatnik^2^, Calla Holmgren^1^, Annie Dude^3^

^1^Advocate Health Care, Advocate Health Care, Illinois; ^2^Medical College of Wisconsin, Milwaukee, Wisconsin; ^3^University of North Carolina, Chapel Hill, North Carolina

11:45–13:00

**Objective**: To determine whether a personal history of cancer is associated with adverse obstetric outcomes.

**Study Design**: This is a retrospective review of the National Inpatient Sample (HCUP‐AHRQ), which includes a random sampling of US hospitalizations. Data for all deliveries after 20 weeks’ gestation between 2017‐2020 were included. Subjects with active cancer were excluded. A personal history of cancer was determined using ICD‐10 codes Z85.00‐Z85.99. Outcomes examined include fetal demise, fetal growth restriction, preterm delivery ( < 37 weeks, < 34 weeks, and < 28 weeks), cesarean delivery, preeclampsia, obstetric hemorrhage, and maternal venous thromboembolism. Unadjusted logistic regression and chi square analysis were used to assess the relationship between a personal history of cancer and obstetric outcomes. Adjusted logistic regression was used to account for potential confounders. Race was included in the regression model to account for the impact of racism on adverse outcomes.

**Results**: Between 2017‐2020, 10,106 deliveries occurred among patients with a personal history of cancer and 2,862,206 deliveries occurred among those without a personal history of cancer. The percentage of deliveries affected by a personal history of cancer increased over time (0.32% of all deliveries in 2017, 0.34% in 2018, 0.36% in 2019, and 0.39% in 2020, *p* < 0.001). Those with a personal history of cancer, as compared to those without, were more likely to have any preterm delivery (13.7% vs 11.4%, *p* < 0.001), preterm delivery < 34 weeks (5.1% vs 4.1%, *p* < 0.001), preterm delivery < 28 weeks (1.6% vs 1.4%, *p* = 0.015), undergo cesarean delivery (38.4% vs 32.2%, *p* < 0.001), hemorrhage (6.0% vs 4.6%, *p* < 0.001), and develop venous thromboembolism (0.22% vs 0.09%, *p* < 0.001). These associations remained significant when adjusting for confounders (Table).

**Conclusion**: Any personal history of cancer is associated with adverse obstetric outcomes in this nationally–representative sample of deliveries after 20 weeks’ gestation.

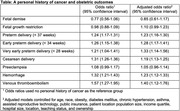

## 291 | Prevalence of cancer complicating pregnancy and obstetric outcomes in the United States from 2017‐2020

Rachel K. Harrison^1^, Annie Dude^2^, Calla Holmgren^1^, Anna Palatnik^3^

^1^Advocate Health Care, Advocate Health Care, Illinois; ^2^University of North Carolina, Chapel Hill, North Carolina; ^3^Medical College of Wisconsin, Milwaukee, Wisconsin

11:45–13:00

**Objective**: To evaluate the prevalence of cancer diagnosis (active malignancy and personal history of cancer) in a gravid population in the U.S. across 2017‐2020 and assess obstetric outcomes in these patients.

**Study Design**: Retrospective review of the National Inpatient Sample database (HCUP‐AHRQ), an ICD‐10 based random sampling of U.S. hospitalizations from 2017‐2020. We determined the number of cancer diagnoses (active malignancy and/or personal history of cancer) in pregnant patients greater than 20 weeks gestation with delivery hospitalizations by year. Those with cancer diagnoses complicating pregnancy were analyzed by year in four different groups (2017, 2018, 2019 and 2020), with 2017 serving as the historical comparison group. The association between cancer complicating pregnancy and obstetric outcomes was analyzed using unadjusted and adjusted logistic regression models.

**Results**: Among over 2.8 million delivery hospitalizations, 12,340 had an ICD‐10 code for cancer diagnosis between 2017‐2020. The rate of active malignancy remained stable from 2017 (0.07%) to 2020 (0.08%) (*p* = 0.071), whereas personal history of cancer increased slightly from 2017 (0.32%) to 2020 (0.39%) (*p* < 0.001). The rate of preterm delivery (PTD) < 37 weeks among patients with cancer diagnosis decreased from 17.4% in 2017 to 14.0% in 2020 (*p* < 0.001). Similarly, the rate of PTD at < 34 and < 28 weeks was lower in 2019 and 2020 compared to 2017 (*p* < 0.001). After adjusting for age, tobacco use, hospital location, hospital settings, and active versus personal history of cancer, the risk of PTD during the years 2019 (adjusted OR 0.83; 95% CI 0.72‐0.96 for < 37 weeks) and 2020 (adjusted OR 0.76; 95% CI 0.66‐0.88) remained lower compared to 2017 (Table). These findings persisted in adjusted analysis with PTD < 37 weeks, < 34 weeks, and < 28 weeks (Table).

**Conclusion**: The percentage of delivery hospitalizations with active cancer diagnosis was unchanged between 2017‐2020, while personal history of cancer increased slightly per year. Preterm delivery rates decreased over the years among pregnant patients with cancer diagnosis.

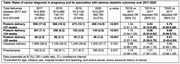

## 292 | Blood Brain Barrier Dysfunction in a rat model of preeclampsia

Owen T. Herrock^1^, Valentina Bucher^1^, Joakim Ek^2^, Catherine Anne. Cluver^3^, Lina Bergman^4^

^1^Department of Obstetrics and Gynecology, Institute of Clinical Sciences, Sahlgrenska Academy, University of Gothenburg, Gothenburg, Vastra Gotaland; ^2^University of Gothenburg, Physiology and Neurobiology, University of Gothenburg, Vastra Gotaland; ^3^Stellenbosch University, Stellenbosch University, Western Cape; ^4^Department of Obstetrics and Gynecology, Sahlgrenska Academy, University of Gothenburg, Gothenburg, Vastra Gotaland

11:45–13:00

**Objective**: Cerebral complications to preeclampsia are among the most common causes to maternal morbidity and mortality. The underlying pathophysiology remains largely unknown and is challenging to study in humans. Animal models can provide insight in how the brain is affected in preeclampsia. We hypothesized that the reduced uterine perfusion pressure (RUPP) model of preeclampsia leads to increased blood brain barrier (BBB) permeability and cerebral edema, which would make is suitable as a model for cerebral complications in preeclampsia. To describe changes in BBB integrity in specific brain regions and presence of cerebral edema in the RUPP model.

**Study Design**: 20 rats underwent either RUPP or Sham surgery on gestational day 14 followed by carotid catheterization on gestational day 19. On gestational day 20, blood pressure was measured and cerebrospinal fluid (CSF) was collected and later analysed for concentrations of claudin‐5 as a marker for BBB integrity. At termination, brains were collected and weighted as a measure of cerebral edema. A separate cohort of rats underwent the same procedures, followed by intraperitoneal injection of 14C‐sucrose (342 Da) which was then measured in brain regions by liquid scintillation to investigate regional BBB disruption.

**Results**: The RUPP procedure induced hypertension and increased brain wet weight, compared to Sham controls. This was accompanied by an increased in 14C‐sucrose accumulation in the cortex, hippocampus, striatum, cerebellum, and CSF compared to controls. Lastly, RUPP rats had a trending increase in claudin‐5 in the CSF.

**Conclusion**: Together these data suggest that placental ischemia in the RUPP model induces increased blood brain barrier permeability in a majority of brain regions and cerebral edema, potentially through dysfunction of tight junctionproteins. These findings suggest that the RUPP model can be used to investigate cerebral complications and new neuroprotective treatments in preeclampsia.

## 293 | Assessment of maternal characteristics, PlGF, and UtA PI: Contrasting FGR and non‐FGR cases in Indonesia

Amanda S D. Hertika^1^

^1^Indonesian Prenatal Institute, South Jakarta, Jakarta Raya

11:45–13:00

**Objective**: Foetal growth restriction (FGR) presents long‐term consequences with no known reversal therapy, emphasising the significance of early detection and prompt management. Yet, research on FGR diagnosis in Indonesia remains inadequate. This study aims to characterise FGR versus non‐FGR foetuses concerning maternal factors, biomarkers, and Doppler measurements.

**Study Design**: A prospective study spanning from 2019 to 2023 across two maternal health centres in Jakarta, Indonesia, collected data at the first trimester visit and recorded birth weights at delivery.

**Results**: Among 1311 deliveries, 43 (3.3%) neonates were FGR, and 103 (7.9%) were prenatally diagnosed as small‐for‐gestational‐age (SGA). Maternal conditions included diabetes mellitus, chronic hypertension, systemic lupus erythematosus, and antiphospholipid syndrome. No difference in the family history of PE was noted between FGR and non‐FGR groups. While 567 (43,2%) women had a BMI of ≥25.0 kg/m^2^, BMI did not vary between groups. PlGF levels (*p* = 0.01, 95% CI; PlGF MoM: *p* = 0.03, 95% CI) and UtA pulsatility index (PI) (*p* =  < 0.001, 95% CI) were significantly different. Fetal sex did not influence FGR or SGA incidences, but PlGF levels differed between sexes (PlGF: *p* = 0.011, 95% CI; PlGF MoM: *p* =  < 0.001, 95% CI).

**Conclusion**: First trimester PlGF and UtA PI can be helpful in distinguishing FGR and non‐FGR cases.

## 294 | Inequalities in preterm birth in England: A national cohort study focusing on deprivation and ethnicity

Iona Hindes^1^, Jennifer Jardine^2^, Dominik Zenner^2^, Stamatina Iliodromiti^1^

^1^Queen Mary University of London, Queen Mary University of london/London, England; ^2^Queen Mary University London, Queen Mary University of London/London, England

11:45–13:00

**Objective**: This study aimed to quantify socioeconomic and ethnic inequalities in preterm birth rates using routinely collected maternity data in England from 2018‐2021, to identify any changes to subgroup rates which may have arisen during a period where preterm birth rates decreased overall.

**Study Design**: Retrospective analysis of cohort data from the NHS England Hospital Episode Statistics (HES). All women aged 13‐55 with a singleton livebirth (April 2018 to March 2021) at 24‐42 weeks were included. The primary outcome was preterm birth defined as livebirth between 24+0 and 36+6 weeks’.

Multivariate Poisson regression was used to estimate rate of preterm birth for each ethnic and deprivation postcode group, these were compared to white women from the least deprived 20% of areas to obtain relative rate ratios. We iteratively developed the model, adjusting for covariates associated with preterm birth. A post‐hoc calculation identified the rate of preterm birth for each ethnic group at each level of deprivation.

**Results**: There were 1,111,045 livebirths between April 2018 and March 2021. The rate of preterm birth increased with socioeconomic deprivation and was highest at 7.1% per 100 livebirths (95%CI:7.00‐7.20) in women living in the 20% most deprived areas, compared to 5.6% (95%CI:5.50‐5.74) in women in the 20% least deprived areas. White women had the lowest preterm birth rate at 6.5% (95%CI:6.47‐6.58) and South Asian women the highest rate at 6.9% (95%CI:6.76‐7.04) (Table 2). The rate of preterm birth increased according to increased area‐level deprivation across all ethnicity groups, although in the least deprived areas, minority ethnicity groups had higher rates compared to White and Other ethnicity women.

**Conclusion**: Inequalities in preterm birth remain, despite overall decreases in preterm birth. The preterm birth rate was similar across ethnicity groups in areas of high deprivation, whereas in less deprived areas inequalities based on ethnicity remained. Higher area‐level deprivation appears to interact with ethnicity and is a key predictor of preterm birth.

## 295 | Analysis the regeneration efficacy of Prolactin in the amniotic epithelia in vitro

Soon Cheol Hong^1^, Deqi Kong^2^, Soowon Hwang^2^, Hee Ryun Cho^2^, Yu‐Jin Song^2^, Young Mi Jung^3^, Dong Ho Geum^2^, Ho‐Yeon Kim^2^, Min‐Jeong Oh^3^, Ki Hoon Ahn^2^

^1^Korea University, Korea University, Seoul‐t'ukpyolsi; ^2^Korea University Medicine, Seoul, Seoul‐t'ukpyolsi; ^3^Korea University College of Medicine, Seoul, Seoul‐t'ukpyolsi

11:45–13:00

**Objective**: To evaluate the regeneration ability of prolactin (PRL) in the amniotic epithelial cells (hAESCs) in vitro

**Study Design**: Intact human amnion membranes were collected from patients underwent cesarean section for full‐term pregnancies without any complications with written informed consent. The hAESCs were treated with various concentrations of PRL, and the proliferation ability were measured by CCK‐8 kit and Ki67 staining. Expression of apoptosis related genes were measured via real‐time PCR.

**Results**: The hAESCs treated with PRL from low concentration 10 ng/mL to high concentration 500 ng/mL all exerted good morphology and proliferation ability. Via CCK‐8 proliferation kit, PRL 500 ng/mL treatment group exerted accelerated proliferation ability significantly. In addition, the proportion of Ki‐67 positive cells in the PRL treatment group was significantly enhanced. Apoptosis‐related genes were also analyzed, and the results indicated that the expression of Caspase 3, Caspase 8, and Bax were down regulated after PRL treatment, while the expression of Bcl‐xl was up‐regulated in hAESCs.

**Conclusion**: Prolactin enhances proliferation ability in the amniotic epithelial cells (hAESCs) in vitro.

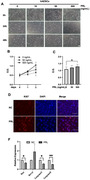

## 296 | Comparing proliferation in culture media with HSA, SSS, and FBS in Amniotic Epithelial Stem Cells

Soon Cheol Hong^1^, Hee Ryun Cho^2^, Deqi Kong^2^, Soowon Hwang^2^, Yu‐Jin Song^2^, Sung Hoon Hong^2^, Yunbae Kim^3^, Young Mi Jung^4^, Dong Ho Geum^2^, Ho‐Yeon Kim^2^, Ki Hoon Ahn^2^, Min‐Jeong Oh^4^, Geum Joon Cho^4^

^1^Korea University, Korea University, Seoul‐t'ukpyolsi; ^2^Korea University Medicine, Seoul, Seoul‐t'ukpyolsi; ^3^Chungbuk National University, Cheongju, Ch'ungch'ong‐bukto; ^4^Korea University College of Medicine, Seoul, Seoul‐t'ukpyolsi

11:45–13:00

**Objective**: Human amniotic epithelial stem cells(hAESCs) are promising in regenerative medicine. Fetal Bovine Serum(FBS) poses ethical, quality, and physiological challenges during cultivation. Human Serum Albumin(HSA) and Serum Substitute Supplement(SSS) offer safe, animal‐free alternatives, addressing FBS‐related issues and potentially boosting cell proliferation. This study assesses HSA and SSS effects on hAESCs proliferation compared to FBS, exploring substitution potential.

**Study Design**: Amnion was collected from women during cesarean section with informed consent provided. The culture medium was formulated by substituting 10% FBS with 10% HSA or 20% SSS in DME/F‐12 medium containing 1% penicillin‐streptomycin and 10ng/mL epidermal growth factor(EGF). Cell state, morphology, and proliferation were observed under a microscope at magnifications of 4x and 20x. Cell count was determined using Trypan Blue staining. Additionally, hAESCs proliferation was quantified via CCK‐8 Assay and BrdU expression. Furthermore, CD324(E‐cadherin) and other key stem cell markers(CD19, CD34, CD90, CD105, Oct3/4, Sox2, SSEA4) were examined to characterize the cells.

**Results**: When using HSA instead of FBS, the cells assumed a relatively round, small shape, and growth was slower. Gradually, they began to form elongated shapes, indicating faster differentiation pattern. When substituting SSS for FBS, the cells displayed an intermediate morphology, more similar to FBS, and exhibited a comparable growth rate. Proliferation in FBS and SSS showed a statistically significant increase, however, in HSA showed no significance. Also, overall values of HSA were significantly lower than those in the other two media. The expression of BrdU and stem cell markers was confirmed, notably demonstrating expression of epithelial and embryonic stem cell markers.

**Conclusion**: When comparing proliferation using SSS and HSA as substitutes for FBS, SSS presents itself as a more promising alternative in cell proliferation. However, our findings indicate that FBS continues to demonstrate superior effectiveness, so further validation is required.

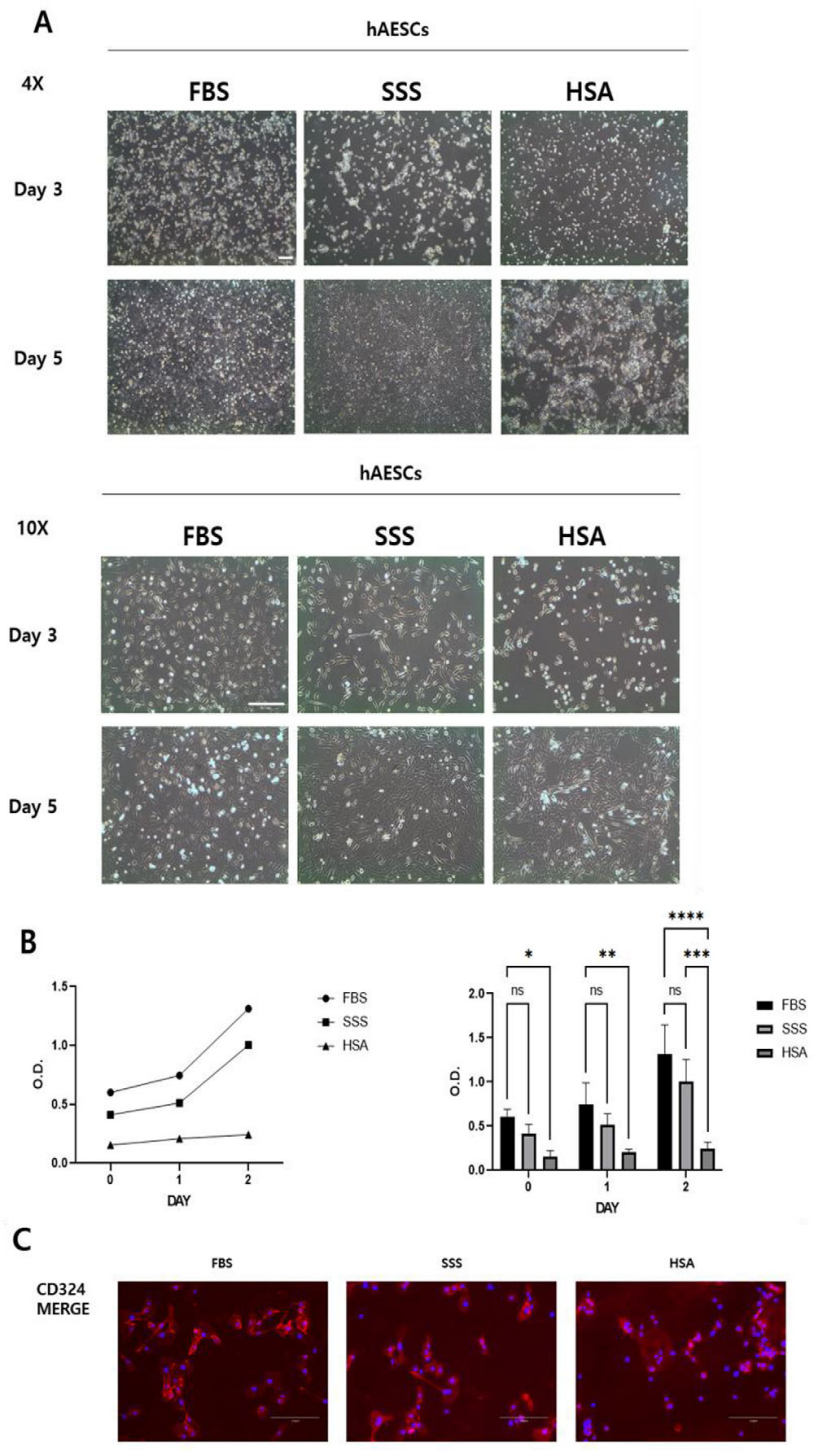

## 297 | Clinical evaluation of a new cut‐off in fetal blood sampling measuring lactate

Sara Norrestam^1^, Ylva Carlsson^2^, Linda Iorizzo^3^

^1^Department of Obstetrics and gynecology Helsingborg Hospital, Helsingborg, Skane Lan; ^2^Department of Obstetrics and Gynaecology, Region Vastra Gotaland, Sahlgrenska University Hospital, Gothenburg, Department of Obstetrics and Gynecology, Sahlgrenska University Hospital, Gothenburg, Vastra Gotaland; ^3^Department of Obstetrics and gynecology Lund, Skane University Hospital, Skane University Hospital, Lund, Skane Lan

11:45–13:00

**Objective**: The use of Fetal blood sampling (FBS) during labor as adjunctive tool to cardiotocography (CTG) to intervene appropriately is debated. Simultaneously, there is a concerning global increase in the rate of caesarean sections. FBS lactate (FBS‐L) is an attractive alternative to FBS pH since it can differentiate between early metabolic and respiratory acidosis with a much higher sampling success rate of ≈99%. A new cut‐off for FBS‐L for intervention of 5.2mmol/L was proposed based on a large cohort study and for the first time showed a high predictive value (AUC 0.87) for metabolic acidosis(MA) in umbilical cord blood at birth. The objective of this study was to assess the clinical use of the new FBS‐L cut‐off in a large delivery unit.

**Study Design**: Prospective observational study. February 2022–January 2023, in Helsingborg, Sweden. Inclusion criteria: indication for FBS, singleton pregnancy, vertex presentation, ≥35+0 weeks of gestation. FBS measured bedside with Statstrip®Lactate meter (0.6µl of capillary blood and testtime13 seconds). Data was collected from the lactate meter, electronic files, and analyzes of umb cord blood (ABL800). Main outcome measures were pH < 7.0 and MA in umbilical blood, the latter defined as pH < 7.05+BD_ecf_> 10mmol/l or lactate > 10mmol/l.

**Results**: Out of 3070 planned vaginal labors, FBS‐L was used in 17.2%(529). In the initial FBS‐L measurement 90.4% showed normal values. The negative predictive value was 100% for pH < 7.0 and pH < 7.05+BD_ecf_ ≥ 12mmol/l in umb cord blood. For FBS‐L samples analyzed within 25 minutes, there was one case of MA with FBS‐L below the cut‐off of 4.1mmol/l. For MA and FBS‐L within 25 minutes from birth the sensitivity, specificity, and negative predictive value were 80%, 71.1% and 99.3% respectively. Emergency caesarean section was performed in 9.6%(294) in the entire cohort.

**Conclusion**: In cases of non‐reassuring intrapartal CTG, FBS lactate values < 5.2mmol/l, measured with Statstrip®Lactate, can effectively rule out pH < 7.0 in umbilical cord blood at birth to allow labor to progress safely.

## 298 | A new perspective on the mechanism and prediction of atonic postpartum hemorrhage from immunological standpoint

Hai Jiang^1^, Nana Huang^1^, Jiangxue Qu^1^, Yangyu zhao^2^

^1^Peking University Third Hospital, Peking University Third Hospital, Beijing; ^2^Obstetrics & Gynecology Department, Peking University Third Hospital, Beijing, Beijing

11:45–13:00

**Objective**: To examine changes in maternal plasma cytokines in women with atonic postpartum hemorrhage (PPH). Dissecting the maternal‐fetal interface molecular profiles of atonic postpartum hemorrhage by single‐cell RNA sequencing.

**Study Design**: This was a retrospective longitudinal case‐control study of pregnant women with singleton gestations at term undergoing vaginal delivery. Cases were women with atonic PPH, and 1:1 propensity‐score matching was used to match the control group. Maternal plasma was collected in the first trimester, early third trimester, and late third trimester, and multiplex Luminex assay was used to determine the cytokine concentrations. Multivariate logistic regressions were used to determine the association between maternal cytokines at different stages of pregnancy and atonic PPH. Using a single‐cell RNA‐sequencing technique, we dissected the maternal‐fetal interface molecular profiles of atonic PPH.

**Results**: In the late third trimester, higher plasma concentrations of 12 cytokines were significantly associated with increased risk of atonic PPH. The prediction model of atonic PPH constructed by Basic FGF, MIP‐1α, and SCF had a high predictive efficiency. There were significant differences in tissue distribution and polarization status of myeloid cells in atonic PPH. M1‐type polarization signal was dominant. The function of differential expressed genes was closely related to inflammatory response and cytokine secretion. The expression of inflammasome related genes was significantly up‐regulated in vaginal blood myeloid cells. The pyroptosis ratio of macrophages and the expression level of inflammasome‐related proteins in placental tissues of patients with atonic PPH were significantly increased.

**Conclusion**: Higher concentrations of maternal plasma inflammatory cytokines before onset of labor are associated with high risk of subsequent atonic PPH. Abnormal activation of the NLRP3 inflammasome pathway in the local maternal‐fetal interface and the polarization of M1‐type macrophages can induce the pyroptosis of macrophages and form a pro‐inflammatory immune microenvironment in atonic PPH.

## 299 | Metabolic dysfunction‐associated steatotic liver disease increases the risk of adverse pregnancy outcomes: A nationwide study

Young Mi Jung^1^, Wonyoung Wi^2^, Seung Mi Lee^3^, Min‐Jeong Oh^1^, Young‐Han Kim^4^, Won Kim^5^, Geum Joon Cho^1^

^1^Korea University College of Medicine, Seoul, Seoul‐t'ukpyolsi; ^2^Korea University College of Medicine/Seoul, Korea University College of Medicine/Seoul, Seoul‐t'ukpyolsi; ^3^Seoul National University College of Medicine, Seoul, Seoul‐t'ukpyolsi; ^4^Yonsei University College of Medicine/Seoul, Yonsei University College of Medicine/Seoul, Seoul‐t'ukpyolsi; ^5^Seoul National University College of Medicine/Seoul, Seoul National University College of Medicine/Seoul, Seoul‐t'ukpyolsi

11:45–13:00

**Objective**: A recent consensus group, supported by international liver societies, has introduced a classification system that delineates distinct subcategories within the broader term of steatotic liver disease (SLD). The objective of this study was to analyze the impact of steatotic liver disease on adverse pregnancy outcomes (APO), following the recently revised criteria.

**Study Design**: This national data study was conducted among women who had received health check‐ups within one year of pregnancy and subsequently gave birth to singletons. Participants were classified into three groups: those without SLD, those with metabolic dysfunction‐associated steatotic liver disease (MASLD), and those with MASLD accompanied by increased alcohol intake (MetALD). The risk of APO was compared among these three groups.

**Results**: A total of 83,452 women were ultimately included in the analysis. When compared to women without SLD, women with MASLD (aOR 2.02, 95% CI 1.86–2.21) and women with MetALD (aOR 2.31, 95% CI 1.97–2.70) had a higher risk of adverse pregnancy outcomes. These trends were observed in gestational diabetes (GDM), hypertensive disorders of pregnancy, and preterm birth; however, there was no significant difference in low birthweight. Additionally, when comparing the MASLD and MetALD groups, only the risk of GDM showed marginal significance (*p* = 0.09). Furthermore, the risk of APO increased according to the number of cardiometabolic risk factors (CMRF).

**Conclusion**: Both MASLD and MetALD increase the risk of APO, and this elevated risk is further exacerbated with an increasing number of CMRFs. Women with pre‐existing SLD before pregnancy require more vigilant management during pregnancy, especially when there are more CMRFs, in order to prevent the occurrence of APO.

## 300 | The impact of the COVID‐19 on pre‐pregnancy BMI and biometric changes in pregnant women

Young Mi Jung^1^, Heechul Jeong^2^, Min‐Jeong Oh^1^, Young‐Han Kim^3^, Geum Joon Cho^1^

^1^Korea University College of Medicine, Seoul, Seoul‐t'ukpyolsi; ^2^Department of Statistics, Sungkyunkwan University, Sungkyunkwan University, Seoul‐t'ukpyolsi; ^3^Yonsei University College of Medicine/Seoul, Yonsei University College of Medicine/Seoul, Seoul‐t'ukpyolsi

11:45–13:00

**Objective**: The outbreak of COVID‐19 has significantly impacted daily life and physical activity, especially affecting pregnant women in terms of their exercise, outdoor activities, and diet. While many studies have reported increases in adverse pregnancy outcomes following the outbreak, research on the changes in body mass index(BMI), waist circumference(WC), and obesity which significantly influence pregnancy complications, is lacking. The purpose of this study was to examine the changes in pre‐pregnancy biometrics and obesity before and after COVID‐19.

**Study Design**: This retrospective nationwide cohort study included singleton pregnant women who underwent national health screening exam within one year of pregnancy between 2015 and 2022. We divided the study period into pre‐pandemic and pandemic eras, using 2020 as the dividing line, to assess changes in the pre‐pregnancy BMI, WC, and obesity rates among these women. In our analysis, we adjusted for maternal age, gestational age, and the sex of the offspring, employing an interrupted time series design that accounts for underlying temporal trends.

**Results**: Among the total cohort of 494,111 participants, 438,978 belonged to the pre‐pandemic period and 55,133 to the pandemic period. The average BMI during the pre‐pandemic period was 21.6, while during the pandemic, it was 22.2. Additionally, the rate of obesity was 2.4% in the pre‐pandemic period and increased to 3.6% during the pandemic. After adjusting for maternal age, gestational age at delivery, and the offspring's sex, the odds ratio for pre‐pregnancy obesity during the pandemic compared to the pre‐pandemic period was found to be 1.44 (95% confidence interval 1.37‐1.51).

**Conclusion**: It was observed that among pregnant women, pre‐pregnancy BMI and WC increased after the pandemic, and the rate of obesity also increased. Given that BMI, WC, and obesity are significant factors influencing the occurrence of adverse pregnancy outcomes, further research is needed.

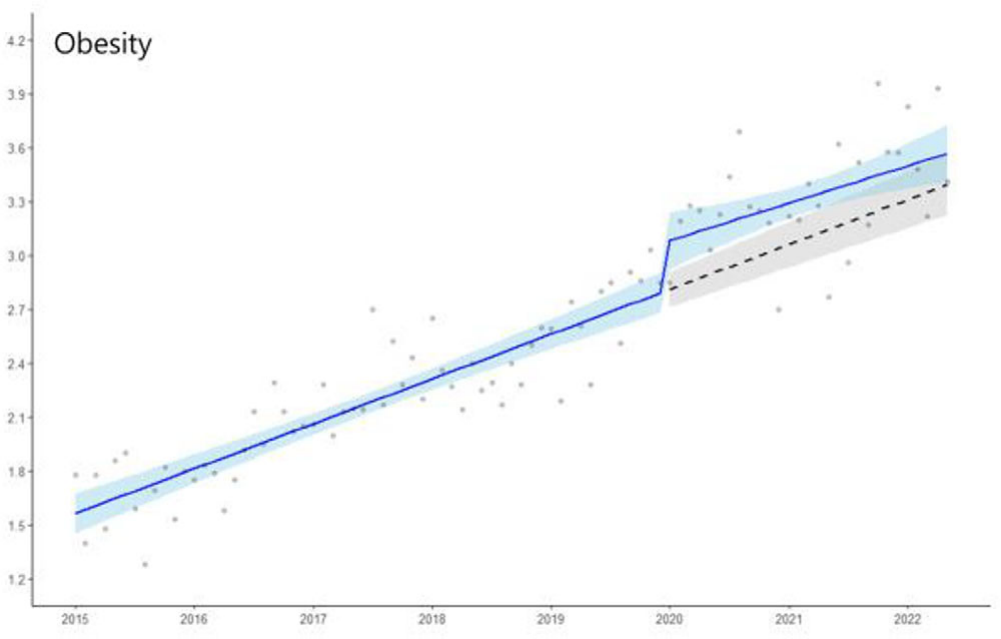

## 301 | Does the new definition for fetal growth restriction (FGR) affect mode of delivery?

Keren Khromchenko^1^, Fatima Ali^2^, Jennifer E. Powel^1^, Noelle Aikman^2^, Joseph Canterino^1^, Karen Koscica^1^, Valeria Distefano^1^, Sheveta Jain^1^, Maria Martins^1^, Jonathan Faro^3^

^1^Hackensack Meridian Health–Jersey Shore University Medical Center, Neptune, New Jersey; ^2^Hackensack Meridian Health–Jersey Shore University Medical Center, Neptune, New Jersey; ^3^Jersey Shore University Medical Center/Hackensack Meridian School of Medicine, Marlboro, New Jersey

11:45–13:00

**Objective**: Recently, the Society of Maternal Fetal Medicine and the American College of Obstetricians and Gynecologists redefined fetal growth restriction (FGR) from estimated fetal weight (EFW) < 10th percentile to EFW or abdominal circumference (AC) < 10th percentile. The objective of the study is to assess the difference in rates of operative or cesarean delivery in FGR diagnosed by isolated AC < 10th percentile (iAC group) versus those diagnosed by EFW < 10th percentile (EFW group).

**Study Design**: A retrospective cohort study on all singleton, non‐anomalous pregnancies diagnosed with FGR between October 2019 to December 2021 was performed. Patients who underwent cesarean section or operative delivery for indications unrelated to abnormal fetal heart rate tracings were excluded. Patients were divided based on criteria at time of initial diagnosis of FGR. The iAC group included patients diagnosed due to AC < 10th percentile and EFW ≥ 10th percentile. The EFW group included patients with EFW < 10th percentile regardless of AC percentile.

The primary outcome is the rate of cesarean section or operative delivery due to category II/III tracing.

**Results**: The iAC group had a rate of cesarean section or operative delivery of 10.4% (19/182) as compared to 15.2% (45/297) in the EFW group (*p* = 0.17).

The average gestational age at diagnosis was 33.84 weeks in the iAC group and 31.85 weeks in the EFW group (*p* < 0.001).

**Conclusion**: The rate of cesarean section or operative delivery in patients with isolated AC less than the 10th percentile at first diagnosis of FGR is less than those diagnosed based on EFW, however our results did not reach statistical significance.

## 302 | How good are chatbots at explaining soft ultrasound markers for aneuploidy?

Keren Khromchenko^1^, Jonathan D. Baum^1^, Joseph Canterino^1^, Jonathan Faro^2^, Valeria Distefano^1^, Karen Koscica^1^, Sheveta Jain^1^, Jennifer E. Powel^1^, Maria Martins^1^

^1^Hackensack Meridian Health–Jersey Shore University Medical Center, Neptune, New Jersey; ^2^Jersey Shore University Medical Center/Hackensack Meridian School of Medicine, Marlboro, New Jersey

11:45–13:00

**Objective**: Evaluate the performance of the most commonly used chatbots ChatGPT (GPT) and Google Bard (Bard) as sources of information on the subject of soft markers for aneuploidy in the second trimester.

**Study Design**: A qualitative analysis was performed. ChatGPT Version 3.5 and Google Bard were queried on 8 isolated soft markers listed in Society for Maternal‐Fetal Medicine (MFM) Consult Series #57: *Evaluation and management of isolated soft ultrasound markers for aneuploidy in the second trimester*. Each query was conducted and recorded from September 20‐25, 2023. Query responses were graded as “acceptable” or “not acceptable” based on correctness and completeness in comparison to SMFM publications, other evidence‐based references, and clinical experience. The reason for an answer being deemed “not acceptable” was also evaluated and recorded. Review and grading of responses were performed by the MFM co‐authors individually and then as a group to formulate a consensus.

**Results**: A grade of “not acceptable” was assigned to 75% of GPT and 87.5% of Bard responses. For GPT, 3 responses were incomplete, 5 were incorrect, and 2 were both incomplete and incorrect. For Bard, 3 responses were incomplete, 5 were incorrect, and 1 was both incomplete and incorrect.

**Conclusion**: Chatbot performance when queried on isolated soft ultrasound markers for aneuploidy was disappointing. The majority of responses by ChatGPT and Google Bard were incorrect, incomplete, or both. The use of chatbots for explanations of these ultrasound findings warrants further evaluation prior to being accepted as accurate and reliable for this purpose.

## 303 | Is mode of delivery associated with method of induction in term first births?

Lisa T. Leeves^1^, Nina Gunnes^2^, Anne Flem Jacobsen^3^, Trond Melbye Michelsen^3^, Ingvil Krarup Sørbye^3^

^1^Oslo University Hospital/Univeristy of Oslo, Oslo University hospital, Oslo; ^2^Oslo University Hospital, Oslo, Oslo; ^3^Oslo University Hospital/University of Oslo, Oslo, Oslo

11:45–13:00

**Objective**: To investigate the association between method of induction and mode of delivery in a Norwegian birth cohort of a nulliparous pregnant population with a single live cephalic fetus and induction of labor at ≥ 37 weeks of gestation. We also explored whether the associations varied according to indication for induction.

**Study Design**: We used data from the Medical Birth Registry of Norway from 2020–2021. Methods of induction were grouped according to clinical sequence and practice. The primary outcome was mode of delivery classified as cesarean delivery, operative vaginal delivery, or spontaneous vaginal delivery. We applied multinomial logistic regression to estimate crude and adjusted relative risk ratios (RRRs) for cesarean delivery and operative vaginal delivery, with associated 95% confidence intervals (CIs). We did not have information on cervical status. In supplementary analyses, we assessed whether the relative risks varied by indication for induction of labor.

**Results**: Out of 110,640 births, 12,992 (11.7%) met the inclusion criteria. Balloon catheter with consecutive prostaglandin (± amniotomy/oxytocin) were used among 38.5%, whilst 30.5% were induced with balloon catheter (± amniotomy/oxytocin), 21.6% with prostaglandins (± amniotomy/oxytocin), and 9.7% with amniotomy and/or oxytocin only. Overall, 21.4% had a cesarean delivery, 21.6% had an operative vaginal delivery, and 57.0% had a spontaneous vaginal delivery.

The risk of cesarean delivery was reduced in the groups receiving prostaglandins (adjusted RRR [aRRR]: 0.80; 95% CI: 0.70–0.91) and amniotomy/oxytocin (aRRR: 0.72; 95% CI: 0.60–0.86), compared to the group receiving balloon and prostaglandin.

The risk of operative vaginal delivery did not seem to vary by method of induction. Risks of operative delivery and maternal and fetal safety outcomes varied by the indications for induction.

**Conclusion**: In induced term first births initiated with prostaglandins and/or amniotomy/oxytocin, we found lower risks of cesarean delivery compared to induced first births initiated with balloon catheter in this Norwegian population‐based registry study.

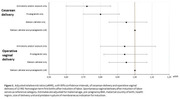

## 305 | Quantification of placental membrane transporters in gestational diabetes with normal birth weight using proteomics approach

Muhammad Pradhiki Mahindra^1^, Riccardo Zenezini Chiozzi^2^, Dimitrios Siassakos^1^, Sara Hillman^1^, Owen Vaughan^1^

^1^Elizabeth Garrett Anderson Institute for Women's Health, University College London, London, England; ^2^University College London Mass‐Spectrometry Science Technology Platform, London, England

11:45–13:00

**Objective**: We hypothesized that placental membrane transporter abundance is altered in pregnancies complicated by GDM with normal fetal growth and aimed to quantify these transporters using an unbiased proteomics approach.

**Study Design**: A cross‐sectional sampling of placental villous tissue was done at term from women with GDM (*n* = 7) and normal glucose tolerance controls (*n* = 9) and snap‐frozen after collection. GDM was diagnosed according to modified IADPSG criteria at University College London Hospital (UCLH). Placentas were sent to the UCL mass‐spectrometry facility for proteomics profiling using LC‐MS/MS. Differentially expressed proteins in GDM compared to control placentas [fold change > 0 (upregulated) or < 0 (downregulated); *p* < 0.05] were entered into Uniprot. Membrane transporter proteins were identified by filtering based on Gene Ontology terms (GO term: 0055085).

**Results**: From a total of 4176 proteins captured by mass‐spectrometry, there were fourteen significantly upregulated proteins (ABCD1, ATP6AP2, ATP5MC1, ATP5PB, ATP5PD, COX6B1, MPC1, NDUFB7, NDUFB10, ROM01, TIMM23, SLC4A2, USP9X, VDAC1) and sixteen significantly downregulated proteins (ATP1A3, RAB32, SLC1A2, DCD, ABCB9, SLC25A22, SLC16A10, SHROOM2, PLP2, FLNA, ATP6V1C1, ATP6V1B2, GP1BB, HPX, SLC16A4, and CLIC1) were identified with transmembrane transport functions according to Uniprot analysis.

**Conclusion**: In GDM with normal birth weight, there are changes in transmembrane transporter protein abundance in placental villous tissue. Alterations in the specific mixture of nutrients delivered to the fetus and placenta may contribute to adverse pregnancy outcomes, even when fetal growth is unaffected.

## 306 | Uterine Artery Pulsatility Index and adverse outcomes in pregnancies with reactive hypoglycaemia

Muhammad Pradhiki Mahindra^1^, Muhammad Pradhika Mapindra^1^, Adly Nanda Al‐Fattah^2^, Raden Aditya Kusuma^3^, Sara Hillman^1^, Dimitrios Siassakos^1^

^1^Elizabeth Garrett Anderson Institute for Women's Health, University College London, London, England; ^2^Indonesian Prenatal Institute, Jakarta, Jakarta Raya; ^3^Harapan Kita National Women and Children Hospital, Jakarta, Jakarta Raya

11:45–13:00

**Objective**: Although there are no agreements on the appropriate cut‐off value to diagnose the condition, glucose values lower than 4 mmol/l during an oral glucose tolerance test (OGTT) in pregnancy may cause adverse fetal‐neonatal events. This study aims to investigate first‐trimester UtA‐PI and outcomes in pregnancies with reactive hypoglycaemia (RH) diagnosed as a value under 4 mmol/l.

**Study Design**: Retrospective Cohort Study of pregnant women underwent an OGTT after 24 gestational weeks between 2021 and 2023 in maternity units in Indonesia. Women with multifetal pregnancy, pre‐pregnancy diabetes, chronic hypertension, and fetal anomalies were excluded. Gestational diabetes mellitus (GDM) was diagnosed according to IADPSG criteria. RH was defined as 2‐hour OGTT values < 4 mmol/l/.

**Results**: From a total of 284 participants who underwent OGTT, 10 women had RH whilst GDM was diagnosed in 38 women. Women with RH had a significantly higher mean uterine artery pulsatility index (UtA‐PI) than women with normal OGTT group (2.24 ± 0.26 vs 1.62 ± 0.45, *p* < 0.05) whereas GDM did not. Compared to the normal OGTT, the RH group had higher incidence of oligohydramnios (30% vs 4.7%, *p* < 0.05), neonatal intensive care unit (NICU) admission (50% vs 3%, *p* < 0.05), preterm prelabour rupture of membrane (PPROM) (40% vs 13.2%, *p* < 0.05), and preterm birth (30% vs 1.7%, *p* < 0.05). Women with GDM, also had higher incidence of PPROM (20.5% vs 13.2%, *p* < 0.05) and preterm birth (10.3% vs 1.7%, *p* < 0.05) than controls, but not as high as women with RH.

**Conclusion**: RH may be another clinical presentation of glucose dysmetabolism in pregnancy, prone to similar adverse outcomes as GDM but with higher frequency in the absence of treatment. Moreover, women with RH showed a higher uterine artery resistance than controls which implies that the adverse outcomes, including preterm birth, may be placenta‐driven.

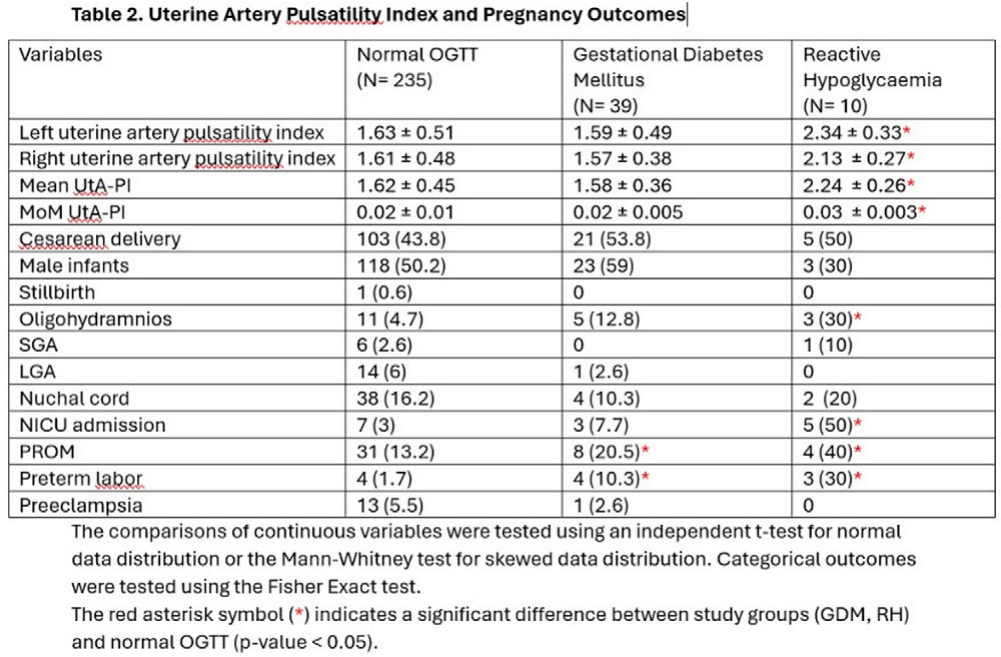

## 307 | Do secundiparous women who undergo VBAC have a different partogram than primiparous who deliver vaginally?

Maayan Maor^1^, Emmanuel Attali^2^, Yariv Yogev^3^, Yoav Baruch^4^

^1^Lis Maternity and Women Hospital, Tel Aviv Sourasky Medical Center, Tel Aviv University, Israel., Lis Maternity and Women Hospital, Tel Aviv Sourasky Medical Center, Tel Aviv University, Tel Aviv; ^2^Lis Hospital for Women's Health, Tel Aviv Sourasky Medical Center, Tel‐Aviv, Tel Aviv; ^3^Lis Maternity Hospital, Sourasky Medical Center, Tel Aviv University, Tel Aviv Sourasky Medical Center, Tel Aviv; ^4^Lis Maternity Hospital, Sourasky Medical Center, Tel Aviv University, Israel, Tel Aviv, Tel Aviv

11:45–13:00

**Objective**: To compare the progression of labor between secundiparous at their first vaginal birth after cesarean section (fVBAC) to primiparous who delivered vaginally.

**Study Design**: A retrospective cohort study in a tertiary university‐affiliated medical center with approximately 12,500 deliveries annually (January 2011‐January 2021). Eligibility was limited to maternal age > 18, singleton pregnancy at term (37‐41 weeks) in vertex presentation. Only women with spontaneous onset of delivery who delivered in vaginal birth (no instrumental delivery or augmentation of labor) were enrolled for this analysis. Active phase of delivery was defined at 3‐6 cm with the highest value preferred.

**Results**:

1. During the study period, 13,983 primiparous and 736 women after fVBAC met the inclusion criteria.
2. Participants from the fVBAC group were older, had higher rates of epidural analgesia, and mean birth‐weight (Table).
3. The rate of progression of vaginal dilatation in the active phase (cm/h) was significantly higher in the fVBAC group (3.26 vs. 2.85, *p* = 0.011).
4. Furthermore, women in the fVBAC group had a significantly shorter second stage of labor (77.8 vs. 86.6 min, *p* < 0.001).
5. The rate of prolonged second stage was higher in the primiparous group (9.5% vs. 7.1%, *p* = 0.029).
6. In a multivariable logistic regression to study the association between fVBAC and prolonged second stage, fVBAC was found to be inversely correlated with prolonged second stage (OR 0.541, CI 95% 0.388‐0.753, *p* = 0.001).

**Conclusion**: When compared to primiparous women, women at their first vaginal birth after cesarean section had significantly shorter active phase and increased progression rate as well as a shorter second stage of labor.

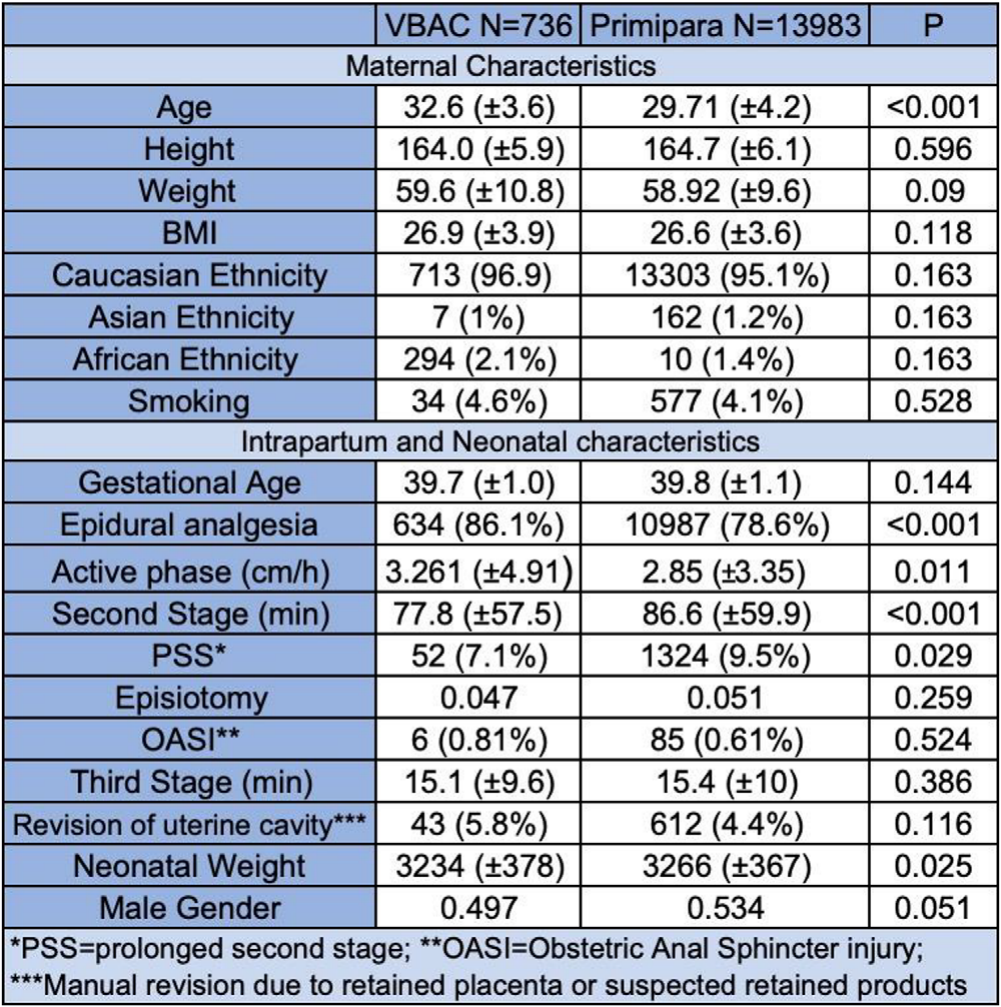

## 308 | Cervical stiffness in mid‐pregnancy as predictor of spontaneous preterm birth

Begoña Martinez de Tejada^1^, Irene Hösli^2^, Gundula Hebisch^3^, Michael Bajka^4^, Katharina Quack Lötscher^5^, Alice Winkler^6^, Leonhard Schäffer^7^, Monya Todesco Bernasconi^8^, Tina Fischer^9^, Vincent Uerlings^6^, Jute Richter^10^, Stephanie Von Orelli^11^, Markus Kuther^12^, Elke Prentl^13^, Ursula Fellmann^13^, Alexander Krafft^14^, Lea Köchli^15^, David Scheiner^16^

^1^University Hospitals of Geneva (HUG), Department of Pediatrics, Gynecology and Obstetrics, Geneva, Geneve; ^2^University Hospital Basel, Women's Clinic, Basel, Basel‐Stadt; ^3^Cantonal Hospital Frauenfeld, Department of Obstetrics and Gynaecology, Frauenfeld, Thurgau; ^4^University of Zurich; Practice Prof. Dr. med. Bajka Michael, Volketswil, Zurich; ^5^University Hospital of Zürich, Department of Obstetrics, Zürich, Zurich; ^6^Lucerne Cantonal Hospital, Women's Clinic, Lucerne, Luzern; ^7^Cantonal Hospital Baden AG, Clinic for Obstetrics; University of Zurich, Baden, Aargau; ^8^Cantonal Hospital Aarau, Women's Clinic, Aarau, Aargau; ^9^Cantonal Hospital St. Gallen, Women's Clinic Obstetrics, St. Gallen, Sankt Gallen; ^10^UZ Leuven, Gynecology and Obstetrics, Leuven, Vlaams‐Brabant; ^11^Municipal Hospital Zurich Triemli, Women's Clinic, Zurich, Zurich; ^12^Cantonal Hospital Münsterlingen, Clinic for Gynecology and Obstetrics, Münsterlingen, Thurgau; ^13^Cantonal Hospital Winterthur, Clinic for Obstetrics, Winterthur, Zurich; ^14^Practice PD Dr. med. Alexander Krafft, Zollikon, Zurich; ^15^Women's Health Centre Dr. med. Lea Köchli, Zurich, Zurich; ^16^University Hospital Zürich, Department of Gynecology, Zurich, Zurich

11:45–13:00

**Objective**: Preterm birth (PTB) is a global, yet unsolved problem. Cervical stiffness has the potential to improve identification of women at risk. The Pregnolia System is an aspiration‐based device measuring cervical stiffness objectively. This study assessed the cervical stiffness index (CSI) in unselected women using this device with the aim of evaluating its difference between women delivering at term (TB) and those with spontaneous preterm birth (sPTB) delivering preterm.

**Study Design**: Cross‐sectional, prospective study of 1000 pregnant women at mid‐pregnancy (18+0/7–22+0/7 weeks) conducted at 12 Swiss and 1 Belgian sites (clinicaltrials.gov: NCT02037334). All women underwent transvaginal ultrasound examination to measure cervical length (CL) and obtained CSI measurement during speculum examination.

**Results**: 1002 women (990 singleton) completed the study. The PTB rate (5.2%) was lower than expected (52 PTBs, of which 25 were spontaneous). The median CSI for women delivering at term (*n* = 950) was 71 mbar (51,97) (median; interquartile range), CSI was 65 mbar (44,70) for women with a sPTB (*n* = 25; *p* = 0.097). When analyzing women with singleton pregnancy only, CSI was 71 mbar (51,97) for TB (*n* = 948) and 65 mbar (42,70) for sPTB (*n* = 19; *p* = 0.039). Women with no history of vaginal delivery (VD) (*n* = 590) and a TB had a significantly stiffer cervix (75 mbar (55,103)) than those with a previous VD (*n* = 358; 64 mbar (45,85); *p* < 0.001). In the sub‐population without history of VD, the difference in CSI between women with a TB (75 mbar (55,103)) and women with a sPTB (*n* = 15; 59 mbar (42,70)) was more significant (*p* = 0.007). CSI showed a prediction improvement over CL: area under the curve (AUC) 0.638 for CSI vs AUC 0.617 for CL for all women with a singleton pregnancy and AUC 0.703 for CSI vs AUC 0.592 for CL for the sub‐population without history of VD.

**Conclusion**: Cervical stiffness was significantly lower in women with singleton pregnancy with sPTB compared to women with TB. The difference was stronger in the sub‐group without history of vaginal delivery and CSI performed better than the measurement of CL by ultrasound.

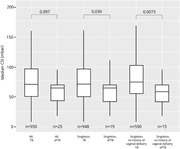

## 309 | Impact of Induction of Labor indications on obstetric and neonatal outcomes in nulliparous women

Gal cohen^1^, Maya Gat^2^, Jill Savren^2^, Michal Mor^2^, Hanoch Schreiber^3^, Tal Biron‐Shental^4^, Michal Kovo^3^

^1^Meir Hospital, Meir Hospital, HaMerkaz; ^2^Faculty of Medicine, Tel Aviv University, Tel Aviv, HaMerkaz; ^3^Meir Medical Center, Kfar Saba, HaMerkaz; ^4^Meir Medical Center, Meir Medical Center, HaMerkaz

11:45–13:00

**Objective**: We aimed to assess obstetric outcomes in nulliparous undergoing Induction of Labor (IOL) for different indications.

**Study Design**: This retrospective study included all singleton nulliparous ≥37 weeks with intact membranes and an unfavorable cervix, undergoing IOL during the last 7 years. Participants were categorized based on the indication for IOL: 1.placental complications (including hypertensive disorders, FGR, abruption, oligohydramnios), 2.fetal indications (including non‐reassuring fetal heart rate (NRFHR), reduced fetal movements), 3.prolonged pregnancy (≥41 weeks), 4.diabetes (DM)/isolated macrosomia, and 5.elective IOL (control). Outcomes were compared between each indication group and the control.

**Results**: A total of 2,618 IOL procedures were included, with 492 in the elective IOL group, 680 in the placental complications group, 427 in the DM/macrosomia group, 1,003 in the prolonged pregnancy group, and 400 in the fetal indications group. Higher rates of cesarean deliveries (CD) or vacuum extractions due to NRFHR were observed in IOL for placental complications, prolonged pregnancy, and fetal indications compared to the elective IOL group (*p* < 0.001). IOL for DM/macrosomia showed increased rates of CD, particularly due to arrest of labor progression (*p* = 0.002). Higher rates of composite adverse neonatal outcomes were observed in IOL for placental complications and DM/macrosomia compared to the control (*p* = 0.002, *p* = 0.001, respectively). Multivariable logistic regression, adjusted for confounders, revealed that IOL for DM/macrosomia were associated with an increased risk for CD, while IOL for placental complications & fetal indications were associated with an increased risk for interventions due to NRFHR. IOL for placental complications and DM/macrosomia increased the risk of composite neonatal adverse outcomes.

**Conclusion**: Specific indications necessitating IOL in nulliparous individuals exhibit an increased propensity for obstetric complications in contrast to a planned induction at 39 weeks. These associated risks ought to be comprehensively elucidated during the informed consent procedure.

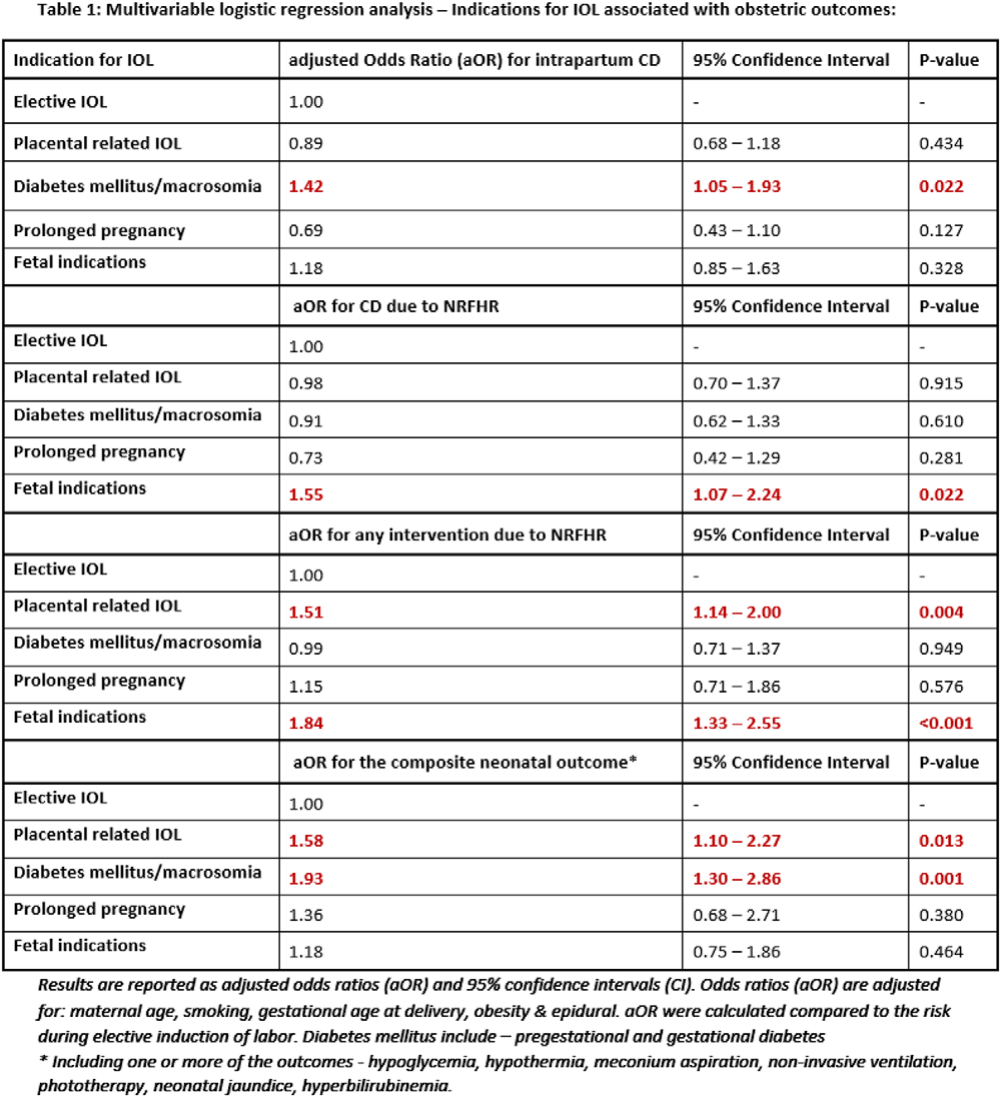

## 310 | Management of persistent pregnancy of unknown location: Should we be more cautious? A retrospective study

Montse Palacio^1^, Clàudia Galan^1^, Iris Liu^1^, Núria Baños^1^, Anna Goncé^1^, Federico Migliorelli^1^

^1^Hospital Clínic de Barcelona, Barcelona, Catalonia

11:45–13:00

**Objective**: Women diagnosed with a persistent pregnancy of unknown location (pPUL) (positive pregnancy test with no ultrasound image after 7 days of follow‐up) may be managed expectantly or treated with systemic methotrexate (MTX). The latter is preferred when bHCG levels are high (i.e., ≥ 2000 UI/L) while the former is an option if bHCG concentrations are low. We aimed to evaluate the outcomes of women with pPUL, regardless of the treatment they underwent.

**Study Design**: Retrospective observational study of the outcomes of women diagnosed of a pPUL, established when location of pregnancy could not be confirmed by ultrasound seven days after the first consultation. Women were followed up until resolution of the case.

**Results**: From February 2017 to June 2023, 302 women diagnosed with a PUL were admitted at our Early Pregnancy Clinic. Of these, 38 (12.6%) met criteria for pPUL. Two cases (one with bHCG < 2000 IU/L and one above) were lost to follow‐up.

Twenty‐eight women had bHCG < 2000 UI/L, and they all followed initial expectant management. Of these, 14 (50.0%) were failed PULs and did not require any further intervention. Five (17.9%) had ectopic pregnancies, with two requiring surgery and three responding to MTX therapy. Four women (14.3%) had pregnancies that were ultimately intrauterine, and the remaining five (17.9%) could not be located and received MTX for pPUL.

21.1% (8/38) had bHCG ≥ 2000 UI/L. Of these, three had intrauterine pregnancies, three remained as pPUL and received MTX, and two had ectopic pregnancies, with one requiring an emergency laparoscopy due to rupture (with bHCG > 7000 UI/L).

A location of the pregnancy was established in 29 (76.3%) cases, seven (24.1%) of which were intrauterine. Median time to reach a final diagnosis was 12 (range 8‐26) and 13 days (range 9‐21) if bHCG levels were below or above 2000 UI/L, respectively.

**Conclusion**: Expectant management in pPUL seems a reasonable option, even in the case of bHCG ≥ 2000 UI/L, when strict follow‐up can be assured. A final location will be established in most cases, with a very low risk of incidental rupture of an ectopic pregnancy.

## 311 | Prediction of spontaneous onset of labor at term: The PREDICT study

Federico Migliorelli^1^, Catherine McCarey^2^, Ludovica Ferrero^2^, Océane Pecheux^2^, Sara Marcenaro^2^, Oriane Tossa^2^, Véronique Othenin‐Girard^2^, Antonina Chilin^2^, Begona Martinez de Tejada Weber^3^

^1^Hospital Clínic de Barcelona, Barcelona, Catalonia; ^2^Hôpitaux Universitaires de Genève, Geneva, Geneve; ^3^Hopitaux Universitaires Genève (HUG), Geneve, Geneve

11:45–13:00

**Objective**: As the evidence supporting the benefits of systematic induction of labor (IOL) at term grows, obstetrical practices are shifting towards more interventionist approaches, potentially leading to less individualized care in labor wards. In this context, our objective was to develop a predictive tool to differentiate between women likely to undergo spontaneous labor and those who might require IOL after an expectant period.

**Study Design**: We conducted a prospective observational study enrolling women at 38^+5^ to 39^+6^ weeks of gestation with singleton pregnancies. After obtaining informed consent, we collected various clinical, ultrasonographic, and biochemical variables. All data were blinded to both participants and care providers. Using this information, we developed a logistic regression‐based model to predict spontaneous onset of labor between 39 and 41 weeks of gestation. The accuracy of the model was assessed using a receiver‐operating characteristics (ROC) curve.

**Results**: Between September 2019 and November 2022, 431 women were recruited. The average maternal age was 32.3 years (SD 3.9), mean body mass index (BMI) at delivery was 27.9 kg/m^2^ (SD 4.1), and 167 (38.7%) women were multiparous. The average gestational age at delivery was 40+4 weeks, with 43 women delivering by cesarean (10.0%). Spontaneous onset of labor occurred in 272 (63.1%) participants, while the remainder required either IOL for various reasons (157, 36.4%) or cesarean before onset of labor (2, 0.5%). Variables retained in the stepwise logistic regression model included maternal age (aOR 0.92, 95% CI 0.87‐0.97), BMI (aOR 0.88, 95% CI 0.84‐0.93), multiparity (aOR 2.17, 95% CI 1.38‐3.41) and cervical length as measured by transvaginal ultrasound (aOR 0.95, 95% CI 0.93‐0.97). The model demonstrated moderate predictive capacity, with an area under the ROC curve of 0.70 (95% CI 0.65‐0.75).

**Conclusion**: Maternal age, BMI, multiparity, and cervical length are independent factors associated with the likelihood of spontaneous onset of labor. However, the predictive capacity of our model remains moderate, suggesting further refinement is necessary.

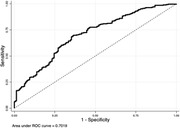

## 312 | Maternal early warning signs and escalation to intensive care unit for pregnant and postpartum women

Ahmed Zaki Moustafa^1^, Sandra Sadek^2^, Carly Shahbazian^3^, Sean C. Blackwell^4^

^1^Division of Maternal‐Fetal Medicine, Department of Obstetrics, Gynecology and Reproductive Sciences, McGovern Medical School at UTHealth Houston, University of Texas–Houston, Texas; ^2^University of Texas Health Science Center in Houston, McGovern Medical School, Houston, Texas; ^3^Children's Memorial Hermann Hospital, Houston, Texas; ^4^McGovern Medical School at UTHealth Houston, Houston, Texas

11:45–13:00

**Objective**: Early identification of individuals at greatest risk of developing pregnancy morbidity is crucial to allow for timely interventions to avert severe morbidity and mortality. The purpose of this study is to evaluate our real‐world experience utilizing the Maternal Early Warning Signs (MEWS) and evaluating its role in need for care escalation to intensive care unit (ICU) admissions at a level IV Maternal Care facility.

**Study Design**: This is a retrospective cohort quality improvement research study of all pregnant and postpartum women who had MEWS trigger over a one‐year period (Jan 1, 2023‐Dec 31, 2023) at level IV Maternal Care facility. Women were excluded if already in an ICU or intermediate care (IMU) unit or were transferred from another facility directly to ICU/IMU setting. We evaluated the frequency and primary trigger for each MEWS event for those with and without ICU admission.

**Results**: Over the one‐year study period, there were 5002 deliveries at our center with 1,065 recorded MEWS events and 99 ICU admissions for pregnant or postpartum women. Of the captured MEWS triggers, blood pressure parameters accounted for 470 triggers (46.9%), followed by 145 for heart rate (17%). Of the women who required ICU escalation, 24% were sent from OR or PACU. The most common etiologies for ICU admissions included cardiopulmonary disease (39%), hemorrhage (16%) and sepsis (12%). Among our ICU admissions from within the hospital 34% triggered a single MEWS and 66% triggered multiple MEWS prior to ICU transfer. Of the remaining who were transferred from floor status after MEWS trigger event(s), tachycardia, hypertension and hypoxia were the most strongly associated with need for escalation (see Figure).

**Conclusion**: MEWS is an important tool in identifying patients at risk of clinical deterioration. In our center, the most common MEWS triggers were for hypertension, tachycardia, and low urine output. The majority of these women were evaluated and resuscitated and did not require ICU care. MEWS criteria that resulted in need for ICU care were most associated with cardiopulmonary conditions, hemorrhage and sepsis.

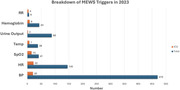

## 313 | Current clinical practice patterns in late gestational diabetes mellitus screening among prenatal care providers

Brian Z. Druyan^1^, Anna Sfakianaki^1^, Eva Hoffmann^1^, Rima Hajjar^2^, Nicholas Tinker^3^, Megan Piacquadio^1^, Staci Marbin^2^, Shauna T. Harris^4^, Yuri Cruz^1^

^1^University of Miami, Miami, Florida; ^2^University of Miami Miller School of Medicine, Miami, Florida; ^3^University of Miami and Jackson Memorial Hospital, Miami, Florida; ^4^University of Miami/Jackson Memorial Hospital–‐ Miami, FL, Coral Springs, Florida

11:45–13:00

**Objective**: Current Gestational Diabetes Mellitus (GDM) screening guidelines from the American College of Obstetrics and Gynecology (ACOG) and the United States Preventative Services Task Force (USPSTF) differ, potentially leading to practice variations. This study aims to explore these variations and assess the validity of using existing screening methods beyond the recommended 24–28‐week window.

**Study Design**: A structured questionnaire was distributed electronically to Obstetrics and Gynecology (OBGYN) generalists, Maternal Fetal Medicine (MFM) physicians and fellows, Family Medicine physicians, Family Planning physicians, Certified Nurse Midwives (CNMs), and Advanced Practice Nurse Practitioners (APRNs). The survey assessed screening practices, familiarity with guidelines, barriers to routine screening, and approaches to late GDM screening after the recommended timeframe.

**Results**: Sixty‐two healthcare providers participated thus far, including 60% generalist OB‐GYN physicians, 20% MFM Fellows, and others. Most practiced in urban settings (80%) in the southeastern United States. Over 90% were willing to conduct late GDM screening if patients missed the recommended window. The 50‐G glucose challenge test was the preferred screening method, used by nearly 90% of respondents. Most were familiar with ACOG guidelines (60%) but less so with USPSTF guidelines (16%). Two‐thirds believed late screening improves both maternal and neonatal outcomes.

**Conclusion**: Providers show willingness to conduct late GDM screening and prefer the 50‐G glucose challenge test. Barriers to routine screening include late presentation to prenatal care and patient nonadherence. There's consensus on the need for guideline clarification and more education on late GDM screening. Further research is warranted to understand potential negative consequences and improve patient care.

## 314 | Fetal fraction in patients with Systemic Lupus Erythematosus

Chi Nguyen^1^, Nicole Legro^1^, Sara N. Iqbal^1^, Victoria R. Greenberg^2^

^1^Medstar Washington Hospital Center/Georgetown, Washington, District of Columbia; ^2^Medstar Washington Hospital Center /Georgetown, Washington, District of Columbia

11:45–13:00

**Objective**: We sought to evaluate for differences in fetal fraction in individuals with SLE compared to those without SLE. Additionally, we assessed whether use of immunomodulating medications (IM) at time of cell‐free DNA (cfDNA) screening has any effect on fetal fraction for patients with systemic lupus erythematous (SLE).

**Study Design**: We performed a retrospective cohort study of singleton pregnancies with and without SLE that underwent cfDNA testing between 2017 and 2024. Patients without SLE served as controls and were matched to patients with SLE during pregnancy in a 3:1 fashion, controlling for gestational age and body mass index. Exclusion criteria included individuals taking anticoagulants, history of organ transplant, and high‐risk cfDNA results concerning for aneuploidy. IM therapy included corticosteroids, tacrolimus, hydroxychloroquine, and azathioprine. We used the student t‐test for normally distributed variables, chi‐square and Fisher's exact tests for categorical variables and ANOVA to compare three groups. Multivariate regression analysis was performed to evaluate the effect of immunomodulator use on fetal fraction, after adjusting for BMI and gestational age.

**Results**: In the study period, 196 patients were included (49 with SLE and 147 without SLE). Baseline demographics between the two groups were similar with respect to BMI, race, and smoking status (Table 1) with a statistically significant difference in age. Mean fetal fractions were similar in those with and without SLE (Table 1). In the subset of individuals with SLE, use of IM medication did not result in higher fetal fraction compared to individuals with SLE not on IM medication, even after adjusting for gestational age and BMI (aβ .104, 95% CI −1.6, 3.7)

**Conclusion**: Contrary to other reports, in this retrospective cohort analysis, autoimmune disease status did not show an effect on fetal fraction even when adjusting for ‐IM use. Additional studies with different autoimmune diseases are needed to evaluate the effect of autoimmune disease on fetal fraction.

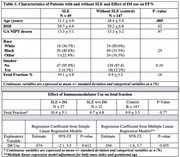

## 315 | Leptin and visfatin levels in women with obesity in active labor and obstetric outcomes

Oskar Nydal^1^, Sarah Arrowsmith^2^, Sofia Nevander^1^, Marie Blomberg^3^, Sara Carlhäll^3^

^1^Dept of Obstetrics and Gynecology and Dept of Biomedical and Clinical Sciences, Linköping University, Linköping, Sweden, University of Linköping, Linköping, Ostergotlands Lan; ^2^Department of Life Sciences, Manchester Metropolitan University, Manchester Metropolitan University, England; ^3^Dept of Obstetrics and Gynecology and Dept of Biomedical and Clinical Sciences, Linköping University, Linköping, Sweden, Linköping University, Linköping, Ostergotlands Lan

11:45–13:00

**Objective**: Leptin and visfatin are adipokines with altered levels in complicated pregnancies and suppress myometrial contractility in vitro. They are also associated with obesity in general. Obese pregnant women are at risk for labor dystocia, cesarean section and postpartum hemorrhage, indicating dysfunctional uterine contractions. We examined the association between leptin and visfatin levels in obese women in labor and obstetric outcomes related to impaired uterine contractility.

**Study Design**: A Swedish prospective cohort study including 263 obese women. Maternal blood was collected in active labor. Plasma leptin and visfatin concentrations were determined by sandwich ELISA. The study population was categorized into three groups according to leptin and visfatin levels ( < 25, 25‐75 and ≥75 percentiles). Maternal and obstetric outcomes were compared between the leptin/visfatin categories using Chi^2^ test. Pearson's correlation, linear regression and Kaplan Meier analyses were performed to measure the association between leptin/visfatin levels and body mass index (BMI) and time in labor respectively.

**Results**: Leptin and visfatin levels positively correlated with maternal BMI; *r* = 0.300, and *r* = 0.139 (*p* < 0.001 and *p* = 0.045 respectively). Labor duration ≥12 hours was more common in obese women with high leptin levels (*p* = 0.023). Leptin was not associated with postpartum hemorrhage or mode of birth. Visfatin was not associated with any outcomes associated with impaired uterine contractility. An increase in leptin, but not visfatin, was associated with an increase in labor duration: 1.83min/1ng/mL leptin, *p* = 0.002; 0.60 min/1ng/mL visfatin, *p* = 0.218. More women with combined high leptin and visfatin levels had longer labors compared to women with low levels after 7 hours active labor (Figure).

**Conclusion**: Both leptin and visfatin levels positively correlated with BMI in obese women in labor. Leptin but not visfatin was associated with longer labor. A plausible underlying mechanism for prolonged labor in obese women may be high leptin levels. A potential synergistic effect of high visfatin levels may also be possible.

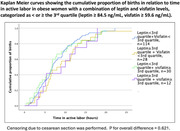

## 316 | Decreased blood loss in placenta accreta deliveries with implementation of stapler for hysterotomy entry

AnneMarie E. Opipari^1^, Katie Foug^2^, Aimee Rolston^3^, Shitanshu Uppal^4^, David E. Arnolds^5^, Lena Napolitano^4^, Molly J. Stout^5^, Jourdan E. Triebwasser^6^

^1^University of Michigan, University of Michigan/Ann Arbor, MI; ^2^University of Michigan, University of Michigan/Ann Arbor, Michigan; ^3^University of Michigan, University of Michigan/ Ann Arbor, Michigan; ^4^University of Michigan, University of Michigan/Ann Arbor, Michigan; ^5^University of Michigan Medical Center, Ann Arbor, Michigan; ^6^University of Michigan, Ann Arbor, Michigan

11:45–13:00

**Objective**: Massive blood loss and requirement for blood transfusion are common in cesarean hysterectomy performed for placenta accreta spectrum disorder (PAS). Surgical innovation such as utilization of stapler devices to provide hemostasis for hysterotomy may decrease intraoperative blood loss and maternal morbidity. Our objective was to evaluate blood loss and blood transfusion after implementation of stapler hysterotomy technique.

**Study Design**: Retrospective cohort of all scheduled cesarean hysterectomies completed for PAS from January 1st 2022–April 1st 2024 at a single academic center. Routine use of stapler for hemostatic uterine incision started May 2023. We included scheduled cesarean hysterectomy completed for PAS from January 1st 2022–April 1st 2024. We excluded emergent cases, cases without hysterectomy, cases conservatively managed at the time of delivery of infant, and any case where surgical procedure involved attempt of removal of placenta prior to proceeding with hysterectomy. Primary exposure was utilization of stapler for hysterotomy compared to traditional scalpel hysterotomy. Primary outcome was EBL (ml). Secondary outcomes were EBL >1500 mL and receipt of packed red blood cell (pRBC) transfusion.

**Results**: 31 cases of PAS were included, 13 (41.9%) utilized the stapler and 18 (58.1%) utilized scalpel hysterotomy. Blood loss was numerically, though not statistically, less in cases that utilized the stapler (median 1200ml versus 1963ml, *p* = 0.14). Stapler use was associated with reduced blood loss > 1500mL (23% vs. 61%, *p* = 0.04). The frequency of pRBC transfusion in stapler group was 31% compared to 50% in scalpel group *p* = 0.3.

**Conclusion**: Utilization of a commonly available surgical stapler device for hysterotomy entry in PAS cases likely decreases surgical blood loss to a clinically significant magnitude. Given heterogenous practices in PAS management and low volumes at single sites, multicenter studies and randomized control trials are necessary to test best practices and identify opportunities for surgical innovation to continue to reduce significant maternal morbidity with this condition.

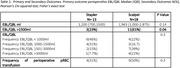

## 317 | Efficacy of antepartum fetal surveillance for stillbirth prevention

Sara Ornaghi^1^, Alessandro Ghidini^2^, Marietta Cacace^3^, Simona Fumagalli^4^, Kavya Kuttuva^3^, Alyssa Cacace^3^, Anna Locatelli^1^

^1^Department of Obstetrics and Gynecology, Unit of Obstetrics, Fondazione IRCCS San Gerardo dei Tintori, Monza, Lombardia; ^2^Antenatal Testing Center, Inova Alexandria Hospital, Antenatal Testing Center, Inova Alexandria Hospital, MD; ^3^Antenatal Testing Center, Inova Alexandria Hospital, Antenatal Testing Center, Inova Alexandria Hospital, Maryland; ^4^University of Milan‐Bicocca, University of Milan‐Bicocca School of Medicine and Surgery, Lombardia

11:45–13:00

**Background**: The goal of antepartum fetal surveillance (AFS) is to reduce stillbirth (SB); however, evidence for its efficacy is based on dated studies and indications for AFS have changed over the years due to identification of risk factors previously unknown.

**Objective**: In 2019 the ACOG issued specific recommendations for AFS based on individual risk factors. As similar recommendations were already in place at our institution, we have evaluated the efficacy of AFS for SB prevention in a 5‐year cohort.

**Study Design**: Retrospective cohort study of all deliveries at a single institution between 7/1/2013 and 6/30/2018). Excluded were multiples, anomalous fetuses or newborns, and deliveries before 32 weeks. AFS was conducted with a modified biophysical profile (non‐stress test–NST and amniotic fluid index), with a complete biophysical profile as back‐up for non‐reactive NST. All cases of SB were individually reviewed to verify the presence of risk factors, the results of AFS if done, and calculate the interval between last test and delivery. The electronic medical records were queried to identify women who underwent AFS and those who did not. Chi‐square was used to compare SB rates between the two groups.

**Results**: 16,827 women fulfilled the inclusion criteria. Of them, 5711 (34%) had risk factors which prompted AFS; 37% of such women had 2 or more risk factors. SB occurred in 1.6‰ of them (9/5711) (3 had 1 risk factor, 4 had 2, and 2 had 3 risk factors). Rates of SB were similar between women who had AFS and those who did not (9/5711 or 1.6‰ vs 27/11,116 or 2.2‰, *p* = 0.42, OR 0.73, 95% CI 0.34‐1.57). The false‐negative rate at < 7 days of reassuring AFS among compliant women was 1.2‰ (7/5711). Rates of preterm delivery were similar in the tested vs untested population (6.5% or 371/5711 vs 6.0% or 669/11,116, *p* = 0.22).

**Conclusion**: Indications for AFS have greatly changed compared with traditional indications. Implementation of AFS in women with risk factors similar to those recommended by the ACOG lowers the risk of SB to that of low‐risk pregnancies.

## 319 | Clinical presentation associated with placental pathology in spontaneous preterm birth

Sunitha Suresh^1^, Alexa A. Freedman^2^, Emmet Hirsch^1^, Linda M. Ernst^1^

^1^Endeavor Health, Evanston, Illinois; ^2^Northwestern University, Chicago, Illinois

11:45–13:00

**Objective**: Two clinical presentations leading to spontaneous preterm birth (sPTB) are labor with intact membranes (“preterm labor”, PTL), or preterm premature rupture of membranes (PPROM). The objective of this study is to correlate the initiating event of labor with the 4 major categories of placental pathology to understand how patterns of placental injury relate to clinical presentations.

**Study Design**: In this retrospective study of patients with sPTB >20 and < 37 weeks at a hospital system, placental pathology was classified into 4 primary classes: maternal vascular malperfusion (MVM), fetal vascular malperfusion (FVM), chronic inflammation (CI), and acute inflammation (AI). An obstetrician classified patients into PPROM and PTL. Clinical presentation was correlated with placental pathology. Results were stratified by “early” and “late” sPTB, using a cutoff of 34 weeks. Chi squared analysis was used, followed by a logistic model including all 4 placental pathologies. A sensitivity analysis was done, limited to subjects delivered within 48 hours of rupture of membranes (ROM).

**Results**: A total of 342 participants with sPTB were included. 68.7% of participants presented with PPROM and 31.3% presented with PTL. The prevalence of AI was 42.7%, CI was 40.6%, MVM was 31.3%, and FVM was 21.3%. In early sPTB, those with AI were more likely to present with PPROM (74.0% vs 51.0%, aOR 2.73 for PPROM, 95% CI 1.19, 6.23, Table), and those with CI were more likely to present with PTL (26.0% vs 47.1%, aOR for PTL 2.65 95% CI 1.19, 5.88). In late sPTB, those with AI were more likely to present as PTL (48.2% vs 24.1%, aOR 3.06 for PTL, 95% CI 1.60, 5.83). A sensitivity analysis for the 81% of patients delivered within 48 hours of ROM demonstrated a persistent association between CI and PTL among early sPTB (aOR 3.48, 95% CI 1.30, 9.33), and between AI and PTL only in late sPTB (aOR 3.7 95% CI 1.88, 7.29).

**Conclusion**: Among sPTB, there were differences in clinical presentation by pathology, which differed by early and late PTB. Further work is needed to understand how common placental injury leads to clinical presentations.

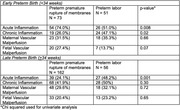

## 320 | Placental pathology associated with hypertensive disorders of pregnancy by timing of delivery

Sunitha Suresh^1^, Alexa A. Freedman^2^, Sandhya Chandrasekaran^3^, Beth A. Plunkett^1^, Linda M. Ernst^1^

^1^Endeavor Health, Evanston, Illinois; ^2^Northwestern University, Chicago, Illinois; ^3^University of Chicago School of Medicine, Chicago, Illinois

11:45–13:00

**Objective**: Twin pregnancies have a high prevalence of hypertensive disorders of pregnancy (HDP). Knowledge of the underlying placental pathology associated with preterm HDP vs term HDP is limited. We hypothesize that maternal vascular malperfusion (MVM), will occur more frequently among those delivered < 36 weeks.

**Study Design**: This retrospective cohort study includes patients from a single health care system with a diamniotic twin gestation diagnosed with a HDP and with placental pathology, excluding pregnancy complicated by vanishing twin, pregnancy reduction, chronic hypertension. Charts were audited and placentas reviewed by a placental pathologist. Placentas were evaluated for the presence (in one or both placentas) of any of four pathologic categories ‐acute inflammation (AI), chronic inflammation (CI), maternal vascular malperfusion (MVM), fetal vascular malperfusion (FVM). Cutoff of 36 weeks was used due to clinical indication for delivery of monochorionic gestations. Univariable analyses compared demographic, clinical and placental characteristics between those patients with delivery < 36 vs ≥36 weeks. Multivariable logistic regression assessed the independent association of placental histology with timing of delivery, adjusted for remaining pathologies. The prevalence of MVM was examined by gestational age (GA) at delivery.

**Results**: 151 twin dyads were included, of which 37.7% experienced delivery < 36 weeks. There were no demographic differences (Table). MVM was more common among births < 36 weeks as compared to births ≥ 36 weeks (56.1% vs 24.5%, aOR 4.80 (95% CI 2.23, 10.35) *p* < 0.01), and CI was less common (aOR 0.27, 95% CI 0.12, 0.63, *p* < 0.01). There was no difference in AI or FVM. The prevalence of MVM decreased with increasing GA, at > 80% among those < 33 weeks, and decreasing to 19% among those ≥ 37 weeks (*p* < 0.01).

**Conclusion**: MVM is more common among twin pregnancies with HDP delivering < 36 weeks and CI is more common among those delivering ≥ 36 weeks. The prevalence of MVM decreased with increasing GA. Further work is needed in understanding the pathophysiology of HDP in twin gestation.

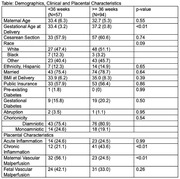

## 321 | Placental pathology associated with hypertensive disorders of pregnancy in twin gestation

Sunitha Suresh^1^, Alexa A. Freedman^2^, Sandhya Chandrasekaran^3^, Beth A. Plunkett^1^, Linda M. Ernst^1^

^1^Endeavor Health, Evanston, Illinois; ^2^Northwestern University, Chicago, Illinois; ^3^University of Chicago School of Medicine, Chicago, Illinois

11:45–13:00

**Objective**: Twin pregnancies have a high prevalence of hypertensive disorders of pregnancy (HDP). Knowledge of the underlying placental pathology specific to twin gestations complicated by HDP is lacking. The purpose of this study is to characterize placental pathology associated with HDP in twin gestations.

**Study Design**: This is retrospective cohort study of patients with a diamniotic twin gestation and placental pathology available in a single health care system. Exclusion criteria included pregnancy complicated by vanishing twin, pregnancy reduction, and chronic hypertension. Charts were audited and placentas reviewed by a perinatal pathologist. Placentas were evaluated for the presence (in one or both placentas) of any of four pathologies: acute inflammation (AI), chronic inflammation (CI), maternal vascular malperfusion (MVM), fetal vascular malperfusion (FVM). Univariable analyses compared demographic, clinical and placental characteristics between patients with and without HDP. Multivariable logistic regression assessed the independent associations among placental pathology and HDP, adjusted for remaining placental pathologies.

**Results**: HDP was present in 151/456 (33.1%) of twin gestations. Patients with HDP had a higher BMI, otherwise there were no significant clinical or demographic differences (Table). All 4 patterns of injury were seen among twin gestation. In unadjusted models, prevalence of AI and CI was less in HDP than non HDP (AI–no HDP 35.4% vs 24.5% HDP *p* = 0.02, CI–no HDP 44.9% vs 35.1% HDP, *p* = 0.04). There was no difference in the frequency of MVM or FVM (MVM–no HDP 28.2% vs HDP 36.4%, *p* = 0.07, FVM–no HDP 45.6% vs HDP 36.4%, *p* = 0.06). In adjusted models, there was a decreased odds of AI among HDP (aOR 0.6, 95% CI 0.39, 0.94), and no difference in other pathologies including MVM (aOR MVM 1.47 95% CI 0.96, 2.24).

**Conclusion**: The full range of placental pathology is seen in HDP among twin pregnancy. Interestingly, MVM is a common finding among twin gestation and does not occur with a statistically greater frequency among twin gestations complicated by HDP.

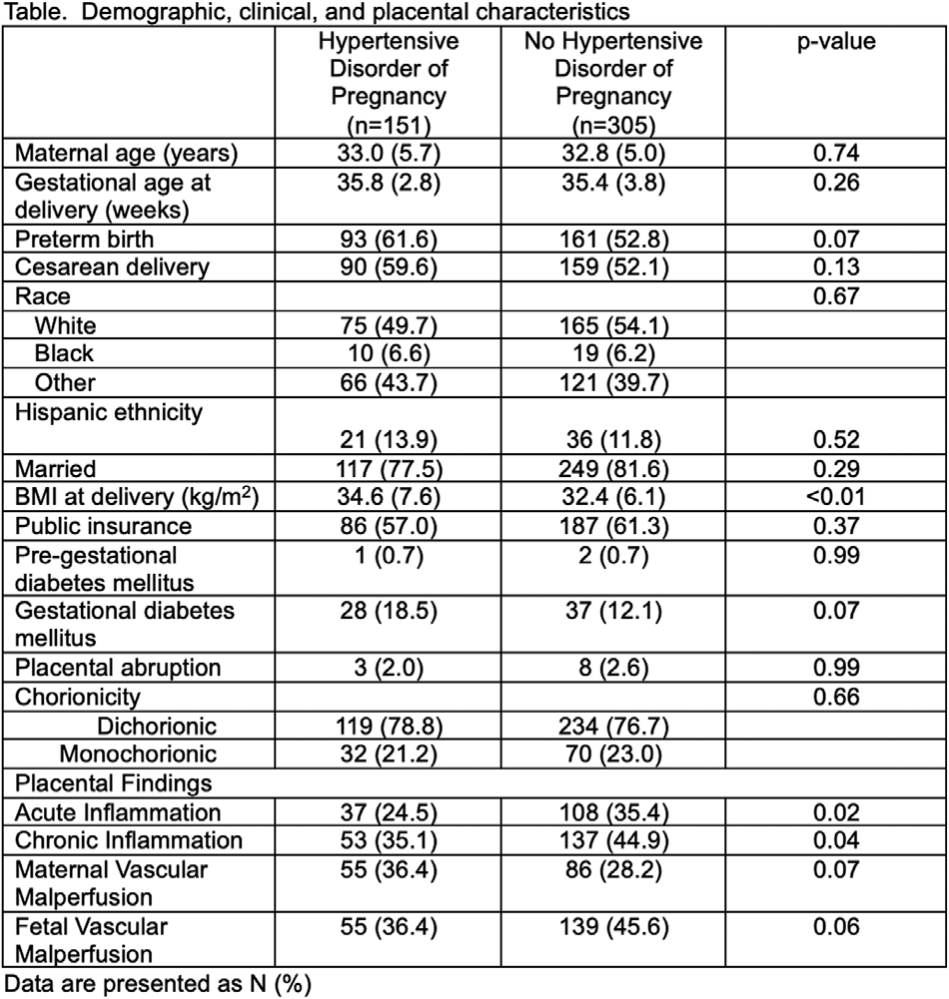

## 322 | Association between first‐trimester maternal biomarkers and skin microvascular reactivity assessed by laser speckle contrast imaging

Viola SERAVALLI^1^, Mor Huri^1^, Isabella Abati^2^, Alessia Piacenza^1^, Chiara Bartolini^1^, Arianna Vallario^1^, Mariarosaria Di Tommaso^1^

^1^University of Florence, University of Florence, Toscana; ^2^University of Florence, Florence, Toscana

11:45–13:00

**Objective**: To investigate the association between first trimester maternal serum biochemical and biophysical markers and skin microvascular reactivity by using laser speckle contrast imaging (LSCI) combined with post‐occlusive reactive hyperemia (PORH).

**Study Design**: Fifty‐three women with a singleton gestation were enrolled during the first trimester scan. Microvascular skin blood flux was recorded using LSCI coupled with PORH. Skin perfusion was recorded before (baseline flux), during and after (peak flux) a 3‐minutes occlusion obtained with an inflated pneumatic cuff. The percentage increase of flux from baseline to the peak (base‐to‐peak flux) was calculated and correlated with serum biochemical markers (Placental growth factor, PlGF, Pregnancy‐associated protein A, PAPP‐A, free beta human Chorionic Gonadotropin, free β‐hCG), expressed as multiple of the median (MoM), and with maternal biophysical markers (Mean arterial pressure; Uterine artery pulsatility index).

**Results**: PlGF MoM levels showed a moderate positive correlation with base‐to‐peak flux (r = 0.51, 95% Confidence Interval, CI, 0.27‐0.69), and values above the 90^th^ percentile were associated with higher base‐to‐peak flux compared to lower levels (320.4% versus 230.9%, *p* = 0.0009). PAPP‐A MoMs presented a weak but significant positive correlation with base‐to‐peak flux, and levels above the median were associated with higher base‐to peak flux compared to values below the median (253.4% versus 215.1%, *p* = 0.02). Furthermore, a moderate positive correlation was found between free β‐hCG MoM levels and peak flux (r = 0.4, 95% CI 0.15‐0.60). No correlations were found between the hyperemic response and the explored maternal biophysical marker.

**Conclusion**: Our study demonstrates positive correlations between the parameters of hyperemic response and first trimester serum biochemical markers. These novel findings suggest the potential value of first trimester assessment of microvascular response as an early marker of placental function.

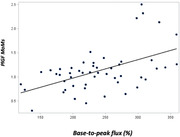

## 323 | Microvascular function assessed by laser speckle contrast imaging in pregnancies complicated by preeclampsia and FGR

Viola SERAVALLI^1^, Mor Huri^1^, Isabella Abati^2^, Arianna Vallario^1^, Chiara Bartolini^1^, Gaia Impastato^1^, Michela Santalucia^1^, Alessia Piacenza^1^, Mariarosaria Di Tommaso^1^

^1^University of Florence, University of Florence, Toscana; ^2^University of Florence, Florence, Toscana

11:45–13:00

**Objective**: To compare microvascular function assessed by laser speckle contrast imaging (LSCI) combined with post‐occlusive reactive hyperemia (PORH) between pregnancies complicated by preeclampsia and/or fetal growth restriction (FGR) and uncomplicated pregnancies.

**Study Design**: Twenty‐five women with a singleton gestation complicated by preeclampsia and/or FGR, along with 25 uncomplicated pregnancies serving as controls, were enrolled. Cases and controls were matched for gestational age. Skin microvascular blood perfusion was recorded using LSCI coupled with PORH. Skin perfusion was recorded before (baseline flux), during and after (peak flux) a 3‐minutes occlusion obtained with an inflated pneumatic cuff. The percentage increase of flux from baseline to the maximal post‐occlusive response (base‐to‐peak flux) was calculated.

**Results**: Out of the 25 cases, 12 had both FGR and preeclampsia, another 12 had FGR only, and one patient had only preeclampsia. Base‐to‐peak flux was significantly lower in the group of the 25 pregnancies complicated by FGR or preeclampsia compared to the uncomplicated controls (mean and SD: 215.5 ± 61.2% versus 283.6 ± 64.2%, *p* < 0.001), indicating impaired microvascular reactivity. The 13 pregnancies complicated by preeclampsia had significant decreased microvascular function compared to the 12 pregnancies with FGR without preeclampsia (mean and SD: 190.5 ± 52.9% versus 246.2 ± 59.8, *p* = 0.018).

**Conclusion**: Microvascular function is impaired in pregnancies complicated by placental‐related diseases, and in particular in those with preeclampsia. Further investigation into the use of the LSCI technique as an adjunctive tool for the prediction and diagnosis of preeclampsia is warranted.

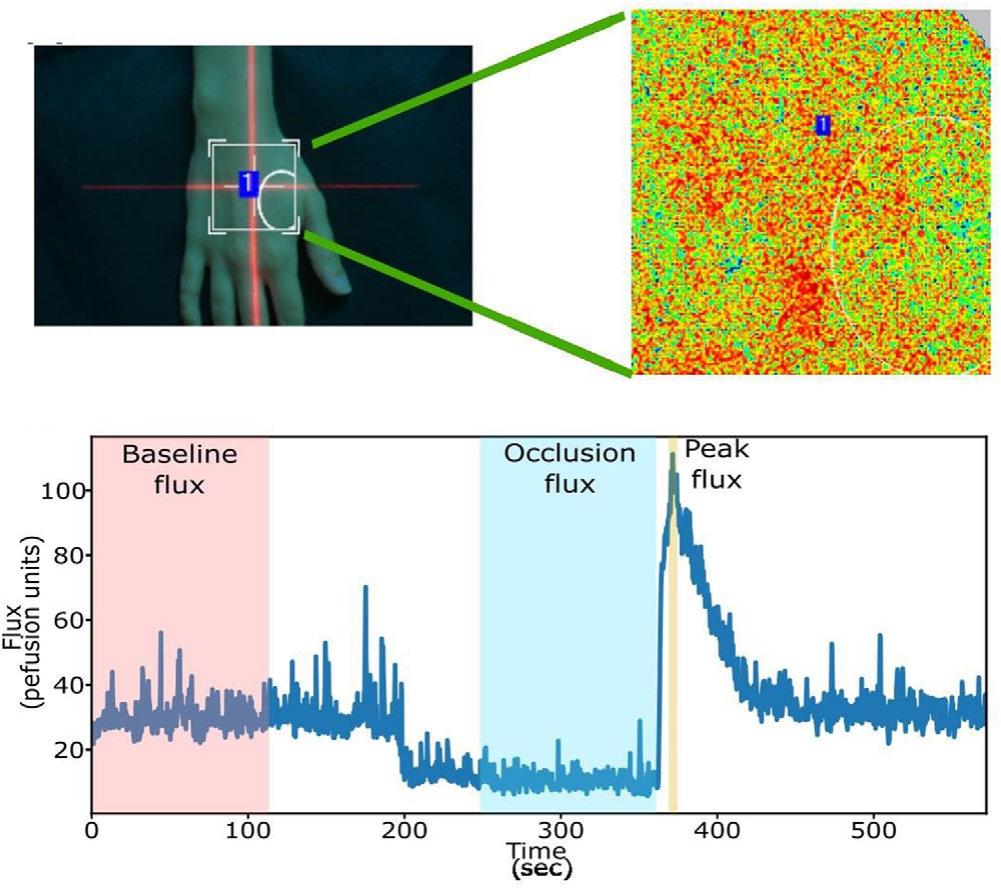

## 324 | The Epidemic of Congenital Syphilis in Arizona

Meghan M. Shea^1^, Darean Hunt^2^, Linda R. Chambliss^3^

^1^Creighton University Residency in Obstetrics and Gynecology, Phoenix Campus, Phoenix, AZ; ^2^Creighton University Residency in Obstetrics and Gynecology, Phoenix Campus, Phoenix, Arizona; ^3^Valleywise Medical Center, Phoenix, Arizona

11:45–13:00

**Objective**: Determine the rates of congenital syphilis in Arizona from January, 2015 to the December, 2023.

**Study Design**: Retrospective cohort study using data from the Arizona Department of Health and the Center for Disease Control and Prevention.

**Results**: In the years 2017‐2021, the rate of congenital syphilis has increased in the U.S. by 219% but increased in Arizona by 494%. The cases have doubled every year since 2015. In 2023 there were 213 cases of congenital syphilis with 21 infants either stillborn or who suffered a neonatal death. The 3 counties in Arizona with the highest rates of syphilis (Apache, La Paz and Gila) are all underserved rural areas with large Native American populations.

**Conclusion**: Arizona continues to have increasingly high levels of congenital syphilis that will require a concerted public health effort to reduce. Cases are particularly prevalent in underserved rural areas with large Native American populations. This epidemic is especially tragic given that syphilis in pregnancy is easily treated with penicillin and adequate maternal treatment would reduce, and possibly eliminate, these devastating sequelae in the fetus and newborn.

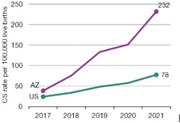

## 325 | Quantitative assessment of Fetal Heart Rate and Amniotic Fluid Volume in self‐operated home ultrasound

Anat Pardo^1^, Shiri Barabash Hazan^2^, Shir Nahum Fridland^2^, Anat Shmueli^2^, Or Lee Rak^2^, Emilie Klochendler Frishman^2^, Hadas Zafrir Danieli^2^, Ronit Shtrichamn^3^, shmuel Azulay^3^, Eran Hadar^4^

^1^The Helen Schneider Hospital for Women at Rabin Medical Center, Petach Tikwa, HaMerkaz; ^2^Helen Schneider Hospital for Women, Rabin Medical Center, petach tikwa, HaMerkaz; ^3^Pulsenmore, Omer, HaDarom; ^4^Rabin Medical Center, Petach Tikva, HaMerkaz

11:45–13:00

**Objective**: Self‐operated home ultrasound (Pulsenmore, Israel) has enabled remote fetal monitoring. We aimed to evaluate the accuracy of quantitative measurement tools for Fetal Heart Rate (FHR) and Amniotic Fluid Volume (AFV) using the Pulsenmore (PNM) home ultrasound system operated in two modes: App‐Guided (AG)–where the user follows video tutorials in the Pulsenmore App, and Clinician Guided (CG)–where the user's self‐scan is guided by a clinician via telemedicine.

**Study Design**: An observational study compared FHR and Maximal Vertical Pocket (MVP) measurements obtained from ultrasound clips, generated by participant's self‐scanning using Pulsenmore ultrasound, with those from In‐Clinic conventional ultrasound. Each participant underwent four scans, comprising two pairs: (1) Self‐scan using AG mode followed by an In‐Clinic conventional scan (INC1), and (2) Self‐scan using CG mode followed by an In‐Clinic conventional scan (INC2). Scans were reviewed by three independent blinded readers, with agreement defined for differences in average measured values for FHR ( < 32BPM) and MVP (within 20%) between modalities.

**Results**: Twenty‐eight participants completed the study, 9 (32%) with previous diagnosis of abnormal AFV. Fetal heart rate and AFV were visualized in > 94% of scans with adequate image quality score for diagnosis (3.44 of 5). FHR and MVP measurements were obtained in 85% and 92% of AG scans, respectively, and in 95% of CG scans. PNM‐INC FHR average absolute difference was 10.8 ± 7.5 bpm for AG vs INC1 (7.3% percent error [PE]), and 5.8 ± 5.1 bpm for CG vs. INC2 (4% PE) (both *p* < 0.001). PNM‐INC MVP average difference was 1.3 ± 1.4 cm (*p* < 0.16) for AG vs. INC1 (22% PE), and 0.9 ± 0.8 cm (*p* < 0.002) for CG vs. INC2 (14.5% PE). Evaluation of AFV as normal or abnormal, based on MVP measurements, revealed sensitivity of 88% and 100% for AG and CG, respectively, and specificity of 95% for both PNM modes.

**Conclusion**: The Pulsenmore home ultrasound, operated by lay users, provides high‐quality and accurate evaluation of AFV and FHR, non‐inferior to standard assessments.

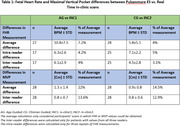

## 326 | The Effect of Chorionicity on Hypertensive Disorders in Pregnancy

Anat Pardo^1^, Or Bercovich^2^, David Danon^3^, Yuval Gielchinsky^4^, Sharon Sigal‐Kaplun^3^, Yael haring^3^, Noam Pardo^5^, Shiri Barabash Hazan^3^, Eran Hadar^2^, Kinneret Tenenbaum Gavish^3^

^1^The Helen Schneider Hospital for Women at Rabin Medical Center, Petach Tikwa, HaMerkaz; ^2^Rabin Medical Center, Petach Tikva, HaMerkaz; ^3^Helen Schneider Hospital for Women, Rabin Medical Center, petach tikwa, HaMerkaz; ^4^Rabin Medical Center, Rabin Medical Center, HaMerkaz; ^5^Maternal Fetal Medicine Division, Sheba Medical Center, Tel Hashomer, Sheba Medical Center/Ramat Gan, HaMerkaz

11:45–13:00

**Objective**: Monochorionic (MC) in comparison to Dichorionic (DC) pregnancies have higher rates of neonatal complications due to their shared placenta. It is unclear whether maternal complications, influenced by the placenta, such as Hypertensive (HTN) disorders are affected by chorionicity.

We aimed to examine whether the rate of HTN disorders in pregnancy differ by chorionicity.

**Study Design**: Retrospective cohort of 1,242 twin gestations from 2015 to 2022 in a single tertiary center. 1,002 DC pregnancies were compared to 240 MC pregnancies. Primary outcome was HTN disorders such as gestational hypertension (GHTN), preeclampsia (PE) and HELLP syndrome. Secondary outcomes included preterm birth (PTB), gestational diabetes (GDM), cholestasis of pregnancy, fetal growth restriction (FGR) and neonatal intensive care unit (NICU) admission.

**Results**: Women with MC pregnancies were younger in comparison to women with DC pregnancies (31.2 vs 32.8 years, *p* < 0.001), had higher rates of spontaneous conception (79.4% vs 49.9%, *p* < 0.001), delivered at an earlier gestational age (34+6 vs 36+0 weeks, *p* < 0.001) and had higher rates of GDM in prior pregnancy (7.5% vs 4.1%, *p* = 0.041).

MC and DC pregnancies did not differ in the rate of HTN disorders, including GHTN, PE with or without severe features and HELLP syndrome. No differences were found between the groups in the incidence of GDM and cholestasis of pregnancy.

Women with MC compared with DC pregnancies had higher rates of PTB at < 37 (adjusted OR (aOR) 5.690, 95% CI 1.689‐19.182) and < 34 gestational weeks (aOR 2.576, 95% CI 1.238‐5.362) and more NICU admission (aOR 2.953, 95% CI 1.487‐5.866).

When estimating the cumulative incidence of HTN disorders during pregnancy (Figure 1) there is a non‐significant trend towards higher rates among DC compared with MC pregnancies (adjusted hazard ratio 1.165, 95% CI 0.598‐2.271).

**Conclusion**: In our cohort DC and MC pregnancies did not differ in the occurrence of HTN disorders. Cumulative incidence of HTN disorders during pregnancy showed a trend towards higher rates in DC pregnancies.

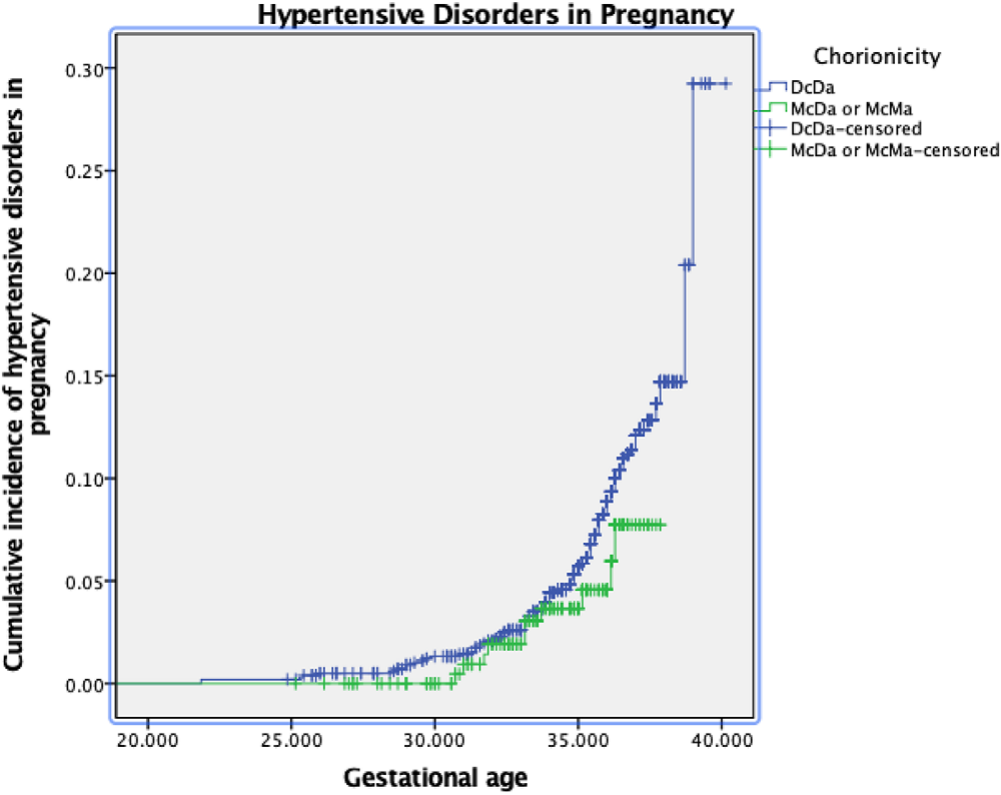

## 327 | What is the accuracy of the ultrasonographic estimated fetal weight over 37 weeks of pregnancy?

Ilenia Paterniti^1^, Calogero Russo^1^, Roberta Granese^1^, Ferdinando Antonio Gulino^2^, Maria Clara Gama^1^, Alfredo Ercoli^3^

^1^University of Messina, Messina, Sicilia; ^2^University of Messina, University Hospital “G. Martino” of Messina, Sicilia; ^3^University of Messina, University Hospital “G. Martino” of Messina, Sicilia

11:45–13:00

**Objective**: The estimated fetal weight (EFW) is challenging if performed beyond 37 weeks of gestation, considering the size of the baby. Our study aims to evaluate the EFW performed beyond 37 weeks of gestation.

**Study Design**: A retrospective observational monocentric cohort study on women with a single‐term pregnancy between 37+0 and 41+0 weeks’ gestation, hospitalized at the University Hospital of Messina (Italy) from September 2023 to April 2024.

The fetal weight was estimated through transabdominal ultrasound (US) according to the first‐trimester dating by fetal crown‐rump length (CRL). The US exams were performed by 4 consultants with training > 5 years in the obstetric US, using a “Samsung WS80A” machine, the day before a planned C‐section or an induction of labour. The EFW was performed using the Hadlock formula, measuring fetal biparietal diameter (BPD), head circumference (HC), abdominal circumference (AC) and femur length (FL). Birthweights (BW) were measured using a “Wunder” Balance with gram as the unit of measure. The statistical analysis consisted of using the paired T‐test and the Pearson correlation coefficient.

**Results**: A group of 102 women were included in this study. The mean value of EFW (**M_EFW_**) was 3232 grams. The mean value of birthweight (**M_BW_**) was 3293 grams. The difference between the two upper cited mean values is **M_BW_–M_EFW_ = 61 grams**. We analyze the relationship between these two means finding a statistically significant concordance (*p*‐value 0,02). We subdivided the obtained values according to the BW into three groups (Group 1 < 3000 grams, Group 2 between 3000 and 4000 grams and Group 3 beyond 4000 grams). We have noticed that over 4000 grams there is a tendency to underestimate the weights while under 3 Kilograms to overestimate. The last cited overestimation was statistically proved (*p*‐value: 0,02).

**Conclusion**: The analysis of our database shows how EFW may be considered accurate also in the late third trimester if properly performed.

## 328 | Risk of uterine rupture during trial of labour following one previous caesarean delivery

Christina Roeck Hansen^1^, Ängla Mantel^2^, Ingela Hulthén Varli^3^, Kari Johansson^1^, Charlotte Lindblad Wollmann^1^

^1^Karolinska Institutet, Karolinska Institutet, Stockholms Lan; ^2^KI, KI/Stockholm, Stockholms Lan; ^3^Department of Women's and Children's Health, Karolinska Institute, Stockholm, Stockholms Lan

11:45–13:00

**Objective**: More than one in five primiparous women in Sweden underwent a caesarean delivery (CD) in 2023, and globally the CD rates increase. A previous CD predisposes to an increased risk of obstetric catastrophic events in subsequent pregnancies and deliveries, such as uterine rupture. There is a discourse within the obstetric community regarding intrapartal factors, such as induction methods, increasing the risk of uterine rupture. The objective of this study is to investigate risk of uterine rupture in women with one previous CD undergoing trial of labour after caesarean (TOLAC) and the association between induction methods and labour management with the risk of uterine rupture.

**Study Design**: A population‐based cohort study using data from the Stockholm‐Gotland Perinatal Cohort, which contains detailed clinical information on antenatal, delivery and postnatal care for all inhabitants of the region (*∼*25% of all deliveries in Sweden). Women with a TOLAC after one previous CD between 2008 and 2020 were included (11947 women). Information on delivery onset, methods used to induce or manage labour dystocia was collected, as well as baseline characteristics, demographic information and comorbidities. Logistic regression models were used to calculate odds ratios (ORs) and adjusted for potential confounders.

**Results**: Of all women with only one previous birth, a CD, 1.8% had a uterine rupture. Induction of labour was associated with an increased risk of uterine rupture, aOR 1.63 (95% CI 1.20‐2.22). Induction with a balloon catheter had no risk of uterine rupture, aOR 0.99 (95% CI 0.61‐1.61), while induction with prostaglandins had an increased risk, aOR 2.58 (95% CI 1.77‐3.74). Induction with prostaglandins had an increased risk of uterine rupture in comparison with balloon catheter, aOR 2.73 (95% CI 1.54‐4.76).

**Conclusion**: Induction of labour with prostaglandins is associated with an increased risk of uterine rupture, while induction using balloon catheter did not increase the risk. When considering TOLAC, induction of labour should be carefully monitored and balloon catheter should be the primary choice.

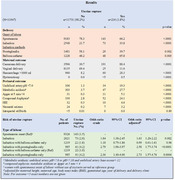

## 329 | Postpartum glucose screening compliance amongst women diagnosed with gestational diabetes in the postpartum period

Miranda Roland^1^, Michael Shu^2^, Michele Fritz^3^, Sakibur Hasan^3^

^1^Henry Ford Wyandotte Ob/Gyn Residency Program, Las Vegas, NV; ^2^Henry Ford Health System, Wyandotte, Michigan; ^3^MSU College of Osteopathic Medicine, East Lansing, Michigan

11:45–13:00

**Objective**: The American College of Obstetricians and Gynecologists (ACOG) recommends screening for diabetes in the postpartum period (PPP), as approximately 50% of women diagnosed with gestational diabetes (GDM) will develop overt disease later in life. Our objective is to assess compliance of postpartum glucose screening (PGS) within a large, urban tertiary care referral center.

**Study Design**: A retrospective chart review was performed between January 2016 and January 2022 after Institutional Review Board (IRB) approval was obtained at a university‐affiliated community hospital in Detroit, Michigan, USA. The primary outcome was compliance of physicians to order postpartum glucose screening (2 hour glucose screening, HgbA1C, or fasting glucose) in those with GDM. The secondary outcome was compliance to complete the screening order between 4‐12 weeks of the PPP. Demographics of age, race, ethnicity, parity, and body mass index (BMI) were recorded. A secondary analysis was performed, stratifying the results based upon diet (A1) versus medication‐controlled (A2) GDM. Data was analyzed together with a graduate‐level data scientist.

**Results**: A total of 3054 women were included in this study who were diagnosed with GDM. Of those, only 34.6% (*n* = 1058) had orders placed for PGS. Of total orders placed, 45.7% (*n* = 484) of them were completed in the PPP. Total compliance rate in accordance with ACOG was 15.8%. There was a statistically significant difference in race and parity amongst orders placed for PGS, and additionally location and ethnicity amongst orders completed in the PPP. A secondary analysis demonstrated a statistically significant preference of PGS orders placed, and subsequently completed, amongst GDMA2 versus GDMA1 patients.

**Conclusion**: Compliance with ACOG PPP recommendations ensures the safe transition of mothers from the gravid state to the postpartum. This study highlights a need for quality assurance and quality improvement focused on mothers previously diagnosed with GDM in the PPP. Physicians should maintain conscious awareness of underlying demographic bias that may impact PGS in select populations.

## 330 | Outcomes in breech presentation at term: A comparison between two third level birth center protocols

Federica Romanzi^1^, Elisa Bevilacqua^2^, Jacques C. Jani^3^, Federica Meli^1^, Andrew Carlin^3^, Giulia Bonanni^4^, Antonio Lanzone^5^, Dominique A. Badr^3^

^1^Fondazione Policlinico Universitario Agostino Gemelli IRCCS, Rome, Italy, Rome, Lazio; ^2^Department of Women, Children, and Public Health Sciences, IRCCS Agostino Gemelli University Polyclinic Foundation, Catholic University of the Sacred Heart, Rome, Lazio; ^3^University Hospital Brugmann, Université Libre de Bruxelles, Brussels, Belgium, Bruxelles, Brussels Hoofdstedelijk Gewest; ^4^Postdoctoral Research Fellow Fetal Care and Surgery Center Division of Fetal Medicine and Surgery Boston Children's Hospital, Harvard Medical School, Boston, Massachusetts; ^5^Fondazione Policlinico Agostino Gemelli, IRCSS, Rome Italy, Rome, Lazio

11:45–13:00

**Objective**: Medical literature supports planned caesarean delivery (CD) for breech presentation at term. Since the publication of the Term Breech Trial (TBT) in 2000, the rate of Vaginal Birth Delivery (VBD) has rapidly declined. However, the long‐term follow‐up of TBT reinforced the protective value of vaginal birth, against infections and other non‐transmissible diseases, leading some authors to support VBD but in strictly selected populations and adopting a specific protocol.

The contradictory of the background still cause a non‐univocal approach. comparing neonatal and maternal outcomes resulting from two different care pathways: patients of Protocol I delivered in a center which routinely offers VBD; patients of Protocol II delivered in a center where breech presentations are routinely assigned to elective CD.

**Study Design**: International retrospective cohort study

**Results**: After matching for confounding factors, 257 pregnancies for each group were analysed (Table 1).

Composite Adverse Obstetrical Outcome (CAOO) was similar in the two groups, but the Composite Neonatal Adverse Outcome (CANO) was statistically significantly higher in patients of Protocol I (*p*‐value < 0.001) especially regarding short‐term, non‐severe neonatal outcomes such as NICU admission (*p* = 0.004), respiratory distress at birth (*p* < 0.001) and the rate of APGAR score < 7 after 5 minutes (*p* < 0.001).

The rate of postpartum blood loss >1000ml was the only secondary outcome significantly higher in Protocol I (*p* < 0.001).

**Conclusion**: This is the first study to directly compare two different management protocols for breech presentations at term.

Short‐term, non‐severe adverse neonatal outcomes were significantly increased in the Protocol I group. This must be balanced against the possible negative impacts of caesarean birth on long‐term infant and maternal health.

Our results are relatively reassuring and currently support continuation of VBD as long as clear management protocol are applied and an appropriate expertise in VBD in breech presentation is achieved.

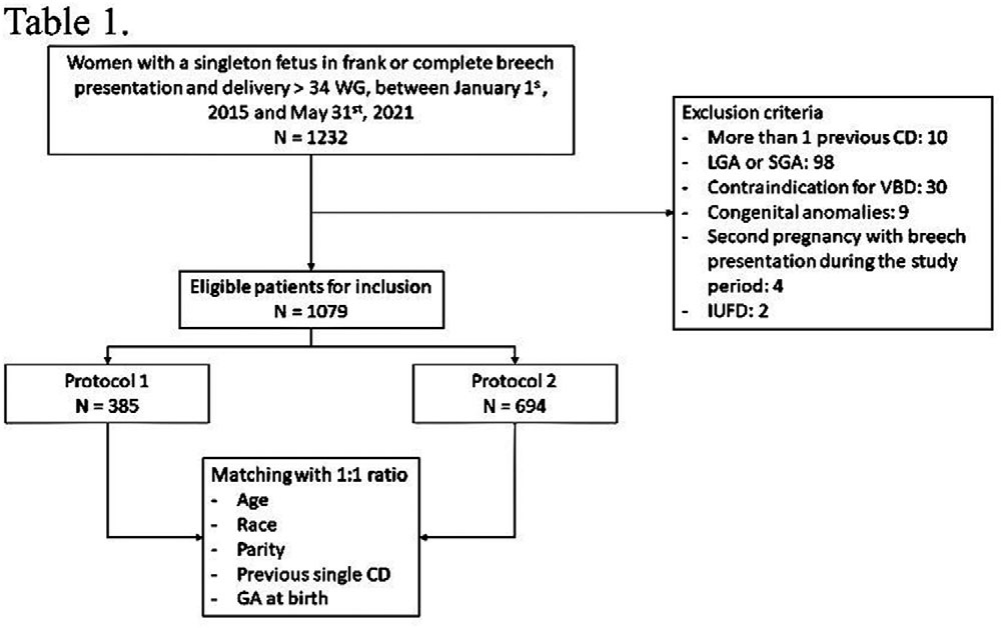

## 331 | Usefulness and trustworthiness of clinical research evaluating tocolytics

Ruxanda Rusu^1^, Helen Vu^2^, Ben W. Mol^3^

^1^Monash University, Warrnambool, Victoria; ^2^Monash University, Monash University, Victoria; ^3^Monash University, Clayton, Victoria

11:45–13:00

**Objective**: Preterm birth is the main cause of perinatal mortality and morbidity. As it is unclear whether and when tocolytic drugs are effective, high quality clinical research is needed. We evaluated usefulness and trustworthiness of published randomised clinical trials (RCTs) on tocolysis.

**Study Design**: We searched the Cochrane library for systematic reviews on tocolytic drugs for preterm labour. From there, we extracted RCTs on the topic. Cochrane reviews were excluded if they reported on a combination of tocolytics, maintenance tocolytic therapy, and prophylactic therapy commenced prior to preterm labour. We limited our analysis to original RCTs with full text English manuscripts.

We assess usefulness and trustworthiness through two predefined checklists with eight and seven criteria, respectively. Data is reported as proportions of studies that fulfil criteria with a 95% confidence interval.

**Results**: We identified 139 RCTs extracted on 24 different tocolytic therapies from 11 Cochrane reviews. The usefulness categories that papers scored most poorly on included: a search for ongoing studies (0/139, 0%), patient centeredness (1/139, 0.7% 95% CI [0.02, 3.9]), new intervention (2/139, 1.4% 95% CI [0.2, 5.1]), value for money (3/139, 2.2% 95% CI [0.5, 6.2]), protocol publication and data sharing (both 4/139, 2.9% 95% CI [0.8, 7.2]). Majority of RCTs scored well on appropriate use of placebo/current treatment (121/139, 87.1% 95% CI [80.3, 92.1]), identifying a problem base (93/139, 66.9% 95% CI [58.4, 74.7]), and identifying a gap in knowledge (83/139, 59.7% 95% CI [51.1, 67.9]).

The trustworthiness categories that papers scored most poorly on included: drop‐out rate of zero (72/139, 51.8% 95% CI [43.2, 60.4]), prospective trial registration (only papers published after 2010) (9/23, 39.1% 95% CI [19.7, 61.5]), and no comment of ethics approval (51/139, 36.7% 95% CI [28.7, 45.3]).

**Conclusion**: The majority of the RCTs evaluating tocolytics for threatened preterm labour have limited usefulness while a substantial number of RCTs have concerns about trustworthiness.

## 332 | Hypomethylation of PGF Gene and Low Expression of PlGF as a Risk Factor for Preeclampsia

Mochammad Imam Santoso^1^, Anak Agung Ngurah Jaya Kusuma^2^, I Made Darmayasa^2^, I Nyoman Hariyasa Sanjaya^2^, I Gede Mega Putra^2^, I Gede Ngurah Harry Wijaya Surya^2^

^1^Prof. Dr. I.G.N.G Ngoerah General Hospital, Denpasar, Bali/Faculty of Medicine Udayana University, Denpasar, Bali; ^2^Prof. Dr. I.G.N.G Ngoerah General Hospital, Denpasar, Bali/Faculty of Medicine Udayana University, Prof. Dr. I.G.N.G Ngoerah General Hospital, Denpasar, Bali/Faculty of Medicine Udayana University, Bali

11:45–13:00

**Objective**: This study aims to investigate the role of epigenetic modifications, specifically DNA methylation, in the pathogenesis of preeclampsia, a life‐threatening condition in pregnancy. It focuses on the methylation status of the placental growth factor (PGF) gene promoter and its impact on placental growth factor (PlGF) expression.

**Study Design**: This study employs an analytical observational design with a case‐control approach. It involves pregnant women diagnosed with preeclampsia (*n* = 30) as the case group and normotensive healthy pregnant women (*n* = 30) as the control group, conducted at the Obstetrics and Gynecology Departement of Prof. Dr. I.G.N.G. Ngoerah Hospital, Bali, Indonesia. DNA methylation of the PGF gene promoter and PlGF expression levels will be assessed in placental tissue samples using PCR analysis. Statistical analysis will be performed to compare DNA methylation patterns in promoter region of PGF gene and PlGF expression between the two groups.

**Results**: Hypomethylation at CpG promoter region was observed in PGF gene in women with preeclampsia compared to normotensive controls. PlGF expression was lower in women with preeclampsia. DNA methylation at CpG in promoter region of PGF gene was negatively correlated with expression of PlGF.

**Conclusion**: We report that lower placental DNA methylation at promoter region of PGF gene, a critical genes involved in angiogenesis, are strongly associated with preeclampsia. We also demonstrate lower PlGF expression between preeclampsia and normotensive controls. Our findings indicate a potential mechanism by which the hypomethylation PGF gene promoter and PlGF expression serves as a biomarker for predicting the risk of preeclampsia. Changes in the methylation of these genes in the placenta could affect angiogenesis, placental growth, and fetal development, potentially increasing the risk of future cardiometabolic disorders in offspring.

## 333 | Self‐reported breast symptom patterns in pregnant and non‐pregnant young women

Moriah Peyser‐Rosenberg^1^, Doron Kabiri^2^, Tanir Allweis^3^, tal hadar^3^, Hen Y. Y. Sela^4^

^1^Hebrew University School of Medicine, Jerusalem, Yerushalayim; ^2^Department of Obstetrics and Gynecology, Hadassah‐Hebrew University Medical Center, Jerusalem, Israel., Jerusalem, Yerushalayim; ^3^Department of Surgery and Breast Health Center, Hadassah and Hebrew University Medical Center, Jerusalem, Israel, Jerusalem, Yerushalayim; ^4^Shaare Zedek, Jerusalem, Yerushalayim

11:45–13:00

**Objective**: Breast symptoms during pregnancy usually represent benign situations. Paucity of data regarding breast symptoms in pregnancy exists, which may lead to delayed diagnosis of Pregnancy‐associated breast cancer (PABC). Our aim was to prospectively collect and compare data regarding breast symptoms in pregnant versus non‐pregnant young women.

**Study Design**: Prospective observational study including young women (18‐40 years old) presenting for obstetric and gynecological reasons unrelated to breast complaints. We excluded women with BC or such history and carriers of BRCA1/2 mutations. A priori sample size estimation was calculated and based on an observation of 18% breast disorders on clinical exam during pregnancy, assuming double symptoms rate and half in non‐pregnant women. A total sample of 94 women in each group was sufficient to detect a >2‐fold difference in breast symptoms. Following IRB approval, women were approached to participate with self‐administered questionnaires.

**Results**: Overall, 372 women completed the questionnaire, 253 pregnant (68%) and 119 non‐pregnant (32%). Prevalence of at least one breast symptom was 26.9% among pregnant and 11.9% in non‐pregnant women (*p* < 0.001). Mastalgia and skin discoloration were significantly more common in pregnant vs. non‐pregnant women (14.5% vs. 6% *p* = 0.019 and 7.2% vs. none *p* = 0.003). Breast lump and nipple discharge were similarly uncommon in both groups (Figure 1).

Multivariate analysis revealed that pregnancy was associated with increased risk of at least one breast symptom (aOR 3.78).

**Conclusion**: Young women commonly have breast symptoms. Pregnancy was an independent risk factor for breast symptoms. Yet prevalence of specific symptoms more suspicious for malignancy (breast lump; nipple discharge) were rare and not affected by pregnancy status. This highlights the importance of thorough evaluation of breast symptoms in pregnant women as is recommended in all women.

Improvement in maternal health requires awareness and research of medical conditions during pregnancy and outside of it however data regarding breast are scarce.

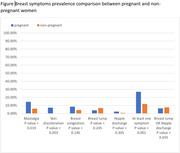

## 334 | The impact of intrapartum oxytocin administration on exclusive breastfeeding success: A prospective cohort study

Roza Berkovitz‐Shperling^1^, reut berkovitz^2^, Yariv Yogev^3^, maya Ram^2^

^1^Tel Aviv Sourasky Medical Center, Tel Aviv Sourasky Medical Center, Tel Aviv; ^2^ichilov, ichilov, Tel Aviv; ^3^Lis Maternity Hospital, Sourasky Medical Center, Tel Aviv University, Tel Aviv Sourasky Medical Center, Tel Aviv

11:45–13:00

**Objective**: As paucity of data exists regarding the potential impact of intrapartum oxytocin administration on breastfeeding we aimed to investigate the potential impact of intrapartum oxytocin on lactogenesis and subsequent breastfeeding outcomes.

**Study Design**: A prospective single‐blind cohort study of women who desired exclusive breastfeeding (EBF) following delivery was performed in a single tertiary university affiliated medical center, with approximately 12,500 deliveries annually. Overall, 372 women following a singleton delivery, were randomly enrolled on postpartum day 2. Exclusion criteria included: preterm birth ( < 37+0 gestational weeks), Cesarean delivery, and medical conditions that contraindicated BF. Women were divided into the two study groups: EBF and non‐exclusive (non‐EBF). Both groups were assessed for the total dose of oxytocin received and various labor and delivery parameters.

**Results**: The mean dosage of oxytocin per minute showed no significant difference between the exclusive and the non‐ exclusive breastfeeding group (3.19 ± 2.68 vs 4.32 ± 5.86, *p* = .146) (Table 1).

Women in the exclusive breastfeeding (EBF) group had shorter durations in the delivery room (459.9 ± 315.6 vs 558.9 ± 368.7, *p* = .007) and from rupture of membranes to delivery (273.9 ± 423.5 vs. 467.2 ± 721.3 min, *p* = .*001*). Additionally, active labor at admission (>4 cm) was more common in the EBF group (81 (51.3%) vs 85 (39.7%), *p* = .*027*). Instrumental delivery was less common in the EBF group (7% vs 13%, *p* = .*054*).

In logistic regression analysis, the only variable found to have a significant positive association with EBF success was BF initiation in the delivery room (aRR = 2.042 95% confidence interval CI [1.198‐3.480]).

**Conclusion**: The administration of intrapartum oxytocin did not have a significant impact on the success of exclusive breastfeeding. Factors associated with higher exclusive breastfeeding rates were labor progression and early breastfeeding initiation.

## 335 | The role of CGH array analysis in fetuses with structural abnormalities: A single‐center cohort study

Filomena G. Sileo^1^, Vincenza Dipace^2^, Giulia Galeati^3^, Licia Lugli^4^, Antonio La Marca^3^, Emma Bertucci^5^, Giovanna Sartor^6^, Mina Grippa^7^, Olga Calabrese^7^

^1^Prenatal Diagnosis, University of Modena and Reggio Emilia, University of modena and reggio emilia, modena, Emilia‐Romagna; ^2^AUSL di Modena, Modena, Emilia‐Romagna; ^3^Azienda Ospedaliero–Universitaria Policlinico di Modena, Obstetrics and Gynaecology Unit, Modena, Emilia‐Romagna; ^4^Neonatal Intensive Care Unit, Women's and Children's Health Department, University Hospital of Modena, Modena, Italy, Modena, Emilia‐Romagna; ^5^Prenatal Medicin Center, Obstetrics and Gynecology Unit, Department of Medical and Surgical Sciences for Mother, Child and Adult, University of Modena and Reggio Emilia, Modena, Italy, Modena and Reggio Emilia University, Modena, Itally, Emilia‐Romagna; ^6^Genetics Department, AUSL Modena, Modena, Emilia‐Romagna; ^7^Genetics Department, Policlinico di Modena, Modena, Emilia‐Romagna

11:45–13:00

**Objective**: to evaluate the role of CGH‐array (aCGH) in parents’ decision‐making process in fetuses with structural anomalies.

**Study Design**: this is a retrospective single‐centre cohort study conducted at Policlinico di Modena (January 2014‐November 2022) including 139 singleton pregnancies with a structural abnormality. Twins or invasive diagnosis performed with normal scans were excluded. The Chi‐square or Fishers' exact tests were used for binary or categorical variables. A *p*‐value < 0.05 was considered statistically significant.

**Results**: Karyotype was abnormal in 10 (7.2%); in 129 (92.8%) the CGHa analysis was performed showing a submicroscopic alteration in 25 (19.4%). In 7 fetuses, the genetic analysis was extended to Rasopathies, being positive in 2, and in 1 a genetic mutation in the panel for skeletal dysplasia was found. Termination of pregnancy (TOP) was chosen by 46 (33.1%) women, 26 (56.5%) in the negative aCGH group, 13 (28.3%) in the positive aCGH group, 7 (15.2%) with standard karyotype abnormalities. TOP was chosen based on the prognosis of the fetal anomaly only by 21 patients. The rates of positive aCGH was higher (50% vs. 19%, *p* = 0.04) among those opting for TOP after waiting for the genetic results. 90 patients (64.7%) carried on the pregnancy: 74 (53.2%) and 11 (7.9%) in the negative or positive aCGH group, respectively; 3 (2.2%) in the group of patients with standard karyotype abnormalities and 2 (1.4%) with a Rasopathy. The rate of pregnancies continued among the negative aCGH group was higher (76.3% vs. 44%, *p* = 0.002). Finally, the rate of positive aCGH did not differ according to the system involved (*p* > .05) but it was significantly higher when 3 systems were involved (16% vs. 2.1%, *p* = 0.003).

**Conclusion**: The aCGH was confirmed to be useful in the patient's decision‐making process not only after a positive result but also being reassuring, when negative, in case of an isolated fetal anomaly with a good prognosis.

## 336 | Physician interruptions: An observational study of daily resident interruptions on labor and delivery

Sierra Treiman^1^, Marisa Griego^2^, Linda R. Chambliss^3^

^1^Creighton University Residency of Obstetrics and Gynecology–Phoenix Campus, Phoenix, AZ; ^2^Creighton University Residency of Obstetrics and Gynecology–Phoenix Campus, Espanola, NM; ^3^Valleywise Medical Center, Phoenix, Arizona

11:45–13:00

**Objective**: The purpose of this study is to quantify how many interruptions residents receive during a typical shift on labor and delivery. We also aim to provide insight into resident physician attitudes towards interruptions.

**Study Design**: This was an observational study. Resident physicians working on labor and delivery at two teaching hospitals in a large metropolitan area were provided with clickers and instructed to count the number of phone calls, pages, and in person interruptions received on their shifts. Data were collected for residents working during the day shift and night shifts at each hospital over a two week period. Residents then completed a survey regarding their attitudes towards interruptions. The mean number of interruptions were calculated and compared to a composite score obtained from questions in the survey. Composite scores indicated positive or negative attitudes towards interruptions.

**Results**: 13 residents participated in this study. The overall mean number of resident interruptions per shift at both hospitals was 15.1. There was a negative correlation between PGY level and number of interruptions, with PGY1 residents receiving the most interruptions, with a mean of 23.9 and 17.5 by hospital. PGY4 residents experienced the fewest interruptions, with a mean of 11.9 and 8.5 interruptions. No correlation was found between number of interruptions and attitude towards interruptions however a non‐statistically significant trend occurred between increasing PGY level and negative attitude towards interruptions.

**Conclusion**: This study demonstrates the high quantity of interruptions residents receive on a daily basis as well as the correlation with attitudes towards interruptions as physicians progress through residency. It suggests that training programs would benefit from developing innovative quality improvement approaches to communication to reduce the number of non‐urgent interruptions in order to enhance patient safety, maximize learner education, and reduce physician burnout.

## 337 | Maternal cardiac morbidities and placental related complications

Tamar Wainstock^1^, Eyal Sheiner^1^

^1^Department of Obstetrics and Gynecology, Soroka University Medical Center, Ben‐Gurion University of the Negev, Beer‐Sheva, Israel., Beer Sheva, HaDarom

11:45–13:00

**Objective**: Cardiac morbidities may impair perfusion and placental vascularization. The aim of the study was to address the association between severe maternal cardiac morbidities and placental‐related complications in pregnancy including preterm deliveries (PTD), intrauterine growth restriction (IUGR), low birthweight (LBW), preeclampsia and placental abruption.

**Study Design**: A retrospective population based cohort study was conducted, including all women who delivered at a tertiary medical center between the years 1991‐2021. A comparison was performed between women with and without severe maternal cardiac morbidities, in terms of placental‐related pregnancy complications. Multivariable generalized estimation equation binary logistic models were used to account for confounding variables.

**Results**: The study included 232,476 deliveries, of them 570 (0.2%) were by mothers with severe cardiac morbidities. These patients were older (30.5 ± 5.8 vs. 28.0 ± 5.8, *p* < .001), more likely to be diagnosed with preeclampsia or eclampsia (4.9% vs. 3.8%, *p* = .014), IUGR (4.0% vs. 1.8%, *p* < .001), placental abruption (1.1% vs. 0.5%, *p* = .066) and their pregnancies were more likely to end preterm (13.0% vs. 7.0%, *p* < .001), and their offspring more likely to be with LBW (12.6% vs. 7.2%, *p* < .001). The multivariable analyses, which accounted for maternal recurrence in the cohort and her age, revealed that mothers with severe cardiac morbidities are 1.80 (95% CI 1.38‐2.35) more likely to delivery preterm; 2.50 (95% CI 1.64‐3.80) more likely to have IUGR and 1.89 (95% CI 1.44‐2.48) more likely to delivery LBW. The association with preeclampsia and placental abruption was not statistically significant in the models.

**Conclusion**: Mothers with severe cardiac morbidities are at higher risk for placental‐related complications in pregnancy. Their pregnancies might be considered as “high risk”, requiring targeted intervention to minimize the risk for adverse outcomes.

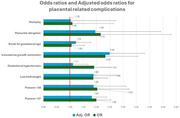

## 338 | A medical home for pregnant victims of sex trafficking

Amy Williamson^1^, Elaine Medeiros^2^, Shauna Sexton^2^

^1^Phoenix Children's, Phoenix, AZ; ^2^Phoenix Dream Center, Phoenix, Arizona

11:45–13:00

**Objective**: This manuscript describes a model of obstetrical care that serves pregnant human sex‐trafficking victims in a clinic on the campus of a residential program for human sex‐trafficking survivors. This long‐term residential program has 48 beds for female victims of trafficking in which up to forty percent of the women entering the program are pregnant.

**Study Design**: This is a descriptive study detailing a unique medical care model in which obstetrical care is provided on‐site clinic at a residential program for victims of sex‐trafficking.

**Results**: Medical care has been provided to trafficking survivors in a clinic on the campus of a residential program since September 2017. In 2023 there were 276 patient visits with 104 visits for obstetrical care in a 4‐hour weekly clinic with an Ob/Gyn physician.

**Conclusion**: There is increasing awareness about sex‐trafficking as a crime, but significant gaps exist in the health care provided to these victims. Since 4.5 million people throughout the world are victims of forced sexual exploitation, sex trafficking is a world‐wide public health issue. While data in this population is lacking, trafficking victims experience high rates of violence, alcohol and drug use, mental health issues, and STIs. All these conditions have been associated with poor obstetric outcomes.

Key factors in this model's success include:

1. Consistent provider since the inception of the clinic.
2. Release of information signed be each patient to allow the provider and the program staff to communicate.
3. Coordinated referrals to MFM and specialists for consults and ultrasounds. Program staff accompanies patients to ensure successful navigation of the health care system.
4. Consistent clinic staff who provide trauma‐informed care in an environment in which patients feel respected and empowered.
5. Support from nursing instructors and students who provide childbirth and breastfeeding support.
6. Support of the health care system that provides care to every patient regardless of ability to pay.

## 339 | Prevalence of congenital anomalies detected through detailed anatomy ultrasound at a referral hospital in Ghana

Alim Swarray‐Deen^1^, Morgan Yapundich^2^, Jerry Coleman^3^, Sarah Boudova^4^, Samuel Oppong^5^

^1^University of Ghana Medical School, University of Ghana Medical School, Greater Accra; ^2^Wake Forest University School of Medicine, Winston‐Salem, NC; ^3^Korle Bu Teaching Hospital, Accra, Greater Accra; ^4^Thomas Jefferson University, Department of Obstetrics and Gynecology, Division of Maternal‐Fetal Medicine, Philadelphia, Pennsylvania; ^5^University of Ghana, University of Ghana, Greater Accra

11:45–13:00

**Objective**: Africa has a high burden of congenital anomalies due in part to limited prenatal care, infections, and environmental exposures. However, the true prevalence of congenital anomalies is unclear because of insufficient access to prenatal diagnostic services, comprehensive neonatal assessments, and postmortem fetal autopsies. We aimed to characterize the anomalies detected prenatally at a referral hospital in Ghana.

**Study Design**: We performed a retrospective chart review of congenital anomalies detected by anatomy ultrasounds completed from 2020 to 2023 at a referral hospital in Ghana. Data were extracted from the electronic medical record on maternal age, gestational age, and congenital anomalies as categorized by ICD‐10 code. Descriptive statistics were performed.

**Results**: The mean maternal and gestational age at the time of ultrasound were 33 (SD 8.5) years and 26 (SD 5.4) weeks, respectively. Of the 4,010 anatomy scans, 290 (7.2%) fetuses had anomalies; most (184, 63.4%) had isolated anomalies. The highest prevalence of anomalies was identified in the Central Nervous (CNS) (131, 45.2%), Genitourinary (GU) (81, 27.9%), and Gastrointestinal (GI) Systems (63, 21.7%). The top CNS anomaly ICD‐10 codes were *Other specified congenital malformations of brain*, (50, 38.2%), most of which were Ventriculomegaly (49 cases), and *Congenital malformations of corpus callosum* (18, 13.7%). The top GU anomaly codes were *Congenital hydronephrosis* (31, 38.3%), and *Congenital posterior urethral valves* (15, 18.5%). The top GI anomaly codes were *Exomphalos* (26, 41.3%), and *Congenital absence, atresia and stenosis of duodenum* (16, 25.4%). Unrelated to an organ system, 18(5.9%) had hydrops and 23(7.5%) had soft markers of aneuploidy.

**Conclusion**: Our work demonstrates the significant burden of congenital anomalies identified by prenatal ultrasound in Ghana. We highlight the feasibility and need to perform anatomy ultrasounds in Africa. Beyond the clinical benefit, these data lay the groundwork for studies to identify the underlying causes of high rates of anomalies and best direct limited resources towards health interventions.

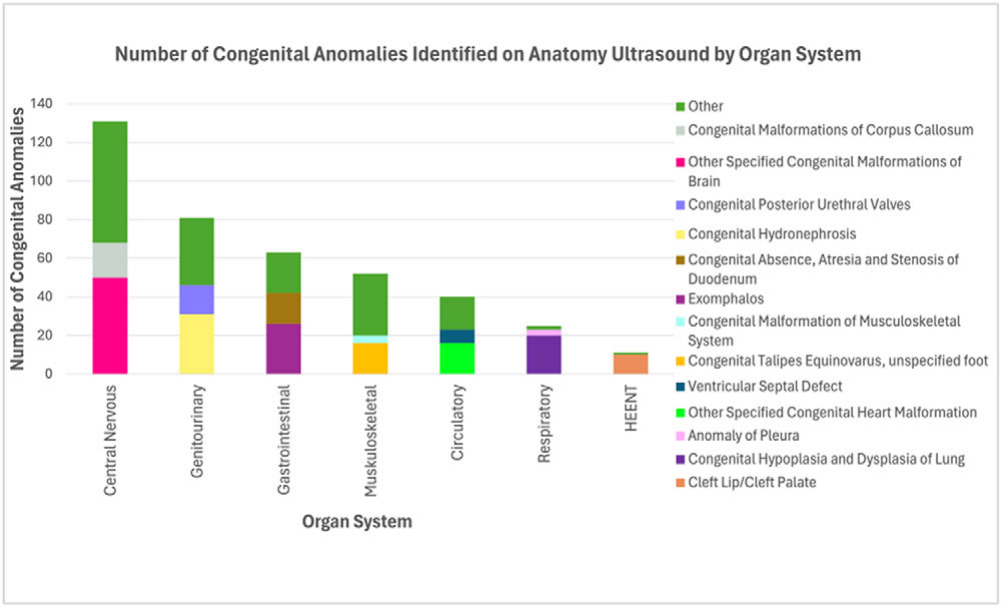

## 340 | Intraperitoneal adhesions prevalence in second cesarean sections: Emergent vs. planned first cesarean — A retrospective study

Fayrooz Yossef^1^, Hanin Dabaja Saqr^2^, Shlomi Sagi^3^, Rami sammour^3^

^1^Bnai Zion Medical Center, Hefa; ^2^Bnai Zion Medical Center, Haifa, Hefa; ^3^Bnai Zion MC, Bnai Zion MC, Hefa

11:45–13:00

**Objective**: To compare intraperitoneal adhesions prevalence in second cesarean sections (CS) between women with prior emergency versus planned cesarean deliveries.

**Study Design**: This is a retrospective cohort study of women undergoing second cesarean sections from January 2021 to March 2023. Exclusions included missing data regarding first or second CS. Primary outcome was the prevalence and severity of intraperitoneal adhesions. Secondary outcomes included extraction time, blood loss and need for relaparotomy.

**Results**: During the study period, a total of 483 women underwent a second cesarean section. 38 cases were excluded due to missing data. Of the remaining cases, 294 had previously undergone an emergency CS, while 151 had a previous planned CS. Background characteristics were similar between the groups (Table 1). In the previous planned‐CS group, 83 women (55%) had no adhesions during the second surgery, while 68 women (45%) presented with adhesions. In the previous emergency‐CS group, 161 women (54.8%) had no adhesions, and 133 women (45.2%) had adhesions. This difference was not statistically significant.

**Conclusion**: Prevalence of intraperitoneal adhesions during repeat cesarean sections did not significantly differ between planned and emergency cesarean cases, suggesting emergency cesareans may not independently increase adhesions formation risk.

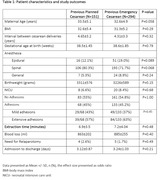

## 341 | Copy Number Variants and Fetal Growth in Fetuses with Normal Karyotypes

Jing Zhu^1^, Xu Han^1^, Yonggang Lu^1^, Fengping Yang^2^, Yan Bi^1^, Yi Wu^1^, Li Gao^1^, Xinrong Zhao^1^, Yiru Shi^1^, Guang He^2^, Jun Zhang^3^, Yanlin Wang^1^

^1^The International Peace Maternity and Child Health Hospital, Shanghai Jiao Tong University School of Medicine, Shanghai, Shanghai; ^2^Bio‐X Institutes, Key Laboratory for the Genetics of Developmental and Neuropsychiatric Disorders, Shanghai Jiao Tong University, Shanghai, Shanghai; ^3^Shanghai Jiao Tong University School of Medicine, Shanghai, Shanghai

11:45–13:00

**Objective**: To evaluate the association between copy number variants (CNVs) detected by chromosomal microarray analysis and extreme fetal growth in fetuses with normal karyotypes.

**Study Design**: This was a retrospective cohort of pregnant women who underwent invasive genetic testing and delivered at ≥ 24 weeks of gestation at a tertiary hospital between January 2015 and October 2022. Pregnancies with multiple gestation, aneuploidy or mosaicism, uniparental disomy, and unknown birthweight were excluded. CNVs were classified into pathogenic/likely pathogenic CNVs, variants of unknown clinical significance (VOUS), and normal CNVs (including no CNVs and likely benign/benign CNVs). Main outcomes were small for gestational age (SGA, a birthweight < 10th percentile for gestational age), severe SGA (a birthweight < 3rd percentile for gestational age), large for gestational age (a birthweight > 90th percentile for gestational age), and macrosomia (a birthweight ≥ 4000g). The association between CNV classifications and study outcomes was examined by Poisson regression models using normal CNVs as referents, with adjustment for potential confounders.

**Results**: A total of 7883 singleton pregnancies were eligible for the analysis. The incidence of pathogenic/likely pathogenic CNVs and VOUS was 1.1% and 3.4% in fetuses with normal karyotypes. Compared with normal CNVs, pathogenic/likely pathogenic CNVs were significantly associated with increased risks of SGA and macrosomia births, with relative risks (RRs) of 2.12 (95% confidence interval [CI] 1.29–3.49) for SGA, 2.92 (95% CI 1.44–5.92) for severe SGA, and 2.06 (95% CI 1.02–4.16) for macrosomia. VOUS was independently associated with increased risks of macrosomia, particularly in fetuses without structural anomalies (RR 1.65, 95% CI 1.07–2.54).

**Conclusion**: Compared with normal CNVs, pathogenic/likely pathogenic CNVs and VOUS were significantly associated with extreme fetal growth in fetuses with normal karyotypes. In addition to prenatal counseling, intensive obstetrical and neonatal care should be provided to those women and their fetuses who carry abnormal CNVs.

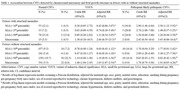

## POSTER SESSION 4

Friday, 27 September 2024 16:45–18:00

## 342 | Breast‐stimulation vs. oxytocin for labor‐augmentation in women with a previous cesarean delivery: A randomized‐controlled trial

Raneen Abu Shqara^1^, Gabriela Goldinfeld^2^, Tikva Assulyn^3^, Inshirah Sgayer^1^, Nadir Ganem^3^, Lior Lowenstein^1^, Maya Frank Wolf^4^

^1^Galilee Medical Center, Nahariya, HaZafon; ^2^Bar‐Ilan university, Bar‐Ilan University, HaZafon; ^3^Raya Strauss Wing of Obstetrics and Gynecology Galilee Medical Center, Nahariya, HaZafon; ^4^Galilee Medical Center, Naharyia, HaZafon

16:45–18:00

**Objective**: The optimal method of labor‐augmentation for women with a prior cesarean‐delivery (CD) has not been established. We aimed to compare outcomes according to the labor‐augmentation method; breast‐stimulation vs. intravenous (IV) oxytocin.

**Study Design**: A prospective, single‐center, randomized controlled trial was conducted. The participants had one previous‐CD, a cervical dilatation of 2‐6 cm and were candidates for labor‐augmentation. Participants were randomized for augmentation by breast‐stimulation using a breast pump, or by an IV low‐dose oxytocin. An intrauterine pressure‐catheter was inserted. Both augmentation treatments were continued for a maximum of 12h. Intervention failure was defined If active labor did not occur within 12h. An intention‐to‐treat analysis was performed. The co‐primary outcomes were augmentation to delivery time, and uterine contraction intensity (Montevideo units). Secondary outcomes included induction, obstetrical and perinatal outcomes.

**Results**: The breast‐stimulation and the IV‐oxytocin group included 33 and 34 women, respectively. The participants’ demographic and obstetric characteristics were similar. Augmentation to delivery interval was longer in the breast‐stimulation than the oxytocin‐group: 10.9 (1.5‐63.2) vs. 5.1 (0.8‐30), *p* < 0.001. The median (range) contraction intensity (Montevideo‐units) was similar between the groups in the first stage of labor, 125 (70‐270) vs. 180 (80‐280), *p* = 0.110. Intervention failure was more prevalent in the breast‐stimulation than oxytocin group, 24% vs. 6%, *p* = 0.044. The tachysystole rate was lower in the breast‐stimulation than the oxytocin group, 6% vs. 27%, *p* = 0.044. Uterine rupture, delivery within 24h of intervention, delivery route and neonatal outcomes were similar between the groups.

**Conclusion**: In women with a previous‐CD, breast‐stimulation was safe and effective, and compared to IV oxytocin caused fewer tachysystole episodes, and with similar vaginal delivery rates and deliveries within 24h. In view of the increase in labor‐augmentation, investigating the role of breast‐stimulation in women with a previous CD is valuable.

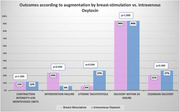

## 343 | Inducing labor after PROM with Oral Misoprostol: Results from the largest cohort in the literature

Adi Ashkenazi Katz^1^, Lior Kashani Ligumsky^2^, Ella Segal^3^, Elad Miron^1^, Ariel J Lasry^4^, Ariel Many^4^, Miriam Lopian^5^

^1^Tel Aviv University, Tel Aviv University, Tel Aviv; ^2^University of California, Los Angeles, Los Angeles, California; ^3^Sheba Medical Center, Tel Aviv University, HaMerkaz; ^4^Department of Obstetrics and Gynecology, Mayanei Hayeshua Medical Center, BNEI BRAK, HaMerkaz; ^5^Mayanei Hayeshua Medical Center, Mayanei Hayeshua Medical Center, Tel Aviv

16:45–18:00

**Objective**: To assess the efficacy and safety of oral misoprostol (OM) for induction of labour (IOL) in patients with Prelabor rupture of membranes (PROM) at term.

**Study Design**: A retrospective cohort was study conducted at a single medical center. The cohort was comprised of patients with PROM at ≥ 37+0 weeks of gestation with an unfavorable Bishop score. Group 1–IOL with OM (50mcg orally every 4 hours) Group 2–IOL with IV Oxytocin.

Primary outcome was vaginal delivery within 24 hours. Secondary outcomes included time from PROM to delivery, time from IOL to delivery, Composite Maternal Outcome(CMO) (Postpartum hemorrhage, Chorioamnionitis, Placental Abruption, Blood Product Transfusion, Uterine Rupture, Endometritis & Postpartum Readmission) and Composite Neonatal Outcome (CNO) (APGAR < 7 at 5 mins, Umbilical Artery pH < 7.1, Infectious morbidity, Respiratory morbidity, NICU admission, Hypoglycemia, Jaundice). Data was analyzed using ANOVA and Chi‐squared tests.

**Results**: 493 patients (62.4%) undergoing IOL with Misoprostol compared to 296 (37.6%) patients receiving IV Oxytocin. Misoprostol had reduced chances of vaginal delivery within 24 (76.3% vs. 87.5%, OR 0.6 (0.4‐0.9), *p* < 0.001) but not 48 hours. They also had a longer interval from PROM (1190 ± 26.7min vs. 856.9 ± 33.6min, *p* < 0.001) and induction 671.6 ± 26.1min vs. 453 ± 27.5min, *p* < 0.001) to delivery but a shorter duration in the labor ward (233.7 ± 10.5 min vs. 471 ± 13.8 min, *p* < 0.001). They were not at increased risk of maternal and neonatal complications. Restricting the analysis to multiparas, there was no difference in the primary outcome.

**Conclusion**: Induction of labor for PROM at term using oral Misoprostol is associated with a high chance of achieving vaginal delivery without increased risks of maternal or neonatal morbidity. While this induction method extends the delivery duration, it decreases the time spent in the labor ward, which has significant benefits for patients and their healthcare providers. This evidence should be integrated into patient counseling to support informed decision‐making about labor induction options.

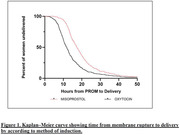

## 344 | The influence of Parity on perineal tears: Results from the largest cohort in the literature

Adi Ashkenazi Katz^1^, Olena Minich^2^, shanny kolp asis^3^, Elad Miron^1^, Ariel J Lasry^4^, Ariel Many^4^, Miriam Lopian^5^

^1^Tel Aviv University, Tel Aviv University, Tel Aviv; ^2^Tel Aviv University, Kfar Saba, HaMerkaz; ^3^Tel Aviv university, Tel aviv, HaMerkaz; ^4^Department of Obstetrics and Gynecology, Mayanei Hayeshua Medical Center, BNEI BRAK, HaMerkaz; ^5^Mayanei Hayeshua Medical Center, Mayanei Hayeshua Medical Center, Tel Aviv

16:45–18:00

**Objective**: To assess the effect of parity on the rate of perineal tears in a cohort of 85,977 patients.

**Study Design**: A retrospective cohort study was conducted between 2011 and 2023 at a single university‐affiliated medical center with a highly parous population. The study group was comprised of patients who had a singleton vaginal delivery at term and no history of previous cesarean delivery. The prevalence of first, second, third, and fourth‐degree perineal tears was calculated for each parity group ranging from 0 to 12. Outcomes were stratified according to the degree of tear‐ combined first and second degree and combined third and fourth degree. Multivariate logistic regression analyses were performed to adjust for including gestational age, delivery mode (spontaneous or operative vaginal delivery) birth weight, and macrosomia (birthweight >4000g).

**Results**: In the study group comprising 85,977 patients, parity (P0‐P12) was distributed as follows: 19,178 (22.3%) women were categorized as P0, 16,250 (18.9%) as P1, 14,158 (16.4%) as P2, 11,225 (13.1%) as P3, 8,093 (9.4%) as P4, 5,780 (6.7%) as P5, 4,069 (4.7%) as P6, 2,885 (3.4%) as P7, 1,876 (2.2%) as P8, 1,256 (1.5%) as P9, 766 (0.9%) as P10, 441 (0.5%) as P11, and 236 (0.3%) as P12.

There was a negative correlation between the number of births (parity) and the incidence of first through fourth degree tears (*p*‐value < 0.001). For first and second degree tears, the incidence was 89.73% in nulliparas (P0), this decreased to 64.98% and 44.24% in P1 and P2, respectively. By P3, the incidence was 32.45%, and this downward trend continues to P12, where it reaches a nadir of 2.12%. A similar trend was noted for the incidence of third and fourth degree tears (1.5% for P0, 0.5% in P1, 0.03% in P5 and none were reported at P6 onwards *p* < 0.0001) (Figure 1). This effect remained significant even when adjusting for the presence of confounders such as birthweight, macrosomia and operative vaginal delivery.

**Conclusion**: The risk of first, second, third and fourth degree tears decreases with increasing parity irrespective of birthweight and mode of delivery.

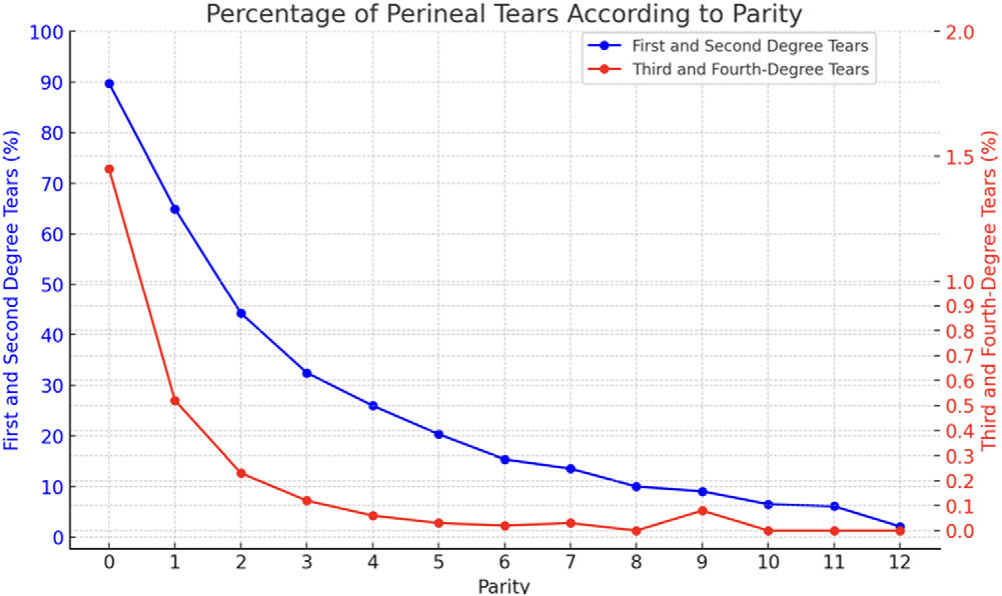

## 345 | The risk of postpartum blood transfusion. Does parity play a role?

Adi Ashkenazi Katz^1^, Olena Minich^2^, shanny kolp asis^3^, Ariel Many^4^, Miriam Lopian^5^

^1^Tel Aviv University, Tel Aviv University, Tel Aviv; ^2^Tel Aviv University, Kfar Saba, HaMerkaz; ^3^Tel Aviv university, Tel aviv, HaMerkaz; ^4^Department of Obstetrics and Gynecology, Mayanei Hayeshua Medical Center, bnei brak, HaMerkaz; ^5^Mayanei Hayeshua Medical Center, Mayanei Hayeshua Medical Center, Tel Aviv

16:45–18:00

**Objective**: To assess the effect of parity on the risk of postpartum blood product transfusion in a cohort of 86,908 patients.

**Study Design**: A retrospective cohort study was conducted between 2011 and 2023 at a single university‐affiliated medical center characterized by a highly parous population. The study groups were comprised of patients who had a delivered a singleton liveborn at term with no history of previous cesarean delivery. The cohort was divided into 3 groups: Group 1:Nulliparas, Group 2:Multiparas (parity 1‐5) and Group 3:Grand multiparas (parity ≥6. The primary outcome was the rate of blood product transfusion. Demographic and obstetric characteristics were compared across all three groups as was the rate of blood product transfusion. Data was analyzed using ANOVA and Chi‐squared tests and multivariate logistic regression analyses were performed to adjust for the presence of potential confounders

**Results**: There were 85, 977 women included in the study group. Group 1 comprised 20,077 (23.4%) patients, Group 2, 55,212 (64.2%) patients and Group 3–11,619 patients (13.5%).

Compering the group we saw significant different in maternal age, birth weight and macrosomia, and for group 1 compare to group 2 and 3 there was higher precantage of cs and operative delivery.

After adjusting for confounders including the mode of delivery, neonatal birthweight and the presence of macrosomia (birthweight >4000g) nulliparas had a significantly increased risk of blood transfusion compared to multiparas (2.7% vs 0.6% adjusted odds ratio (aOR) 0.26, CI 0.22‐0.29, *p* < 0.00001) and grandmultiparas (2.7% vs. 0.8%, 0.30, CI 0.24‐0.38, *p* < 0.00001) however there was no difference in the risk of receiving blood transfusion between multiparas and grandmultiparas (0.6% vs 0.8% 1.21), CI 0.96‐1.53, *p* = 0.112).

**Conclusion**: Nulliparity is an independent risk factor for post‐partum blood product transfusion.

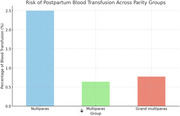

## 347 | Diagnostic criteria for gestational diabetes – Should hypoglycemia also be considered abnormal?

Dafna Ben Yehuda Raz^1^, Rami sammour^2^, Shlomi Sagi^2^, Inna Bleicher^1^

^1^Bnai Zion Medical Center, Haifa, Hefa; ^2^Bnai Zion MC, Bnai Zion MC, Hefa

16:45–18:00

**Objective**: Traditionally, abnormally low glucose levels are not considered part of the diagnostic criteria for GDM on oral glucose tolerance testing (OGTT). Our aim was to compare obstetrical outcomes between patients with a traditional gestational diabetes mellitus (GDM) diagnosis (2 or more high glucose levels on 100g OGTT) and patients with any two abnormal values (high or low) that are not defined as GDM according to traditional diagnosis.

**Study Design**: This is a retrospective cohort study conducted in a single tertiary medical center from 2021‐2022. We included patients with a singleton pregnancy who had a 100 grams oral glucose tolerance testing showing at least 2 abnormal results, either high (according to Carpenter and Coustan criteria) or low (plasma glucose ≤ 70 mg/dL). We compared patients with ≥ 2 high levels, traditionally diagnosed as GDM (2 high group), and patients with any two abnormal values (maximum one high) that are not diagnosed with GDM (other abnormal group). Our primary outcome was the rate of small for gestational age (SGA) and large for gestational age (LGA) infants. Secondary outcomes included mode of delivery, postpartum hemorrhage, neonatal intensive care unit admission, and length of hospital stay.

**Results**: A total of 277 patients were analyzed, 214 in the traditional GDM group and 63 in the other abnormal group. The rates of SGA, LGA, mode of delivery, gestational age, birth weight, and length of hospital stay were similar between the groups (Table 1).

**Conclusion**: Pregnancy outcomes appear to be comparable in patients with the traditional GDM diagnosis (≥ 2 hyperglycemic values) and those with ≥ 2 abnormal values of any kind. Our findings suggest that low glucose level on OGTT should be considered a diagnostic criteria for GDM.

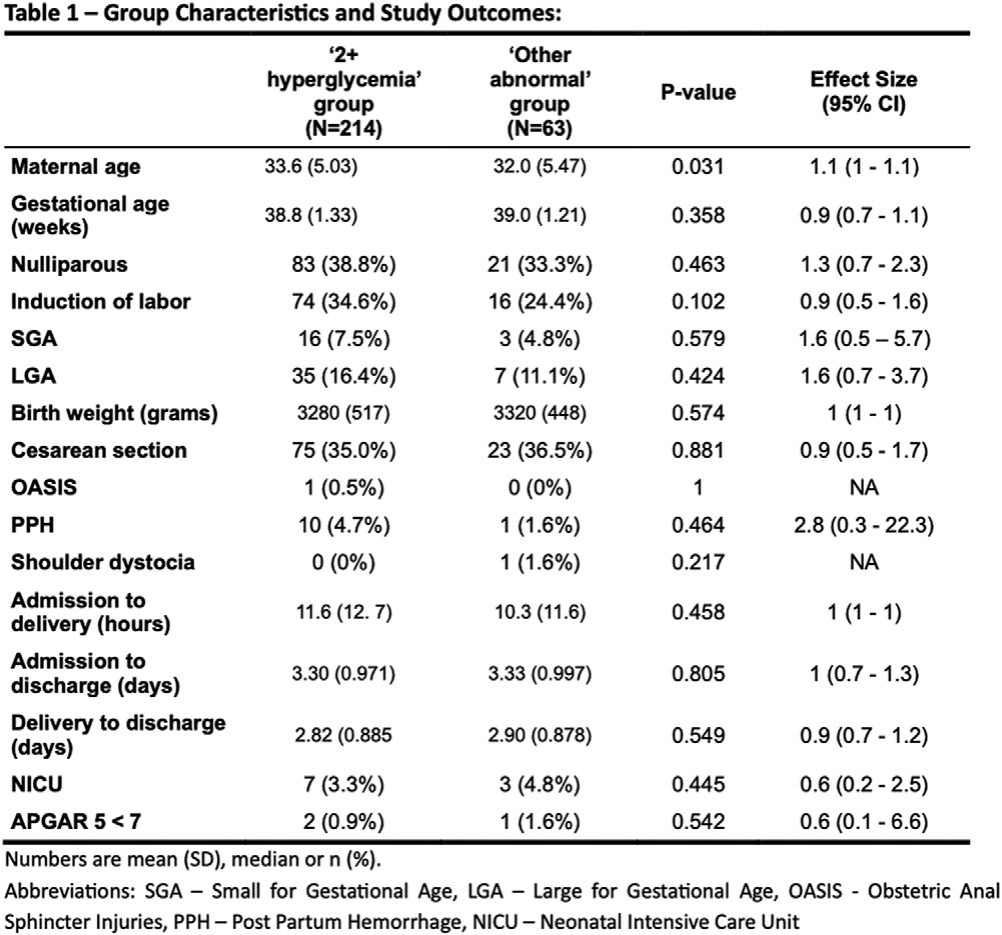

## 348 | Should hypoglycemia be considered as abnormal value when screening for gestational diabetes?

Dafna Ben Yehuda Raz^1^, Rami sammour^2^, Shlomi Sagi^2^, Inna Bleicher^1^

^1^Bnai Zion Medical Center, Haifa, Hefa; ^2^Bnai Zion MC, Bnai Zion MC, Hefa

16:45–18:00

**Objective**: To compare pregnancy outcomes between patients with and without hypoglycemic values during screening for gestational diabetes mellitus (GDM).

**Study Design**: This retrospective cohort study included all patients with singleton pregnancies who delivered in a tertiary university‐affiliated hospital between January 2021 and December 2022 and had 1 or more abnormal values during 100 grams oral glucose tolerance test (OGTT). We compared patients who had only abnormal high values without hypoglycemia (no hypoglycemia group) to patients who had abnormal low values, with or without abnormal high values (hypoglycemia group). Abnormal glucose levels were defined as: ≤ 70 mg/dL (indicating hypoglycemia), GCT > 140 mg/dL, OGTT–fasting ≥ 95 mg/dL, 60‐min ≥ 180 mg/dL, 120‐min ≥ 155 mg/dL and 180‐min ≥ 140 mg/dL. Primary outcome was the rate of small and large for gestational age (SGA and LGA, respectively) babies at delivery. Secondary outcomes included mode of delivery, postpartum hemorrhage (PPH), admission to neonatal intensive care unit (NICU) and duration of hospitalization.

**Results**: A total of 787 patients were included in this analysis, 409 exhibiting hypoglycemia on at least one value, while 378 did not have hypoglycemia at any point. Individuals in the hypoglycemia group were found to be younger, delivered at a later gestational age and higher birthweights at delivery (table 1). The incidence of SGA and LGA babies was similar between the groups. Furthermore, individuals in the hypoglycemia group had a lower rate of cesarean delivery (25.4% vs. 33.9%, *p* = 0.012, OR 0.7 (CI 0.5‐0.9)) and shorter intervals of admission to discharge and delivery to discharge. No significant differences were observed in other maternal and neonatal outcomes between the groups (table 1).

**Conclusion**: Diagnosis of gestational diabetes should be considered on the presence of any dysglycemic values, as the rate of pregnancy complications in comparable between patients with hypoglycemia and those with hyperglycemia only.

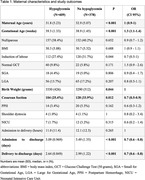

## 349 | Perinatal outcome of fetoscopic laser surgery for TTTS in early gestation

MAR BENNASAR^1^, Julia Ponce^2^, Elisenda Eixarch^3^, Joan Sabrià^4^, Josep Maria Martínez^5^, Eduard Gratacós^6^

^1^BCNatal–Barcelona Center for Maternal–Fetal and Neonatal Medicine (Hospital Clínic and Hospi tal Sant Joan de Deu), IDIBAPS, University of Barcelona, Center for Biomedical Research on Rare Diseases (CIBER–ER) Barcelona, Spain, Hospital Clínic of Barcelona, Catalonia; ^2^BCNatal. Hospital Clínic and Hospital Sant Joan de Déu, Hospital Clínic of Barcelona. Spain, Catalonia; ^3^BCNatal. Hospital Clínic and Hospital Sant Joan de Déu, Hospital Clínic of Barcelona, Catalonia; ^4^BCNatal. Hospital Clínic and Hospital Sant Joan de Déu, Hospital Sant Joan de Déu. Barcelona, Catalonia; ^5^BCNatal. Hospital Clínic and Hospital Sant Joan de Déu, BCNatal. Hospital Clínic and Hospital Sant Joan de Déu, Catalonia; ^6^BCNatal. Barcelona Centre de Medicina Maternofetal i Neonatal. Hospital Clínic and Hospital Sant Joan de Déu, Esplugues de Llobregat, Catalonia

16:45–18:00

**Objective**: To compare the perinatal outcome of early laser surgery for twin‐to‐twin transfusion syndrome (TTTS) performed before 17 weeks of gestation with procedures performed later in pregnancy

**Study Design**: Prospectively evaluation of monochorionic twin pregnancies, complicated by TTTS, treated before 17+0 weeks of gestation during the last ten years in a reference center for fetal therapy. The control group consisted of randomly selected patients undergoing fetoscopic laser (FL) therapy between 17+0‐26+0 week of gestational age during the same period. Patients with a primary intention of laser therapy were included. Exclusion criteria: severe congenital malformations, chromosomal abnormalities and triplets. Selective FL coagulation of placental anastomoses was performed using a 2.0 mm endoscope through an operative sheath of 10 French, while a 7‐8 French sheath was mainly used below 16 weeks gestation. All procedures were performed percutaneously under maternal sedation and local anesthesia and antibiotic prophylaxis.

**Results**: A total of 300 MC pregnancies with complete follow‐up were included in the analysis. The median GA at treatment was 18+2 w (14.5‐25.6), with a median GA at delivery of 33+1 w (24‐41.2). Pregnancy loss occurred in 30 cases (10%). Logistic regression showed significant association between GA at treatment and the occurrence of miscarriage (OR 0.76 (95% CI 0.63‐0.93) *p* = 0.007;) and PPROM (OR 0.81 (95% CI 0.73‐0.89) *p* < 0.0001). The early group consisted in 71 MCDA twins operated before 17+0 weeks of gestation. Results were compared to 229 patients treated after 17w and are presented in Table 1.

**Conclusion**: Early fetoscopic laser surgery is feasible, although associated to an increased rate of adverse perinatal outcome, emphasizing the need for strict criteria definition in selecting candidates for surgery. Nevertheless, when conducted in experienced fetal therapy centers, neonatal survival of at least one fetus occurs in 83% of cases.

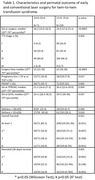

## 350 | Cohen vs Pfannensteil cesarean skin incision for BMI ≥35 kg/m^2^: A randomized controlled trial

Rebekah McCurdy^1^, siani Harding^1^, Laura felder^1^, Vincenzo Berghella^2^

^1^Thomas Jefferson University/Philadelphia, Thomas Jefferson University/Philadelphia, Pennsylvania; ^2^Sidney Kimmel Medical College of Thomas Jefferson University, Philadelphia, Pennsylvania

16:45–18:00

**Objective**: To evaluate the impact of skin incision type at cesarean delivery (CD) in individuals with BMI ≥35kg/m^2^ on maternal morbidity.

**Study Design**: Participants were randomized to either Pfannenstiel or Cohen skin incisions at CD at Thomas Jefferson University Hospital from October 2016 to March 2020. Inclusion criteria were BMI≥35kg/m^2^ undergoing CD between 20‐41 weeks. Traditional Pfannenstiel and Cohen skin incisions were employed, with the choice of sharp or blunt dissection left to the attending surgeon's preference. The primary outcome was a compositive maternal morbidity ≤6 weeks post‐partum, which consisted of the following: wound infection, hematoma, seroma, separation of skin ≥1 cm, readmission for wound complications, endometritis, and postpartum hemorrhage. Statistical analyses included bivariate tests, t‐tests, and non‐parametric analyses.

**Results**: Of 331 elegible individuals, 72 participants with BMI≥35kg/m^2^ and CD completed the trial. Composite maternal morbidity occurred in 47.1% of participants in the Cohen group and 36.8% in the Pfannenstiel group (RR 1.24, 95% CI 0.71‐2.08). Rates of wound infection, hematoma, seroma, wound separation, readmission, endometritis, and postpartum hemorrhage were similar (Table). Neonatal outcomes were also similar, except for a statistically significant lower Apgar score at 5 minutes and a higher need for respiratory support in the Cohen group. Physicians reported significantly lower satisfaction with the Cohen incision compared to the Pfannenstiel incision. Patient satisfaction and likelihood of recommending the incision to a friend did not significantly differ between the groups

**Conclusion**: Cohen and Pfannesteil CD skin incisions in individuals with BMI≥35kg/m^2^ are associated with similar maternal morbidity rates. There are potential benefits in neonatal outcomes and physician satisfaction associated with Pfannenstiel incisions.

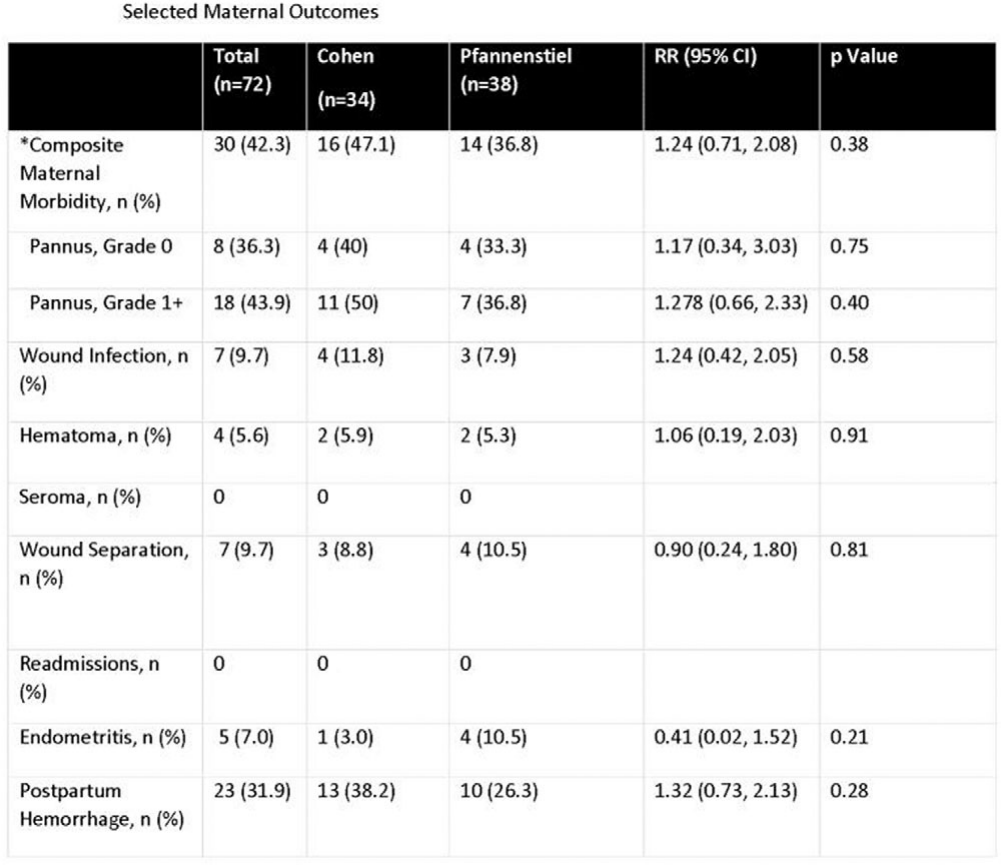

## 351 | Time from dose to delivery and efficacy of maternal azithromycin prophylaxis in planned vaginal births

Rupsa C. Boelig^1^, Avinash Kavi^2^, Janet Moore^3^, Denise Babineau^3^, Waldemar A. Carlo^4^, Alan T. Tita^4^

^1^Sidney Kimmel Medical College, Thomas Jefferson University, Philadelphia, Pennsylvania; ^2^Jawaharlal Nehru Medical College, Belgaum, Karnataka; ^3^RTI, Research Triangle Park, North Carolina; ^4^University of Alabama at Birmingham, Birmingham, Alabama

16:45–18:00

**Objective**: A multi‐center international placebo controlled randomized trial (A‐PLUS), demonstrated that a single dose of 2g oral azithromycin in labor reduced the risk of maternal sepsis and other infections, or death but had no impact on newborn sepsis. We aimed to determine whether efficacy of azithromycin in prevention of maternal or infant infections varied by time from dose to delivery.

**Study Design**: This is a secondary analysis of the A‐PLUS trial. All randomized participants where timing of dose was captured and who delivered within 120 hours were included in this analysis. The primary outcome was any maternal infection and the secondary outcome was any neonatal infection. The estimated relative risks (and 95% confidence interval) of any maternal infection comparing azithromycin to placebo were obtained by fitting a Poisson model adjusting for site, treatment arm, hours between dose administration and delivery (≤12 or >12) and the two‐way interaction between treatment arm and hours between dose administration and delivery. Subgroup analyses were also performed by region (South Asia, Sub‐Saharan Africa) and gestational age (preterm, term). Analyses were repeated for any neonatal infection with a 9‐hour change point variable and 9‐hours as the cut‐point for a categorical variable. Two sided *p* < 0.05 considered significant.

**Results**: Included in the analysis were *n* = 14,569 randomized to azithromycin and *n* = 14,667 to placebo. The interaction *p*‐value between treatment arm and time between dose administration and delivery was non‐significant for both maternal and neonatal infections (*p* = 0.987 and *p* = 0.997 respectively), indicating no evidence that the effect of azithromycin on the risk of composite maternal or neonatal infection was modified by time from dose to delivery. Results were similar across subgroups, but with borderline significance of an interaction in Sub‐Saharan Africa (*p* = 0.053) (Figure).

**Conclusion**: A single prophylactic 2g oral azithromycin dose in labor is effective in prevention of maternal, but not neonatal, infections regardless of time from dose administration to delivery.

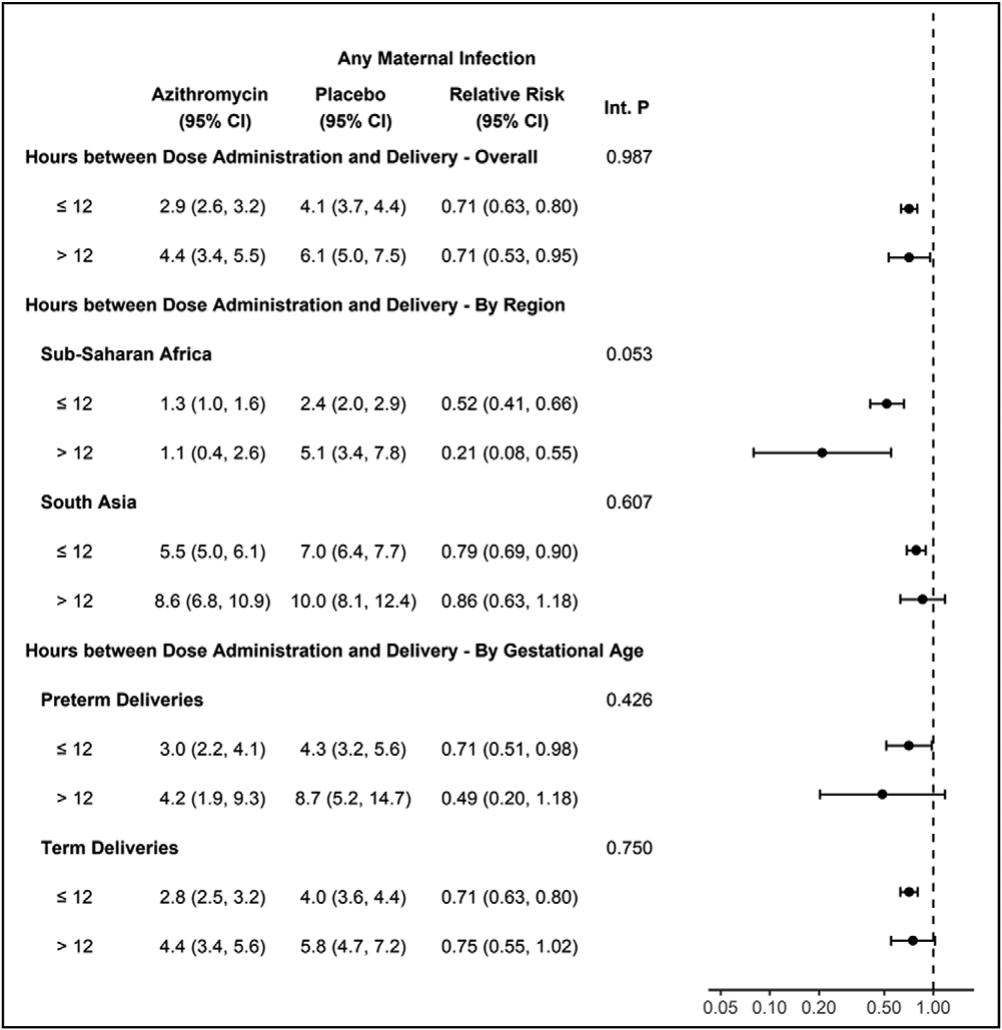

## 352 | Automatic assessment of Midline Angle (MLA) using intrapartum ultrasound

Rocco Morello^1^, Francesco Conversano^1^, Chiara Botrugno^2^, Alberto Bottino^3^, Roberto Franchini^1^, Paola Pisani^1^, Marco Di Paola^1^, Sergio Casciaro^1^

^1^National Research Council (CNR‐IFC), Lecce, Puglia; ^2^National Research Council, Lecce, Puglia; ^3^University of Salento, Lecce, Puglia

16:45–18:00

**Objective**: Fetal head station is clinically measured during the second stage of labor in order to assess descent, identify obstructed labor, and determine how to speed delivery when necessary. In this context, Midline Angle (MLA) is an important index of fetal progression, measured in the axial plane as the angle between the echogenic line separating the two cerebral hemispheres (midline) and the anteroposterior axis of the maternal pelvis. This study aims to assess accuracy and performances of an automatic algorithm for measuring MLA in a routine clinical context.

**Study Design**: Several sessions of B‐mode acquisitions were performed during the active labor phase carried out by 40 women and a total of 106 images of the fetal head were selected.

The algorithm for MLA evaluation works as follows:

1. Image validation, relying on gray level and anatomical features analysis;
2. Raw bone structure research, based on pixel cluster positions and gray value intensities;
3. Fetal head and midline detection, by applying morphological filters and pattern tracking methods;
4. Maternal anteroposterior axis identfication and co‐registration of relative positions;
5. Midline angle calculation, adopting the definition specified above.

The correlation coefficient, the determination coefficient and the mean difference were calculated to evaluate the algorithm performance.

**Results**: A statistically significant correlation was found between the value of MLA automatically measured by the algorithm and the manually segmented one (the “gold standard”): in particular, the Pearson's coefficient was r = 0.99 (*p* < 0.001) and the determination coefficient was R2 = 0.98, with a mean difference of the obtained MLA values compared with the gold standard equal to 1.39° ± 6.18°.

**Conclusion**: The developed algorithm can estimate MLA from translabial sonography images, acquired in the second stage of labor, automatically and with an accuracy comparable to the gold standard. It might be applied to help with accurate fetal position identification and the related decision‐making in labor management.

## 353 | Safety of aortic compression in cesarean delivery to prevent blood loss, a pilot randomized trial

Helena Grobecker^1^, Anette Hein^1^, Ulrika Johannesson^1^, Sophia Brismar Wendel^1^

^1^Karolinska Institutet, Stockholm, Stockholms Lan

16:45–18:00

**Objective**: External aortic compression (EAC) may reduce blood loss in cesarean delivery, but may also affect the kidneys, cause discomfort, and alter the placental circulation in delayed cord clamping. The objective was to assess acceptability and maternal and neonatal safety of preventive EAC in women undergoing elective cesarean delivery.

**Study Design**: This is a pilot randomized open‐label trial. Women with a singleton pregnancy ≥ 34 gestational weeks undergoing elective cesarean delivery were randomly assigned to EAC or not. EAC was performed by the assistant surgeon immediately after the baby was born. Maternal acceptability was assessed by numeric rating scales 0‐10 of intraoperative pain, breathing discomfort, nausea, and overall experience. Maternal safety was assessed by S‐creatinine and glomerular filtration rate (GFR) postoperative day 1‐2, and any anesthesiologic complications. Neonatal safety was assessed by umbilical pH, pO_2_, and pCO_2_, Apgar score, need for neonatologist support, and neonatal intensive care admission. Groups were compared according to intention‐to‐treat.

**Results**: In total, 149 women were included. Of 76 allocated to EAC, all received EAC. Of 73 women allocated to no compression, one (1.4%) received EAC. There was no significant difference in intraoperative pain between the intervention group and the comparison group (median 0 (range 0‐8) and 0 (0‐8), *P* = 0.40), respiratory discomfort (median 0 (0‐7) and 0 (0‐7), *P* = 0.44), nausea (median 2 (0‐9) and 3 (0‐10), *P* = 0.43), or overall experience (median 9 (3‐10) and 9 (3‐10), *P* = 0.56). There was no significant difference in mean S‐creatinine between the intervention group and the comparison group (51.0 µmol/L [standard deviation, SD, 10.8] and 53.0 [9.8] µmol/L, *P* = 0.26) or mean GFR (89.2 [2.8] ml/min and 89.3 [2.7] ml/min, *P* = 0.91). No significant anesthesiologic complications were recorded. Neonatal outcomes did not differ significantly between groups (see table).

**Conclusion**: External aortic compression to prevent blood loss in women undergoing elective cesarean delivery was acceptable for the mother, and safe for the mother and neonate.

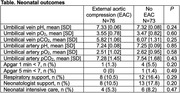

## 354 | Efficacy and Safety of Late Intra‐Uterine Blood Transfusions in Managing Fetal Anemia

Adva Cahen Peretz^1^, Lilah Tsaitlin‐Mor^1^, Gideon Leibner^1^, Sarah Cohen^2^, Danielle Amosi Victor^1^, Nitsan Haham^1^, Tomer Shwartz^1^, Nili Yanai^1^, Shay Porat^1^, Simcha Yagel^3^, Dan Valsky^1^

^1^Hadassah medical center, jerusalem, Yerushalayim; ^2^Washington University in St. Louis, St. Louis, Missouri; ^3^Hadassah Medical Center, Faculty of Medicine, Hebrew University, Yerushalayim, Yerushalayim

16:45–18:00

**Objective**: To assess the safety and efficacy of intrauterine blood transfusions (IUT) administered after 34 weeks of gestation on obstetrical and neonatal outcomes, comparing these to outcomes from earlier interventions (last IUT before 34 weeks of gestation).

**Study Design**: A retrospective cohort study at Hadassah Medical Center reviewed data from pregnant women who received IUT for fetal anemia between 2006 and 2023. We compared outcomes between those receiving late IUT (after 34 weeks gestation) and those treated before 34 weeks, the current standard practice.

**Results**: The study included 50 patients, with 20 undergoing late IUT and 30 receiving their last IUT before 34 weeks of gestation. Controlling for maternal age, parity, occurrence of IUT in previous pregnancies, presence of hydrops in previous pregnancies, and gestational age at the first IUT, late IUT showed an independent odds ratio favorably impacting preterm labor, emergency cesarean section, low birth weight, and NICU admission. Rates of additional neonatal treatment for alloimmune anemia, such as phototherapy, top‐up blood transfusion, and IVIG, were similar between the groups. No cases of fetal or neonatal death occurred in the late IUT group.

**Conclusion**: Extending intrauterine treatment for fetal anemia beyond 34 weeks is a safe and effective strategy for delaying birth and reducing the obstetrical and neonatal complications associated with prematurity. These findings offer significant insights into the optimal timing of IUT for managing fetal anemia, potentially influencing clinical practice.

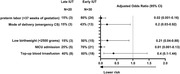

## 355 | Insights from Israeli National Data on COVID‐19 Related Preterm Labor and Preeclampsia

Adva Cahen Peretz^1^, Joshua Guedalia^1^, Michal Lipschuetz^2^, Sarah Cohen^3^, Gideon Leibner^1^, Simcha Yagel^4^, Ronit Calderon Margalioth^5^, Ofer Beharier^1^

^1^Hadassah medical center, jerusalem, Yerushalayim; ^2^Henrietta Szold Hadassah Hebrew University School of Nursing in the Faculty of Medicine, Henrietta Szold Hadassah Hebrew University School of Nursing in the Faculty of Medicine, Yerushalayim; ^3^Washington University in St. Louis, St. Louis, Missouri; ^4^Hadassah Medical Center, Faculty of Medicine, Hebrew University, Yerushalayim, Yerushalayim; ^5^SZMC, SZMC, Yerushalayim

16:45–18:00

**Objective**: This study aims to evaluate the impact of COVID‐19 vaccination on the occurrence of preterm labor and preeclampsia within 28 days following a confirmed COVID‐19 infection during pregnancy. This timeframe is chosen based on established literature standards that suggest complications within 28 days of infection strongly indicate a causal relationship, enhancing our understanding of the potential protective effects of vaccination against these critical obstetric complications.

**Study Design**: This retrospective study utilizes data from the Israeli National Birth Registry, spanning January 2020 to January 2023. Employing descriptive statistics, we assessed the impact of maternal COVID‐19 infection and vaccination status on the incidence of preterm labor and preeclampsia.

**Results**: Our study encompassed a cohort of 527,714 women who delivered in Israel during the analysis period. Of these, 73,095 had a documented COVID‐19 infection. The risk of preterm labor within 28 days of infection rose to 11%, up from a background risk of 5.4% during the pandemic. Among vaccinated women, who received between 1 and 3 vaccine doses, this risk was significantly reduced to between 6.6% and 7%. In contrast, the risk of preeclampsia after infection increased to 3%, from a baseline risk of 2.1%; notably, vaccination status did not mitigate the risk of preeclampsia (Figure).

**Conclusion**: Our findings reveal that while COVID‐19 vaccination significantly reduces the risk of preterm labor within 28 days following infection in pregnant women, it does not similarly mitigate the risk of preeclampsia. These findings highlight the importance of vaccination to reduce the risk of COVID‐19‐related preterm labor and point to the need for further research into preventive strategies for preeclampsia in the context of COVID‐19.

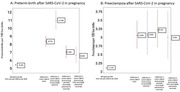

## 356 | Antepartum versus postpartum risk factors for pulmonary edema in women with preeclampsia

Khalil M. Chahine^1^, Baha M. Sibai^2^, Farah H. Amro^2^

^1^McGovern Medical School at UTHealth, Houston, Texas; ^2^McGovern Medical School at UTHealth Houston, Houston, Texas

16:45–18:00

**Objective**: To compare risk factors for pulmonary edema in women with preeclampsia that develops in the antepartum period versus that occurring postpartum.

**Study Design**: This was a retrospective cohort study of individuals with a diagnosis of preeclampsia and pulmonary edema in two academic medical centers in Southeast Texas, from January 2020 to July 2023. Individuals were categorized based on the timing of pulmonary edema diagnosis: prior to delivery (antepartum/intrapartum) versus postpartum. Maternal baseline characteristics and outcomes were evaluated.

**Results**: A total of 71 individuals were included in our study. 27 (38%) developed pulmonary edema in the antepartum period and 44 (62%) in the postpartum period. Both groups had similar baseline maternal characteristics (Table 1).

In the antepartum group, gestational age at time of delivery was significantly lower (29.6 weeks versus 35.1 weeks, P value < 0.001), with a significantly higher rate of preterm birth (100% versus 55%, P value < 0.001). The antepartum group was also more likely to be on long‐acting antihypertensive medications prior to diagnosis of pulmonary edema (70% versus 43%, P value < 0.05). The proportion of patients who received a blood transfusion prior to diagnosis of pulmonary edema was significantly higher in the postpartum group (23% versus 0%, P value < 0.05). In cases where BNP was collected, values >100 pg/mL were seen more frequently in the postpartum group (95% versus 63%, P value < 0.05). No differences in complications related to pulmonary edema were noted between the groups nor in their management, as evidenced by the similar level of respiratory support needed and frequency of furosemide use.

**Conclusion**: Compared to individuals with preeclampsia with postpartum pulmonary edema, patients with preeclampsia with antepartum pulmonary edema are more likely to be on long‐acting antihypertensive medications prior to diagnosis and to deliver prematurely. On the other hand, those with postpartum pulmonary edema are more likely to have had a blood transfusion prior to diagnosis and to have an abnormal BNP.

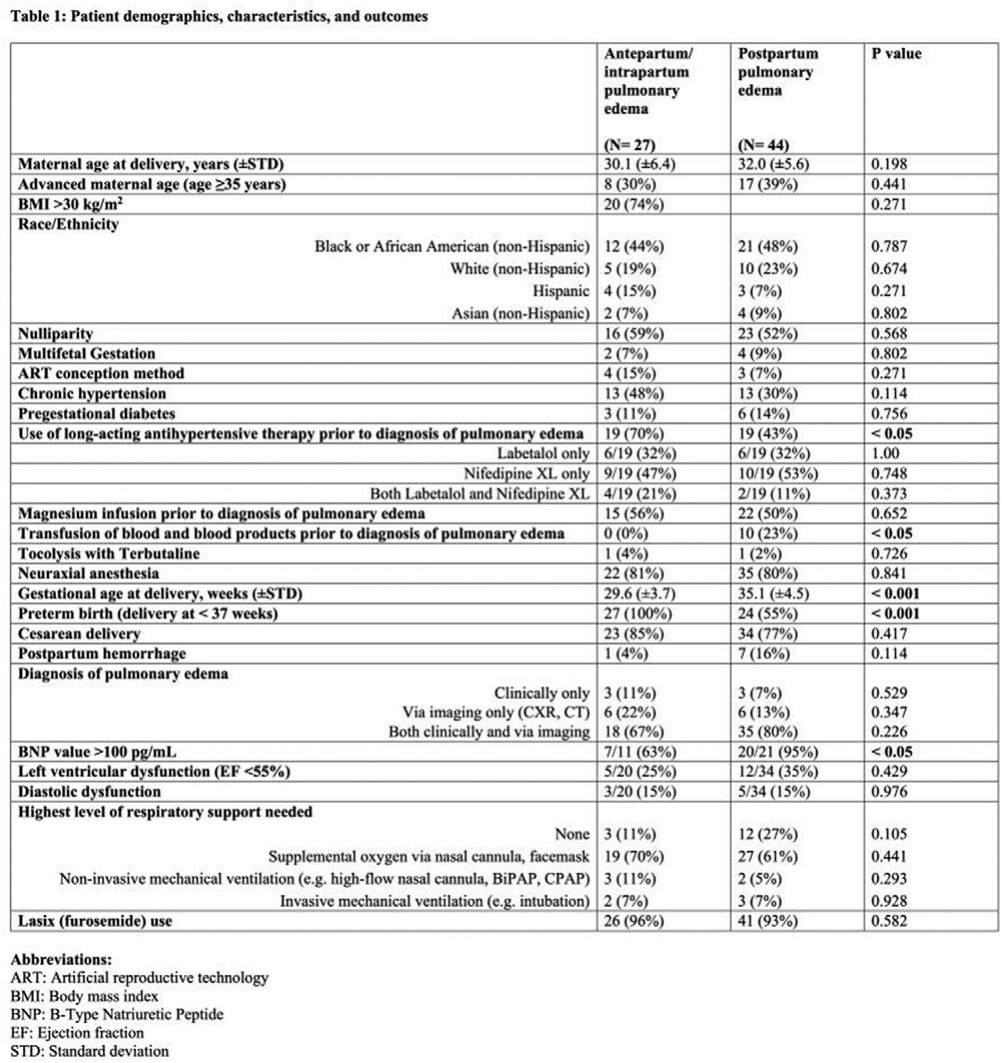

## 357 | Maternal and perinatal outcomes of subcapsular liver hematoma in preeclampsia: A report of 13 cases

Khalil M. Chahine^1^, Megan Shepherd^2^, Sean C. Blackwell^3^, Baha M. Sibai^3^

^1^McGovern Medical School at UTHealth, Houston, Texas; ^2^University of Texas Health Science Center at Houston (UTHealth), University of Texas Health Science Center at Houston (UTHealth), Texas; ^3^McGovern Medical School at UTHealth Houston, Houston, Texas

16:45–18:00

**Objective**: Subcapsular liver hematoma (SLH) in pregnancy is an extremely rare condition, occurring in less than 2% of pregnancies complicated by HELLP. The aim of this study is to review 13 recent cases managed by the senior author, and describe the presenting signs and symptoms, laboratory findings, obstetrical outcomes, management options, and follow‐up, including outcomes in subsequent pregnancies.

**Study Design**: This was a retrospective case series of individuals with SLH managed at a Level IV center between 2013‐2024. Presenting signs and symptoms, laboratory findings, time of onset, management strategies, acute perinatal and maternal outcomes, and long‐term outcomes including subsequent pregnancies, were reviewed.

**Results**: Between 2013 and 2024, there were 13 cases of SLH associated with preeclampsia/eclampsia/HELLP. 4 (30.8%) had chronic hypertension and 11(84.6%) had HELLP syndrome. 11 (76.9%) delivered prematurely, most commonly via Cesarean delivery. The most common presenting symptoms were epigastric and/or right upper quadrant pain (53.8%), followed by abdominal distention (38.5%). Diagnosis of SLH was made in the antepartum period in 6 individuals (46.2%) and postpartum in 7 (53.8%). The diagnosis was confirmed in all cases by computed tomography. Conservative management alone with transfusion of blood and blood products was sufficient in 11 (84.6%) cases, and 2 patients required laparotomy. The mean duration of hospital stay was 10 days (2‐22 days). There were no maternal deaths, however maternal complications such as pleural effusions, pulmonary edema, and acute kidney injury were common. Perinatal mortality rate was 33.3%. 5 individuals had subsequent pregnancies, all 5 had preterm birth, 2 had subsequent HELLP, and one had recurrent SLH.

**Conclusion**: SLH is a rare complication of HELLP syndrome that is associated with substantial maternal and perinatal morbidities. Conservative management with hemodynamic monitoring in association with transfusion of blood and blood products is safe and sufficient in most cases.

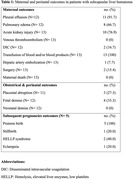

## 359 | Planned Cesarean Delivery Versus Attempted Labor in Low‐Risk Pregnancies: Associations with Adverse Outcomes

Claudia J. Ibarra^1^, Rachel L. Wiley^2^, Han‐Yang M. Chen^3^, Hector M. Mendez‐Figueroa^1^, Suneet P. Chauhan^4^

^1^McGovern Medical School at UTHealth, Houston, Texas; ^2^University of California San Diego, San Diego, California; ^3^McGovern Medical School at UTHealth Houston, Houston, Texas; ^4^Delaware Center of Maternal‐Fetal Medicine at Christiana Care, Delaware, Delaware

16:45–18:00

**Objective**: The primary objective was to compare the composite neonatal adverse outcomes (CNAO) among low‐risk pregnancies that had planned cesarean (PL‐CD) versus those who labored and either had a vaginal delivery or an intrapartum cesarean (IN‐CD; the secondary objective was to compare the composite maternal adverse outcomes (CMAO) among the groups.

**Study Design**: Our population‐based study utilized U.S. vital statistic data from 6 years (2016‐2021) to ascertain the CNAO and CMAO among individuals who delivered at a hospital, 37 to 41 weeks, with non‐anomalous, singletons neonate in the absence of hypertensive disorders, diabetes, or prior cesarean delivery. The main exposure variable was labor status, which was grouped as: no labor (PL‐CD) and had labor. Multivariable Poisson regression models were utilized to estimate adjusted relative risk (aRR) and 95% confidence intervals (CI).

**Results**: Of the 22.6 million live births during the 6 years, 13.6 million (60%) met the inclusion criteria and among these low‐risk pregnancies, 0.8 million (6%) had PL‐CD. Among the 12.8 million that labored, 1.3 million (10%) had an IN‐CD. Overall, the rate of CNAO was 7.9 per 1000 live births and the risk was 42% lower (aRR 0.58; 95 CI 0.57‐0.59) among neonates delivered by those who labored versus those who had a PL‐CD; the overall rate of CMAO was 3.17 per 1000 live births and the risk was 38% lower (aRR 0.62; 95% CI 0.60‐0.64) among those that labored. A sensitivity analysis indicates that compared to PL‐CD, the risk of CNAO was 53% lower (aRR 0.47; 95% CI 0.46‐0.48) among neonates that were delivered vaginally, but 24% higher (aRR 1.24; 95% 1.21‐1.26) if delivered by IN‐CD. The risk of CMAO was 55% lower (aRR 0.45; 95% CI 0.44‐0.47) when delivered vaginally, and 75% higher if had IN‐CD (aRR 1.75; 95% CI 1.69‐1.81).

**Conclusion**: Among low‐risk pregnancies, individuals who labored had a significantly lower risk of neonatal and maternal adverse outcomes than those who underwent PL‐CD Countries with high‐rate of PL‐CD should examine the rate of adverse outcomes among those who labor versus those who do not.

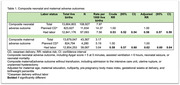

## 360 | Skin‐to‐Skin contact after delivery and postpartum hemorrhage among term vaginal births

Rachel L. Wiley^1^, Ipsita Ghose^2^, Hector M. Mendez‐Figueroa^3^, Suneet P. Chauhan^4^

^1^University of California San Diego, San Diego, California; ^2^Baylor College of Medicine, Houston, Texas; ^3^McGovern Medical School at UTHealth, Houston, Texas; ^4^Delaware Center of Maternal‐Fetal Medicine at Christiana Care, Delaware, Delaware

16:45–18:00

**Objective**: Mother and infant skin‐to‐skin contact (SSC) has benefits physiologically and psychologically immediately after delivery and is associated with oxytocin release which may prevent postpartum blood loss. This study examined rates of postpartum hemorrhage and related morbidity among term (≥ 37 weeks), singleton vaginal births who did versus did not have immediate SSC.

**Study Design**: This study was a retrospective cohort of all births at a Level IV center in 2 years. Exclusion criteria included preterm deliveries, cesarean delivery, fetal anomalies and multiple pregnancies. Individuals were identified as having SSC if they had at least 15 minutes of direct SSC initiated within the first 15 minutes after birth. Data was abstracted by qualified medical personnel and a composite maternal hemorrhagic outcome (CMHO; Table 1) was compared among those with and without SSC. Multivariate Poisson regression with robust error variance was used to examine CHMO to calculate relative risks and adjusted for possible cofounders, and blood loss was compared using students t‐test.

**Results**: Of 8,623 deliveries, 3,485 (40.4%) vaginal births met inclusion criteria and among them, 2,892 (83%) received SSC and 593 (17%) did not. The average blood loss in the SCC care group (288 ± 261 mL) was significantly less than in the no SCC group (366 cc ± 452 mL; *p* < 0.05). The CMHM was significantly less the SSC group (15.2%) than with no SSC (21.8%; aRR 0.75, 95% CI 0.62‐0.92), driven by decreased blood loss (aRR 0.46, 95% CI 0.28‐0.75) and uterotonic use (aRR 0.75, 95% CI 0.61‐0.93). Transfusion and use of mechanical tamponade for hemorrhage were not significantly different, and other components were too infrequent to calculate.

**Conclusion**: Compared to no SSC, immediate skin to skin contact with term vaginal deliveries, was associated with a significant reduction in the likelihood of postpartum hemorrhage. Early skin‐to‐skin contact may be an intervention that can mitigate the postpartum hemorrhagic morbidities in developing countries.

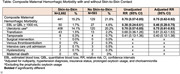

## 361 | Changes in cause‐specific maternal mortality in Western Tanzania during 2013‐2022: A trend analysis

Jessica S. Chung^1^, Florina Serbanescu^2^, Abdulaziz Msuya^3^, Alicia Ruiz^4^, Sunday A. Dominico^3^, Ahmad M. Makuwani^5^

^1^Medical College of Wisconsin, Milwaukee, Wisconsin; ^2^Centers for Disease Control and Prevention, Atlanta, GA; ^3^Tanzania Ministry of Health, Dar es Salaam, Dar es Salaam; ^4^Centers for Disease Control and Prevention, Atlanta, Georgia; ^5^Division of Reproductive, Maternal and Child Health; Tanzania Ministry of Health, Dodoma, Dodoma

16:45–18:00

**Objective**: Kigoma Region in Western Tanzania had some of the greatest needs for improving maternal/neonatal health services and outcomes. The Program to Reduce Maternal Deaths was implemented between 2006 and 2019 in 3 phases—1: 2006–2012, limited; 2: 2013–2015, expanded; 3: 2016–2018, established—to improve and sustain the availability of, access to, and demand for high‐quality maternal health care services. Program activities transitioned to the Tanzanian government in mid‐2019. A sustainability evaluation was conducted in 2023 to document changes in maternal/newborn health and services after the program ended. The aim of this study was to analyze the trends of cause‐specific maternal mortality and facility‐based care indicators during and after the program implementation.

**Study Design**: We used pregnancy outcome monitoring and Rapid Ascertainment Process of Institutional Death methodology to extract, verify, and triangulate data on maternal deaths from all available sources in all facilities providing maternity care; we calculated period cause‐specific maternal mortality ratios (MMRs) per 100,000 live births, case fatality rates, institutional delivery and cesarean section rates for 2013–2015; 2016–2018; 2019–2022 and used z‐test for rates to assess significance of change.

**Results**: Over periods 1, 2 and 3, institutional delivery rates rose from 49.8% to 71.6% to 93.5%; cesarean deliveries increased from 3.0% to 4.1% to 6.2%. Case fatality rates of obstetric complications fell from 1.7% to 1.5% to 1.1%. MMR fell from 286 to 220 to 147 maternal deaths per 100,000 live births, declining by 49% from period 1 to period 3 and 33% from period 2 to 3. Direct‐obstetric MMRs fell by 45% and 28% in those periods, respectively, consistent with continuous declines in sepsis‐ and abortion‐related MMRs.

**Conclusion**: The overall declining trends in maternal mortality continued after the program ended attesting to the long‐term regional impact and sustained in‐country efforts to reduce maternal deaths through improved access to and availability of quality emergency obstetric care.

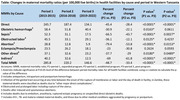

## 363 | The cervical sliding sign: A new ultrasound tool in the assessment of threatened preterm labor

Nicola Volpe^1^, Enrico Corno^2^, Andrea Dall'asta^3^, Giovanni Morganelli^4^, Alessia Casciaro^5^, Elvira Di Pasquo^4^, Tullio Ghi^6^

^1^UOC Medicina Fetale, Ospedale Di Venere, ASL Bari, Bari, Puglia; ^2^University of Parma, University of Parma, Emilia‐Romagna; ^3^Department of Medicine and Surgery, Obstetrics and Gynecology Unit, University of Parma, Department of Medicine and Surgery, Obstetrics and Gynecology Unit, University of Parma, Emilia‐Romagna; ^4^Azienda Ospedaliero‐Universitaria di Parma, Parma, Emilia‐Romagna; ^5^Università di Parma, Università di Parma, Emilia‐Romagna; ^6^Dipartimento di Medicina e Chirurgia, Università di Parma, Parma, Emilia‐Romagna

16:45–18:00

**Objective**: To assess the impact of the cervical sliding sign in case of threatened preterm labor (TPL)

**Study Design**: Single centre prospective study. A non‐consecutive series of pregnant women between 24+0 and 36+6 weeks presenting with uterine contractions were assessed by transvaginal ultrasound to obtain cervical length measurement. Threatened preterm labor was defined as evidence of > 6 contractions/hour + cervical length modifications at the digital examination. The cervical sliding sign was defined as the sliding of the anterior cervical lip on the posterior one under gentle pressure of the TV probe. After the initial evaluation, the time‐to‐delivery (TtD–days) was recorded in each case. In case of cervical length >30mm the patient was discharged, whilst if cervical length was 20‐30 mm, the management depended on the fibronectin test result. The main outcomes were: delivery before 34wks, within 7days and 14 days, TtD.

**Results**: We recruited 146 patients for the study purpose. Of these, 23 delivered before 34 weeks (15.8%) and the average time‐to‐delivery was 41 (20‐58) days. The cervical sliding sign was negative in all but 4 of the 60 cases with cervical length >20mm and was positive in 7 of the 13 cases with cervical length < 10mm. In the subgroup of remaining 73 patients with cervical length between 10 and 20mm the cervical sliding sign was found in 37.0%, and was associated with a shorter TtD (10[5‐37.5] vs 44[34.3‐65.5], *p* < .001) and with a higher risk of delivery < 34wks (9/27 or 32.2% cervical sliding sign positive vs 4/46 or 8.7% cervical sliding sign negative, *p* = 0.008), < 7days (12/27 or 44.4% vs 4/46 or 8.7%, *p* < .001) and < 14days (16/27 or 59.3% vs 5/46 or 10.9%, *p* < .001).

**Conclusion**: In case of threatened preterm labor, the cervical sliding sign seems to be associated with a higher risk of preterm delivery in the subgroup of patients with cervical length 10‐20mm.

## 364 | Patient‐level predictors of accessing care for vaginal bleeding in early pregnancy

Efe E. Cudjoe^1^, Emma Gilmore^2^, Nathanael C. Koelper^3^, Alice Abernathy^1^, Courtney Schreiber^3^

^1^Hospital of the University of Pennsylvania, Philadelphia, Pennsylvania; ^2^University of Pennsylvania, Philadelphia, PA; ^3^University of Pennsylvania, Philadelphia, Pennsylvania

16:45–18:00

**Objective**: To determine whether Rhesus type, pregnancy history, neighborhood socioeconomic disadvantage, and other patient‐level characteristics influence whether patients seek care for first trimester bleeding.

**Study Design**: This was a secondary analysis of a retrospective cohort study of patients seeking first‐trimester abortion care in the United States. We surveyed participants about vaginal bleeding in their current pregnancy and their knowledge of Rh sensitization. We computed the association between seeking care for pregnancy bleeding, and clinical, demographic, and neighborhood characteristics as measured by the Area Deprivation Index (ADI).

**Results**: Of 506 diverse participants, 121 (24%) reported vaginal bleeding during the index pregnancy. Of those experiencing bleeding, 36 (30%) sought care. Age, race and Rh status were not significantly associated with participant behavior. However, a history of prior miscarriage or ectopic pregnancy was significantly associated with increased odds of seeking care for bleeding in this pregnancy (aOR 3.10 95% CI 1.22‐7.85 *p* = 0.017). Patients were more likely to seek care if they lived in a more disadvantaged neighborhood (aOR 1.02 95% CI 1.01‐1.04 *p* = 0.004).

**Conclusion**: Patient blood type may not be a motivation for care‐seeking for a first‐trimester vaginal bleeding. However, personal risk factors for adverse pregnancy events are associated with seeking care. Furthermore, neighborhood socioeconomic disadvantage may lead to higher healthcare utilization in this population. While the behavior of this cohort was likely influenced by numerous circumstances specific to their pregnancy desires, these results suggest a need for further study in order to understand the variables that affect healthcare seeking patterns among pregnant individuals.

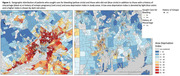

## 365 | The impact of ‘In‐Clinic’ phlebotomy in prenatal care

Efe E. Cudjoe^1^, Matthew K. Janssen^2^, Elizabeth Clement^1^, Eileen Wang^1^

^1^Hospital of the University of Pennsylvania, Philadelphia, Pennsylvania; ^2^University of Pennsylvania, Pittsburgh, Pennsylvania

16:45–18:00

**Objective**: We aimed to evaluate the impact of offering in‐clinic phlebotomy services on improving the timely completion of laboratory tests for pregnant patients, addressing barriers such as the physical distance between laboratories and prenatal clinics.

**Study Design**: We conducted a retrospective cohort study at a single obstetric clinic, comparing pre‐intervention (1/ 2018‐7/2018) and post‐intervention (1/2023‐7/2023) periods, where ‘in‐clinic’ phlebotomy services were introduced in January 2023. In the pre‐intervention phase, providers ordered laboratory tests, and patients scheduled appointments at external locations, while the ‘in‐clinic’ model allowed for lab draws during the prenatal visit. Hemoglobin A1C was chosen as a proxy for all prenatal labs since universal A1C screening is conducted at initial prenatal visits with significant pregnancy implications. The primary outcome measure was the time from lab order to completed result. Pre‐ and post‐implementation outcomes were compared using appropriate statistical tests. Additionally, a subgroup analysis was performed to evaluate the impact at different gestational ages.

**Results**: A total of 2266 pregnant patients were included in the study, with 998 patients in the pre‐implementation group and 1187 in the post‐implementation group. The mean completion time was reduced from 17.4 days ± 31 pre‐intervention to 5.0 days ± 12.1, *p* < 0.001, post‐intervention, with 76% of lab draws occurring within 3 days. The overall completion rate pre‐ and post‐implementation were 89% and 98%, respectively (Table 1). Subgroup analysis showed consistent reductions in the days to completion across various gestational ages, with the most substantial improvement observed in patients between 13‐ 18 weeks gestation.

**Conclusion**: Implementing ‘in‐clinic’ phlebotomy services shortened the time to complete prenatal labs at initial obstetric visits, enhancing efficiency and enabling earlier intervention for abnormal results. Future research should investigate other barriers to lab completion, the cost‐effectiveness of in‐clinic phlebotomy, and its impact on clinical outcomes.

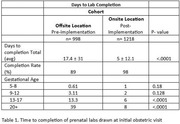

## 367 | “It's a Priority”: A qualitative analysis of the implementation of a maternal equity safety bundle

Anna K. Daoud^1^, Elysia Larson^2^, Tonia Janae Rhone^3^, Heather Olden^4^, Kali Vitek^5^, Hafsatou Diop^6^, Ndidiamaka Amutah‐Onukagha^7^, Audra R. Meadows^8^

^1^NewYork‐Presbyterian/Weill Cornell Medical Center, New York, NY; ^2^Department of Obstetrics and Gynecology, Beth Israel Deaconess Medical Center, Boston, Massachusetts; ^3^UT Southwestern Medical Center, Dallas, Texas; ^4^Harvard T.H. Chan School of Public Health, Boston, Massachusetts; ^5^The Perinatal Neonatal Quality Improvement Network of Massachusetts (PNQIN), Boston, Massachusetts; ^6^Massachusetts Department of Public Health, Boston, Massachusetts; ^7^Tufts University School of Medicine, Boston, Massachusetts; ^8^University of San Diego Medical Center, La Jolla, California

16:45–18:00

**Objective**: Black‐White inequities in severe maternal morbidity in the United States are extreme and growing. Maternal safety bundles (MSBs) have been shown to reduce these inequities. We designed and implemented a standalone equity‐focused MSB across the five hospitals in MA. This study describes the experiences of clinicians implementing the Equity MSB and identifies themes in the process.

**Study Design**: Focus group discussions (FGDs) were conducted in Fall 2022 and Fall 2023, before and after Equity MSB implementation among obstetric nurses, residents, and attending physicians. FGDs were facilitated using a semi‐structured guide developed using the Consolidated Framework for Implementation Research (CFIR). A codebook was developed using CFIR for deductive coding and inductive codes were added as appropriate. Transcripts were independently coded by two analysts using NVivo 14. Cohen's kappa coefficients were calculated to assess inter‐rater reliability. Themes were generated through an iterative process and compared across study time points.

**Results**: Fifteen clinicians participated at each time point with similar distributions across race, ethnicity, gender, and profession. Seven themes emerged: 1) the importance of leadership support and engagement, 2) a culture of equity as a facilitator for implementation, 3) the need for improved processes for self‐reported race, ethnicity, and language data collection, stratification, and dissemination, 4) staff, time, and funding as necessary resources, 5) an early focus on staff education, 6) existing siloes between physicians and nurses and exclusion of trainees as barriers to implementation, and 7) the difference between an Equity‐MSB and other MSBs.

**Conclusion**: Participating hospitals successfully implemented governance through departmental equity teams and processes for stratifying quality outcomes by demographics. Challenges identified included resistance to change, limited resources, and clinician siloes. When compared to previously implemented MSBs, participants found that leadership made this work a priority.

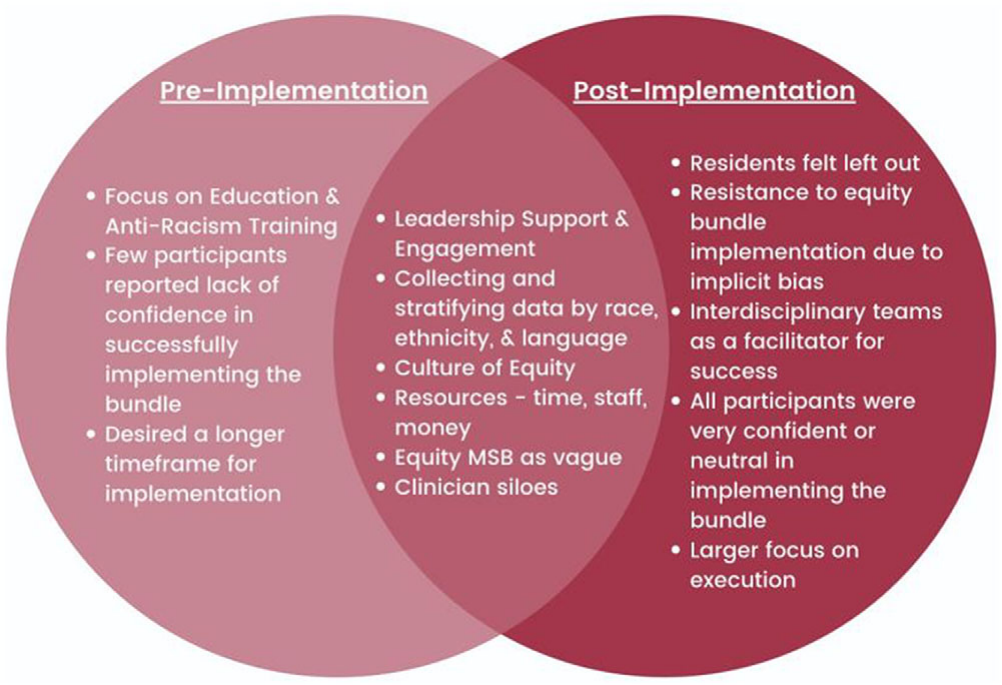

## 368 | Trends in implementing structure and process measures of a Maternal Equity Safety Bundle

Anna K. Daoud^1^, Kali Vitek^2^, Claire Conklin^3^, Hafsatou Diop^4^, Elysia Larson^5^, Ndidiamaka Amutah‐Onukagha^6^, Audra R. Meadows^7^

^1^NewYork‐Presbyterian/Weill Cornell Medical Center, New York, NY; ^2^The Perinatal Neonatal Quality Improvement Network of Massachusetts (PNQIN), Boston, Massachusetts; ^3^Kaiser Permanente San Francisco Medical Center, San Francisco, California; ^4^Massachusetts Department of Public Health, Boston, Massachusetts; ^5^Department of Obstetrics and Gynecology, Beth Israel Deaconess Medical Center, Boston, Massachusetts; ^6^Tufts University School of Medicine, Boston, Massachusetts; ^7^University of San Diego Medical Center, La Jolla, California

16:45–18:00

**Objective**: Black‐White inequities in severe maternal morbidity in the United States are extreme and growing. Maternal safety bundles (MSBs) have been shown to reduce these inequities. We designed and implemented a standalone equity‐focused MSB across the five hospitals in MA. This study identifies patterns among structure and process measures implemented as part of an Equity MSB aimed at reducing severe maternal morbidity, improving patient experience, and eliminating racial inequities.

**Study Design**: The Equity MSB was implemented between September 2022 and September 2023. Sites submitted structure and process measures monthly via REDCap. Data across the five sites at the end of the study were aggregated and changes from pre‐ to post‐implementation were assessed using descriptive statistics and paired t‐tests on Stata/SE 18.0 and visualized on Tableau.

**Results**: Three structure measures were fully implemented during the study period: (1) the establishment of a formal equity team, (2) the ability to collect self‐reported race and ethnicity data, and (3) the ability to stratify outcome and process data by race and ethnicity. The paired t‐test for structure measures revealed a significant difference between structures in place pre‐ and post‐implementation (t = ‐3.3, df = 15, *p* = 0.0048). Process measures demonstrated differences in care delivery for obstetric hemorrhage and severe hypertension in pregnancy between non‐Hispanic White (NHW) and non‐Hispanic Black (NHB) patients. Notably, timely treatment of hypertension increased by 47% among NHW and by 134% among NHB patients.

**Conclusion**: Implementation of an equity MSB led to the establishment of infrastructure for maternal equity and improvement in data stratification. Practicing evidence‐based processes of care also improved, including timely treatment of hypertension for both NHB and NHW patients. This study identified future target areas for quality improvement projects, such as community engagement and collecting patient experience data.

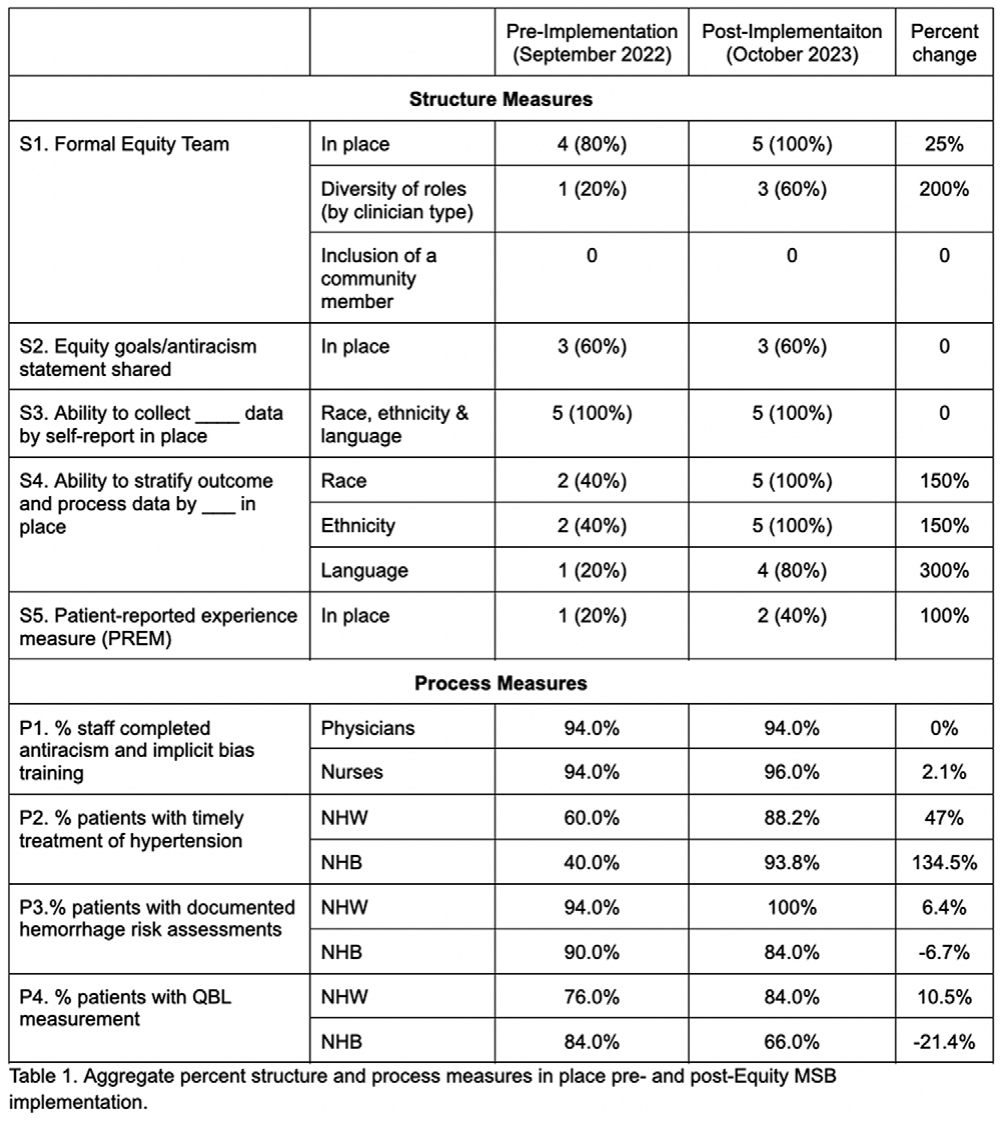

## 369 | Establishing evidence‐based dose recommendations for amoxicillin (with or without clavulanic acid) in pregnancy

Anna Caroline Dibbets^1^, Inca Slaats^2^, Angela Colbers^1^, Hubertina CJ Scheepers^3^, Saskia de Wildt^1^

^1^Radboud University Medical Center, Radboud University Medical Center, Gelderland; ^2^Radboud Univeristy Medical Center, Radboud University Medical Center, Gelderland; ^3^Maastricht University Medical Center, Maastricht University Medical Center, Limburg

16:45–18:00

**Objective**: Amoxicillin and its combination with clavulanic acid (Augmentin) are commonly used antibiotics in pregnancy. Dutch dosing guidelines often advise higher doses for non‐pregnant individuals than for pregnant women. However, physiological changes in pregnancy might result in antenatal doses falling below therapeutic levels, which may necessitate dose adjustments. This evidence review aimed to establish evidence‐based antenatal dose recommendations for amoxicillin and Augmentin for implementation in clinical practice.

**Study Design**: Data on pharmacodynamics, safety, and efficacy of amoxicillin and Augmentin was collected, along with evidence from pharmacokinetic models, to develop antenatal dose recommendations using our previously developed Framework for Dose Selection in Pregnancy (FDSP). The FDSP and the proposed doses were reviewed by a multidisciplinary working committee of project MADAM, including healthcare practitioners, other experts, and patient representatives.

**Results**: Published pharmacokinetic simulations show slightly lower concentrations of amoxicillin in the second and third trimester of pregnancy compared to non‐pregnant individuals, due to an increase in clearance. Aligning pregnancy doses with non‐pregnant doses for treating mild‐to‐moderate infections may mitigate the risk of antimicrobial resistance, underdosing and associated complications. Recommending the highest dose within a dose range during pregnancy could also be advisable. For prophylaxis during preterm prelabour rupture of the membranes, increasing the daily dose from three oral doses of 500 mg to three oral doses of 750 mg may be warranted. Pharmacokinetic simulations suggest that the current dosing regimen (2000 mg iv bolus followed by 1000 mg q6h) for prophylaxis during childbirth for prevention of early‐onset neonatal sepsis is adequate. The dose recommendations were endorsed by the working committee and will be integrated into clinical practice.

**Conclusion**: This evidence review underscores the current risk of underdosing of amoxicillin and Augmentin for most indications and the need to adjust doses accordingly.

## 370 | Impact of a strict vaginal delivery protocol on second twin cesarean rate in twin pregnancies

Ari Weiss^1^, Misgav Rottenstreich^2^, Tzuria Peled^1^, Hen Y. Y. Sela^3^, Sorina Grisaru Granovsky^4^, Zvi Ehrlich^5^

^1^Shaare Zedek Medical Center, Shaare Zedek Medical Center, Yerushalayim; ^2^McMaster University, Hamilton, Ontario; ^3^Shaare Zedek, Jerusalem, Yerushalayim; ^4^Hebrew University, Jerusalem, Yerushalayim; ^5^Shaare Zedek Medical Center, Jerusalem, Yerushalayim

16:45–18:00

**Objective**: Cesarean delivery of the second twin following vaginal delivery of the first twin is associated with adverse outcomes. This study aims to assess the influence of a strict departmental protocol on the incidence of second twin cesarean delivery.

**Study Design**: A retrospective study of all twin deliveries at a tertiary medical center between 2005 and 2023. Patients with planned or urgent cesarean delivery of the first twin were excluded. The departmental protocol offers vaginal delivery to diamniotic twins pregnancies (regardless of chorionicity) at 32 0/7 weeks of gestation or later with a vertex‐presenting first twin, irrespective of the second twin's presentation. Vaginal delivery is discouraged if the estimated fetal weight of the second twin exceeds the first by 20% or more. Continuous fetal heart rate monitoring and routine epidural analgesia are employed. A senior obstetrician experienced in total breech extraction of the second twin, along with a resident, two midwives, and a pediatrician, are present during delivery. Second twin presentation is assessed through vaginal examination and transabdominal sonography. Immediate total breech extraction is performed for breech presentation or transverse lie, while efforts to shorten the delivery interval are made for vertex presentation. Oxytocin is administered, vaginal delivery is attempted, and membranes are ruptured when conditions are suitable. Both neonates are assessed by the attending pediatrician.

**Results**: Over the study period, 5,037 twin pairs were born. Among them, 1,853 (36.4%) had planned cesarean delivery, and 3,202 (63.6%) underwent trial of labor. A total of 786 (15.6%) patients had cesarean delivery for both twins. There were 2,416 (47.9%) successful vaginal deliveries of twin A, with only 32 cases (1.3%) of cesarean deliveries for the second twin despite a successful vaginal delivery of twin A.

**Conclusion**: Implementing a strict protocol for selecting appropriate candidates during trial of labor for twin pregnancies resulted in a low rate of second twin cesarean delivery compared to the rates reported in the literature.

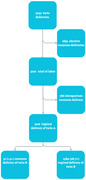

## 371 | Impact of systematic simulation‐based training on vacuum delivery competencies among OBGYN residents

Hagit Eisenberg^1^, Ilia Kleiner^2^, Eran Weiner^1^, Liliya Tamayev^3^, Masha Ben‐Zvi^2^, Daniel Tairy^3^, Laura Guzy^2^, Yael Ganor Paz^3^

^1^Wolfson Medical Center, Wolfson Medical Center, Tel Aviv; ^2^Edith Wolfson Medical Center, Edith Wolfson Medical Center, Tel Aviv; ^3^Edith Wolfson Medical center, Holon, HaMerkaz

16:45–18:00

**Objective**: In May 2023 we introduced routine weekly multidisciplinary simulation‐based training in obstetrics as part of our OBGYN residency program. This study aimed to investigate the effect of this weekly simulation‐based training on residents' knowledge and competency in performing complex vacuum deliveries.

**Study Design**: A quantitative interventional study was conducted over a one year period between May 2023 and April 2024. A 2‐hour simulation in the delivery unit was held weekly, using an obstetric trainer for practicing the manual skills, and a trained actor as the patient, for the purpose of communication skills. Multiple complex scenarios were planned and tailored to each simulation by our medical‐education team and the research team. Participants' knowledge and technical skills were evaluated pre‐ and post‐intervention using a knowledge‐based questionnaire and the validated Objective Structured Assessment of Technical Skills (OSATS) scale.

**Results**: During the 1 year study period, our 26 residents were trained in 42 simulation sessions. The knowledge‐based questionnaire scores showed a significant improvement, with the mean score rising from 58.80 ± 15.92 points pre‐simulation vs. 69.85 ± 15.29 points post‐simulation (*p* = 0.008). Similarly, a statistically significant difference was observed in the OSATS scores, with the mean score increasing from 59.73 ± 14.79 points before the simulation to 66.88 ± 11.06 points after the simulations (*p* = 0.013).

**Conclusion**: Systematic weekly simulation‐based training within the clinical setting of the labor and delivery unit–significantly improved physicians' knowledge and technical skills in performing vacuum‐assisted deliveries. The increased proficiency, as evidenced by the improved OSATS scores and knowledge questionnaire results, underscores the value of incorporating simulation training into obstetric residency programs to potentially improve obstetric outcomes. Further research is planned to explore the direct impact of this training on maternal and neonatal outcomes.

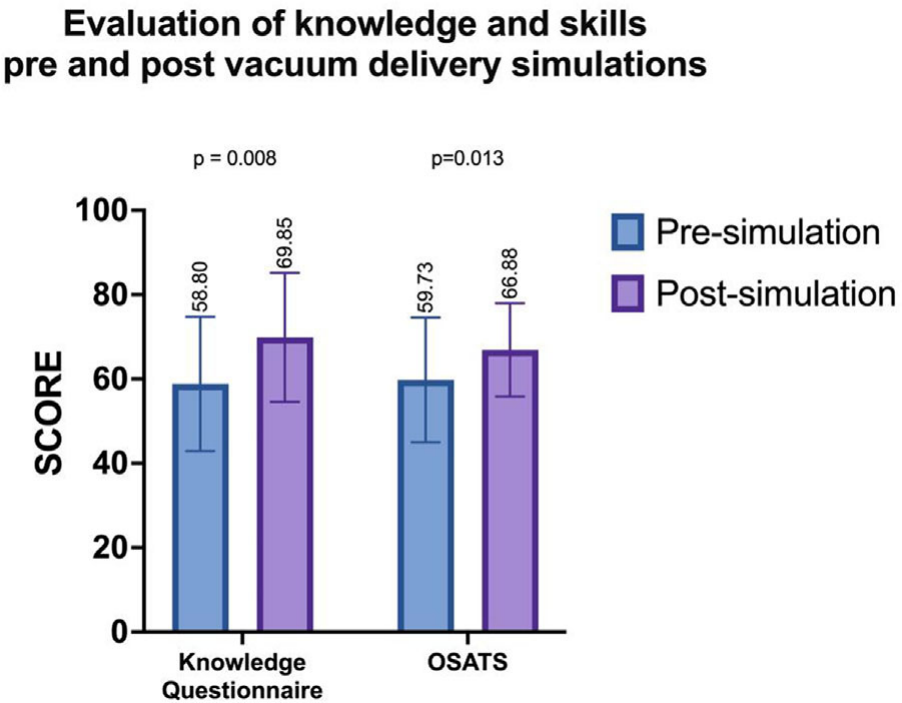

## 372 | Association between SARS‐CoV‐2 in pregnancy and offspring neurodevelopmental outcomes: A retrospective cohort study

Malia S.Q. Murphy^1^, Karen Wong^2^, Robin Ducharme^3^, Anna Clarke^4^, Jason Brophy^5^, Steven Hawken^2^, Darine El‐Chaâr^6^

^1^Ottawa Hospital Research Institute, Ottawa, ON; ^2^Ottawa Hospital Research Institute, Ottawa, Ontario; ^3^Ottawa Hospital Research Institute, Ottawa Hospital Research Institute, ON; ^4^Institute for Clinical Evaluative Sciences, Ottawa, Ontario; ^5^Children's Hospital of Eastern Ontario, Ottawa, Ontario; ^6^The Ottawa Hospital, Ottawa, ON

16:45–18:00

**Objective**: The objective of this study was to examine offspring neurodevelopmental risks following SARS‐CoV‐2 infection in pregnancy.

**Study Design**: This was a population‐based retrospective cohort study of pregnant individuals who delivered between March 1, 2020 and March 31, 2024 in Ontario, Canada, using health administrative data. The exposure group included pregnancies with confirmed SARS‐CoV‐2 infection. The comparator group included pregnancies without confirmed SARS‐CoV‐2 infection, matched (1:10) for age, geography, and last menstrual period date. Multivariable generalized linear models were used to assess the association between exposure and neurosensory deficits and neurodevelopmental disorder.

**Results**: There were 222,448 live births during the study period. The exposed group included 5,291 pregnancies and the comparator group included 40,667 pregnancies. Demographic and obstetric characteristics were similar between groups. There was no statistical difference between groups for neurosensory deficits (HR (95% CI): 0.85 (0.67‐1.07) or neurodevelopmental disorders (HR 1.02 (0.92‐1.13). When the comparator group was limited to pregnancies with a negative SARS‐CoV‐2 test, there was no difference in risk of neurodevelopmental disorder (HR 1.02 (0.92‐1.15), but there appears to be a protective effect for neurosensory deficits (HR 0.77 (0.60‐0.98).

**Conclusion**: A positive SARS‐CoV‐2 test in pregnancy was not associated with an increased risk of neurosensory deficits or neurodevelopmental disorder in the offspring's follow up period of up to two years’ old, which may be reassuring to parents and healthcare providers. We emphasize caution in interpretation of these results given the likelihood of residual confounding.

## 373 | Placental histopathology and SARS‐CoV‐2 infection in pregnancy: A prospective cohort study

Darine El‐Chaâr^1^, Sapir Fellus^2^, Malia S.Q. Murphy^3^, Serine Ramlawi^2^, Sonia Dancey^2^, Ruth Rennicks White^2^, Alysha Dingwall‐Harvey^4^, Shuhiba Mohammad^2^, Mark Walker^2^, Marc‐Andre Langlois^5^, Shannon Bainbridge‐Whiteside^5^, Barbra de Vrijer^6^, Dina El‐Demellawy^7^

^1^The Ottawa Hospital, Ottawa, ON; ^2^Ottawa Hospital Research Institute, Ottawa, Ontario; ^3^Ottawa Hospital Research Institute, Ottawa, ON; ^4^Better Outcomes Registry & Network (BORN) Ontario, Ottawa, Ontario; ^5^University of Ottawa, Ottawa, Ontario; ^6^London Health Sciences Centre, London, Ontario; ^7^Children's Hospital of Eastern Ontario, Ottawa, Ontario

16:45–18:00

**Objective**: Data on the effects of SARS‐CoV‐2 on placental pathology are inconsistent. Our objective is to investigate the impact of SARS‐CoV‐2 infection in pregnancy on placental histopathology compared to pre‐pandemic controls.

**Study Design**: This was a sub‐analysis of a large multi‐center prospective cohort study of pregnant individuals with laboratory‐confirmed SARS‐CoV‐2 infection in Ontario who delivered between Mar‐2020 and Jul‐2021. Infected cases were matched to uninfected pre‐pandemic controls based on the presence of maternal comorbidities, gestational age at delivery and maternal age. Placentas were evaluated for differences in frequency and type of histologic lesions using a placental phenotypic classification tool.

**Results**: Our sample of 112 COVID‐19 cases included those with 1^st^, 2^nd^, and 3^rd^ trimester infections (13.4%, 38.4% and 45.5%, respectively). The majority had experienced mild (81.3%) and symptomatic (88.4%) disease at the time of illness. COVID‐19 cases exhibited higher median placental weights than pre‐pandemic controls (*p* = 0.006), but lower fetoplacental weight ratios (*p* < 0.001). COVID‐19 cases exhibited a higher frequency of acute inflammation compared to controls (31.3% versus 7.1%, *p* < 0.001), and higher rates of maternal vascular malperfusion (34.8% versus 21.4%, *p* = 0.037) and fetal vascular malperfusion (11.6% versus 1.8%, *p* = 0.006). Among cases, third trimester infections had a lower frequency of lesions compared to those infected with SARS‐CoV‐2 earlier in pregnancy (1^st^ trimester, 73.3% versus 2^nd^ trimester, 58.1% versus 3^rd^ trimester, 7.8%).

**Conclusion**: These findings suggest that SARS‐CoV‐2 infection in pregnancy effects placental development, resulting in notable changes in growth and pathology. Given the potential long‐term health effects of placental deficiencies on affected offspring, further investigation is warranted.

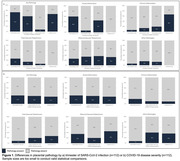

## 374 | Fetal ventricular disproportion and postnatal outcome in congenital diaphragmatic hernia

Alessia Sala^1^, Chiara Vassallo^2^, Flaminia Pugnaloni^3^, Milena Viggiano^4^, Domenico Umberto De Rose^3^, Irma Capolupo^3^, Alessandra Di Pede^3^, Laura Valfrè^3^, Andrea Conforti^3^, Andrea Dotta^3^, Lorenza Driul^5^, Pietro Bagolan^3^, Leonardo Caforio^4^, Isabella Fabietti^4^

^1^University of Udine, Udine, Friuli‐Venezia Giulia; ^2^Ospedale Pediatrico Bambino Gesù, Ospedale Pediatrico Bambino Gesù, Lazio; ^3^Ospedale Pediatrico Bambino Gesù, Rome, Lazio; ^4^Ospedale Pediatrico Bambino Gesù, Roma, Lazio; ^5^Ospedale Santa Maria della Misericordia di Udine, Udine, Friuli‐Venezia Giulia

16:45–18:00

**Objective**: To relate fetal ventricular disproportion in congenital diaphragmatic hernia (CDH) to postnatal outcomes.

**Study Design**: Retrospective analysis of right ventricle (RV)‐to‐left ventricle (LV) diameter (RV_D_/LV_D_), RV and LV end‐diastolic areas, aortic inlet valve and pulmonary artery inlet valve diameters in CDH fetuses and t‐test comparison with survival and postnatal pulmonary hypertension (PH).

**Results**: During the study period, a total of 41 patients were enrolled. Among them, 23 patients had a complete evaluation of LV and RV end‐diastolic diameters for the second scan (28‐36 gestational weeks). Data about RV and LV end‐diastolic areas were available for a total of 32 patients whereas measurements of the aortic inlet valve and pulmonary artery inlet valve diameters were obtainable for 31 patients.

Student's t‐test revealed that non‐survivors CDH newborn exhibited a significantly higher RV‐to‐LV diameter, consistent with postnatal observations (p 0.019). No statistically significant differences were observed concerning the end‐diastolic area ratio nor the diameters of the aortic inlet valve or pulmonary valve between survivors and non‐survivors. Furthermore, no significant association was detected between the severity of pulmonary hypertension and the RV_D_/LV_D_ (p 0.525).

**Conclusion**: In our series of CDH, fetal ventricular disproportion with an higher RV_D_/LV_D, _is a potential predictor of neonatal mortality, but does not relate to PH severity.

## 375 | The effect of Prolonged 2nd stage on Breastfeeding rates

Naama Farago^1^, Gal Bachar^2^, Merav Shumsky Fan^3^, Naphtali Justman^2^, Yaniv Zipori^2^, Nizar khatib^4^, Zeev Weiner^5^, Dana Vitner^2^

^1^Rambam Health Care Center, Rambam Health Care Center, HaZafon; ^2^Rambam Medical Center, Haifa, Hefa; ^3^Technion–Israel Institute of Technology, Haifa, Hefa; ^4^Rambam Medical Health Campus, Rambam Medical Health Campus, Hefa; ^5^Rambam Medical Health Center, Haifa, Hefa

16:45–18:00

**Objective**: Breastfeeding is considered the ultimate infant feeding method. Several obstetrical factors affecting breastfeeding have already been investigated, including mode of delivery, epidural analgesia, and oxytocin administration.

In this study, we aimed to assess the effect of 2^nd^ stage length on breastfeeding rates in the early postpartum period.

**Study Design**: A retrospective cohort study of all singleton vaginal deliveries at term between October 2009 and April 2022 at a single tertiary center. Women taking lactation suppression medications were excluded from the study. The cohort was divided by the length of the 2^nd^ stage: < 2 hours (group 1), 2‐3 hours (group 2), and > 3 hours (group 3). The primary outcome was exclusive breastfeeding rate.

**Results**: The study cohort included 10,678 women; 7,282 in group 1, 1752 in group 2, and 1,644 in group 3. Women with a shorter 2^nd^ stage duration ( < 2 hours) were more likely to be multiparous (1.58 ± 0.49 vs. 1.20 ± 0.4 vs. 1.1 ± 0.3, *p* < .0001), with a lower rate of epidural analgesia (71.97% vs. 95.49% vs. 95.44%, *p* < .0001), with a lower rate of oxytocin use (34.25% vs. 66.67% vs. 86.44%, *p* < .001) and shorter total labor duration (8.88 ± 21 hours vs. 14.64 ± 24 hours vs. 15.84 ± 29.4 hours, *p* < .0001) compared to the other two groups.

When 2^nd^ stage was longer, women were less likely to breastfeed exclusively 12 hours following delivery (47.65% vs. 48.46% vs. 51.83%, *p* < .005). Exclusive breastfeeding rate decreased further during 12‐48 hours until discharge (24.38% vs. 20.20% vs. 29.42%, *p* < .001). The effect was similar among nulliparous and multiparous women. The results remained statistically significant after adjusting for confounders using a multivariate regression analysis.

**Conclusion**: Breastfeeding rates tend to be lower among women who experience a longer 2nd stage of labor, up to 48 hours following childbirth. This suggests that they may take longer to recover from delivery. Identifying the population at risk for breastfeeding difficulties can help in designing special intervention programs aimed at providing support to those mothers who need it.

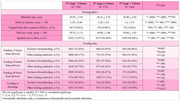

## 376 | The interaction between maternal age and fetal macrosomia on adverse pregnancy and neonatal outcomes

Naama Farago^1^, Gal Bachar^2^, Liri lola sharoni^3^, Dana Vitner^2^, Zeev Weiner^4^, Yaniv Zipori^2^, Nizar khatib^5^

^1^Rambam Health Care Center, Rambam Health Care Center, HaZafon; ^2^Rambam Medical Center, Haifa, Hefa; ^3^Technion, Haifa, Hefa; ^4^Rambam Medical Health Center, Haifa, Hefa; ^5^Rambam Medical Health Campus, Rambam Medical Health Campus, Hefa

16:45–18:00

**Objective**: Over the last few decades, an ascending trend in advanced maternal age, widely accepted as 35 years or more, has been observed in high‐income countries. There are several risk factors for adverse pregnancy outcomes with advanced age; fetal macrosomia is among the most significant. This study seeks to analyze the relationship between advanced maternal age and fetal macrosomia, and the resulting effects on pregnancy and neonatal outcomes.

**Study Design**: This retrospective cohort study, conducted at a single tertiary care center between 2009 and 2022, enrolled women with advanced maternal age and singleton pregnancies diagnosed with fetal macrosomia. We compared pregnancy outcomes in women aged up to 34, 35‐39, and ≥40 years. The primary outcome is the mode of delivery, and the secondary outcome is a composite of maternal and neonatal complications.

**Results**: A total of 2,326 women who gave birth to macrosomic neonates were included in the study. Women were divided into three groups, 1‐ 1756 aged 18 to 34 years, 2–469 were between 35‐39 years; and 3‐ 101 in the advanced maternal age group, women older than 40. Women in the advanced maternal age were more parous (2.15 ± 1.17 vs. 3.40 ± 1.73 and 4.03 ± 1.93, *p* < 0.001), had higher rates of gestational diabetes (111 (6.3%) vs 40 (8.5%) and 18 (17.8%), *p* < 0.001). The primary outcome, CS rates, was comparable between the groups (306 (17.4%) vs. 76 (16.2%) and 13 (18.8%), *p* = 0.24). Maternal and neonatal outcomes and complications were similar between the groups.

**Conclusion**: The interaction between fetal macrosomia and advanced maternal age did not result in increased adverse outcomes for either mother or neonate and did not affect the mode of delivery.

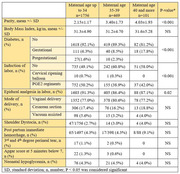

## 377 | Prophylactic Aspirin administration for twin pregnancies

Naphtali Justman^1^, Roee David. Goldfreind, Sr.^1^, Hiba Abu‐Rass^2^, Yoav Siegler^3^, Gilad Shahak^1^, Gal Bachar^1^, Amir Wolfovitz^1^, Nimrod Loberman^1^, Roee Iluz^1^, Yaniv Zipori^1^, Nizar khatib^4^, Zeev Weiner^5^, Dana Vitner^1^

^1^Rambam Medical Center, Haifa, Hefa; ^2^rambam medica center, Haifa, Hefa; ^3^Rambam Health Care Center, Rambam Health Care Center, HaZafon; ^4^Rambam Medical Health Campus, Rambam Medical Health Campus, Hefa; ^5^Rambam Medical Health Center, Haifa, Hefa

16:45–18:00

**Objective**: The aim was to investigate whether prophylactic aspirin administration for twin pregnancies reduced the incidence of hypertensive disorders of pregnancy and associated maternal and neonatal adverse outcome.

**Study Design**: A retrospective cohort study was conducted on twin pregnancies delivered at a university‐affiliated medical center between January 1^st^, 2010 and December 31^st^, 2019. The study compared outcomes between twin pregnancies receiving 100 mg/day aspirin treatment during pregnancy (study group) and those not receiving treatment (control group). Additionally, a comparative analysis of outcomes was performed between the two groups.

**Results**: The study included two groups: the aspirin group (*n* = 229) and the Control group (*n* = 1525). The rate of advanced maternal age, and morbidly obese gravidas was higher in the aspirin group (36.7% vs. 26.2%, *p* < 0.001; 26.7% vs. 15.9%, *p* = 0.002, respectively). The rate of IVF pregnancy was also higher in the aspirin group (47.2% vs. 39.1%, *p* = 0.02).

Women in the aspirin group were more likely to have gestational diabetes and chronic hypertension (7.9% vs. 3.5%, *p* = 0.002; 3.1% vs. 0.8%, *p* = 0.002, respectively). After matching for nulliparity, advanced maternal age, and morbid obesity, the rate of pre‐eclampsia/hypertension of pregnancy in the aspirin group was significantly lower than the control group (21.4% vs. 58.6%, *p* < 0.001). However, the incidence of severe pre‐eclampsia, intrauterine growth restriction, placental abruption, and postpartum hemorrhage did not significantly differ between groups.

**Conclusion**: Our study suggests a potential protective effect of prophylactic aspirin against pre‐eclampsia/hypertensive disorders of pregnancy in twin pregnancies, especially since the aspirin group was in high risk for pre‐eclampsia and related complications. However, further research is warranted to validate these findings and to isolate the group that will benefit the most.

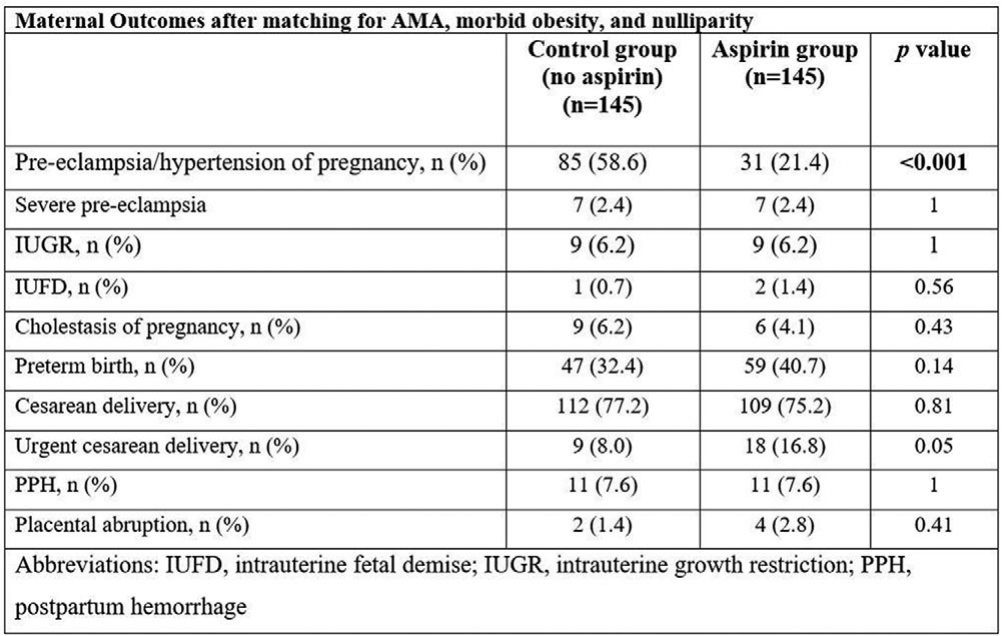

## 378 | Chorioamnionitis following two cervical ripening methods: PGE2 versus Cervical Ripening Balloon

Naphtali Justman^1^, Nimrod Loberman^1^, Shmuel Somer^2^, Roee Iluz^1^, Amir Wolfovitz^1^, Gal Bachar^1^, Yaniv Zipori^1^, Zeev Weiner^3^, Dana Vitner^1^, Nizar khatib^4^, Roni Avrahami^1^, Zvi Millo^1^

^1^Rambam Medical Center, Haifa, Hefa; ^2^technion institute of technology, haifa, Hefa; ^3^Rambam Medical Health Center, Haifa, Hefa; ^4^Rambam Medical Health Campus, Rambam Medical Health Campus, Hefa

16:45–18:00

**Objective**: This study aimed to compare pregnancy outcomes, with a particular focus on chorioamnionitis, between two cervical ripening methods: prostaglandin E2 (PGE2) and cervical ripening balloon (CRB).

**Study Design**: A retrospective cohort study was conducted at a tertiary medical center from 2010 to 2020. Pregnant patients who underwent cervical ripening using either PGE2 or CRB were included in the study. The primary outcome was the rate of chorioamnionitis. The secondary outcomes included cesarean delivery (CD), postpartum hemorrhage (PPH), neonatal acidemia, 5‐minute Apgar scores ≤7, and NICU admission rates. We used the chi‐square test for categorical variables and t‐tests or Mann‐Whitney tests for continuous variables. A significance level of *p* > 0.05 was considered significant for all analyses.

**Results**: A total of 2089 patients were included in the analysis, with 1579 in the PGE2 group and 510 in the CRB group. The number pf women with obesity was lower in the CRB group compared with the PGE2 group (46.8% vs. 54.5%, *p* = 0.01). More women were induced due to fetal indication in the CRB group (62% vs. 43.4%, *p* < 0.001), and the active phase was longer by 30 minutes in the CRB group (3.17 ± 3.51 vs. 2.47 ± 2.35, *p* < 0.001).

The rate of chorioamnionitis was significantly higher in the CRB group (7.3% vs. 3.9%, *p* = 0.002). No significant difference was observed in CD rate and PPH rate between the two groups (*p* = 0.42; *p* = 0.07, respectively). Neonatal outcomes, including acidemia (*p* = 0.32), and 5‐minute Apgar scores (*p* = 0.90), were similar between the groups. However, NICU admission rates were higher in the CRB group (7.3% vs. 4.3%, *p* = 0.002).

In a multivariate analysis, after controlling for second stage of ≥ 3 hours (RR = 1.7, *p* = 0.049), and gestational age at delivery ≥ 39 weeks (RR = 2.8, *p* = 0.047), and BMI (*p* = 0.33), CRB was associated with increased risk for chorioamnionitis (RR = 2.7, *p* < 0.001).

**Conclusion**: The use of CRB is associated with a higher rate of chorioamnionitis and NICU admission compared to PGE2. These findings underscore the importance of considering the risk of chorioamnionitis associated with CRB use.

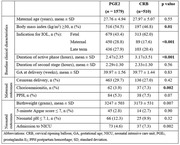

## 379 | Pregnancy outcomes with decreased fetal movements at term: Impact of a novel protocol

Naphtali Justman^1^, Roee David. Goldfreind, Sr.^1^, Roee Iluz^1^, Chen Ben David^2^, Gal Bachar^1^, Nimrod Loberman^1^, Yaniv Zipori^1^, Yoav Siegler^3^, Amir Wolfovitz^1^, Zeev Weiner^4^, Dana Vitner^1^, Nizar khatib^5^, Ron Beloosesky^4^, Yuval Ginsberg^1^

^1^Rambam Medical Center, Haifa, Hefa; ^2^Rambam health care campus, Haifa, Hefa; ^3^Rambam Health Care Center, Rambam Health Care Center, HaZafon; ^4^Rambam Medical Health Center, Haifa, Hefa; ^5^Rambam Medical Health Campus, Rambam Medical Health Campus, Hefa

16:45–18:00

**Objective**: We aimed to compare pregnancy outcomes in singleton pregnancies with subjective decreased fetal movements at term (≥37 weeks of gestation) before and after the implementation of a novel protocol recommending induction of labor after 39 weeks compared to induction of labor up to 42 weeks.

**Study Design**: A retrospective cohort study was conducted on pregnant women presenting with decreased fetal movements at term (≥37 weeks of gestation) at a single tertiary care center during two distinct time periods before (January 2012 until July 2015) and after (July 2015 until July 2021) the implementation of a novel protocol (June 2015). In the 1st period induction was performed up to 42 weeks and in the 2nd period of time at 39 weeks. Maternal demographic information, obstetric history, and delivery characteristics were recorded and compared between the two cohorts. The primary outcome assessed was the mode of delivery. The secondary outcomes included neonatal acidemia, 5‐minute Apgar scores, as well as admission and length of stay in the Neonatal Intensive Care Unit (NICU).

**Results**: The study included 644 patients, 218 gravidas before and 426 after the implementation of the novel protocol. Maternal demographics and obstetric characteristics were similar comparing between the two groups. The rate of cesarean delivery and late‐term delivery were higher in period I (33.6% vs. 24.2%, *p* = 0.03; 20.6% vs. 13.1%, *p* = 0.01, respectively). All other maternal outcomes were similar between the groups. Neonatal outcomes, including neonatal acidemia (*p* = 0.26), 5‐minute Apgar scores (*p* = 0.55), and admission to NICU (*p* = 0.09) were comparable between the groups.

**Conclusion**: Labor induction at 39 weeks in women with decreased fetal movement was associated with a reduced rate of cesarean delivery and late‐term delivery without significant differences in other maternal and neonatal outcomes. These findings suggest that encouraging labor induction at 39 weeks for patients with decreased fetal movements may improve pregnancy outcomes

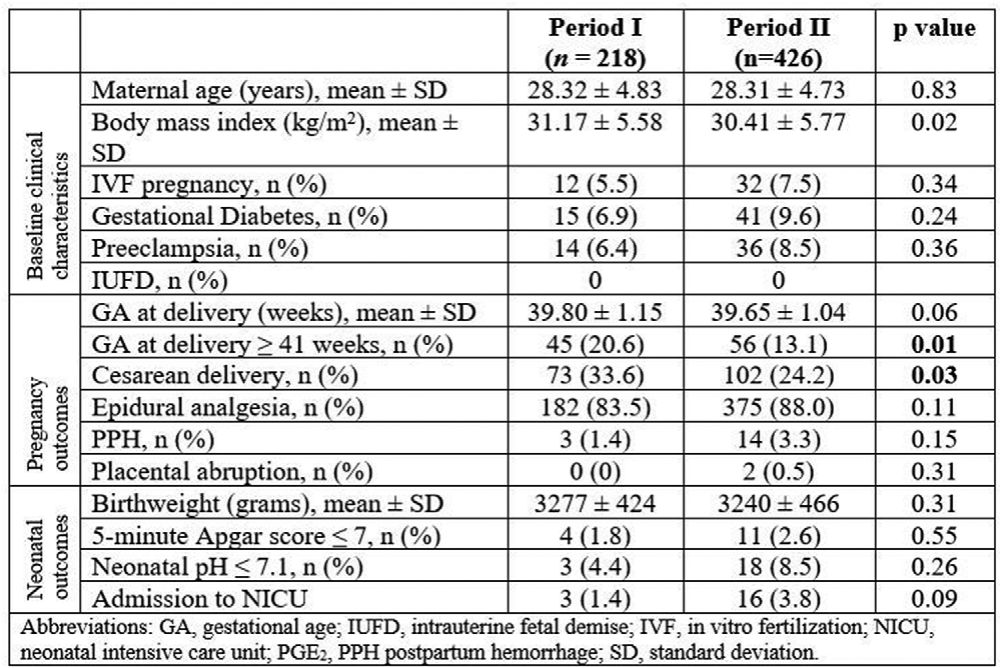

## 381 | Changes in fetal membranes mechanical properties during preterm labor

Alaa A. Arraf^1^, Rachel Bitton^2^, Nizar khatib^3^, Yuval Ginsberg^4^, Joshua Grolman^2^, Ron Beloosesky^5^

^1^Rambam Health Care Campus, Rambam Heath Care Campus, Hefa; ^2^Technion Israel Institute of Technology, Technion Israel Institute of Technology, Hefa; ^3^Rambam Medical Health Campus, Rambam Medical Health Campus, Hefa; ^4^Rambam medical center, Haifa, Hefa; ^5^Rambam Medical Health Center, Haifa, Hefa

16:45–18:00

**Objective**: Preterm birth, occurring in 10 to 12 percent of pregnancies is the leading cause of fetal and neonatal morbidity and mortality. Preterm premature rupture of membranes (PPROM) is the leading cause of preterm birth. However, efforts in predicting and preventing PPROM have been hampered by poor comprehensive understanding of the underlying pathophysiological mechanism. In this study, we use the fetal membranes in preterm labor, term labor and induced term pregnancies in order to understand nanoscale features and biological propertiesmof fetal membranes in preterm and term pregnancies.

**Study Design**: Fetal membranes (chorion and amnion) was dissected at the rupture site immediately after delivery. Three groups of membranes were investigated: Preterm labor membranes (*n* = 5), term labor membranes (*n* = 6), and medically induced term labor membranes (using Pitocin or Prostaglandin E2) (*n* = 12). Following, micro‐dissection was used to separate chorion and amnion layers. Stiffness and visco‐elastic properties of the membranes were measured using Nanoindenter. Collagen quantification assessment was measured using Hydroxyproline assay. Collagen ECM architecture was visualized using SHG (Second Harmonic Generation) two photon Microscope and Immunohistochemistry staining.

**Results**: Nanoindentation results demonstrated higher stiffness and viscoelastic properties in the Amnion layer of preterm samples compared to term (in both induced an spontaneous labors), whereas such significant differences are not observed in the chorion membrane. Collagen quantity assessments indicate significantly higher collagen percentage in preterm Amnion layer comparing to term labors. Analyzing Collagen budndling using the SHG two photon microscope demonstrates substantial collagen bundling in pre‐term births comparing with term labors.

**Conclusion**: Our study demonstrates differences in fetal membranes mechanical properties in preterm vs term labors that correlates with changes in the collagen quantity and architecture. We suggest that altering the stiffness and viscoelastic properties precipitating early membrane ruptures in preterm labor.

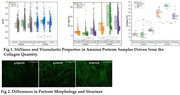

## 384 | Local anesthetic wound infiltration at cesarean delivery effect on pain: Systematic review and meta analysis

Valentina Frusone^1^, Kavisha Khanuja^2^, Jacqueline Sinnott^3^, Valentina Bellussi^4^, Guillaume DUCARME^5^, Amanda Roman^6^, Vincenzo Berghella^7^

^1^Drexel University, Merion Station, PA; ^2^Thomas Jefferson University Hospital, Philadelphia, Pennsylvania; ^3^Sidney Kimmel Medical College, Philadelphia, Pennsylvania; ^4^Ospedale Di Borgo Trento Verona, Verona, Veneto; ^5^Centre Hospitalier Departemental, LA ROCHE SUR YON, Pays de la Loire; ^6^Department of Maternal‐Fetal Medicine, Thomas Jefferson University Hospital, Philadelphia, Pennsylvania; ^7^Sidney Kimmel Medical College of Thomas Jefferson University, Philadelphia, Pennsylvania

16:45–18:00

**Objective**: To evaluate the effect on maternal pain of local anesthetic wound infiltration for intra‐cesarean delivery analgesia in patients who also received intrathecal opioids.

**Study Design**: Scopus, PubMed, and Cochrane Central Register of Controlled Trials were searched from inception of each database to August 2023. Inclusion criteria were randomized controlled trials (RCTs) comparing use of local anesthetic wound infiltration at time of cesarean delivery vs no such infiltration in patients who also received intrathecal opioids. The primary outcome was pain scores at 24 hours with movement. Secondary outcomes included pain scores at 12, 48, 72 hours with movement and at rest, opioid consumption at 48 hours, length of hospitalization, and side‐effects. Results presented as mean difference (MD) or risk ratio (RR) with 95% confidence intervals. Quality assessed by Cochrane Handbook for Systematic Reviews of Interventions for judging risk of bias, and heterogeneity by I‐squared (Higgins I^2^).

**Results**: Seven RCTs included (*n* = 672) comparing local anesthetic wound infiltration to no infiltration during cesarean delivery met inclusion criteria. Local anesthetic (usually with ropivacaine, bupivacaine, or levobupivacaine) wound infiltration was associated with significantly lower pain scores with movement compared to no infiltration (MD −0.95 (‐1.57, −0.33) (I^2^ = 0%) (Table). Secondary outcomes showed decreased but not statistically different pain scores at rest: 24 hours (MD: −2.57 (‐7.79, 2.65), 48 hours (‐3.98 (MD: −8.18, 0.23)), and 72 hours (MD: −4.28 (‐9.56, 1.00)). Analysis of morphine consumption at 48 hours showed a significant decrease in the wound infiltration group (MD −3.09 (‐4.46, −1.72), I^2^ = 46%) (Table). Outcomes were similar for the side effects of nausea, vomiting, pruritus, and sedation.

**Conclusion**: Local anesthetic wound infiltration at the time of cesarean delivery is associated with reduced pain intensity scores with movement. It also reduces overall postoperative morphine consumption with comparable maternal side effects.

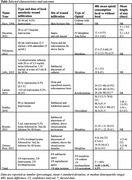

## 385 | Reinforcing cerclage following primary cerclage procedure for preterm birth prevention: A systematic review and meta‐analysis

Virali Patel^1^, Neel Iyer^2^, Vincenzo Berghella^3^

^1^Sidney Kimmel Medical College at Thomas Jefferson University, Philadelphia, Pennsylvania; ^2^Thomas Jefferson University Hospital, Philadelphia, Pennsylvania; ^3^Sidney Kimmel Medical College of Thomas Jefferson University, Philadelphia, Pennsylvania

16:45–18:00

**Objective**: The effectiveness of placing a reinforcing transvaginal cerclage (RC) after the failure of a primary cerclage to prevent preterm birth is still poorly characterized, although several studies have examined this method. This meta‐analysis will determine the effectiveness of an RC to prevent preterm birth.

**Study Design**: Electronic databases were searched from inception to December 2023 for studies comparing RC to expectant management after initial cerclage placement and subsequent diagnosis of short cervix. RC was defined as a second cerclage placed ≥ 1 day after the first history‐, ultrasound‐, or physical exam‐indicated cerclage failed. Primary outcomes were gestational age (GA) at delivery and rate of preterm birth prior to 37 weeks. Subgroup analyses examined outcomes based on initial cerclage indication (history‐, ultrasound‐, or physical exam‐indicated). Results were summarized as weighted mean difference (MD) or risk ratio (RR) with 95% confidence intervals (CI). Heterogeneity was measured using Higgins I^2^.

**Results**: Out of an initial 7,433 identified titles, 8 studies met inclusion criteria. From a total of 440 patients, 129 had RC and 311 received expected management. Patients with RC delivered around 4 weeks earlier than the expectant management group (MD −3.98 weeks; 95% CI [‐5.29, −2.67], *p* < 0.001, I^2^ = 84%). Similarly, the rate of preterm birth was 83.5% in the RC group compared to 52.9% for expectant management (RR 1.46; 95% CI [1.03, 2.06], *p* = 0.03, I^2^ = 84%). Subgroup analyses showed an earlier GA at delivery in the cerclage reinforcement group compared to expectant management, regardless of initial cerclage type: history‐indicated (MD −5.94 weeks, 95% CI [‐7.83, −4.04], *p* < 0.001), ultrasound‐indicated (MD −5.33 weeks, 95% CI [‐8.18, −2.47], *p* < 0.001), physical exam‐indicated (MD −1.91 weeks, 95% CI [‐6.04, 2.21], *p* = 0.36).

**Conclusion**: There is no benefit to RC in preventing preterm birth for patients with failing initial cerclage placement. In fact, RC may be associated with earlier preterm birth compared to expectant management.

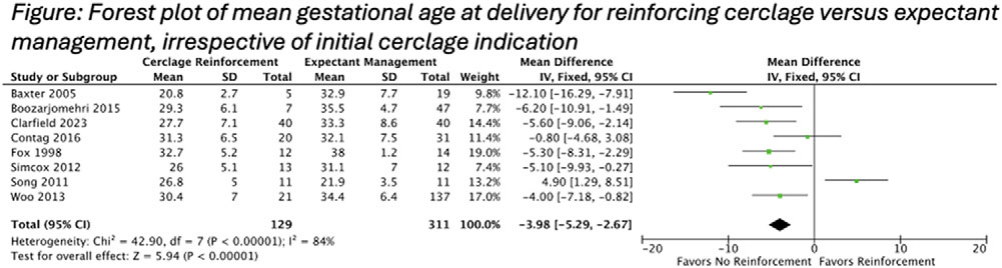

## 386 | A new angiogenic classification with PlGF and sFlt‐1 for hypertensive disorders of pregnancy

Valentina Giardini^1^, Alice Angela Francesca Santagati^2^, Marco Casati^3^, Arianna Pelucchi^2^, Patrizia Vergani^4^, Anna Cantarutti^5^, Anna Locatelli^2^

^1^Department of Obstetrics, IRCCS San Gerardo dei Tintori Foundation, University of Milano‐Bicocca, 20900 Monza, Italy, Monza, Lombardia; ^2^Department of Obstetrics and Gynecology, Unit of Obstetrics, Fondazione IRCCS San Gerardo dei Tintori, Monza, Lombardia; ^3^Laboratory Medicine, IRCCS San Gerardo dei Tintori, Monza, Lombardia; ^4^Department of Obstetrics and Gynecology, Unit of Obstetrics, Fondazione MBBM, Monza, Lombardia; ^5^Division of Biostatistics, Epidemiology and Public Health, Department of Statistics and Quantitative Methods, University of Milano‐Bicocca, Milan, Lombardia

16:45–18:00

**Objective**: The ratio between two angiogenic markers, sFlt‐1 (Soluble fms‐Like Tyrosine Kinase‐1) and PlGF (Placental Growth Factor), plays a crucial role in managing hypertensive disorders of pregnancy (HDP), notably preeclampsia (preE), aiding in diagnosis and outcome prediction. This study aimed to propose a novel angiogenic classification using PlGF and sFlt‐1 to enhance the assessment of maternal and/or fetal risk in pregnant women with an HDP.

**Study Design**: A retrospective study analyzed singleton pregnancies hospitalized for HDP after the 20th gestational week from May 2018 to December 2020. Patients were classified based on the sFlt‐1/PlGF ratio (low, medium, high and very high) and into nine new categories comparing the PlGF and sFlt‐1 levels to expected levels for gestational age (within range, above, or below): (1) PlGF and sFlt‐1 in the range; (2) PlGF in the range, sFlt‐1 below the range; (3) PlGF in the range, sFlt‐1 above the range; (4) PlGF below the range, sFlt‐1 in the range; (5) PlGF and sFlt‐1 below the range; (6) PlGF below the range, sFlt‐1 above the range; (7) PlGF above the range, sFlt‐1 in the range; (8) PlGF above the range, sFlt‐1 below the range; (9) PlGF and sFlt‐1 above the range.

**Results**: The cohort comprised 182 patients, with a mean gestational age at testing of 35+1 weeks (range 21‐41+1) and at delivery of 36+6 weeks (range 23+3‐41+3). Categories 1 (*n* = 54, 30%), 3 (*n* = 34, 19%), 4 (*n* = 19, 10%), and 6 (*n* = 69, 38%) were the most common. Category 6 patients had a statistically significant highest risk of adverse outcomes, including premature birth (75%), urgent cesarean section for HDP complications (48%), the necessity of antihypertensive therapy before and after delivery (64 and 67%), a higher percentage of growth‐restricted fetuses–FGR (59%), and severe clinical presentations (36%). Notably, 44% of low/medium‐risk patients classified by the ratio were high risk by the new algorithm.

**Conclusion**: This new PlGF and sFlt‐1 classification offers improved management of HDP compared to the current stratification.

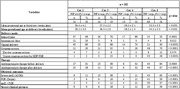

## 387 | Does a vegan diet impact pregnancy and neonatal outcomes? A retrospective case control study

Hadar Gluska^1^, Raud Qassim^2^, Maor Benjamin^3^, Shiri Margalit^4^, Michal Chana. Ovadia^5^, chen manor^6^, Tal Biron‐Shental^7^, Michal Kovo^6^

^1^Department of Obstetric and Gynecology, Meir Medical Center, Raanana, HaMerkaz; ^2^Helen Schneider Hospital for Women, Rabin Medical Center, Petach‐Tikva, Petach‐Tikva, HaMerkaz; ^3^Department of Obstetric and Gynecology, Meir Medical Center, Meir Medical Center, HaMerkaz; ^4^Tel Aviv University, Tel Aviv, Israel; ^5^The Department of Obstetrics and Gynecology, Meir Medical Center, Kfar Saba, HaMerkaz; ^6^Meir Medical Center, kfar saba, HaMerkaz; ^7^Meir Medical Center, Meir Medical Center, HaMerkaz

16:45–18:00

**Objective**: Ensuring a balanced diet during pregnancy is crucial for the health of both the mother and the newborn. Most studies in the field of pregnancy and nutrition focused on vegetarian diet. Although the prevalence of vegans has increased, data regarding its effects on pregnancy outcome are limited. We aimed to assess the impact of three different diets‐vegan (V), vegetarian (VG) and omnivore (OM)‐on pregnancy and neonatal outcomes.

**Study Design**: This retrospective case‐control study included all vegan parturitions with singleton pregnancy who gave birth between 2016 and 2022. For each vegan woman (V group) data from the next five consecutives parturients were collected. Maternal and obstetric characteristics were compared between the V, VG, and OM parturients.

**Results**: A total of 827 patients were included, with 140 in the V group, 272 in the VG group and 415 in the OM group. Maternal BMI (kg/m^2^), the rate of gestational diabetes and preterm delivery did not differ between the groups. The V group showed lower 1^st^ trimester glucose levels compared to the VG and OM groups (83.5 ± 9.0 g/dl vs. 86.7 ± 12.5 g/dl, vs. 85.1 ± 7.8 g/dl, respectively, *p* = 0.049). The OM group had the lowest Hb levels on admission and increased rate of hypertensive disorders compared to the V and VG groups (*p* < 0.001, *p* = 0.028, respectively).

Post hoc analysis pairwise comparisons revealed that the V group delivered at a higher gestational age (GA) (*p* = 0.003), with lower rates of labor induction (27.9% vs. 41.9% *p* < 0.001), compared to the OM group. Similarly, when compared to the VG group, the V group delivered at higher GA (*p* = 0.031), with lower rate of induction (27.9% vs. 43%, *p* = 0.007), and had higher rate of vaginal delivery (87.1% vs. 69.5%, *p* = 0.016), compared to the VG group. Neonatal birth weights, Apgar scores and the rate of neonatal hypoglycemia did not differ between the groups.

**Conclusion**: Following vegan diet during pregnancy can have a positive impact on pregnancy outcome. These findings could provide reassurance to women and their clinicians who choose plant‐based diet during pregnancy.

## 388 | Arterial placental mega‐jets as a reliable predictive sign for placenta accreta spectrum disorders

Homero Flores‐Mendoza^1^, John C. Kingdom^2^, Rory C Windrim^1^, Sebastian R. Hobson^3^

^1^Mount Sinai Hospital, Toronto, Mount Sinai Hospital, Toronto, Ontario; ^2^Dept OBGYN Mount Sinai Hospital University of Toronto, Toronto, Ontario; ^3^Mount Sinai Hospital, University of Toronto, Toronto, ON

16:45–18:00

**Objective**: To describe and explore a highly under‐reported sonographic sign, arterial placental mega‐jets, associated with placenta accreta spectrum (PAS) disorders and to evaluate its clinical usefulness in the prediction of this condition.

**Study Design**: This was a retrospective review of patients referred to a specialized Placenta Clinic with suspicion of PAS disorders for diagnosis and management, with clinical and histopathological confirmation. Prediction of this condition was done by evaluation of ultrasound descriptors by two maternal‐fetal medicine specialists, where ultrasound images were reviewed for the presence of arterial placental mega‐jets feeding into placental lacunae using spectral Doppler. A control group of 20 cases with placenta previa and placental lacunae with no PAS were assessed for the presence of this sonographic sign by the two same assessors.

**Results**: Arterial placental mega‐jets were present in 40 out of 48 confirmed cases of PAS. Sensitivity, specificity, positive predictive value, and negative predictive value were 83.8%, 100%, 94.3% and 95%, respectively. This sonographic finding was present in 1 out of the 20 control cases.

**Conclusion**: We have described the predictive value of a frequently under‐reported ultrasound descriptor for the prediction of PAS disorders. Our findings suggest that the presence of arterial placental mega‐jets feeding into placental lacunae may be a useful tool in the detection of this condition. Prospective evaluation of this sonographic sign would be beneficial, especially in routine clinical screening ultrasound settings to aid in decision‐making regarding referral to specialized centers.

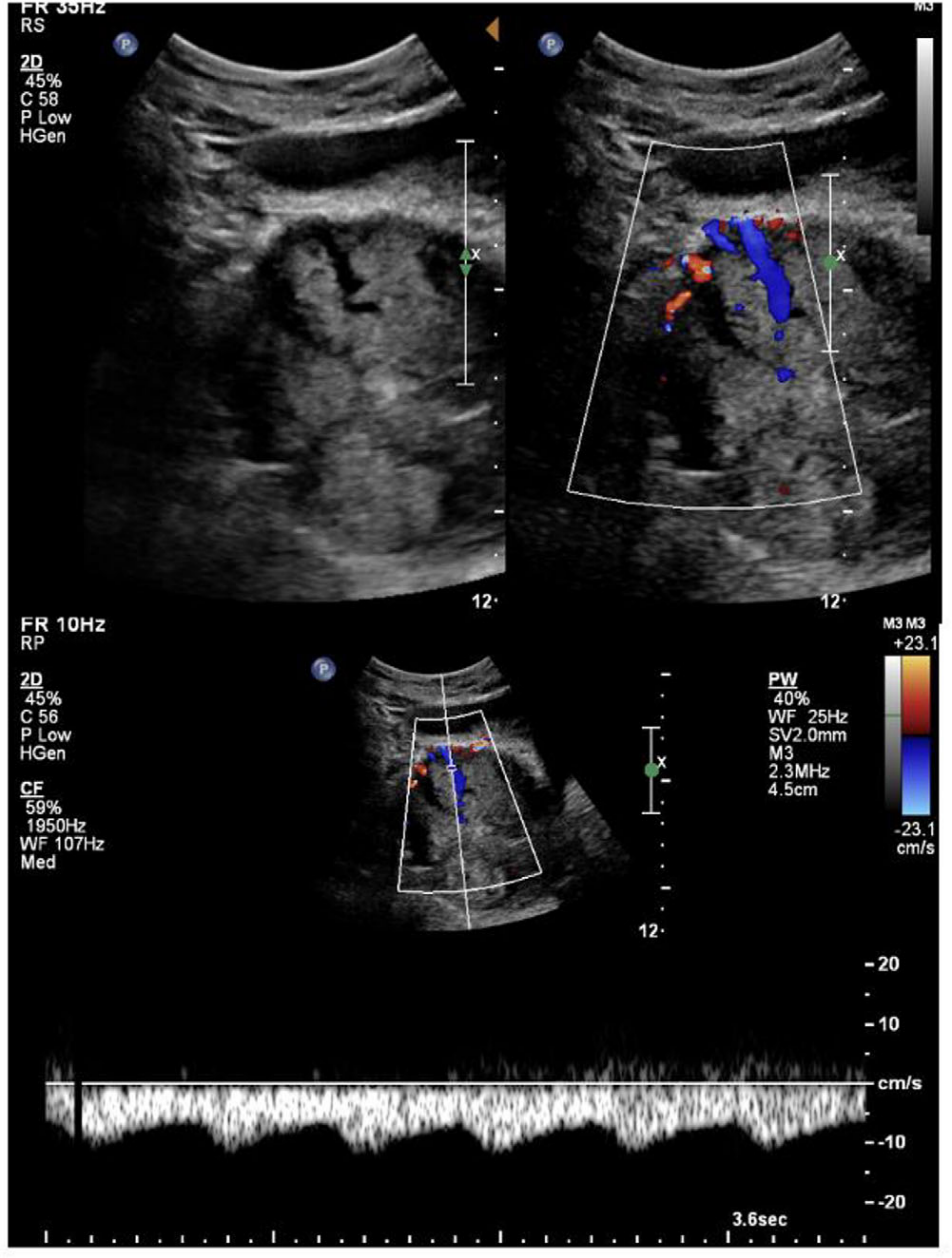

## 389 | Urologic morbidity following cesarean‐hysterectomy for placenta accreta spectrum disorder

Homero Flores‐Mendoza^1^, John C. Kingdom^2^, Rory C Windrim^1^, Ally Murji^1^, Lisa M Allen^1^, Anjana Ravi Chandran^1^, Nicholas Papalia^1^, Sebastian R. Hobson^3^

^1^Mount Sinai Hospital, Toronto, Mount Sinai Hospital, Toronto, Ontario; ^2^Dept OBGYN Mount Sinai Hospital University of Toronto, Toronto, Ontario; ^3^Mount Sinai Hospital, University of Toronto, Toronto, ON

16:45–18:00

**Objective**: Cesarean hysterectomy (C‐Hyst) at delivery for placenta accreta spectrum (PAS) disorder places the individual at significant risk of urologic morbidity. In some circumstances, unintentional or intentional cystostomy may occur and there is an ongoing risk of ureteral injury. It is not currently known whether the use of pre‐operative cystoscopy or temporary ureteral stenting is beneficial. We therefore evaluated the urologic morbidity outcomes and describe the surgical technique used for C‐Hyst for PAS in a large single center.

**Study Design**: Retrospective analysis of all patients with clinically‐ and histopathology‐confirmed PAS at a single Canadian center from 2014 to 2021. In 2014, we standardized our approach to the diagnosis and surgical management of PAS; including bilateral retroperitoneal dissection, ureterolysis, and meticulous bladder dissection without the routine use of either cystoscopy or temporary ureteral stenting. Descriptive urologic morbidity was defined as the presence of intentional or unintentional cystotomy, ureteral injury, or bladder fistula formation.

**Results**: 127 patients had a C‐Hyst for PAS of which 17 (13.4%) experienced urologic injury (bladder 13.4%, ureter 1.8%, bladder fistula 1.8%). Patients with urological injury had significantly higher median blood loss, a higher median units of blood products transfused and these individuals remained in hospital for a median 1 day greater postoperative stay compared with patients without urological injury (*p* < 0.001; *p* < 0.001 for all; *p* = 0.007, respectively). The rate of urologic injury was higher in patients with confirmed placenta percreta than with either placenta increta or accreta.

**Conclusion**: Our high‐volume center study demonstrated low rates of urologic injury at C‐Hyst for confirmed PAS disorders, achieved with minimal use of either pre‐operative cystoscopy or ureteric stent placement. Since most individuals had an intact urologic system at completion of their surgeries, our cohort had earlier mobilizations and favorable post‐operative recoveries in comparison with other contemporary centers.

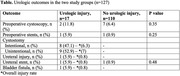

## 390 | Calculated allowable blood loss to predict transfusion in obstetric hemorrhage

Aaron W. Roberts^1^, Ipsita Ghose^2^, Rachel L. Wiley^3^, Hector M. Mendez‐Figueroa^4^, Suneet P. Chauhan^5^

^1^McGovern Medical School at UTHealth Houston, Houston, Texas; ^2^Baylor College of Medicine, Houston, Texas; ^3^University of California San Diego, San Diego, California; ^4^McGovern Medical School at UTHealth, Houston, Texas; ^5^Delaware Center of Maternal‐Fetal Medicine at Christiana Care, Delaware, Delaware

16:45–18:00

**Objective**: Guidelines for anesthesiologists suggest determination of “allowable blood loss” (ABL) using estimated blood volume (EBV), starting hematocrit (sHct), and threshold hematocrit (tHct) for transfusion. This method has not been validated in pregnant patients, whose physiology may alter its usefulness in postpartum hemorrhage (PPH). We aimed to determine if ABL could predict need for transfusion in obstetric patients.

**Study Design**: We analyzed our PPH database which collected all deliveries over two years at a Level IV center. The equation for ABL is: EBV x [(sHct–tHct)/sHct]. We used a tHct of 21%. Four variations of ABL were used with both 65 ml/kg EBV for nonpregnant and 100 ml/kg EBV for pregnant adult females, with and without adjustment for hemodilution. Test performance metrics were calculated using EBL > ABL as a positive test, and transfusion of PRBC as the outcome. Vaginal delivery and cesarean were also compared separately. There was no adjustment for variables not in the ABL equation.

**Results**: Of 8630 records analyzed, PPH with EBL > 1L occurred in 634 (7.3%) and 366 (4.3%) were transfused. All ABL equation variants had high specificity but poor sensitivity, resulting in a high positive likelihood ratio, poor negative likelihood ratio, and poor positive predictive value (Table 1). The most sensitive was the unadjusted variation 1. Of the 163 patients with EBL > ABL, this method identified 130/366 (35.4%) but missed 238 patients (64.8%) that required transfusion. There was little difference in test performance when comparing vaginal delivery vs cesarean.

**Conclusion**: ABL is not an effective tool to detect need for transfusion in obstetric patients, because sensitivity and PPV is poor and most patients that required transfusion were not identified with this method. However, a positive result (EBL > ABL) has a high specificity and likelihood ratio for transfusion. Simple tools that can be used to evaluate bleeding in obstetrics can be appealing. But caution should be used when applying these in obstetric patients. This method should only be used alongside, but not replace, clinical judgement.

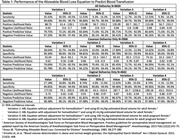

## 391 | Omics‐driven systems interrogation of metabolic dysregulation in selective fetal growth restriction and brain injury

Nana Huang^1^, Jing Yang^2^, Hai Jiang^1^, Yangyu zhao^3^

^1^Peking University Third Hospital, Peking University Third Hospital, Beijing; ^2^Department of Obstetrics and Gynecology, Peking University Third Hospital, BeiJing, Beijing; ^3^Obstetrics & Gynecology Department, Peking University Third Hospital, Beijing, Beijing

16:45–18:00

**Objective**: To investigate the correlation between maternal‐fetal metabolic imbalance and selective fetal growth restriction (sFGR) as well as fetal brain injury, aiming to develop an early prediction model and explore potential intervention strategies.

**Study Design**: This study was a longitudinal prospective cohort investigation, wherein maternal and fetal biological samples were collected from pregnancies with monochorionic diamniotic (MCDA) and sFGR twins during the first, second, and third trimesters. The neurocognitive behavioral development of the infants was monitored. The research encompassed an extensive utilization of targeted metabolomics, transcriptomics, placental pathology analysis, cell biology techniques, molecular biology methods, as well as neurobehavioral assessments to conduct a comprehensive systematic inquiry.

**Results**: The alterations in maternal metabolites during the first and second trimesters of pregnancy are associated with the occurrence of sFGR and the level of fetal physical and neurocognitive behavioral development at 2‐3 years of age. Mitochondrial stress phenotype is observed in sFGR‐derived HUVECs, indicating that secondary angiogenesis dysfunction primarily contributes to developmental imbalance in sFGR twins. Supplementation with citric acid can ameliorate oxidative stress injury, regulate vascular endothelial cell function, enhance fetal birth weight, and mitigate neuronal damage as well as long‐term neurobehavioral characteristics in offspring rats.

**Conclusion**: The alterations in maternal metabolites during the first and second trimesters exhibit a close association with both sFGR and fetal brain injury. Maternal‐fetal metabolic imbalance, oxidative stress, and placental vascular dysfunction play pivotal roles in the pathogenesis of sFGR and fetal brain injury, while citric acid demonstrates significant potential for enhancing fetal development.

## 392 | GLP‐1 mediated pregnancy and neonatal complications in mice

Michael J. Paidas^1^, Rajalakshmi Ramamoorthy^2^, Nila Elumalai^2^, Rima Hajjar^2^, Amirah B. Rashed^2^, Brian Z. Druyan^1^, Carmen Fernandez^2^, Arianna K. Carden^2^, Staci Marbin^2^, Bradley Safro^2^, Hussain Hussain^3^, Arumugam R. Jayakumar^4^

^1^University of Miami, Miami, Florida; ^2^University of Miami Miller School of Medicine, Miami, Florida; ^3^Larkin Community Hospital, Miami, Florida; ^4^University of Miami Miller School of Medicine, Miami, FL

16:45–18:00

**Objective**: Glucagon‐like peptide 1 (GLP‐1) is a hormone released by the intestine's enteroendocrine cells following food intake. It is known to have several critical regulatory functions that affect different organ systems, including the cardiovascular, neurological, gastrointestinal, and endocrine systems. Considering GLP‐1's ubiquitous effects, we aim to investigate its impact on pregnancy.

**Study Design**: Female A/J mice were injected (S.C.) with recombinant GLP‐1 (rGLP‐1, 1000 nmol/kg b.wt. once) on day one and day 15 of pregnancy. Mothers and Pups born to rGLP‐1 injection (at day 21 of pregnancy) were measured for their weight. Gross morphology and the number of deaths in pups born to mothers injected with rGLP‐1 and controls were evaluated. Data was analyzed using Tukey's multiple comparisons test by GraphPad 9.5.1 software.

**Results**: Pups born on day 21 to rGLP‐1 injection (one injection given on day 15 of pregnancy) die within 12 hours of birth and show considerable skin detachment uniformly all over the body (**B**) as compared to the non‐rGLP‐1 group (**A**). However, no significant gross morphological changes were observed in pups born to mothers who received rGLP1 immediately after pregnancy confirmation (Figure not shown). Further, the body weight of pregnant mice (**E**) is reduced post‐rGLP‐1. Similarly, pups born to mothers exposed to rGLP‐1 in early pregnancy (one‐day post‐exposure) or late pregnancy (15 days post‐exposure) lost weight (**C** and **D**) (*p* < 0.04 for pups from late pregnancy and *p* < 0.02 for pups from early pregnancy, respectively). We also identified a higher number of deaths in pups born to rGLP‐1 injected mice at a late stage, while no death was observed in mice when rGLP‐1 was given at early stages (**F**).

**Conclusion**: Our findings strongly suggest that rGLP‐1 consumption in the late pregnancy stages adversely impacts maternal and neonatal weight. Late prenatal exposure to rGLP‐1 was associated with neonatal death. Additional investigation is warranted into both experimental and clinical aspects of rGLP‐1 exposure and mechanisms involved in such maternal and perinatal effects.

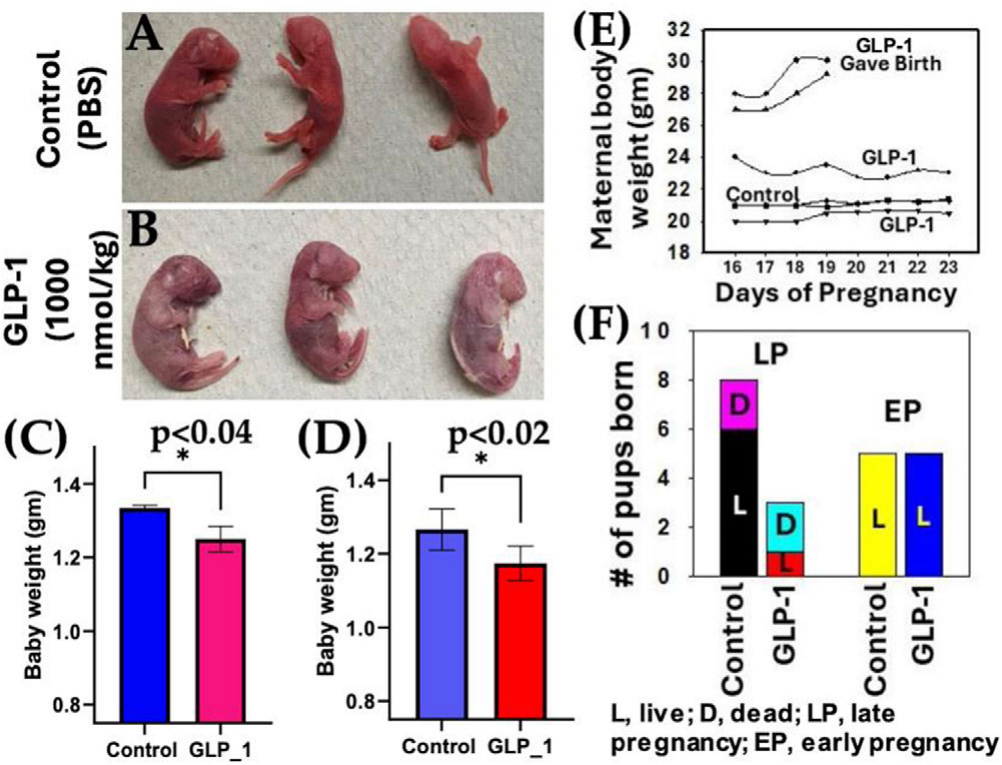

## 393 | HIV but not HCV or CMV facilitates trophoblast cell injury post SARS‐CoV‐2 infection in vitro

Brian Z. Druyan^1^, Amirah B. Rashed^2^, Nila Elumalai^2^, Arianna K. Carden^2^, Rajalakshmi Ramamoorthy^2^, Eva Hoffmann^2^, Hussain Hussain^3^, Michael J. Paidas^1^, Arumugam R. Jayakumar^4^

^1^University of Miami, Miami, Florida; ^2^University of Miami Miller School of Medicine, Miami, Florida; ^3^Larkin Community Hospital, Miami, Florida; ^4^University of Miami Miller School of Medicine, Miami, FL

16:45–18:00

**Objective**: The adverse impact of SARS‐CoV‐2 infection on placental pathophysiology is well documented. However, the role of simultaneous co‐infection with other viruses is poorly understood. This study investigates the effect of concurrent viral infections on cellular metabolic activity in human trophoblasts.

**Study Design**: HTR‐8/SVneo human trophoblasts were exposed to varying concentrations of SARS‐CoV‐2 nucleocapsid, cytomegalovirus (CMV), recombinant human immunodeficiency viral protein p24 (HIV‐1 p24), and recombinant Hepatitis C Virus (HCV) nucleoproteins, both individually and in combination. Metabolically active cells (MAC) viability was assessed using a cell proliferation/metabolism assay. Data analysis was conducted using Tukey's multiple comparisons test with GraphPad 9.5.1. Results were expressed as the percent change versus controls.

**Results**: Trophoblasts were exposed to 500 ng of viral proteins (once) and analyzed three days later. SARS‐CoV‐2 exhibited a 7.1% decrease in MAC, while exposure to HCV resulted in a 24.5% increase in MAC (*p* = 0.30). Interestingly, co‐exposure to HCV and SARS‐CoV‐2 mitigated the effect of SARS‐CoV‐2 on MAC.

Exposure to HIV led to a significant increase (49.5%) in MAC (*p* = 0.0006). However, co‐exposure to HIV and SARS‐CoV‐2 resulted in a pronounced decrease (54.1%) compared to HIV alone (*p* < 0.0001).

Moreover, trophoblasts exposed to CMV had a 17.7% increase in MAC (*p* = 0.58). Simultaneous exposure to CMV and SARS‐CoV‐2, however, resulted in a 15.3% decrease relative to CMV alone (*p* = 0.61).

**Conclusion**: Our results indicate that HIV exacerbates trophoblast cell injury following SARS‐CoV‐2 infection while HCV plays a protective role. This suggests the simultaneous presence of SARS‐CoV‐2 and other viruses may negatively or positively impact placental function and may confer additional adverse or beneficial effects on pregnancy outcomes. Further investigations are warranted to fully elucidate these critical findings.

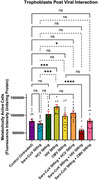

## 394 | Comparative assessment of antenatal growth charts in estimating abdominal circumference in indian population

SOUKHYA KARRI^1^, Marvel Angela Vijaya. Nallimelli^2^

^1^Lotus Hospital For Women And Children, Visakhapatnam, Andhra Pradesh; ^2^Logan Hospital, Brisbane, Queensland

16:45–18:00

**Objective**: To assess the difference in abdominal circumference centiles using three antenatal growth charts (GC) i.e., FMF, MEDISCAN AND HADLOCK GCs and to assess its clinical significance.

**Study Design**: The study included 797 ultrasounds in 481 patients. The AC centiles used in the dataset were obtained from the converter provided on FMF website, Mediscan from Sonocare software and Hadlock **V** for their respective GCs. Inclusion criteria were low risk, singleton pregnancies, gestational age between 18 to 40 weeks, availability of a dating scan between 8 to 14 weeks. All the women were of Indian origin. Exclusion criteria were major structural anomalies. The AC at two gestational age(GA) groups 18 −32 weeks and 32 to 40 weeks was compared between the three GCs i.e., FMF, MEDISCAN, HADLOCK and its clinical significance in identifying Small for Gestational Age fetus was noted.

**Results**: At GA of 18 −32weeks, 7.2% of fetuses had AC < 10 centile according to FMF GC, 2.9% according to MEDISCAN GC and 11.7% according to HADLOCK GC. At GA of 32‐40 weeks, 16.5% of fetuses had AC < 10 centile according to FMF GC, 2.7% according to MEDISCAN GC and 23.7% according to HADLOCK GC.

**Conclusion**: The study confirms the variation in AC centiles across the two gestational age groups in the three antenatal GCs.

The wide disparity among the GCs in identifying the small for gestational age fetus across both the gestational age groups poses a challenge to the clinician in assigning the fetus to be small for gestational age.

## 396 | Investigation of Novel Predictive Biomarkers for Preeclampsia in Second‐Trimester Amniotic Fluid

Yeonseong Jeong^1^, Jue Young Kim^2^, Hyo Eun Lee^3^, Min‐A Kim^2^

^1^FORSYTHIA410@yuhs.ac, Yonsei University College of Medicine, Seoul‐t'ukpyolsi; ^2^Yonsei University College of Medicine, Yonsei University, Seoul‐t'ukpyolsi; ^3^Yonsei University College of Medicine, Yonsei Univeristy, Seoul‐t'ukpyolsi

16:45–18:00

**Objective**: Preeclampsia (PE), a pregnancy‐specific hypertensive disorder, poses significant risks to maternal and fetal health. The principal aim of this study was to investigate potential markers for the early identification of women at risk of PE using amniotic fluid supernatant (AFS) samples before the onset of PE.

**Study Design**: AFS samples were obtained from 14 asymptomatic pregnant women (subsequently developing PE: 7, normal pregnancy: 7) undergoing amniocentesis for genetic diagnosis at 16 to 21 weeks of pregnancy. We analyzed differentially expressed genes (DEGs) between the control and PE groups via RNA sequencing of AFS before PE onset. Candidate genes were validated using reverse transcription polymerase chain reaction in HTR‐8/SVneo trophoblast cells exposed to fluid shear stress using the Ibidi pump system to mimic PE conditions.

**Results**: RNA sequencing revealed 19 DEGs, with 3 upregulated and 16 downregulated in the PE group. We chose HOOK2, CCDC160, CKB, and PARP15, which emerged as DEGs from the RNA sequencing data. To simulate PE conditions, HTR‐8/SVneo cells were cultured for 24 hours under various shear stress intensities (3, 10, or 20 dyn/cm^2^). HOOK2 expression significantly increased with increasing shear stress intensities (10 dyn/cm^2^; *p* < 0.0001, 20 dyn/cm^2^; *p* < 0.001). We explored the influence of HOOK2 knockdown on migration and invasion in HTR‐8/SVneo cells, finding that invasion activity significantly increased after HOOK2 knockdown compared to si‐control. Our results indicate that HOOK2 gene overexpression in PE‐conditioned HTR‐8/SVneo cells decreases trophoblast cell invasiveness. This finding implies that HOOK2 may serve as a potential marker for the early identification of women at risk of PE, given its involvement in regulating trophoblast cell invasion.

**Conclusion**: HOOK2 gene expression patterns offer promise as potential markers for early PE risk detection in pregnant women. Analyzing these profiles could enable clinicians to stratify women based on their PE likelihood, facilitating personalized monitoring and intervention strategies.

## 397 | Whole blood point‐of‐care Placental Growth Factor to predict serious adverse maternal and fetal outcomes

Katy Kuhrt^1^, Moses M'Bayoh^2^, Rossetta Cole^3^, Paul T. Seed^4^, Kate Bramham^5^, Andrew Shennan^4^

^1^King's College London, hartfield, England; ^2^Welbodi Partnership, Welbodi Partnership, Western Area; ^3^Ministry of Health and Sanitation, Ministry of Health and Sanitation, Western Area; ^4^King's College London, King's College London, England; ^5^King's College London, London, England

16:45–18:00

**Objective**: Hypertensive disorders of pregnancy, are the second leading maternal mortality globally, mainly due to delayed detection and definitive management (delivery). Placental growth factor (PlGF) testing improves time to Pre‐eclampsia diagnosis and maternal outcomes. Early delivery from 34 weeks’ can reduce stillbirth by 75%. Correct diagnosis is key when training and resources are limited. We evaluated the novel LEPZI® Quanti whole blood point‐or‐care (POC)‐PLGF, small, easy‐to‐use, with a result in ten minutes, for prediction of adverse maternal and neonatal outcomes in hypertensive women (24 to 34+6 weeks’) presenting to a maternity hospital in Freetown, Sierra Leone.

**Study Design**: Eligible women providing consent were offered a LEPZI® Quanti POC‐PLGF Test by trained research assistants. Results were blinded and management guided by routine clinical protocols. An optimal rule‐out threshold was determined based on sensitivities and specificities. POC‐PlGF prediction performance of predefined adverse outcomes (maternal death, eclampsia, stillbirth or a composite outcome of all three) at this threshold was assessed using sensitivity, specificity and positive and negative predictive values.

**Results**: 32/87 (36.8%) of pregnancies had at least one of maternal death, stillbirth or eclampsia. Key descriptors and outcomes are displayed in Table 1. The optimal rule‐out threshold was < or = 90, with 93.3% sensitivity for composite outcomes. Significantly more severe adverse events occurred with PlGF < or = 90 (44.6 vs 13.6%, *p* < 0.01). Some adverse events with PlGF >90 were unrelated to Pre‐eclampsia.

**Conclusion**: A whole blood POC‐PlGF test can be used in challenging LMIC environments. In hypertensive pregnancies admitted to hospital there is high sensitivity for severe adverse outcomes. This could aid identification and management (ie.: timing of delivery or conservative management where appropriate). More planned work will evaluate PlGF integration into clinical management pathways, and confirm optimal rule‐in and rule‐out thresholds.

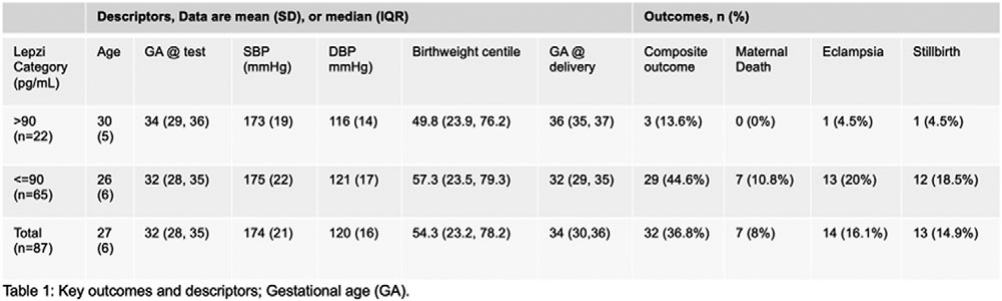

## 398 | Uterus conservative surgery for Placenta Accreta Spectrum ‐ Novel Surgical Approach and associated Outcomes

Hila Lahav Ezra^1^, Lior Yahav^2^, Michal Fishel Bartal^3^, Shali Mazaki‐Tovi^4^, Eran Kassif^4^, Eyal Sivan^4^, Elias Castel^1^

^1^Sheba Medical Center, Ramat Gan, HaMerkaz; ^2^Soroka Medical Center, Beer Sheva, HaDarom; ^3^UTH Houston & Sheba Medical Center Israel, Houston, Texas; ^4^Obstetrics and Gynecology, Sheba Medical Center, Tel‐Hashomer, Obstetrics and Gynecology, Sheba Medical Center, Tel‐Hashomer, Ramt‐Gan, HaMerkaz

16:45–18:00

**Objective**: The traditional approach of managing placenta accreta spectrum disorder in the United States is a cesarean hysterectomy. However, conservative management of PAS has become more popular due to the maternal morbidity associated with cesarean hysterectomy. Several techniques have been proposed and performed, including bilateral uterine artery ligation followed by separation of the placenta and resection of the defective uterine segment (LSR). The study aimed to describe maternal morbidity associated with two methods of conservative surgical approach in PAS management.

**Study Design**: A retrospective cohort study of all individuals who were prenatally diagnosed with PAS at a single referral care center between 2019‐2022. Individuals that were managed with the novel surgical approach, the LSR technique, which includes bilateral uterine artery ligation, placenta separation, and resection of the defective uterine segment (05/2021 to 12/2022) were compared to a control group managed prior to the implementation of the LSR technique (01/2019 to 04/2021). Outcomes assessed included intraoperative and postoperative complications, as described in the Table.

**Results**: During the study period, 135 patients were diagnosed with PAS and managed by the PAS multidisciplinary team; of them, 53 patients (39%) underwent the LSR technique, and 82 (61%) had other interventions. Maternal demographic and pregnancy complications did not differ between the groups. Individuals managed with LSR had a higher rate of severity of placental invasion as assessed by ultrasound prior to surgery. Blood transfusion rate was higher in the LSR group (73.6% vs 52.4%, *p* = 0.01), however, the rate of hysterectomy (21% with LSR, 17% with other interventions, *p* = 0.65), operative complications, and postoperative complications were similar between the groups.

**Conclusion**: In the cohort of individuals managed by a conservative surgical approach, only 1 of 5 required a hysterectomy. The novel LSR surgical approach for conservative management in placenta accreta spectrum patients appears to be a safe option for individuals with PAS.

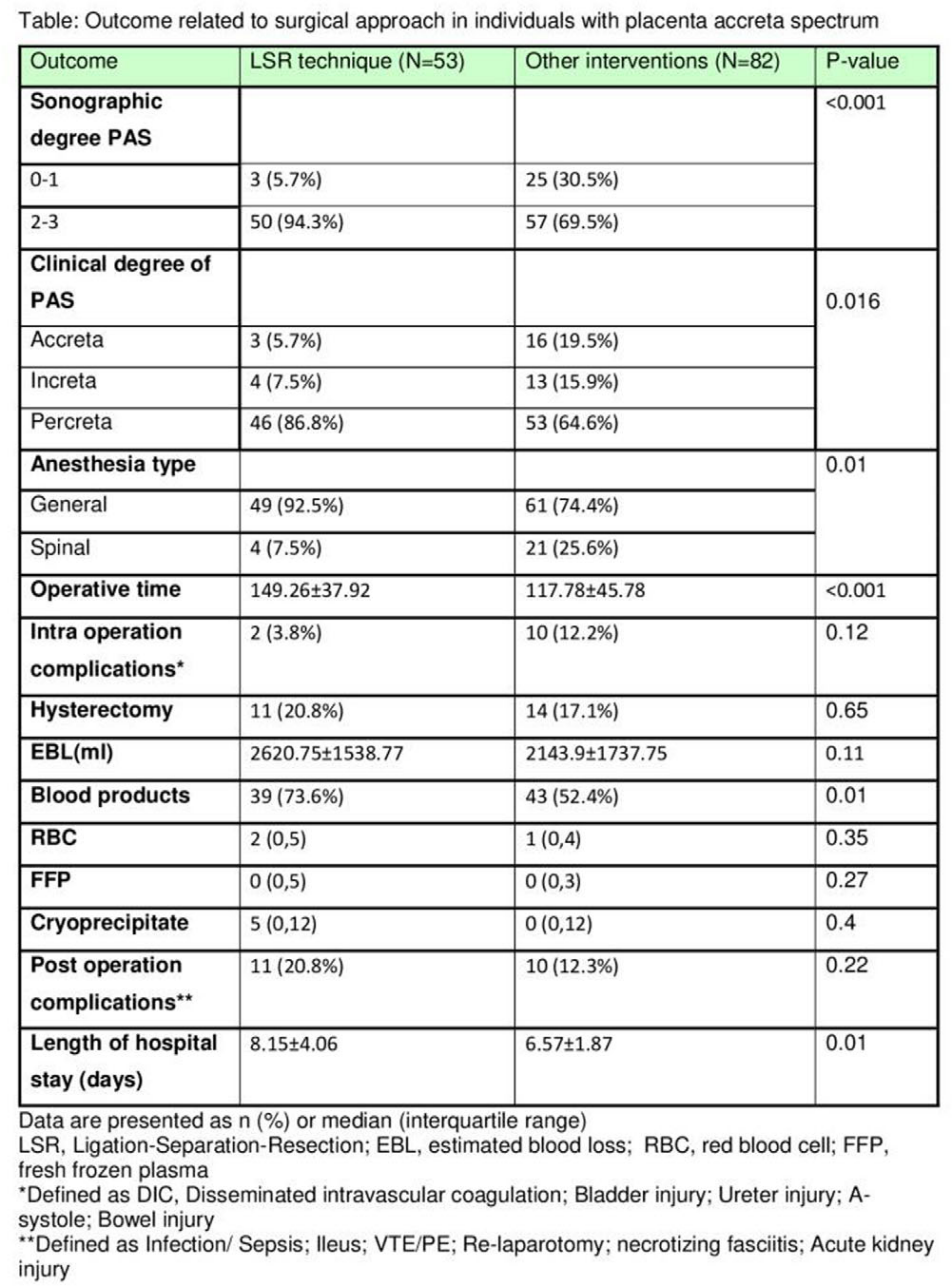

## 400 | The prognostic value of uric acid in preterm preeclampsia without severe features

Alberto Muniz Rodriguez^1^, Andrea Pelletier^2^, Jonathan Y. Siden^3^, Sarah Little^4^, Sarah Lassey^5^

^1^Mass General Brigham, Boston, MA; ^2^Brigham & Women's Hospital, Boston, Massachusetts; ^3^Massachusetts General Hospital, Harvard Medical School, Boston, Massachusetts; ^4^Beth Israel Deaconess Medical Center, Boston, Massachusetts; ^5^Mass General Brigham, Boston, Massachusetts

16:45–18:00

**Objective**: Preeclampsia (PE) has been associated with hyperuricemia, and higher uric acid (UA) levels may correlate with adverse obstetric outcomes. Limited data exists to define the prognostic value of UA in preterm PE without severe features. The objective of this study was to evaluate the association between UA and progression of disease in patients with PE without severe features undergoing expectant management.

**Study Design**: This was a retrospective cohort study of singleton pregnancies diagnosed with PE without severe features prior to 37 weeks at a single academic institution from 2019 to 2023. Patients with prior chronic hypertension were excluded. Serum biomarkers of PE were assessed at time of diagnosis and again on admission for delivery. Mean UA and highest recorded blood pressures were compared between patients expectantly managed until induction at term and those induced preterm due to development of severe features. Multivariate regression analysis was performed for prediction of severe disease.

**Results**: 97 patients were diagnosed with PE without severe features and expectantly managed. Of these, 45 (46%) were induced at term, 42 (43%) were induced preterm due to development of PE with severe features, and 10 (10%) delivered for another indication. There were no differences in demographic data between the groups. Mean gestational age at diagnosis was 35 weeks for those induced at term and 32 weeks for those who developed severe features preterm (*p* < 0.01). The mean UA at diagnosis was 4.7 mg/dL for the term group and 5.7 mg/dL for the group delivered preterm (*p* < 0.01). On multivariate regression analysis, GA and UA level at diagnosis were associated with development of severe disease (*p* > 0.01). The model showed good discrimination between induction at term and induction preterm (AUC = 0.719).

**Conclusion**: In the expectant management of PE without severe features, a higher UA level may be associated with increased risk for development of severe disease and could potentially be used for risk stratification at time of diagnosis.

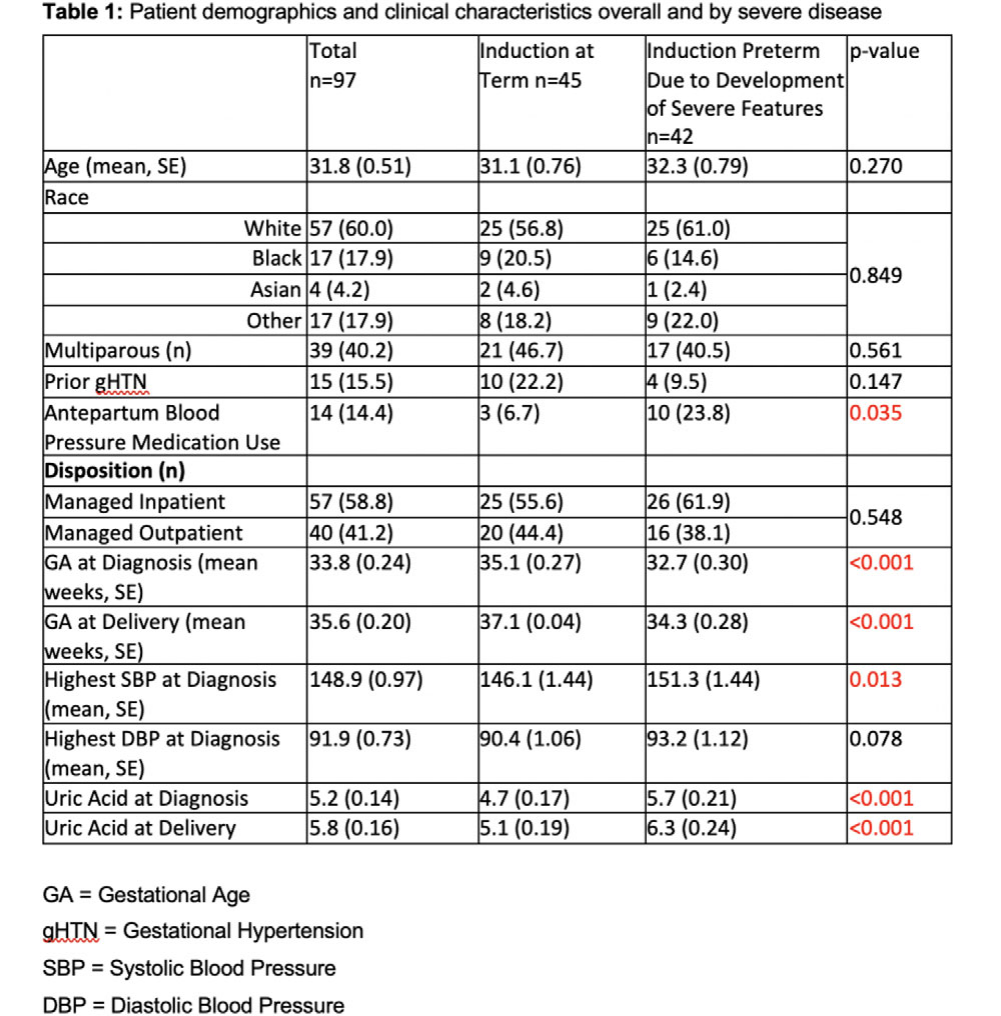

## 401 | The Impact of Placental Location on the Accuracy of Sonographic Estimated Fetal Weight at Term

Shiran Loewenstein^1^, Anat Pardo^2^, Or Bercovich^3^, Asaf Romano^4^, Natav Hendin^3^, Ohad Houri^3^, Eran Hadar^3^

^1^Helen Schneider Hospital for Women, Rabin Medical Center, petach tikwa, HaMerkaz; ^2^The Helen Schneider Hospital for Women at Rabin Medical Center, Petach Tikwa, HaMerkaz; ^3^Rabin Medical Center, Petach Tikva, HaMerkaz; ^4^Rabin Medical Center, Tel Aviv, HaMerkaz

16:45–18:00

**Objective**: Ultrasound measurements provide a noninvasive means of assessing fetal weight, crucial for antenatal preparations. Maternal and obstetric factors might influence the accuracy of ultrasound‐based. The precise influence of specific placental locations on discrepancies in estimated fetal weight (EFW) are not fully elucidated. We aimed to explore the association between placental location and disparities between sonographic EFW and actual birthweight (ABW).

**Study Design**: Retrospective data analysis from a single tertiary, university‐affiliated obstetric registry, between 2007‐2017. We enrolled singleton‐term pregnancies (37‐41 gestational weeks) with documented EFW within 14 days before delivery, while excluding missing data, placenta previa, or preterm delivery cases. Pregnancy groups were categorized into three placental location groups: 1) Anterior, 2) Posterior, and 3) Lateral. The primary outcome was an inaccurate EFW, defined as a difference exceeding 10% between sonographic EFW and ABW. A multivariate linear regression analysis was conducted to explore associations between placental location, age, and poly‐ or oligohydramnios.

**Results**: The analysis encompassed 16,113 deliveries, 8227 (51.1%) with an anterior placenta, 7622 (47.3%) with a posterior placenta, and 224 (1.6%) with a lateral placenta. Patients with lateral placenta were relatively older (Lateral 36.9 ± 5.1; Anterior 31.5 ± 5.2; Posterior: 31.4 ± 5.3 years; *p* < 0.001) and had higher rates of oligohydramnios (Lateral 15.6%; Anterior: 11.9%; Posterior: 9.5%; *p* < 0.001). Discrepancies of over 10% between EFW and ABW occurred less frequently for posterior placenta births (8.0%; Anterior: 8.6%; Lateral 8.7%; *p* = 0.039), a difference that persisted following multivariate regression analysis in comparison to anterior placenta location (OR 0.87, CI 0.79‐0.96 for posterior placentas).

**Conclusion**: Our findings indicate that placental location may influence the precision of EFW in singleton pregnancies. In women with a posterior placental location EFW is more accurate compared to anterior placenta locations.

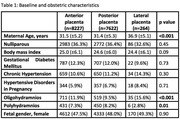

## 402 | Mifepristone and misoprostol in patients with a partial molar pregnancy: An option to consider?

Malou Lugthart^1^, Annemijn Aarts^2^, Elisabeth van leeuwen^3^, Eva Pajkrt^4^

^1^Amsterdam UMC, Amsterdam UMC, Noord‐Holland; ^2^Amsterdam UMC, locatie AMC, Amsterdam UMC, location AMC, Noord‐Holland; ^3^Amsterdam UMC, location AMC, Amsterdam UMC, location AMC, Noord‐Holland; ^4^Amsterdam UMC, Amsterdam, Noord‐Holland

16:45–18:00

**Objective**: We aimed to explore whether mifepristone and misoprostol can be offered without increasing the risk of GTN and other adverse outcomes as compared to curettage.

**Study Design**: We conducted a retrospective cohort study from 2000 to July 2023 in Amsterdam UMC. We included all patients with a prenatally diagnosed partial molar pregnancy (by ultrasound and DNA analysis) or diagnosed by DNA analysis and/or histopathological examination after management. We investigated the incidence of GTN between management with medication using mifepristone and misoprostol and curettage. Secondary outcomes were maternal death, pulmonary embolism, postpartum hemorrhage (≥1000 mL) and incomplete removal leading to subsequent surgical evacuation. Follow‐up time was at least six months.

**Results**: We included 57 patients with a partial molar pregnancy, 77.2% (44/57) diagnosed prenatally and 22.8% (13/57) diagnosed postnatally. Median gestational age at diagnosis was 12+6 weeks (IQR, 11+4–15+3 weeks). Mifepristone and misoprostol was used in 47.4% (*n* = 27) and curettage in 52.6% (*n* = 30). The median gestational age at management for mifepristone and misoprostol and curettage were 15+0 and 12+0 weeks’ gestation, respectively (*P* < 0.001). GTN did not occur in any of the patients. We did not find a significant difference between groups in postpartum hemorrhage or incomplete removal (22.2% versus 6.7%, *P* = 0.091), but in total more adverse outcomes occurred after mifepristone and misoprostol as opposed to curettage (44.4% versus 13.3%, respectively 95% CI −0.09–0.53, *P* = 0.009), especially in first trimester (14.3% versus 50.0% respectively, *P* = 0.03).

**Conclusion**: The use of mifepristone and misoprostol does not lead to an increased risk of GTN as compared to curettage. Patients with a partial molar pregnancy should be counseled on these different management options. The pros and cons of both options should be discussed, addressing the risk of postpartum hemorrhage and additional surgical evacuation, with first‐trimester curettage linked to fewer adverse outcomes

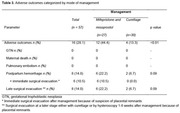

## 403 | Absorbable staples are associated with the lowest odds of post‐cesarean wound complications: A network meta‐analysis

Jordan A. McKinney^1^, Gustavo Vilchez^2^, Julio Mateus Nino^3^, Katherine Gonzalez^4^, Lifeng Lin^5^, Luis Sanchez‐Ramos^4^

^1^University of Florida College of Medicine, Gainesville, FL; ^2^UTHealth Houston, Houston, Texas; ^3^Wake Forest University School of Medicine, Concord, North Carolina; ^4^University of Florida College of Medicine, Jacksonville, Florida; ^5^University of Arizona, Tucson, Arizona

16:45–18:00

**Objective**: Although multiple randomized controlled trials (RCTs) and meta‐analyses have compared different methods for post‐cesarean skin closure, there is still not enough data to identify the best material to use, especially considering the variety of available closure techniques. To address this gap, we conducted a systematic review and network meta‐analysis of RCTs that compared the rates of wound complications associated with various skin closure methods.

**Study design**: We performed a computerized search of multiple electronic databases and gray literature to identify RCTs that compared various methods of post‐cesarean wound closure. The primary outcome was wound complications, with secondary outcomes including wound infection and separation. We employed a frequentist framework to compute odds ratios (ORs) accompanied by 95% confidence intervals (CIs). To identify the optimal material, we utilized measures such as the surface area under the curve (SUCRA), rank probabilities, and mean ranks.

**Results**: Thirty‐three RCTs (7,676 patients) compared the following treatments: absorbable staples (astaples), metal staples, glue, closure device, and suture. Concerning wound complications, astaples and sutures demonstrated the top SUCRA value (0.7) and the lowest mean ranks (2.3). Astaples led with a 43.2% probability of being optimal, with a pairwise OR (95% CI) of 0.83 (0.14–5.01). Regarding infections, both astaples and sutures had a SUCRA value of 0.6 and a mean rank of 2.8. Among these, astaples had a 32.6% chance of superiority, with a pairwise OR (95% CI) of 0.81 (0.07–9.66). For wound separations, the SUCRA values for astaples and sutures were 0.9 and 0.7, with mean ranks of 1.4 and 2.3, respectively. Astaples was the most favorable with a 78.8% probability.

**Conclusion**: Among patients undergoing cesarean delivery, using astaples for skin closure appears to be associated with the least complications compared to other methods studied. No significant statistical difference was found between astaples and sutures in pairwise comparisons.

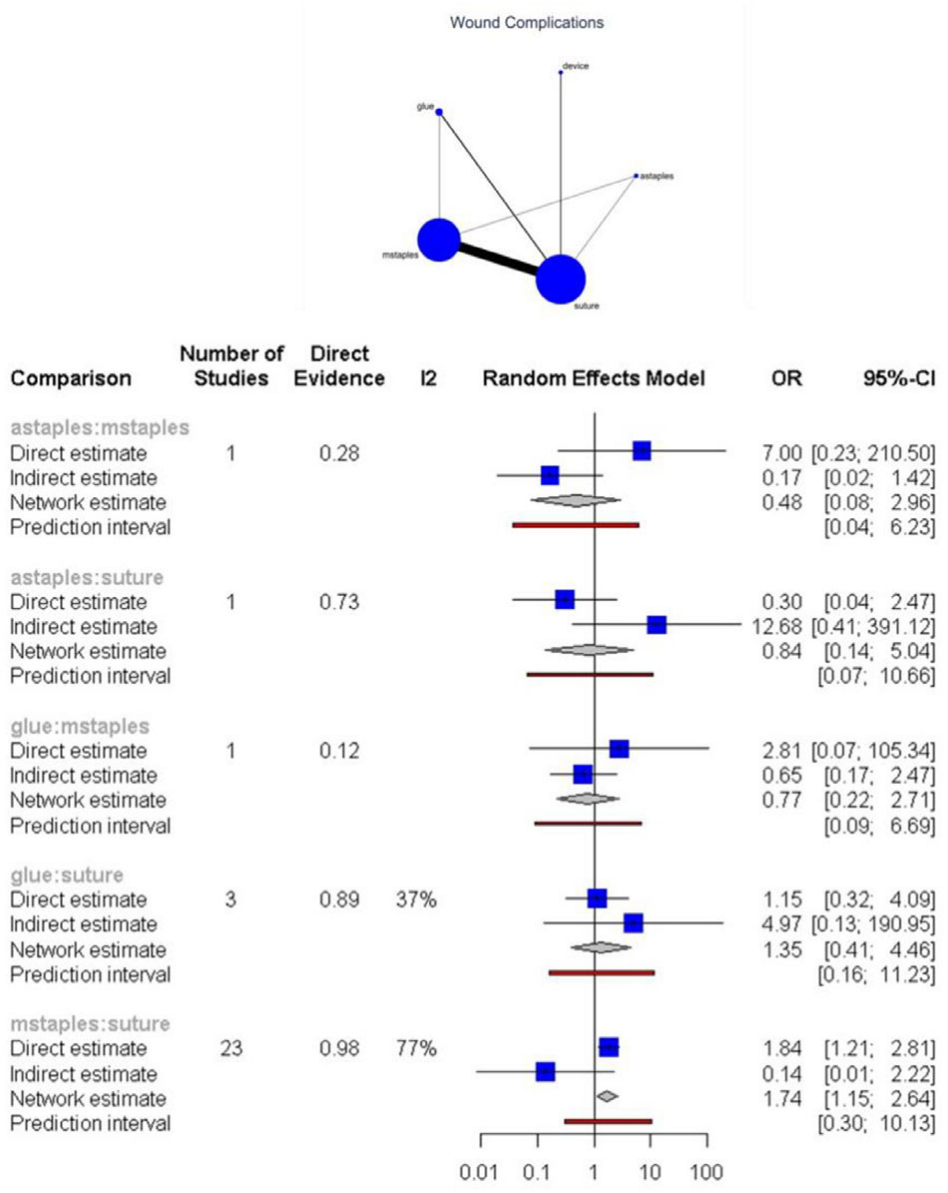

## 404 | Prophylactic antibiotics at the time of episiotomy repair do not improve wound outcomes: A meta‐analysis

Jordan A. McKinney^1^, Gustavo Vilchez^2^, Lifeng Lin^3^, Luis Sanchez‐Ramos^4^

^1^University of Florida College of Medicine, Gainesville, FL; ^2^UTHealth Houston, Houston, Texas; ^3^University of Arizona, Tucson, Arizona; ^4^University of Florida College of Medicine, Jacksonville, Florida

16:45–18:00

**Objective**: There is limited information available on the effectiveness of using prophylactic antibiotics after episiotomy repair. The previous meta‐analysis, which included only one randomized controlled trial (RCT), did not provide sufficient evidence. Moreover, the World Health Organization endorses routine antibiotic prophylaxis for women with third‐ or fourth‐degree perineal tears; however, this recommendation does not extend to women who require an episiotomy. Subsequently, several additional RCTs have been conducted. To address this gap, we conducted a thorough systematic review and meta‐analysis to assess whether prophylactic antibiotic administration reduces wound complications associated with episiotomy repair.

**Study Design**: We systematically searched multiple electronic databases and grey literature from their inception up to July 23, 2023, to identify RCTs that compared wound outcomes following the use of antibiotics to no use after episiotomy. The primary outcome was wound complications, with secondary outcomes including wound infection and separation. We performed a meta‐analysis employing random‐effects models to calculate odds ratios (OR) along with 95% confidence intervals (CI).

**Results**: This systematic review and meta‐analysis included data from 8 RCTs involving 2,716 participants. The Modified Jadad score indicated an overall low risk of bias across the included studies. The meta‐analysis showed that prophylactic antibiotics do not reduce the chances of wound complications (OR 0.88; 95% CI 0.51‐1.52; RFI 18), wound infection (OR 0.42; 95% CI 0.75‐1.35; RFI 7), or wound separation (OR 0.99; 95% CI 0.56‐1.75; RFI 15).

**Conclusion**: Our findings indicate that the use of prophylactic antibiotics during episiotomy repair following vaginal delivery does not reduce the likelihood of wound complications. These results emphasize that lacerations resulting from episiotomies should be managed similarly to spontaneous obstetric lacerations.

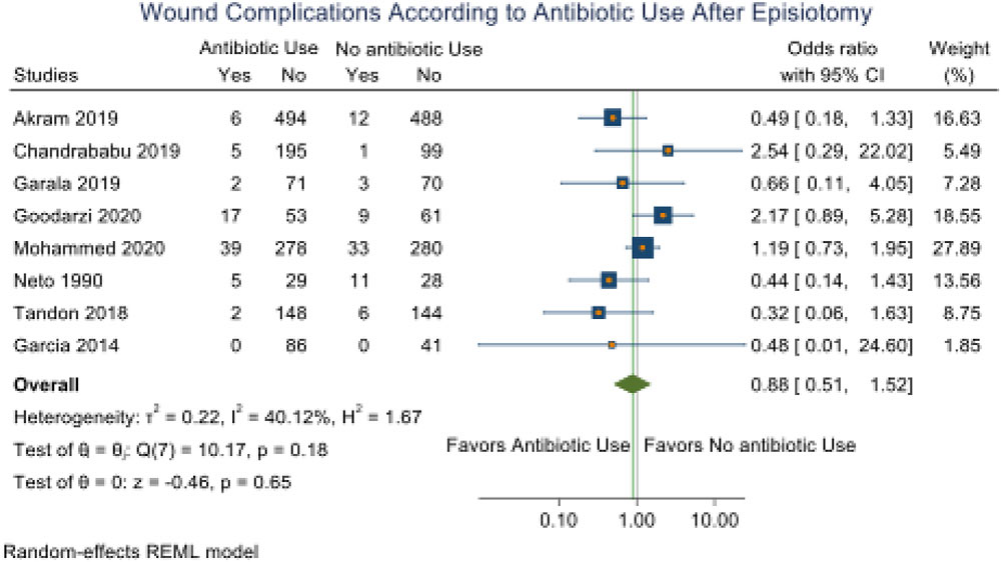

## 406 | The global burden of obesity and adverse perinatal outcomes

Julie A. Mina^1^, Jordan H. Perlow^2^

^1^University of Arizona COM‐Phoenix, Phoenix, AZ; ^2^University of Arizona COM‐Phoenix, University of Arizona COM‐Phoenix, Arizona

16:45–18:00

**Objective**: Evidence demonstrates that obese gravidae are at significantly greater risk for a multitude of serious adverse perinatal outcomes including stillbirth, fetal anomalies, and preeclampsia. The purpose of this study was to estimate the numbers of avoidable adverse outcomes, assuming the modifiable risk of obesity was eliminated. We also compared these most contemporary data to a limited look at this topic presented in abstract form based on data from ∼10 years ago.

**Study Design**: Countries included in this study are those of the original Group of Eight (G8). For each of the adverse pregnancy outcomes reviewed, the relative risk or odds ratios were derived from current systematic meta‐analyses examining the relationships of adverse pregnancy outcomes and maternal obesity. All data are derived for the year 2019. National incidence of adverse perinatal outcomes was obtained via Global Burden of Disease Database; 2019. Pre‐pregnancy BMI data was obtained or calculated for each country. The percent attributable risk for each adverse outcome was calculated for all countries. Perinatal adverse outcomes included congenital heart defects, stillbirths, infant deaths, neural tube defects, maternal sepsis, maternal hypertension, and neonatal encephalopathy.

**Results**: Across all adverse perinatal outcomes, the U.S. had the highest percent attributable to obesity and had the highest pre‐pregnancy BMI. A decrease in PAR was noted for congenital heart defects, infant deaths, and neural tube defects in countries that also had a decrease in obesity compared to prior data.

**Conclusion**: Thousands of stillbirths, infant deaths, congenital cardiac and neural tube defects, and hundreds of thousands of other serious obstetrical morbidities are potentially avoidable in the G8 countries with reductions in the prevalence of obesity during pregnancy. We believe these data emphasize the need for initiation of global and culturally sensitive public education regarding the risks of obesity and potential for causing these tragic outcomes. Furthermore, given these data, we advocate for broader access and to meaningful preconception care.

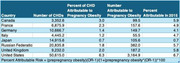

## 407 | Risk Factors Associated with Severity of Laceration among Women with ≥3 Obstetric Anal Sphincter Injury

Olena Minich^1^, shanny kolp asis^2^, Elad Miron^3^, Anath Beck^3^, Adi Ashkenazi Katz^3^, Tata Coblic‐Zelter^3^, Olena Minich^1^

^1^Tel Aviv University, Kfar Saba, HaMerkaz; ^2^Tel Aviv university, Tel aviv, HaMerkaz; ^3^Tel Aviv University, Tel Aviv University, Tel Aviv

16:45–18:00

**Objective**: High‐degree perineal tears remain a significant obstetric complication with short and long‐term morbidity for women after delivery. Grade 3a obstetric anal sphincter injury (OASIS) is considered less severe and women may be offered a repeated vaginal delivery if desired. We seek to describe factors associated with having grade 3a OASIS vs grade ≥3b OASIS.

**Study Design**: A retrospective analysis of a single university‐affiliated center with 12000 deliveries/year. Included were women that had ≥3rd‐degree perineal lacerations during vaginal deliveries between the years 2017‐2021. Within the cohort, we looked at factors associated with having either grade 3a OASIS or above. We performed univariate and multivariate analysis to examine predictors associated with having ≥3b lacerations.

**Results**: Overall, 50442 deliveries took place during the study period. A total of 271 (women had grade ≥3rd OASIS (0.005%). The median age was 24 (IQR 22‐27) and the median week of gestation was 40 (IQR 39‐40.5), 206 women had spontaneous vaginal delivery (76%) and the remaining had assisted vaginal delivery. Twenty‐eight women were grand multipara (>3 previous deliveries). Ninety‐two patients had ≥3b lacerations (33.9%), No difference was found between the severity of the laceration and being grand multipara. Birth weight of ≥3800g was more commonly found in the ≥3b laceration group (27.2% vs 16.2%, *p* = 0.037), **Table 1**. On univariate and multivariate analysis examining age, birth weight, type of birth (vaginal/assisted vaginal delivery), parity, episiotomy, vertex presentation, newborn sex, recipient of induction, and length of 2nd phase >3 hours. Age (odds ratio [OR] 1.27 [1.1‐1.49], *p* = 0.002) and birth weight ≥3800g (OR 3.11 [1.14‐8.81], *p* = 0.028) were found as predictors of having ≥3b laceration on multivariate analysis.

**Conclusion**: High‐grade perineal lacerations rarely occurred within our cohort, increased age and birth weight ≥3800g were found to be predictors of having grade ≥3b laceration. Further research is warranted to validate these results.

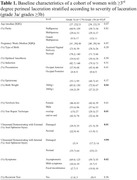

## 408 | Association between Intrapartum Fetal Oxygen Saturation and Adverse Perinatal Outcomes; A Systematic Review and Meta‐Analysis

Jill M. Mitchell^1^, Siobhan Walsh^1^, Laura O'Byrne^1^, Virginia Conrick^2^, Ray Burke^3^, Ali S. Khashan^4^, John R. Higgins^5^, Richard Greene^6^, Gillian M. Maher^4^, Fergus P. McCarthy^1^

^1^Department of Obstetrics and Gynaecology, University College Cork, Cork, Cork; ^2^UCC Library, University College Cork, Cork, County Cork, Ireland, Cork, Cork; ^3^Tyndall National Institute, University College Cork, Cork, Cork; ^4^School of Public Health, University College Cork, Cork, Cork; ^5^Massachusetts General Hospital, Boston, Massachusetts; ^6^National Perinatal Epidemiology Centre, University College Cork, Cork, Cork

16:45–18:00

**Objective**: To assess the association between abnormal intrapartum fetal oxygen saturation (FSp02) and perinatal and long term behavioural outcomes.

**Study Design**: Randomised control trials, non‐randomised control trials, cohort and case‐control studies were included. Population of interest included women in labour with a cephalic baby. Two interventions were reviewed; 1) Low FSp02 defined as < 30%, and 2) the use of fetal pulse oximetry during labour as a means of measuring intrapartum FSp02.

A comprehensive literature search was conducted in PubMed, EMBASE, CINAHL, The Cochrane Library, Web of Science, ClinicalTrials.Gov and WHO ICTRP from inception of database to February 2024.

Independent reviewers screened studies, extracted data, and assessed quality using the Risk of Bias tool and Newcastle‐Ottawa Scale. The GRADE approach was used to evaluate the certainty of the evidence. A random‐effects meta‐analysis was performed following PRISMA and MOOSE guidelines.

**Results**: From 3,280 articles, 47 studies with 13,071 mother‐infant pairs were included. FSpO2 < 30% was associated with umbilical artery pH < 7.15 (OR = 7.86, 95% CI = [3.29‐18.75], I^2^ = 71%, *p* = 0.0007), 5‐minute Apgar score < 7 (OR = 16.63, 95% CI = [5.64–49.01], *p* < 0.00001, I^2^ = 30%) and admission to the neonatal intensive care unit (OR = 5.89, 95% CI = [1.73–20.01], *p* < 0.005, I^2^ = 0%). The addition of FSp02 monitoring to fetal heart rate monitoring was associated with lower odds of Caesarean section for non‐reassuring fetal status (OR = 0.59, 95% CI = [0.40, 0.86], I^2^ = 71%, *p* = 0.006) without impacting neonatal outcomes in terms of 5 minute Apgar < 7 (OR = 0.66, 95% CI = [0.37, 1.17], I^2^ = 0%, *p* = 0.16) and neonatal intensive care unit admissions (OR = 0.98, 95% CI = [0.82, 1.18], I^2^ = 0%, *p* = 0.84).

**Conclusion**: FSp02 combined with fetal heart rate monitoring may reduce unnecessary intrapartum Caesareans for suspected fetal distress without affecting short‐term neonatal outcomes. The link between FSpO2 < 30% and adverse perinatal outcomes supports its potential as a valuable adjunct in intrapartum monitoring.

## 410 | Integrative medicine during labor induction for nulliparous patients — A retrospective trial

Tzlil Ganon^1^, Mays awawdeh^2^, Bat‐Zion Zidenberg^1^, Dani Steinberg^1^, Shlomi Sagi^3^, Elad Schiff^1^, Rami sammour^3^, Inna Bleicher^2^

^1^Bnai‐Zion MC, Haifa, Hefa; ^2^Bnai Zion Medical Center, Haifa, Hefa; ^3^Bnai Zion MC, Bnai Zion MC, Hefa

16:45–18:00

**Objective**: Complementary medicine has progressively integrated into clinical practice; however, the existing literature on its efficacy and safety in modern obstetrics remains insufficient. This study aims to evaluate the utilization of oxytocin during labor among nulliparous patients undergoing labor induction, comparing those who received complementary medical interventions to those who did not.

**Study Design**: This was a retrospective cohort study, retrieved from electronic medical records of nulliparous patients admitted for labor induction in a single tertiary medical center. Patients were included if they were ≥ 34 weeks of gestation, vertex presentation, and no contraindications for vaginal delivery. Patients who experienced spontaneous labor and patients with non‐live birth were excluded. Patients who received acupuncture or reflexology during labor process (integrative treatment group) were compared to patients who had no such intervention (control group). Primary outcome was the rate of oxytocin utilization and oxytocin duration. Secondary outcomes included mode of delivery and other maternal and neonatal outcomes.

**Results**: A total of 867 patients met inclusion criteria, 227 received integrative treatments. Patients in the integrative treatment group were slightly younger and less likely to have induction of labor due to intrauterine fetal growth restriction.

The rate of oxytocin utilization and oxytocin to delivery interval were similar between groups. Integrative treatments were associated with lower rates of instrumental vaginal delivery (5.3% vs. 11.3%, *p* = 0.018, OR 2.1, 95% CI 1.1‐3.8) and lower rates of non‐reassuring fetal heart rate as indication for immediate delivery (Table 1).

**Conclusion**: Integration of acupuncture and reflexology during labor induction had no effect on oxytocin utilization, but was associated with lower rates of instrumental vaginal delivery and lower rates of fetal distress as indication for prompt delivery. Further prospective randomized studies are needed to confirm these findings.

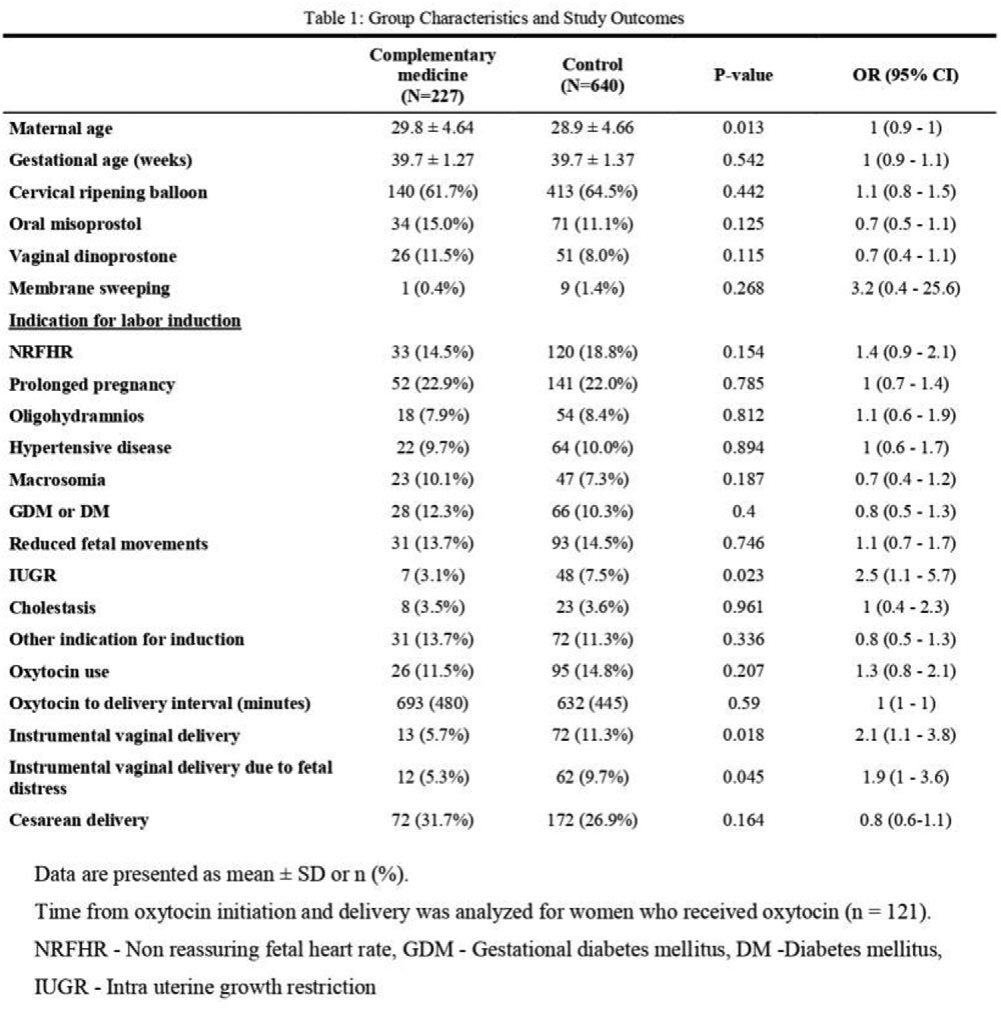

## 411 | Sequence of events leading to medical abortion for fetal indications after 34 weeks of gestation

Marina Pekar Zlotin^1^, Yael Nehama Berman^2^, Yaakov Melcer^2^, Howard Cuckle^3^, Ron Maymon^4^

^1^shamir medical sener, shamir medical center, HaMerkaz; ^2^shamir medical center, shamir medical center, HaMerkaz; ^3^Department of Obstetrics and Gynecology, Faculty of Medicine, Tel Aviv University, Tel Aviv, Israel., Department of Obstetrics and Gynecology, Faculty of Medicine, Tel Aviv University, Tel Aviv, Israel., HaMerkaz; ^4^Department of Obstetrics and Gynecology, Shamir (Assaf Harofeh) Medical Center, Zerifin, Tel Aviv

16:45–18:00

**Objective**: To identify the sequence of events leading to abortion ≥ 34 weeks of gestation, and determine if the procedure could have been carried out earlier

Method: This is a retrospective study of fetal abortions in singleton pregnancies carried out in our department during the 23‐year period, 1998‐2021.

**Study Design**: This is a retrospective study of fetal abortions in singleton pregnancies carried out in our department during the 23‐year period, 1998‐2021.

**Results**: 36/4055 (0.88%) abortions were carried out ≥ 34 weeks of gestation. The indications were anatomical in 20 (55%), chromosomal or genetic in 14 (39%) and 2 others (CMV infection). From evaluation of the sequence of events, earlier diagnosis in 18 cases (50%) was unlikely before the third trimester because the disorder was developmental and ultrasound findings are not expected earlier. In one of these and 5 other cases (17%) (14%) the delay was due to patient choice, less than full adherence to screening tests and procedures accounted to 6 more (17%). The reason for delay in the remainder was not determined

**Conclusion**: In our series, full adherence to local screening tests and protocols, and timely decision making could have substantially reduced the late abortion rate.

## 412 | Postpartum psychosis: Could chronic sleep deprivation be protective?

Chiara Petrosellini^1^, Sofia Eriksson^1^, Olivia Protti^1^, Dimitrios Siassakos^2^, Andrew McQuillin^1^

^1^UCL, UCL, England; ^2^Elizabeth Garrett Anderson Institute for Women's Health, University College London, London, England

16:45–18:00

**Objective**: Postpartum Psychosis (PP) is a severe but treatable perinatal mental illness. Women with pre‐existing Bipolar Disorder (BD) have a significant risk of PP following childbirth, but individualised prediction of perinatal relapse is challenging.

Sleep disturbance is almost universal in the perinatal period. It is a well‐established trigger of psychosis and one of the earliest and most common manifestations of PP. A relationship between sleep disruption and PP is often assumed but is poorly understood.

Genome‐wide association studies of complex traits can be used to derive Polygenic Risk Scores (PRS), which can be used to estimate an individual's susceptibility to disease. We aimed to establish whether PRS of sleep traits can be used to improve prediction of PP in women with BD.

**Study Design**: Women with BD were recruited from general practices, hospitals and community mental health teams in England. Participants who have given birth were screened for perinatal psychiatric complications. 117 women with BD who developed PP and 226 women who did not develop PP following childbirth were compared in our analyses.

Polygenic Risk Scores (PRS) were computed using summary statistics of Genome‐Wide Association Studies of Insomnia (INS), Short Sleep (SS), Long Sleep (LS) and Sleep Efficiency (SE). Logistic regression was used to model the effect of each PRS on PP following childbirth.

**Results**: INS PRS and SS PRS were significantly lower in women who developed PP compared to those who did not develop PP following childbirth. Long sleep and Sleep Efficiency PRS were not associated with PP but were underpowered to detect a significant effect.

**Conclusion**: Genetic vulnerability to insomnia and short sleep contributes to an increased risk of chronic sleep deprivation. This may foster a heightened tolerance to sleep disruption, mitigating the impact of childbirth on mood stability. A higher individual PRS for INS and/or SS may therefore be protective against PP in women with BD. PRS can improve outcome prediction and support perinatal decision‐making in women with mental illness.

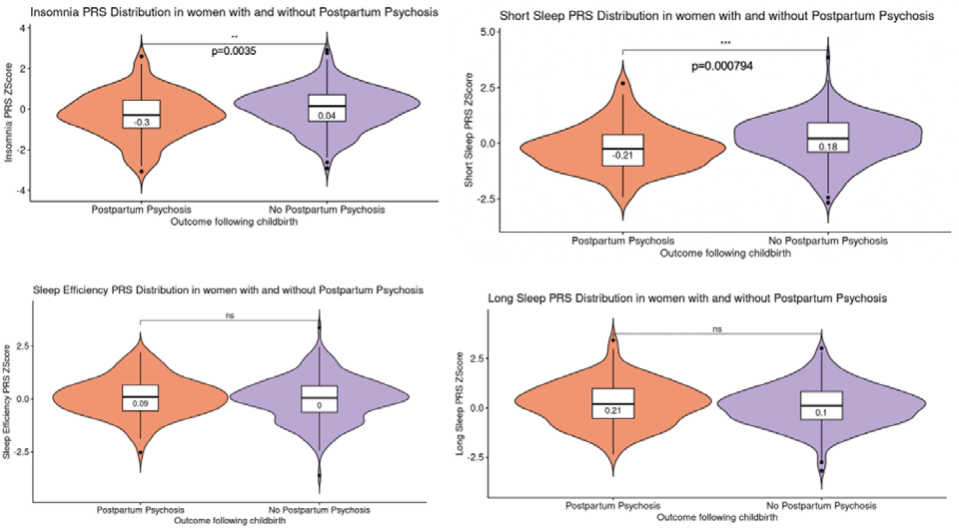

## 413 | Effect of gestational age in neonatal outcome following intrauterine transfusion: A retrospective study

Mandakini Pradhan^1^, Yashika Gadela^2^, Neeta Singh^2^, Sangeeta Yadav^2^

^1^Sanjay Gandhi Post Graduate Institute of Medical Sciences, Lucknow, Uttar Pradesh; ^2^Sanjay Gandhi Post Graduate Institute of Medical Sciences, Sanjay Gandhi Post Graduate Institute of Medical Sciences, Uttar Pradesh

16:45–18:00

**Objective**: To determine if gestational age at first transfusion has effect on perinatal outcome in pregnancies requiring intra uterine transfusion (IUT) for severe red blood cell (RBC) alloimmunization.

**Study Design**: This was a retrospective analysis of 953 IUT done in 345 patients in a single center between January 2010 to December 2023. Timing of the first IUT was based on evaluation of either the middle cerebral artery peak systolic velocity (MCA‐PSV) or development of fetal hydrops. Patients were divided into group I (less than or equal to 24 week, *n* = 105) and group II (more than 24 weeks, *n* = 240). Procedure related complications and perinatal outcome were compared in both the group.

**Results**: Affection of at least one previous pregnancy was found in 75.3% cases. The median gestational age at the first IUT was 23 weeks and 30.5 weeks in group I and II respectively. Mean hematocrit (HCT) level was 12.7% (range 2.8%‐21.7%) in group I and 22% (range 1.75%‐28%) in group II. Hydrops fetalis was detected in 38% (*n* = 40) of group I and 25% (*n* = 60) of group II fetuses. In addition to antibody against Rh D, at least one additional antibody was present in 97 (28.1%) women and comparable in each group. Fetal transfusion was through the intrahepatic vein in 36.1% and umbilical vein in 63.9%. Median gestation at delivery was 35.2 weeks in group I and 35.6 weeks in group II. There were 5 intrauterine fetal death in group I and 19 losses in group II. Overall perinatal survival rate was 87.6% and 92% in group I and II respectively. Survival in hydropic group was 85% and 78.3% in group I and II respectively. A subgroup analysis of survival in less than 20 weeks (*n* = 12), 20‐22 weeks (*n* = 22) and between 22‐24 weeks (*n* = 71) did not show any difference.

**Conclusion**: Fetal survival following IUT is not influenced by gestational age at first IUT. IUT is feasible even before 20 weeks if required. Information about perinatal survival rate of more than 86% can be given to the couple.

## 414 | Treatment Patterns in Abnormal Postpartum Uterine Bleeding or Hemorrhage (2016‐2022)

Patricia I. Carney^1^, Candice Yong^2^, Brian Seal^2^, Seungyoung Hwang^2^, Kara M. Rood^3^, Roy Brooks^4^

^1^Organon, Organon/Jersey City, NJ; ^2^Organon, Organon/Jersey City, New Jersey; ^3^The Ohio State University, Columbus, Ohio; ^4^Capital Women's Care, Capital Women's Care/Laurel, Maryland

16:45–18:00

**Objective**: Postpartum hemorrhage (PPH) is a leading cause of maternal morbidity and mortality worldwide. We describe rates of abnormal postpartum uterine bleeding (PPAUB)/PPH over time and treatment patterns for uterotonics (UTs), tranexamic acid (TXA), and other interventions among hospital births involving PPAUB or PPH.

**Study Design**: This was a retrospective, observational study of all hospital births in patients ≥15 years old associated with PPAUB or PPH in the US Premier Healthcare Database (January 2016‐March 2022). PPH was defined by ≥1 ICD‐10 code. Second‐line UT use beyond oxytocin, or TXA use, was a proxy for PPAUB.

**Results**: Among 5,345,753 births, 14.7% (*n* = 787,964) had a PPH diagnosis or received second‐line UTs and/or TXA, of which 3.4% had PPH ± UT/TXA use and 11.3% had UT/TXA use without a PPH diagnosis. Mean age was 29.0 years, 50.7% were non‐Hispanic white and 34.1% of births were cesarean. The observed rate of PPAUB or PPH increased from 12.1% in 2016 to 19.7% in 2022. Increased use of the PPH ICD‐10 diagnosis code (2.7% in 2016, 4.3% in 2022) contributed to the increase in PPAUB/PPH during the observation period. Methylergonovine use decreased from 53.3% in 2016 to 46.1% in 2022, whereas use of misoprostol ≥800 mcg (48.9% in 2016, 46.2% in 2022) and carboprost (14.6% in 2016, 15.0% in 2022) was more stable. Tranexamic acid use increased from 0.3% in 2016 to 27.8% in 2022. Combination therapy with 2 or more UTs/TXA increased, from 25.6% in 2016 to 33.4% in 2022. Nonsurgical treatment with uterine balloon tamponade ranged from 2.4% in 2016‐2017 to 2.9% in 2019 and 2021. Of patients who received balloon tamponade + UTs/TXA, the proportion receiving 3 or more UTs/TXA increased from 26.6% in 2016 to 52.4% in 2022. Rates of hysterectomy were 0.6% in 2016‐2017, 0.4% in 2018‐2021, and 0.3% in 2022.

**Conclusion**: The observed rate of PPAUB or PPH was relatively high and increased from 2016 to 2022. Treatment patterns evolved during the observation period, with use of methylergonovine decreasing and use of TXA and combination therapy increasing.

## 415 | Preventing accreta by optimizing prior cesarean uterine closure techniques: A systematic review

Megan B. Raymond^1^, Julie Gomez^2^, Federica Cortese^3^, Dante F. Varotsis^4^, Vincenzo Berghella^5^

^1^Thomas Jefferson University, Philadelphia, Pennsylvania; ^2^Thomas Jefferson University, Philadelphia, PA; ^3^Thomas Jefferson University Hospital, Rome, Lazio; ^4^Thomas Jefferson University, Thomas Jefferson University/Philadelphia, PA; ^5^Sidney Kimmel Medical College of Thomas Jefferson University, Philadelphia, Pennsylvania

16:45–18:00

**Objective**: Placenta accreta spectrum (PAS) is a significant contributor to maternal morbidity worldwide and has a rising incidence, with an overall rate as high as 1 in 272 pregnancies in the United States. Our objective was to review studies looking at how cesarean delivery (CD) uterine closure techniques may affect the development of PAS in a subsequent pregnancy.

**Study Design**: A systematic review was conducted according to PRISMA guidelines. We searched OVID Medline between January 1977 and January 2024 for studies describing CD uterine closure techniques with subsequent PAS pregnancy outcomes. Preference given to randomized controlled trials (RCTs) and case‐control studies. Studies were excluded if only short‐term outcomes after the CD such as niche formation or residual myometrial thickness (RMT) were reported, but not PAS; and if no controls.

**Results**: Of 3,529 studies retrieved, in the two studies evaluating single‐ vs double‐layer closure, no significant differences were noted, possibly confounded by the inclusion of the endometrium in closure (OR 0.61, CI 0.3‐1.17). In one small study, there were no PAS cases in either prior purse‐string vs double‐layer uterine closure (table). In 1 study, an interrupted uterine closure technique was associated with significantly decreased subsequent PAS, compared to continuous (OR 0.31, 0.13‐0.74). No studies were found on inclusion or exclusion of endometrium, or type of suture, at uterine closure. Studies which found significant findings in their short‐term ultrasonographic outcomes did not have a clear clinical correlation in their long‐term outcomes.

**Conclusion**: There are few studies looking at PAS as an outcome of prior CD surgical technique. In 1 study, interrupted uterine closure was found to be associated with a lower risk of PAS at subsequent pregnancy. Single‐layer, endometrium‐free, and purse‐string techniques are all promising methods for additional study. More long‐term follow‐up data beyond niche and RMT is needed to assess the relationship between different uterine closure techniques and PAS. Optimizing surgical techniques may help prevent PAS.

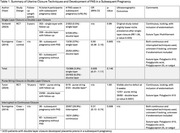

## 416 | Preconceptional bariatric surgery in women and fetal growth restriction: The rotterdam periconception cohort

Katinka Snoek^1^, Sten Willemsen^1^, Joop S.E. Laven^2^, Regine Steegers‐Theunissen^1^, Sam Schoenmakers^3^

^1^Erasmus University Medical Center, Erasmus University Medical Center, Zuid‐Holland; ^2^Erasmus University Medical Center, Zuid‐Holland; ^3^Erasmus University Medical Centre, DEN HAAG

16:45–18:00

**Objective**: To study the impact of preconception bariatric surgery on embryonic and fetal growth trajectories.

**Study Design**: A prospective cohort study of women who have undergone bariatric surgery (cases). The study is designed as a subcohort of cases within the Rotterdam Periconception cohort (the Predict Study). For analysis a case cohort design has been used.

Growth trajectories of crown‐rump length (CRL) and embryonic volume (EV) at 7, 9 and 11 weeks of gestation, fetal growth parameters (head and abdominal circumference, biparietal diameter, femur length and estimated fetal weight) during the 2^nd^ and 3^rd^ trimester and birth weight were investigated.

Embryonic and fetal outcomes were compared with women without bariatric surgery (controls) from the Predict study, a tertiary prospective cohort in Rotterdam, the Netherlands.

**Results**: Sixty‐five cases of pregnancies after bariatric surgery were included, and 1493 pregnancies without bariatric surgery.

The majority (70.8%) of the 65 post‐bariatric patients had undergone gastric bypass surgery, 27.7% had undergone a gastric sleeve and 1.5% had undergone gastric banding. Median time between bariatric surgery and conception was 39.6 months (IQR 25.9‐64.7).

After adjustment for covariates, bariatric surgery was not associated with CRL and EV trajectories in the first trimester.

Growth velocity of estimated fetal weight and abdominal circumference as compared with controls was decreased from 20 weeks of gestation. This resulted in significant reduced growth trajectories of EFW at 26^+0^ weeks (ß −0.031, 95% CI −0.061–‐0.002, *p* = 0.037) and 32^+0^ weeks (ß −0.053, 95% CI −0.088–‐0.017, *p* = 0.003). Abdominal circumference was also significantly lower at 26^+0^ weeks (ß −0.014, 95% CI −0.027–‐0.002, *p* = 0.026) and 32^+0^ weeks (ß −0.024, 95% CI–0.040–‐0.009, *p* = 0.002).

Birth weight was significantly lower after bariatric surgery (‐209,3 grams, 95% CI −348,5–‐70.2, *p* = 0.003).

**Conclusion**: Bariatric surgery is not associated with embryonic growth, but with fetal growth restriction from 20 weeks of gestation onwards.

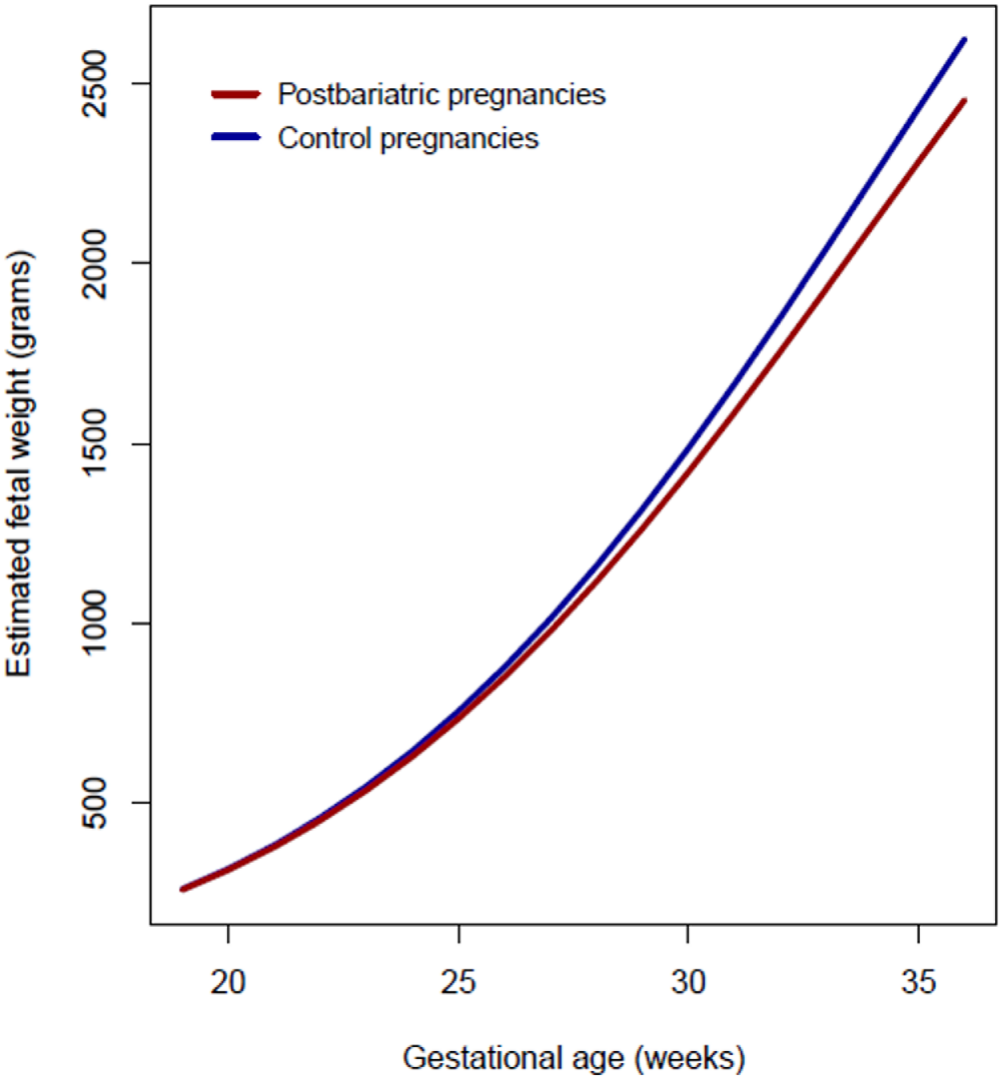

## 417 | Segregated placental immune profiles in SARS‐CoV‐2‐placentitis & chronic histiocytic intervillositis and normal profiles after mRNA‐COVID19‐vaccination

Michelle Broekhuizen^1^, Marie Louise van der Hoorn^2^, Disha Vadgama^1^, Michael Eikmans^2^, Johannes Duvekot^1^, Pieter Fraaij^1^, Irwin Reiss^1^, Dana Mustafa^3^, Lotte van der Meeren^1^, Sam Schoenmakers^4^

^1^Erasmus University Medical Center, Erasmus University Medical Center, Zuid‐Holland; ^2^Leiden UMC, Leiden UMC, Zuid‐Holland; ^3^Erasmus University Medical Center, er, Zuid‐Holland; ^4^Erasmus University Medical Centre, DEN HAAG

16:45–18:00

**Objective**: Severe acute respiratory syndrome coronavirus 2 (SARS‐CoV‐2) infection in the placenta can lead to fetal distress and demise by inducing SARS‐CoV‐2 placentitis, characterized by severe trophoblast necrosis, chronic histiocytic intervillositis, and perivillous fibrin deposition interfering with maternal‐fetal exchange. SARS‐CoV‐2 mRNA vaccines prevent adverse COVID‐19 effects, but their safety during pregnancy and placental physiology has raised concerns. We aim to gain insight into placental immune alterations to understand mechanisms leading to impaired placental function.

**Study Design**: Placentas were retrospectively collected from cases with SARS‐CoV‐2 placentitis and fetal distress (*n* = 9), with CHI (*n* = 9), after mRNA COVID‐19 vaccination during pregnancy (*n* = 9) and from uncomplicated term controls (*n* = 9). The expression of 53 immune‐related proteins was quantified using GeoMx® Digital Spatial Profiler in three separate compartments: villi (fetal compartment), intervillous space, and decidua (both maternal compartments).

**Results**: SARS‐CoV‐2 placentitis displayed alterations in the expression of 18 proteins in the intervillous space only, including upregulation of myeloid markers (e.g. CD40, CD68) and downregulation of apoptotic proteins (e.g. BAD, BCL6). The intervillous space of placentas with CHI showed similar changes in myeloid proteins, but not in apoptotic proteins. Importantly, placentas after mRNA COVID‐19 vaccination showed no significant differences in immune protein expression compared to controls. The decidua and villi displayed no immune alterations in any group.

**Conclusion**: SARS‐CoV‐2 placentitis and CHI are associated with distinct enhanced immune alterations in the intervillous space. The reduced apoptotic protein expression in SARS‐CoV‐2 placentitis could relate to the widespread placental destruction hampering its function. During SARS‐CoV‐2 infection, the placental barrier against viral pathogens remains intact, while the local immune response has adverse effects on the fetus. The absence of placental immune alterations after mRNA COVID19 vaccines indicate reassurance about its safety.

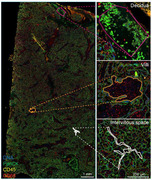

## 418 | Unique insights into the genetics of the Puerto Rican population through reproductive carrier screening

Camila Rivera‐Lynch^1^, Madeleine Armer‐Cabral^2^, Emily Becraft^2^, Katherine Howard^2^, Yang Wang^2^, Vivienne Souter^2^, Alberto De La Vega^3^

^1^University of Puerto Rico, University of Puerto Rico Medical Sciences Campus, PR; ^2^Natera, Inc., San Carlos, California; ^3^University of Puerto Rico Medical Sciences Campus, University of Puerto Rico Medical Sciences Campus, Puerto Rico

16:45–18:00

**Objective**: Offering carrier screening to people who are pregnant or considering pregnancy is standard of care in the US. The Hispanic population of Puerto Rico has a diverse racial and ethnic background that might differ from the overall US Hispanic population. However, there are limited data on carrier status for genetic conditions in Puerto Rico. We report carrier frequency for a 14‐gene reproductive carrier screening (CS) panel in a cohort of pregnant people on the island of Puerto Rico.

**Study Design**: This retrospective study included samples from Puerto Rico submitted to a commercial laboratory for a 14‐gene CS panel (Jan 2022–Dec 2023). The study was restricted to pregnant people, aged 18 to 55 years. Data on patient characteristics were collected from the test requisition form. Positive carrier screening results were defined as any pathogenic or likely pathogenic (P/LP) variant (see Table 1 footnote).

**Results**: Of 10,845 samples evaluated, race/ethnicity was available for 80.7%, and 79.0% were reported to be of Hispanic ancestry. Approximately 21.1% were carriers for one, 3.3% for two, and 0.2% for three conditions. The genes, their associated conditions and observed carrier frequencies are shown in Table 1. Carrier frequency was higher than expected for medium‐chain acyl‐CoA dehydrogenase deficiency (MCAD) compared to the US population and for cystic fibrosis compared to reports in the US Hispanic population. 1 in 47 were carriers for spinal muscular atrophy.

**Conclusion**: Results from this 14‐gene CS panel revealed some unexpected results in the Puerto Rican pregnant population. The relatively high carrier frequency for SMA is important as SMA is not part of newborn screening (NBS) in Puerto Rico. The higher than expected carrier frequency for MCAD is also notable as this condition may manifest in neonates due to poor feeding before NBS results become available. This study highlights the need for greater understanding of the genetics of the Puerto Rican population.

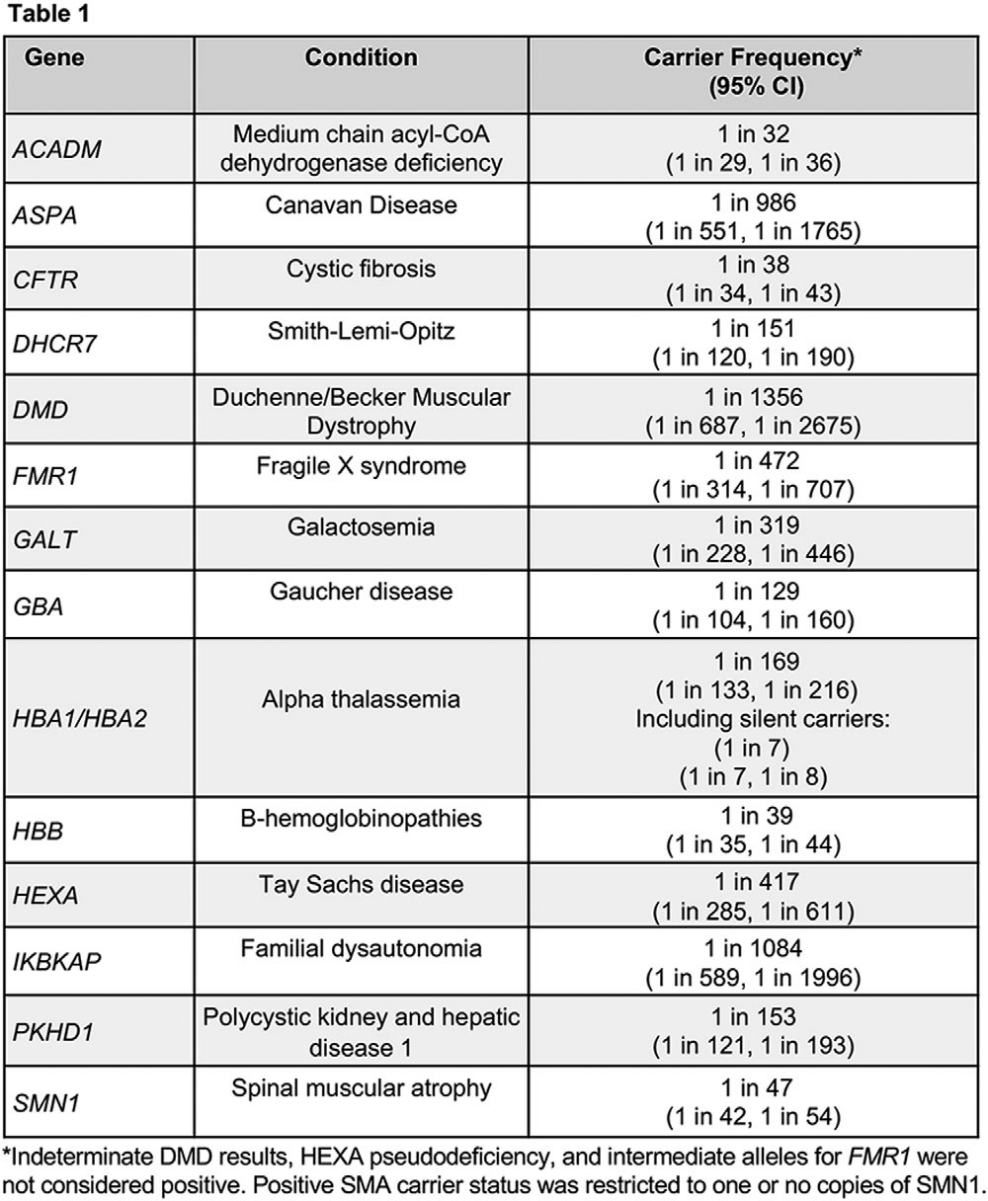

## 419 | First trimester preeclampsia prediction in twins by fetal fraction of cell free DNA and PlGF

Adi Sharabi‐Nov^1^, Ran Svirsky^2^, Ron Maymon^3^, Richard Brown^4^, Heidy Portillo Rodriguez^5^, Linda Peltier^6^, Kypros NICOLAIDES^7^, Hamutal Meiri^8^

^1^Tel Hai Academic Collage, ZIV medical center, HaZafon; ^2^Ben‐Gurion University, School of Medicine, HaDarom; ^3^Department of Obstetrics and Gynecology, Shamir (Assaf Harofeh) Medical Center, Zerifin, Tel Aviv; ^4^McGill University, Montreal, Quebec; ^5^Division of Maternal Fetal Medicine, Department of Obstetrics & Gynaecology, McGill University, McGill University Health Centre, Montreal, Quebec; ^6^McGill University, Cellular Therapy Laboratory, Quebec; ^7^The Fetal Medicine Research Institute, King's College Hospital, London, England; ^8^Department of Obstetrics and Gynecology, Shamir (Assaf Harofeh) Medical Center, TeleMarpe, Tel Aviv

16:45–18:00

**Objective**: To assess first trimester prediction of preeclampsia (PE) in twin pregnancy by fetal fraction of circulating cell‐free DNA (ccfDNA) using non‐invasive prenatal screening (NIPS) alone and combined with placental growth factors (PLGF), mean arterial blood pressure (MAP), and uterine arteries pulsatility index (UtA‐PI).

**Study Design**: NIPS was conducted in pregnant women with mono‐and‐ di‐chorionic twins who enrolled at gestational weeks 11‐13 at Zerifin (Israel) and Montreal (Canada). The fetal fraction of ccfDNA was determined by NIPS using whole genome sequencing. PLGF was measured by immunodiagnostics of serum samples. MAP was calculated from systolic and diastolic blood pressure, and UtA‐PI was determined by Doppler sonography. Women were followed every 4 weeks till labor, and their delivery records were recorded. Medians and interquartile range (IQR) were determined for continuous parameters. Uni‐and‐multi‐variate prediction accuracy was estimated from the area under the receiver operating characteristic curve (AUC of ROC). Data was analyzed by non‐parametric Kruskal Wallis and Mann–Whitney tests. *P* < 0.05 was considered significant.

**Results**: There were 20 cases of preterm PE (delivery at 35.9 weeks (IQR:34.4‐36.6), 7 cases of HELLP (delivery at 35.7 (IOR: 35.1‐36.7), and 165 cases without hypertensive complications (mean delivery 36.9 weeks (IQR: 35.0‐37.4). The median fetal fraction was reduced in the preterm PE cases to 9.0% (IQR: 8.4‐10.3) compared to HELLP–12% (9%‐17%) (*p* > 0.05), and to 14% (11%‐16%) in non‐PE cases (*p* < 0.001). AUC = 0.73 (95% CI: 0.61‐0.85) for the reduced fetal fraction, AUC = 0.71 (95% CI: 0.59‐0.83) for reduced PLGF, AUC = 0.61 (95% CI 0.50‐0.72) for the slightly higher MAP, but AUC = 0.54 (95% CI: 0.39‐0.69) for the elevated UtA‐PI. Combining fetal fraction, PLGF, and MAP yielded AUC = 0.89 (95% CI: 0.78‐0.98), sensitivity of 81% at 90% specificity. NPV = 50% and PPV = 97.5%. UtA‐PI didn't add to the combined prediction of preterm PE.

**Conclusion**: Effective prediction of PE in twin pregnancy appeared promising by combining reduced fetal fraction of ccfDNA with serum PLGF and MAP.

## 420 | Uterine artery pulsatility index and TNFΑ as dual markers for early PTD and late FGR

Adi Sharabi‐Nov^1^, Vesna VODUŠEK^2^, Tanja Premru SRŠEN^2^, Kristina KUMER^2^, Teja FABJAN^3^, Nataša TUL^4^, Josko OSREDKAR^5^, Kypros NICOLAIDES^6^, Berthold HUPPERTZ^7^, Hamutal Meiri^8^

^1^Tel Hai Academic Collage, ZIV medical center, HaZafon; ^2^University Medical Center, Ljubljana, Ljubljana; ^3^University Medical Center, University Medical Center, Ljubljana; ^4^Faculty of Medicine, Faculty of Medicine, Ljubljana; ^5^University of Ljublijana, University of Ljublijana, Ljubljana; ^6^The Fetal Medicine Research Institute, King's College Hospital, London, England; ^7^Medical University of Graz, Graz, Steiermark; ^8^Department of Obstetrics and Gynecology, Shamir (Assaf Harofeh) Medical Center, TeleMarpe, Tel Aviv

16:45–18:00

**Objective**: Evaluate tumor necrosis factor alpha (TNFα) and uterine artery pulsatility index (UtA‐PI) in the triage patients with suspected preterm delivery (PTD), preeclampsia (PE), fetal growth restriction (FGR), and PE+FGR

**Study Design**: A secondary analysis of a database of 125 pregnant women attending high‐risk pregnancy clinics for triage of pregnancy complications included cases of PTD, PE, FGR and FGR combined with PE at 24–39 weeks gestation and term controls with no pregnancy complications. Maternal serum TNFα was determined by immune‐diagnostic testing. UtA‐PI was measured by Doppler sonography using trans‐abdominal ultrasound. Demographic, medical and pregnancy history, and mean arterial blood pressure (MAP) were extracted from the hospital medical records. Linear regression and Box‐plots were calculated and depicted using Kruskal Wallis and Mann–Whitney tests. Spearman's regression coefficient was used to assess marker accuracy; *p* < 0.05 was considered significant.

**Results**: In early cases of PTD ( < 34 weeks gestation), serum TNFα levels were significantly higher while UtA‐PI and MAP were not different from term controls. At 34‐37 weeks gestation, TNFα decreased in PTD cases. In cases with late FGR TNFα was low at midst gestation and continuously increased towards delivery >37 wks gestation while UtA‐PI was high throughout the period. Cases who also have high MAP (FGR+PE) followed same pattern as pure FGR with high UtA‐PI but the increase of their TNFα was more moderate compared to pure FGR cases. No correlations were found between serum TNFα and maternal BMI, MAP, or any of the drugs administrated to treat any of the complications.

**Conclusion**: High TNFα < 34 at low UtA‐PI and without hypertension, reflects early PTD linked to maternal inflammation. For FGR and PE+FGR (divided by hypertension) TNFα is low < 34 and is high >37 weeks gestation reflecting inflammation, and maternal and fetal hypoxia.

## 421 | Intrauterine exposure to COVID‐19 infection by trimester and risk for congenital hearing loss: A meta‐analysis

Gabriel Ghanadan^1^, Eric Shawkey^2^

^1^Georgetown University School of Medicine, Washington DC, DC; ^2^Georgetown University School of Medicine, Washington DC, District of Columbia

16:45–18:00

**Objective**: Viral infections during pregnancy are the most common non‐genetic cause of congenital hearing loss. The objective of this study is to evaluate the risk of developing hearing loss in babies born to mothers that tested positive to COVID‐19 at varying times during pregnancy.

**Study Design**: A meta‐analysis was performed of retrospective cohort studies published up to April 2024. Primary articles containing data on COVID‐19 infections during pregnancy and neonatal hearing screening (NHS) results were found using the online MEDLINE database. A total of 16 studies were reviewed regarding time of COVID‐19 infection categorized by trimester. Eight studies met inclusion criteria, and the results for 3,843 pregnant individuals were compared.

**Results**: Of the 1,799 women who reported having COVID‐19 during pregnancy, 123 (6.8%) contracted in the first trimester, 449 (25.0%) in the second, and 1227 (68.2%) in the third. The number of neonates who failed their first NHS were 20 (16.3%), 101 (22.5%), and 204 (16.6%) in each trimester group respectively. In the control group, 359 of 2,044 (17.6%) neonates failed their first NHS. Relative risk for failing the NHS was 0.93 (*p* = 0.36), 1.28 (*p* < 0.01), and 0.95 (*p* = 0.64) for neonates in the first, second, and third trimester group respectively compared to controls. There was no significant difference in risk for failing NHS when comparing neonates from first and second trimester groups (RR = 0.72, *p* = 0.08), however there was when comparing second and third trimester groups (RR = 1.35, *p* < 0.01).

**Conclusion**: Neonates born to pregnant women who contracted COVID‐19 in their second trimester were at a significantly increased risk of failing their first NHS compared to those born to women who did not contract COVID‐19 in pregnancy. Neonates in the second trimester group were also at an increased risk compared to those in the third trimester group. These findings suggest that timing of COVID‐19 exposure may impact hearing development in‐utero. Further research should be directed to understand the relationship of second trimester COVID‐19 exposure and auditory development.

## 422 | Maternal‐to‐child‐transmission of human immunodeficiency virus in pre‐term premature rupture of the membranes: A systematic review

Sara Ismail^1^, Alexa Del Corpo^2^, Andrew Zakhari^3^, Benjamin Jacob^4^, Karen Wou^3^

^1^McGill University, Lachine, QC; ^2^University of Limerick, Limerick, Limerick; ^3^McGill University, Montreal, Quebec; ^4^Royal College of Surgeons in Ireland, Dublin, Dublin

16:45–18:00

**Objective**: Pre‐term Premature Rupture of Membranes (PPROM) in the context of maternal human immunodeficiency (HIV) infection poses a significant management dilemma, as the clinician must balance the risks of prematurity with the risks of delaying delivery and potentially increasing the chance of mother‐to‐child transmission (MTCT) of HIV. The objective of this study is to identify the rate of MTCT of HIV in HIV‐positive women with PPROM and neonatal outcomes.

**Study Design**: A systematic search strategy was developed by an information specialist, and applied to the following online databases from inception to 2023: Ovid MEDLINE, Ovid EMBASE, PubMed (non‐MEDLINE), Web of Science, CENTRAL, WHO and International Clinical Trials Registry. Prospective and retrospective cohort studies of HIV positive patients with pre‐term premature rupture of membranes in which rates of MTCT were reported were included. Case reports and case series were eligible for inclusion.

**Results**: Initial screening of 312 articles resulted in the inclusion of three studies from the USA and one from South Africa. The crude MTCT rate of HIV across the included 58 pregnancies was 6.9% (*n* = 4). Unadjusted logistic regression of two studies revealed no association between MTCT of HIV and antiretroviral use, CD4 count, gestational age, and length of PPROM. There was however a trend towards higher risk of MTCT with an elevated viral load (*p* = 0.09) or spontaneous vaginal delivery (*p* = 0.06).

**Conclusion**: This review found a crude MTCT rate of HIV in women with PPROM of approximately 6.9%, albeit in a heterogeneous population with variable antiretroviral use. More recent data with contemporary management of HIV is lacking to better inform counselling and decision making in this challenging clinical setting.

## 423 | Predicting neonatal infection in PPROM using vaginal microbiology and metagenomics: A prospective cohort study

Laurent Mandelbrot^1^, Sean Kennedy^2^, Jessica Rousseau Rousseau^3^, Francois Goffinet^4^, Luce Landraud^3^, Céline Plainvert^3^, Pierre‐Henri Jarreau^5^, Luc Desfrère^6^, Lahçene Allal^5^, Nathalie Grall^3^, Pierre‐Yves Ancel^3^, Claire Poyart^5^, Asmaa Tazi^3^

^1^Hôpital Louis Mourier, APHP, Université Paris Cité, Colombes, Ile‐de‐France; ^2^Institut Pasteur, Paris, Ile‐de‐France; ^3^Assistance Publique Hôpitaux de Paris, Paris, Ile‐de‐France; ^4^Université Paris Cité, Inserm, Centre for Research in Epidemiology and StatisticS (CRESS), Obstetrical Perinatal and Pediatric Epidemiology Research Team (EPOPé), Paris, Ile‐de‐France; ^5^Assistance Publique Hopitaux de Paris, Paris, Ile‐de‐France; ^6^Assistance Publique Hopitaux de Paris, Colombes, Ile‐de‐France

16:45–18:00

**Objective**: To determine whether culture and metagenomic analysis of vaginal swab samples are predictive of the risk of early‐onset neonatal sepsis (EONS) in PPROM.

**Study Design**: In a prospective 3‐center cohort, patients with PPROM were enrolled between 22 and 36 WG + 6 days. Vaginal swab samples were taken at presentation and delivery, with routine antibiotics, obstetrical and neonatal care. We defined an algorithm interpreting vaginal sample cultures according to the flora and presence and quantification of bacteria with known pathogenic potential. Whole‐genome metagenomic sequencing analysis was performed.

**Results**: 563 PPROM cases were enrolled, with 651 neonates, 99% liveborn. PPROM was < 32 WG in 41% and deliveries were < 34 WG in 46%. Fever in labor occurred in 10%. 88% of mothers received antibiotics (ampicillin or macrolides 74%, cephalospirin or combination therapy 26%) at delivery.

The incidence of EONS was 29/651 (4.4%). The main pathogens in neonates were E coli (48.3%) and other Gram‐negatives 17.2%; there was one case with Group B streptococcus (GBS) (3.4%).

Pathogenic vaginal cultures according to the algorithm were associated with EONS in 14/147 (9.52%), vs 7/205 (3.41%) without pathogenic cultures, a more than 3‐fold increased risk, persisting after adjustment for latency period and gestational age. PPV and NPV of bacterial culture to predict EONS were 9.5 % and 96.6%, respectively. The pathogens identified from vaginal sample cultures were E coli (41%), other Gram‐negatives (17%), GBS (17%), and S aureus (10%), other or none (15%). Metagenomic signatures (*n* = 306) were highly associated with EONS (*p* < .001).

**Conclusion**: In PPROM, conventional microbial culture of maternal vaginal samples was associated with EONS, but its predictive values are insufficient to guide perinatal care. Metagenomic microbial signatures have the potential to improve the predictive values relatively to conventional culture. This opens the perspective for a rapid point‐of‐care test, also incorporating inflammation biomarkers.

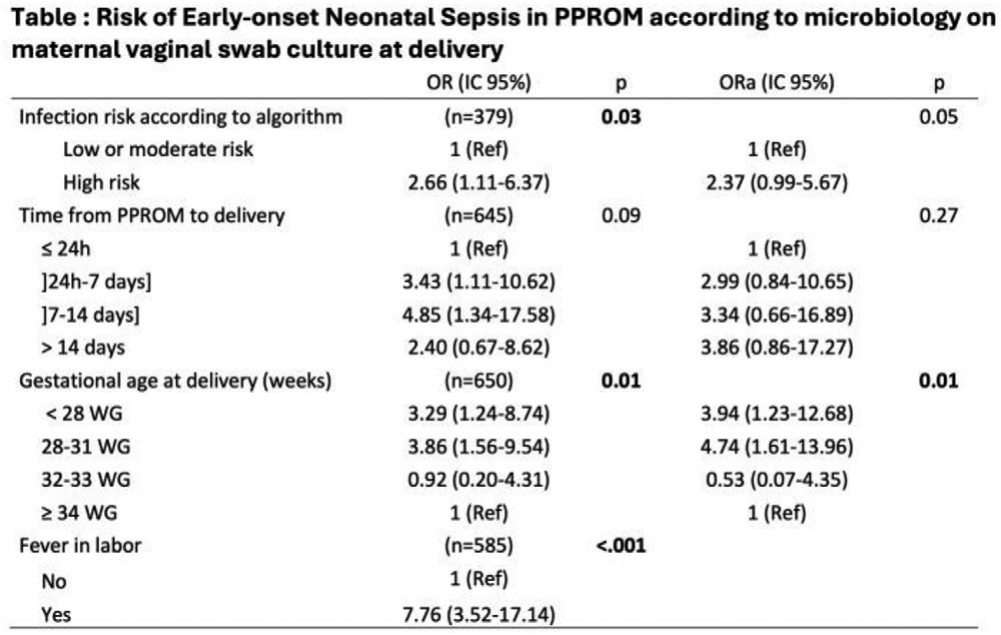

## 424 | Determining the knowledge levels and self‐confidence of fourth year midwifery students about graduation about nonstress‐test

Yasemin Sökmen^1^, Zeliha Koç^1^

^1^Ondokuz Mayıs University, Ondokuz Mayıs University, Samsun

16:45–18:00

**Objective**: The aim of this study is to determine the knowledge levels and self‐confidence of fourth‐year midwifery students who are at the graduation stage regarding nonstress testing.

**Study Design**: In this study, descriptive phenomenological design, one of the qualitative research methods, was used. The study was conducted with 15 fourth‐year students studying in the midwifery department of a state university between March and April 2024. Midwifery students' nonstress test knowledge levels and self‐confidence were examined using the individual in‐depth interview technique. Thematic analysis method was used when analyzing the data. As a result of the analysis, four main themes were determined: (I) fetal health, (II) nonstress test knowledge level, (III) self‐confidence and (IV) education need. The results obtained were reported in accordance with the COREQ criteria.

**Results**: Midwifery students know the tests used to evaluate fetal health in the antenatal period; their level of knowledge about nonstress testing and their self‐confidence in evaluating nonstress test results were not sufficient; it was determined that they needed to repeat their lecture notes and attend training or courses in order to increase their knowledge and self‐confidence regarding the nonstress test.

**Conclusion**: It was determined that the knowledge level and self‐confidence of fourth‐year midwifery students, who are at the graduation stage, regarding the nonstress test were insufficient.

## 425 | The role of fetal head station during rupture of membranes; a retrospective large cohort study

Yishai Sompolinsky^1^, Joshua Guedalia^2^, Naama Vilk‐Ayalon^3^, Sarah Cohen^4^, Shirley Greenbaum^1^, Doron Kabiri^5^, Simcha Yagel^6^, Michal Lipschuetz^7^

^1^HADASSAH HEBREW UNIVERSITY MEDICAL CENTER, Jerusalem, Yerushalayim; ^2^Hadassah medical center, jerusalem, Yerushalayim; ^3^Hadassah Hebrew University Medical Center, +972 52‐688‐8140, Yerushalayim; ^4^Washington University in St. Louis, St. Louis, Missouri; ^5^Department of Obstetrics and Gynecology, Hadassah‐Hebrew University Medical Center, Jerusalem, Israel., Jerusalem, Yerushalayim; ^6^Hadassah Medical Center, Faculty of Medicine, Hebrew University, Yerushalayim, Yerushalayim; ^7^Henrietta Szold Hadassah Hebrew University School of Nursing in the Faculty of Medicine, Henrietta Szold Hadassah Hebrew University School of Nursing in the Faculty of Medicine, Yerushalayim

16:45–18:00

**Objective**: The aim of this study was to investigate the association between fetal head station (FHS) during artificial rupture of membranes (AROM) and time to delivery and other obstetrical outcomes

**Study Design**: A retrospective cohort study encompassing data from labors during a 12‐year period were analyzed. All cases of singleton, term pregnancy with documented AROM time were included. The study population was stratified by parity.

**Results**: This study cohort included 45,898 singleton, term vaginal delivery parturients with time stamp at time of AROM and delivery; Stratification by parity yielded 11,947 primiparas (26%) and 33,951 multiparas (74%). Across all sub‐cohorts, as FHS decreased at AROM the duration from ROM to delivery was shorter. This trend seems to be stronger for multiparas (Figure 2) than primiparas (Figure 1). Cesarean delivery, postpartum hemorrhage, neonatal intensive care unit admission and low 5‐minutes Apgar scores were also negatively associated with decrease in FHS at AROM across all cervical dilations.

**Conclusion**: The study findings highlight the crucial role of considering FHS alongside cervical dilation during AROM, as it significantly impacts time to delivery and various obstetrical outcomes. This underscores the necessity for personalized timing of AROM, especially in multiparous women, to enhance maternal and neonatal health outcomes.

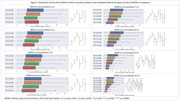

## 427 | Variation in use of MFM services among commercially insured people: The other maternity care desert

Haley K. Sullivan^1^, Joanne C. Armstrong^2^, Kathe P. Fox^3^, Jessica L. Cohen^4^, Anna D. Sinaiko^5^

^1^Harvard Health Policy PhD Program, Boston, MA; ^2^CVS Health, Houston, Texas; ^3^Health Care Cost Institute, Verona, New Jersey; ^4^Harvard T.H. Chan School of Public Health, Boston, Massachusetts; ^5^Harvard School of Public Health, Boston, Massachusetts

16:45–18:00

**Objective**: Improving access to high‐quality maternity care and reducing maternal morbidity and mortality are major policy priorities in the United States. Previous research has primarily focused on access to general obstetric care (e.g., maternity care deserts) and has not explored access to high‐risk pregnancy care provided by maternal fetal medicine subspecialists (MFMs). We aim to measure access to MFM services and determine patient factors predictive of MFM service use, including MFM telemedicine, in the US.

**Study Design**: We identified a cohort of 2.1 million pregnancies in commercial health insurance claims from the Health Care Cost Institute from 2016‐2021. Primary study outcomes included whether a pregnancy ever had a service provided by an MFM (termed “MFM involvement in care”), the type of MFM services provided, and whether MFM care occurred via telemedicine. We analyzed the association of patient and pregnancy covariates with MFM involvement in care using logistic regression and reported rates of telemedicine use by urban vs. rural status over time.

**Results**: Among pregnancies with maternal or fetal conditions that might require MFM involvement (“at‐risk pregnancies”), 51.5% received care from an MFM. Rates of MFM involvement in care varied considerably by geography, with rural areas having lower use than urban areas. Use of telemedicine MFM care increased in 2020 and 2021 with the COVID‐19 pandemic but remained low. In 2021, only 2.7% of urban and 1.7% of rural pregnancies received MFM services via telemedicine.

**Conclusion**: This paper contributes a new dimension to the existing concept of maternity care deserts and demonstrates wide variation in MFM involvement in pregnancy care throughout the US that is particularly acute in rural areas. Telemedicine is a potential solution to close these geographic gaps in access, although current usage is low. As policymakers focus attention on improving the maternity care crisis in the US, access to perinatal specialist care from MFMs should inform policy and programs.

## 429 | Severe Acute Maternal Morbidity (SAMM)–An indicator of quality and complexity in obstetrics

Julia Unterscheider^1^

^1^Royal Women's Hospital, Royal Women's Hospital, Victoria

16:45–18:00

**Objective**: The audit of SAMM outcomes, defined as an admission to an adult intensive care unit (ICU) during pregnancy, childbirth or within 42 days of birth or termination of pregnancy, acts as a quality and complexity indicator of obstetric care.

**Study Design**: All instances of SAMM between January 1, 2023 and December 31, 2023 at the Women's, Australia's largest single‐site maternity hospital, were classified with a focus on accurately identifying the underlying cause or causes of morbidity, associated conditions, contributing factors and where required, the identification of opportunities for improving perinatal practice and service provision.

**Results**: In 2023, there were 22 SAMM cases among 6,808 women who gave birth at the Women's, accounting for 1 in 310 women being admitted to the ICU at the co‐located general hospital. An additional 32 women were admitted directly to the co‐located ICU. The number of ICU admissions on campus has steadily increased from 40 cases in 2020 to 54 cases in 2023.

The majority of women (95%; *n* = 21) were admitted to ICU in the immediate postpartum period. Eight women were affected by one morbidity (42%), 3 by two morbidities (16%) and 8 by three or more morbidities (42%). The primary causes of morbidity were major obstetric haemorrhage (*n* = 8; 42%) with a mean blood loss of 7,668 mls (range 4,000‐14,000 mls), pre‐eclampsia related liver and renal dysfunction (*n* = 6; 32%) and peripartum hysterectomy (*n* = 5; 26%).

Of the 32 admissions to the co‐located ICU, 21 women (66%) were admitted during pregnancy at an average gestational age of 25.6 weeks (range 5.0‐37.4 weeks): 3 women were in their first trimester, 7 in their second trimester, 11 were in the third trimester; a further 11 women (34%) were postpartum. The most common indication for ICU admission was septicaemic shock (*n* = 7).

Figure 1 outlines the overall morbidity as a total count of each classification for both sites.

**Conclusion**: A concise and detailed review of SAMM is an important step towards promoting reflective practice and safe pregnancy care.

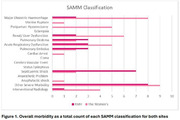

## 430 | Shoulder dystocia: Contemporary management and outcome

Pamela Gebrehiwot^1^, Julia Unterscheider^2^

^1^The Royal Women's Hospital, Parkville, Victoria; ^2^Royal Women's Hospital, Royal Women's Hospital, Victoria

16:45–18:00

**Objective**: Shoulder dystocia is an obstetric emergency with significant impact on maternal and neonatal morbidity. The objective of our study was to provide contemporary data on the incidence, management and outcome of shoulder dystocia.

**Study Design**: This retrospective cohort study was conducted at the Women's, Australia's largest single‐site quaternary maternity hospital. Women who had a vaginal birth complicated by shoulder dystocia between January 1, 2023 and December 31, 2023 were included in this analysis. Maternal demographics and risk factors, number and type of maneuvers, and neonatal outcome data was obtained through review of individual patient records from our Electronic Medical Records System (Epic Systems Corporation).

**Results**: In 2023, shoulder dystocia complicated 129 vaginal births with an overall incidence of 3.38%. One‐hundred and twenty‐eight babies were liveborn; there was one pre‐labour FDIU at 37 weeks.

The mean maternal age was 32.4 years, and the mean BMI was 24.5 kg/m^2^; Labor induction occurred in 52%. Birth was assisted in 48% including 34 forceps and 26 vacuum births. Sixty‐four percent of women had an episiotomy and 10% had an obstetric anal sphincter injury.

The most commonly used maneuvers were McRoberts in 118 (92%), and suprapubic pressure in 51 (42%) births, respectively. Additional maneuvers were needed in 42 births, with delivery of the posterior arm associated with the highest success. Posterior axillar sling traction was used in one instance.

The mean gestational age at birth was 39.6 weeks. The mean birthweight was 3806 grams. Forty‐three infants (34%) of babies required resuscitation at birth, and 14 (11%) were admitted to the neonatal unit. Of the 14 admitted infants, 4 infants had sequelae from birth including 2 cases of moderate HIE requiring therapeutic hypothermia, 3 humeral fractures with 1 resulting in a brachial plexus injury.

**Conclusion**: Shoulder dystocia is a common obstetric emergency. While McRoberts resolves the majority of shoulder dystocias, familiarity with additional maneuvers including release of the posterior arm and axillar sling traction is essential.

## 432 | Cervical length above cerclage, relative cerclage position and subsequent preterm birth risk

Laura CB. van der Krogt^1^, Andrew Shennan^2^, Natalie Suff^3^

^1^King's College London, London, England; ^2^King's College London, King's College London, England; ^3^Kings College London, London, England

16:45–18:00

**Objective**: A transvaginal cerclage placed high in the cervix is associated with cerclage success. The significance of the cervical length above the cerclage following insertion has not been fully elucidated. However, a shorter length of cervix measured above the cerclage could reflect active cervical change. The relative position of the cerclage (the length above the cerclage as a proportion of the total cervical length) was investigated to determine if this was associated with cerclage failure and preterm birth.

**Study Design**: This was a retrospective cohort study of women who received a transvaginal cerclage in a singleton pregnancy. Women who had a repeat cerclage or iatrogenic preterm delivery were excluded. Demographic data, cervical length before and after cerclage insertion, length above the cerclage (measured as distance from cerclage to internal os), and gestation at delivery were collected. The relative position of the cerclage was calculated as the length above the cerclage as a proportion of the total cervical length following the procedure. The difference in this proportion between those who delivered preterm and term was assessed.

**Results**: Of the 53 women included, 49%(*n* = 26) had a history‐indicated cerclage, 45%(*n* = 24) had an ultrasound indicated cerclage and 6%(*n* = 3) had an emergency rescue cerclage. 25%(*n* = 13) delivered preterm and 75%(*n* = 40) delivered term. There was a significant difference in the relative position of the cerclage; the mean relative position was 0.41 in those who delivered preterm compared to 0.52 in those who delivered term. The difference between the means was 0.11(95% confidence interval 0.008 to 0.22; p value 0.03).

**Conclusion**: The relative position of a transvaginal cerclage is a risk factor for cerclage failure and may suggest active cervical remodelling above the level of the cerclage. Although a cerclage placed high in the cervix is known to be associated with better outcomes, a shortening cervix above the level of the cerclage may be a poor prognostic sign. This was a small retrospective study, and larger studies are required to assess relative cerclage position further.

## 433 | Antenatal corticosteroids for cervical insufficiency: Can we improve timing?

Lennart Van der Veeken^1^, Diya Ahmad^2^, Simonne Holubeshen^2^, Kellie Murphy^2^, John W. Snelgrove^2^, John W. Snelgrove^2^

^1^Fetal Medicine Unit, Department of Obstetrics and Gynaecology, Mount Sinai Hospital and University of Toronto, Toronto, Ontario; ^2^University of Toronto, Toronto, Ontario

16:45–18:00

**Objective**: A course of antenatal corticosteroids (ACS) improves the outcome for children born before 34 weeks. One of the most important aspects is the timing of administration. We investigated the rate of optimally administered ACS for different indications, assess the evolution in the past 10 years and examine improved strategies for ACS timing.

**Study Design**: This is a retrospective cohort study including all pregnant patients treated with ACS (betamethasone or dexamethasone) at Mount Sinai Hospital from 2014 to 2023. Cases lost‐to‐follow‐u*p* < 14 days after administration were excluded. Patient characteristics and birth outcomes were collected. Administered ACS were categorized by interval‐to‐birth: < 2d, 2‐7days, 7‐14days and >14days. Timing accuracy was determined for each indication and changes in accuracy were examined.

**Results**: We reviewed 4578 patients treated with ACS. The most frequent indications were threatened preterm labor (24%), PPROM (19%) and cervical insufficiency (16%). Overall, 33% ACS were given within 7 days of delivery and 44% within 14 days. This number has remained stable over the past 10 years. For fetal indications and cervical insufficiency (23% of all patients), only 16% and 15% respectively were treated within 14 days of delivery. For cervical insufficiency, if ACS had been given ACS only to those with an open cervix, 50% of patients would have had an ACS‐to‐birth interval < 14days. For patients with a short but closed cervix, if ACS were had been given only if they became symptomatic, ACS would be late or missed in 4%. With a strategy of withholding ACS for asymptomatic patients with a short but closed cervix, overall ACS for cervical insufficiency could be avoided or appropriately/postponed in 76% of patients. Accuracy < 14days to birth would have been achieved in 45% with ACS administered less than 48h prior to birth in 3%.

**Conclusion**: ACS treatment for asymptomatic cervical insufficiency is rarely given at the optimal time. To improve timing, we propose giving ACS only to patients with an open cervix and those with a closed short cervix who have additional clinical symptoms.

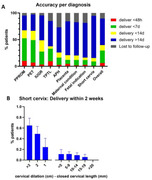

## 435 | Quantitative fetal fibronectin and QUiPP App safely reduces inappropriate steroid use in threatened preterm labour

Helena Watson^1^, Naomi Carlisle^2^, Andrew Shennan^2^

^1^King's College London, KIng, England; ^2^King's College London, KIng's College London, England

16:45–18:00

**Objective**: *T*o compare the ability of quantitiative fetal fibronectin (qfFN), and qfFN combined with the QUiPP app (a clinical decision‐making tool for preterm birth), with a treat‐all strategy for appropriate antenatal corticosteroid (ACS) administration for women in threatened preterm labour (TPTL)

**Study Design**: Post‐hoc analysis of secondary outcomes from 1495 women from 12 maternity units in the UK where qfFN is used in maternity triage protocols in the EQUIPTT trial (ISRCTN17846337). Appropriate steroid use was defined as within 7 days of delivery before 35 weeks’.

**Results**: When qfFN was used alone, 13.1% (196/1495) of women in TPTL were given ACS, compared to 11.2% (87/725) when qfFN and QUiPP risk of 5% within 7 days were used. Of those women receiving ACS, 18% (35/196) went on to deliver within seven days when qfFN used, vs 28% of women where fFN and QUiPP were used. If all women presenting in TPTL had been given steroids, only 4% would have needed them, meaning 96% of women would be unnecessarily exposed to the risks of steroids. In terms of “missed steroids”, using fFN, 8 women 4% (8/179) did not receive well‐timed steroids. For the majority (5/8) this was because they received steroids at a previous admission. There was only one such case (1/87) in the subset of women where fFN combined with QUiPP app, who had also had ACS previously.

**Conclusion**: Worldwide, women in TPTL are recommended (ACOG and NICE) to receive ACS, although the majority will not deliver imminently. This over‐treatment is often justified as to avoid missing those who really need them. This analysis shows that with qfFN and QUiPP app, ACS are better targeted to those who need them, minimising unnecessary exposure to the majority. The assumption that in a treat‐all group, no women would miss steroids is flawed: women would still miss appropriately timed ACS because of previous episodes of TPTL and unnecessary steroids, an impact which would be magnified in settings without qfFN.

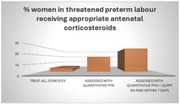

## 436 | The potential of advanced contrast‐enhanced ultrasound imaging for visualising the placental vasculature

Pascalle Wijntjes^1^, Sophie C.A. Dassen^2^, Luna Beijnsberger^3^, Ferenc Kandi^1^, Peiran Chen^1^, Jingchao Sui^1^, Myrthe van der Ven^1^, Judith O.E.H. van Laar^4^, Beatrijs (M.B.). Hout‐van der Jagt, van der^5^, Massimo Mischi^1^, simona Turco^1^, Loes Monen^6^

^1^Eindhoven University of Technology, Eindhoven, Noord‐Brabant; ^2^Máxima Medical Center, Tilburg, Noord‐Brabant; ^3^Máxima Medical Center, Veldhoven, Noord‐Brabant; ^4^Máxima Medical Centre, Veldhoven, The Netherlands, Veldhoven, Noord‐Brabant; ^5^Department of Obstetrics and Gynecology, Maxima Medical Center, Veldhoven, Eindhoven, Noord‐Brabant; ^6^Department of Gynaecology and Obstetrics, Máxima Medical Centre, Veldhoven, The Netherlands, Máxima Medical Centre, Noord‐Brabant

16:45–18:00

**Objective**: Diminished vascular remodelling in pregnancy can cause placental dysfunction, leading to complications like foetal growth restriction and pre‐eclampsia, common causes of perinatal and maternal morbidity and mortality. Currently, little is known about the feto‐placental microvasculature. Understanding this anatomy may offer insight into how to make an early diagnosis of placental dysfunction. Current ultrasound and Doppler techniques cannot probe these small vessels. Hence, this study investigates the potential of Contrast‐enhanced Ultrasound (CEUS) for visualising the placental (micro)vasculature, aiming to assess the feasibility as a future diagnostic tool.

**Study Design**: For testing the feasibility of this method and for lack of safety data with Ultrasound Contrast Agents (UCAs) in pregnancy, the study employed ex‐vivo placentas within three days after birth. Placentas were flushed with heparine and saline and perfused with SonoVue® (Bracco, Geneva) UCAs (1x10^6^ microbubbles/mL), through cannulated umbilical veins. The CEUS measurements were performed using a C10‐3V probe connected to a Philips EPIQ 7 ultrasound machine. Maximum intensity projection maps were derived from CEUS videos. Moreover, higher resolution (micro)vascular maps were created by ultrasound localization microscopy (ULM) based on the CEUS videos.

**Results**: Thirteen placentas were investigated, showing larger chorionic vessels during early perfusion and smaller villus vessels as perfusion continued. A distinction between central and peripheral vessels was observed. Bifurcations were more regularly and longitudinal in the central region, while vessels were more clustered peripherally. The obtained ULM maps are shown in Figure 1. Here, a part of the microvasculature is shown. Longitudinal vessels directed towards the basal plate are clearly visible. Also, some vessels directed outwards of the measurement plane are visible as red dots.

**Conclusion**: To conclude, CEUS is a promising diagnostic tool for visualising and assessing the macro‐ and microvasculature of the placenta.

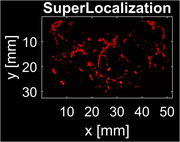

## 437 | Temporal trends in pregnancy‐related maternal mortality and preventability in Louisiana from 2011‐2020

James D. Toppin^1^, John A. Morgan^1^, Shannon McCloskey^2^, Joseph R. Biggio^1^, Frank B. Williams^2^, Jane Martin^2^

^1^Ochsner Health, New Orleans, Louisiana; ^2^Ochsner Clinic Foundation, New Orleans, Louisiana

16:45–18:00

**Objective**: Maternal mortality rates (MMR) in the United States have risen over the last decade, with Louisiana's among the highest in the US. Recent methodological criticisms suggest that expanding the definition of maternal deaths from 6 to 52 weeks postpartum and inclusion of the pregnancy checkbox on death‐certificates have falsely inflated MMRs. We aim to evaluate the Louisiana MMR trends following expansion of the postpartum definition to 52 weeks.

**Study Design**: We performed a cohort study of pregnancy‐related deaths reviewed by the Louisiana Pregnancy Associated Mortality Review Committee (PAMR) from 2011‐2020. PAMR investigates all deaths during pregnancy or in the postpartum period, determining pregnancy relatedness, cause, and preventability of death. Although capturing cases of maternal death depends on the death certificate pregnancy checkbox, methods for determining pregnancy relatedness do not. Prior to 2017, deaths were reviewed by PAMR only if they occurred ≤ 6 weeks after delivery. Since 2017, all deaths occurring within 52 weeks of delivery are reviewed by PAMR. The primary outcome was pregnancy‐related MMR, compared between 2011‐16 vs. 2017‐2020. Secondary outcomes were cause of death and preventability. Chi square test generated odds ratios (OR) with 95% confidence intervals (CI).

**Results**: From 2011‐2016, pregnancy‐related MMR was 12.4 deaths per 100,000 live births, of which 45.0% were preventable. From 2017‐2020, pregnancy‐related MMR increased to 24.9 deaths per 100,000 live births (OR 2.00 95% CI 1.00‐4.00), of which 83.5% were preventable (OR 6.07, 95% CI 2.49‐14.78). Cardiovascular disease was the most frequent cause of death in both periods (Figure).

**Conclusion**: Expanding the timeframe in which maternal deaths were defined from 6 to 52 weeks postpartum was associated with a two‐fold increase in pregnancy‐related MMR in Louisiana, suggesting that increases in MMR are not solely attributed to implementation of the pregnancy checkbox. Increases in the proportion of preventable deaths may represent opportunities for targeted policy interventions.

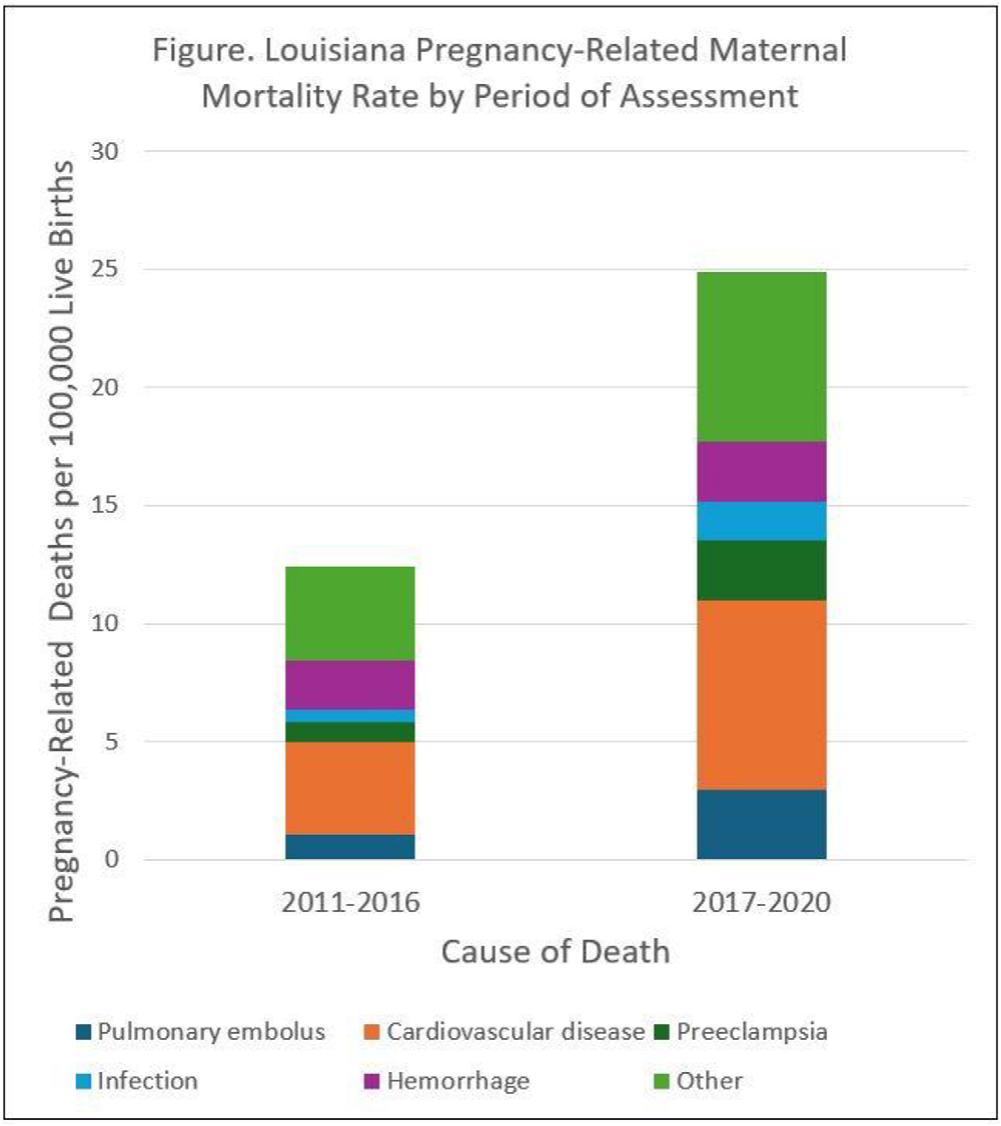

## 438 | Accuracy of ultrasound versus magnetic resonanace imaging in placenta accreta spectrum disorder diagnosis

Oxana M. Zarudskaya^1^, Shriya Devkumar. Das^1^, Kayla E. Ireland^1^, Patrick S. Ramsey^2^

^1^University of Texas Health Science Center at San Antonio, San Antonio, Texas; ^2^University of Texas Health Science Center San Antonio, San Antonio, Texas

16:45–18:00

**Objective**: Placenta Accreta Spectrum Disosrder (PASD) is a broad category describing abnormal adherence of the placenta beyond the uterine decidua. PASD is associated with a marked increase in maternal morbidity and mortality. Ultrasonography (US) is a primary diagnosis modality for PASD. It is controversial whether is an added benefit with magnetic resonance images (MRI) for accurate identification of PASD. The purpose of this study was to compare accuracy of US and MRI for prenatal diagnosis of PASD, including accuracy for the severity of PASD.

**Study Design**: This was a single center, retrospective cohort study at level IV maternal care facility with PASD Center of Excellence. We included all patients undergoing evaluation for PASD from January 2022 through December 2023. At our institution we universaly perform both imaging modalities (US and MRI) for patients with suspected PASD. Comparison of diagnostic tests was performed using the McNemar test in R.

**Results**: Of the total 49 patients included in this study, 36 (73.5%) had histologically confirmed PASD, while 13 (26.5%) did not have PASD. Of patients with PASD, 2 had placenta accreta (5%), 6 had placenta increta (17%) and 28 had placenta percreta (78%). In our cohort diagnostic accuracy was 79.6% with US and 78% with MRI (*p* = 0.86). US versus MRI sensitivity was 77.8% vs 90.3% (*p* = 0.327), specificity 84.6% vs 40% (*p* = 0.046), positive predictive value PPV 93.3% vs 82.4% (*p* = 0.192), negative predictive value 57.9% vs 57.1% (*p* = 0.972). Data presented in Tabl.1

**Conclusion**: Both imaging modalities US and MRI revealed similar sensitivity in prenatal diagnosis of PASD, in contrast to specificity where US showed better performance. Given the cost and limited availibility of MRI, US should be considered primary and only modality for prenatal diagnosis of PASD. Further studies are needed to compare performance of US and MRI in different histological categories and utility of MRI for surgical planning with extensive disease.

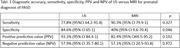

## 439 | Composite Postpartum Hemorrhage morbidity with and without stillbirth

Fabrizio Zullo^1^, Rachel L. Wiley^2^, Ipsita Ghose^3^, Antonella Giancotti^4^, Giuseppe Rizzo^5^, Daniele Di Mascio^6^, Hector M. Mendez‐Figueroa^7^, Suneet P. Chauhan^8^

^1^University of Rome La Sapienza, Rome, Lazio; ^2^University of California San Diego, San Diego, California; ^3^Baylor College of Medicine, Houston, Texas; ^4^Sapienza Università di Roma (ROMA), Rome, Lazio; ^5^Università Roma Sapienza, Università Roma La Sapienza, Lazio; ^6^Università Roma La Sapienza, Roma, Lazio; ^7^McGovern Medical School at UTHealth, Houston, Texas; ^8^Delaware Center of Maternal‐Fetal Medicine at Christiana Care, Delaware, Delaware

16:45–18:00

**Objective**: ACOG publications on stillbirth or postpartum hemorrhage (PPH) do not consider stillbirth as a risk factor for postpartum hemorrhagic morbidity. Our objective was to ascertain the likelihood of composite postpartum hemorrhagic morbidity (CPHM) among individuals that delivered vaginally with versus without a stillbirth.

**Study Design**: This was a retrospective cohort study of all parturients who delivered vaginally at a single level IV site within 2 years. Demographic differences and baseline PPH risks were analyzed. CPHM included any of the following: estimated blood loss ≥ 1000mL, use of uterotonics, Bakri balloon, surgical management of postpartum, blood transfusion, hysterectomy, thromboembolism, admission to the intensive care unit (ICU) or maternal death. Statistical analysis included Chi‐square, Kruskal‐Wallis, and Poisson regression with robust error variance for risk ratios, adjusting for gestational age (GA), bleeding on admission, chorioamnionitis, and prior uterine surgery.

**Results**: Of 4,386 consecutive vaginal births, 100 (2.2%) were stillbirths. Maternal age, marital status, GA at delivery, and PPH risk stratification at admission differed significantly. CPHM was significantly higher with a stillbirth delivery (39.0% vs 16.7%; aRR 1.67 95% CI 1.11‐2.53). The components of the CPHM that differed significantly between stillbirth vs. livebirths were: estimated blood loss ≥ 1000mL (12.3% vs. 2.3%; aRR 1.96 95% CI 1.04‐3.66), and ICU admission (5.0% vs. 0.2%; aRR 3.43 95% CI 1.38‐8.52). Use of uterotonic use (15.5 % vs 32.0%; RR 2.50 CI 95% 1.64‐3.80) and transfusion (1.9% vs 8.0%; RR 4.20 CI 95% 2.04‐8.65) were similar after adjustment.

**Conclusion**: Pregnancies complicated by stillbirth exhibit an increased risk of maternal hemorrhage, morbidity, and ICU admission compared to live births, even after adjusting for confounding factors. Early recognition and management of maternal hemorrhage risk with stillbirth cases may improve maternal outcomes.

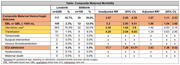

## 440 | Magnesium Sulfate and Prevention of Mortality/Cerebral Palsy among Preterm Births: A Systematic Review and Meta‐Analysis

Fabrizio Zullo^1^, Daniele Di Mascio^2^, Sara Sorrenti^3^, Teresa C. Logue^4^, Antonella Giancotti^3^, Giuseppe Rizzo^5^, Anthony C. Sciscione^4^, Matthew K. Hoffman^4^, Suneet P. Chauhan^6^

^1^University of Rome La Sapienza, Rome, Lazio; ^2^Università Roma La Sapienza, Roma, Lazio; ^3^Sapienza Università di Roma (ROMA), Rome, Lazio; ^4^Christiana Care Health System, Newark, Delaware; ^5^Università Roma Sapienza, Università Roma La Sapienza, Lazio; ^6^Delaware Center of Maternal‐Fetal Medicine at Christiana Care, Delaware, Delaware

16:45–18:00

**Objective**: Since the publication of MAGENTA trial (Crowther et al. JAMA 2023), systematic review and meta‐analysis (SR&MA) on magnesium sulphate (MgSO_4_) and fetal neuroprotection needs to be updated. (Wolf et al. BJOG 2020)

**Study Design**: The inclusion criteria were double blind randomized controlled trials (RCT) of individuals at risk of preterm birth who received either MgSO_4_ or placebo for neuroprotection. The co‐primary outcome were i) pediatric mortality; ii) death or moderate/severe cerebral palsy (M/S‐CP); secondary outcomes were death or any CP, MS‐CP or any CP (Table). We performed random‐effect head‐to‐head meta‐analyses to outcomes with MgSO_4_ or placebo, reporting summary risk ratio (RR) with 95% confidence interval (CI). Subgroup analyses were conducted for the primary outcomes by twins, GA < 28 weeks, loading dose and maintenance therapy. Number needed to treat (NNT), and 95% CI were calculated. Fragility index (FI) and Reverse Fragility Index (RFI) were determined. (PROSPERO #CRD42024526272).

**Results**: Electronic search identified 2087 abstracts, of which 5 trials (*n* = 5864) conducted in 5 countries met the inclusion criteria. The likelihood of pediatric mortality (7.9% vs 8.4%; *p* = 0.54; RR 0.95 [95% CI 0.79, 1.13]) or death + MS‐CP(9.9% vs 11.0% *p* = 0.28; RR 0.90 95% CI[10.74, 1.09]) did not differ between the groups.

Death or any CP was significantly reduced with MgSO_4_ (11.7%; 236/2015) than with placebo (14.0% [277/1973]; *p* = 0.02; RR 0.83, 95% CI 0.71‐0.97; heterogeneity 0%). The associated NNT was 43 (95% CI 23‐399). Among survivors, the rate of moderate or severe CP was also significantly lower with MgSO_4_ (2.2%; 66/2948) than with placebo (3.2% [96/2958]; *p* = 0.02; RR 0.69, 95% CI 0.50‐0.0.94; heterogeneity 0%). The associated NNT was 99 (95% CI 54‐575). Sub‐groups analysis confirms the findings.

**Conclusion**: Though the updated SR&MA indicates that the pediatric mortality alone or with moderate/severe CP are similar, it attests to the benefits of magnesium sulfate for prevention of cerebral palsy with preterm birth (i.e. NNT to avert preterm death or cerebral palsy is ∼45).

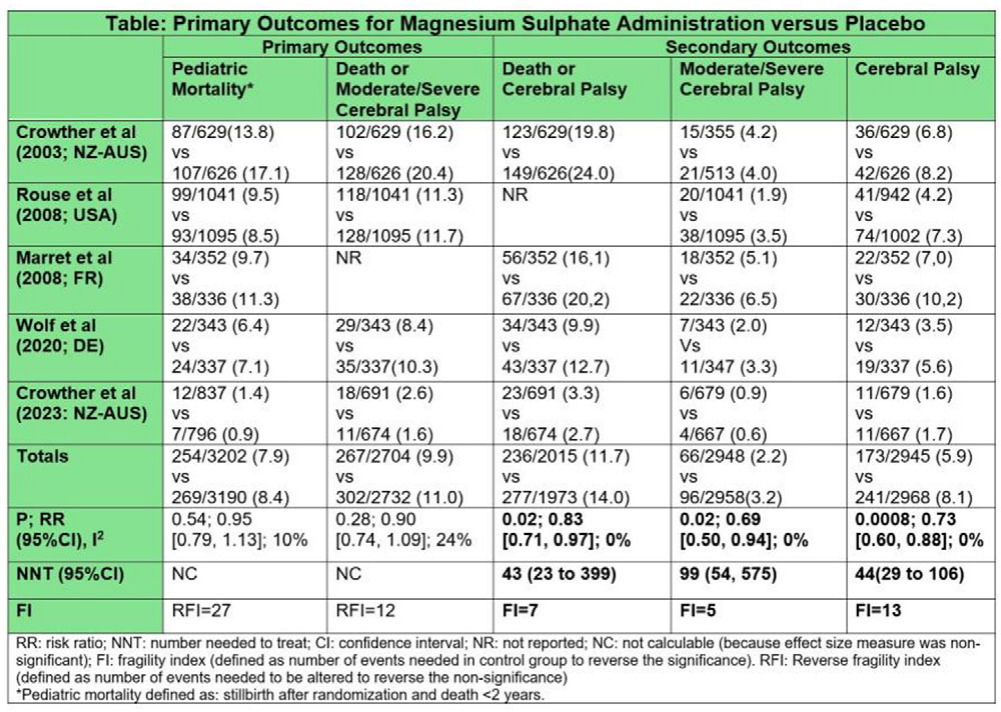

## 441 | US Delivery trends and pregnancy outcomes in Transgender and Gender‐Expansive individuals

Fabrizio Zullo^1^, Timothy Wen^2^, Daniele Di Mascio^3^, Vincenzo Berghella^4^, Suneet P. Chauhan^5^, Alexander M. Friedman^6^, Teresa C. Logue^7^

^1^University of Rome La Sapienza, Rome, Lazio; ^2^University of California, San Diego, Irvine, California; ^3^Università Roma La Sapienza, Roma, Lazio; ^4^Sidney Kimmel Medical College of Thomas Jefferson University, Philadelphia, Pennsylvania; ^5^Delaware Center of Maternal‐Fetal Medicine at Christiana Care, Delaware, Delaware; ^6^Columbia University Irving Medical Center, New York, New York; ^7^Christiana Care Health System, Newark, Delaware

16:45–18:00

**Objective**: Transgender and gender‐expansive individuals (TGE) represent a growing share of the US population. 1.6 million adults and youth identify as TGE in the US. Despite this demographic shift, there is scarcity of data regarding their pregnancy outcomes. Moreover, due to concerns about being misgendered or encountering an unsupportive environment, TGE individuals may be less inclined to seek prenatal care raising concerns on potential for heightened risks of pregnancy complications.

**Study Design**: We conducted a repeated cross‐sectional analysis of 51.9 million U.S. delivery hospitalizations in the Healthcare Cost and Utilization Project (HCUP)’s National Inpatient Sample during 2016‐2021. We identified deliveries to people with a gender identity disorder (GID) diagnosis or with stated gender different from expected gender for associated diagnosis and procedure codes (per HCUP). We described temporal trends in deliveries to TGE patients, reporting average annual percent change (AAPC). Using survey‐adjusted log‐linear regression, we calculated unadjusted risk ratios (RR) for the association between TGE status at delivery and risk for: hypertensive disorders of pregnancy (HDP); preterm delivery (PTD); abruption and antenatal hemorrhage (AAH), postpartum hemorrhage (PPH); postpartum infection (PPI); cesarean delivery (CD) and severe maternal morbidity (SMM), defined using a CDC composite excluding transfusion.

**Results**: The proportion of deliveries to TGE patients grew more than 50‐fold over 2016‐2021, rising from 1.2 to 58.7 per 1 million for an AAPC of 49.0% (95% CI 24.8%, 79.1%) (Figure). Delivery hospitalizations to TGE patients were at higher risk of CD (RR 1.17 95% CI 1.01, 1.34), HDP (RR 1.33 95% CI 1.07, 1.64), PPH (RR 1.62 95% CI 1.16, 2.26) with no difference in risk for PTD, PPI, AAH. SMM was suppressed due to the absence of events in the TGE group.

**Conclusion**: The present analysis shows a surge in deliveries to TGE patients over the analyzed period, reflecting the increasing presence of this demographic in childbirth. Our findings demonstrate that TGE patients are at elevated risk of CD, HDP and PPH.